# Supplementary material for: Automatic Identification of Players in the Flavonoid Biosynthesis with Application on the Biomedicinal Plant Croton tiglium
Source: Plants (Basel). 2020 Aug 27;9(9):1103. doi: 10.3390/plants9091103 (PMC7570183; doi:10.3390/plants9091103)
Supplement: Supplementary file 1 [file plants-09-01103-s001.zip › 20200724_supplements/File_S3.pdf]

```
                                                     η1
sp|P24481.1_PAL1_Petroselinum_crispum                                            TT        T
                                          1          10                          20
sp|P24481.1_PAL1_Petroselinum_crispum    ..ME.....N....GNGATTN...G....H....VN..GN.G..MD........FC.MKT
TRINITY_DN23351_c0_g1_i2                 ..ME.....F.CRKT....PN...G....N........AN.G...........WLG....
TRINITY_DN32981_c5_g1_i1                 ..MA.....K.I..........................GSFDD........LC.MT.
TRINITY_DN32981_c5_g1_i16                ..MA.....K.I..........................GSFDD........LC.MT.
TRINITY_DN32981_c5_g1_i9                 ..MA.....K.I..........................GSFDD........LC.MT.
TRINITY_DN32981_c5_g1_i17                ..MA.....K.I..........................GSFDD........LC.MT.
TRINITY_DN32981_c5_g1_i12                ..MA.....K.I..........................GSFDD........LC.MT.
TRINITY_DN32981_c5_g1_i5                 ..MA.....K.I..........................GSFDD........LC.MT.
TRINITY_DN32981_c5_g1_i13                ..MA.....K.I..........................GSFDD........LC.MT.
TRINITY_DN32981_c5_g1_i14                ..MA.....K.I..........................GSFDD........LC.MT.
TRINITY_DN23351_c0_g2_i1                 ..MAFE.........................................VC....
sp|P45728.1_PAL2_Petroselinum_crispum    ..ME.....N....GNGAITN...G....H....VN..GN.G..MD........FC.MKT
AWW24969.1_PAL_Lycoris_radiata           ..MA.....Y.A......NGN...G....NA..................NGFC.IL.
AIY24976.1_PAL_Mangifera_indica          ..ME.....F.CH......D...N............RN.G...........GLC.T..
AHY94892.1_PAL_Prunella_vulgaris         ..MA.....A.........EN...G...HH......ASN.G............FC.VKQ
AHA42443.1_PAL_Cannabis_sativa           ..ME.....Q.........KN...D................VSLES.......FC.VK.
AGL81344.1_PAL_Pyrus_communis            MEAE.....T.IT......QN...GKNGHH........QN.GAVES......P.LC.IK.
QIC54077.1_PAL_Paeonia_lactiflora        ..ME.....N.........GN...G....N..........GM..........SIC....
NP_181241.1_PAL1_Arabidopsis_thaliana    ..ME.....I..........N...GA...H......KSNGGGVDA.......MLC.GGD
NP_190894.1_PAL2_Arabidopsis_thaliana    ..MD.......................................QIEA.......MLC.GGG
NP_187645.1_PAL4_Arabidopsis_thaliana    ..MELC............NQN.......NH........................ITA
sp|Q42667.1_PAL_Citrus_limon             ..ME.....L.SHETCNGIKN...D............RN.GGTSS......LGLC.TG.
CAB42793.1_PAL_Citrus_clementina_x_Citrus_r ..MD.....RGAVI.....EN...G....H.......QN.GCLEG........LC....
CAB42794.1_PAL_Citrus_clementina_x_Citrus_r ..ME.....IGATT.....EN...G....H.......QN.GGLEG........LC....
sp|P27991.1_PAL_Glycine_max              ..ME.....A.........TN...G....H.......QN.G...........SFC.LST
sp|O49835.1_PAL1_Lithospermum_erythrorhizon ..ME.....T.IV......EN...G....N..........GKTME........FC....
sp|P35511.1_PAL1_Lycopersicon_esculentum ..............................................MD........LC.KKS
sp|P26600.1_PAL5_Lycopersicon_esculentum ..MA.....SSIV......QN...G....H.......VN.GEAMD........LC.KKS
sp|P25872.1_PAL1_Nicotiana_tabacum       ..MA.....S..........N...G....H.......VNGGENFE........LC.KKS
sp|P35513.2_PAL2_Nicotiana_tabacum       ..MA.....G.VA......QN...G....H.......QE....MD........FC.MK.
sp|P45733.1_PAL3_Nicotiana_tabacum       ..MA.....G.VA......QN...G....H.......QE....MD........FC.VK.
sp|P19142.1_PAL2_Phaseolus_vulgaris      ..MD.....A.T.......PN...G....K.......D.............AFV.VTA
sp|Q01861.1_PAL1_Pisum_sativum           ..ME.....T.VAAAITK.NN...G....Y.......E.............SFC.VTN
sp|Q04593.1_PAL2_Pisum_sativum           ..ME.....A.IGAAITK.NNS..G....Y.......D.............SFC.LTN
sp|P45731.1_PAL1_Populus_sieboldii_x_Populu ...........................................................
sp|P45730.1_PAL_Populus_trichocarpa      ..ME.....T.VT......KN...G....Y.......QN.GSLES........LCVNQ.
sp|Q43052.1_PAL2_Populus_sieboldii_x_Populu ..ME.....F.CQ.......D...S............RN.GNGSP........GFN.T..
AAF40223.1_PAL1_Rubus_idaeus             ..MEF.............YKN...G....N..........GAVES........FC....
AAF40224.1_PAL2_Rubus_idaeus             ..ME.....S.IT......QN...G...HHH...QNGIQN.GSLDD......G.LC.IKT
AAC78457.1_PAL_Prunus_avium              MATN.....S.IK......QN...G....H.......KN.GSVEL......PELC.IK.
sp|P31425.1_PAL1_Solanum_tuberosum       ..MA.....PSIA......QN...G....H.......VN.GEVEE......V.LW.KKS
sp|A2X7F7.1_PAL2_Oryza_sativa            ..ME...............CEN...G....RV......SAN.G.........MSGLC.VAA
sp|P52777.1_PAL_Pinus_taeda              ..MV.....AAAEIT...QANEV......QV.......KSTG............LC.TDF
ABU49842.1_PAL_Ginkgo_biloba             ..MV.....AGAERM...QSNPQNG..SQYV.......KSGGI.GD........LC.QSF
ACS28225.2_PAL_Pinus_massoniana          ..MV.....AAAEMT...QANEV......QV.......KSTG............LC.TDF
BAG74771.1_PAL_Ephedra_sinica            ..MV.....AGAEMA...QTAFV.....QHV.......KDGGIREF........LC.KGS
ABC69916.1_PAL_Brassica_napus            ..ME.....V..........N...GL............SHGGEVDA.......MLC.GGE
BAC56977.1_PAL_Daucus_carota             ...............MAYTN...G...HH....EN..GN.G..VD........LC.MKK
ABK24709.1_PAL_Picea_sitchensis          .MVA.....AAAEMA...QTNEV.....QQV.......KSTG............LC.TGL
AHA44840.1_PAL_Larix_kaempferi           ..MV.....AGAEIM...QTNEV.....QQV.......KSTG............LC.TSF
AGC23439.1_PAL_Dendrobium_candidum       ..ME.....H.V.......KGN...G....YA.......N.GAKAM......EGLC.LKG
ABI33979.1_PAL_Jatropha_curcas           ..MA.....T.II......GN...G....H.......QN.GSLEG........LC.IT.
XP_002326186.1_PAL_Populus_trichocarpa   ..ME.....T.IT......KN...G....Y.......QN.GSSES........LC.TQ.
XP_002519521.1_PAL_Ricinus_communis      ..MA.....A.MA......EN...G....S.......KN.DSLES........FCNMG.
XP_002268732.1_PAL_Vitis_vinifera        ..MD.....A.T........N...C....H.......GS.NKVKS........FC..V.
AFG26322.1_PAL_Cinnamomum_osmophloeum    ..ME.....T.T......QET...G....NV..................LQSFC.LK.
BAG70992.1_PAL_Musa_balbisiana           ..MEFAPKAQ.V......VEN...G............................EAFC.LKA
AAP34199.1_PAL_Phalaenopsis_x_Doritaenopsis ..ME.....V.S......KEN..........................................GLC.LQG
ACM61988.1_PAL_Lycoris_radiata           ..MA.....Y.A......NGN...G....NA..................NGFC.IL.
ABN79671.2_PAL_Rudbeckia_hirta           ..ME.....N......GNHVN...G....NH....VN..GN.GAAAE........FC.IK.
ACJ66297.1_PAL4_Nicotiana_tabacum        ..MA.....S..........N...G....H.......VNGGENFE........LC.KKS
ABG75911.1_PAL2_Nicotiana_attenuata      ..MA.....S..........N...G....H.......VNGGEDFE........LC.KKS
BAL49995.1_PAL_Eucalyptus_robusta        MEME.....S.TTGT....GN...GL...H..........G............LCATGS
ACM61988.1_PAL_Lycoris_radiata           ..MA.....Y.A......NGN...G....NA..................NGFC.IL.
BAM28963.1_PAL_Lilium_hybrid_division_I  ..MA.....H.IV...CENGN...G....FAATAADGNIN.GFIATAADGNSNGLC.LHA
                                    acc
```

```
                                                        α1           α2            β1        α3
sp|P24481.1_PAL1_Petroselinum_crispum       ............T
                                                        30        40        50        60        70
sp|P24481.1_PAL1_Petroselinum_crispum       ............EDPLYWGIAAEAMTGSHLDEVKKMVAEY.RKPVVKLGGETLTISQVAA
TRINITY_DN23351_c0_g1_i2                    .......FDV..NDPLNWGAVAESLKGSHLDEVKRMVKEY.RKPVVKLGGETLTIAQVAS
TRINITY_DN32981_c5_g1_i1                    ............RDPLNWGVAAESMKGSHFDEVKQMVSDF.RSPLVKLGGETLTVAQVAA
TRINITY_DN32981_c5_g1_i16                   ............RDPLNWGVAAESMKGSHFDEVKQMVSDF.RSPLVKLGGETLTVAQVAA
TRINITY_DN32981_c5_g1_i9                    ............RDPLNWGVAAESMKGSHFDEVKQMVSDF.RSPLVKLGGETLTVAQVAA
TRINITY_DN32981_c5_g1_i17                   ............LDPLNWGVAAESMKGSHFDEVKQMVRDF.RSPLVKLGGETLTVAQVAA
TRINITY_DN32981_c5_g1_i12                   ............LDPLNWGVAAESMKGSHFDEVKQMVRDF.RSPLVKLGGETLTVAQVAA
TRINITY_DN32981_c5_g1_i5                    ............RDPLNWGVAAESMKGSHFDEVKQMVSDF.RSPLVKLGGETLTVAQVAA
TRINITY_DN32981_c5_g1_i13                   ............RDPLNWGVAAESMKGSHFDEVKQMVSDF.RSPLVKLGGETLTVAQVAA
TRINITY_DN32981_c5_g1_i14                   ............LDPLNWGVAAESMKGSHFDEVKQMVRDF.RSPLVKLGGETLTVAQVAA
TRINITY_DN23351_c0_g2_i1                    ............QTVSHWQKAAELLQSSHFDEVQEMVSQFANAITVDIKGTSLTVAEVTA
sp|P45728.1_PAL2_Petroselinum_crispum       ............EDPLYWGIAAEAMTGSHLDEVKKMVAEY.RKPVVKLGGETLTISQVAA
AWW24969.1_PAL_Lycoris_radiata              .............DPLNWGAAAEALTGSHLDEVKRMVKDY.REAYVRLEGATLKVAQIAA
AIY24976.1_PAL_Mangifera_indica             ............SDPLNWNFAAESLKGSHLDEVKRMVEEF.RKPVVKLGGETLTIGQVTA
AHY94892.1_PAL_Prunella_vulgaris            ............SDPLNWGAAAESMSGSHLQEVKRMVEEF.RKPVVRLGGETLTISQVAA
AHA42443.1_PAL_Cannabis_sativa              ............GDPLNWGLAAESMSGSHLDEVKRMVAEY.RKPVVGLGGETLTISQVAA
AGL81344.1_PAL_Pyrus_communis               ............KDPLNWGLAADSLKGSHLDEVKRMVAEY.RKPVVKLGGESLTISQVAA
QIC54077.1_PAL_Paeonia_lactiflora           .........T..QDPLSWGVAAESLKGSHLDEVKRMVEEF.RKPVIRLAGKALTIAQVAG
NP_181241.1_PAL1_Arabidopsis_thaliana       IKTKNMVINA..EDPLNWGAAAEQMKGSHLDEVKRMVAEF.RKPVVNLGGETLTIGQVAA
NP_190894.1_PAL2_Arabidopsis_thaliana       EKTKVAVTTKTLADPLNWGLAADQMKGSHLDEVKKMVEEY.RRPVVNLGGETLTIGQVAA
NP_187645.1_PAL4_Arabidopsis_thaliana       VS..........GDPLNWNATAEALKGSHLDEVKRMVKEY.RKEAVKLGGETLTIGQVAA
sp|Q42667.1_PAL_Citrus_limon                ............TDPLNWTVAADSLKGSHLDEVKRMIDEY.RRPVVKLGGESLTIGQVTA
CAB42793.1_PAL_Citrus_clementina_x_Citrus_r .KDN..NYSS..GDALNWGVMAETLKGSHLEEVKRMVAEY.RKPVVNLGGETLTVAQVAA
CAB42794.1_PAL_Citrus_clementina_x_Citrus_r .KNNNYNYSS..GDALNWGVMAETLKGSHLEEVKRMVAEY.RKPVVNLGGETLTVAQVAA
sp|P27991.1_PAL_Glycine_max                 AKGNN........DPLNWGAAAEAMKGSHLDEVKRMVAEY.RKPVVRLGGETLTIAQVAA
sp|O49835.1_PAL1_Lithospermum_erythrorhizon .........M..KDPLNWEMASESMKGSHLDEVKNMVAEF.RKPVVQLAGKTLTIGQVAA
sp|P35511.1_PAL1_Lycopersicon_esculentum    I...........NDPLNWEMAADSLRGSHLDEVKKMVDEF.RKPIVKLGGETLSVAQVAS
sp|P26600.1_PAL5_Lycopersicon_esculentum    INV.........NDPLNWEMAAESLRGSHLDEVKKMVDEF.RKPIVKLGGETLTVAQVAS
sp|P25872.1_PAL1_Nicotiana_tabacum          A............DPLNWEMAAESLRGSHLDEVKKMVSEF.RKPMVKLGGESLTVAQVAA
sp|P35513.2_PAL2_Nicotiana_tabacum          ............VDPLNWEMAADSLKGSHLDEVKKMVAEF.RKPVVKLGGETLTVAQVAA
sp|P45733.1_PAL3_Nicotiana_tabacum          ............VDPLNWEMAADSLKGSHLDEVKKMVAEF.RKPVVKLGGETLTVAQVAA
sp|P19142.1_PAL2_Phaseolus_vulgaris         ANAAG........DPLNWAAAAEALSGSHLDEVKRMVAEY.RKPAVRLGGQTLTIAQVAA
sp|Q01861.1_PAL1_Pisum_sativum              AKNNNMKVNS..ADPLNWGVAAEAMKGSHLDEVKRMVEEY.RKPVVRLGGETLTISQVAA
sp|Q04593.1_PAL2_Pisum_sativum              AKNNNIKVSD..SDPLNWGVAAEAMKGSHLDEVKRMVDEY.RKPVVRLGGETLTISQVAA
sp|P45731.1_PAL1_Populus_sieboldii_x_Populu ...................MAADSLKGSHLDEVKRMIEEY.RNPVVKLGGETLTIGQVTA
sp|P45730.1_PAL_Populus_trichocarpa         ............RDPLSWGVAAEAMKGSHLDEVKRMVADY.RKPVVKLGGETLTIAQVAS
sp|Q43052.1_PAL2_Populus_sieboldii_x_Populu ............NDPLNWGMAAESLKGSHLDEVKRMIEEY.RNPVVKLGGETLTIGQVTA
AAF40223.1_PAL1_Rubus_idaeus                ........EG..HDPLNWNMAAESLKGSHVDELKRMVSDY.RKPVVKLGGETLTIGQVAA
AAF40224.1_PAL2_Rubus_idaeus                ESIKT.GYSV..SDPLNWGAAAESMTGSHLDEVRRMVAEY.RKPVVKLGGETLTISQVAA
AAC78457.1_PAL_Prunus_avium                 ............KDPLNWGVAAETLKGSHLDEVKRMVAEY.RKPVVKLGGESLTISQVAA
sp|P31425.1_PAL1_Solanum_tuberosum          I...........HDPLNWEMAVDSLRGSHLDEVKKMVDEF.RKPIVKLWGETLTVAQVAS
sp|A2X7F7.1_PAL2_Oryza_sativa               PR..........ADPLNWGKATEEMTGSHLDEVKRMVAEY.RQPLVKIEGASLRIAQVAA
sp|P52777.1_PAL_Pinus_taeda                 GSSG........SDPLNWVRAAKAMEGSHFEEVKAMVDSYFGAKEISIEGKSLTISDVAA
ABU49842.1_PAL_Ginkgo_biloba                .DST........TDPLNWARAAKALQGSHFEEVKQMVDSYFKSGEISIEGKTLTVADVTA
ACS28225.2_PAL_Pinus_massoniana             GSSG........SDPLNWVRAAKAMEGSHFEEVKAMVDSYLGVKEIFIEGKSLTISDVAA
BAG74771.1_PAL_Ephedra_sinica               .DSS........NDPLNWVAAAKSMSGSHFDMVRDMVEVYLNAKEVSIEGKTLTVAQVTA
ABC69916.1_PAL_Brassica_napus               IK.KNATVVG..ADPLNWGAAAEQMKGSHLDEVKRMVKEF.RRPVVNLGGETLTIGQVAA
BAC56977.1_PAL_Daucus_carota                ............EDPLSWGVAAEALKGSHLEEVKRMVAEY.RKPVVKLGGETLTISQVAA
ABK24709.1_PAL_Picea_sitchensis             SSSS........SDPLNWIRAAKAMEGSHFEEVKTMVDSYFESKEIFIEGKTLTIADVTA
AHA44840.1_PAL_Larix_kaempferi              GSST........SDPLNWIQAAKAMEGSHFEEVKSMVNSYLGSKEISIEGKSLTISDVAA
AGC23439.1_PAL_Dendrobium_candidum          ............RDQLGWAAAAKALEGSHLDEVKRMVKEF.RSPVVRLEGAELKISQVAA
ABI33979.1_PAL_Jatropha_curcas              ............RDPLSWGVAAESMKGSHLDEVKKMVSEY.RKPLVKLGGETLTVAQVAA
XP_002326186.1_PAL_Populus_trichocarpa      ............RDPLSWGVAAEAMKGSHLDEVKRMVAEY.RKPVVNLAGQTLTIAQVAS
XP_002519521.1_PAL_Ricinus_communis         ............RDPLSWGLAAESMKGSHLDEVKKMVAEY.RKPFVKLGGETLTVAQVAA
XP_002268732.1_PAL_Vitis_vinifera           ............SDPLNWGMAAETLKGSHLDEVKRMVAEY.RKPVVRLGGETLTISQVAA
AFG26322.1_PAL_Cinnamomum_osmophloeum       .............DPLNWEMAAESLKGSHLEEVKRMVDEF.RKPLIQLEGATLTISQVAA
BAG70992.1_PAL_Musa_balbisiana              .............DPLNWIKAAESLTGSHLDEVKRMVEEF.RKPLVRLEGATLTISQVAA
AAP34199.1_PAL_Phalaenopsis_x_Doritaenopsis ............HDPLNWGAAAAELQGSHLDEVKKMVEEF.RRPVVQLEGAKLKISQVAA
ACM61988.1_PAL_Lycoris_radiata              .............DPLNWGAAAEALTGSHLDEVKRMVKDY.REAYVKLEGATLKVAQIAA
ABN79671.2_PAL_Rudbeckia_hirta              .............DPLNWGAAAEALTGSHLDEVKKMVAEF.RKPVVKLGGETLTVSQVAG
ACJ66297.1_PAL4_Nicotiana_tabacum           SA..........TDPLNWEMAAESLRGSHLDEVKKMVSEF.RKPMVKLGGETLTVAQVAA
ABG75911.1_PAL2_Nicotiana_attenuata         SA..........TDPLNWEMASESLRGSHLDEVKKMVSEF.RKPMVKLGGETLTVAQVAA
BAL49995.1_PAL_Eucalyptus_robusta           RH..........ADPLNWGAAAAALTGSHLDEVKRMVEEY.RRPAVRLGGESLTIAQVAA
ACM61988.1_PAL_Lycoris_radiata              .............DPLNWGAAAEALTGSHLDEVKRMVKDY.REAYVKLEGATLKVAQIAA
BAM28963.1_PAL_Lilium_hybrid_division_I     .............DPLNWRAAAEAMSGSHLDEVRRMAAEF.RKPVVRLEGASLNISQVAA
                                        acc
```

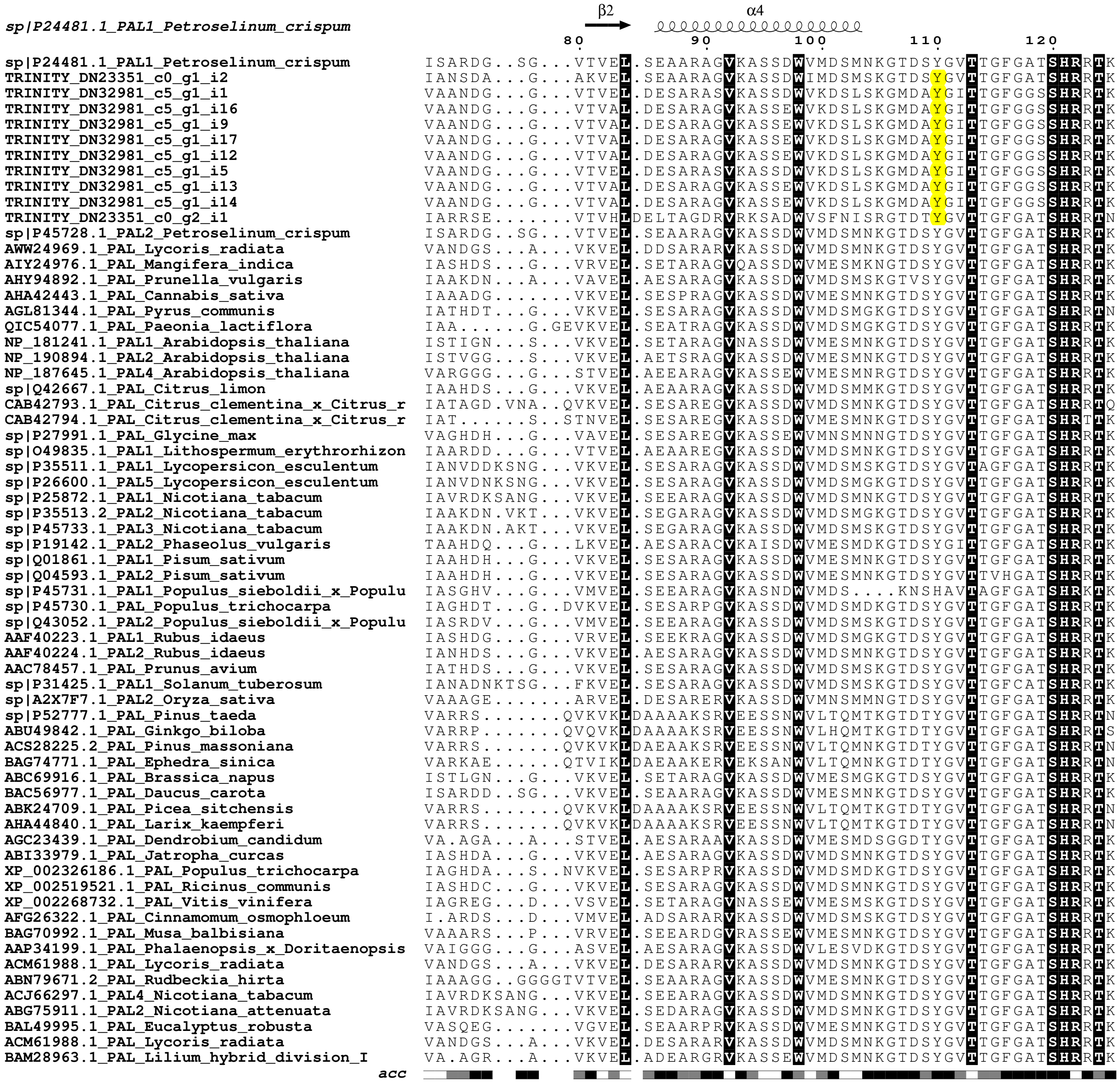

sp|P24481.1_PAL1_Petroselinum_crispum
β2
α4
80
90
100
110
120
sp|P24481.1_PAL1_Petroselinum_crispum ISARDG..SG...VTVEL.SEAARAGVKASSDWVMDSMNKGTDSYGVTTGFGATSHRRTK
TRINITY_DN23351_c0_g1_i2 IANSDA...G...AKVEL.SEAARAGVKASSDWIMDSMSKGTDSYGVTTGFGATSHRRTK
TRINITY_DN32981_c5_g1_i1 VAANDG...G...VTVEL.DESARASVKASSDWVKDSLSKGMDAYGITTGFGGSSHRRTK
TRINITY_DN32981_c5_g1_i16 VAANDG...G...VTVAL.DESARAGVKASSEWVKDSLSKGMDAYGITTGFGGSSHRRTK
TRINITY_DN32981_c5_g1_i9 VAANDG...G...VTVAL.DESARAGVKASSEWVKDSLSKGMDAYGITTGFGGSSHRRTK
TRINITY_DN32981_c5_g1_i17 VAANDG...G...VTVAL.DESARAGVKASSEWVKDSLSKGMDAYGITTGFGGSSHRRTK
TRINITY_DN32981_c5_g1_i12 VAANDG...G...VTVAL.DESARAGVKASSEWVKDSLSKGMDAYGITTGFGGSSHRRTK
TRINITY_DN32981_c5_g1_i5 VAANDG...G...VTVEL.DESARASVKASSDWVKDSLSKGMDAYGITTGFGGSSHRRTK
TRINITY_DN32981_c5_g1_i13 VAANDG...G...VTVAL.DESARAGVKASSEWVKDSLSKGMDAYGITTGFGGSSHRRTK
TRINITY_DN32981_c5_g1_i14 VAANDG...G...VTVAL.DESARAGVKASSEWVKDSLSKGMDAYGITTGFGGSSHRRTK
TRINITY_DN23351_c0_g2_i1 IARRSE......VTVHLDELTAGDRVRKSADWVSFNISRGTDTYGVTTGFGATSHRRTN
sp|P45728.1_PAL2_Petroselinum_crispum ISARDG..SG...VTVEL.SEAARAGVKASSDWVMDSMNKGTDSYGVTTGFGATSHRRTK
AWW24969.1_PAL_Lycoris_radiata VANDGS...A...VKVEL.DDSARARVKASSDWVMDSMNKGTDSYGVTTGFGATSHRRTK
AIY24976.1_PAL_Mangifera_indica IASHDS...G...VRVEL.SETARAGVQASSDWVMESMKNGTDSYGVTTGFGATSHRRTK
AHY94892.1_PAL_Prunella_vulgaris IAAKDN...A...VAVEL.AESARAGVKASSDWVMDSMSKGTVSYGVTTGFGATSHRRTK
AHA42443.1_PAL_Cannabis_sativa IAAADG......VKVEL.SESPRAGVKASSDWVMESMNKGTDSYGVTTGFGATSHRRTK
AGL81344.1_PAL_Pyrus_communis IATHDT...G...VKVEL.SESARAGVKASSDWVMDSMGKGTDSYGVTTGFGATSHRRTN
QIC54077.1_PAL_Paeonia_lactiflora IAA......G.GEVKVEL.SEATRAGVKASSDWVMDSMGKGTDSYGVTTGFGATSHRRTK
NP_181241.1_PAL1_Arabidopsis_thaliana ISTIGN...S...VKVEL.SETARAGVNASSDWVMESMNKGTDSYGVTTGFGATSHRRTK
NP_190894.1_PAL2_Arabidopsis_thaliana ISTVGG...S...VKVEL.AETSRAGVKASSDWVMESMNKGTDSYGVTTGFGATSHRRTK
NP_187645.1_PAL4_Arabidopsis_thaliana VARGGG...G...STVEL.AEEARAGVKASSEWVMESMNRGTDSYGVTTGFGATSHRRTK
sp|Q42667.1_PAL_Citrus_limon IAAHDS...G...VKVEL.AEAARAGVKASSDWVMDSMMKGTDSYGVTTGFGATSHRRTK
CAB42793.1_PAL_Citrus_clementina_x_Citrus_r IATAGD.VNA..QVKVEL.SESAREGVKASSDWVMDSMNKGTDSYGVTTGFGATSHRRTQ
CAB42794.1_PAL_Citrus_clementina_x_Citrus_r IAT......S..STNVEL.SESAREGVKASSDWVMESMNKGTDSYGVTTGFGATSHRTTK
sp|P27991.1_PAL_Glycine_max VAGHDH...G...VAVEL.SESAREGVKASSEWVMNSMNNGTDSYGVTTGFGATSHRRTK
sp|O49835.1_PAL1_Lithospermum_erythrorhizon IAARDD...G...VTVEL.AEAAREGVKASSDWVMDSMNKGTDSYGVTTGFGATSHRRTK
sp|P35511.1_PAL1_Lycopersicon_esculentum IANVDDKSNG...VKVEL.SESARAGVKASSDWVMDSMSKGTDSYGVTAGFGATSHRRTK
sp|P26600.1_PAL5_Lycopersicon_esculentum IANVDNKSNG...VKVEL.SESARAGVKASSDWVMDSMGKGTDSYGVTTGFGATSHRRTK
sp|P25872.1_PAL1_Nicotiana_tabacum IAVRDKSANG...VKVEL.SEEARAGVKASSDWVMDSMNKGTDSYGVTTGFGATSHRRTK
sp|P35513.2_PAL2_Nicotiana_tabacum IAAKDN.VKT...VKVEL.SEGARAGVKASSDWVMDSMGKGTDSYGVTTGFGATSHRRTK
sp|P45733.1_PAL3_Nicotiana_tabacum IAAKDN.AKT...VKVEL.SEGARAGVKASSDWVMDSMSKGTDSYGVTTGFGATSHRRTK
sp|P19142.1_PAL2_Phaseolus_vulgaris TAAHDQ...G...LKVEL.AESARACVKAISDWVMESMDKGTDSYGITTGFGATSHRRTK
sp|Q01861.1_PAL1_Pisum_sativum IAAHDH...G...VKVEL.SESARAGVKASSDWVMESMNKGTDSYGVTTGFGATSHRRTK
sp|Q04593.1_PAL2_Pisum_sativum IAAHDH...G...VKVEL.SESARAGVKASSDWVMESMNKGTDSYGVTTVHGATSHRRTK
sp|P45731.1_PAL1_Populus_sieboldii_x_Populu IASGHV...G...VMVEL.SEEARAGVKASNDWVMDS....KNSHAVTAGFGATSHRKTK
sp|P45730.1_PAL_Populus_trichocarpa IAGHDT...G..DVKVEL.SESARPGVKASSDWVMDSMDKGTDSYGVTTGFGATSHRRTK
sp|Q43052.1_PAL2_Populus_sieboldii_x_Populu IASRDV...G...VMVEL.SEEARAGVKASSDWVMDSMSKGTDSYGVTTGFGATSHRRTK
AAF40223.1_PAL1_Rubus_idaeus IASHDG...G...VRVEL.SEEKRAGVKASSDWVMDSMGKGTDSYGVTTGFGATSHRRTK
AAF40224.1_PAL2_Rubus_idaeus IANHDS...G...VKVEL.AESARAGVKASSDWVMDSMNKGTDSYGVTTGFGATSHRRTK
AAC78457.1_PAL_Prunus_avium IATHDS...G...VKVEL.SESARAGVKASSDWVMDSMSKGTDSYGVTTGFGATSHRRTK
sp|P31425.1_PAL1_Solanum_tuberosum IANADNKTSG...FKVEL.SESARAGVKASSDWVMDSMSKGTDSYGVTTGFCATSHRRTK
sp|A2X7F7.1_PAL2_Oryza_sativa VAAAGE......ARVEL.DESARERVKASSDWVMNSMMNGTDSYGVTTGFGATSHRRTK
sp|P52777.1_PAL_Pinus_taeda VARRS.......QVKVKLDAAAAKSRVEESSNWVLTQMTKGTDTYGVTTGFGATSHRRTN
ABU49842.1_PAL_Ginkgo_biloba VARRP.......QVQVKLDAAAAKSRVEESSNWVLHQMTKGTDTYGVTTGFGATSHRRTS
ACS28225.2_PAL_Pinus_massoniana VARRS.......QVKVKLDAEAAKSRVEESSNWVLTQMNKGTDTYGVTTGFGATSHRRTN
BAG74771.1_PAL_Ephedra_sinica VARKAE......QTVIKLDAEAAKERVEKSANWVLTQMNKGTDTYGVTTGFGATSHRRTN
ABC69916.1_PAL_Brassica_napus ISTLGN...G...VKVEL.SETARAGVKASSDWVMESMGKGTDSYGVTTGFGATSHRRTK
BAC56977.1_PAL_Daucus_carota ISARDD..SG...VKVEL.SEAARAGVKASSDWVMESMNKGTDSYGVTTGFGATSHRRTK
ABK24709.1_PAL_Picea_sitchensis VARRS.......QVKVKLDAAAAKSRVEESSNWVLTQMTKGTDTYGVTTGFGATSHRRTN
AHA44840.1_PAL_Larix_kaempferi VARRS.......QVKVKLDAAAAKSRVEESSNWVLTQMNKGTDTYGVTTGFGATSHRRTN
AGC23439.1_PAL_Dendrobium_candidum VA.AGA...A...STVEL.AESARAAVKASSDWVMESMDSGGDTYGVTTGFGATSHRRTK
ABI33979.1_PAL_Jatropha_curcas IASHDA...G...VKVEL.AESARAGVKASSDWVMDSMNKGTDSYGVTTGFGATSHRRTK
XP_002326186.1_PAL_Populus_trichocarpa IAGHDA...S..NVKVEL.SESARPRVKASSDWVMDSMDKGTDSYGVTTGFGATSHRRTK
XP_002519521.1_PAL_Ricinus_communis IASHDC...G...VKVEL.SESARAGVKASSDWVMDSMNKGTDSYGVTTGFGATSHRRTK
XP_002268732.1_PAL_Vitis_vinifera IAGREG...D...VSVEL.SETARAGVNASSEWVMESMSKGTDSYGVTTGFGATSHRRTK
AFG26322.1_PAL_Cinnamomum_osmophloeum I.ARDS...D...VMVEL.ADSARARVKASSDWVMDSMSKGTDSYGVTTGFGATSHRRTK
BAG70992.1_PAL_Musa_balbisiana VAAARS...P...VRVEL.SEEARDGVRASSEWVMESMNKGTDSYGVTTGFGATSHRRTK
AAP34199.1_PAL_Phalaenopsis_x_Doritaenopsis VAIGGG...G...ASVEL.AESARAGVKASSDWVLESVDKGTDSYGVTTGFGATSHRRTK
ACM61988.1_PAL_Lycoris_radiata VANDGS...A...VKVEL.DESARARVKASSDWVMDSMNKGTDSYGVTTGFGATSHRRTK
ABN79671.2_PAL_Rudbeckia_hirta IAAAGG..GGGGTVTVEL.SEAARAGVKASSDWVMESMNKGTDSYGVTTGFGATSHRRTK
ACJ66297.1_PAL4_Nicotiana_tabacum IAVRDKSANG...VKVEL.SEEARAGVKASSDWVMDSMNKGTDSYGVTTGFGATSHRRTK
ABG75911.1_PAL2_Nicotiana_attenuata IAVRDKSANG...VKVEL.SEDARAGVKASSDWVMDSMNKGTDSYGVTTGFGATSHRRTK
BAL49995.1_PAL_Eucalyptus_robusta VASQEG......VGVEL.SEAARPRVKASSDWVMESMNKGTDSYGVTTGFGATSHRRTK
ACM61988.1_PAL_Lycoris_radiata VANDGS...A...VKVEL.DESARARVKASSDWVMDSMNKGTDSYGVTTGFGATSHRRTK
BAM28963.1_PAL_Lilium_hybrid_division_I VA.AGR...A...VKVEL.ADEARGRVKASSEWVMDSMNKGTDSYGVTTGFGATSHRRTK
acc

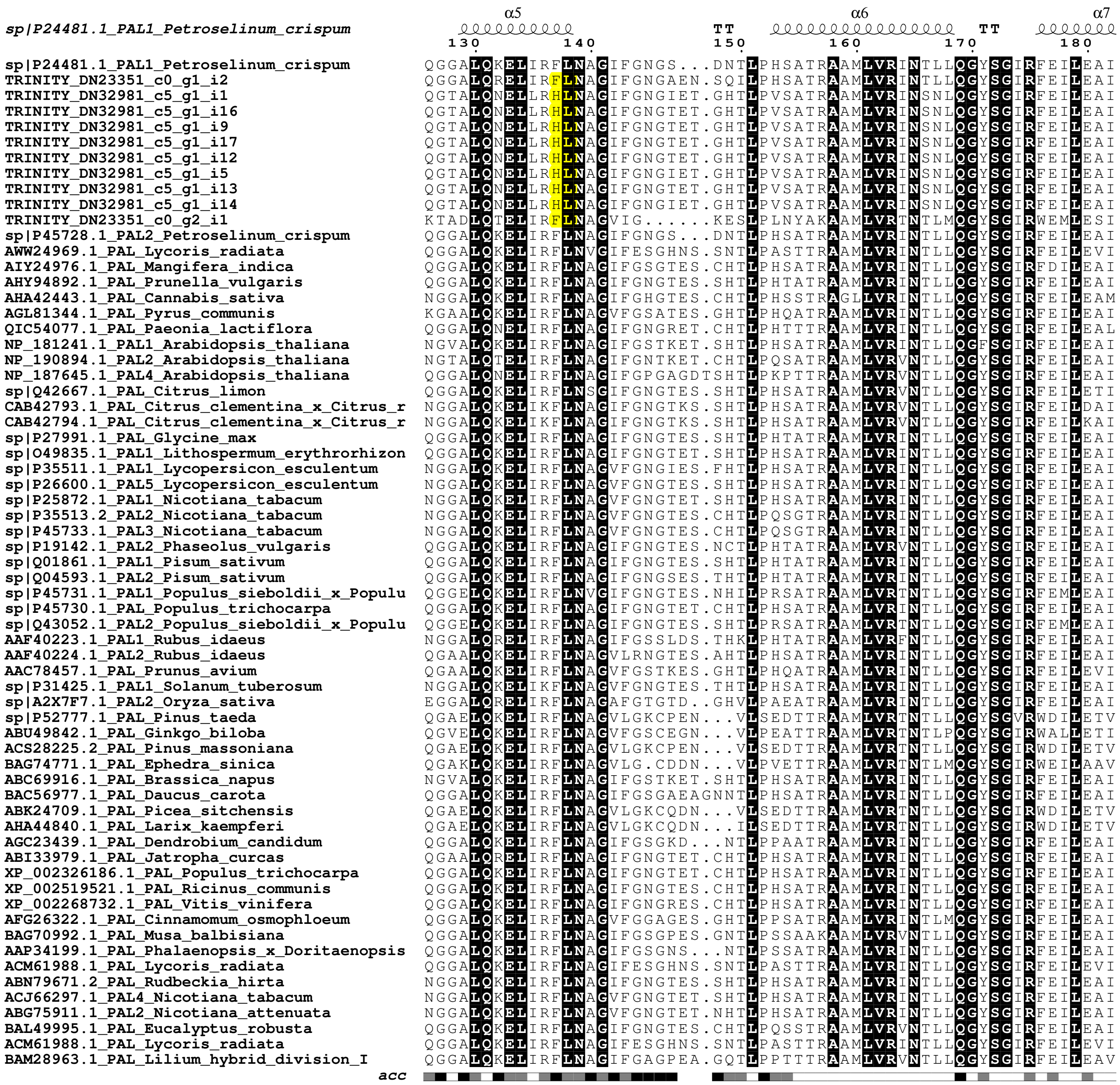
sp|P24481.1_PAL1_Petroselinum_crispum
α5
α6
α7
TT
TT
130
140
150
160
170
180
sp|P24481.1_PAL1_Petroselinum_crispum QGGALQKELIRFLNAGIFGNGS...DNTLPHSATRAAMLVRINTLLQGYSGIRFEILEAI
TRINITY_DN23351_c0_g1_i2 QGGALQRELIRFLNAGIFGNGAEN.SQILPHSATRAAMLVRINTLLQGYSGIRFEILEAI
TRINITY_DN32981_c5_g1_i1 QGTALQNELLRHLNAGIFGNGIET.GHTLPVSATRAAMLVRINSNLQGYSGIRFEILEAI
TRINITY_DN32981_c5_g1_i16 QGTALQNELLRHLNAGIFGNGTET.GHTLPVSATRAAMLVRINSNLQGYSGIRFEILEAI
TRINITY_DN32981_c5_g1_i9 QGTALQNELLRHLNAGIFGNGTET.GHTLPVSATRAAMLVRINSNLQGYSGIRFEILEAI
TRINITY_DN32981_c5_g1_i17 QGTALQNELLRHLNAGIFGNGTET.GHTLPVSATRAAMLVRINSNLQGYSGIRFEILEAI
TRINITY_DN32981_c5_g1_i12 QGTALQNELLRHLNAGIFGNGTET.GHTLPVSATRAAMLVRINSNLQGYSGIRFEILEAI
TRINITY_DN32981_c5_g1_i5 QGTALQNELLRHLNAGIFGNGIET.GHTLPVSATRAAMLVRINSNLQGYSGIRFEILEAI
TRINITY_DN32981_c5_g1_i13 QGTALQNELLRHLNAGIFGNGTET.GHTLPVSATRAAMLVRINSNLQGYSGIRFEILEAI
TRINITY_DN32981_c5_g1_i14 QGTALQNELLRHLNAGIFGNGIET.GHTLPVSATRAAMLVRINSNLQGYSGIRFEILEAI
TRINITY_DN23351_c0_g2_i1 KTADLQTELIRFLNAGVIG......KESLPLNYAKAAMLVRTNTLMQGYSGIRWEMLESI
sp|P45728.1_PAL2_Petroselinum_crispum QGGALQKELIRFLNAGIFGNGS...DNTLPHSATRAAMLVRINTLLQGYSGIRFEILEAI
AWW24969.1_PAL_Lycoris_radiata QGGALQKELIRFLNVGIFESGHNS.SNTLPASTTRAAMLVRINTLLQGYSGIRFEILEVI
AIY24976.1_PAL_Mangifera_indica QGGALQKELIRFLNAGIFGSGTES.CHTLPHSATRAAMLVRINTLLQGYSGIRFDILEAI
AHY94892.1_PAL_Prunella_vulgaris QGGALQKELIRFLNAGIFGNGTES.SHTLPHTATRAAMLVRINTLLQGYSGIRFEILEAI
AHA42443.1_PAL_Cannabis_sativa NGGALQKELIRFLNAGIFGHGTES.CHTLPHSSTRAGLLVRINTLLQGYSGIRFEILEAM
AGL81344.1_PAL_Pyrus_communis KGAALQKELIRFLNAGVFGSATES.GHTLPHQATRAAMLVRINTLLQGYSGIRFEILEAI
QIC54077.1_PAL_Paeonia_lactiflora QGGALQNELIRFLNAGIFGNGRET.CHTLPHTTTRAAMLVRINTLLQGYSGIRFEILEAL
NP_181241.1_PAL1_Arabidopsis_thaliana NGVALQKELIRFLNAGIFGSTKET.SHTLPHSATRAAMLVRINTLLQGFSGIRFEILEAI
NP_190894.1_PAL2_Arabidopsis_thaliana NGTALQTELIRFLNAGIFGNTKET.CHTLPQSATRAAMLVRVNTLLQGYSGIRFEILEAI
NP_187645.1_PAL4_Arabidopsis_thaliana QGGALQNELIRFLNAGIFGPGAGDTSHTLPKPTTRAAMLVRVNTLLQGYSGIRFEILEAI
sp|Q42667.1_PAL_Citrus_limon QGGALQKELIRFLNSGIFGNGTES.SHTLPHSATRAAMLVRVNTLLQGYSGIRFEILETI
CAB42793.1_PAL_Citrus_clementina_x_Citrus_r NGGALQKELIKFLNAGIFGNGTKS.SHTLPHSATRAAMLVRVNTLLQGYSGIRFEILDAI
CAB42794.1_PAL_Citrus_clementina_x_Citrus_r NGGALQKELIKFLNAGIFGNGTKS.SHTLPHSATRAAMLVRVNTLLQGYSGIRFEILKAI
sp|P27991.1_PAL_Glycine_max QGGALQKELIRFLNAGIFGNGTES.SHTLPHTATRAAMLVRINTLLQGYSGIRFEILEAI
sp|O49835.1_PAL1_Lithospermum_erythrorhizon QGGALQKELIRFLNAGIFGNGTET.SHTLPHSATRAAMLVRINTLLQGYSGIRFEILEAI
sp|P35511.1_PAL1_Lycopersicon_esculentum NGGALQKELIRFLNAGVFGNGIES.FHTLPHSATRAAMLVRINTLLQGYSGIRFEILEAI
sp|P26600.1_PAL5_Lycopersicon_esculentum NGGALQKELIRFLNAGVFGNGTES.SHTLPHSATRAAMLVRINTLLQGYSGIRFEILEAI
sp|P25872.1_PAL1_Nicotiana_tabacum NGGALQKELIRFLNAGVFGNGTET.SHTLPHSATRAAMLVRINTLLQGYSGIRFEILEAI
sp|P35513.2_PAL2_Nicotiana_tabacum NGGALQKELIRFLNAGVFGNGTES.CHTLPQSGTRAAMLVRINTLLQGYSGIRFEILEAI
sp|P45733.1_PAL3_Nicotiana_tabacum NGGALQKELIRFLNAGVFGNGTES.CHTLPQSGTRAAMLVRINTLLQGYSGIRFEILEAI
sp|P19142.1_PAL2_Phaseolus_vulgaris QGGALQKELIRFLNAGIFGNGTES.NCTLPHTATRAAMLVRVNTLLQGYSGIRFEILEAI
sp|Q01861.1_PAL1_Pisum_sativum QGGALQKELIRFLNAGIFGNGTES.SHTLPHTATRAAMLVRINTLLQGYSGIRFEILEAI
sp|Q04593.1_PAL2_Pisum_sativum QGGALQKELIRFLNAGIFGNGSES.THTLPHTATRAAMLVRINTLLQGYSGIRFEILEAI
sp|P45731.1_PAL1_Populus_sieboldii_x_Populu QGGELQKELIRFLNVGIFGNGTES.NHILPRSATRAAMLVRTNTLLQGYSGIRFEMLEAI
sp|P45730.1_PAL_Populus_trichocarpa QGGALQKELIRFLNAGIFGNGTET.CHTLPHSATRAAMLVRINTLLQGYSGIRFEILEAI
sp|Q43052.1_PAL2_Populus_sieboldii_x_Populu QGGELQKELIRFLNAGIFGNGTES.SHTLPRSATRAAMLVRTNTLLQGYSGIRFEMLEAI
AAF40223.1_PAL1_Rubus_idaeus NGGALQRELIRFLNAGIFGSSLDS.THKLPHTATRAAMLVRFNTLLQGYSGIRFEILEAI
AAF40224.1_PAL2_Rubus_idaeus QGAALQKELIRFLNAGVLRNGTES.AHTLPHSATRAAMLVRINTLLQGYSGIRFEILEAI
AAC78457.1_PAL_Prunus_avium QGAALQKELIRFLNAGVFGSTKES.GHTLPHQATRAAMLVRINTLLQGYSGIRFEILEVI
sp|P31425.1_PAL1_Solanum_tuberosum NGGALQKELIKFLNAGVFGNGTES.THTLPHSATRAAMLVRINTLLQGYSGIRFEILEAI
sp|A2X7F7.1_PAL2_Oryza_sativa EGGALQRELIRFLNAGAFGTGTD..GHVLPAEATRAAMLVRINTLLQGYSGIRFEILEAI
sp|P52777.1_PAL_Pinus_taeda QGAELQKELIRFLNAGVLGKCPEN...VLSEDTTRAAMLVRTNTLLQGYSGVRWDILETV
ABU49842.1_PAL_Ginkgo_biloba QGVELQKELIRFLNAGVFGSCEGN...VLPEATTRAAMLVRTNTLPQGYSGIRWALLETI
ACS28225.2_PAL_Pinus_massoniana QGAELQKELIRFLNAGVLGKCPEN...VLSEDTTRAAMLVRTNTLLQGYSGIRWDILETV
BAG74771.1_PAL_Ephedra_sinica QGAKLQKELIRFLNAGVLG.CDDN...VLPVETTRAAMLVRTNTLMQGYSGIRWEILAAV
ABC69916.1_PAL_Brassica_napus NGVALQKELIRFLNAGIFGSTKET.SHTLPHSATRAAMLVRINTLLQGYSGIRFEILEAI
BAC56977.1_PAL_Daucus_carota QGGALQKELIRFLNAGIFGSGAEAGNNTLPHSATRAAMLVRINTLLQGYSGIRFEILEAI
ABK24709.1_PAL_Picea_sitchensis QGAELQKELIRFLNAGVLGKCQDN...VLSEDTTRAAMLVRTNTLLQGYSGIRWDILETV
AHA44840.1_PAL_Larix_kaempferi QGAELQKELIRFLNAGVLGKCQDN...ILSEDTTRAAMLVRTNTLLQGYSGIRWDILETV
AGC23439.1_PAL_Dendrobium_candidum QGGALQKELIRFLNAGIFGSGKD...NTLPPAATRAAMLVRINTLLQGYSGIRFEILEAI
ABI33979.1_PAL_Jatropha_curcas QGAALQRELIRFLNAGIFGNGTET.CHTLPHSATRAAMLVRINTLLQGYSGIRFEILEAI
XP_002326186.1_PAL_Populus_trichocarpa QGGALQKELIRFLNAGIFGNGTET.CHTLPHSATRAAMLVRINTLLQGYSGIRFEILEAI
XP_002519521.1_PAL_Ricinus_communis QGGALQKELIRFLNAGIFGNGTES.CHTLPHSATRAAMLVRINTLLQGYSGIRFEILEAI
XP_002268732.1_PAL_Vitis_vinifera QGGALQKELIRFLNAGIFGNGRES.CHTLPHSATRAAMLVRINTLLQGYSGIRFEILEAI
AFG26322.1_PAL_Cinnamomum_osmophloeum QGGALQKELIRFLNAGVFGGAGES.GHTLPPSATRAAMLVRINTLMQGYSGIRFEILEAI
BAG70992.1_PAL_Musa_balbisiana QGGALQKELIRFLNAGIFGSGPES.GNTLPSSAAKAAMLVRVNTLLQGYSGIRFEILEAI
AAP34199.1_PAL_Phalaenopsis_x_Doritaenopsis QGGALQKELIRFLNAGIFGSGNS...NTLPSSATRAAMLVRINTLLQGYSGIRFEILEAI
ACM61988.1_PAL_Lycoris_radiata QGGALQKELIRFLNAGIFESGHNS.SNTLPASTTRAAMLVRINTLLQGYSGIRFEILEVI
ABN79671.2_PAL_Rudbeckia_hirta NGGALQKELIRFLNAGIFGNGTES.SHTLPHSATRAAMLVRINTLLQGYSGIRFEILEAI
ACJ66297.1_PAL4_Nicotiana_tabacum NGGALQKELIRFLNAGVFGNGTET.SHTLPHSATRAAMLVRINTLLQGYSGIRFEILEAI
ABG75911.1_PAL2_Nicotiana_attenuata NGGALQKELIRFLNAGVFGNGTET.NHTLPHSATRAAMLVRINTLLQGYSGIRFEILEAI
BAL49995.1_PAL_Eucalyptus_robusta QGGALQKELIRFLNAGIFGNGTES.CHTLPQSSTRAAMLVRVNTLLQGYSGIRFEILEAI
ACM61988.1_PAL_Lycoris_radiata QGGALQKELIRFLNAGIFESGHNS.SNTLPASTTRAAMLVRINTLLQGYSGIRFEILEVI
BAM28963.1_PAL_Lilium_hybrid_division_I QGGALQKELIRFLNAGIFGAGPEA.GQTLPPTTTRAAMLVRVNTLLQGYSGIRFEILEAV
acc

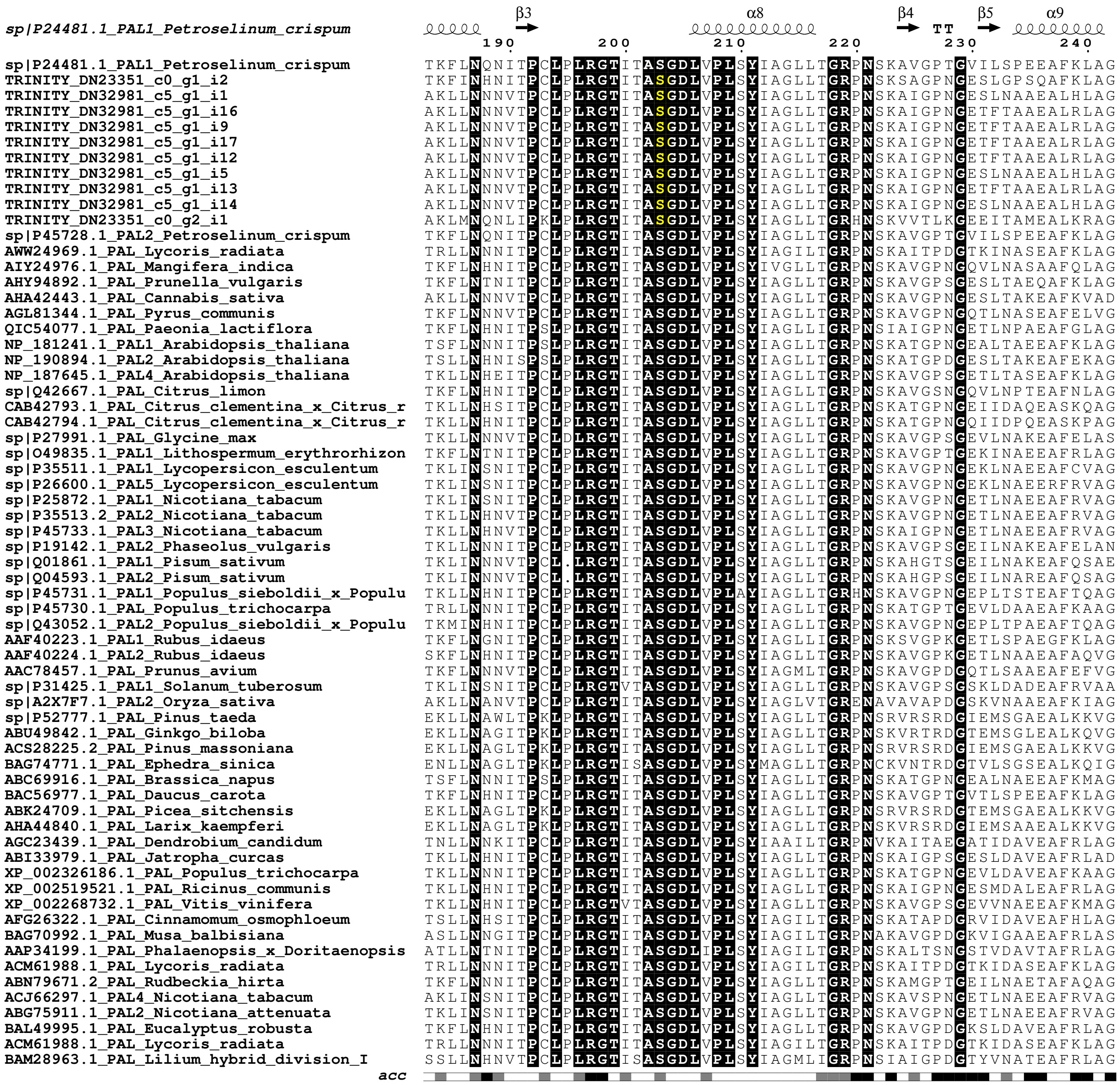

sp|P24481.1_PAL1_Petroselinum_crispum
β3
α8
β4
TT
β5
α9
190
200
210
220
230
240
sp|P24481.1_PAL1_Petroselinum_crispum TKFLNQNITPCLPLRGTITASGDLVPLSYIAGLLTGRPNSKAVGPTGVILSPEEAFKLAG
TRINITY_DN23351_c0_g1_i2 TKFINHNITPCLPLRGTITASGDLVPLSYIAGLLTGRPNSKSAGPNGESLGPSQAFKLAG
TRINITY_DN32981_c5_g1_i1 AKLLNNNVTPCLPLRGTITASGDLVPLSYIAGLLTGRPNSKAIGPNGESLNAAEALHLAG
TRINITY_DN32981_c5_g1_i16 AKLLNNNVTPCLPLRGTITASGDLVPLSYIAGLLTGRPNSKAIGPNGETFTAAEALRLAG
TRINITY_DN32981_c5_g1_i9 AKLLNNNVTPCLPLRGTITASGDLVPLSYIAGLLTGRPNSKAIGPNGETFTAAEALRLAG
TRINITY_DN32981_c5_g1_i17 AKLLNNNVTPCLPLRGTITASGDLVPLSYIAGLLTGRPNSKAIGPNGETFTAAEALRLAG
TRINITY_DN32981_c5_g1_i12 AKLLNNNVTPCLPLRGTITASGDLVPLSYIAGLLTGRPNSKAIGPNGETFTAAEALRLAG
TRINITY_DN32981_c5_g1_i5 AKLLNNNVTPCLPLRGTITASGDLVPLSYIAGLLTGRPNSKAIGPNGESLNAAEALHLAG
TRINITY_DN32981_c5_g1_i13 AKLLNNNVTPCLPLRGTITASGDLVPLSYIAGLLTGRPNSKAIGPNGETFTAAEALRLAG
TRINITY_DN32981_c5_g1_i14 AKLLNNNVTPCLPLRGTITASGDLVPLSYIAGLLTGRPNSKAIGPNGESLNAAEALHLAG
TRINITY_DN23351_c0_g2_i1 AKLMNQNLIPKLPLRGTITASGDLVPLSYIAGLLTGRHNSKVVTLKGEEITAMEALKRAG
sp|P45728.1_PAL2_Petroselinum_crispum TKFLNQNITPCLPLRGTITASGDLVPLSYIAGLLTGRPNSKAVGPTGVILSPEEAFKLAG
AWW24969.1_PAL_Lycoris_radiata TRLLNNNITPCLPLRGTITASGDLVPLSYIAGILTGRPNSKAITPDGTKINASEAFKLAG
AIY24976.1_PAL_Mangifera_indica TKFLNHNITPCLPLRGTITASGDLVPLSYIVGLLTGRPNSKAVGPNGQVLNASAAFQLAG
AHY94892.1_PAL_Prunella_vulgaris TKFLNTNITPCLPLRGTITASGDLVPLSYIAGLLTGRPNSKAVGPSGESLTAEQAFKLAG
AHA42443.1_PAL_Cannabis_sativa AKLLNNNVTPCLPLRGTITASGDLVPLSYIAGLLTGRPNSKAVGPNGESLTAKEAFKVAD
AGL81344.1_PAL_Pyrus_communis TKFLNNNVTPCLPLRGTITASGDLVPLSYIAGLLTGRPNSKAVGPNGQTLNASEAFELVG
QIC54077.1_PAL_Paeonia_lactiflora TKFLNHNITPSLPLRGTITASGDLVPLSYIAGLLIGRPNSIAIGPNGETLNPAEAFGLAG
NP_181241.1_PAL1_Arabidopsis_thaliana TSFLNNNITPSLPLRGTITASGDLVPLSYIAGLLTGRPNSKATGPNGEALTAEEAFKLAG
NP_190894.1_PAL2_Arabidopsis_thaliana TSLLNHNISPSLPLRGTITASGDLVPLSYIAGLLTGRPNSKATGPDGESLTAKEAFEKAG
NP_187645.1_PAL4_Arabidopsis_thaliana TKLLNHEITPCLPLRGTITASGDLVPLSYIAGLLTGRPNSKAVGPSGETLTASEAFKLAG
sp|Q42667.1_PAL_Citrus_limon TKFLNHNITPCLPLRGTITASGDLVPLSYIAGLLTGRPNSKAVGSNGQVLNPTEAFNLAG
CAB42793.1_PAL_Citrus_clementina_x_Citrus_r TKLLNHSITPCLPLRGTITASGDLVPLSYIAGLLTGRPNSKATGPNGEIIDAQEASKQAG
CAB42794.1_PAL_Citrus_clementina_x_Citrus_r TKLLNHNITPCLPLRGTITASGDLVPLSYIAGLLTGRPNSKATGPNGQIIDPQEASKPAG
sp|P27991.1_PAL_Glycine_max TKLLNNNVTPCLDLRGTITASGDLVPLSYIAGLLTGRPNSKAVGPSGEVLNAKEAFELAS
sp|O49835.1_PAL1_Lithospermum_erythrorhizon TKFLNTNITPCLPLRGTITASGDLVPLSYIAGLLTGRPNSKAVGPTGEKINAEEAFRLAG
sp|P35511.1_PAL1_Lycopersicon_esculentum TKLINSNITPCLPLRGTITASGDLVPLSYIAGLLTGRPNSKAVGPNGEKLNAEEAFCVAG
sp|P26600.1_PAL5_Lycopersicon_esculentum TKLINSNITPCLPLRGTITASGDLVPLSYIAGLLTGRPNSKAVGPNGEKLNAEERFRVAG
sp|P25872.1_PAL1_Nicotiana_tabacum TKLINSNITPCLPLRGTITASGDLVPLSYIAGLLTGRPNSKAVGPNGETLNAEEAFRVAG
sp|P35513.2_PAL2_Nicotiana_tabacum TKLLNHNVTPCLPLRGTITASGDLVPLSYIAGLLTGRPNSKAVGPNGETLNAEEAFRVAG
sp|P45733.1_PAL3_Nicotiana_tabacum TKLLNHNVTPCLPLRGTITASGDLVPLSYIAGLLTGRPNSKAIGPNGETLNAEEAFRVAG
sp|P19142.1_PAL2_Phaseolus_vulgaris TKLLNNNITPCLPLRGTITASGDLVPLSYIAGLLTGRPNSKAVGPSGEILNAKEAFELAN
sp|Q01861.1_PAL1_Pisum_sativum TKLINNNVTPCL.LRGTITASGDLVPLSYIAGLLTGRPNSKAHGTSGEILNAKEAFQSAE
sp|Q04593.1_PAL2_Pisum_sativum TKLINNNVTPCL.LRGTITASGDLVPLSYIAGLLTGRPNSKAHGPSGEILNAREAFQSAG
sp|P45731.1_PAL1_Populus_sieboldii_x_Populu TKLLNHNITPCLPLRGTITASGDLVPLAYIAGLLTGRHNSKAVGPNGEPLTSTEAFTQAG
sp|P45730.1_PAL_Populus_trichocarpa TRLLNNNITPCLPLRGTITASGDLVPLSYIAGLLTGRPNSKATGPTGEVLDAAEAFKAAG
sp|Q43052.1_PAL2_Populus_sieboldii_x_Populu TKMINHNITPCLPLRGTITASGDLVPLSYIAGLLTGRPNSKAVGPNGEPLTPAEAFTQAG
AAF40223.1_PAL1_Rubus_idaeus TKFLNGNITPCLPLRGTITASGDLVPLSYIAGLLIGRPNSKSVGPKGETLSPAEGFKLAG
AAF40224.1_PAL2_Rubus_idaeus SKFLNHNITPCLPLRGTITASGDLVPLSYIAGLLTGRPNSKAVGPKGETLNAAEAFAQVG
AAC78457.1_PAL_Prunus_avium TKFLNNNVTPCLPLRGTITASGDLVPLSYIAGMLTGRPNSKAVGPDGQTLSAAEAFEFVG
sp|P31425.1_PAL1_Solanum_tuberosum TKLINSNITPCLPLRGTVTASGDLVPLSYIAGLLTGRPNSKAVGPSGSKLDADEAFRVAA
sp|A2X7F7.1_PAL2_Oryza_sativa AKLLNANVTPCLPLRGTITASGDLVPLSYIAGLVTGRENAVAVAPDGSKVNAAEAFKIAG
sp|P52777.1_PAL_Pinus_taeda EKLLNAWLTPKLPLRGTITASGDLVPLSYIAGLLTGRPNSRVRSRDGIEMSGAEALKKVG
ABU49842.1_PAL_Ginkgo_biloba EKLLNAGITPKLPLRGTITASGDLVPLSYIAGLLTGRPNSKVRTRDGTEMSGLEALKQVG
ACS28225.2_PAL_Pinus_massoniana EKLLNAGLTPKLPLRGTITASGDLVPLSYIAGLLTGRPNSRVRSRDGIEMSGAEALKKVG
BAG74771.1_PAL_Ephedra_sinica ENLLNAGLTPKLPLRGTISASGDLVPLSYMAGLLTGRPNCKVNTRDGTVLSGSEALKQIG
ABC69916.1_PAL_Brassica_napus TSFLNNNITPSLPLRGTITASGDLVPLSYIAGLLTGRPNSKATGPNGEALNAEEAFKMAG
BAC56977.1_PAL_Daucus_carota TKFLNHNITPCLPLRGTITASGDLVPLSYIAGLLTGRPNSKAVGPTGVTLSPEEAFKLAG
ABK24709.1_PAL_Picea_sitchensis EKLLNAGLTPKLPLRGTITASGDLVPLSYIAGLLTGRPNSRVRSRDGTEMSGAEALKKVG
AHA44840.1_PAL_Larix_kaempferi EKLLNAGLTPKLPLRGTITASGDLVPLSYIAGLLTGRPNSKVRSRDGIEMSAAEALKKVG
AGC23439.1_PAL_Dendrobium_candidum TNLLNNKITPCLPLRGTITASGDLVPLSYIAAILTGRPNVKAITAEGATIDAVEAFRLAG
ABI33979.1_PAL_Jatropha_curcas TKLLNHNITPCLPLRGTITASGDLVPLSYIAGLLTGRPNSKAIGPSGESLDAVEAFRLAD
XP_002326186.1_PAL_Populus_trichocarpa TKLLNNNITPCLPLRGTITASGDLVPLSYIAGLLTGRPNSKATGPNGEVLDAVEAFKAAG
XP_002519521.1_PAL_Ricinus_communis TKLLNHNITPCLPLRGTITASGDLVPLSYIAGLLTGRPNSKAIGPNGESMDALEAFRLAG
XP_002268732.1_PAL_Vitis_vinifera TKLLNHNITPCLPLRGTVTASGDLVPLSYIAGLLTGRPNSKAVGPSGEVVNAEEAFKMAG
AFG26322.1_PAL_Cinnamomum_osmophloeum TSLLNHSITPCLPLRGTITASGDLVPLSYIAGILTGRPNSKATAPDGRVIDAVEAFHLAG
BAG70992.1_PAL_Musa_balbisiana ASLLNNGITPCLPLRGTITASGDLVPLSYIAGILTGRPNAKAVAPDGKVIGAAEAFRLAS
AAP34199.1_PAL_Phalaenopsis_x_Doritaenopsis ATLLNTNITPCLPLRGTITASGDLIPLSYIAGILTGRPNSKALTSNGSTVDAVTAFRLAG
ACM61988.1_PAL_Lycoris_radiata TRLLNNNITPCLPLRGTITASGDLVPLSYIAGILTGRPNSKAITPDGTKIDASEAFKLAG
ABN79671.2_PAL_Rudbeckia_hirta TKFLNNNITPCLPLRGTITASGDLVPLSYIAGLLTGRPNSKAMGPTGEILNAETAFAQAG
ACJ66297.1_PAL4_Nicotiana_tabacum AKLINSNITPCLPLRGTITASGDLVPLSYIAGLLTGRPNSKAVSPNGETLNAEEAFRVAG
ABG75911.1_PAL2_Nicotiana_attenuata TKLINSNITPCLPLRGTITASGDLVPLSYIAGLLTGRPNSKAVGPNGETLNAEEAFRVAG
BAL49995.1_PAL_Eucalyptus_robusta TKFLNHNITPCLPLRGTITASGDLVPLSYIAGLLTGRPNSKAVGPDGKSLDAVEAFRLAG
ACM61988.1_PAL_Lycoris_radiata TRLLNNNITPCLPLRGTITASGDLVPLSYIAGILTGRPNSKAITPDGTKIDASEAFKLAG
BAM28963.1_PAL_Lilium_hybrid_division_I SSLLNHNVTPCLPLRGTISASGDLVPLSYIAGMLIGRPNSIAIGPDGTYVNATEAFRLAG
acc

```
                                                        α10                    α11                  η2
sp|P24481.1_PAL1_Petroselinum_crispum
                                                 250        260       270       280       290       300
sp|P24481.1_PAL1_Petroselinum_crispum        VEGGFFELQPKEGLALVNGTAVGSGMASMVLFEANILAVLAEVMSAIFAEVMQGKPEFTD
TRINITY_DN23351_c0_g1_i2                     IVTGFFELQPKEGLALVNGTAVGSGLASMVLFEANVLAILAEVLSGIFAEVMQGKPEFTD
TRINITY_DN32981_c5_g1_i1                     IDTGFFELQPKEGLAMVNGTAVGSGLASMVLFEANILAVLSEVFSAIFAEVMNGKPEFTD
TRINITY_DN32981_c5_g1_i16                    VDSGFFELQPKEGLAMVNGTAVGSGLASMVLFEANILAVLSEVFSAIFAEVMNGKPEFTD
TRINITY_DN32981_c5_g1_i9                     VDSGFFELQPKEGLAMVNGTAVGSGLASMVLFEANILAVLSEVFSAIFAEVMNGKPEFTD
TRINITY_DN32981_c5_g1_i17                    VDSGFFELQPKEGLAMVNGTAVGSGLASMVLFEANILAVLSEVFSAIFAEVMNGKPEFTD
TRINITY_DN32981_c5_g1_i12                    VDSGFFELQPKEGLAMVNGTAVGSGLASMVLFEANILAVLSEVFSAIFAEVMNGKPEFTD
TRINITY_DN32981_c5_g1_i5                     IDTGFFELQPKEGLAMVNGTAVGSGLASMVLFEANILAVLSEVFSAIFAEVMNGKPEFTD
TRINITY_DN32981_c5_g1_i13                    VDSGFFELQPKEGLAMVNGTAVGSGLASMVLFEANILAVLSEVFSAIFAEVMNGKPEFTD
TRINITY_DN32981_c5_g1_i14                    IDTGFFELQPKEGLAMVNGTAVGSGLASMVLFEANILAVLSEVFSAIFAEVMNGKPEFTD
TRINITY_DN23351_c0_g2_i1                     IKSP.FELQAKEGLALVNGTAVGSAVAATVCFDANILAFLAEIVSALFCEVMHGKPEYTD
sp|P45728.1_PAL2_Petroselinum_crispum        VEGGFFELQPKEGLALVNGTAVGSGMASMVLFEANILAVLAEVMSAIFAEVMQGKPEFTD
AWW24969.1_PAL_Lycoris_radiata               ITDGFFQLQPKEGLALVNGTAVGSGLASTVLYDTNILAVLAEVLSAIFCEVMQGKPEFTD
AIY24976.1_PAL_Mangifera_indica              IGSGFFELQPKEGLALVNGTAVGSGLASTVLFEANILAILSEVLSAIFAEVMNGKPEFTD
AHY94892.1_PAL_Prunella_vulgaris             VSGGFFELQPKEGLALVNGTAVGSGLATMALYNANLLAVLSVVMSAVFAEVMNGKPEFTD
AHA42443.1_PAL_Cannabis_sativa               INSSFFELQPKEGLALVNGTAVGSGVASTVLFEANILAVLAEVLSAIFAEVIQGKPEFTD
AGL81344.1_PAL_Pyrus_communis                INSGFFELQPKEGLALVNGTAVGSGLASTVLFETNILALLAEILSAIFAEVMHGKPEFTD
QIC54077.1_PAL_Paeonia_lactiflora            INTGFFELQPKEGLALVNGTAVGSGLASMVLFEANILAILSEVLSGLFAEVMQGKPEFTD
NP_181241.1_PAL1_Arabidopsis_thaliana        ISSGFFDLQPKEGLALVNGTAVGSGMASMVLFETNVLSVLAEILSAVFAEVMSGKPEFTD
NP_190894.1_PAL2_Arabidopsis_thaliana        ISTGFFDLQPKEGLALVNGTAVGSGMASMVLFEANVQAVLAEVLSAIFAEVMSGKPEFTD
NP_187645.1_PAL4_Arabidopsis_thaliana        VSS.FFELQPKEGLALVNGTAVGSGLASTVLFDANILAVLSEVMSAMFAEVMQGKPEFTD
sp|Q42667.1_PAL_Citrus_limon                 VTSGFFELQPKEGLALVNGTAVGSGLAATVLFEANILAIMSEVLSAIFAEVMNGKPEFTD
CAB42793.1_PAL_Citrus_clementina_x_Citrus_r  F..GFFELQPKEGLALVNGTAVGSGLASMVLFDANNLALLSEILSAIFAEVMQGKPEFTD
CAB42794.1_PAL_Citrus_clementina_x_Citrus_r  F..GFFELQPKEGLALVNGTAVGSGLASMVLFEANNLALLSEILSAIFAEVMQGKPEFTD
sp|P27991.1_PAL_Glycine_max                  INSEFFELQPKEGLALVNGTAVGSGLASMVLFEANILAVLSEVLSAIFAEVMQGKPEFTD
sp|O49835.1_PAL1_Lithospermum_erythrorhizon  ISTGFFELQPKEGLALVNGTAVGSGMASMVLYEANILAVLSEVISAIFAEVMNGKPEFTD
sp|P35511.1_PAL1_Lycopersicon_esculentum     ISGGFFELQPKEGLALVNGTAVGSAMASIVLFESNIFAVMSEVLSAIFTEVMNGKPEFTD
sp|P26600.1_PAL5_Lycopersicon_esculentum     VTSGFFELQPKEGLALVNGTAVGSGMASMVLFESNILAVMSEVLSAIFAEVMNGKPEFTD
sp|P25872.1_PAL1_Nicotiana_tabacum           VNGGFFELQPKEGLALVNGTAVGSGMASMVLFDSNILAVMSEVLSAIFAEVMNGKPEFTD
sp|P35513.2_PAL2_Nicotiana_tabacum           VNGGFFELQPKEGLALVNGTAVGSGLASMVLFDANVLAVFSEVLSAIFAEVMNGKPEFTD
sp|P45733.1_PAL3_Nicotiana_tabacum           VNSGFFELQPKEGLALVNGTAVGSGLASMVLFDANILAVFSEVLSAIFAEVMNGKPEFTD
sp|P19142.1_PAL2_Phaseolus_vulgaris          IGSEFFELQPKEGLALVNGTAVGSGLASIVLFEANILAVLSEVISAIFAEVMQGKPEFTD
sp|Q01861.1_PAL1_Pisum_sativum               INDGFFELQPKEGLALVNGTAVGSGLASIVLFEANILAVLSEVLSAIFAEVMQGKPEFTD
sp|Q04593.1_PAL2_Pisum_sativum               INDGFFELQPKEGLALVNGTAVGSGLASIVLFEANILAVLSEVLSAIFAEVMQGKPEFTD
sp|P45731.1_PAL1_Populus_sieboldii_x_Populu  INGGFFELQPKEGLALVNGTAVGSGLASMVLFEANVLAILSEVLSAIFAEVMQGKPEFTD
sp|P45730.1_PAL_Populus_trichocarpa          IESGFFELQPKEGLALVNGTAVGSGLASMVLFETNVLAVLSELLSAIFAEVMNGKPEFTD
sp|Q43052.1_PAL2_Populus_sieboldii_x_Populu  IDGGFFELQPKEGLALVNGTAVGSGLASMVLFEANVLAILSEVLSAIFAEVMQGKPEFTD
AAF40223.1_PAL1_Rubus_idaeus                 IDGGFFELQPKEGLALVNGTAVGSGMASMVLFDANTLAVLSEVMSAIFAEVMQGKPEFTD
AAF40224.1_PAL2_Rubus_idaeus                 ISSGFFELQPKEGLALVNGTAVGSGLASTVLFETNILALLSEILSAIFAEVMQGKPEFTD
AAC78457.1_PAL_Prunus_avium                  INSGFFELQPKEGLALVNGTAVGSGLASTVLFDTNILALLSEILSAIFAEVMQGKPEFTD
sp|P31425.1_PAL1_Solanum_tuberosum           VSGGFFELQPKEGLALVNGTAVGSGMASIVLYDSNILAVMFEVLSAIFAEVMNGKPEFTD
sp|A2X7F7.1_PAL2_Oryza_sativa                IQGGFFELQPKEGLAMVNGTAVGSGLASTVLFEANILAILAEVLSAVFCEVMNGKPEYTD
sp|P52777.1_PAL_Pinus_taeda                  LEKP.FELQPKEGLAIVNGTSVGAALASIVCFDANVLALLSEVISAMFCEVMNGKPEFTD
ABU49842.1_PAL_Ginkgo_biloba                 LEKP.FELQPKEGLAIVNGTSVGAALASIVCFDANVLAVLSEVMSAMFCEVMNGKPEFTD
ACS28225.2_PAL_Pinus_massoniana              LEKP.FELQPKEGLAIVNGTSVGAALASIVCFDANVLALLSEVISAMFCEVMNGKPEFTD
BAG74771.1_PAL_Ephedra_sinica                IEKP.FELQPKEGLAIVNGTAVCAALASLVCFDANVLALLSEVMAAMFCEVMNGKPEFAD
ABC69916.1_PAL_Brassica_napus                VTSGFFDLQPKESLALVNGTAVGSGMASMVLFEANVLSVLAEVLSAVFAEVMSGKPEFTD
BAC56977.1_PAL_Daucus_carota                 VEGGFFELQPKEGLALVNGTAVGSGMASMVLFEANILAVLAEVMSAIFAEVMQGKPEFTD
ABK24709.1_PAL_Picea_sitchensis              LEKP.FELQPKEGLAIVNGTSVGAALASIVCFDANVLALLSEVISALFCEVMNGKPEFTD
AHA44840.1_PAL_Larix_kaempferi               VEKP.FELAPKEGLAIVNGTSVGAALASIVCFDANVLALLSEVISAMFCEVMNGKPEFTD
AGC23439.1_PAL_Dendrobium_candidum           ISGGFFELQPKEGLALVNGTAVGSGLASTVLFEANILSVMAEVLSAVFCEVMQGKPEYTD
ABI33979.1_PAL_Jatropha_curcas               IDSGFFELQPKEGLALVNGTAVGSGLASMVLFEANVLAVLSEILSAIFAEVMNGKPEFTD
XP_002326186.1_PAL_Populus_trichocarpa       IDSGFFELQPKEGLALVNGTAVGSGLASMVLFETNVLAVLSELISAIFAEVMNGKPEFTD
XP_002519521.1_PAL_Ricinus_communis          IESGFFELQPKEGLALVNGTAVGSGLASMVLFEANILAVLSEILSAIFAEVMNGKPEFTD
XP_002268732.1_PAL_Vitis_vinifera            IESGFFELQPKEGLALVNGTAVGSGLASMVLFETNVLAVLSEVLSAVFAEVMQGKPEFTD
AFG26322.1_PAL_Cinnamomum_osmophloeum        ITDGFFELQPKEGLALVNGTAVGSGLASMVLFEANVLSVLSEVISAIFCEVMQGKPEYTD
BAG70992.1_PAL_Musa_balbisiana               IADGFFELQPKEGLALVNGTAVGSGLASMVLFEANVLAVLSEVLSAVFCEVMQGKPEFTD
AAP34199.1_PAL_Phalaenopsis_x_Doritaenopsis  ISTGFFELQPKEGLALVNGTAVGSGLASLVLFETNILAIMAEVLSALFCEVMQGKPEYTD
ACM61988.1_PAL_Lycoris_radiata               ITDGFFQLQPKEGLALVNGTAVGSGLASTVLYDTNILAVLAEVLSAIFCEVMQGKPEFTD
ABN79671.2_PAL_Rudbeckia_hirta               VKGGFFELQPKEGLALVNGTAVGSGMASMVLFEANVLALLSEVLSAIFAEVMQGKPEFTD
ACJ66297.1_PAL4_Nicotiana_tabacum            VNGGFFELQPKEGLALVNGTAVGSGMASMVLFDSNILAVMSEVLSAIFAEVMNGKPEFTD
ABG75911.1_PAL2_Nicotiana_attenuata          VNGGFFELQPKEGLALVNGTAVGSGMASMVLFDSNILAVMSEVLSAIFAEVMNGKPEFTD
BAL49995.1_PAL_Eucalyptus_robusta            IDTGFFELQPKEGLALVNGTAVGSGLASIVLFEANILAVLSEVLSAIFAEVMQGKPEFTD
ACM61988.1_PAL_Lycoris_radiata               ITDGFFQLQPKEGLALVNGTAVGSGLASTVLYDTNILAVLAEVLSAIFCEVMQGKPEFTD
BAM28963.1_PAL_Lilium_hybrid_division_I      ISGGFFELQPKEGLALVNGTAVGSGLASMVLFEANILAVLAEVLSAIFCEVMQGKPEFTD
                                        acc
```

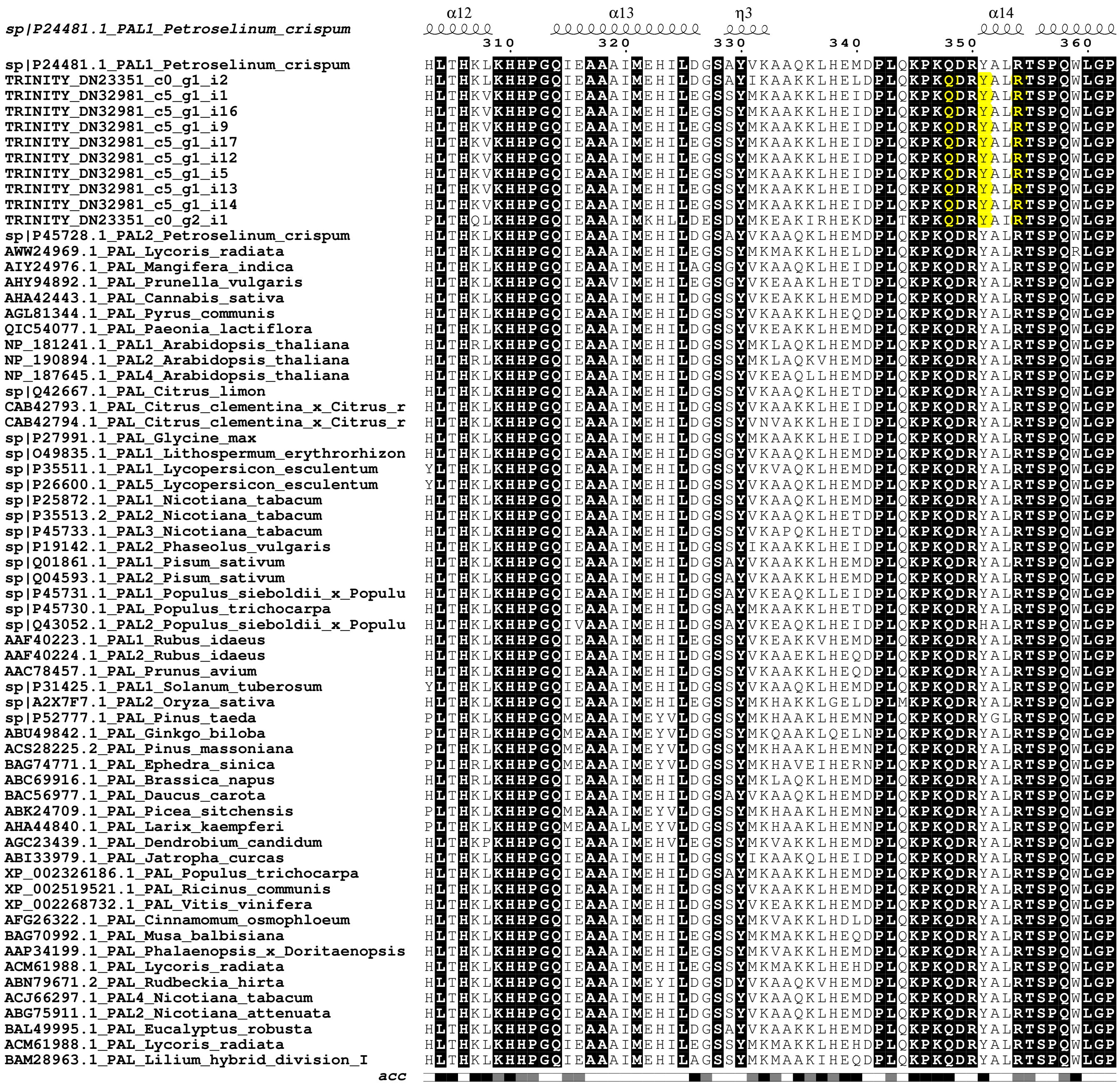

α12
α13
η3
α14
sp|P24481.1_PAL1_Petroselinum_crispum
310
320
330
340
350
360
sp|P24481.1_PAL1_Petroselinum_crispum
TRINITY_DN23351_c0_g1_i2
TRINITY_DN32981_c5_g1_i1
TRINITY_DN32981_c5_g1_i16
TRINITY_DN32981_c5_g1_i9
TRINITY_DN32981_c5_g1_i17
TRINITY_DN32981_c5_g1_i12
TRINITY_DN32981_c5_g1_i5
TRINITY_DN32981_c5_g1_i13
TRINITY_DN32981_c5_g1_i14
TRINITY_DN23351_c0_g2_i1
sp|P45728.1_PAL2_Petroselinum_crispum
AWW24969.1_PAL_Lycoris_radiata
AIY24976.1_PAL_Mangifera_indica
AHY94892.1_PAL_Prunella_vulgaris
AHA42443.1_PAL_Cannabis_sativa
AGL81344.1_PAL_Pyrus_communis
QIC54077.1_PAL_Paeonia_lactiflora
NP_181241.1_PAL1_Arabidopsis_thaliana
NP_190894.1_PAL2_Arabidopsis_thaliana
NP_187645.1_PAL4_Arabidopsis_thaliana
sp|Q42667.1_PAL_Citrus_limon
CAB42793.1_PAL_Citrus_clementina_x_Citrus_r
CAB42794.1_PAL_Citrus_clementina_x_Citrus_r
sp|P27991.1_PAL_Glycine_max
sp|O49835.1_PAL1_Lithospermum_erythrorhizon
sp|P35511.1_PAL1_Lycopersicon_esculentum
sp|P26600.1_PAL5_Lycopersicon_esculentum
sp|P25872.1_PAL1_Nicotiana_tabacum
sp|P35513.2_PAL2_Nicotiana_tabacum
sp|P45733.1_PAL3_Nicotiana_tabacum
sp|P19142.1_PAL2_Phaseolus_vulgaris
sp|Q01861.1_PAL1_Pisum_sativum
sp|Q04593.1_PAL2_Pisum_sativum
sp|P45731.1_PAL1_Populus_sieboldii_x_Populu
sp|P45730.1_PAL_Populus_trichocarpa
sp|Q43052.1_PAL2_Populus_sieboldii_x_Populu
AAF40223.1_PAL1_Rubus_idaeus
AAF40224.1_PAL2_Rubus_idaeus
AAC78457.1_PAL_Prunus_avium
sp|P31425.1_PAL1_Solanum_tuberosum
sp|A2X7F7.1_PAL2_Oryza_sativa
sp|P52777.1_PAL_Pinus_taeda
ABU49842.1_PAL_Ginkgo_biloba
ACS28225.2_PAL_Pinus_massoniana
BAG74771.1_PAL_Ephedra_sinica
ABC69916.1_PAL_Brassica_napus
BAC56977.1_PAL_Daucus_carota
ABK24709.1_PAL_Picea_sitchensis
AHA44840.1_PAL_Larix_kaempferi
AGC23439.1_PAL_Dendrobium_candidum
ABI33979.1_PAL_Jatropha_curcas
XP_002326186.1_PAL_Populus_trichocarpa
XP_002519521.1_PAL_Ricinus_communis
XP_002268732.1_PAL_Vitis_vinifera
AFG26322.1_PAL_Cinnamomum_osmophloeum
BAG70992.1_PAL_Musa_balbisiana
AAP34199.1_PAL_Phalaenopsis_x_Doritaenopsis
ACM61988.1_PAL_Lycoris_radiata
ABN79671.2_PAL_Rudbeckia_hirta
ACJ66297.1_PAL4_Nicotiana_tabacum
ABG75911.1_PAL2_Nicotiana_attenuata
BAL49995.1_PAL_Eucalyptus_robusta
ACM61988.1_PAL_Lycoris_radiata
BAM28963.1_PAL_Lilium_hybrid_division_I
acc

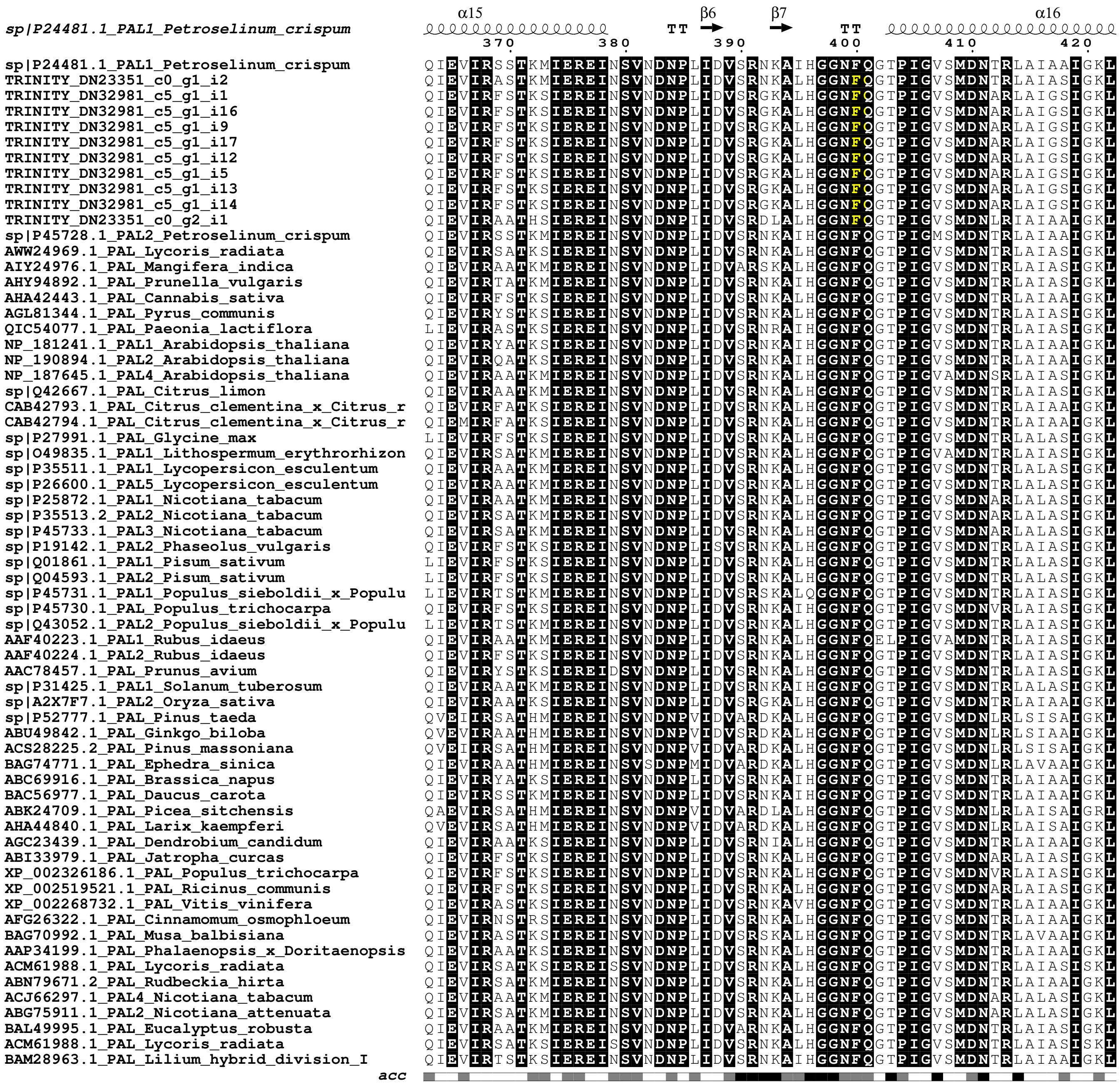

sp|P24481.1_PAL1_Petroselinum_crispum
α15
TT
β6
β7
TT
α16
370
380
390
400
410
420
sp|P24481.1_PAL1_Petroselinum_crispum
TRINITY_DN23351_c0_g1_i2
TRINITY_DN32981_c5_g1_i1
TRINITY_DN32981_c5_g1_i16
TRINITY_DN32981_c5_g1_i9
TRINITY_DN32981_c5_g1_i17
TRINITY_DN32981_c5_g1_i12
TRINITY_DN32981_c5_g1_i5
TRINITY_DN32981_c5_g1_i13
TRINITY_DN32981_c5_g1_i14
TRINITY_DN23351_c0_g2_i1
sp|P45728.1_PAL2_Petroselinum_crispum
AWW24969.1_PAL_Lycoris_radiata
AIY24976.1_PAL_Mangifera_indica
AHY94892.1_PAL_Prunella_vulgaris
AHA42443.1_PAL_Cannabis_sativa
AGL81344.1_PAL_Pyrus_communis
QIC54077.1_PAL_Paeonia_lactiflora
NP_181241.1_PAL1_Arabidopsis_thaliana
NP_190894.1_PAL2_Arabidopsis_thaliana
NP_187645.1_PAL4_Arabidopsis_thaliana
sp|Q42667.1_PAL_Citrus_limon
CAB42793.1_PAL_Citrus_clementina_x_Citrus_r
CAB42794.1_PAL_Citrus_clementina_x_Citrus_r
sp|P27991.1_PAL_Glycine_max
sp|O49835.1_PAL1_Lithospermum_erythrorhizon
sp|P35511.1_PAL1_Lycopersicon_esculentum
sp|P26600.1_PAL5_Lycopersicon_esculentum
sp|P25872.1_PAL1_Nicotiana_tabacum
sp|P35513.2_PAL2_Nicotiana_tabacum
sp|P45733.1_PAL3_Nicotiana_tabacum
sp|P19142.1_PAL2_Phaseolus_vulgaris
sp|Q01861.1_PAL1_Pisum_sativum
sp|Q04593.1_PAL2_Pisum_sativum
sp|P45731.1_PAL1_Populus_sieboldii_x_Populu
sp|P45730.1_PAL_Populus_trichocarpa
sp|Q43052.1_PAL2_Populus_sieboldii_x_Populu
AAF40223.1_PAL1_Rubus_idaeus
AAF40224.1_PAL2_Rubus_idaeus
AAC78457.1_PAL_Prunus_avium
sp|P31425.1_PAL1_Solanum_tuberosum
sp|A2X7F7.1_PAL2_Oryza_sativa
sp|P52777.1_PAL_Pinus_taeda
ABU49842.1_PAL_Ginkgo_biloba
ACS28225.2_PAL_Pinus_massoniana
BAG74771.1_PAL_Ephedra_sinica
ABC69916.1_PAL_Brassica_napus
BAC56977.1_PAL_Daucus_carota
ABK24709.1_PAL_Picea_sitchensis
AHA44840.1_PAL_Larix_kaempferi
AGC23439.1_PAL_Dendrobium_candidum
ABI33979.1_PAL_Jatropha_curcas
XP_002326186.1_PAL_Populus_trichocarpa
XP_002519521.1_PAL_Ricinus_communis
XP_002268732.1_PAL_Vitis_vinifera
AFG26322.1_PAL_Cinnamomum_osmophloeum
BAG70992.1_PAL_Musa_balbisiana
AAP34199.1_PAL_Phalaenopsis_x_Doritaenopsis
ACM61988.1_PAL_Lycoris_radiata
ABN79671.2_PAL_Rudbeckia_hirta
ACJ66297.1_PAL4_Nicotiana_tabacum
ABG75911.1_PAL2_Nicotiana_attenuata
BAL49995.1_PAL_Eucalyptus_robusta
ACM61988.1_PAL_Lycoris_radiata
BAM28963.1_PAL_Lilium_hybrid_division_I
acc

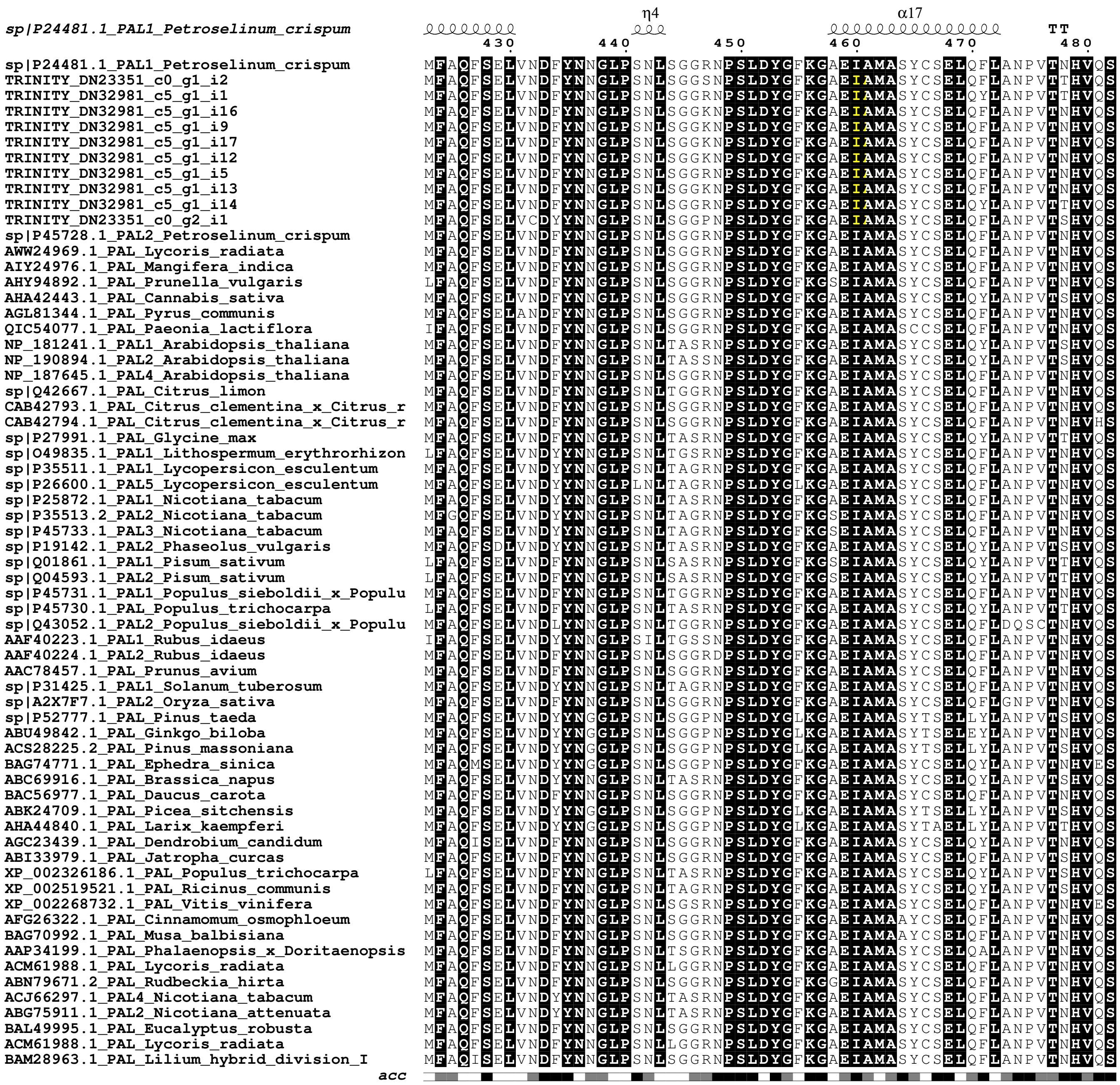
sp|P24481.1_PAL1_Petroselinum_crispum
η4
α17
TT
430
440
450
460
470
480
sp|P24481.1_PAL1_Petroselinum_crispum
TRINITY_DN23351_c0_g1_i2
TRINITY_DN32981_c5_g1_i1
TRINITY_DN32981_c5_g1_i16
TRINITY_DN32981_c5_g1_i9
TRINITY_DN32981_c5_g1_i17
TRINITY_DN32981_c5_g1_i12
TRINITY_DN32981_c5_g1_i5
TRINITY_DN32981_c5_g1_i13
TRINITY_DN32981_c5_g1_i14
TRINITY_DN23351_c0_g2_i1
sp|P45728.1_PAL2_Petroselinum_crispum
AWW24969.1_PAL_Lycoris_radiata
AIY24976.1_PAL_Mangifera_indica
AHY94892.1_PAL_Prunella_vulgaris
AHA42443.1_PAL_Cannabis_sativa
AGL81344.1_PAL_Pyrus_communis
QIC54077.1_PAL_Paeonia_lactiflora
NP_181241.1_PAL1_Arabidopsis_thaliana
NP_190894.1_PAL2_Arabidopsis_thaliana
NP_187645.1_PAL4_Arabidopsis_thaliana
sp|Q42667.1_PAL_Citrus_limon
CAB42793.1_PAL_Citrus_clementina_x_Citrus_r
CAB42794.1_PAL_Citrus_clementina_x_Citrus_r
sp|P27991.1_PAL_Glycine_max
sp|O49835.1_PAL1_Lithospermum_erythrorhizon
sp|P35511.1_PAL1_Lycopersicon_esculentum
sp|P26600.1_PAL5_Lycopersicon_esculentum
sp|P25872.1_PAL1_Nicotiana_tabacum
sp|P35513.2_PAL2_Nicotiana_tabacum
sp|P45733.1_PAL3_Nicotiana_tabacum
sp|P19142.1_PAL2_Phaseolus_vulgaris
sp|Q01861.1_PAL1_Pisum_sativum
sp|Q04593.1_PAL2_Pisum_sativum
sp|P45731.1_PAL1_Populus_sieboldii_x_Populu
sp|P45730.1_PAL_Populus_trichocarpa
sp|Q43052.1_PAL2_Populus_sieboldii_x_Populu
AAF40223.1_PAL1_Rubus_idaeus
AAF40224.1_PAL2_Rubus_idaeus
AAC78457.1_PAL_Prunus_avium
sp|P31425.1_PAL1_Solanum_tuberosum
sp|A2X7F7.1_PAL2_Oryza_sativa
sp|P52777.1_PAL_Pinus_taeda
ABU49842.1_PAL_Ginkgo_biloba
ACS28225.2_PAL_Pinus_massoniana
BAG74771.1_PAL_Ephedra_sinica
ABC69916.1_PAL_Brassica_napus
BAC56977.1_PAL_Daucus_carota
ABK24709.1_PAL_Picea_sitchensis
AHA44840.1_PAL_Larix_kaempferi
AGC23439.1_PAL_Dendrobium_candidum
ABI33979.1_PAL_Jatropha_curcas
XP_002326186.1_PAL_Populus_trichocarpa
XP_002519521.1_PAL_Ricinus_communis
XP_002268732.1_PAL_Vitis_vinifera
AFG26322.1_PAL_Cinnamomum_osmophloeum
BAG70992.1_PAL_Musa_balbisiana
AAP34199.1_PAL_Phalaenopsis_x_Doritaenopsis
ACM61988.1_PAL_Lycoris_radiata
ABN79671.2_PAL_Rudbeckia_hirta
ACJ66297.1_PAL4_Nicotiana_tabacum
ABG75911.1_PAL2_Nicotiana_attenuata
BAL49995.1_PAL_Eucalyptus_robusta
ACM61988.1_PAL_Lycoris_radiata
BAM28963.1_PAL_Lilium_hybrid_division_I
acc

```
                                                             α19
sp|P24481.1_PAL1_Petroselinum_crispum
                                          490       500       510       520       530       540
sp|P24481.1_PAL1_Petroselinum_crispum      AEQHNQDVNSLGLISSRKTSEAVEILKLMSTTFLVGLCQAIDLRHLEENLKSTVKNTVSS
TRINITY_DN23351_c0_g1_i2                   AEQHNQDVNSLGLISSRKTAEVVEILKLMSTTFLVALCQAIDLRHLEENLKNVVKNTISQ
TRINITY_DN32981_c5_g1_i1                   AEQHNQDVNSLGLISSRKTQEAVEILKLMSSTYLVALCQAIDLRHLEENLKNTVKTTVSQ
TRINITY_DN32981_c5_g1_i16                  AELHNQDVNSLGLISSRKTQEAVEILKLMSSTYLVALCQAIDLRHLEENLKHAVKNTVSQ
TRINITY_DN32981_c5_g1_i9                   AELHNQDVNSLGLISSRKTQEAIDILKLMSSTYLVALCQAIDLRHLEENLKHTVKNTVNQ
TRINITY_DN32981_c5_g1_i17                  AELHNQDVNSLGLISSRKTQEAIDILKLMSSTYLVALCQAIDLRHLEENLKHTVKNTVNQ
TRINITY_DN32981_c5_g1_i12                  AELHNQDVNSLGLISSRKTQEAVEILKLMSSTYLVALCQAIDLRHLEENLKHAVKNTVSQ
TRINITY_DN32981_c5_g1_i5                   AELHNQDVNSLGLISSRKTQEAVEILKLMSSTYLVALCQAIDLRHLEENMKHTVKNTVSQ
TRINITY_DN32981_c5_g1_i13                  AELHNQDVNSLGLISSRKTQEAIDILKLMSSTYLVALCQAIDLRHLEENLKNTVKTTVSQ
TRINITY_DN32981_c5_g1_i14                  AEQHNQDVNSLGLISSRKTQEAVEILKLMSSTYLVALCQAIDLRHLEENMKHTVKNTVSQ
TRINITY_DN23351_c0_g2_i1                   AEQHNQDVNSLGLISARKSAEAIEILKLMSATFMVALCQAIDLRHLEENMREVVKQIVRQ
sp|P45728.1_PAL2_Petroselinum_crispum      AEQHNQDVNSLGLISSRKTSEAVEILKLMSTTFLVGLCQAIDLRHLEENLKSTVKNTVSS
AWW24969.1_PAL_Lycoris_radiata             AEQHNQDVNSLGLISARKTEEAVQILKLMSTTFLVGLCQAIDLRHLEENLKSTVKTTISQ
AIY24976.1_PAL_Mangifera_indica            AEQHNQDVNSLGLISSRKTAEAVDILKLMSSTYLVALCQAIDLRHLEENLKNTVKNTVSQ
AHY94892.1_PAL_Prunella_vulgaris           AEQHNQDVNSLGLISSRKTVEAMDILKLMSSTYLVALCQAIDLRHLEENLKLAVKNTVSQ
AHA42443.1_PAL_Cannabis_sativa             AEQHNQDVNSLGLISSRKSAESIEILKLMSTTFLVALCQAIDLRHLEENLRHTVKKTVSL
AGL81344.1_PAL_Pyrus_communis              AEQHNQDVNSLGLISSRKTAEAVDILKLMSSTFLVALCQSVDLRHLEENLRNTVKNTVSQ
QIC54077.1_PAL_Paeonia_lactiflora          AEQHNQDVNSLGLISSRKTEEAVDILKLMSSTFLVALCQAIDLRHLEENMRNTVKNTVSQ
NP_181241.1_PAL1_Arabidopsis_thaliana      AEQHNQDVNSLGLISSRKTSEAVDILKLMSTTFLVAICQAVDLRHLEENLRQTVKNTVSQ
NP_190894.1_PAL2_Arabidopsis_thaliana      AEQHNQDVNSLGLISSRKTSEAVDILKLMSTTFLVGICQAVDLRHLEENLRQTVKNTVSQ
NP_187645.1_PAL4_Arabidopsis_thaliana      AEQHNQDVNSLGLISSRKTAEAVDILKLMSTTYLVALCQAVDLRHLEENLKKAVKSAVSQ
sp|Q42667.1_PAL_Citrus_limon               AEQHNQDVNSLGLNSSRKTAEAVDILKLMSSTFLVALCQAIDLRHLEENLKNTVKNTVSQ
CAB42793.1_PAL_Citrus_clementina_x_Citrus_r AEQHNQDVNSLGLISSRKTAEAVDILKLMSSTFLVALCQAIDLRHLEENLKHTVKDTVSQ
CAB42794.1_PAL_Citrus_clementina_x_Citrus_r AEQHNQDVNSLGLISSRKTAEAVDILKLMSSTFLVALCQAIDLRHLEENLKHTVKNTVSQ
sp|P27991.1_PAL_Glycine_max                AEQHNQDVNSLGLISSRKTNEAIEILKLMSSTFLIALCQAIDLRHLEENLKNSVKNTVSQ
sp|O49835.1_PAL1_Lithospermum_erythrorhizon AEQHNQDVNSLGLISSRKTSEAVEILKLMSSSFLVALFQAVDLRHIEENVRLAVKNTVSQ
sp|P35511.1_PAL1_Lycopersicon_esculentum   AEQHNQDVNSLGLISARKTAKAVDILKIMSSTYLVALCQAIDLRHLEENLKSVVKNTVSQ
sp|P26600.1_PAL5_Lycopersicon_esculentum   AEQHNQDVNSLGLISARKTAEAVDILKLMSSTYLVALCQAIDLRHLEENLKNAVKNTVSQ
sp|P25872.1_PAL1_Nicotiana_tabacum         AEQHNQDVNSLGLISARKTAEAVDILKLMSSTYLVALCQAIDLRHLEENLKNAVKNTVSQ
sp|P35513.2_PAL2_Nicotiana_tabacum         AEQHNQDVNSLDLISARKTAEAVDILKLMSSTYLVALCQAIDLRHLEENLRNAVKNTVSQ
sp|P45733.1_PAL3_Nicotiana_tabacum         AEQHNQDVNSLGLISARKTAEAVDILKLMSSTYLVALCQAIDLRHLEENLRNAVKNTVSQ
sp|P19142.1_PAL2_Phaseolus_vulgaris        AEQHNQDVNSLGLISSRKTNEALEILKLMSSTFLVALCQAIDLRHLEENLKNTVKNVVSQ
sp|Q01861.1_PAL1_Pisum_sativum             AEQHNQDVNSLGLISSRKTYEAIEILQLMSSTFLIALCQAVDLRHLEENLKNSVKNIVSQ
sp|Q04593.1_PAL2_Pisum_sativum             AEQHNQDVNSLGLISSRKTYEAIEILQLMSSTFLIALCQAIDLRHLEENLKNSVKNMVSH
sp|P45731.1_PAL1_Populus_sieboldii_x_Populu AEQHNQDVNSLGLISSRKTAEAVDILKLMSTTFLVGLCQAVDLRHIEENLKNTVKNTVSQ
sp|P45730.1_PAL_Populus_trichocarpa        AEQHNQDVNSLGLISSRKTAEAVDILKLMSTTFLVALCQAIDLRHLEENLKSAVKNTVSQ
sp|Q43052.1_PAL2_Populus_sieboldii_x_Populu AEQHNQDVNSLGLISSRKTAEAIDILKLMSTTFLVGLCHSVDLRHIEENLKNTVKISVSQ
AAF40223.1_PAL1_Rubus_idaeus               AEQHNQDVNSLGLISSRKTSEAVDILKLMSSTFLVALCQAIDLRHLEENLKIVVKTTVSN
AAF40224.1_PAL2_Rubus_idaeus               AEQHNQDVNSLGLISSRKTAEAVDILKLMSSTFLVALCQAIDLRHLEENLKSTVKNTVSQ
AAC78457.1_PAL_Prunus_avium                AEQHNQDVNSLGLISSRKTAEAVDILKLMSSTFLVALCQAIDLRHLEENLRNTVKNTVSQ
sp|P31425.1_PAL1_Solanum_tuberosum         AEQHNQDVNSLGLISARKTAEAVDILKLMSSTYLVALCQAIDLRHLEENLKSVVKNTVSQ
sp|A2X7F7.1_PAL2_Oryza_sativa              AEQHNQDVNSLGLISSRKTAEAIDILKLMSSTFLIALCQAVDLRHIEENVKSAVKSCVMT
sp|P52777.1_PAL_Pinus_taeda                AEQHNQDVNSLGLVSARKSAEAIDILKLMLSTYLTALCQAVDLRHLEENMLATVKQIVSQ
ABU49842.1_PAL_Ginkgo_biloba               AEQHNQDVNSLGLVSARKSAEALEILKLMLSTYLIALCQAVDLRHLEENMQATVKQVVAQ
ACS28225.2_PAL_Pinus_massoniana            AEQHNQDVNSLGLVSARKSAEAIDILKLMLSTYLTALCQAVDLRHLEENMLATVKQIVSQ
BAG74771.1_PAL_Ephedra_sinica              AEQHNQDVNSLGMVSARKSEEALEILRLMLSTYLTAICQAIDLRHLEENMLATVKQTVAQ
ABC69916.1_PAL_Brassica_napus              AEQHNQDVNSLGLISSRKTSEAVDILKLMSTTFLVGICQAIDLRHLEENLKQTVKNTVSQ
BAC56977.1_PAL_Daucus_carota               AEQHNQDVNSLGLISSRKTSEAVEILKLMSTTFLVGLCQAIDLRHLEENLKSTVKNTVSQ
ABK24709.1_PAL_Picea_sitchensis            AEQHNQDVNSLGLISARKSAEAVDILKLMLSTYLTALCQAVDLRHLEENMLATVKQIVSQ
AHA44840.1_PAL_Larix_kaempferi             AEQHNQDVNSLGLVSARKSAEAIDILKLMLSTYLTALCQAVDLRHLEENMLATVKQIVSQ
AGC23439.1_PAL_Dendrobium_candidum         AEQHNQDVNSLGLISSRKTAEAVEILKLMTSTFLVALCQAIDLRHLEENLKSAVKNTVSQ
ABI33979.1_PAL_Jatropha_curcas             AEQHNQDVNSLGLISSRKTQEAIDILKLMSSTFLVALCQAIDLRHLEENLKHAVKNTVTQ
XP_002326186.1_PAL_Populus_trichocarpa     AEQHNQDVNSLGLISSRKTAESVDILKLMSTTFLVALCQAIDLRHLEENLRSAVKNTVSH
XP_002519521.1_PAL_Ricinus_communis        AEQHNQDVNSLGLISSRKTAEAVDILKLMSTTYLVALCQAIDLRHLEENLRQAVKNTVSQ
XP_002268732.1_PAL_Vitis_vinifera          AEQHNQDVNSLGLISSRKTAEAVDILKLMSTTYLVALCQAIDLRHLEENLKSTVKKTVSH
AFG26322.1_PAL_Cinnamomum_osmophloeum      AEQHNQDVNSLGLISSRKTAEAVDILKLMSATYLVGLCQAIDLRHLEENLKSTVKNTVSQ
BAG70992.1_PAL_Musa_balbisiana             AEQHNQDVNSLGLISARKTAEAVDILKLMSTTYLVALCQAVDLRHLEENLKSAVKSTVSQ
AAP34199.1_PAL_Phalaenopsis_x_Doritaenopsis AEQHNQDVNSLGLISSRKTAESVDILKLMTTTFLVGLCQAVDLRHLEENLKNAVKNTVSQ
ACM61988.1_PAL_Lycoris_radiata             AEQHNQDVNSLGLISARKTEEAVQILKLMSTTFLVGLCQAIDLRHLEENLKSTVKTTISQ
ABN79671.2_PAL_Rudbeckia_hirta             AEQHNQDVNSLGLISARKTAEAVEILKLMSSTYLVALCQSVDLRHLEENMKSTVKNTVSQ
ACJ66297.1_PAL4_Nicotiana_tabacum          AEQHNQDVNSLGLISARKTAEAVDILKLMSSTYLVALCQAIDLRHLEENLKNAVKNTVSQ
ABG75911.1_PAL2_Nicotiana_attenuata        AEQHNQDVNSLGLISARKTAEAVDILKLMSSTYLVALCQAIDLRHLEENLKNAVKNTVSQ
BAL49995.1_PAL_Eucalyptus_robusta          AEQHNQDVNSLGLISSRKTAEAIDILKLMSSTFLVALCQAVDLRHLEENLKSTVKNTVGR
ACM61988.1_PAL_Lycoris_radiata             AEQHNQDVNSLGLISARKTEEAVQILKLMSTTFLVGLCQAIDLRHLEENLKSTVKTTISQ
BAM28963.1_PAL_Lilium_hybrid_division_I    AEQHNQDVNSLGLISSRKTAEAVEILKLMSSTFLVALCQAVDLRHLEENLKAAVKNTVSQ
                                       acc
```

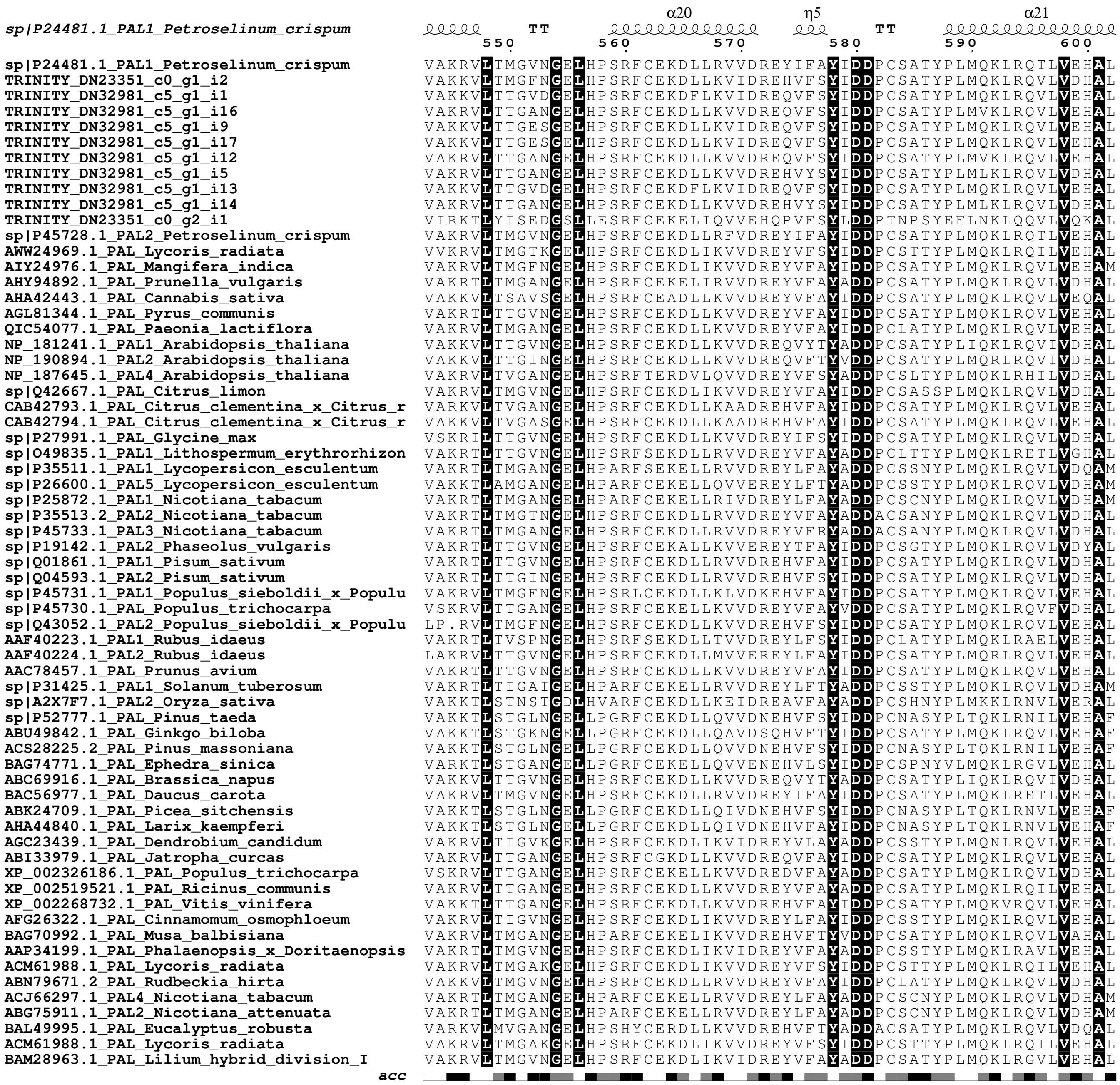

α20
η5
α21
sp|P24481.1_PAL1_Petroselinum_crispum
TT
TT
550
560
570
580
590
600
sp|P24481.1_PAL1_Petroselinum_crispum VAKRVLTMGVNGELHPSRFCEKDLLRVVDREYIFAYIDDPCSATYPLMQKLRQTLVEHAL
TRINITY_DN23351_c0_g1_i2 VAKKVLTMGFNGELHPSRFCEKDLLKVIDREHVFSYIDDPCSATYPLMQKLRQVLVDHAL
TRINITY_DN32981_c5_g1_i1 VAKKVLTTGVDGELHPSRFCEKDFLKVIDREQVFSYIDDPCSATYPLMQKLRQVLVEHAL
TRINITY_DN32981_c5_g1_i16 VAKRVLTTGANGELHPSRFCEKDLLKVVDREQVFSYIDDPCSATYPLMVKLRQVLVEHAL
TRINITY_DN32981_c5_g1_i9 VAKKVLTTGESGELHPSRFCEKDLLKVIDREQVFSYIDDPCSATYPLMQKLRQVLVEHAL
TRINITY_DN32981_c5_g1_i17 VAKKVLTTGESGELHPSRFCEKDLLKVIDREQVFSYIDDPCSATYPLMQKLRQVLVEHAL
TRINITY_DN32981_c5_g1_i12 VAKRVLTTGANGELHPSRFCEKDLLKVVDREQVFSYIDDPCSATYPLMVKLRQVLVEHAL
TRINITY_DN32981_c5_g1_i5 VAKRVLTTGANGELHPSRFCEKDLLKVVDREHVYSYIDDPCSATYPLMLKLRQVLVDHAL
TRINITY_DN32981_c5_g1_i13 VAKKVLTTGVDGELHPSRFCEKDFLKVIDREQVFSYIDDPCSATYPLMQKLRQVLVEHAL
TRINITY_DN32981_c5_g1_i14 VAKRVLTTGANGELHPSRFCEKDLLKVVDREHVYSYIDDPCSATYPLMLKLRQVLVDHAL
TRINITY_DN23351_c0_g2_i1 VIRKTLYISEDGSLLESRFCEKELIQVVEHQPVFSYLDDPTNPSYEFLNKLQQVLVQKAL
sp|P45728.1_PAL2_Petroselinum_crispum VAKRVLTMGVNGELHPSRFCEKDLLRFVDREYIFAYIDDPCSATYPLMQKLRQTLVEHAL
AWW24969.1_PAL_Lycoris_radiata VVKRVLTMGTKGELHPSRFCEKDLIKVVDREYVFSYIDDPCSTTYPLMQKLRQILVEHAL
AIY24976.1_PAL_Mangifera_indica VAKRVLTMGFNGELHPSRFCEKDLIKVVDREYVFAYIDDPCSATYPLMQKLRQVLVEHAM
AHY94892.1_PAL_Prunella_vulgaris VAKRTLTMGANGELHPSRFCEKELIRVVDREYVFAYADDPCSATYPLMQKLRQVLVDHAL
AHA42443.1_PAL_Cannabis_sativa VAKKVLTSAVSGELHPSRFCEADLLKVVDREYVFAYIDDPCSATYPLMQKLRQVLVEQAL
AGL81344.1_PAL_Pyrus_communis VAKRTLTTGVNGELHPSRFCEKDLLKVVDREYVFAYIDDPCSATYPLMQKLRQVLVEHAL
QIC54077.1_PAL_Paeonia_lactiflora VAKRVLTMGANGELHPSRFCEKDLLKVVDREYVFAYIDDPCLATYPLMQKLRQVLVEHAL
NP_181241.1_PAL1_Arabidopsis_thaliana VAKKVLTTGVNGELHPSRFCEKDLLKVVDREQVYTYADDPCSATYPLIQKLRQVIVDHAL
NP_190894.1_PAL2_Arabidopsis_thaliana VAKKVLTTGINGELHPSRFCEKDLLKVVDREQVFTYVDDPCSATYPLMQRLRQVIVDHAL
NP_187645.1_PAL4_Arabidopsis_thaliana VAKRVLTVGANGELHPSRFTERDVLQVVDREYVFSYADDPCSLTYPLMQKLRHILVDHAL
sp|Q42667.1_PAL_Citrus_limon VAKRVLTMGVNGELHPSRFCEKDLIKVVDREYVFAYIDDPCSASSPLMQKLRQVLVDHAL
CAB42793.1_PAL_Citrus_clementina_x_Citrus_r VARKVLTVGANGELHPSRFCEKDLLKAADREHVFAYIDDPCSATYPLMQKLRQVLVEHAL
CAB42794.1_PAL_Citrus_clementina_x_Citrus_r VAKKVLTVGASGELHPSRFCEKDLLKAADREHVFAYIDDPCSATYPLMQKLRQVLVEHAL
sp|P27991.1_PAL_Glycine_max VSKRILTTGVNGELHPSRFCEKDLLKVVDREYIFSYIDDPCSATYPLMQKLRQVLVDHAL
sp|O49835.1_PAL1_Lithospermum_erythrorhizon VAKRTLTTGVNGELHPSRFSEKDLLRVVDREYVFAYADDPCLTTYPLMQKLRETLVGHAL
sp|P35511.1_PAL1_Lycopersicon_esculentum VAKRTLTMGANGELHPARFSEKELLRVVDREYLFAYADDPCSSNYPLMQKLRQVLVDQAM
sp|P26600.1_PAL5_Lycopersicon_esculentum VAKKTLAMGANGELHPARFCEKELLQVVEREYLFTYADDPCSSTYPLMQKLRQVLVDHAM
sp|P25872.1_PAL1_Nicotiana_tabacum VAKRTLTMGANGELHPARFCEKELLRIVDREYLFAYADDPCSCNYPLMQKLRQVLVDHAM
sp|P35513.2_PAL2_Nicotiana_tabacum VAKRTLTMGTNGELHPSRFCEKDLLRVVDREYVFAYADDACSANYPLMQKLRQVLVDHAL
sp|P45733.1_PAL3_Nicotiana_tabacum VAKRTLTMGANGELHPSRFCEKDLLRVVDREYVFRYADDACSANYPLMQKLRQVLVDHAL
sp|P19142.1_PAL2_Phaseolus_vulgaris VAKRTLTTGVNGELHPSRFCEKALLKVVEREYTFAYIDDPCSGTYPLMQKLRQVLVDYAL
sp|Q01861.1_PAL1_Pisum_sativum VAKRTLTTGVNGELHPSRFCEKDLLRVVDREHVFAYIDDPCSATYPLMQKLRQVLVDHAL
sp|Q04593.1_PAL2_Pisum_sativum VAKRTLTTGINGELHPSRFCEKDLLRVVDREHVFSYIDDPCSATYPLMQKLRQVLVDHAL
sp|P45731.1_PAL1_Populus_sieboldii_x_Populu VAKRVLTMGFNGELHPSRLCEKDLLKLVDKEHVFAYIDDPCSATYPLMQKLRQVLVEHAL
sp|P45730.1_PAL_Populus_trichocarpa VSKRVLTTGANGELHPSRFCEKELLKVVDREYVFAYVDDPCSATYPLMQKLRQVFVDHAL
sp|Q43052.1_PAL2_Populus_sieboldii_x_Populu LP.RVLTMGFNGELHPSRFCEKDLLKVVDREHVFSYIDDPCSATYPLMQKLRQVLVEHAL
AAF40223.1_PAL1_Rubus_idaeus VAKRTLTVSPNGELHPSRFSEKDLLTVVDREYLFSYIDDPCLATYPLMQKLRAELVEHAL
AAF40224.1_PAL2_Rubus_idaeus LAKRVLTTGVNGELHPSRFCEKDLLMVVEREYLFAYIDDPCSATYPLMQRLRQVLVEHAL
AAC78457.1_PAL_Prunus_avium VAKRTLTTGVNGELHPSRFCEKDLLKVVDREYVFAYIDDPCSATYPLMQKLRQVLVEHAL
sp|P31425.1_PAL1_Solanum_tuberosum VAKRTLTIGAIGELHPARFCEKELLRVVDREYLFTYADDPCSSTYPLMQKLRQVLVDHAM
sp|A2X7F7.1_PAL2_Oryza_sativa VAKKTLSTNSTGDLHVARFCEKDLLKEIDREAVFAYADDPCSHNYPLMKKLRNVLVERAL
sp|P52777.1_PAL_Pinus_taeda VAKKTLSTGLNGELLPGRFCEKDLLQVVDNEHVFSYIDDPCNASYPLTQKLRNILVEHAF
ABU49842.1_PAL_Ginkgo_biloba VAKKTLSTGKNGELLPGRFCEKDLLQAVDSQHVFTYIDDPCSATYPLMQKLRQVLVEHAF
ACS28225.2_PAL_Pinus_massoniana VAKKTLSTGLNGELLPGRFCEKDLLQVVDNEHVFSYIDDPCNASYPLTQKLRNILVEHAF
BAG74771.1_PAL_Ephedra_sinica VARKTLSTGANGELLPGRFCEKELLQVVENEHVLSYIDDPCSPNYVLMQKLRGVLVEHAL
ABC69916.1_PAL_Brassica_napus VAKKVLTTGVNGELHPSRFCEKDLLKVVDREQVYTYADDPCSATYPLIQKLRQVIVDHAL
BAC56977.1_PAL_Daucus_carota VAKRVLTMGVNGELHPSRFCEKDLLRVVDREYIFAYIDDPCSATYPLMQKLRETLVEHAL
ABK24709.1_PAL_Picea_sitchensis VAKKTLSTGLNGELLPGRFCEKDLLQIVDNEHVFSYIDDPCNASYPLTQKLRNVLVEHAF
AHA44840.1_PAL_Larix_kaempferi VAKKTLSTGLNGELLPGRFCEKDLLQIVDNEHVFAYIDDPCNASYPLTQKLRNVLVEHAF
AGC23439.1_PAL_Dendrobium_candidum VAKRVLTIGVKGELHPSRFCEKDLIKVIDREYVLAYADDPCSSTYPLMQNLRQVLVEHAL
ABI33979.1_PAL_Jatropha_curcas VAKRVLTTGANGELHPSRFCGKDLLKVVDREQVFAYIDDPCSATYPLMQKLRQVLVEHAL
XP_002326186.1_PAL_Populus_trichocarpa VSKRVLTTGANGELHPSRFCEKELLKVVDREDVFAYADDPCSATYPLMQKLRQVLVDHAL
XP_002519521.1_PAL_Ricinus_communis VAKRVLTTGANGELHPSRFCEKDLLKVVDREYVFAYADDPCSATYPLMQKLRQILVEHAL
XP_002268732.1_PAL_Vitis_vinifera VAKKTLTTGANGELHPSRFCEKDLLKVVDREHVFAYIDDPCSATYPLMQKVRQVLVEHAL
AFG26322.1_PAL_Cinnamomum_osmophloeum VAKRVLTIGVNGELHPSRFCEKDLIKVVDREYLFAYIDDPCSSTYPLMQKLRQVLVEHAL
BAG70992.1_PAL_Musa_balbisiana VAKRVLTMGANGELHPARFCEKELIKVVDREHVFTYVDDPCSATYPLMQKLRQVLVAHAL
AAP34199.1_PAL_Phalaenopsis_x_Doritaenopsis VAKRTLTMGVNGELHPSRFCEKDLIKVIDREYVFAYADDPCSSTYPLMQKLRAVLVEHAL
ACM61988.1_PAL_Lycoris_radiata VAKRVLTMGAKGELHPSRFCEKDLIKVVDREYVFSYIDDPCSTTYPLMQKLRQILVEHAL
ABN79671.2_PAL_Rudbeckia_hirta VAKKVLTMGVNGELHPSRFCEKDLLRVVDREYVFAYIDDPCLATYPLMQKLRQVLVDHAL
ACJ66297.1_PAL4_Nicotiana_tabacum VAKRTLTMGANGELHPARFCEKELLRVVDREYLFAYADDPCSCNYPLMQKLRQVLVDHAM
ABG75911.1_PAL2_Nicotiana_attenuata VAKRTLTMGANGELHPARFCEKELLRVVDREYLFAYADDPCSCNYPLMQKLRQVLVDHAM
BAL49995.1_PAL_Eucalyptus_robusta VARKVLMVGANGELHPSHYCERDLLKVVDREHVFTYADDACSATYPLMQKLRQVLVDQAL
ACM61988.1_PAL_Lycoris_radiata VAKRVLTMGAKGELHPSRFCEKDLIKVVDREYVFSYIDDPCSTTYPLMQKLRQILVEHAL
BAM28963.1_PAL_Lilium_hybrid_division_I VAKRVLTMGVNGELHPSRFCEKDLIKVIDREYVFAYADDPCSATYPLMQKLRGVLVEHAL
acc

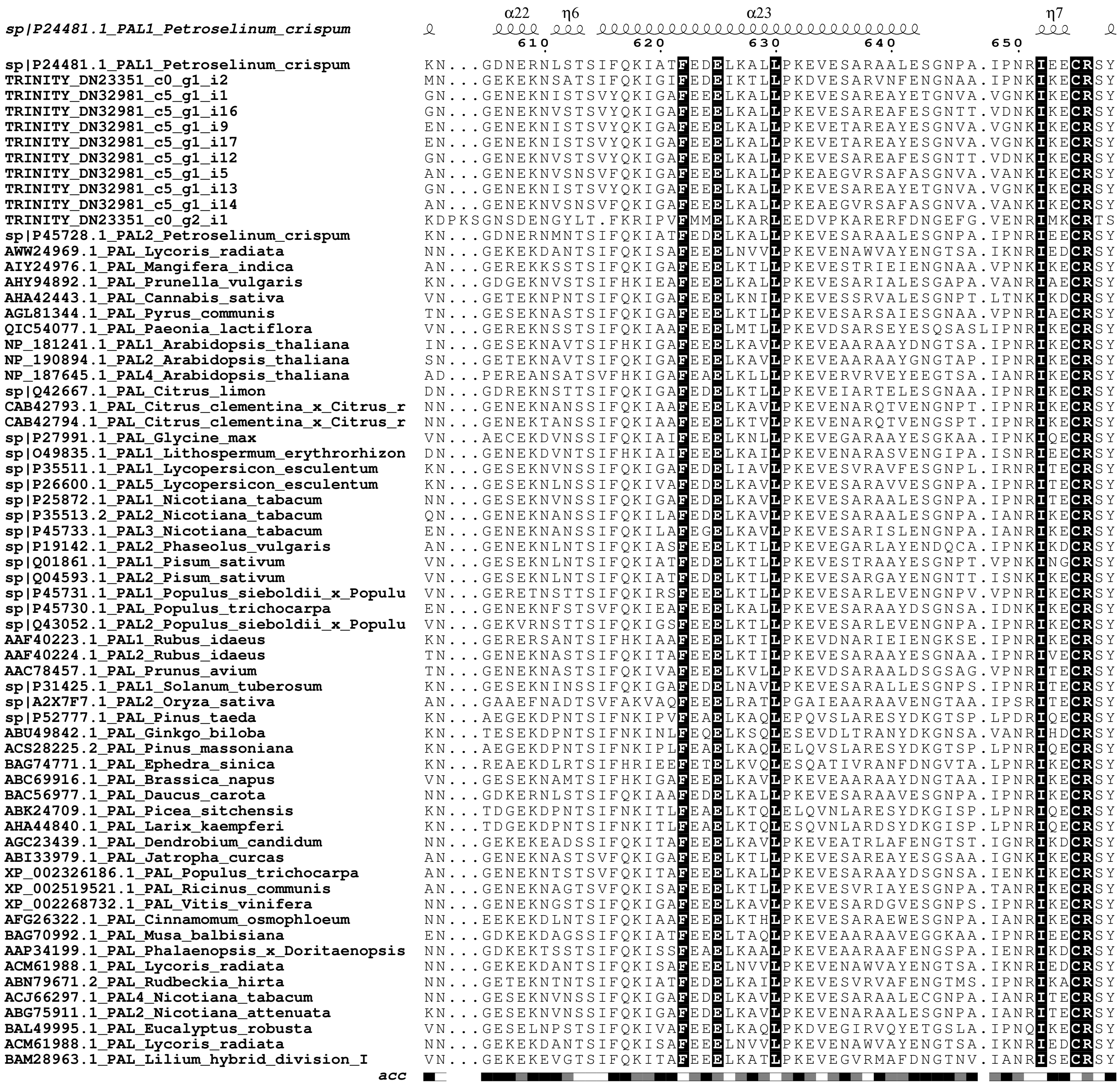

sp|P24481.1_PAL1_Petroselinum_crispum
α22 η6 α23 η7
610 620 630 640 650
sp|P24481.1_PAL1_Petroselinum_crispum KN...GDNERNLSTSIFQKIATFEDELKALLPKEVESARAALESGNPA.IPNRIEECRSY
TRINITY_DN23351_c0_g1_i2 MN...GEKEKNSATSIFQKIGIFEDEIKTLLPKDVESARVNFENGNAA.IPNKIKECRSY
TRINITY_DN32981_c5_g1_i1 GN...GENEKNISTSVYQKIGAFEEELKALLPKEVESAREAYETGNVA.VGNKIKECRSY
TRINITY_DN32981_c5_g1_i16 GN...GENEKNVSTSVYQKIGAFEEELKALLPKEVESAREAFESGNTT.VDNKIKECRSY
TRINITY_DN32981_c5_g1_i9 EN...GENEKNISTSVYQKIGAFEEELKALLPKEVETAREAYESGNVA.VGNKIKECRSY
TRINITY_DN32981_c5_g1_i17 EN...GENEKNISTSVYQKIGAFEEELKALLPKEVETAREAYESGNVA.VGNKIKECRSY
TRINITY_DN32981_c5_g1_i12 GN...GENEKNVSTSVYQKIGAFEEELKALLPKEVESAREAFESGNTT.VDNKIKECRSY
TRINITY_DN32981_c5_g1_i5 AN...GENEKNVSNSVFQKIGAFEEELKALLPKEAEGVRSAFASGNVA.VANKIKECRSY
TRINITY_DN32981_c5_g1_i13 GN...GENEKNISTSVYQKIGAFEEELKALLPKEVESAREAYETGNVA.VGNKIKECRSY
TRINITY_DN32981_c5_g1_i14 AN...GENEKNVSNSVFQKIGAFEEELKALLPKEAEGVRSAFASGNVA.VANKIKECRSY
TRINITY_DN23351_c0_g2_i1 KDPKSGNSDENGYLT.FKRIPVFMMELKARLEEDVPKARERFDNGEFG.VENRIMKCRTS
sp|P45728.1_PAL2_Petroselinum_crispum KN...GDNERNMNTSIFQKIATFEDELKALLPKEVESARAALESGNPA.IPNRIEECRSY
AWW24969.1_PAL_Lycoris_radiata NN...GEKEKDANTSIFQKISAFEEELNVVLPKEVENAWVAYENGTSA.IKNRIEDCRSY
AIY24976.1_PAL_Mangifera_indica AN...GEREKKSSTSIFQKIGAFEEELKTLLPKEVESTRIEIENGNAA.VPNKIKECRSY
AHY94892.1_PAL_Prunella_vulgaris KN...GDGEKNVSTSIFHKIEAFEEELKALLPKEVESARIALESGAPA.VANRIAECRSY
AHA42443.1_PAL_Cannabis_sativa VN...GETEKNPNTSIFQKIGAFEEELKNILPKEVESSRVALESGNPT.LTNKIKDCRSY
AGL81344.1_PAL_Pyrus_communis TN...GESEKNASTSIFQKIGAFEEELKTLLPKEVESARSAIESGNAA.VPNRIAECRSY
QIC54077.1_PAL_Paeonia_lactiflora VN...GEREKNSSTSIFQKIAAFEEELMTLLPKEVDSARSEYESQSASLIPNRIKECRSY
NP_181241.1_PAL1_Arabidopsis_thaliana IN...GESEKNAVTSIFHKIGAFEEELKAVLPKEVEAARAAYDNGTSA.IPNRIKECRSY
NP_190894.1_PAL2_Arabidopsis_thaliana SN...GETEKNAVTSIFQKIGAFEEELKAVLPKEVEAARAAYGNGTAP.IPNRIKECRSY
NP_187645.1_PAL4_Arabidopsis_thaliana AD...PEREANSATSVFHKIGAFEAELKLLLPKEVERVRVEYEEGTSA.IANRIKECRSY
sp|Q42667.1_PAL_Citrus_limon DN...GDREKNSTTSIFQKIGAFEDELKTLLPKEVEIARTELESGNAA.IPNRIKECRSY
CAB42793.1_PAL_Citrus_clementina_x_Citrus_r NN...GENEKNANSSIFQKIAAFEEELKAVLPKEVENARQTVENGNPT.IPNRIKECRSY
CAB42794.1_PAL_Citrus_clementina_x_Citrus_r NN...GENEKTANSSIFQKIAAFEEELKTVLPKEVENARQTVENGSPT.IPNRIKECRSY
sp|P27991.1_PAL_Glycine_max VN...AECEKDVNSSIFQKIAIFEEELKNLLPKEVEGARAAYESGKAA.IPNKIQECRSY
sp|O49835.1_PAL1_Lithospermum_erythrorhizon DN...GENEKDVNTSIFHKIAIFEEELKAILPKEVENARASVENGIPA.ISNRIEECRSY
sp|P35511.1_PAL1_Lycopersicon_esculentum KN...GESEKNVNSSIFQKIGAFEDELIAVLPKEVESVRAVFESGNPL.IRNRITECRSY
sp|P26600.1_PAL5_Lycopersicon_esculentum KN...GESEKNLNSSIFQKIVAFEDELKAVLPKEVESARAVVESGNPA.IPNRITECRSY
sp|P25872.1_PAL1_Nicotiana_tabacum NN...GESEKNVNSSIFQKIGAFEDELKAVLPKEVESARAALESGNPA.IPNRITECRSY
sp|P35513.2_PAL2_Nicotiana_tabacum QN...GENEKNANSSIFQKILAFEDELKAVLPKEVESARAALESGNPA.IANRIKECRSY
sp|P45733.1_PAL3_Nicotiana_tabacum EN...GENEKNANSSIFQKILAFEGELKAVLPKEVESARISLENGNPA.IANRIKECRSY
sp|P19142.1_PAL2_Phaseolus_vulgaris AN...GENEKNLNTSIFQKIASFEEELKTLLPKEVEGARLAYENDQCA.IPNKIKDCRSY
sp|Q01861.1_PAL1_Pisum_sativum VN...GESEKNLNTSIFQKIATFEDELKTLLPKEVESTRAAYESGNPT.VPNKINGCRSY
sp|Q04593.1_PAL2_Pisum_sativum VN...GESEKNLNTSIFQKIATFEDELKTLLPKEVESARGAYENGNTT.ISNKIKECRSY
sp|P45731.1_PAL1_Populus_sieboldii_x_Populu VN...GERETNSTTSIFQKIRSFEEELKTLLPKEVESARLEVENGNPV.VPNRIKECRSY
sp|P45730.1_PAL_Populus_trichocarpa EN...GENEKNFSTSVFQKIEAFEEELKALLPKEVESARAAYDSGNSA.IDNKIKECRSY
sp|Q43052.1_PAL2_Populus_sieboldii_x_Populu VN...GEKVRNSTTSIFQKIGSFEEELKTLLPKEVESARLEVENGNPA.IPNRIKECRSY
AAF40223.1_PAL1_Rubus_idaeus KN...GERERSANTSIFHKIAAFEEELKTILPKEVDNARIEIENGKSE.IPNRIKECRSY
AAF40224.1_PAL2_Rubus_idaeus TN...GENEKNASTSIFQKITAFEEELKTILPKEVESARAAYESGNAA.IPNRIVECRSY
AAC78457.1_PAL_Prunus_avium TN...GENEKNASTSIFQKIVAFEEELKVLLPKEVDSARAALDSGSAG.VPNRITECRSY
sp|P31425.1_PAL1_Solanum_tuberosum KN...GESEKNINSSIFQKIGAFEDELNAVLPKEVESARALLESGNPS.IPNRITECRSY
sp|A2X7F7.1_PAL2_Oryza_sativa AN...GAAEFNADTSVFAKVAQFEEELRATLPGAIEAARAAVENGTAA.IPSRITECRSY
sp|P52777.1_PAL_Pinus_taeda KN...AEGEKDPNTSIFNKIPVFEAELKAQLEPQVSLARESYDKGTSP.LPDRIQECRSY
ABU49842.1_PAL_Ginkgo_biloba KN...TESEKDPNTSIFNKINLFEQELKSQLESEVDLTRANYDKGNSA.VANRIHDCRSY
ACS28225.2_PAL_Pinus_massoniana KN...AEGEKDPNTSIFNKIPLFEAELKAQLELQVSLARESYDKGTSP.LPNRIQECRSY
BAG74771.1_PAL_Ephedra_sinica KN...REAEKDLRTSIFHRIEEFETELKVQLESQATIVRANFDNGVTA.LPNRIKECRSY
ABC69916.1_PAL_Brassica_napus VN...GESEKNAMTSIFHKIGAFEEELKAVLPKEVEAARAAYDNGTAA.IPNRIKECRSY
BAC56977.1_PAL_Daucus_carota NN...GDKERNLSTSIFQKIAAFEDELKALLPKEVESARAAVESGNPA.IPNRIKECRSY
ABK24709.1_PAL_Picea_sitchensis KN...TDGEKDPNTSIFNKITLFEAELKTQLELQVNLARESYDKGISP.LPNRIQECRSY
AHA44840.1_PAL_Larix_kaempferi KN...TDGEKDPNTSIFNKITLFEAELKTQLESQVNLARDSYDKGISP.LPNRIQECRSY
AGC23439.1_PAL_Dendrobium_candidum NN...GEKEKEADSSIFQKITAFEEELKAVLPKEVEATRLAFENGTST.IGNRIKDCRSY
ABI33979.1_PAL_Jatropha_curcas AN...GENEKNASTSVFQKIGAFEEELKTLLPKEVESAREAYESGSAA.IGNKIKECRSY
XP_002326186.1_PAL_Populus_trichocarpa AN...GENEKNTSTSVFQKITAFEEELKALLPKEVESARAAYDSGNSA.IENKIKECRSY
XP_002519521.1_PAL_Ricinus_communis AN...GENEKNAGTSVFQKISAFEEELKTLLPKEVESVRIAYESGNPA.TANRIKECRSY
XP_002268732.1_PAL_Vitis_vinifera NN...GENEKNGSTSIFQKIGAFEEELKAVLPKEVESARDGVESGNPS.IPNRIKECRSY
AFG26322.1_PAL_Cinnamomum_osmophloeum NN...EEKEKDLNTSIFQKIAAFEEELKTHLPKEVESARAEWESGNPA.IANRIKECRSY
BAG70992.1_PAL_Musa_balbisiana EN...GDKEKDAGSSIFQKIATFEEELTAQLPKEVEAARAAVEGGKAA.IPNRIEECRSY
AAP34199.1_PAL_Phalaenopsis_x_Doritaenopsis NN...GDKEKTSSTSIFQKISSFEAELKAALPKEVEAARAAFENGSPA.IENRIKDCRSY
ACM61988.1_PAL_Lycoris_radiata NN...GEKEKDANTSIFQKISAFEEELNVVLPKEVENAWVAYENGTSA.IKNRIEDCRSY
ABN79671.2_PAL_Rudbeckia_hirta NN...GETEKNTNTSIFQKIATFEDELKAILPKEVESVRVAFENGTMS.IPNRIKACRSY
ACJ66297.1_PAL4_Nicotiana_tabacum NN...GESEKNVNSSIFQKIGAFEDELKAVLPKEVESARAALECGNPA.IANRITECRSY
ABG75911.1_PAL2_Nicotiana_attenuata KN...GESEKNVNSSIFQKIGAFEDELKAVLPKEVENARAALESGNPA.IANRITECRSY
BAL49995.1_PAL_Eucalyptus_robusta VN...GESELNPSTSIFQKIVAFEEELKAQLPKDVEGIRVQYETGSLA.IPNQIKECRSY
ACM61988.1_PAL_Lycoris_radiata NN...GEKEKDANTSIFQKISAFEEELNVVLPKEVENAWVAYENGTSA.IKNRIEDCRSY
BAM28963.1_PAL_Lilium_hybrid_division_I VN...GEKEKEVGTSIFQKITAFEEELKATLPKEVEGVRMAFDNGTNV.IANRISECRSY
acc

```
                                              α24                      α25              α26
sp|P24481.1_PAL1_Petroselinum_crispum                            TT
                                           660       670       680       690       700       710
sp|P24481.1_PAL1_Petroselinum_crispum      PLYKFVRKELGTEYLTGEKVTSPGEEFEKVFIAMSKGEIIDPLLECLESWNGA.......
TRINITY_DN23351_c0_g1_i2                   PLYKFVREDLSTSLLTGENLRSPGEDFDKVFEAICAGKLIDPLFECLKEWNGA.......
TRINITY_DN32981_c5_g1_i1                   PLYKFVREELGTRLLTGEKGRSPGQDFDKVFTAMCEGKIIDPMMECLKEWNGT.......
TRINITY_DN32981_c5_g1_i16                  PLYKFVRQELSTGLLTGEKGRSPGEDFDKVFTAMCQGKIIDPMMDCLKEWNGA.......
TRINITY_DN32981_c5_g1_i9                   PLYKFVREELGTGLLTGEKGRSPGQDFDKVFTAMCEGKIIDPMMECLKEWNGS.......
TRINITY_DN32981_c5_g1_i17                  PLYKFVREELGTGLLTGEKGRSPGQDFDKVFTAMCEGKIIDPMMECLKEWNGS.......
TRINITY_DN32981_c5_g1_i12                  PLYKFVRQELSTGLLTGEKGRSPGEDFDKVFTAMCQGKIIDPMMDCLKEWNGA.......
TRINITY_DN32981_c5_g1_i5                   PLYKFLREELGTGLLTGEKGRSPGEDFDKVFTAMCQGKLIDPMMDCLKEWNGA.......
TRINITY_DN32981_c5_g1_i13                  PLYKFVREELGTRLLTGEKGRSPGQDFDKVFTAMCEGKIIDPMMECLKEWNGT.......
TRINITY_DN32981_c5_g1_i14                  PLYKFLREELGTGLLTGEKGR.......................................
TRINITY_DN23351_c0_g2_i1                   PIYEFVRSEVGTSLLSGAKKVSPGEDIEKVHEAINEGKLGQVLIRCLKDWKQA.......
sp|P45728.1_PAL2_Petroselinum_crispum      PLYKFVRKELGIEYLTGEKVTSPGEEFDKVFIAMSKGEIIDPLLECLESWNGA.......
AWW24969.1_PAL_Lycoris_radiata             PLYRFVREEIGTSLLTGEKVRSPGEEFDKVFNAICKGKLVDPLLECLEDWNGA.......
AIY24976.1_PAL_Mangifera_indica            PLYKFVREELGASFLTGEKVKSPGEEFDKVFSAICAGKLIDPMMECLKEWNGA.......
AHY94892.1_PAL_Prunella_vulgaris           PLYKFIREGLGTGFLTGEKAVSPGEECDKVFTALCDGLIVDPLLECLQGWNGE.......
AHA42443.1_PAL_Cannabis_sativa             PLYKFVRDNLETNLLSGENVRSPGEEFDKVFTAMCQGKIIDPLLECLSNWNGT.......
AGL81344.1_PAL_Pyrus_communis              PLYKFVREELGGEYLTGEKVRSPGEECDRVFQAICQGKIIDPILGCLEGWNGA.......
QIC54077.1_PAL_Paeonia_lactiflora          PLYKFVREELGSGLLTGDKTRSPGEDFDKVFSAMCEGKLIDPMLDCLKEWNGA.......
NP_181241.1_PAL1_Arabidopsis_thaliana      PLYRFVREELGTELLTGEKVTSPGEEFDKVFTAICEGKIIDPMMECLNEWNGA.......
NP_190894.1_PAL2_Arabidopsis_thaliana      PLYRFVREELGTKLLTGEKVVSPGEEFDKVFTAMCEGKLIDPLMDCLKEWNGA.......
NP_187645.1_PAL4_Arabidopsis_thaliana      PLYRFVRDELNTELLTGENVRSPGEEFDKVFLAISDGKLIDPLLECLKEWNGA.......
sp|Q42667.1_PAL_Citrus_limon               PLYKIVREDIGTSLLTGEKVRSPGEEFDKVFTAMCEGKLIDPMLECLKEWNGA.......
CAB42793.1_PAL_Citrus_clementina_x_Citrus_r PLYRLVREELGTNFLTGEKVTSPGEKFDKVFTAMCQGKIIDPMLECLREWNGA.......
CAB42794.1_PAL_Citrus_clementina_x_Citrus_r PLYRLVREGLGSNFLTGEKVTSPGEEFDKVFTAMCQGKIIDPMLECLREWNGA.......
sp|P27991.1_PAL_Glycine_max                PLYKFVREELGTGLLTGEKVRSPGEEFDKLFTAMCQGKIIDPLMECLGEWNGA.......
sp|O49835.1_PAL1_Lithospermum_erythrorhizon PLYKFVREELGTELLTGEKVRSPGEELDKVFTAMCEGKLVDPLLACLEAWNGA.......
sp|P35511.1_PAL1_Lycopersicon_esculentum   PLYRLVREELGTELLTGEKVRSPGEEIDKVFTAICNGQIIDPLLECLKSWNGA.......
sp|P26600.1_PAL5_Lycopersicon_esculentum   PLYRLVRQEVGTELLTGEKVRSPGEEIDKVFTAFCNGQIIDPLLECLKSWNGA.......
sp|P25872.1_PAL1_Nicotiana_tabacum         PLYRFVRKELGTELLTGEKVRSPGEECDKVFTAMCNGQIIDPMLECLKSWNGA.......
sp|P35513.2_PAL2_Nicotiana_tabacum         PLYRFVRGELGAELLTGEKVRSPGEECDKVFTAMCNGQIIDSLLECLKEWNGA.......
sp|P45733.1_PAL3_Nicotiana_tabacum         PLYRFVREELGAELLTGEKVRSPGEECDKVFTAMCNGQIIDSLLECLKEWNGA.......
sp|P19142.1_PAL2_Phaseolus_vulgaris        PLYKFVREELGTSLLTGEKVISPGEECDKVFSAMCQGKIIDPLLECLGEWNGA.......
sp|Q01861.1_PAL1_Pisum_sativum             PLYRFVRQELGTGLLTGEKVISPGEECDKLFTAICQGKIIDPLLQCLGDWNGA.......
sp|Q04593.1_PAL2_Pisum_sativum             PLYKFVREELGTSLLTGEKVISPGEECDKLFTAICQGKIIDPLLECLGDWNGA.......
sp|P45731.1_PAL1_Populus_sieboldii_x_Populu PLYKFVREELGTSLLTGEKVKSPGEDFDKVFTAICAGKLMDPLLECLKEWNGA.......
sp|P45730.1_PAL_Populus_trichocarpa        PLYKFVREELGTVLLTGEKVQSPGEEFDKVFTAMCQGKIIDPMLECLGEWNGS.......
sp|Q43052.1_PAL2_Populus_sieboldii_x_Populu PLYKFVREELGTSLLTGEKVKSPGEEFDKVFTAICAGKLIDPLLECLKEWDGA.......
AAF40223.1_PAL1_Rubus_idaeus               PLYRFVREELGTSLLTGEKIKSPGEECYKVFNAICAGKLVDPLLECLKEWNGA.......
AAF40224.1_PAL2_Rubus_idaeus               PLYKFVREELGGEFLTGEKVRSPGEECDKVFTAMCQGNIIDPILDCLSGWNGE.......
AAC78457.1_PAL_Prunus_avium                PLYKFVREELGAEYLTGEKVRSPGEECDKVFTAICEGKIIDPILDCLEGWNGA.......
sp|P31425.1_PAL1_Solanum_tuberosum         PLYRLVRQELGTELLTGEKVRSPGEEIEKVFTAMCNGQINDPLLECLKSWNGA.......
sp|A2X7F7.1_PAL2_Oryza_sativa              PLYRFVREELGTKYLTGEKTRSPGEELNKVLVAINEGKHIDPLLECLKEWNGE.......
sp|P52777.1_PAL_Pinus_taeda                PLYEFVRNQLGTKLLSGTRTISPGEVIEVVYDAISEDKVIVPLFKCLDGWKGTLAHSEIN
ABU49842.1_PAL_Ginkgo_biloba               PLYEFVRTQLGTKLLSGTRATSPGEYIEKVFDAICEDKIIEPIIKCLDGWKGT.......
ACS28225.2_PAL_Pinus_massoniana            PLYEFVRNQLGTKLLSGTRTTSPGEVIEVVYDAISEDKVIVPLFKCLDGWKGT.......
BAG74771.1_PAL_Ephedra_sinica              PLYSFVRETLGTQLLSGDRDISPGEDIEVVYKAVKANDIIVPLFKCLHGWKGT.......
ABC69916.1_PAL_Brassica_napus              PLYRFVREELGTELLTGEKATSPGEEFDKVFTAICEGKIIDPLMECLDEWNGA.......
BAC56977.1_PAL_Daucus_carota               PLYKFVREELGTEYLTGEKVTSPGEEFDKVFTAMTKGEIIDPLLECLQSWNGA.......
ABK24709.1_PAL_Picea_sitchensis            PLYEFVRTQLGTKLLSGTRTTSPGEVIELVYDAISEDKIIGPLLKCVEGWKAT.......
AHA44840.1_PAL_Larix_kaempferi             PLYEFVRIQLGTKLLSGARTTSPGEVIELVYDAISEDKIIGPLFKCLDGWKAT.......
AGC23439.1_PAL_Dendrobium_candidum         PIYRFVREELGTSFLTGEEVRSPGEEFDKVFNAICEGKAIDPLLECLQEWDGA.......
ABI33979.1_PAL_Jatropha_curcas             PLYKFVREELGSGLLTGEKVRSPGEEFDKVFTAMCEGKIIDPMMECLKEWNGA.......
XP_002326186.1_PAL_Populus_trichocarpa     PLYKFVREELGTGLLTGEKVRSPGEEFDKVFTAMCQGKIIDPMLECLGEWNGA.......
XP_002519521.1_PAL_Ricinus_communis        PLYKFVREELGTGLLTGDKVMSPGEEFDKVFTAMCQGKIIDPMMDCLKEWNGA.......
XP_002268732.1_PAL_Vitis_vinifera          PLYKFVREELGTGLLTGETVRSPGEDFDKVFTAMCEGKIIDPLLDCLSAWNGA.......
AFG26322.1_PAL_Cinnamomum_osmophloeum      PLYKFVREELKTSLLTGERVRSPGEEFDKVFAAICEGKVIDPFLECLREWNGA.......
BAG70992.1_PAL_Musa_balbisiana             PLYRLVREELKTGFLTGEKVRSPGEEFDKVFDAICQGKVIDPLLECLKEWNGA.......
AAP34199.1_PAL_Phalaenopsis_x_Doritaenopsis PLYRFVK.QVGAGFLTGEKIVSPGEELDKVFNAICEGKAIDPMLDCLKEWNGA.......
ACM61988.1_PAL_Lycoris_radiata             PLYRFVREEIGTSLLTGEKVRSPGEEFDKVFNAICKGKLVDPLLECLEDWNGA.......
ABN79671.2_PAL_Rudbeckia_hirta             PLYRFVREELSGAYLTGEKVTSPGEEFDKVFTAMSKGQIIDPLLECVEGWNGA.......
ACJ66297.1_PAL4_Nicotiana_tabacum          PLYRFVRKELGTELLTGERVRSPGEECEKVFTAMCNGQIIDPMLECLKSWNGA.......
ABG75911.1_PAL2_Nicotiana_attenuata        PLYRFVRKELGTELLTGERVRSPGEECDKVFTAMCNGQIIDPMLECLKSWNGA.......
BAL49995.1_PAL_Eucalyptus_robusta          PLYKLVREELGTALLTGEGVISPGEDFDKVFTAICAGKLIDPLLECLSGWNGA.......
ACM61988.1_PAL_Lycoris_radiata             PLYRFVREEIGTSLLTGEKVRSPGEEFDKVFNAICKGKLVDPLLECLEDWNGA.......
BAM28963.1_PAL_Lilium_hybrid_division_I    PLYKFVREELGAGYLTGEKVRSPGEEFNKVFIAMSQWKVIDPLLECLSDWNGA.......
                                       acc
```

sp|P24481.1_PAL1_Petroselinum_crispum

**sp|P24481.1_PAL1_Petroselinum_crispum**	...PLPI...C......................
**TRINITY_DN23351_c0_g1_i2**	...PLPI...C......................
**TRINITY_DN32981_c5_g1_i1**	...PIPI...C......................
**TRINITY_DN32981_c5_g1_i16**	...PLPL...S......................
**TRINITY_DN32981_c5_g1_i9**	...PLPI...C......................
**TRINITY_DN32981_c5_g1_i17**	...PLPI...C......................
**TRINITY_DN32981_c5_g1_i12**	...PLPL...S......................
**TRINITY_DN32981_c5_g1_i5**	...PVPI...C......................
**TRINITY_DN32981_c5_g1_i13**	...PIPI...C......................
**TRINITY_DN32981_c5_g1_i14**	....
**TRINITY_DN23351_c0_g2_i1**	....
**sp|P45728.1_PAL2_Petroselinum_crispum**	...PLPI...C......................
**AWW24969.1_PAL_Lycoris_radiata**	...PLPI...C......................
**AIY24976.1_PAL_Mangifera_indica**	...PLPL...C......................
**AHY94892.1_PAL_Prunella_vulgaris**	...PLPI...C......................
**AHA42443.1_PAL_Cannabis_sativa**	...PLPL...C......................
**AGL81344.1_PAL_Pyrus_communis**	...PLPI...C......................
**QIC54077.1_PAL_Paeonia_lactiflora**	...PLPI...C......................
**NP_181241.1_PAL1_Arabidopsis_thaliana**	...PIPI...C......................
**NP_190894.1_PAL2_Arabidopsis_thaliana**	...PIPI...C......................
**NP_187645.1_PAL4_Arabidopsis_thaliana**	...PVSI...C......................
**sp|Q42667.1_PAL_Citrus_limon**	...PLPI...CQN....................
**CAB42793.1_PAL_Citrus_clementina_x_Citrus_r**	...PLPI...C......................
**CAB42794.1_PAL_Citrus_clementina_x_Citrus_r**	...PLPI...C......................
**sp|P27991.1_PAL_Glycine_max**	...PLPI...S......................
**sp|O49835.1_PAL1_Lithospermum_erythrorhizon**	...PLPI...C......................
**sp|P35511.1_PAL1_Lycopersicon_esculentum**	...PLPI...C......................
**sp|P26600.1_PAL5_Lycopersicon_esculentum**	...PIPI...C......................
**sp|P25872.1_PAL1_Nicotiana_tabacum**	...PLPI...C......................
**sp|P35513.2_PAL2_Nicotiana_tabacum**	...PLPI...C......................
**sp|P45733.1_PAL3_Nicotiana_tabacum**	...PLPI...C......................
**sp|P19142.1_PAL2_Phaseolus_vulgaris**	...PLPI...C......................
**sp|Q01861.1_PAL1_Pisum_sativum**	...PLPI...S......................
**sp|Q04593.1_PAL2_Pisum_sativum**	...PLPI...S......................
**sp|P45731.1_PAL1_Populus_sieboldii_x_Populu**	...PLPI...C......................
**sp|P45730.1_PAL_Populus_trichocarpa**	...PLPI...C......................
**sp|Q43052.1_PAL2_Populus_sieboldii_x_Populu**	...PLPI...C......................
**AAF40223.1_PAL1_Rubus_idaeus**	...PLPI...S......................
**AAF40224.1_PAL2_Rubus_idaeus**	...PLPI...C......................
**AAC78457.1_PAL_Prunus_avium**	...PLPI...C......................
**sp|P31425.1_PAL1_Solanum_tuberosum**	...PLPI...C......................
**sp|A2X7F7.1_PAL2_Oryza_sativa**	...PLPI...C......................
**sp|P52777.1_PAL_Pinus_taeda**	NLPRSPLYNDCYDLSPRMLLLMLLFSDPEFDWS
**ABU49842.1_PAL_Ginkgo_biloba**	...PGPF..........................
**ACS28225.2_PAL_Pinus_massoniana**	...PGPF..........................
**BAG74771.1_PAL_Ephedra_sinica**	...PGPF..........................
**ABC69916.1_PAL_Brassica_napus**	...PIPI...C......................
**BAC56977.1_PAL_Daucus_carota**	...PLPI...C......................
**ABK24709.1_PAL_Picea_sitchensis**	...PGPF..........................
**AHA44840.1_PAL_Larix_kaempferi**	...PGPF..........................
**AGC23439.1_PAL_Dendrobium_candidum**	...PLPI...C......................
**ABI33979.1_PAL_Jatropha_curcas**	...PLPI...C......................
**XP_002326186.1_PAL_Populus_trichocarpa**	...PLPI...C......................
**XP_002519521.1_PAL_Ricinus_communis**	...PLPI...C......................
**XP_002268732.1_PAL_Vitis_vinifera**	...PLPI...C......................
**AFG26322.1_PAL_Cinnamomum_osmophloeum**	...PLPI...C......................
**BAG70992.1_PAL_Musa_balbisiana**	...PLPI...C......................
**AAP34199.1_PAL_Phalaenopsis_x_Doritaenopsis**	...PLPI...C......................
**ACM61988.1_PAL_Lycoris_radiata**	...PLPI...C......................
**ABN79671.2_PAL_Rudbeckia_hirta**	...PLPI...C......................
**ACJ66297.1_PAL4_Nicotiana_tabacum**	...PLPI...C......................
**ABG75911.1_PAL2_Nicotiana_attenuata**	...PLPI...C......................
**BAL49995.1_PAL_Eucalyptus_robusta**	...PLPI...S......................
**ACM61988.1_PAL_Lycoris_radiata**	...PLPI...C......................
**BAM28963.1_PAL_Lilium_hybrid_division_I**	...PLPI...C......................
acc	

AAK54447.1_C4H_Sorghum_bicolor

```
                                                                  1                                 10
AAK54447.1_C4H_Sorghum_bicolor                ....................MDL.........................VLLEKALLGLFA
TRINITY_DN32464_c6_g3_i2                      ....................MDL.........................ILLEKTLLGLFA
TRINITY_DN15593_c0_g1_i1                      ...................................MP...........LSIAKSI..IFG
NP_180607.1_C4H_Arabidopsis_thaliana          ....................MDL.........................LLLEKSLIAVFV
ACA25599.1_C4H_Parthenocissus_henryana        ....................MDL.........................ILIEKALLAVFC
CAK95273.1_C4H_Cucumis_sativus                ....................MDL.........................LLLEKTLLGLFL
QIC52990.1_C4H_Solanum_nigrum                 ....................MDL.........................LLLEKTLIGLFF
AHY94893.1_C4H_Prunella_vulgaris              ....................MDL.........................LLVEKTLIGLFF
AWW24970.1_C4H_Lycoris_radiata                ....................MDL.........................ILLEKSLLAVFF
ASA39653.1_C4H_Marchantia_emarginata          ...................MSEL........LEKVL...PGSSAA...FTVQNFLYALLA
ASA39650.1_C4H_Conocephalum_japonicum         ...................MTEL........MEKVL...PGSKAN...FTFQNVLLALLA
ASA39648.1_C4H_Marchantia_paleacea            ...................MVDQ........LERLL...PGSSAA...LTMQNVLYGLLA
ASA39646.1_C4H_Plagiochasma_appendiculatum    MGTGFSTMAVCLIAAGMSKVTAE........AEEVVAAPSGSNSA...WTLQNVLYGLLA
AFX98061.1_C4H_Cunninghamia_lanceolata        ...................MADV........QQGSL............AVVEKSLVALFV
AFZ78542.1_C4H_Populus_tomentosa              ....................MDL.........................LLLEKTLLGSFV
AUG71936.1_C4H_Narcissus_pseudonarcissus      ....................MDL.........................ILLEKSLLAVFF
AEA72281.1_C4H_Angelica_gigas                 ....................MDF.........................LLLEKALLGLFI
ADO24190.1_C4H_Allium_sativum                 ....................MEL.........................LLLEKTLLSLFF
ADN32769.1_C4H_Scutellaria_baicalensis        ....................MDL.........................LLLEKSLLGLFL
AIS92509.1_C4H_Epimedium_sagittatum           ....................MDL.........................LLLEKSLIASFI
AGP25594.1_C4H_Aquilaria_sinensis             ....................MDL.........................LLLEKTLLALFA
AAG50231.1_C4H_Populus_trichocarpa_x_Populus  ....................MDL.........................LLLEKTLLGSFV
sp|Q43054.1_C4H_Populus_sieboldii_x_Populus_  ....................MDL.........................LLLEKTLLGSFV
AAG10197.1_C4H_Gossypium_arboreum             ....................MDL.........................LFLEKVLISLFF
AAG10196.1_C4H_Gossypium_arboreum             ....................MDL.........................LFLEKALLGLFV
sp|Q43033.1_C4H_Petroselinum_crispum          ...................MMDF.........................VLLEKALLGLFI
CAC35977.1_C4H_Ruta_graveolens                ....................MDL.........................LLLEKALLGLFA
AAF66066.2_C4H_Citrus_sinensis                ....................MDLNGWCNSGNQNMCCCQ.SYVKRGYDRVLSFNGL.....
AAK57011.1_C4H_Citrus_x_paradisi              ....................MDL.........................LLLEKTLLALFA
sp|P48522.1_C4H_Catharanthus_roseus           ....................MDL.........................LLLEKTLLGLFA
BAB71717.1_C4H_Lithospermum_erythrorhizon     ....................MDL.........................LLLEKVLIGLFI
BAB71716.1_C4H_Lithospermum_erythrorhizon     ....................MDL.........................LLLEKALIGLFF
AAC35857.1_C4H_Capsicum_chinense              ............................................................
AAG43824.1_C4H_Capsicum_annuum                ....................MDL.........................LLLEKTLVGLFF
sp|Q43240.1_C4H_Zinnia_elegans                ....................MDL.........................LLVEKTLLALFA
sp|Q04468.1_C4H_Helianthus_tuberosus          ....................MDL.........................LLIEKTLVALFA
sp|Q42797.1_C4H_Glycine_max                   ....................MDL.........................LLLEKTLIGLFL
sp|P37115.1_C4H_Vigna_radiata                 ....................MDL.........................LLLEKTLLGLFL
sp|Q96423.1_C4H_Glycyrrhiza_echinata          ....................MDL.........................LLLEKTLLGLFI
sp|O81928.2_C4H_Cicer_arietinum               ....................MDL.........................LLLEKTLLALFI
sp|P37114.1_C4H_Medicago_sativa               ....................MDL.........................LLLEKTLLALFI
sp|Q43067.2_C4H_Pisum_sativum                 ....................MDL.........................LLLEKTLLALFL
AAD11427.1_C4H_Mesembryanthemum_crystallinum  .......MA..........KMET........HSKPMS...........KKLARNL..ILL
sp|O24315_C4H_Phaseolus_vulgaris              ............................................................
AAK62344.1_C4H_Nicotiana_tabacum              ................................MKNMA...........KLLNKTIFCILF
AAK62345.1_C4H_Nicotiana_tabacum              ...................................MA...........KLLNNTIFCILF
AAG17469.1_C4H_Triticum_aestivum              ....................MDV.........................LLLEKALLGLFA
AAD23378.1_C4H_Pinus_taeda                    ....................MEI..........MTV............ASLEKGLLAIFA
                                         acc
```

AAK54447.1_C4H_Sorghum_bicolor

```
                                                  20                                                30
AAK54447.1_C4H_Sorghum_bicolor                AAVLAVAVAK.....LT...................................GKRYRLPP
TRINITY_DN32464_c6_g3_i2                      AIIVAIVVSK.....LR...................................GKRFKLPP
TRINITY_DN15593_c0_g1_i1                      SLIIAIFTKF.....L..YLNI.FN..NVSFPHVWKIVLFLLPFAYLFTSICKSSKNLPP
NP_180607.1_C4H_Arabidopsis_thaliana          AVILATVISK.....LR...................................GKKLKLPP
ACA25599.1_C4H_Parthenocissus_henryana        AIILAITISK.....LL...................................GKKFKLPP
CAK95273.1_C4H_Cucumis_sativus                SVVLAIAISK.....LR...................................GKRFKPPP
QIC52990.1_C4H_Solanum_nigrum                 AILIAIIVSK.....LR...................................SKRFKLPP
AHY94893.1_C4H_Prunella_vulgaris              AIVVAAVVSE.....LR...................................GKKFKLPP
AWW24970.1_C4H_Lycoris_radiata                AIILSILISK.....LR...................................GKKLRLPP
ASA39653.1_C4H_Marchantia_emarginata          ATLLVILIIE.....LR...................................KEKLNLPP
ASA39650.1_C4H_Conocephalum_japonicum         ATLLVIVIVE.....LT...................................RKRLKLPP
ASA39648.1_C4H_Marchantia_paleacea            ATLLVILIIE.....LR...................................KERLKLPP
ASA39646.1_C4H_Plagiochasma_appendiculatum    ATLGVILLMK.....LK...................................EKKLKLPP
AFX98061.1_C4H_Cunninghamia_lanceolata        VVVGAVLVNK.....FR...................................GKKLKLPP
AFZ78542.1_C4H_Populus_tomentosa              AVLVAILVSK.....LR...................................GKRFKLPP
AUG71936.1_C4H_Narcissus_pseudonarcissus      AVIFSIIVSK.....LR...................................GKKLKLPP
AEA72281.1_C4H_Angelica_gigas                 ATIVAITVSK.....LR...................................GKKLKLPP
ADO24190.1_C4H_Allium_sativum                 AITLAIVISK.....LR...................................GRKFKLPP
ADN32769.1_C4H_Scutellaria_baicalensis        AIIVATMVSK.....LR...................................GKKFKLPP
AIS92509.1_C4H_Epimedium_sagittatum           AIIVAIAISK.....LR...................................GKRFKLPP
AGP25594.1_C4H_Aquilaria_sinensis             TVILAIAISK.....LC...................................GKKYRLPP
AAG50231.1_C4H_Populus_trichocarpa_x_Populus  AILVAILVSK.....LR...................................GKRFKLPP
sp|Q43054.1_C4H_Populus_sieboldii_x_Populus_  AVLVAILVSK.....LR...................................GKRFKLPP
AAG10197.1_C4H_Gossypium_arboreum             TIIFAILVSK.....LR...................................GKRFKLPP
AAG10196.1_C4H_Gossypium_arboreum             AVVLAITISK.....LR...................................GKRFKLPP
sp|Q43033.1_C4H_Petroselinum_crispum          ATIVAITISK.....LR...................................GKKLKLPP
CAC35977.1_C4H_Ruta_graveolens                AAVVAIAVSK.....LR...................................GKRFKLPP
AAF66066.2_C4H_Citrus_sinensis                .....ITVSK.....LR...................................GKRFKLPP
AAK57011.1_C4H_Citrus_x_paradisi              AVVVAITVSK.....LR...................................GKRFKLPP
sp|P48522.1_C4H_Catharanthus_roseus           AIIVASIVSK.....LR...................................GKKFKLPP
BAB71717.1_C4H_Lithospermum_erythrorhizon     AIILSIIISK.....LG...................................GKKFKLPP
BAB71716.1_C4H_Lithospermum_erythrorhizon     SFIIAIVISK.....LR...................................GKKFKLPP
AAC35857.1_C4H_Capsicum_chinense              ............................................................
AAG43824.1_C4H_Capsicum_annuum                AIVVAIIVSK.....LR...................................SKRFKLPP
sp|Q43240.1_C4H_Zinnia_elegans                AIIASIFISK.....LR...................................GKRFKLPP
sp|Q04468.1_C4H_Helianthus_tuberosus          AIIGAILISK.....LR...................................GKKFKLPP
sp|Q42797.1_C4H_Glycine_max                   AAVVAIAVST.....LR...................................GRKFKLPP
sp|P37115.1_C4H_Vigna_radiata                 AAVVAIVVSK.....LR...................................GKRFKLPP
sp|Q96423.1_C4H_Glycyrrhiza_echinata          AAITAIAISK.....LR...................................GRRFKLPP
sp|O81928.2_C4H_Cicer_arietinum               AATIAITISK.....LR...................................GKRFKLPP
sp|P37114.1_C4H_Medicago_sativa               AATIAVTISK.....LR...................................GKRFKLPP
sp|Q43067.2_C4H_Pisum_sativum                 AAITAITISK.....LR...................................GKPFKLPP
AAD11427.1_C4H_Mesembryanthemum_crystallinum  AISIIVLTTSSSNPNFSYYLAIFLP............IIVYLVHSICFHRA.QNSGTTPP
sp|O24315_C4H_Phaseolus_vulgaris              ......MTKL.....LHSYFSIPFSPFYVSIP..IATVLFVL.IIYNFFLASKNHSSTPP
AAK62344.1_C4H_Nicotiana_tabacum              TIAFLSFAKL.....LSSYLSMPFPLKYMSL......IVPLLPLIINFLYV.KPQNNLPP
AAK62345.1_C4H_Nicotiana_tabacum              SIVFLSFAKL.....LSSYLSIPFPLEYISL......IVLLLPLIINFLCV.KPQNNLPP
AAG17469.1_C4H_Triticum_aestivum              AAVLAIAVAK.....LT...................................GKRFRLPP
AAD23378.1_C4H_Pinus_taeda                    VIVGAIFISK.....LK...................................SKKLKLPP
                                         acc
```

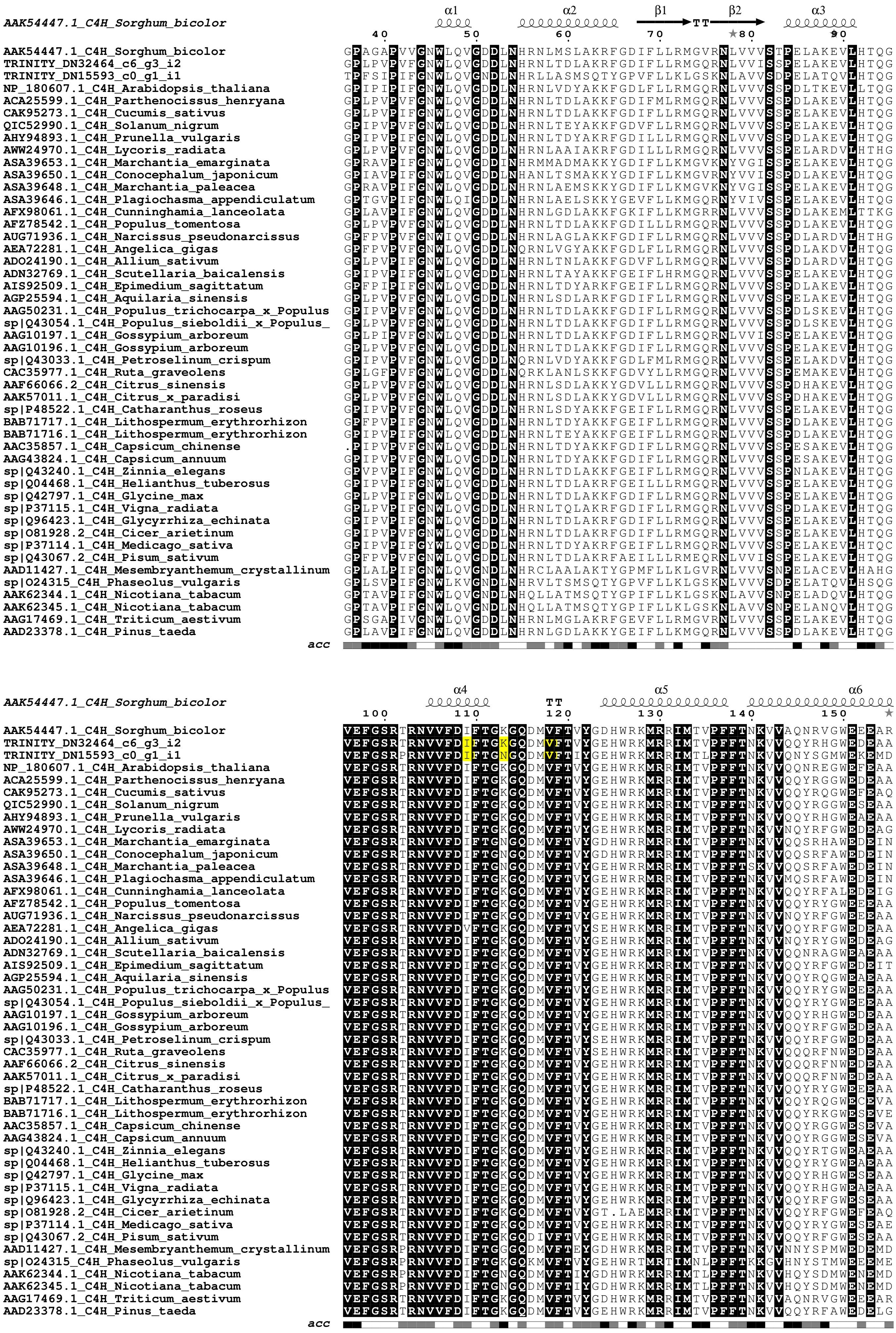

AAK54447.1_C4H_Sorghum_bicolor
α1
α2
β1
TT
β2
α3
40
50
60
70
80
90
acc
AAK54447.1_C4H_Sorghum_bicolor
α4
TT
α5
α6
100
110
120
130
140
150
acc

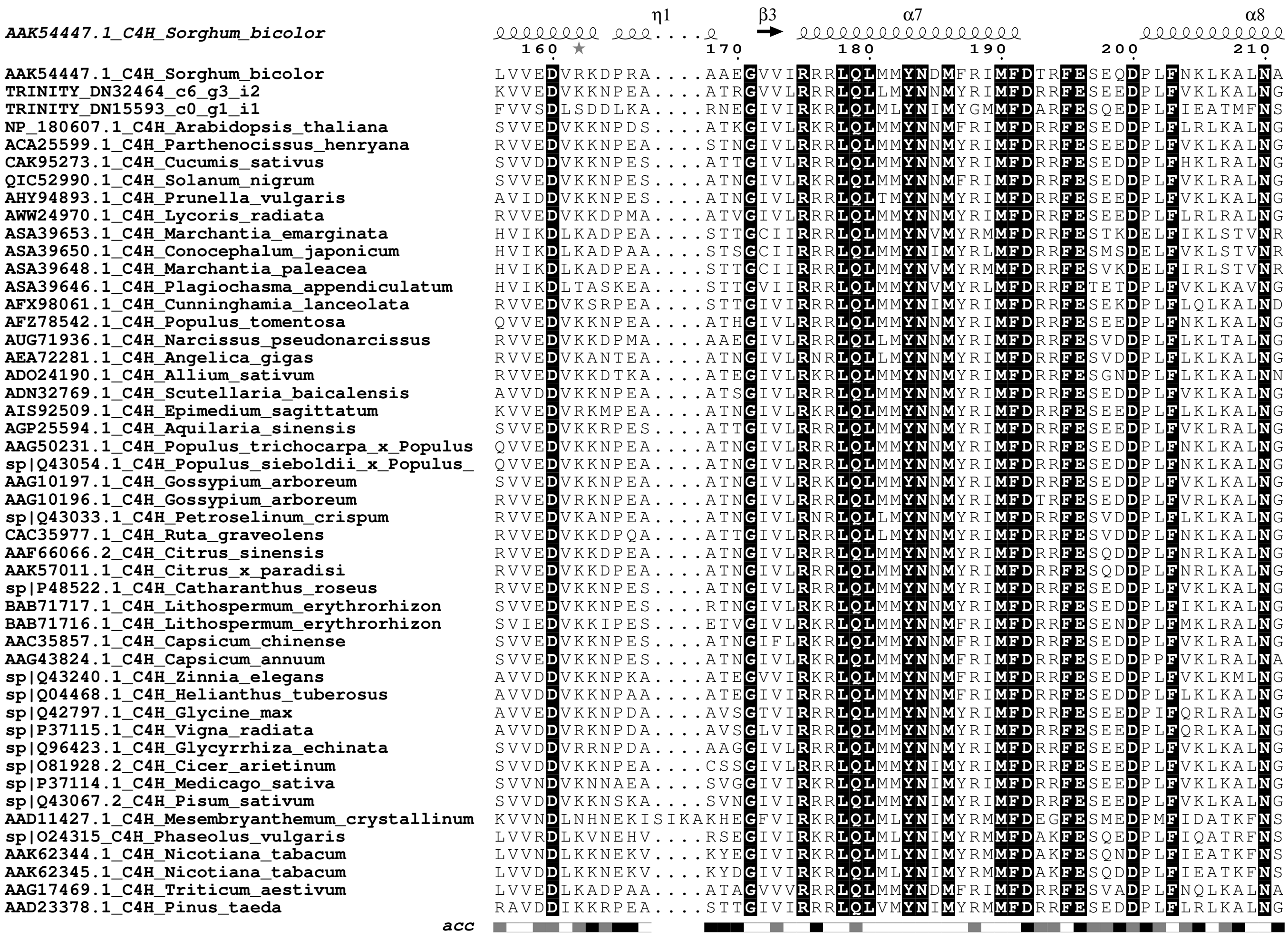
AAK54447.1_C4H_Sorghum_bicolor
η1
β3
α7
α8
acc

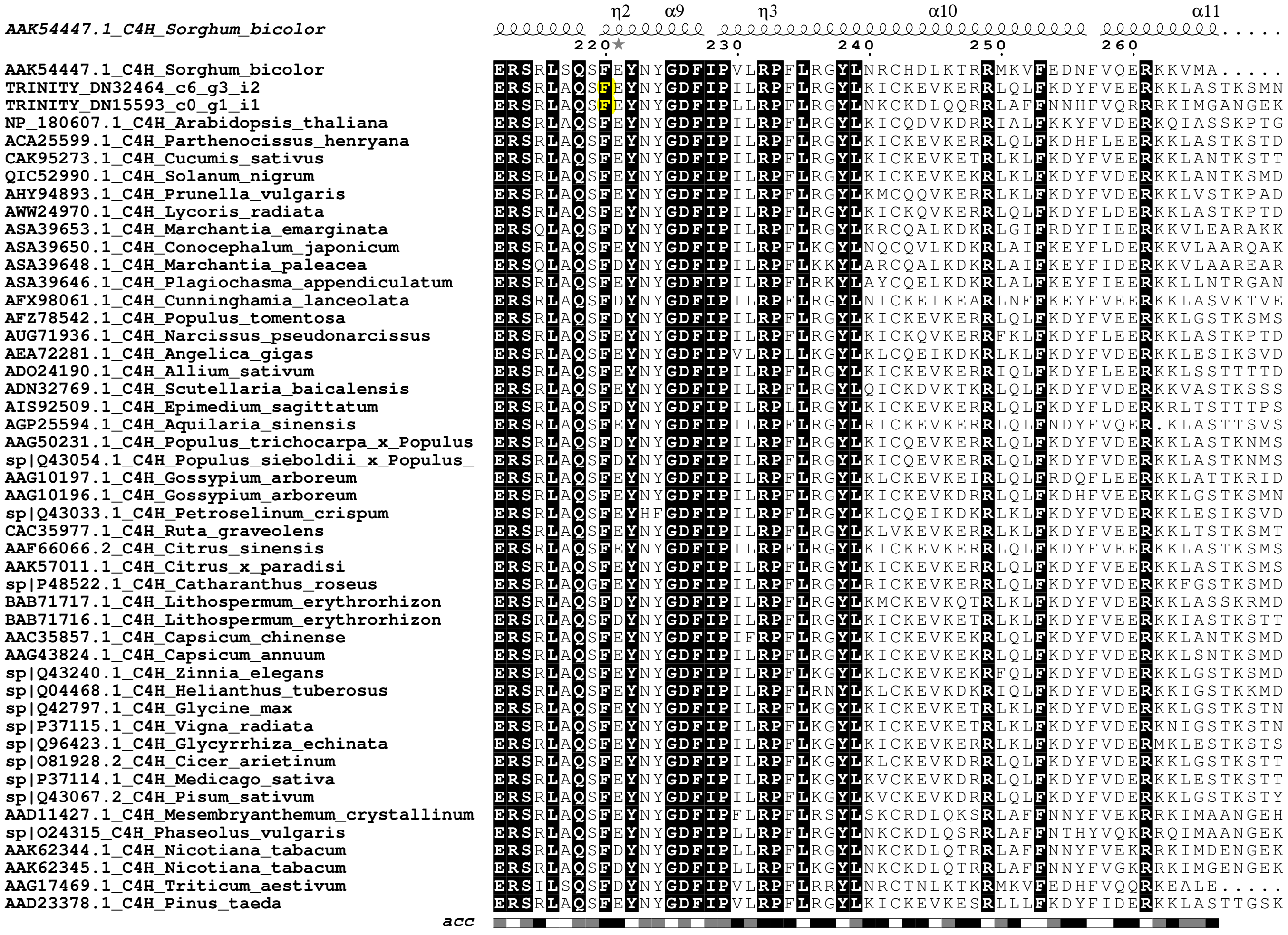
AAK54447.1_C4H_Sorghum_bicolor
η2
α9
η3
α10
α11
acc

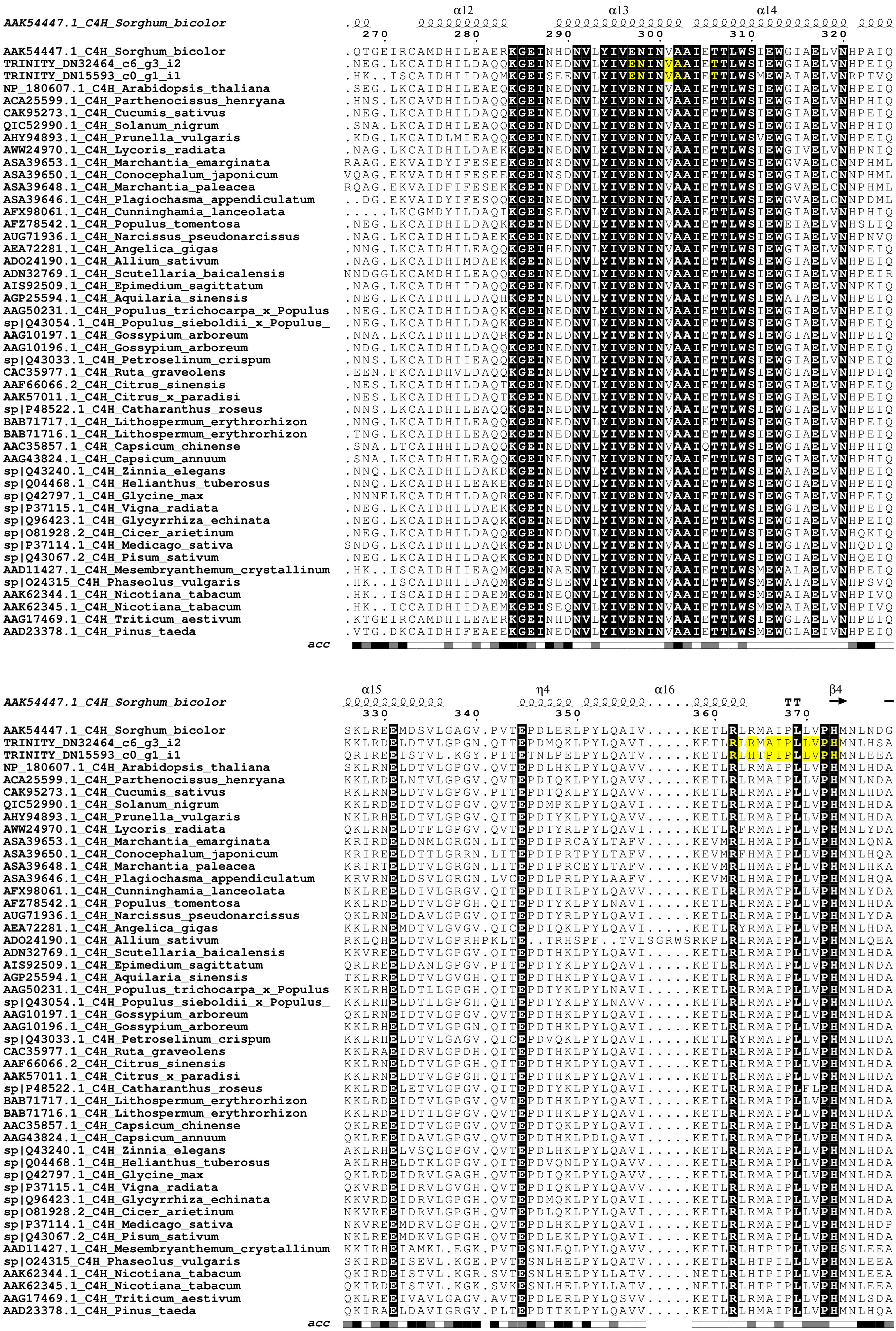

AAK54447.1_C4H_Sorghum_bicolor
α12 α13 α14
270 280 290 300 310 320
AAK54447.1_C4H_Sorghum_bicolor .QTGEIRCAMDHILEAERKGEINHDNVLYIVENINVAAIETTLWSIEWGIAELVNHPAIQ
TRINITY_DN32464_c6_g3_i2 .NEG.LKCAIDHILDAQQKGEINEDNVLYIVENINVAAIETTLWSIEWGIAELVNHPEIQ
TRINITY_DN15593_c0_g1_i1 .HK..ISCAIDHIIDAQMKGEISEENVLYIVENINVAAIETTLWSMEWAIAELVNRPTVQ
NP_180607.1_C4H_Arabidopsis_thaliana .SEG.LKCAIDHILEAEQKGEINEDNVLYIVENINVAAIETTLWSIEWGIAELVNHPEIQ
ACA25599.1_C4H_Parthenocissus_henryana .HNS.LKCAVDHILDAQQKGEINEDNVLYIVENINVAAIETTLWSIEWGIAELVNHPHIQ
CAK95273.1_C4H_Cucumis_sativus .NEG.LKCAIDHILDAQQKGEINEDNVLYIVENINVAAIETTLWSIEWGIAELVNHPEIQ
QIC52990.1_C4H_Solanum_nigrum .SNA.LKCAIDHILEAQQKGEINDDNVLYIVENINVAAIETTLWSIEWGIAELVNHPHIQ
AHY94893.1_C4H_Prunella_vulgaris .KDG.LKCAIDLMIEAQQKGEINEDNVLYIVENINVAAIETTLWSVEWGIAELVNHPEIQ
AWW24970.1_C4H_Lycoris_radiata .NAG.LKCAIDHILDAEKKGEINEDNVLYIVENINVAAIETTLWSIEWGIVELVNHPNIQ
ASA39653.1_C4H_Marchantia_emarginata RAAG.EKVAIDYIFESEEKGEINSDNVLYIVENINVAAIETTLWSIEWGVAELCNNPHML
ASA39650.1_C4H_Conocephalum_japonicum VQAG.EKVAIDHIFESEEKGEINSDNVLYIVENINVAAIETTLWSIEWGIAELCNNPHML
ASA39648.1_C4H_Marchantia_paleacea RQAG.EKVAIDFIFESEKKGEINFDNVLYIVENINVAAIETTLWSIEWGVAELCNNPHML
ASA39646.1_C4H_Plagiochasma_appendiculatum ..DG.EKVAIDYIFESQQKGEINEDNVLYIVENINVAAIETTLWSIEWGVAELCNNPDML
AFX98061.1_C4H_Cunninghamia_lanceolata .....LKCGMDYILDAQIKGEISEDNVLYIVENINAAAIETTLWSIEWGIAELVNHPHIQ
AFZ78542.1_C4H_Populus_tomentosa .NEG.LKCAIDHILDAQKKGEINEDNVLYIVENINVAAIETTLWSIEWEIAELVNHSLIQ
AUG71936.1_C4H_Narcissus_pseudonarcissus .NAG.LKCAIDHILDAEKKGEINEDNVLYIVENINVAAIETTLWSIEWGIAELVNHPNVQ
AEA72281.1_C4H_Angelica_gigas .NNG.LKCAIDHILEAQQKGEIHEDNVLYIVENINVAAIETTLWSIEWGIAELVNNPEIQ
ADO24190.1_C4H_Allium_sativum .NAG.LKCAIDHIMDAEKKGEINEDNVLYIVENINVAAIETTLWSIEWGIAELVNHPEIQ
ADN32769.1_C4H_Scutellaria_baicalensis NNDGGLKCAMDHILEAQQKGEINEDNVLYIVENINVAAIETTLWSIEWGIAELVNHPEIR
AIS92509.1_C4H_Epimedium_sagittatum .NAG.LKCAIDHIIDAQQKGEINEDNVLYIVENINVAAIETTLWSIEWGIAELVNNPKIQ
AGP25594.1_C4H_Aquilaria_sinensis .NEG.LKCAIDHILDAQHKGEINEDNVLYIVENINVAAIETTLWSIEWAIAELVNHPEIQ
AAG50231.1_C4H_Populus_trichocarpa_x_Populus .NEG.LKCAIDHILDAQKKGEINEDNVLYIVENINVAAIETTLWSIEWGIAELVNHPEIQ
sp|Q43054.1_C4H_Populus_sieboldii_x_Populus_ .NEG.LKCAIDHILDAQKKGEINEDNVLYIVENINVAAIETTLWSIEWGIAELVNHPEIQ
AAG10197.1_C4H_Gossypium_arboreum .NNA.LKCAIDHILDAQRKGEINEDNVLYIVENINVAAIETTLWSIEWGIAELVNHPEIQ
AAG10196.1_C4H_Gossypium_arboreum .NDG.LKCAIDHILDAQQKGEINEDNVLYIVENINVAAIETTLWSIEWGIAELVNHPEIQ
sp|Q43033.1_C4H_Petroselinum_crispum .NNS.LKCAIDHIIEAQQKGEINEDNVLYIVENINVAAIETTLWSIEWGIAELVNNPEIQ
CAC35977.1_C4H_Ruta_graveolens .EEN.FKCAIDHVLDAQQKGEINEDNVLYIVENINVAAIETTLWSIEWGIAELVNHPDIQ
AAF66066.2_C4H_Citrus_sinensis .NES.LKCAIDHILDAQTKGEINEDNVLYIVENINVAAIETTLWSIEWGIAELVNHPEIQ
AAK57011.1_C4H_Citrus_x_paradisi .NES.LKCAIDHILDAQTKGEINEDNVLYIVENINVAAIETTLWSIEWGIAELVNHPEIQ
sp|P48522.1_C4H_Catharanthus_roseus .NNS.LKCAIDHILEAQQKGEINEDNVLYIVENINVAAIETTLWSIEWGIAELVNHPEIQ
BAB71717.1_C4H_Lithospermum_erythrorhizon .NNG.LKCAIDHILEAQQKGEINEDNVLYIVENINVAAIETTLWSIEWGIAELVNHPEIQ
BAB71716.1_C4H_Lithospermum_erythrorhizon .TNG.LKCAIDHILEAQQKGEINEDNVLYIVENINVAAIETTLWSIEWGIAELVNHPEIQ
AAC35857.1_C4H_Capsicum_chinense .SNA.LTCAIHHILDAQQKGEINEDNVLYIVENINVAAIQTTLWSIEWGIAELVNHPHIQ
AAG43824.1_C4H_Capsicum_annuum .SNA.LKCAIDHILEAQQKGEINEDNVLYIVENINVAAIETTLWSIEWGIAELVNHPHIQ
sp|Q43240.1_C4H_Zinnia_elegans .NNQ.LKCAIDHILDAKDKGEINEDNVLYIVENINVAAIETTLWSIEWAIAELVNHPEIQ
sp|Q04468.1_C4H_Helianthus_tuberosus .NNQ.LKCAIDHILEAKEKGEINEDNVLYIVENINVAAIETTLWSIEWGIAELVNHPEIQ
sp|Q42797.1_C4H_Glycine_max .NNNELKCAIDHILDAQRKGEINEDNVLYIVENINVAAIETTLWSIEWGIAELVNHPEIQ
sp|P37115.1_C4H_Vigna_radiata .NEG.LKCAIDHILDAEKKGEINEDNVLYIVENINVAAIETTLWSIEWGIAELVNHPEIQ
sp|Q96423.1_C4H_Glycyrrhiza_echinata .NEG.LKCAIDHILDAQKKGEINEDNVLYIVENINVAAIETTLWSIEWGIAELVNHPEIQ
sp|O81928.2_C4H_Cicer_arietinum .NEG.LKCAIDHILDAQQKGEINEDNVLYIVENINVAAIETTLWSIEWGIAELVNHQKIQ
sp|P37114.1_C4H_Medicago_sativa SNDG.LKCAIDHILDAQKKGEINDDNVLYIVENINVAAIETTLWSIEWGIAELVNHQDIQ
sp|Q43067.2_C4H_Pisum_sativum .NEG.LKCAIDHILDAQKKGEINEDNVLYIVENINVAAIETTLWSIEWGIAELVNHQEIQ
AAD11427.1_C4H_Mesembryanthemum_crystallinum .HK..ISCAIDHIIEAQMKGEINAENVLYIVENINVAAIETTLWSMEWALAELVNHPEIQ
sp|O24315_C4H_Phaseolus_vulgaris .HK..ISCAIDHIIDAQMKGEISEENVIYIVENINVAAIETTLWSMEWAIAELVNHPSVQ
AAK62344.1_C4H_Nicotiana_tabacum .HK..ISCAIDHIIDAEMKGEINEQNVLYIVENINVAAIETTLWSMEWAIAELVNHPIVQ
AAK62345.1_C4H_Nicotiana_tabacum .HK..ICCAIDHIIDAEMKGEISEQNVLYIVENINVAAIETTLWSMEWAIAELVNHPIVQ
AAG17469.1_C4H_Triticum_aestivum .KTGEIRCAMDHILEAERKGEINHDNVLYIVENINVAAIETTLWSIEWGLAELVNHPEIQ
AAD23378.1_C4H_Pinus_taeda .VTG.DKCAIDHIFEAEEKGEINEDNVLYIVENINVAAIETTLWSMEWGLAEIVNHPEIQ
acc
AAK54447.1_C4H_Sorghum_bicolor
α15 η4 α16 TT β4
330 340 350 360 370
AAK54447.1_C4H_Sorghum_bicolor SKLREEMDSVLGAGV.PVTEPDLERLPYLQAIV.....KETLRLRMAIPLLVPHMNLNDG
TRINITY_DN32464_c6_g3_i2 KKLRDELDTVLGPGN.QITEPDMQKLPYLQAVI.....KETLRLRMAIPLLVPHMNLHSA
TRINITY_DN15593_c0_g1_i1 QRIREEISTVL.KGY.PITETNLPELPYLQATV.....KETLRLHTPIPLLVPHMNLEEA
NP_180607.1_C4H_Arabidopsis_thaliana SKLRNELDTVLGPGV.QVTEPDLHKLPYLQAVV.....KETLRLRMAIPLLVPHMNLHDA
ACA25599.1_C4H_Parthenocissus_henryana KKLRDELNTVLGPGV.QVTEPDIQKLPYLQAVV.....KETLRLRMAIPLLVPHMNLNDA
CAK95273.1_C4H_Cucumis_sativus RKLRNELDTVLGPGV.PITEPDTQKLPYLQAVV.....KETLRLRMAIPLLVPHMNLHDA
QIC52990.1_C4H_Solanum_nigrum KKLRDEIDTVLGPGV.QVTEPDMPKLPYLQAVI.....KETLRLRMAIPLLVPHMNLHDA
AHY94893.1_C4H_Prunella_vulgaris NKLRHELDTVLGPGV.QITEPDIYKLPYLQAVV.....KETLRLRMAIPLLVPHMNLYDA
AWW24970.1_C4H_Lycoris_radiata QKLRNELDTVLGPGV.QVTEPDTYRLPYLQAVI.....KETLRFRMAIPLLVPHMNLYDA
ASA39653.1_C4H_Marchantia_emarginata KRIRDELDNMLGRGN.LITEPDIPRCAYLTAFV.....KEVMRLHMAIPLLVPHMNLQNA
ASA39650.1_C4H_Conocephalum_japonicum KRIREELDTTLGRRN.LITEPDIPRTPYLTAFV.....KEVMRFHMAIPLLVPHMNLHQA
ASA39648.1_C4H_Marchantia_paleacea KRIRTELDTVLGRGN.LITEPDIPRCEYLTAFV.....KEVMRLHMAIPLLVPHMNLHKA
ASA39646.1_C4H_Plagiochasma_appendiculatum KRVRNELDSVLGRGN.LVCEPDLPRLPYLAAFV.....KEVMRLHMAIPLLVPHMNLHQA
AFX98061.1_C4H_Cunninghamia_lanceolata NKLREELDIVLGRGV.QITEPDIIRLPYLQAVV.....KETLRLRMATPLLVPHMNLYDA
AFZ78542.1_C4H_Populus_tomentosa KKLRDELDTVLGPGH.QITEPDTYKLPYLNAVI.....KETLRLRMAIPLLVPHMNLHDA
AUG71936.1_C4H_Narcissus_pseudonarcissus QKLRNELDAVLGPGV.QITEPDTYRLPYLQAVI.....KETLRLRMAIPLLVPHMNLYDA
AEA72281.1_C4H_Angelica_gigas KKLRNEMDTVLGVGV.QICEPDIQKLPYLQAVI.....KETLRYRMAIPLLVPHMNLHDA
ADO24190.1_C4H_Allium_sativum RKLQHELDTVLGPRHPKLTE..TRHSPF..TVLSGRWSRKPLRLRMAIPLLVPHMNLQEA
ADN32769.1_C4H_Scutellaria_baicalensis KKVREELDTVLGPGV.QITEPDTHKLPYLQAVI.....KETLRLRMAIPLLVPHMNLHDA
AIS92509.1_C4H_Epimedium_sagittatum QRLREELDANLGPGV.PITEPDTYKLPYLQAVI.....KETLRLRMAIPLLVPHMNLNDA
AGP25594.1_C4H_Aquilaria_sinensis TKLRRELDTVLGVGH.QITEPDTHKLPYLQAVI.....KETLRLRMAIPLLVPHMNLHDA
AAG50231.1_C4H_Populus_trichocarpa_x_Populus KKLRHELDTLLGPGH.QITEPDTYKLPYLNAVI.....KETLRLRMAIPLLVPHMNLHDA
sp|Q43054.1_C4H_Populus_sieboldii_x_Populus_ KKLRHELDTLLGPGH.QITEPDTYKLPYLNAVV.....KETLRLRMAIPLLVPHMNLHDA
AAG10197.1_C4H_Gossypium_arboreum QKLRNEIDTVLGPGV.QVTEPDTHKLPYLQAVI.....KETLRLRMAIPLLVPHMNLHDA
AAG10196.1_C4H_Gossypium_arboreum KKLRHELDTVLGPGN.QITEPDTHKLPYLQAVI.....KETLRLRMAIPLLVPHMNLHDA
sp|Q43033.1_C4H_Petroselinum_crispum KKLRHELDTVLGAGV.QICEPDVQKLPYLQAVI.....KETLRYRMAIPLLVPHMNLHDA
CAC35977.1_C4H_Ruta_graveolens KKLRAEIDRVLGPDH.QITEPDTHKLPYLQAVI.....KETLRLRMAIPLLVPHMNLNDA
AAF66066.2_C4H_Citrus_sinensis KKLRNELDTVLGPGH.QITEPDTHKLPYLQAVI.....KETLRLRMAIPLLVPHMNLHDA
AAK57011.1_C4H_Citrus_x_paradisi KKLRNELDTVLGPGH.QITEPDTHKLPYLQAVI.....KETLRLRMAIPLLVPHMNLHDA
sp|P48522.1_C4H_Catharanthus_roseus KKLRDELETVLGPGV.QITEPDTYKLPYLQAVI.....KETLRLRMAIPLFLPHMNLHDA
BAB71717.1_C4H_Lithospermum_erythrorhizon KKLRDEIDTVLGPGV.QVTEPDTHKLPYLQAVI.....KETLRLRMAIPLLVPHMNLHDA
BAB71716.1_C4H_Lithospermum_erythrorhizon KKLRDEIDTILGPGV.QVTEPDTHKLPYLQAVI.....KETLRLRMAIPLLVPHMNLHDA
AAC35857.1_C4H_Capsicum_chinense QKLREEIDTVLGPGV.QVTEPDTQKLPYLQAVI.....KETLRLRMAIPLLVPHMSLHDA
AAG43824.1_C4H_Capsicum_annuum QKLREEIDAVLGPGV.QVTEPDTHKLPDLQAVI.....KETLRLRMAIPLLVPHMNIHDA
sp|Q43240.1_C4H_Zinnia_elegans AKLRHELVSQLGPGV.QVTEPDLHKLPYLQAVI.....KETLRLRMAIPLLVPHMNLHDA
sp|Q04468.1_C4H_Helianthus_tuberosus AKLRHELDTKLGPGV.QITEPDVQNLPYLQAVV.....KETLRLRMAIPLLVPHMNLHDA
sp|Q42797.1_C4H_Glycine_max QKLRDEIDRVLGAGH.QVTEPDIQKLPYLQAVV.....KETLRLRMAIPLLVPHMNLHDA
sp|P37115.1_C4H_Vigna_radiata QKVRDEIDRVLGVGH.QVTEPDIQKLPYLQAVV.....KETLRLRMAIPLLVPHMNLHDA
sp|Q96423.1_C4H_Glycyrrhiza_echinata KKVRDEIDRVLGPGH.QVTEPDMQKLPYLQAVI.....KETLRLRMAIPLLVPHMNLHDA
sp|O81928.2_C4H_Cicer_arietinum NKVREEIDRVLGPGH.QVTEPDLQKLPYLQAVI.....KETLRLRMAIPLLVPHMNLHDA
sp|P37114.1_C4H_Medicago_sativa NKVREEMDRVLGPGH.QVTEPDLHKLPYLQAVI.....KETLRLRMAIPLLVPHMNLHDP
sp|Q43067.2_C4H_Pisum_sativum NKLREEMDKVLGPGH.QVTEPDLEKLPYLQAVI.....KETLRLRMAIPLLVPHMNLHDA
AAD11427.1_C4H_Mesembryanthemum_crystallinum KKIRHEIAMKL.EGK.PVTESNLEQLPYLQAVV.....KETLRLHTPIPLLVPHSNLEEA
sp|O24315_C4H_Phaseolus_vulgaris SKIRDEISEVL.KGE.PVTESNLHELPYLQATV.....KETLRLHTPILLLVPHMNLEEA
AAK62344.1_C4H_Nicotiana_tabacum QKIRDEISTVL.KGR.SVTESNLHELPYLLATV.....NETLRLHTPIPLLVPHMNLEEA
AAK62345.1_C4H_Nicotiana_tabacum QKIRDEISTVL.KGK.SVKESNLHELPYLLATV.....NETLRLHTPIPLLVPHMNLEEA
AAG17469.1_C4H_Triticum_aestivum QKLREEIVAVLGAGV.AVTEPDLERLPYLQSVV.....KETLRLRMAIPLLVPHMNLSDA
AAD23378.1_C4H_Pinus_taeda QKIRAELDAVIGRGV.PLTEPDTTKLPYLQAVV.....KETLRLHMAIPLLVPHMNLHQA
acc

```
                                                β5     β6
AAK54447.1_C4H_Sorghum_bicolor                  →TT→   TT ──
                                               380        390
                                                 .         .
AAK54447.1_C4H_Sorghum_bicolor                  KLAGYDIPAESKIL..............................................
TRINITY_DN32464_c6_g3_i2                        KLAGYDIPAESKIL..............................................
TRINITY_DN15593_c0_g1_i1                        KLGGFTIPKESKVV..............................................
NP_180607.1_C4H_Arabidopsis_thaliana            KLAGYDIPAESKIL..............................................
ACA25599.1_C4H_Parthenocissus_henryana          KLGSYDIPAESKIL..............................................
CAK95273.1_C4H_Cucumis_sativus                  KLAGYDIPAESKIL..............................................
QIC52990.1_C4H_Solanum_nigrum                   KLAGYDIPAESKIL..............................................
AHY94893.1_C4H_Prunella_vulgaris                KLGGFDIPAESKVL..............................................
AWW24970.1_C4H_Lycoris_radiata                  KLGGYDIPAESKIL..............................................
ASA39653.1_C4H_Marchantia_emarginata            KLGGYDIPAESKIL..............................................
ASA39650.1_C4H_Conocephalum_japonicum           KLGGYDIPAESKIL..............................................
ASA39648.1_C4H_Marchantia_paleacea              KLAGYDIPAESKIL..............................................
ASA39646.1_C4H_Plagiochasma_appendiculatum      KLAGYDIPAESKIL..............................................
AFX98061.1_C4H_Cunninghamia_lanceolata          KLGGYDIPAESKIL..............................................
AFZ78542.1_C4H_Populus_tomentosa                KLGGFDIPAESKIL..............................................
AUG71936.1_C4H_Narcissus_pseudonarcissus        KLGSYDIPAESKIL..............................................
AEA72281.1_C4H_Angelica_gigas                   KLAGYDIPAESKILVNAWWLANNPAHWKNPNEFRPERFLEEESKVEANGNDFKYIPFGVG
ADO24190.1_C4H_Allium_sativum                   KLSGYDIPAESKIL..............................................
ADN32769.1_C4H_Scutellaria_baicalensis          KLNGYDIPAESKIL..............................................
AIS92509.1_C4H_Epimedium_sagittatum             KLNGFDIPAESKIL..............................................
AGP25594.1_C4H_Aquilaria_sinensis               KLGGYDIPAESKIL..............................................
AAG50231.1_C4H_Populus_trichocarpa_x_Populus    KLGGFDIPAESKIL..............................................
sp|Q43054.1_C4H_Populus_sieboldii_x_Populus_    KLGGFDIPAESKIL..............................................
AAG10197.1_C4H_Gossypium_arboreum               KLGGYDIPAESKIL..............................................
AAG10196.1_C4H_Gossypium_arboreum               KLGGYDIPAESKIL..............................................
sp|Q43033.1_C4H_Petroselinum_crispum            KLAGYDIPAESKIL..............................................
CAC35977.1_C4H_Ruta_graveolens                  KLAGYDIPAESKIL..............................................
AAF66066.2_C4H_Citrus_sinensis                  KLGGYDVPAESKIL..............................................
AAK57011.1_C4H_Citrus_x_paradisi                KLGGYDVPAESKIL..............................................
sp|P48522.1_C4H_Catharanthus_roseus             KLGGYDIPAESKIL..............................................
BAB71717.1_C4H_Lithospermum_erythrorhizon       KLGEYDIPAESKSL..............................................
BAB71716.1_C4H_Lithospermum_erythrorhizon       KLNGYDIPAESKIL..............................................
AAC35857.1_C4H_Capsicum_chinense                KLAVYDIPAESKIL..............................................
AAG43824.1_C4H_Capsicum_annuum                  KLAGYDIPAESKIL..............................................
sp|Q43240.1_C4H_Zinnia_elegans                  KLGGYDIPAESKIL..............................................
sp|Q04468.1_C4H_Helianthus_tuberosus            KLGGFDIPAESKIL..............................................
sp|Q42797.1_C4H_Glycine_max                     KLGGYDIPAESKIL..............................................
sp|P37115.1_C4H_Vigna_radiata                   KLGGYDIPAESKIL..............................................
sp|Q96423.1_C4H_Glycyrrhiza_echinata            KLGGYDIPAESKIL..............................................
sp|O81928.2_C4H_Cicer_arietinum                 KLSGFDIPAESKIL..............................................
sp|P37114.1_C4H_Medicago_sativa                 KLNGFDIPAESKIL..............................................
sp|Q43067.2_C4H_Pisum_sativum                   KLGGFDIPAESKIL..............................................
AAD11427.1_C4H_Mesembryanthemum_crystallinum    KLGGYTIPKNSKVV..............................................
sp|O24315_C4H_Phaseolus_vulgaris                KLGGYTVPKESKVV..............................................
AAK62344.1_C4H_Nicotiana_tabacum                KLGGYTIPKETKVV..............................................
AAK62345.1_C4H_Nicotiana_tabacum                KLGGYTIPKETKVV..............................................
AAG17469.1_C4H_Triticum_aestivum                KLAGYDIPAESKIL..............................................
AAD23378.1_C4H_Pinus_taeda                      KLGGYDIPAESKIL..............................................
                                           acc
```

```
                                                                                   β7
AAK54447.1_C4H_Sorghum_bicolor                  ............................................................

AAK54447.1_C4H_Sorghum_bicolor                  ............................................................
TRINITY_DN32464_c6_g3_i2                        ............................................................
TRINITY_DN15593_c0_g1_i1                        ............................................................
NP_180607.1_C4H_Arabidopsis_thaliana            ............................................................
ACA25599.1_C4H_Parthenocissus_henryana          ............................................................
CAK95273.1_C4H_Cucumis_sativus                  ............................................................
QIC52990.1_C4H_Solanum_nigrum                   ............................................................
AHY94893.1_C4H_Prunella_vulgaris                ............................................................
AWW24970.1_C4H_Lycoris_radiata                  ............................................................
ASA39653.1_C4H_Marchantia_emarginata            ............................................................
ASA39650.1_C4H_Conocephalum_japonicum           ............................................................
ASA39648.1_C4H_Marchantia_paleacea              ............................................................
ASA39646.1_C4H_Plagiochasma_appendiculatum      ............................................................
AFX98061.1_C4H_Cunninghamia_lanceolata          ............................................................
AFZ78542.1_C4H_Populus_tomentosa                ............................................................
AUG71936.1_C4H_Narcissus_pseudonarcissus        ............................................................
AEA72281.1_C4H_Angelica_gigas                   RRSCPGIILALPILGITIGRMVQNFELLPPPGMSKIDTAEKAGQFSLQISKHYTIVCKPR
ADO24190.1_C4H_Allium_sativum                   ............................................................
ADN32769.1_C4H_Scutellaria_baicalensis          ............................................................
AIS92509.1_C4H_Epimedium_sagittatum             ............................................................
AGP25594.1_C4H_Aquilaria_sinensis               ............................................................
AAG50231.1_C4H_Populus_trichocarpa_x_Populus    ............................................................
sp|Q43054.1_C4H_Populus_sieboldii_x_Populus_    ............................................................
AAG10197.1_C4H_Gossypium_arboreum               ............................................................
AAG10196.1_C4H_Gossypium_arboreum               ............................................................
sp|Q43033.1_C4H_Petroselinum_crispum            ............................................................
CAC35977.1_C4H_Ruta_graveolens                  ............................................................
AAF66066.2_C4H_Citrus_sinensis                  ............................................................
AAK57011.1_C4H_Citrus_x_paradisi                ............................................................
sp|P48522.1_C4H_Catharanthus_roseus             ............................................................
BAB71717.1_C4H_Lithospermum_erythrorhizon       ............................................................
BAB71716.1_C4H_Lithospermum_erythrorhizon       ............................................................
AAC35857.1_C4H_Capsicum_chinense                ............................................................
AAG43824.1_C4H_Capsicum_annuum                  ............................................................
sp|Q43240.1_C4H_Zinnia_elegans                  ............................................................
sp|Q04468.1_C4H_Helianthus_tuberosus            ............................................................
sp|Q42797.1_C4H_Glycine_max                     ............................................................
sp|P37115.1_C4H_Vigna_radiata                   ............................................................
sp|Q96423.1_C4H_Glycyrrhiza_echinata            ............................................................
sp|O81928.2_C4H_Cicer_arietinum                 ............................................................
sp|P37114.1_C4H_Medicago_sativa                 ............................................................
sp|Q43067.2_C4H_Pisum_sativum                   ............................................................
AAD11427.1_C4H_Mesembryanthemum_crystallinum    ............................................................
sp|O24315_C4H_Phaseolus_vulgaris                ............................................................
AAK62344.1_C4H_Nicotiana_tabacum                ............................................................
AAK62345.1_C4H_Nicotiana_tabacum                ............................................................
AAG17469.1_C4H_Triticum_aestivum                ............................................................
AAD23378.1_C4H_Pinus_taeda                      ............................................................
                                           acc
```

```
                                                                                                                   α17
AAK54447.1_C4H_Sorghum_bicolor               .....................................................►  ℓℓℓℓℓ
                                                                                                       400
AAK54447.1_C4H_Sorghum_bicolor               .....................................................VNAWFLA
TRINITY_DN32464_c6_g3_i2                     .....................................................VNAWWLA
TRINITY_DN15593_c0_g1_i1                     .....................................................VNAWWLA
NP_180607.1_C4H_Arabidopsis_thaliana         .....................................................VNAWWLA
ACA25599.1_C4H_Parthenocissus_henryana       .....................................................VNAWWLA
CAK95273.1_C4H_Cucumis_sativus               .....................................................VNAWWLA
QIC52990.1_C4H_Solanum_nigrum                .....................................................VNAWWLA
AHY94893.1_C4H_Prunella_vulgaris             .....................................................VNAWWLA
AWW24970.1_C4H_Lycoris_radiata               .....................................................VNAWWLA
ASA39653.1_C4H_Marchantia_emarginata         .....................................................VNAWWIA
ASA39650.1_C4H_Conocephalum_japonicum        .....................................................VNAWWIA
ASA39648.1_C4H_Marchantia_paleacea           .....................................................VNAWWIA
ASA39646.1_C4H_Plagiochasma_appendiculatum   .....................................................VNAWWIG
AFX98061.1_C4H_Cunninghamia_lanceolata       .....................................................VNSWYLA
AFZ78542.1_C4H_Populus_tomentosa             .....................................................VNAWWLA
AUG71936.1_C4H_Narcissus_pseudonarcissus     .....................................................VNAWWLA
AEA72281.1_C4H_Angelica_gigas                SLTESNLHELPYLQATVKETLRLHTPILLLVPHMNLEEAKLGGYTVPKESKVVVNAWWLA
ADO24190.1_C4H_Allium_sativum                .....................................................VNAWWLA
ADN32769.1_C4H_Scutellaria_baicalensis       .....................................................VNAWWLA
AIS92509.1_C4H_Epimedium_sagittatum          .....................................................VNAWWLA
AGP25594.1_C4H_Aquilaria_sinensis            .....................................................VNAWWLA
AAG50231.1_C4H_Populus_trichocarpa_x_Populus .....................................................VNAWWLA
sp|Q43054.1_C4H_Populus_sieboldii_x_Populus_ .....................................................VNAWWLA
AAG10197.1_C4H_Gossypium_arboreum            .....................................................VNAWWLA
AAG10196.1_C4H_Gossypium_arboreum            .....................................................VNAWWLA
sp|Q43033.1_C4H_Petroselinum_crispum         .....................................................VNAWWLA
CAC35977.1_C4H_Ruta_graveolens               .....................................................VNAWWLA
AAF66066.2_C4H_Citrus_sinensis               .....................................................VNAWWLA
AAK57011.1_C4H_Citrus_x_paradisi             .....................................................VNAWWLA
sp|P48522.1_C4H_Catharanthus_roseus          .....................................................VNAWFLA
BAB71717.1_C4H_Lithospermum_erythrorhizon    .....................................................VNAWWLA
BAB71716.1_C4H_Lithospermum_erythrorhizon    .....................................................VNAWWLA
AAC35857.1_C4H_Capsicum_chinense             .....................................................VNAWWLA
AAG43824.1_C4H_Capsicum_annuum               .....................................................VNPWWLA
sp|Q43240.1_C4H_Zinnia_elegans               .....................................................VNAWWLA
sp|Q04468.1_C4H_Helianthus_tuberosus         .....................................................VNAWWLA
sp|Q42797.1_C4H_Glycine_max                  .....................................................VNAWWLA
sp|P37115.1_C4H_Vigna_radiata                .....................................................VNAWWLA
sp|Q96423.1_C4H_Glycyrrhiza_echinata         .....................................................VNAWWLA
sp|O81928.2_C4H_Cicer_arietinum              .....................................................VNAWWLA
sp|P37114.1_C4H_Medicago_sativa              .....................................................VNAWWLP
sp|Q43067.2_C4H_Pisum_sativum                .....................................................VNAWWLA
AAD11427.1_C4H_Mesembryanthemum_crystallinum .....................................................VNAWWLA
sp|O24315_C4H_Phaseolus_vulgaris             .....................................................VNAWWLA
AAK62344.1_C4H_Nicotiana_tabacum             .....................................................VNAWWLA
AAK62345.1_C4H_Nicotiana_tabacum             .....................................................VNAWWLA
AAG17469.1_C4H_Triticum_aestivum             .....................................................VNAWFLA
AAD23378.1_C4H_Pinus_taeda                   .....................................................VNAWFLA
                                         acc
```

```
                                                           η5                                                  α18
AAK54447.1_C4H_Sorghum_bicolor               ℓ        TT    ℓℓℓℓℓ          TT         TT   TT   TT     ℓℓℓℓℓℓℓℓℓ
                                                   410        420                  430        440        450
AAK54447.1_C4H_Sorghum_bicolor               NDPKRWVRPDEFRPERFLEEEKTVE...AHGN...DFRFVPFGVGRRSCPGIILALPIIG
TRINITY_DN32464_c6_g3_i2                     NNPAHWKNPEEFRPERFLEEESKVE...ANGN...DFRYLPFGVGRRSCPGIILALPILG
TRINITY_DN15593_c0_g1_i1                     NNPKWWKNPEEFRPERFFEEESGTE.AVAGGK..VDFRYLPFGVGRRSCPGIILALPILG
NP_180607.1_C4H_Arabidopsis_thaliana         NNPNSWKKPEEFRPERFFEEESHVE...ANGN...DFRYVPFGVGRRSCPGIILALPILG
ACA25599.1_C4H_Parthenocissus_henryana       NNPSKWKNPEEFRPERFLEEESKVE...PNGN...DFRYLPFGVGRRSCPGIILALPILG
CAK95273.1_C4H_Cucumis_sativus               NNPANWKNPEEFRPERFLEEESKVE...ANGN...DFKYLPFGAGRRSCPGIILALPILG
QIC52990.1_C4H_Solanum_nigrum                NNPAHWKKPEEFRPERFFEEEKHVE...ANGN...DFRFLPFGVGRRSCPGIILALPILG
AHY94893.1_C4H_Prunella_vulgaris             NNPAQWKKPEEFRPERFLEEEAKVE...ANGN...DFRYLPFGVGRRSCPGIILALPILG
AWW24970.1_C4H_Lycoris_radiata               NNPAHWKDPEEFRPERFLEEEAKVE...ANGN...DFRYIPFGVGRRSCPGIILALPILG
ASA39653.1_C4H_Marchantia_emarginata         NNPKYWTEPENFNPDRFLDE..SID...AAGS...DFRFLPFGAGRRSCPGIIVAMPLLH
ASA39650.1_C4H_Conocephalum_japonicum        NNPEYWEEPEKFNPDRFLDG..KID...AAGN...DFRFLPFGSGRRSCPGIIIAMPLLH
ASA39648.1_C4H_Marchantia_paleacea           NNPAYWTEPEKFNPNRFMNE..SID...AAGS...DFRFLPFGAGRRSCPGIIVAMPLLH
ASA39646.1_C4H_Plagiochasma_appendiculatum   NNPAYWDEPERFNPDRFLDG..NIE...SNGN...DFRFLPFGSGRRSCPGIIIAIPLLS
AFX98061.1_C4H_Cunninghamia_lanceolata       NNPAEWDNPQQFRPERFLEEEKKTE...ANGN...DFRYLPFGVGRRSCPGVILALPILG
AFZ78542.1_C4H_Populus_tomentosa             NNPAKWKNPEEFRPERFFEEEAKVE...ANGN...DFRYLPFGVGRRSCPGIILALPILG
AUG71936.1_C4H_Narcissus_pseudonarcissus     NNPAHWKDPQEFRPERFLEEEAKVE...ANGN...DFRYIPFGVGRRSCPGIILALPILG
AEA72281.1_C4H_Angelica_gigas                NNPSWWKNPEEFRPERFLEEECATD...AVAGGKVDFRFVPFGVGRRSCPGIILALPILG
ADO24190.1_C4H_Allium_sativum                NNPAHWKDPEEFRPERFLEEEAKVE...VNGN...DFRYIPFGVGRRSCPGIILALPILG
ADN32769.1_C4H_Scutellaria_baicalensis       NNPAQWKKPEEFRPERFLEEEAKVE...ANGN...DFRYLPFGVGRRSCPGIILALPILC
AIS92509.1_C4H_Epimedium_sagittatum          NNPEHWKNPEEFRPERFLEEESKVE...ANGN...DFRYLPFGVGRRSCPGIILALPILG
AGP25594.1_C4H_Aquilaria_sinensis            NNPANWDRSEEFRPERFLEEESKVE...ANGN...DFRFLPFGVGRRSCPGIILALPILG
AAG50231.1_C4H_Populus_trichocarpa_x_Populus NNPAHWKNPEEFRPERFLEEEAKVE...ANGN...DFRYLPFGVGRRSCPGIILALPILG
sp|Q43054.1_C4H_Populus_sieboldii_x_Populus_ NNPAHWKNPEEFRPERFLEEGAKVE...ANGN...DFRYLPFGVGRRSCPGIILALPILG
AAG10197.1_C4H_Gossypium_arboreum            NNPAHWKNPEEFRPERFFEEESKVE...ANGN...DFRYLPFGVGRRSCPGIILALPILG
AAG10196.1_C4H_Gossypium_arboreum            NNPANWKNPEEFRPERFFEEEAKVE...ANGN...DFRYLPFGVGRRSCPGIILALPILG
sp|Q43033.1_C4H_Petroselinum_crispum         NNPAHWNKPDEFRPERFLEEESKVE...ANGN...DFKYIPFGVGRRSCPGIILALPILG
CAC35977.1_C4H_Ruta_graveolens               NNPAHWKDPQVFRPERFLEEESGVE...ANGN...DFRYIPFGVGRRSCPGIILALPILG
AAF66066.2_C4H_Citrus_sinensis               NNPAQWKKPEEFRPERFLEEESKVE...ANGN...DFRYLPFGVGRRSCPGIILALPILG
AAK57011.1_C4H_Citrus_x_paradisi             NNPAQWKKPEEFRPERFLEEESKVE...ANGN...DFRYLPFGVGRRSCPGIILALPILG
sp|P48522.1_C4H_Catharanthus_roseus          NNPEHWKKPEEFRPERFLEEESKVE...ANGN...DFRYLPFGVGRRSCPGIILALPILG
BAB71717.1_C4H_Lithospermum_erythrorhizon    NNPTQWKNPEEFRPERFLEEEAKVE...ASGN...DFRYLPFGVGRRSCPGIILALPILG
BAB71716.1_C4H_Lithospermum_erythrorhizon    NNPAQWKNPEEFRPERFLEEEAKVE...ANGN...DFRYLPFGVGRRSCPGIILALPILG
AAC35857.1_C4H_Capsicum_chinense             NNPAHWKKPEEFRPERFLEEEKHVD...ANGN...DFRFLPFGVGRRSCPGIILALPILG
AAG43824.1_C4H_Capsicum_annuum               NNPAHWKKPEEFRPERFLKEEKHVD...ANGN...DFRFLPFGVGRRSCPGIILALPILG
sp|Q43240.1_C4H_Zinnia_elegans               NNPDQWKKPEEFRPERFLEEESKVE...ANGN...DFRYLPFGVGRRSCPGIILALPILG
sp|Q04468.1_C4H_Helianthus_tuberosus         NNPDQWKKPEEFRPERFLEEEAKVE...ANGN...DFRYLPFGVGRRSCPGIILALPILG
sp|Q42797.1_C4H_Glycine_max                  NNPAHWKKPEEFRPERFFEEESLVE...ANGN...DFRYLPFGVGRRSCPGIILALPILG
sp|P37115.1_C4H_Vigna_radiata                NNPAHWKKPEEFRPERFFEEESHVE...ANGN...DFRYLPFGVGRRSCPGIILALPILG
sp|Q96423.1_C4H_Glycyrrhiza_echinata         NNPANWKRPEEFRPERFLEEESHVE...ANGN...DFRYLPFGVGRRSCPGIILALPILG
sp|O81928.2_C4H_Cicer_arietinum              NNPAQWKKPEEFRPERFLEEESHVE...ANGN...DFRYLPFGVGRRSCPGIILALPILG
sp|P37114.1_C4H_Medicago_sativa              NNPAHWKKPEEFRPERFLEEESHVE...ANGN...DFRYLPFGVGRRSCPGIILALPILG
sp|Q43067.2_C4H_Pisum_sativum                NNPALWKKPEEFRPERFLEEEAHVE...ANGN...DFRYLPFGVGRRSCPGIILALPILG
AAD11427.1_C4H_Mesembryanthemum_crystallinum NNPEWWRDAEEFRPERFLEEEAGADAAVGGGK..VDFRFVPFGVGRRSCPGIILALPILG
sp|O24315_C4H_Phaseolus_vulgaris             NNPSWWKNPEEFRPERFLEEECATD.AVAGGK..VDFRFVPFGVGRRSCPGIILALPILG
AAK62344.1_C4H_Nicotiana_tabacum             NNPAWWKNPNEFRPERFLEEDSSTEAAVAGGK..VDFRYLPFGMGRRSCPGIILALPILG
AAK62345.1_C4H_Nicotiana_tabacum             NNPAWWKNQNEFRPERFLEEDSSTEAAVAGGK..VDFRYLPFGMGRRSCPGIILALPILG
AAG17469.1_C4H_Triticum_aestivum             NDPKRWVRADEFRPERFLEEEKAVE...AHGN...DFRFVPFGVGRRSCPGIILALPIIG
AAD23378.1_C4H_Pinus_taeda                   NNPEWWEKPEEFIPERFLGEE.KIE...ASGN...DFRFLPFGVGRRSCPGIILALPILA
                                         acc
```

```
                                                   β8                β9     β10        β11
AAK54447.1_C4H_Sorghum_bicolor                                     TT                                  TT
                                              460          470           480           490           500
AAK54447.1_C4H_Sorghum_bicolor                ITLGRLVQNFQLLPPPGQDKIDTTEKPGQFSNQIAKHATIVCKPLEA..
TRINITY_DN32464_c6_g3_i2                      ITLGRLVQNFELLPPPGQSKLDTTEKGGQFSLHILKHSTIVAKP.RSF.
TRINITY_DN15593_c0_g1_i1                      LVIGKLVTNFEMKAPKGIDNIDVSEKGGQFSLHIANHSTVVFEPIK...
NP_180607.1_C4H_Arabidopsis_thaliana          ITIGRMVQNFELLPPPGQSKVDTSEKGGQFSLHILNHSIIVMKP.RNC.
ACA25599.1_C4H_Parthenocissus_henryana        ITIGRMVQNFELLPPPGQNKLDTTEKGGQFSLHILKHSTIVAKP.IEA.
CAK95273.1_C4H_Cucumis_sativus                ITIGRLVQNFELLPPPGQSKLDTSEKGGQFSLHILKHSTIVVKP.RVF.
QIC52990.1_C4H_Solanum_nigrum                 ITLGRLVQNFEMLPPPGQSKIDTSEKGGQFSLHILKHSTIVMKP.RSF.
AHY94893.1_C4H_Prunella_vulgaris              ITLGRLVQNFELLPPPGQKKIGTSEKGGQFSLHILKHSTIVMKP.ISS.
AWW24970.1_C4H_Lycoris_radiata                ITIGRLVQNFELLPPPGQDKLDTTEKGGQFSLHILKHSTIVAKP.RVF.
ASA39653.1_C4H_Marchantia_emarginata          LVLGRLVQSFDLSPPPGVDKIDMTEKGGQFSLFIKNHYNVVVKS.RKD.
ASA39650.1_C4H_Conocephalum_japonicum         LVFGRLVQTFDLFTPPGVDKIDMTEKGGQFSLFIKNHYNIVVKD.RKD.
ASA39648.1_C4H_Marchantia_paleacea            LVLGRLVQTFDFYPPPGVDKIDMTEKGGQFSLFIKNHYNIVVKD.RKD.
ASA39646.1_C4H_Plagiochasma_appendiculatum    LVLGRLVQTFDLLPPPGVDKIDMTEKGGQFSLFIKTHYNIVVKP.RSD.
AFX98061.1_C4H_Cunninghamia_lanceolata        LSLGRLVQNFELLPPPGHTRLDTTEKAGQFSLKILNHSLIVAKP.RVF.
AFZ78542.1_C4H_Populus_tomentosa              ITLGRLVQNFELLPPPGQSKIDTSEKGGQFSLHILKHSTIVAKP.RSF.
AUG71936.1_C4H_Narcissus_pseudonarcissus      ITIGRLVQNFELLPPPGQDKLDTTEKGGQFSLHILKHSTIVAKP.RVF.
AEA72281.1_C4H_Angelica_gigas                 LVIAKMVSNFELSAPQG.TKIDVNEKGGQFSLHIANYSTVLFHPIRTQ.
ADO24190.1_C4H_Allium_sativum                 ITIGRLVQNFDLMPPPGMDKIDTTEKGGQFSLHILNHFTIVAKP.RVF.
ADN32769.1_C4H_Scutellaria_baicalensis        ITIGRLVQNFELLPPPGQAKLDTTEKGGQFSLHIMKHSTIVLKP.RSF.
AIS92509.1_C4H_Epimedium_sagittatum           ITLGRLVQNFELLPPPGQEKLDTTEKGGQFSLHILKHSTIVAKP.RVF.
AGP25594.1_C4H_Aquilaria_sinensis             ITIGRLVQNFELLPPPGCSKLDTTEKGGQFSLHILNHSTIVAKP.RTF.
AAG50231.1_C4H_Populus_trichocarpa_x_Populus  ITLGRLVQNFELLPPPGQSKIDTAEKGGQFSLHILKHSTIVAKP.RSF.
sp|Q43054.1_C4H_Populus_sieboldii_x_Populus_  ITLGRLVQNFELLPPPGQSKIDTSEKGGQFSLHILKHSTIVAKP.RSF.
AAG10197.1_C4H_Gossypium_arboreum             ITLGRLVQNFELLPPKGQSKLDTSEKGGQFSLHILKHSTIVAKP.RVF.
AAG10196.1_C4H_Gossypium_arboreum             ITLGRLVQNFELLPPPGQSQIDTTEKGGQFSLHILKHSTIVAKP.RQF.
sp|Q43033.1_C4H_Petroselinum_crispum          IVIGRLVQNFELLPPPGQSKIDTAEKGGQFSLQILKHSTIVCKP.RSL.
CAC35977.1_C4H_Ruta_graveolens                ITIGRMVQNFELLPPPGQSKIDTSEKGGQFSLFILNHSTIVLKP.RSSV
AAF66066.2_C4H_Citrus_sinensis                ITIGRLVQNFELLPPPGQSKIDTAEKGGQFSLHILKHSTIVAKP.RSF.
AAK57011.1_C4H_Citrus_x_paradisi              ITIGRLVQNFELLPPPGQSKIDTAEKGGQFSLHILKHSTIVAKP.RSF.
sp|P48522.1_C4H_Catharanthus_roseus           ITIGRLVQNFELLPPPGKSKIDTSEKGGQFSLHILKHSTIVLKP.RTF.
BAB71717.1_C4H_Lithospermum_erythrorhizon     ITLGGLVKNFELLPPPGQSKLDTSEKGGQFSLHILKHSTIVMKP.RDL.
BAB71716.1_C4H_Lithospermum_erythrorhizon     ITLGRLVQNFELLPPPGQSKLDTSEKGGQFSLHILKHSTIVMKP.RSL.
AAC35857.1_C4H_Capsicum_chinense              ITLGRLVQNFEMFPPPGQSKLDTSEKGGQFSLHILKHSTIVMKP.RSF.
AAG43824.1_C4H_Capsicum_annuum                ITLGRLVQNFELLPPPGQSKLDTTEKGGQFSLHILKHSTIVMKP.RSF.
sp|Q43240.1_C4H_Zinnia_elegans                ITIGRLVQNFELLPPPGQDKVDTTEKGGQFSLHILKHSTIVAKP.RVL.
sp|Q04468.1_C4H_Helianthus_tuberosus          ITIGRLVQNFELLPPPGQSKIDTDEKGGQFSLHILKHSTIVAKP.RSF.
sp|Q42797.1_C4H_Glycine_max                   ITLGRLVQNFELLPPPGQSQIDTSEKGGQFSLHILKHSTIVAKP.RSF.
sp|P37115.1_C4H_Vigna_radiata                 ITLGRLVQNFELLPPPGQSQIDTSEKGGQFSLHILKHSTVVAKP.RSF.
sp|Q96423.1_C4H_Glycyrrhiza_echinata          ITLGRLVQNFELLPPPGQSKLDTAEKGGQFSLHILKHSTIVAKP.RSF.
sp|O81928.2_C4H_Cicer_arietinum               ITLGRLVQNFELLPPPGQSKIDTAEKGGQFSLHILKHSTIVCKP.RSFN
sp|P37114.1_C4H_Medicago_sativa               ITIGRLVQNVELLPPPGQSKIDTSEKGGQFSLHILKHSTIVAKP.RSF.
sp|Q43067.2_C4H_Pisum_sativum                 ITIGRLVQNFELLPPPGQSKIDTSEKGGQFSLHILKHSTIVAKP.RAF.
AAD11427.1_C4H_Mesembryanthemum_crystallinum  LVIAKLVSNFEMKPPPGEEKIDVSEKGGQFSLHIAKHSTVVFHPIHAA.
sp|O24315_C4H_Phaseolus_vulgaris              LVIAKMVSNFELSAPQGT.KIDVNEKGGQFSLHIANYSTVLFHPIRTQ.
AAK62344.1_C4H_Nicotiana_tabacum              LVIAKLVSNFEMQGPPGVEKVDTSERGGQFSLHIAKHSTVVFKPIAA..
AAK62345.1_C4H_Nicotiana_tabacum              LVIAKLVSNFEMQAPPGVGKVDTSEKGGQFSLHIAKHSTVVFKPIAA..
AAG17469.1_C4H_Triticum_aestivum              ITLGRLVQNFQLLPPPGQDKIDTTEKPGQFTNQILKHATIVCKPLEA..
AAD23378.1_C4H_Pinus_taeda                    LALGRLVQNFELLPPPGQSKVDVTEKGGQFSLHILNHSVVVAKP.IA..
                                         acc
```

```
pdb|5BSR_4CL2a_Nicotiana_tabacum
                                             1                            10        20
pdb|5BSR_4CL2a_Nicotiana_tabacum         ..MEKDTK..................QV...............DII.FRSKLPDIY
TRINITY_DN32164_c5_g1_i2                 ..MLSIASVEPPKPEL.........SSPQT.QNHSS......SSSSETHI.FRSKLPDIP
pdb|3A9U_4CL_Populus_tomentosa           ......MN..................PQE...............EFI.FRSKLPDIY
BAB01715.1_4CL_Arabidopsis_thaliana      ..MVLQQQTHFLTKKIDQED...EEEEPSH...............DFI.FRSKLPDIF
sp|O48869_4CL1_Populus_trichocarpa_x_Popul ..MEAKND..................QAQ...............EFI.FRSKLPDIH
ACF35279.1_4CL_Pinus_radiata             ..MANGIKKV..................................EHL.YRSKLPDIE
CAA36850.1_4CL_Oryza_sativa_Japonica_Group ..MGSMEQQQP...........ESAAPAT.............EASPEII.FRSKLQDIA
BAA08366.2_4CL_Lithospermum_erythrorhizon ..MLSVASPETQKPELSSIAAP.PSSTPQN.QSSISG...DNNSNETII.FRSKLPDIP
AGP02119.1_4CL_Ocimum_basilicum          ..MLSVA..EAQNSELSSH......ALPQT.QT.........SEETADHI.FVSKLPSIS
AIY24982.1_4CL_Mangifera_indica          ..MEVQAK..................LQD...............DLIVFRSKLPDIY
AHY94891.1_4CL_Prunella_vulgaris         ..MLSVASGETQNPDLSSH......ALPQT.IS.........SSTADDHI.FVSKLPTIP
AHA42444.1_4CL_Cannabis_sativa           ..MALVTEIT..............KQEQQQ...............DII.FRSKLPDIY
pdb|3NI2_4CL1__Nicotiana_tabacum         ......MN..................PQE...............EFI.FRSKLPDIY
sp|P17814.2_4CL1_Oryza_sativa            ..MGSMEQQQP...........ESAAPAT.............EASPEII.FRSKLQDIA
sp|Q42982.2_4CL2_Oryza_sativa            ..MITVAAPEA.QPQV...AAAVDEAPPEA........V.........TV.FRSKLPDID
sp|Q6ZAC1.1_4CL5_Oryza_sativa            ..M...................GSLPEQ..................FV.FRSRLPDIA
sp|Q6ETN3.1_4CL3_Oryza_sativa            ..MGSVAA...................................EEVVV.FRSKLPDIE
sp|Q67W82.1_4CL4_Oryza_sativa            ..MGSMAAAA...............EAAQ..............EEETVV.FRSKLPDIE
sp|Q9S725.2_4CL2_Arabidopsis_thaliana    ..MTTQDVIVN...........DQNDQKQ..........CSN...DVI.FRSRLPDIY
sp|Q42524.1_4CL1_Arabidopsis_thaliana    ..MAPQEQAVS...........QVMEKQ...........SNNNNSDVI.FRSKLPDIY
sp|Q9S777.1_4CL3_Arabidopsis_thaliana    ..MITAALHE...PQI.........HKPTD.TSVVSDDVLPHSPPTP.RI.FRSKLPDID
sp|P31684.1_4CL1_Solanum_tuberosum       MPMDTETK..................QSG...............DLI.FRSKLPDIY
sp|P31685.1_4CL2_Solanum_tuberosum       MPMDIETK..................QSG...............DLI.FRSKLPDIY
AAG43823.1_4CL_Capsicum_annuum           MPMENETR....................................DLI.FRSKLPDIY
BAA08365.1_4CL_Lithospermum_erythrorhizon ..MDTQTKT...............DQK...............DII.FRSKLPDIY
BAA08366.2_4CL_Lithospermum_erythrorhizon ..MLSVASPETQKPELSSIAAP.PSSTPQN.QSSISG...DNNSNETII.FRSKLPDIP
sp|P14912.1_4CL1_Petroselinum_crispum    ..MGDCVA..................PKE...............DLI.FRSKLPDIY
sp|P14913.1_4CL2_Petroselinum_crispum    ..MGDCVA..................PKE...............DLI.FRSKLPDIY
AAF91309.1_4CL2_Rubus_idaeus             ..MENKHQ..................DDH...............EFI.FRSKLPDIY
AAF91310.1_4CL1_Rubus_idaeus             ..MAVQT...................PQH...............NIV.YRSKLPDIH
sp|O48868_4CL2_Populus_trichocarpa_x_Popul ..MEANKD..................QVQ...............EFI.FRSKLPDIY
AAK58908.1_4CL3_Populus_trichocarpa_x_Popu ..MDAIMN..................SQE...............EFI.FRSKLPDIY
AAL56850.1_4CL_Populus_tomentosa         ......MN..................PQE...............EFI.FRSKLPDIY
sp|O81139_4CL_Populus_tremuloides        ......MN..................PQ................EFI.FRSKLPDIY
AAL02144.1_4CL_Populus_tomentosa         ......MN..................PQE...............EFI.FRSKLPDIY
AAL35216.1_4CL_Amorpha_fruticosa         ..MAFETE.................EPK...............EFI.FRSKLPEIP
sp|P41636.1_4CL_Pinus_taeda              ..MANGIKKV..................................EHL.YRSKLPDIE
AAF37732.1_4CL1_Lolium_perenne           ..MITVAAPEVQQPQIAAAAAAVEAAAPEA........T.........TI.FRSRLPDID
sp|Q42982.2_4CL2_Oryza_sativa            ..MITVAAPEA.QPQV...AAAVDEAPPEA........V.........TV.FRSKLPDID
sp|P31687.2_4CL2_Glycine_max             ..MITLA......PSL.........DTPKTDQNQVS.....DPQTS.HV.FKSKLPDIP
sp|O81140_4CL_Populus_tremuloides        ..MMSVATVEPPKPEL.........SPPQN.QNAPS.....SHETD.HI.FRSKLPDIT
AAK58909.1_4CL4_Populus_trichocarpa_x_Popu ..MMSVATVEPPKPEL.........SPPQN.QNAPS.....SHETD.HI.FRSKLPDIT
sp|P17814.2_4CL1_Oryza_sativa            ..MGSMEQQQP...........ESAAPAT.............EASPEII.FRSKLQDIA
AAF37733.1_4CL2_Lolium_perenne           ..MGSIAADA...............PPAE..................LV.FRSKLPDIE
AAF37734.1_4CL3_Lolium_perenne           ..MGSVPEESV...........VAVAPAE..................TV.FRSKLPDIE
                                     acc
```

```
                                              α1        η1              β1             β2              α2
pdb|5BSR_4CL2a_Nicotiana_tabacum         TT                                      TTT
                                                30             40           50            60           70
pdb|5BSR_4CL2a_Nicotiana_tabacum         IPNHLPLHSYCFENISEFS....SRPCLINGANKQIYTYADVELNSRKVAAGLHK..QGI
TRINITY_DN32164_c5_g1_i2                 ISNNLPLHTYCFENLLNFS....DRPCLISGSTGKTYTFAETHLISQKVASGLSH..LGI
pdb|3A9U_4CL_Populus_tomentosa           IPKNLPLHSYVLENLSNHS....SKPCLINGANGDVYTYADVELTARRVASGLNK..IGI
BAB01715.1_4CL_Arabidopsis_thaliana      IPNHLPLTDYVFQRFSGDGDGDSSTTCIIDGATGRILTYADVQTNMRRIAAGIHR..LGI
sp|O48869_4CL1_Populus_trichocarpa_x_Popul IPNHLPLHTYCFENLSRFK....DNPCLINGPTGEIHTYAEVELTSRKVASGLNK..LGI
ACF35279.1_4CL_Pinus_radiata             ISDHLPLHSYCFERVAEFA....DRPCLIDGATDRTYCFSEVELISRKVAAGLAK..LGL
CAA36850.1_4CL_Oryza_sativa_Japonica_Group ITNTLPLHRYCFERLPEVA....ARPCLIDGATGGVLTYADVDRLSRRLAAALRRAPLGL
BAA08366.2_4CL_Lithospermum_erythrorhizon ISNNLPLHTYCFQNASEYP....NRTCIIDSKTGKQYTFSETDSICRKVAAGLSN..LGI
AGP02119.1_4CL_Ocimum_basilicum          IPNHLPLHTYCFQNLSQFA....DRQCLISGNDGKSYSFADTHLICRKVAAGLTK..LGI
AIY24982.1_4CL_Mangifera_indica          IPNHLPLHSYCFENIAQVA....SRPCLINGSTGDVYTYSEVEITARKIAVGLNK..LGI
AHY94891.1_4CL_Prunella_vulgaris         ISNDIPLHTYCFQNYSHYP....DRPCLLVGS..KSYSFADTHLMCRRVAAGLSQ..LGI
AHA42444.1_4CL_Cannabis_sativa           IPKHLPLHTYCFGNKTQHD...LSHPCLIYGPTGQVFTYAEVDLTARKLASGLNK..LGV
pdb|3NI2_4CL1__Nicotiana_tabacum         IPKNLPLHSYVLENLSNHS....SKPCLINGANGDVYTYADVELTARRVASGLNK..IGI
sp|P17814.2_4CL1_Oryza_sativa            ITNTLPLHRYCFERLPEVA....ARPCLIDGATGGVLTYADVDRLSRRLAAALRRAPLGL
sp|Q42982.2_4CL2_Oryza_sativa            IPSHLPLHEYCFARAAELP....DAPCLIAAATGRTYTFAETRLLCRRAAAALHR..LGV
sp|Q6ZAC1.1_4CL5_Oryza_sativa            IPDHLPLHDYVFERLADRR....DRACLIDGATGETLSFGDVDALSRRVAAGLSS..IGV
sp|Q6ETN3.1_4CL3_Oryza_sativa            IDNSMTLQEYCFARMAEVG....ARPCLIDGQTGESYTYAEVESASRRAAAGLRR..MGV
sp|Q67W82.1_4CL4_Oryza_sativa            IPSHLTLQAYCFEKLPEVA....ARPCLIDGQTGAVYSYGEVEELSRRAAAGLRR..LGV
sp|Q9S725.2_4CL2_Arabidopsis_thaliana    IPNHLPLHDYIFENISEFA....AKPCLINGPTGEVYTYADVHVTSRKLAAGLHN..LGV
sp|Q42524.1_4CL1_Arabidopsis_thaliana    IPNHLSLHDYIFQNISEFA....TKPCLINGPTGHVYTYSDVHVISRQIAANFHK..LGV
sp|Q9S777.1_4CL3_Arabidopsis_thaliana    IPNHLPLHTYCFEKLSSVS....DKPCLIVGSTGKSYTYGETHLICRRVASGLYK..LGI
sp|P31684.1_4CL1_Solanum_tuberosum       IPKHLPLHSYCFENLSEFN....SRPCLIDGANDRIYTYAEVELTSRKVAVGLNK..LGI
sp|P31685.1_4CL2_Solanum_tuberosum       IPKHLPLHSYCFENLSEFN....SRPCLIDGANDRIYTYAEVELTSRKVAVGLNK..LGI
AAG43823.1_4CL_Capsicum_annuum           IPKHLPLHSYCFENISEFN....SRPCLIDGANDQIYSYAEVELTSRKVAVGLNK..LGV
BAA08365.1_4CL_Lithospermum_erythrorhizon IPKHLPLHSYCGENISQFS....SRPCLINGSNDRVYTYAEVEITSRKVAAGLHK..HGI
BAA08366.2_4CL_Lithospermum_erythrorhizon ISNNLPLHTYCFQNASEYP....NRTCIIDSKTGKQYTFSETDSICRKVAAGLSN..LGI
sp|P14912.1_4CL1_Petroselinum_crispum    IPKHLPLHTYCFENISKVG....DKSCLINGATGETFTYSQVELLSRKVASGLNK..LGI
sp|P14913.1_4CL2_Petroselinum_crispum    IPKHLPLHTYCFENISKVG....DKSCLINGATGETFTYSQVELLSRKVASGLNK..LGI
AAF91309.1_4CL2_Rubus_idaeus             IPNHLPLHTYCFENISQFH....DRPCLINGNTGETFTYAEVELTSRRVAAGLDK..LGI
AAF91310.1_4CL1_Rubus_idaeus             IPNHLPLHSYIFQNKSHLT....SKPCIINGTTGDIHTYAKFKLTARKVASGLNK..LGI
sp|O48868_4CL2_Populus_trichocarpa_x_Popul IPNHLPLHTYCFEKLSQFK....DNPCLINGPTGDIYTYADVELTSRKVASGLYK..LGL
AAK58908.1_4CL3_Populus_trichocarpa_x_Popu IPKNLPLHSYVLENLSKYS....SKPCLINGANGDVYTYADVELTARRVASGLNK..IGI
AAL56850.1_4CL_Populus_tomentosa         IPKNLPLHSYVLENLSKHS....SKPCLINGANGDVYTCADVELTARRVASGLNK..IGI
sp|O81139_4CL_Populus_tremuloides        IPKNLPLHSYVLENLSKHS....SKPCLINGANGDVYTYADVELTARRVASGLNK..IGI
AAL02144.1_4CL_Populus_tomentosa         IPKNLPLHSYVLENLSNHS....SKPCLINGANGDVYTYADVELTARRVASGLNK..IGI
AAL35216.1_4CL_Amorpha_fruticosa         ISKHLPLHSYCFENLSEFG....SRPCLISAPTGDVYTYYDVELTARRVASGLNK..LGV
sp|P41636.1_4CL_Pinus_taeda              ISDHLPLHSYCFERVAEFA....DRPCLIDGATDRTYCFSEVELISRKVAAGLAK..LGL
AAF37732.1_4CL1_Lolium_perenne           IPTHMPLHDYCFATAASAP....DAPCLITAATGKTYTFAETHLLCRKAAAALHG..LGV
sp|Q42982.2_4CL2_Oryza_sativa            IPSHLPLHEYCFARAAELP....DAPCLIAAATGRTYTFAETRLLCRRAAAALHR..LGV
sp|P31687.2_4CL2_Glycine_max             ISNHLPLHSYCFQNLSQFA....HRPCLIVGPASKTFTYADTHLISSKIAAGLSN..LGI
sp|O81140_4CL_Populus_tremuloides        ISNDLPLHAYCFENLSDFS....DRPCLISGSTGKTYSFAETHLISRKVAAGLSN..LGI
AAK58909.1_4CL4_Populus_trichocarpa_x_Popu ISNHLPLHAYCFENLSDFS....DRPCLISGSTGKTYSFAETHLISRKVAAGLSN..LGI
sp|P17814.2_4CL1_Oryza_sativa            ITNTLPLHRYCFERLPEVA....ARPCLIDGATGGVLTYADVDRLSRRLAAALRRAPLGL
AAF37733.1_4CL2_Lolium_perenne           IPTHLTLQDYCFQRLPELS....ARACLIDGATGAALTYGEVDALSRRCAAGLRR..LGV
AAF37734.1_4CL3_Lolium_perenne           INNEQTLQSYCFEKMAEVA....SRPCIIDGQTGASYTYTEVDSLTRRAAAGLRR..MGV
                                     acc
```

```
                                                 β3          α3            β4          α4         β5   η2
pdb|5BSR_4CL2a_Nicotiana_tabacum            TT          ℓℓℓℓℓℓℓℓℓℓℓℓ        TT  ℓℓℓℓℓℓℓℓℓℓℓ       ℓℓℓ
                                                 80        90        100       110       120       130

pdb|5BSR_4CL2a_Nicotiana_tabacum            QPKDTIMILLPNSPEFVFAFIGASYLGAISTMANPLFTPAEVVKQAKASSAKIIVTQACH
TRINITY_DN32164_c5_g1_i2                    KKGDVIMILLQNCPEFVFSFMGASMIGAITTTANPFYTKNEIFKQLTSSRAKLIITQSLY
pdb|3A9U_4CL_Populus_tomentosa              QQGDVIMLFLPSSPEFVLAFLGASHRGAIITAANPFSTPAELAKHAKASRAKLLITQACY
BAB01715.1_4CL_Arabidopsis_thaliana         RHGDVVMLLLPNSPEFALSFLAVAYLGAVSTTANPFYTQPEIAKQAKASAAKMIITKKCL
sp|O48869_4CL1_Populus_trichocarpa_x_Popul  KQGDVILLLLQNSPEFVFAFLGASIIGAISTTANPFYTPAEVAKQATASKAKLIITQAVY
ACF35279.1_4CL_Pinus_radiata                QQGQVVMLLLPNCIEFAFVFMGASVRGAIVTTANPFYKPGEIAKQAKAAGARIIVTLAAY
CAA36850.1_4CL_Oryza_sativa_Japonica_Group  RRGGVVMSLLRNSPEFVLSFFAASRVGAAVTTANPMSTPHEIESQLAAAGATVVITESMA
BAA08366.2_4CL_Lithospermum_erythrorhizon   QKGDVIMVLLQNCAEFVFTFMGASIIGAVITTGNPFYTTAEIFKQVNVSNTKLIITQSNY
AGP02119.1_4CL_Ocimum_basilicum             KKGDVIMVLLQNCAEFVFSFIGASMIGAVTTTANPFCTSKEIFKQFNASRSKLIVTQSQY
AIY24982.1_4CL_Mangifera_indica             QQYDVILLLLPNFPEFILSFLGASYRGAITAANPFYTPAEIAKQADASNAKLIITQACY
AHY94891.1_4CL_Prunella_vulgaris            KKGDVVMALLQNSAEFVFTFMGASMIGAVTTTANPFCTNKEIYKQFHASKSKLIVTQSIY
AHA42444.1_4CL_Cannabis_sativa              KQGDVVMVLLPNSPEYVFTFLGASYRGAMTTAANPFFTAAEIQKQAKASKTKLIVTQASY
pdb|3NI2_4CL1__Nicotiana_tabacum            QQGDVIMLFLPSSPEFVLAFLGASHRGAIITAANPFSTPAELAKHAKASRAKLLITQACY
sp|P17814.2_4CL1_Oryza_sativa               RRGGVVMSLLRNSPEFVLSFFAASRVGAAVTTANPMSTPHEIESQLAAAGATVVITESMA
sp|Q42982.2_4CL2_Oryza_sativa               GHGDRVMVLLQNCVEFAVAFFAASFLGAVTTAANPFCTPQEIHKQFKASGVKLILTQSVY
sp|Q6ZAC1.1_4CL5_Oryza_sativa               CHGSTVMLLLPNSVEFAVAFLASSRLGAVTTTANPLHTPPEIAKQVAASGATVVVTEPAF
sp|Q6ETN3.1_4CL3_Oryza_sativa               GKGDVVMSLLRNCPEFAFSFLGAARLGAATTTANPFYTPHEVHRQAEAAGARVIVTEACA
sp|Q67W82.1_4CL4_Oryza_sativa               GKGDVVMSLLRNCPEFAFTFLGAARLGAATTTANPFYTPHEIHRQASAAGARVIVTEACA
sp|Q9S725.2_4CL2_Arabidopsis_thaliana       KQHDVVMILLPNSPEVVLTFLAASFIGAITTSANPFFTPAEISKQAKASAAKLIVTQSRY
sp|Q42524.1_4CL1_Arabidopsis_thaliana       NQNDVVMLLLPNCPEFVLSFLAASFRGATATAANPFFTPAEIAKQAKASNTKLIITEARY
sp|Q9S777.1_4CL3_Arabidopsis_thaliana       RKGDVIMILLQNSAEFVFSFMGASMIGAVSTTANPFYTSQELYKQLKSSGAKLIITHSQY
sp|P31684.1_4CL1_Solanum_tuberosum          QQKDTIMILLPNCPEFVFAFIGASYLGAISTMANPLFTPAEVVKQAKASSAKIVITQACF
sp|P31685.1_4CL2_Solanum_tuberosum          QQKDTIMILLPNCPEFVFAFIGASYLGAISTMANPLFTPAEVVKQAKASSAKIVITQACF
AAG43823.1_4CL_Capsicum_annuum              QQKDTIMILLPNSPEFVFAFMGASYLGAISTMANPLFTPAEVLKQAKASSAKIIITLACY
BAA08365.1_4CL_Lithospermum_erythrorhizon   KQTETIMLLLPNCPEFVFAFLGASYIGAVSTTANPFFTSSEIIKQAKASKTKLIITVSTT
BAA08366.2_4CL_Lithospermum_erythrorhizon   QKGDVIMVLLQNCAEFVFTFMGASIIGAVITTGNPFYTTAEIFKQVNVSNTKLIITQSNY
sp|P14912.1_4CL1_Petroselinum_crispum       QQGDTIMLLLPNSPEYFFAFLGASYRGAISTMANPFFTSAEVIKQLKASQAKLIITQACY
sp|P14913.1_4CL2_Petroselinum_crispum       QQGDTIMLLLPNSPEYFFAFLGASYRGAISTMANPFFTSAEVIKQLKASLAKLIITQACY
AAF91309.1_4CL2_Rubus_idaeus                QQNDVVMLLLQNCPEFAFAFLGASYIGAMSTTANPFYTPAEVAKQAKASNAKLIITQSAY
AAF91310.1_4CL1_Rubus_idaeus                EKGDVFMLLLPNTSEFVFAFLGASFCGAMMTAANPFFTPAEIAKQAKASKAKLIITFACY
sp|O48868_4CL2_Populus_trichocarpa_x_Popul  QQGDVILLLLQNSPEFVFAFLGASFIGAISSTANPFYTSAEIAKQATASKAKLIITHAAY
AAK58908.1_4CL3_Populus_trichocarpa_x_Popu  QQGDVIMLFLPSSPEFVLAFLGASHRGAIVTAANPFSTPAELAKHAKPPRTKLLITQACY
AAL56850.1_4CL_Populus_tomentosa            QQGDVIMLFLPSSPEFVLAFLGASHRGAITTAANPFSTPAELAKHAKASRAKLLITQACY
sp|O81139_4CL_Populus_tremuloides           QQGDVIMLFLPSSPEFVLAFLGASHRGAMITAANPFSTPAELAKHAKASRAKLLITQACY
AAL02144.1_4CL_Populus_tomentosa            QQGDVIMLFLPSSPEFVLAFLGASHRGAIITAANPFSTPAELAKHAKASRAKLLITQACY
AAL35216.1_4CL_Amorpha_fruticosa            QQGDVIMLLLPNSPEFVFSFLGASYRGAMITAANPFFTSAEIAKQAKASNTKLIITQASY
sp|P41636.1_4CL_Pinus_taeda                 QQGQVVMLLLPNCIEFAFVFMGASVRGAIVTTANPFYKPGEIAKQAKAAGARIIVTLAAY
AAF37732.1_4CL1_Lolium_perenne              RHGDRIMLLLQNSVEFALAFFGASMLGAVSTAANPFCTPQEIHKQLVASGAKLVVTQSAY
sp|Q42982.2_4CL2_Oryza_sativa               GHGDRVMVLLQNCVEFAVAFFAASFLGAVTTAANPFCTPQEIHKQFKASGVKLILTQSVY
sp|P31687.2_4CL2_Glycine_max                LKGDVVMILLQNSADFVFSFLAISMIGAVATTANPFYTAPEIFKQFTVSKAKLIITQAMY
sp|O81140_4CL_Populus_tremuloides           KKGDVIMTLLQNCPEFVFSFIGASMIGAVITTANPFYTQSEIFKQFSASRAKLIITQSQY
AAK58909.1_4CL4_Populus_trichocarpa_x_Popu  KKGDVIMTLLQNCPEFVFSFMGASMIGAVTTTVNPFYTPGEIFKQFSASRAKLIITQSQH
sp|P17814.2_4CL1_Oryza_sativa               RRGGVVMSLLRNSPEFVLSFFAASRVGAAVTTANPMSTPHEIESQLAAAGATVVITESMA
AAF37733.1_4CL2_Lolium_perenne              GKGDVVMALLRNCPEFAFVFLGAARLGAATTTANPFYTPHEIHRQATAAGARVIVTEACA
AAF37734.1_4CL3_Lolium_perenne              GKGDVVMNLLRNCPEFAFSFLGAARLGAATTTANPFYTPHEIHRQAEAAGAKLIVTEACA
                                       acc

                                                     α5                 β6             β7    α6       η3
pdb|5BSR_4CL2a_Nicotiana_tabacum            ℓℓℓ ℓℓℓℓ..........ℓℓ                  T.T       ℓℓℓ.ℓ     ℓℓ
                                               140               150                 160          170

pdb|5BSR_4CL2a_Nicotiana_tabacum            VNKVKDYA..........FEND.VKIICID.......SAPE.GCLHF.SVL.TQ.ANEH
TRINITY_DN32164_c5_g1_i2                    VEKLKDS...........EEN.LILITIDDP.......PE.NCLHF.SVL.SE.SNET
pdb|3A9U_4CL_Populus_tomentosa              YEKVKDFA..........RESD.VKVMCVD.......SAPD.GCLHF.SEL.TQ.ADEN
BAB01715.1_4CL_Arabidopsis_thaliana         VDKLTNLK............NDGVLIVCLDDDGDNGVVSSSDDGCVSF.TEL.TQ.ADET
sp|O48869_4CL1_Populus_trichocarpa_x_Popul  AEKVQEFV..........KENVHVKIVTVD.......SPPE.NYLHF.SEL.TN.SDED
ACF35279.1_4CL_Pinus_radiata                VEKLADLQ..........SHD..VLVITID.......DAPKEGCQHI.SVL.TE.ADET
CAA36850.1_4CL_Oryza_sativa_Japonica_Group  ADKLPSHSHGA............LTVVLID.......ERRD.GCLHFWDDL.MS.EDEA
BAA08366.2_4CL_Lithospermum_erythrorhizon   VDKLRNTTINESDNKYPKLGED.FKVITIDTP........PE.NCLPF.SLL.IENTQEN
AGP02119.1_4CL_Ocimum_basilicum             VDKLRDTG.....DDSVVFGED.FSVVTIDAP........PE.GCLDF.SVL.CE.ADEA
AIY24982.1_4CL_Mangifera_indica             VDKVKALA..........KKRD.VKIMCID.......SAPD.GCLHF.SEL.TQ.ADEN
AHY94891.1_4CL_Prunella_vulgaris            VDKLRDTG.....DDSLVLGVD.FPVVTVDSP........PQ.GCLPF.SAL.SD.ADPS
AHA42444.1_4CL_Cannabis_sativa              YDKVKDLD..........QEGL.VKFVCVDS......PVPE.GWFHF.SELCNN.SDEN
pdb|3NI2_4CL1__Nicotiana_tabacum            YEKVKDFA..........RESD.VKVMCVD.......SAPD.GCLHF.SEL.TQ.ADEN
sp|P17814.2_4CL1_Oryza_sativa               ADKLPSHSHGA............LTVVLID.......ERRD.GCLHFWDDL.MS.EDEA
sp|Q42982.2_4CL2_Oryza_sativa               VDKLRQHEAFPRIDACTVGDDT.LTVITIDDDE.....ATPE.GCLPF.WDL.IADADEG
sp|Q6ZAC1.1_4CL5_Oryza_sativa               VAKVSGLAG..............VTVVATG.......GGAE.RCASF.AGL.AA.ADGS
sp|Q6ETN3.1_4CL3_Oryza_sativa               VEKVREFA..........AERG.VPVVTVD.......GAFD.GCVEFREVL...AAEEL
sp|Q67W82.1_4CL4_Oryza_sativa               VEKVRGFA..........ADRG.IPVVAVD.......GDFD.GCVGFGEAMLDASIEPL
sp|Q9S725.2_4CL2_Arabidopsis_thaliana       VDKIKNLQ..........N.DG.VLIVTTDSD.....AIPE.NCLRF.SEL.TQ.SEEP
sp|Q42524.1_4CL1_Arabidopsis_thaliana       VDKIKPLQ..........NDDG.VVIVCIDDNES...VPIPE.GCLRF.TEL.TQ.STTE
sp|Q9S777.1_4CL3_Arabidopsis_thaliana       VDKLKN............LGEN.LTLITTDEP.......TPE.NCLPF.STL.IT.DDET
sp|P31684.1_4CL1_Solanum_tuberosum          AGKVKDYA..........IEND.LKVICVD.......SVPE.GCVHF.SEL.IQ.SDEH
sp|P31685.1_4CL2_Solanum_tuberosum          AGKVKDYA..........IEND.LKVICVD.......SAPE.GCVHF.SEL.IQ.SDEH
AAG43823.1_4CL_Capsicum_annuum              IGKVKDYA..........TEND.VKLICID.......SAPE.GCIHF.SEL.TQ.SDEH
BAA08365.1_4CL_Lithospermum_erythrorhizon   VPKLKDFS..........QENH.VKIMCID.......DKID.GCLHFSSDL.EN.SDET
BAA08366.2_4CL_Lithospermum_erythrorhizon   VDKLRNTTINESDNKYPKLGED.FKVITIDTP........PE.NCLPF.SLL.IENTQEN
sp|P14912.1_4CL1_Petroselinum_crispum       VDKVKDYA..........AEKN.IQIICID.......DAPQ.DCLHF.SKL.ME.ADES
sp|P14913.1_4CL2_Petroselinum_crispum       VDKVKDYA..........AEKN.IQIICID.......DAPQ.DCLHF.SKL.ME.ADES
AAF91309.1_4CL2_Rubus_idaeus                VDKVKDFA..........KLND.VKVMCVDE......TSSE.DVLHF.SEL.MS.ADES
AAF91310.1_4CL1_Rubus_idaeus                YDKVKDLS..........CDE..VKLMCIDS......PPPDSSCLHF.SEL.TQ.SDEN
sp|O48868_4CL2_Populus_trichocarpa_x_Popul  AEKVQQFA..........QENDHVKIMTID.......SLTE.NCLHF.SEL.TS.SDEN
AAK58908.1_4CL3_Populus_trichocarpa_x_Popu  YDKVKDFA..........RESD.VKVMCVD.......SAPD.GCLHF.SEL.TQ.ADEN
AAL56850.1_4CL_Populus_tomentosa            YEKVKDFA..........RESD.VKVMCVD.......SAPD.GCLHF.SEL.TQ.ADEN
sp|O81139_4CL_Populus_tremuloides           YEKVKDFA..........RESD.VKVMCVD.......SAPD.GASLF.RAH.TQ.ADEN
AAL02144.1_4CL_Populus_tomentosa            YEKVKDFA..........RESD.VKVMCVD.......SAPD.GCLHF.SEL.TQ.ADEN
AAL35216.1_4CL_Amorpha_fruticosa            YDKVKDL.............D.VKLVFVD.......SPPD.GHMHY.SEL.RE.ADES
sp|P41636.1_4CL_Pinus_taeda                 VEKLADLQ..........SHD..VLVITID.......DAPKEGCQHI.SVL.TE.ADET
AAF37732.1_4CL1_Lolium_perenne              VDKLR.HEAFPRI......GEA.LTVITIDEDD.....GTPD.GCQPF.WAL.VSAADEN
sp|Q42982.2_4CL2_Oryza_sativa               VDKLRQHEAFPRIDACTVGDDT.LTVITIDDDE.....ATPE.GCLPF.WDL.IADADEG
sp|P31687.2_4CL2_Glycine_max                VDKLRNHD.......GAKLGED.FKVVTVDDP........PE.NCLHF.SVL.SE.ANES
sp|O81140_4CL_Populus_tremuloides           VNKLGDSDCHE...NNQKPGED.FIVITIDDP........PE.NCLHF.NVL.VE.ASES
AAK58909.1_4CL4_Populus_trichocarpa_x_Popu  VNKLRDSDYHE...NNQKPEED.FIVITIDDP........PE.NCLHFWDDL.MS.EDEA
sp|P17814.2_4CL1_Oryza_sativa               ADKLPSHSHGA............LTVVLID.......ERRD.GCLHFWDDL.MS.EDEA
AAF37733.1_4CL2_Lolium_perenne              VEKVRAFA..........AERG.IPVVSVD.......EGVDGGCLPFAETLLGEESGER
AAF37734.1_4CL3_Lolium_perenne              VEKVLEFA..........AGRG.VPVVTVD.......GRRD.GCVDFAELIAGEELPEA
                                       acc
```

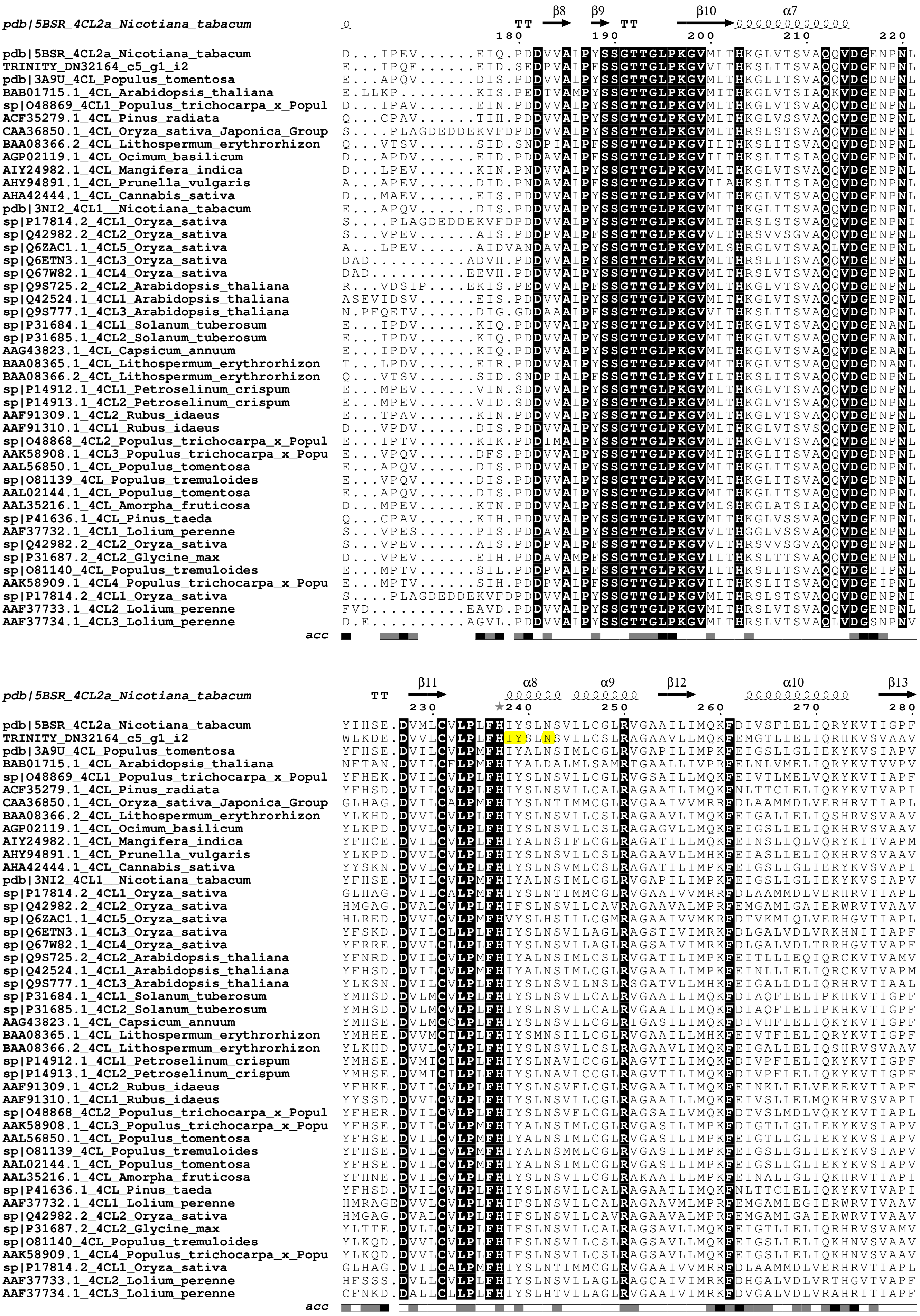
pdb|5BSR_4CL2a_Nicotiana_tabacum
TT β8 β9 TT β10 α7
180 190 200 210 220
acc
pdb|5BSR_4CL2a_Nicotiana_tabacum
TT β11 α8 α9 β12 α10 β13
230 240 250 260 270 280
acc

```
                                            α11         η4              β14          α12            β15  β16  η5
pdb|5BSR_4CL2a_Nicotiana_tabacum                                  TT
                                                    290       300       310       320       330       340
pdb|5BSR_4CL2a_Nicotiana_tabacum           VPPIVLAIAKSPMVDDYDLSSVRTVMSGAAPLGKELEDTVRAKFPNAKLGQGYGMTEAGP
TRINITY_DN32164_c5_g1_i2                   VPPLVLALAKNPMVAGFDLTSIRVVLSGAAPLGKELEDALRSRVPQATLGQGYGMTEAGP
pdb|3A9U_4CL_Populus_tomentosa             VPPVMMSIAKSPDLDKHDLSSLRMIKSGGAPLGKELEDTVRAKFPQARLGQGYGMTEAGP
BAB01715.1_4CL_Arabidopsis_thaliana        APPVVLAFIKSPETERYDLSSVRIMLSGAATLKKELEDAVRLKFPNAIFGQGYGMTESGT
sp|O48869_4CL1_Populus_trichocarpa_x_Popul VPPVVLAVAKCPVVDKYDLSSIRTVMSGAAPMGKELEDTVRAKLPNAKLGQGYGMTEAGP
ACF35279.1_4CL_Pinus_radiata               VPPIVLDITKSPIVSQYDVSSVRIIMSGAAPLGKELEDALRERFPKAIFGQGYGMTEAGP
CAA36850.1_4CL_Oryza_sativa_Japonica_Group VPPIVVAVAKSEAAAARDLSSVRMVLSGAAPMGKDIEDAFMAKLPGAVLGQGYGMTEAGP
BAA08366.2_4CL_Lithospermum_erythrorhizon  VPPLVLALAKNPMVDKYDLSSIRVVLSGAAPLGRELELALLNRVPHAIFGQGYGMTEAGP
AGP02119.1_4CL_Ocimum_basilicum            VPPLVLALAKNPLVDSFDLSSIRLVLSGAAPLGKELEAALLSRLPQAVFGQGYGMTEAGP
AIY24982.1_4CL_Mangifera_indica            VPPIVLAISKSPDVDKYDLSSIRIIKSGAAPLGRELEDSVRAKFPNATLGQGYGMTEAGP
AHY94891.1_4CL_Prunella_vulgaris           VPPLVLALAKNPLVDNFDLSSIRMVLSGAAPLGKELEAALLCRLPQAVFGQGYGMTEAGP
AHA42444.1_4CL_Cannabis_sativa             VPPIVLAIAKYPDLHKYDLSSLKVLKSGGAPLGKELEDTVRAKFPNVTLGQGYGMTEAGP
pdb|3NI2_4CL1__Nicotiana_tabacum           VPPVMMSIAKSPDLDKHDLSSLRMIKSGGAPLGKELEDTVRAKFPQARLGQGYGMTEAGP
sp|P17814.2_4CL1_Oryza_sativa              VPPIVVAVAKSEAAAARDLSSVRMVLSGAAPMGKDIEDAFMAKLPGAVLGQGYGMTEAGP
sp|Q42982.2_4CL2_Oryza_sativa              VPPLVLALAKNPFVERHDLSSIRIVLSGAAPLGKELEDALRARLPQAIFGQGYGMTEAGP
sp|Q6ZAC1.1_4CL5_Oryza_sativa              VPPIVVEMAKSDALDRHDLSSIRMVISGAAPMGKELQDIVHAKLPNAVLGQGYGMTEAGP
sp|Q6ETN3.1_4CL3_Oryza_sativa              VPPIVVEIAKSPRVTAEDLASIRMVMSGAAPMGKDLQDAFMAKIPNAVLGQGYGMTEAGP
sp|Q67W82.1_4CL4_Oryza_sativa              VPPIVVEIAKSPRVTADDLASIRMVMSGAAPMGKDLQDAFMAKIPNAVLGQGYGMTEAGP
sp|Q9S725.2_4CL2_Arabidopsis_thaliana      VPPIVLAIAKSPETEKYDLSSVRMVKSGAAPLGKELEDAISAKFPNAKLGQGYGMTEAGP
sp|Q42524.1_4CL1_Arabidopsis_thaliana      VPPIVLAIAKSSETEKYDLSSIRVVKSGAAPLGKELEDAVNAKFPNAKLGQGYGMTEAGP
sp|Q9S777.1_4CL3_Arabidopsis_thaliana      VPPLVIALAKNPTVNSYDLSSVRFVLSGAAPLGKELQDSLRRRLPQAILGQGYGMTEAGP
sp|P31684.1_4CL1_Solanum_tuberosum         VPPIVLAIAKSPLVDNYDLSSVRTVMSGAAPLGKELEDAVRAKFPNAKLGQGYGMTEAGP
sp|P31685.1_4CL2_Solanum_tuberosum         VPPIVLAIAKSPLVHNYDLSSVRTVMSGAAPLGKELEDAVRAKFPNAKLGQGYGMTEAGP
AAG43823.1_4CL_Capsicum_annuum             VPPIVLAIAKSPLVDHYYLSSVRTVMSGAAPLGKELEDTVRTKFPNAKLGQGYGMTEAGP
BAA08365.1_4CL_Lithospermum_erythrorhizon  VPPIVLAIAKSNVVDQYDLSTVRTVMSGAAPLGSELEDAVRAKFPNAKLGQGYGMTEAGP
BAA08366.2_4CL_Lithospermum_erythrorhizon  VPPLVLALAKNPMVDKYDLSSIRVVLSGAAPLGRELELALLNRVPHAIFGQGYGMTEAGP
sp|P14912.1_4CL1_Petroselinum_crispum      VPPIVLAIAKSPVVDKYDLSSVRTVMSGAAPLGKELEDAVRAKFPNAKLGQGYGMTEAGP
sp|P14913.1_4CL2_Petroselinum_crispum      VPPIVLAIAKSPVVDKYDLSSVRTVMSGAAPLGKELEDAVRAKFPNAKLGQGYGMTEAGP
AAF91309.1_4CL2_Rubus_idaeus               VPPIVLSIAKCPDLHRYDLSSIRMVMSGAAPMGKELEDTVRAKLPNAKLGQGYGMTEAGP
AAF91310.1_4CL1_Rubus_idaeus               VPPTVLAIAKFPDLDKYDLGSIRVLKSGGAPLGKELEDTVRAKFPNVTLGQGYGMTEAGP
sp|O48868_4CL2_Populus_trichocarpa_x_Popul VPPICLAIAKSPVVDQYDLSSIRTVLSGAAPLGKELEDTVRAKLPNAKLGQGYGMTEAGP
AAK58908.1_4CL3_Populus_trichocarpa_x_Popu VPPVMLAIAKSPDFDKHDLSSLRMIKSGGAPLGKELEDTVRAKFPQARLGQGYGMTEAGP
AAL56850.1_4CL_Populus_tomentosa           VPPVMLAIARSPDLDKHDLSSLRMIKSGGAPLGKELEDTVRAKFPQARLGQGYGMTEAGP
sp|O81139_4CL_Populus_tremuloides          VPPVMMAIAKSPDLDKHDLSSLRMIKSGGAPLGKELEDTVRAKFPQARLGQGYGMTEAGP
AAL02144.1_4CL_Populus_tomentosa           VPPVMMSIAKSPDLDKHDLSSLRMIKSGGAPLGKELEDTVRAKFPQARLGQGYGMTEAGP
AAL35216.1_4CL_Amorpha_fruticosa           VPPIVLAIAKSPDLEKYDLSSIRVLKSGGASLGKELEDTVRAKFPKAKLGQGYGMTEAGP
sp|P41636.1_4CL_Pinus_taeda                VPPIVLDITKSPIVSQYDVSSVRIIMSGAAPLGKELEDALRERFPKAIFGQGYGMTEAGP
AAF37732.1_4CL1_Lolium_perenne             VPPLVLALAKNPGVEKHDLSSIRIVLSGAAPLGKELEDALRGRLPQAIFGQGYGMTEAGP
sp|Q42982.2_4CL2_Oryza_sativa              VPPLVLALAKNPFVERHDLSSIRIVLSGAAPLGKELEDALRARLPQAIFGQGYGMTEAGP
sp|P31687.2_4CL2_Glycine_max               VPPLVLALAKNPMVADFDLSSIRLVLSGAAPLGKELEEALRNRMPQAVLGQGYGMTEAGP
sp|O81140_4CL_Populus_tremuloides          VPPLVLALAKNPLEANFDLSSIRVVLSGAAPLGKELEDALRSRVPQAILGQGYGMTEAGP
AAK58909.1_4CL4_Populus_trichocarpa_x_Popu VPPLVLALAKNPMVANFDLSSIRVVLSGAAPLGKELEEALRSRVPQAILGQGYGMTEAGP
sp|P17814.2_4CL1_Oryza_sativa              VPPIVVAVAKSEAAAARDLSSVRMVLSGAAPMGKDIEDAFMAKLPGAVLGQGYGMTEAGP
AAF37733.1_4CL2_Lolium_perenne             VPPIVVEIAKSARVTAADLASIRLVMSGAAPMGKELQDAFMAKIPNAVLGQGYGMTEAGP
AAF37734.1_4CL3_Lolium_perenne             VPPIVVEIAKSDRVGADDLASIRMVLSGAAPMGKDLQDAFMAKIPNAVLGQGYGMTEAGP
                                       acc
```

```
                                           β17  η6                    β18   β19                  β20   β21
pdb|5BSR_4CL2a_Nicotiana_tabacum                          TT                            TT            TT
                                                    350       360       370       380       390       400
pdb|5BSR_4CL2a_Nicotiana_tabacum           VLAMCLAFAKEPFEIKSGACGTVVRNAEMKIVDPKTGNSLPRNQSGEICIRGDQIMKGYL
TRINITY_DN32164_c5_g1_i2                   VLSMCLGFAKHPIPTKSGSCGTVVRNAELKVIHPETGSSLGYNQPGEICIRGQQIMKGYL
pdb|3A9U_4CL_Populus_tomentosa             VLAMCLAFAKEPFDIKPGACGTVVRNAEMKIVDPETGASLPRNQPGEICIRGDQIMKGYL
BAB01715.1_4CL_Arabidopsis_thaliana        V.AKSLAFAKNPFKTKSGACGTVIRNAEMKVVDTETGISLPRNKSGEICVRGHQLMKGYL
sp|O48869_4CL1_Populus_trichocarpa_x_Popul VLSMCLAFAKEPFEIKSGACGTVVRNAEMKIVDPDTGRSLPRNQSGEICIRGSQIMKGYL
ACF35279.1_4CL_Pinus_radiata               VLAMNLAFAKNPFPVKSGSCGTVVRNAQIKILDTETGESLPHHQAGEICIRGPEIMKGYI
CAA36850.1_4CL_Oryza_sativa_Japonica_Group VLSMCLAFAKEPFKVKSGACGTVVRNAELKIIDPDTGKSLGRNLRGEICIRGQQIMKGYL
BAA08366.2_4CL_Lithospermum_erythrorhizon  VLSMSPSFAKHPYPAKSGSCGTVVRNADLKVIDPETGSSLGRNQPGEICIRGEQIMKGYL
AGP02119.1_4CL_Ocimum_basilicum            VLSMSPLFAKQPLPTKSGSCGNVVRNAELKVIDPETGSSLRRNQPGEICIRGPQIMKGYL
AIY24982.1_4CL_Mangifera_indica            VLAMGLAFAKQPFPIKPGACGTVVRNAEMKIVDPDTGVSLPRNQPGEICIRGDQIMKGYL
AHY94891.1_4CL_Prunella_vulgaris           VLSMSPSFAKQPLPTKSGSCGNVVRNAELKVVDPETGCSLPRNQPGEICIRGPQIMKGYL
AHA42444.1_4CL_Cannabis_sativa             VLTMSLAFAKEAFDVKAGACGTVVRNAEMKIVDPETGSSLPRNQPGEICIRGDQIMKGYL
pdb|3NI2_4CL1__Nicotiana_tabacum           VLAMCLAFAKEPFDIKPGACGTVVRNAEMKIVDPETGASLPRNQPGEICIRGDQIMKGYL
sp|P17814.2_4CL1_Oryza_sativa              VLSMCLAFAKEPFKVKSGACGTVVRNAELKIIDPDTGKSLGRNLPGEICIRGQQIMKGYL
sp|Q42982.2_4CL2_Oryza_sativa              VLSMCPAFAKEPTPAKSGSCGTVVRNAELKVVDPDTGFSLGRNLPGEICIRGPQIMKGYL
sp|Q6ZAC1.1_4CL5_Oryza_sativa              VLSMCMAFAKEPTPVKSGACGTVVRNAELKIVDPDTGLSLPRNQPGEICIRGKQIMKGYL
sp|Q6ETN3.1_4CL3_Oryza_sativa              VLAMCLAFAKEPFKVKSGSCGTVVRNAELKIVDPDTGTSLGRNQSGEICIRGEQIMKGYL
sp|Q67W82.1_4CL4_Oryza_sativa              VLAMCLAFAKEPFEVKSGSCGTVVRNAELKIVDPDTGATLGRNQSGEICIRGEQIMKGYL
sp|Q9S725.2_4CL2_Arabidopsis_thaliana      VLAMSLGFAKEPFPVKSGACGTVVRNAEMKILDPDTGDSLPRNKPGEICIRGNQIMKGYL
sp|Q42524.1_4CL1_Arabidopsis_thaliana      VLAMSLGFAKEPFPVKSGACGTVVRNAEMKIVDPDTGDSLSRNQPGEICIRGHQIMKGYL
sp|Q9S777.1_4CL3_Arabidopsis_thaliana      VLSMSLGFAKEPIPTKSGSCGTVVRNAELKVVHLETRLSLGYNQPGEICIRGQQIMKEYL
sp|P31684.1_4CL1_Solanum_tuberosum         VLAMCLAFAKEPFDIKSGACGTVVRNAEMKIVDPDTGCSLPRNQPGEICIRGDQIMKGYL
sp|P31685.1_4CL2_Solanum_tuberosum         VLAMCLAFAKEPFDIKSGACGTVVRNAEMKIVDPDTGCSLPRNQPGEICIRGDQIMKGYL
AAG43823.1_4CL_Capsicum_annuum             VLAMCLAFAKEPFEIKSRACGTGARNAEMKNVDPDTGCSLPRNQPGENCIRGDQIMKGYL
BAA08365.1_4CL_Lithospermum_erythrorhizon  VLAMCLAFAKEPFEIKSGACGTVVRNSEMKIIDTETGASLPRNQSGEICIRGDQIMKGYL
BAA08366.2_4CL_Lithospermum_erythrorhizon  VLSMSPSFAKHPYPAKSGSCGTVVRNADLKVIDPETGSSLGRNQPGEICIRGEQIMKGYL
sp|P14912.1_4CL1_Petroselinum_crispum      VLAMCLAFAKEPYEIKSGACGTVVRNAEMKIVDPETNASLPRNQRGEICIRGDQIMKGYL
sp|P14913.1_4CL2_Petroselinum_crispum      VLAMCLAFAKEPYEIKSGACGTVVRNAEMKIVDPETNASLPRNQRGEICIRGDQIMKGYL
AAF91309.1_4CL2_Rubus_idaeus               VLSMCLAFAKEPYEIKSGACGTVVRNAEMKIIDPDTNESLPRNQSGEICIRGSQIMKGYL
AAF91310.1_4CL1_Rubus_idaeus               VLTMSLAFAKEPFEVKPGGCGTVVRNAELKIVDPETGASLPRNHPGEICIRGHQIMKGYL
sp|O48868_4CL2_Populus_trichocarpa_x_Popul VIAMCLAFAKEPFEIKSGACGTVVRNAEMKIVDPETGESQPRNKTGEICIRGCQIMKGYL
AAK58908.1_4CL3_Populus_trichocarpa_x_Popu VLAMCLAFAKEPFDIKPGACGTVVRNAEMKIVDPETGASLRRNQPGEICIRGDQIMKGYL
AAL56850.1_4CL_Populus_tomentosa           VLAMCLAFAKEPFDIKPGACGTVVRNAEMKIVDPETGASLPRNQPGEICIRGDQIMKGYL
sp|O81139_4CL_Populus_tremuloides          VLAMCLAFAKEPFDIKPGACGTVVRNAEMKIVDPETGVSLPRNQPGEICIRGDQIMKGYL
AAL02144.1_4CL_Populus_tomentosa           VLAMCLAFAKEPFDIKPGACGTVVRNAEMKIVDPETGASLPRNQPGEICIRGDQIMKGYL
AAL35216.1_4CL_Amorpha_fruticosa           VLTMCLAFAKEPIDVKPGACGTVVRNAEMKIVDPETGNSLPRNQSGEICIRGDQIMKGYL
sp|P41636.1_4CL_Pinus_taeda                VLAMNLAFAKNPFPVKSGSCGTVVRNAQIKILDTETGESLPHNQAGEICIRGPEIMKGYI
AAF37732.1_4CL1_Lolium_perenne             VLSMCPAFAREPTPAKSGSCGTVVRNAQLKVVDPDTGVSLGRNLPGEICIRGPQIMKGYL
sp|Q42982.2_4CL2_Oryza_sativa              VLSMCPAFAKEPTPAKSGSCGTVVRNAELKVVDPDTGFSLGRNLPGEICIRGPQIMKGYL
sp|P31687.2_4CL2_Glycine_max               VLSMCLGFAKQPFQTKSGSCGTVVRNAELKVVDPETGRSLGYNQPGEICIRGQQIMKGYL
sp|O81140_4CL_Populus_tremuloides          VLSMCLAFSKQPFPTKSGSCGTVVRNAELKVIDPETGRSLGYNQPGEICIRGSQIMKGYL
AAK58909.1_4CL4_Populus_trichocarpa_x_Popu VLSMCLAFSKQPLPTKSGSCGTVVRNAELKVIDPETGSSLGRNQPGEICIRGSQIMKGYL
sp|P17814.2_4CL1_Oryza_sativa              VLSMCLAFAKEPFKVKSGACGTVVRNAELKIIDPDTGKSLGRNLPGEICIRGQQIMKGYL
AAF37733.1_4CL2_Lolium_perenne             VLAMCLAFAKEPFAVKSGSCGTVVRNAELKIVDPDTGASLGRNLPGEICIRGKQIMKGYL
AAF37734.1_4CL3_Lolium_perenne             VLAMCLAFAKEPFKVKSGSCGTVVRNAELKVVDPDTGASLGRNQPGEICVRGKQIMIGYL
                                       acc
```

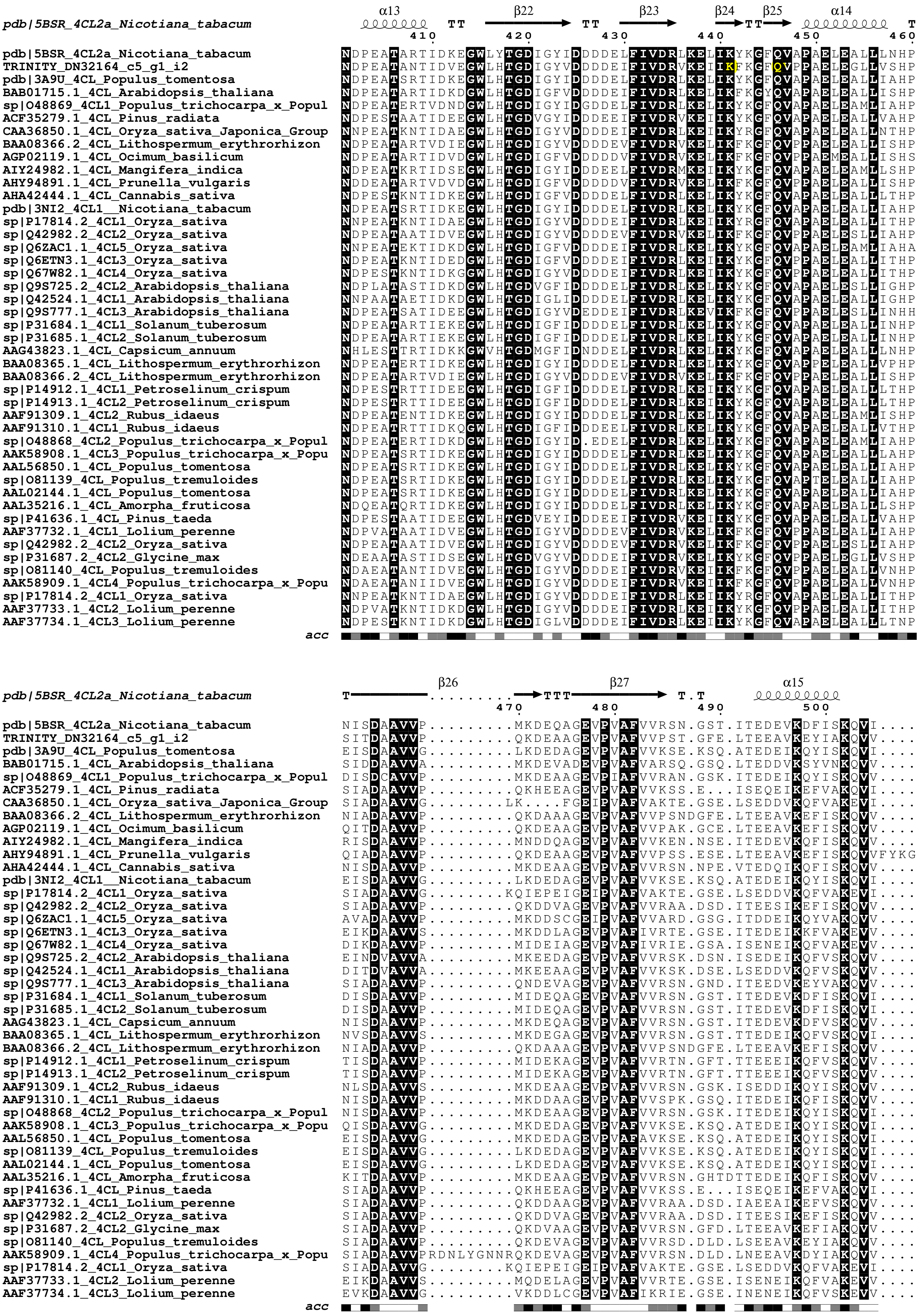
pdb|5BSR_4CL2a_Nicotiana_tabacum
α13
β22
β23
β24
β25
α14
410
420
430
440
450
460
acc
β26
β27
α15
470
480
490
500

```
pdb|5BSR_4CL2a_Nicotiana_tabacum

pdb|5BSR_4CL2a_Nicotiana_tabacum                 ............................................................
TRINITY_DN32164_c5_g1_i2                         ............................................................
pdb|3A9U_4CL_Populus_tomentosa                   ............................................................
BAB01715.1_4CL_Arabidopsis_thaliana              ............................................................
sp|O48869_4CL1_Populus_trichocarpa_x_Popul       ............................................................
ACF35279.1_4CL_Pinus_radiata                     ............................................................
CAA36850.1_4CL_Oryza_sativa_Japonica_Group       ............................................................
BAA08366.2_4CL_Lithospermum_erythrorhizon        ............................................................
AGP02119.1_4CL_Ocimum_basilicum                  ............................................................
AIY24982.1_4CL_Mangifera_indica                  ............................................................
AHY94891.1_4CL_Prunella_vulgaris                 MPKGFFVQAIPKSPFGKILGKNFRAQIGSPTFLFIVDRLKELIKYKGYQVAPAELEALLL
AHA42444.1_4CL_Cannabis_sativa                   ............................................................
pdb|3NI2_4CL1__Nicotiana_tabacum                 ............................................................
sp|P17814.2_4CL1_Oryza_sativa                    ............................................................
sp|Q42982.2_4CL2_Oryza_sativa                    ............................................................
sp|Q6ZAC1.1_4CL5_Oryza_sativa                    ............................................................
sp|Q6ETN3.1_4CL3_Oryza_sativa                    ............................................................
sp|Q67W82.1_4CL4_Oryza_sativa                    ............................................................
sp|Q9S725.2_4CL2_Arabidopsis_thaliana            ............................................................
sp|Q42524.1_4CL1_Arabidopsis_thaliana            ............................................................
sp|Q9S777.1_4CL3_Arabidopsis_thaliana            ............................................................
sp|P31684.1_4CL1_Solanum_tuberosum               ............................................................
sp|P31685.1_4CL2_Solanum_tuberosum               ............................................................
AAG43823.1_4CL_Capsicum_annuum                   ............................................................
BAA08365.1_4CL_Lithospermum_erythrorhizon        ............................................................
BAA08366.2_4CL_Lithospermum_erythrorhizon        ............................................................
sp|P14912.1_4CL1_Petroselinum_crispum            ............................................................
sp|P14913.1_4CL2_Petroselinum_crispum            ............................................................
AAF91309.1_4CL2_Rubus_idaeus                     ............................................................
AAF91310.1_4CL1_Rubus_idaeus                     ............................................................
sp|O48868_4CL2_Populus_trichocarpa_x_Popul       ............................................................
AAK58908.1_4CL3_Populus_trichocarpa_x_Popu       ............................................................
AAL56850.1_4CL_Populus_tomentosa                 ............................................................
sp|O81139_4CL_Populus_tremuloides                ............................................................
AAL02144.1_4CL_Populus_tomentosa                 ............................................................
AAL35216.1_4CL_Amorpha_fruticosa                 ............................................................
sp|P41636.1_4CL_Pinus_taeda                      ............................................................
AAF37732.1_4CL1_Lolium_perenne                   ............................................................
sp|Q42982.2_4CL2_Oryza_sativa                    ............................................................
sp|P31687.2_4CL2_Glycine_max                     ............................................................
sp|O81140_4CL_Populus_tremuloides                ............................................................
AAK58909.1_4CL4_Populus_trichocarpa_x_Popu       ............................................................
sp|P17814.2_4CL1_Oryza_sativa                    ............................................................
AAF37733.1_4CL2_Lolium_perenne                   ............................................................
AAF37734.1_4CL3_Lolium_perenne                   ............................................................
                                             acc

                                                                                                η7      β28
pdb|5BSR_4CL2a_Nicotiana_tabacum                                                                  510

pdb|5BSR_4CL2a_Nicotiana_tabacum                 ................................................FYKRIKRVFFVD
TRINITY_DN32164_c5_g1_i2                         ................................................FYKKLHKVYFVH
pdb|3A9U_4CL_Populus_tomentosa                   ................................................FYKRIKRVFFIE
BAB01715.1_4CL_Arabidopsis_thaliana              ................................................HYKRIKMVFFIE
sp|O48869_4CL1_Populus_trichocarpa_x_Popul       ................................................FYKRISRVFFTE
ACF35279.1_4CL_Pinus_radiata                     ................................................FYKKIHRVYFVD
CAA36850.1_4CL_Oryza_sativa_Japonica_Group       ................................................YYKKIREVFFVD
BAA08366.2_4CL_Lithospermum_erythrorhizon        ................................................FYKRLHKVYFVH
AGP02119.1_4CL_Ocimum_basilicum                  ................................................FYKRLHKVYFVH
AIY24982.1_4CL_Mangifera_indica                  ................................................FYKRINRVFFID
AHY94891.1_4CL_Prunella_vulgaris                 NHPNISDAAVVPMKDEQAGEVPVAFVVKSKGSVITEDDIKAFVSKQVIFYKRIKRVFFVD
AHA42444.1_4CL_Cannabis_sativa                   ................................................FYKRISRVFFID
pdb|3NI2_4CL1__Nicotiana_tabacum                 ................................................FYKRIKRVFFIE
sp|P17814.2_4CL1_Oryza_sativa                    ................................................YYKKIREVFFVD
sp|Q42982.2_4CL2_Oryza_sativa                    ................................................FYKRLHKVHFIH
sp|Q6ZAC1.1_4CL5_Oryza_sativa                    ................................................FYKRLHKIFFVD
sp|Q6ETN3.1_4CL3_Oryza_sativa                    ................................................FYKRINKVFFTD
sp|Q67W82.1_4CL4_Oryza_sativa                    ................................................FYKRLNKVFFAD
sp|Q9S725.2_4CL2_Arabidopsis_thaliana            ................................................FYKRINKVFFTD
sp|Q42524.1_4CL1_Arabidopsis_thaliana            ................................................FYKRINKVFFTE
sp|Q9S777.1_4CL3_Arabidopsis_thaliana            ................................................FYKRLHKVFFVA
sp|P31684.1_4CL1_Solanum_tuberosum               ................................................FYKRIKRVFFVE
sp|P31685.1_4CL2_Solanum_tuberosum               ................................................FYKRIKRVFFVE
AAG43823.1_4CL_Capsicum_annuum                   ................................................FYKRIKRVFFVE
BAA08365.1_4CL_Lithospermum_erythrorhizon        ................................................FYKRINRVFGVD
BAA08366.2_4CL_Lithospermum_erythrorhizon        ................................................FYKRLHKVYFVH
sp|P14912.1_4CL1_Petroselinum_crispum            ................................................FYKRIFRVFFVD
sp|P14913.1_4CL2_Petroselinum_crispum            ................................................FYKRIFRVFFVD
AAF91309.1_4CL2_Rubus_idaeus                     ................................................FYKRISKVFFTD
AAF91310.1_4CL1_Rubus_idaeus                     ................................................FYKRIKRVFFIE
sp|O48868_4CL2_Populus_trichocarpa_x_Popul       ................................................FYKRIGRVFFTE
AAK58908.1_4CL3_Populus_trichocarpa_x_Popu       ................................................FYKRIKRVFFIE
AAL56850.1_4CL_Populus_tomentosa                 ................................................FYKRIKRVFFIE
sp|O81139_4CL_Populus_tremuloides                ................................................FYKRIKRVFFIE
AAL02144.1_4CL_Populus_tomentosa                 ................................................FYKRIKRVFFIE
AAL35216.1_4CL_Amorpha_fruticosa                 ................................................FYKRISRVFFID
sp|P41636.1_4CL_Pinus_taeda                      ................................................FYKKIHRVYFVD
AAF37732.1_4CL1_Lolium_perenne                   ................................................FYKRLHKVYFTH
sp|Q42982.2_4CL2_Oryza_sativa                    ................................................FYKRLHKVHFIH
sp|P31687.2_4CL2_Glycine_max                     ................................................FYKRLHKVYFVH
sp|O81140_4CL_Populus_tremuloides                ................................................FYKKLHKVFFVH
AAK58909.1_4CL4_Populus_trichocarpa_x_Popu       ................................................FYKKLHKVFFVH
sp|P17814.2_4CL1_Oryza_sativa                    ................................................YYKKIREVFFVD
AAF37733.1_4CL2_Lolium_perenne                   ................................................FYKRICKVFFAD
AAF37734.1_4CL3_Lolium_perenne                   ................................................FYKRINKVYFTD
                                             acc
```

```
pdb|5BSR_4CL2a_Nicotiana_tabacum                                  α16
                                             TT     ℓℓℓℓℓℓℓ
                                    520             530
                                      .               .
pdb|5BSR_4CL2a_Nicotiana_tabacum               AIPKSPSGKILRKDLRAKLA.....A..................................
TRINITY_DN32164_c5_g1_i2                       AIPKSPSGKILRKDLKAKLA.....S..................................
pdb|3A9U_4CL_Populus_tomentosa                 AIPKAPSGKILRKNLKEKL......A..................................
BAB01715.1_4CL_Arabidopsis_thaliana            VIPKAVSGKILRKDLRAKLE.....T..................................
sp|O48869_4CL1_Populus_trichocarpa_x_Popul     AIPKAPSGKILRKDLRARLA.....T..................................
ACF35279.1_4CL_Pinus_radiata                   AIPKSPSGKILRKDLRSRLA.....AK.................................
CAA36850.1_4CL_Oryza_sativa_Japonica_Group     KIPKAPSGKILRKELRKQLQHLQQEA..................................
BAA08366.2_4CL_Lithospermum_erythrorhizon      SIPKSPSGKILRKDLRAKLA.....A..................................
AGP02119.1_4CL_Ocimum_basilicum                AIPKSPSGKILRKDLRAKLA.....A..................................
AIY24982.1_4CL_Mangifera_indica                AIPKAPSGKILRKDLRAKLA.....A..................................
AHY94891.1_4CL_Prunella_vulgaris               AVPKSPSGKILRKELRARLAD...GV..................................
AHA42444.1_4CL_Cannabis_sativa                 AIPKSPSGKILRKDLRAKLA.....L..................................
pdb|3NI2_4CL1__Nicotiana_tabacum               AIPKAPSGKILRKNLKEKL......A..................................
sp|P17814.2_4CL1_Oryza_sativa                  KIPKAPSGKILRKELRKQLQHLQQEA..................................
sp|Q42982.2_4CL2_Oryza_sativa                  AIPKSASGKILRRELRAKLA.....A..................................
sp|Q6ZAC1.1_4CL5_Oryza_sativa                  AIPKAPSGKILRKDLRAKLA.....A..................................
sp|Q6ETN3.1_4CL3_Oryza_sativa                  SIPKNPSGKILRKDLRARLA.....A..................................
sp|Q67W82.1_4CL4_Oryza_sativa                  SIPKSPSGKILRKDLRAKLA.....A..................................
sp|Q9S725.2_4CL2_Arabidopsis_thaliana          SIPKAPSGKILRKDLRARLA.....N..................................
sp|Q42524.1_4CL1_Arabidopsis_thaliana          SIPKAPSGKILRKDLRAKLA.....N..................................
sp|Q9S777.1_4CL3_Arabidopsis_thaliana          SIPKSPSGKILRKDLKAKLC........................................
sp|P31684.1_4CL1_Solanum_tuberosum             TVPKSPSGKILRKDLRARLA.....A..................................
sp|P31685.1_4CL2_Solanum_tuberosum             TVPKSPSGKILRKDLRARLA.....A..................................
AAG43823.1_4CL_Capsicum_annuum                 TVPKSPSGKILRKDLRARLA.....A..................................
BAA08365.1_4CL_Lithospermum_erythrorhizon      SIPKSPSGKIVRKDLRAKLA.....ARFLNGPTTNVVPNGGNVAKDNVPNGVSNGVSKAN
BAA08366.2_4CL_Lithospermum_erythrorhizon      SIPKSPSGKILRKDLRAKLA.....A..................................
sp|P14912.1_4CL1_Petroselinum_crispum          AIPKSPSGKILRKDLRARIA.....S..................................
sp|P14913.1_4CL2_Petroselinum_crispum          AIPKSPSGKILRKDLRAKIA.....S..................................
AAF91309.1_4CL2_Rubus_idaeus                   KIPKAPSGKILRKDLRARLA.....A..................................
AAF91310.1_4CL1_Rubus_idaeus                   AIPKSPSGKILRKELRAKLA.....A..................................
sp|O48868_4CL2_Populus_trichocarpa_x_Popul     AIPKAPSGKILRKDLRARVS.....A..................................
AAK58908.1_4CL3_Populus_trichocarpa_x_Popu     AIPKAPSGKILRKNLRETL......P..................................
AAL56850.1_4CL_Populus_tomentosa               AIPKAPSGKILRKNLKEKL......P..................................
sp|O81139_4CL_Populus_tremuloides              AIPKAPSGKILRKNLKEKL......P..................................
AAL02144.1_4CL_Populus_tomentosa               AIPKAPSGKILRKNLKEKL......A..................................
AAL35216.1_4CL_Amorpha_fruticosa               AIPKSPSGKILRKDLRAKLA.....A..................................
sp|P41636.1_4CL_Pinus_taeda                    AIPKSPSGKILRKDLRSRLA.....AK.................................
AAF37732.1_4CL1_Lolium_perenne                 AIPKSASGKILRKELRAKLA.....A..................................
sp|Q42982.2_4CL2_Oryza_sativa                  AIPKSASGKILRRELRAKLA.....A..................................
sp|P31687.2_4CL2_Glycine_max                   AIPKSPSGKILRKDLRAKLE.....T..................................
sp|O81140_4CL_Populus_tremuloides              SIPKSASGKILRKDLRAKLA.....T..................................
AAK58909.1_4CL4_Populus_trichocarpa_x_Popu     SIPKSASGKILRKDLRAKLA.....T..................................
sp|P17814.2_4CL1_Oryza_sativa                  KIPKAPSGKILRKELRKQLQHLQQEA..................................
AAF37733.1_4CL2_Lolium_perenne                 SIPKSPSGKILRKDLRAKLA.....A..................................
AAF37734.1_4CL3_Lolium_perenne                 SIPKNPSGKILRKDLRARLA.....A..................................
                                          acc


pdb|5BSR_4CL2a_Nicotiana_tabacum
                                                                          540
                                                                            .
pdb|5BSR_4CL2a_Nicotiana_tabacum               ...........................G...LPN..........................
TRINITY_DN32164_c5_g1_i2                       ..........................ASQ..LS...........................
pdb|3A9U_4CL_Populus_tomentosa                 ...........................G...I............................
BAB01715.1_4CL_Arabidopsis_thaliana            ...............................MCSK.........................
sp|O48869_4CL1_Populus_trichocarpa_x_Popul     ...........................GDFLIKF..........................
ACF35279.1_4CL_Pinus_radiata                   ............................................................
CAA36850.1_4CL_Oryza_sativa_Japonica_Group     ...............................LTN..........................
BAA08366.2_4CL_Lithospermum_erythrorhizon      ..........................AAS..S............................
AGP02119.1_4CL_Ocimum_basilicum                ..........................PSS..TS...........................
AIY24982.1_4CL_Mangifera_indica                ...........................G...LPN..........................
AHY94891.1_4CL_Prunella_vulgaris               ..........................PA................................
AHA42444.1_4CL_Cannabis_sativa                 ...........................GN..L............................
pdb|3NI2_4CL1__Nicotiana_tabacum               ...........................G...I............................
sp|P17814.2_4CL1_Oryza_sativa                  ............................................................
sp|Q42982.2_4CL2_Oryza_sativa                  ..........................C.................................
sp|Q6ZAC1.1_4CL5_Oryza_sativa                  ...........................G...IPAC.........................
sp|Q6ETN3.1_4CL3_Oryza_sativa                  ...........................G...IP..DAVAAAAA................D
sp|Q67W82.1_4CL4_Oryza_sativa                  ...........................G...IPTNDNTQLKS..................
sp|Q9S725.2_4CL2_Arabidopsis_thaliana          ...........................G...LMN..........................
sp|Q42524.1_4CL1_Arabidopsis_thaliana          ...........................G...L............................
sp|Q9S777.1_4CL3_Arabidopsis_thaliana          ............................................................
sp|P31684.1_4CL1_Solanum_tuberosum             ...........................G...ISN..........................
sp|P31685.1_4CL2_Solanum_tuberosum             ...........................G...ISN..........................
AAG43823.1_4CL_Capsicum_annuum                 ...........................G...VTN..........................
BAA08365.1_4CL_Lithospermum_erythrorhizon      GGVAKEGVANGVPTDGDYGVATKGVANG...ISNGVYKQVSNGVVSNGVANGIVSNGIAN
BAA08366.2_4CL_Lithospermum_erythrorhizon      ..........................AAS..S............................
sp|P14912.1_4CL1_Petroselinum_crispum          ..........................GD...LPK..........................
sp|P14913.1_4CL2_Petroselinum_crispum          ..........................GD...LPK..........................
AAF91309.1_4CL2_Rubus_idaeus                   ...........................G...LPN..........................
AAF91310.1_4CL1_Rubus_idaeus                   ...........................G...FAN..........................
sp|O48868_4CL2_Populus_trichocarpa_x_Popul     ...........................GD..LPC..........................
AAK58908.1_4CL3_Populus_trichocarpa_x_Popu     ...........................G...I............................
AAL56850.1_4CL_Populus_tomentosa               ...........................G...I............................
sp|O81139_4CL_Populus_tremuloides              ...........................G...I............................
AAL02144.1_4CL_Populus_tomentosa               ...........................G...I............................
AAL35216.1_4CL_Amorpha_fruticosa               ...........................G...VPN..........................
sp|P41636.1_4CL_Pinus_taeda                    ............................................................
AAF37732.1_4CL1_Lolium_perenne                 ..........................PAT..A............................
sp|Q42982.2_4CL2_Oryza_sativa                  ..........................C.................................
sp|P31687.2_4CL2_Glycine_max                   ..........................AAT..QTP..........................
sp|O81140_4CL_Populus_tremuloides              ..........................ATT..MS...........................
AAK58909.1_4CL4_Populus_trichocarpa_x_Popu     ..........................ATT..MS...........................
sp|P17814.2_4CL1_Oryza_sativa                  ............................................................
AAF37733.1_4CL2_Lolium_perenne                 ...........................G...IPSSNTTQSKS..................
AAF37734.1_4CL3_Lolium_perenne                 ...........................G...IP....TEVAA..................
                                          acc
```

pdb|5BSR_4CL2a_Nicotiana_tabacum

```
pdb|5BSR_4CL2a_Nicotiana_tabacum            ...........
TRINITY_DN32164_c5_g1_i2                    ...........
pdb|3A9U_4CL_Populus_tomentosa              ...........
BAB01715.1_4CL_Arabidopsis_thaliana         ...........
sp|O48869_4CL1_Populus_trichocarpa_x_Popul  .QHDTYMQKQQ
ACF35279.1_4CL_Pinus_radiata                ...........
CAA36850.1_4CL_Oryza_sativa_Japonica_Group  ...........
BAA08366.2_4CL_Lithospermum_erythrorhizon   ...........
AGP02119.1_4CL_Ocimum_basilicum             ...........
AIY24982.1_4CL_Mangifera_indica             ...........
AHY94891.1_4CL_Prunella_vulgaris            ...........
AHA42444.1_4CL_Cannabis_sativa              ...........
pdb|3NI2_4CL1__Nicotiana_tabacum            ...........
sp|P17814.2_4CL1_Oryza_sativa               ...........
sp|Q42982.2_4CL2_Oryza_sativa               ...........
sp|Q6ZAC1.1_4CL5_Oryza_sativa               ...........
sp|Q6ETN3.1_4CL3_Oryza_sativa               APKSS......
sp|Q67W82.1_4CL4_Oryza_sativa               ...........
sp|Q9S725.2_4CL2_Arabidopsis_thaliana       ...........
sp|Q42524.1_4CL1_Arabidopsis_thaliana       ...........
sp|Q9S777.1_4CL3_Arabidopsis_thaliana       ...........
sp|P31684.1_4CL1_Solanum_tuberosum          ...........
sp|P31685.1_4CL2_Solanum_tuberosum          ...........
AAG43823.1_4CL_Capsicum_annuum              ...........
BAA08365.1_4CL_Lithospermum_erythrorhizon   GVHN.......
BAA08366.2_4CL_Lithospermum_erythrorhizon   ...........
sp|P14912.1_4CL1_Petroselinum_crispum       ...........
sp|P14913.1_4CL2_Petroselinum_crispum       ...........
AAF91309.1_4CL2_Rubus_idaeus                ...........
AAF91310.1_4CL1_Rubus_idaeus                ...........
sp|O48868_4CL2_Populus_trichocarpa_x_Popul  .TSDS......
AAK58908.1_4CL3_Populus_trichocarpa_x_Popu  ...........
AAL56850.1_4CL_Populus_tomentosa            ...........
sp|O81139_4CL_Populus_tremuloides           ...........
AAL02144.1_4CL_Populus_tomentosa            ...........
AAL35216.1_4CL_Amorpha_fruticosa            ...........
sp|P41636.1_4CL_Pinus_taeda                 ...........
AAF37732.1_4CL1_Lolium_perenne              ...........
sp|Q42982.2_4CL2_Oryza_sativa               ...........
sp|P31687.2_4CL2_Glycine_max                ...........
sp|O81140_4CL_Populus_tremuloides           ...........
AAK58909.1_4CL4_Populus_trichocarpa_x_Popu  ...........
sp|P17814.2_4CL1_Oryza_sativa               ...........
AAF37733.1_4CL2_Lolium_perenne              ...........
AAF37734.1_4CL3_Lolium_perenne              .PRS.......
                                       acc
```

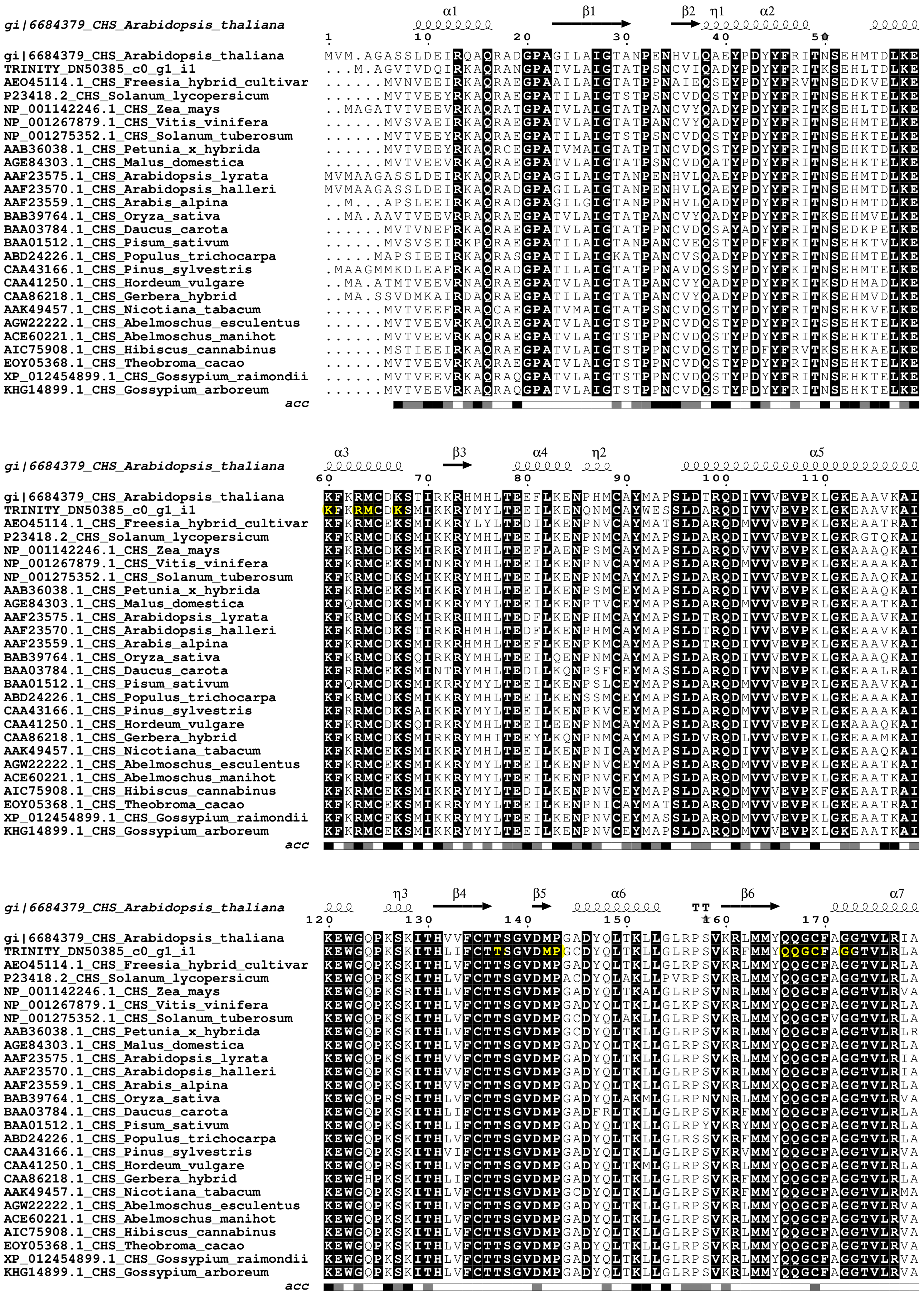
gi|6684379_CHS_Arabidopsis_thaliana
α1 β1 β2 η1 α2
1 10 20 30 40 50
gi|6684379_CHS_Arabidopsis_thaliana MVM.AGASSLDEIRQAQRADGPAGILAIGTANPENHVLQAEYPDYYFRITNSEHMTDLKE
TRINITY_DN50385_c0_g1_i1 ...M.AGVTVDQIRKAQRANGPATVLAIGTATPSNCVIQADYPDYYFRITKSEHLTDLKE
AEO45114.1_CHS_Freesia_hybrid_cultivar ......MVNVEEIRKAQRAEGPAAILAIGTATPPNAIEQSEYPDYYFRVTNSEDKVELKE
P23418.2_CHS_Solanum_lycopersicum ......MVTVEEYRKAQRAEGPATILAIGTSTPSNCVDQSTYPDYYFRITNSEHKTELKE
NP_001142246.1_CHS_Zea_mays ..MAGATVTVEEVRKAQRATGPATVLAIGTATPANCVYQADYPDYYFRITKSEHLTDLKE
NP_001267879.1_CHS_Vitis_vinifera ......MVSVAEIRKAQRAEGPATVLAIGTATPANCVYQADYPDYYFRITNSEHMTELKE
NP_001275352.1_CHS_Solanum_tuberosum ......MVTVEEYRKAQRAEGPATILAIGTSTPSNCVDQSTYPDYYFRITNSEHKTELKE
AAB36038.1_CHS_Petunia_x_hybrida ......MVTVEEYRKAQRCEGPATVMAIGTATPTNCVDQSTYPDYYFRITNSEHKTDLKE
AGE84303.1_CHS_Malus_domestica ......MVTVEEVRKAQRAEGPATVLAIGTATPSNCVDQATYPDYYFRITNSEHKTELKE
AAF23575.1_CHS_Arabidopsis_lyrata MVMAAGASSLDEIRKAQRADGPAGILAIGTANPENHVLQAEYPDYYFRITNSEHMTDLKE
AAF23570.1_CHS_Arabidopsis_halleri MVMAAGASSLDEIRKAQRADGPAGILAIGTANPENHVLQAEYPDYYFRITNSEHMTDLKE
AAF23559.1_CHS_Arabis_alpina ..M...APSLEEIRKAQRADGPAGILGIGTANPPNHVLQADYPDYYFRITNSDHMTDLKE
BAB39764.1_CHS_Oryza_sativa ..MA.AAVTVEEVRRAQRAEGPATVLAIGTATPANCVYQADYPDYYFRITKSEHMVELKE
BAA03784.1_CHS_Daucus_carota ......MVTVNEFRKAQRAEGPATVLAIGTATPPNCVDQSAYADYYFRITNSEDKPELKE
BAA01512.1_CHS_Pisum_sativum ......MVSVSEIRKPQRAEGPATILAIGTANPANCVEQSTYPDFYFKITNSEHKTVLKE
ABD24226.1_CHS_Populus_trichocarpa .....MAPSIEEIRKAQRASGPATILAIGKATPANCVSQADYPDYYFRITNSEHMTELKE
CAA43166.1_CHS_Pinus_sylvestris .MAAGMMKDLEAFRKAQRADGPATILAIGTATPPNAVDQSSYPDYYFKITNSEHMTELKE
CAA41250.1_CHS_Hordeum_vulgare ..MA.ATMTVEEVRNAQRAEGPATVLAIGTATPANCVYQADYPDYYFKITKSDHMADLKE
CAA86218.1_CHS_Gerbera_hybrid ..MA.SSVDMKAIRDAQRAEGPATILAIGTATPANCVYQADYPDYYFRITKSEHMVDLKE
AAK49457.1_CHS_Nicotiana_tabacum ......MVTVEEFRRAQCAEGPATVMAIGTATPSNCVDQSTYPDYYFRITNSEHKVELKE
AGW22222.1_CHS_Abelmoschus_esculentus ......MVTVEEVRKAQRAEGPATVLAIGTSTPPNCVDQSTYPDYYFRITSSEHKTELKE
ACE60221.1_CHS_Abelmoschus_manihot ......MVTVEEVRKAQRAEGPATVLAIGTSTPPNCVDQSTYPDYYFRITKSEHKTELKE
AIC75908.1_CHS_Hibiscus_cannabinus ......MSTIEEIRKAQRAEGPATVLAIGTSTPPNCVDQSTYPDFYFRVTKSEHKAELKE
EOY05368.1_CHS_Theobroma_cacao ......MVTVEEVRKAQRAEGPATVLAIGTSTPPNCVDQSTYPDYYFRITNSEHKTELKE
XP_012454899.1_CHS_Gossypium_raimondii ......MVTVEEVRKAQRAQGPATVLAIGTSTPPNCVDQSTYPDYYFRITNSEHKTELKE
KHG14899.1_CHS_Gossypium_arboreum ......MVTVEEVRKAQRAQGPATVLAIGTSTPPNCVDQSTYPDYYFRITNSEHKTELKE
acc
gi|6684379_CHS_Arabidopsis_thaliana
α3 β3 α4 η2 α5
60 70 80 90 100 110
gi|6684379_CHS_Arabidopsis_thaliana KFKRMCDKSTIRKRHMHLTEEFLKENPHMCAYMAPSLDTRQDIVVVEVPKLGKEAAVKAI
TRINITY_DN50385_c0_g1_i1 KFKRMCDKSMIKKRYLYLTEEILKENQNMCAYWESSLDARQDIVVVEVPKLGKEAAVKAI
AEO45114.1_CHS_Freesia_hybrid_cultivar KFKRMCEKSMIKKRYLYLTEDILKENPNVCAYMATSLDARQDMVVVEVPKLGKEAATRAI
P23418.2_CHS_Solanum_lycopersicum KFKRMCEKSMIKKRYMHLTEEILKENPNMCAYMAPSLDARQDIVVVEVPKLGKRGTQKAI
NP_001142246.1_CHS_Zea_mays KFKRMCDKSMIRKRYMHLTEEFLAENPSMCAYMAPSLDARQDVVVVEVPKLGKAAAQKAI
NP_001267879.1_CHS_Vitis_vinifera KFKRMCDKSMINKRYMHLTEEILKENPNVCAYMAPSLDARQDMVVVEVPKLGKEAAAKAI
NP_001275352.1_CHS_Solanum_tuberosum KFKRMCDKSMIKKRYMHLTEEILKENPNMCAYMAPSLDARQDIVVVEVPKLGKEAAQKAI
AAB36038.1_CHS_Petunia_x_hybrida KFKRMCEKSMIKKRYMHLTEEILKENPSMCEYMAPSLDARQDIVVVEVPKLGKEAAQKAI
AGE84303.1_CHS_Malus_domestica KFQRMCDKSMIKKRYMYLTEEILKENPTVCEYMAPSLDARQDMVVVEVPRLGKEAATKAI
AAF23575.1_CHS_Arabidopsis_lyrata KFKRMCDKSMIRKRHMHLTEDFLKENPHMCAYMAPSLDTRQDIVVVEVPKLGKEAAVKAI
AAF23570.1_CHS_Arabidopsis_halleri KFKRMCDKSTIRKRHMHLTEDFLKENPHMCAYMAPSLDTRQDIVVVEVPKLGKEAAVKAI
AAF23559.1_CHS_Arabis_alpina KFKRMCDKSMIRKRHMHLTEEFLKENPKMCAYMAPSLDTRQDIVVVEVPKLGKEAAVKAI
BAB39764.1_CHS_Oryza_sativa KFKRMCDKSQIRKRYMHLTEEILQENPNMCAYMAPSLDARQDIVVVEVPKLGKAAAQKAI
BAA03784.1_CHS_Daucus_carota KFRRMCEKSMINTRYMHLTEDLLKQNPSFCEYMASSLDARQDIVVNEVPKLGKEAALRAI
BAA01512.1_CHS_Pisum_sativum KFQRMCDKSMIKKRYMYLTEEILKENPSLCEYMAPSLDARQDMVVVEVPRLGKEAAVKAI
ABD24226.1_CHS_Populus_trichocarpa KFKRMCDKSMIKKRYMHLTEEILKENSSMCEYMAPSLDARQDMVVVEVPKLGKEAAAKAI
CAA43166.1_CHS_Pinus_sylvestris KFRRMCDKSAIKKRYMYLTEEILKENPKVCEYMAPSLDARQDMVVVEVPRLGKEAAAKAI
CAA41250.1_CHS_Hordeum_vulgare KFKRMCDKSQIRKRYMHLTEEILEENPNMCAYMAPSLDARQDIVVVEVPKLGKAAAQKAI
CAA86218.1_CHS_Gerbera_hybrid KFKRMCDKSMIRKRYMHITEEYLKQNPNMCAYMAPSLDVRQDIVVVEVPKLGKEAAMKAI
AAK49457.1_CHS_Nicotiana_tabacum KFKRMCEKSMIKKRYMHLTEEILKENPNICAYMAPSLDARQDIVVVEVPKLGKEAAQKAI
AGW22222.1_CHS_Abelmoschus_esculentus KFKRMCEKSMIKKRYMYLTEEILKENPNVCEYMAPSLDARQDMVVVEVPKLGKEAATKAI
ACE60221.1_CHS_Abelmoschus_manihot KFKRMCEKSMIKKRYMYLTEEILKENPNVCEYMAPSLDARQDMVVVEVPKLGKEAATKAI
AIC75908.1_CHS_Hibiscus_cannabinus KFKRMCEKSMIKKRYMYLTEDILKENPNVCEYMAPSLDARQDMVVVEVPKFGKEAATRAI
EOY05368.1_CHS_Theobroma_cacao KFKRMCEKSMIKRRYMYLTEEILKENPNICAYMATSLDARQDMVVVEVPKLGKEAATRAI
XP_012454899.1_CHS_Gossypium_raimondii KFKRMCEKSMIKKRYMYLTEEILKENPNVCEYMASSLDARQDMVVVEVPKLGKEAATKAI
KHG14899.1_CHS_Gossypium_arboreum KFKRMCEKSMIKKRYMYLTEEILKENPNVCEYMAPSLDARQDMVVVEVPKLGKEAATKAI
acc
gi|6684379_CHS_Arabidopsis_thaliana
η3 β4 β5 α6 TT β6 α7
120 130 140 150 160 170
gi|6684379_CHS_Arabidopsis_thaliana KEWGQPKSKITHVVFCTTSGVDMPGADYQLTKLLGLRPSVKRLMMYQQGCFAGGTVLRIA
TRINITY_DN50385_c0_g1_i1 KEWGQPKSKITHLIFCTTSGVDMPGCDYQLTKLLGLRPSVKRFMMYQQGCFAGGTVLRLA
AEO45114.1_CHS_Freesia_hybrid_cultivar KEWGQPKSKITHLVFCTTSGVDMPGADYQLTKLLGLRPSVKRLMMYQQGCFAGGTVLRLA
P23418.2_CHS_Solanum_lycopersicum KEWGQPKSKITHLVFCTTSGVDMPACDYQLAKLLPVRPSVKRLMMYQQGCFAGGTVLRLA
NP_001142246.1_CHS_Zea_mays KEWGQPKSRITHLVFCTTSGVDMPGADYQLTKALGLRPSVNRLMMYQQGCFAGGTVLRVA
NP_001267879.1_CHS_Vitis_vinifera KEWGQPKSKITHLVFCTTSGVDMPGADYQLTKLLGLKPSVKRLMMYQQGCFAGGTVLRLA
NP_001275352.1_CHS_Solanum_tuberosum KEWGQPKSKITHLVFCTTSGVDMPGCDYQLAKLLGLRPSVKRLMMYQQGCFVGGTVLRLA
AAB36038.1_CHS_Petunia_x_hybrida KEWGQPKSKITHLVFCTTSGVDMPGCDYQLTKLLGLRPSVKRLMMYQQGCFAGGTVLRLR
AGE84303.1_CHS_Malus_domestica KEWGQPKSKITHLVFCTTSGVDMPGADYQLTKLLGLRPSVKRLMMYQQGCFAGGTVLRLA
AAF23575.1_CHS_Arabidopsis_lyrata KEWGQPKSKITHVVFCTTSGVDMPGADYQLTKLLGLRPSVKRLMMYQQGCFAGGTVLRIA
AAF23570.1_CHS_Arabidopsis_halleri KEWGQPKSKITHVVFCTTSGVDMPGADYQLTKLLGLRPSVKRLMMYQQGCFAGGTVLRIA
AAF23559.1_CHS_Arabis_alpina KEWGQPKSKITHVVFCTTSGVDMPGADYQLTKLLGLRPSVKRLMMXQQGCFAGGTVLRLA
BAB39764.1_CHS_Oryza_sativa KEWGQPRSRITHLVFCTTSGVDMPGADYQLAKMLGLRPNVNRLMMYQQGCFAGGTVLRVA
BAA03784.1_CHS_Daucus_carota KEWGQPKSKITHLIFCTTSGVDMPGADFRLTKLLGLRPSVKRFMMYQQGCFAGGTVLRLA
BAA01512.1_CHS_Pisum_sativum KEWGQPKSKITHLIFCTTSGVDMPGADYQLTKLLGLRPYVKRYMMYQQGCFAGGTVLRLA
ABD24226.1_CHS_Populus_trichocarpa KEWGQPKSKITHLVFCTTSGVDMPGADYQLTKLLGLRSSVKRFMMYQQGCFAGGTVLRLA
CAA43166.1_CHS_Pinus_sylvestris KEWGQPKSKITHVIFCTTSGVDMPGADYQLTKLLGLRPSVKRVMMYQQGCFAGGTVLRVA
CAA41250.1_CHS_Hordeum_vulgare KEWGQPRSKITHLVFCTTSGVDMPGADYQLTKMLGLRPSVKRLMMYQQGCFAGGTVLRLA
CAA86218.1_CHS_Gerbera_hybrid KEWGHPKSKITHLIFCTTSGVDMPGADYQLTKLLGLRPSVKRFMMYQQGCFAGGTVLRLA
AAK49457.1_CHS_Nicotiana_tabacum KEWGQPKSKITHLVFCTTSGVDMPGCDYQLTKLLGLRPSVKRFMMYQQGCFAGGTVLRMA
AGW22222.1_CHS_Abelmoschus_esculentus KEWGQPKSKITHLVFCTTSGVDMPGADYQLTKLLGLRPSVKRLMMYQQGCFAGGTVLRVA
ACE60221.1_CHS_Abelmoschus_manihot KEWGQPKSKITHLVFCTTSGVDMPGADYQLTKLLGLRPSVKRLMMYQQGCFAGGTVLRVA
AIC75908.1_CHS_Hibiscus_cannabinus KEWGQPKSKITHLVFCTTSGVDMPGADYQLTKLLGLRPSVKRLMMYQQGCFAGGTVLRVA
EOY05368.1_CHS_Theobroma_cacao KEWGQPKSKITHLVFCTTSGVDMPGADYQLTKLLGLRPSVKRLMMYQQGCFAGGTVLRVA
XP_012454899.1_CHS_Gossypium_raimondii KEWGQPKSKITHLVFCTTSGVDMPGCDYQLTKLLGLRPSVKRLMMYQQGCFAGGTVLRVA
KHG14899.1_CHS_Gossypium_arboreum KEWGQPKSKITHLVFCTTSGVDMPGADYQLTKLLGLRPSVKRLMMYQQGCFAGGTVLRVA
acc

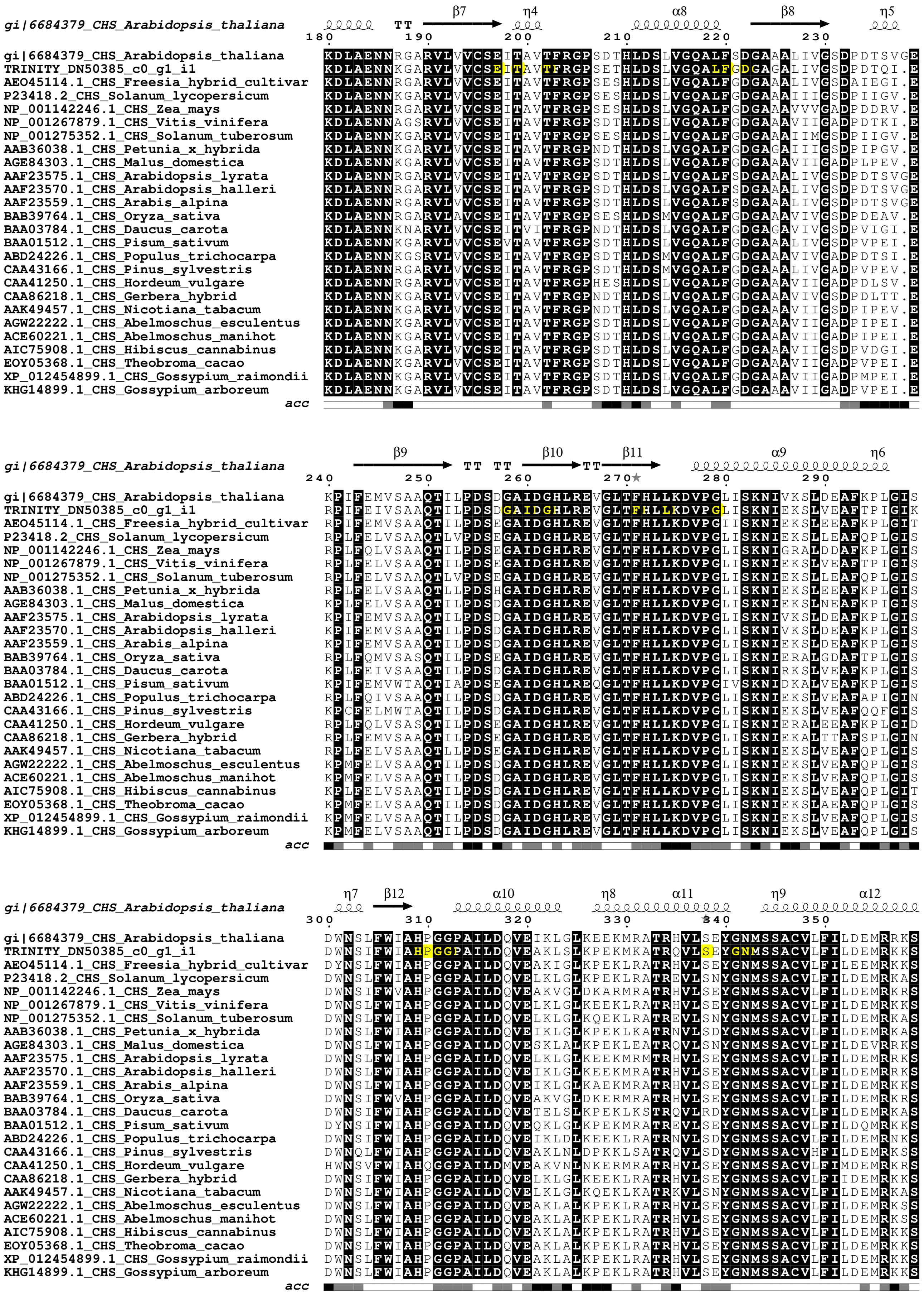

gi|6684379_CHS_Arabidopsis_thaliana
TT β7 η4 α8 β8 η5
180 190 200 210 220 230
gi|6684379_CHS_Arabidopsis_thaliana KDLAENNRGARVLVVCSEITAVTFRGPSDTHLDSLVGQALFSDGAAALIVGSDPDTSVGE
TRINITY_DN50385_c0_g1_i1 KDLAENNKGARVLVVCSEITAVTFRGPSETHLDSLVGQALFGDGAGALIVGSDPDTQI.E
AEO45114.1_CHS_Freesia_hybrid_cultivar KDLAENNRGARVLVVCSEITAVTFRGPSESHLDSLVGQALFGDGAAALIVGSDAIEGI.E
P23418.2_CHS_Solanum_lycopersicum KDLAENNKGARVLVVCSEITAVTFRGPSESHLDSLVGQALFGDGAAAIIIGSDPIIGV.E
NP_001142246.1_CHS_Zea_mays KDLAENNRGARVLVVCSEITAVTFRGPSESHLDSLVGQALFGDGAAAVVVGADPDDRV.E
NP_001267879.1_CHS_Vitis_vinifera KDLAENNAGSRVLVVCSEITAVTFRGPSDTHLDSLVGQALFGDGAAAVIIGADPDTKI.E
NP_001275352.1_CHS_Solanum_tuberosum KDLAENNKGARVLVVCSEITAVTFRGPSESHLDSLVGQALFGDGAAAIIMGSDPIIGV.E
AAB36038.1_CHS_Petunia_x_hybrida KDLAENNKGARVLVVCSEITAVTFRGPNDTHLDSLVGQALFGDGAGAIIIGSDPIPGV.E
AGE84303.1_CHS_Malus_domestica KDLAENNKGARVLVVCSEITAVTFRGPSDTHLDSLVGQALFGDGAAAVIIGADPLPEV.E
AAF23575.1_CHS_Arabidopsis_lyrata KDLAENNRGARVLVVCSEITAVTFRGPSDTHLDSLVGQALFSDGAAALIVGSDPDTSVGE
AAF23570.1_CHS_Arabidopsis_halleri KDLAENNRGARVLVVCSEITAVTFRGPSDTHLDSLVGQALFSDGAAALIVGSDPDTSVGE
AAF23559.1_CHS_Arabis_alpina KDLAENNRGARVLVVCSEITAVTFRGPSDTHLDSLVGQALFSDGAAALIVGSDPDTSVGE
BAB39764.1_CHS_Oryza_sativa KDLAENNRGARVLAVCSEITAVTFRGPSESHLDSMVGQALFGDGAAAVIVGSDPDEAV.E
BAA03784.1_CHS_Daucus_carota KDLAENNKNARVLVVCSEITVITFRGPNDTHLDSLVGQALFGDGAGAVIVGSDPVIGI.E
BAA01512.1_CHS_Pisum_sativum KDLAENNKGARVLVVCSEVTAVTFRGPSDTHLDSLVGQALFGDGAAALIVGSDPVPEI.E
ABD24226.1_CHS_Populus_trichocarpa KDLAENNKGSRVLVVCSEITAVTFRGPSDTHLDSMVGQALFGDGAAAVIVGADPDTSI.E
CAA43166.1_CHS_Pinus_sylvestris KDLAENNRGARVLVVCSEITAVTFRGPSDTHLDSMVGQALFGDGAAALIVGADPVPEV.E
CAA41250.1_CHS_Hordeum_vulgare KDLAENNRGARVLVVCSEITAVTFRGPHESHLDSLVGQALFGDGAAAVIIGADPDLSV.E
CAA86218.1_CHS_Gerbera_hybrid KDLAENNKGARVLVVCSEITAVTFRGPNDTHLDSLVGQALFGDGAAAVIVGSDPDLTT.E
AAK49457.1_CHS_Nicotiana_tabacum KDLAENNKGARVLVVCSEITAVTFRGPNDTHLDSLVGQALFGDGAAAVIIGSDPIPEV.E
AGW22222.1_CHS_Abelmoschus_esculentus KDLAENNKGARVLVVCSEITAVTFRGPSDTHLDSLVGQALFGDGAAAVIIGADPIPEI.E
ACE60221.1_CHS_Abelmoschus_manihot KDLAENNKGARVLVVCSEITAVTFRGPSDTHLDSLVGQALFGDGAAAVIIGADPIPEI.E
AIC75908.1_CHS_Hibiscus_cannabinus KDLAENNKGARVLVVCSEITAVTFRGPSDTHLDSLVGQALFGDGAAAVIIGSDPVDGI.E
EOY05368.1_CHS_Theobroma_cacao KDLAENNKGARVLVVCSEITAVTFRGPSDTHLDSLVGQALFGDGAAAVIIGADPVPEI.E
XP_012454899.1_CHS_Gossypium_raimondii KDLAENNKGARVLVVCSEITAVTFRGPSDTHLDSLVGQALFGDGAAAVIIGADPMPEI.E
KHG14899.1_CHS_Gossypium_arboreum KDLAENNKGARVLVVCSEITAVTFRGPSDTHLDSLVGQALFGDGAAAVIIGADPVPEI.E
acc
gi|6684379_CHS_Arabidopsis_thaliana
β9 TT TT β10 TT β11 α9 η6
240 250 260 270 280 290
gi|6684379_CHS_Arabidopsis_thaliana KPIFEMVSAAQTILPDSDGAIDGHLREVGLTFHLLKDVPGLISKNIVKSLDEAFKPLGIS
TRINITY_DN50385_c0_g1_i1 RPIFEIVSAAQTILPDSDGAIDGHLREVGLTFHLLKDVPGLISKNIEKSLVEAFTPIGIK
AEO45114.1_CHS_Freesia_hybrid_cultivar RPIFEMVSAAQTILPDSEGAIDGHLREVGLTFHLLKDVPGIISKNIEKSLEEAFKPLGIT
P23418.2_CHS_Solanum_lycopersicum RPLFELVSAAQTLVPDSEGAIDGHLREVGLTFHLLKDVPGLISKNIEKSLLEAFQPLGIS
NP_001142246.1_CHS_Zea_mays RPLFQLVSAAQTILPDSEGAIDGHLREVGLTFHLLKDVPGLISKNIGRALDDAFKPLGIS
NP_001267879.1_CHS_Vitis_vinifera RPLFELVSAAQTILPDSEGAIDGHLREVGLTFHLLKDVPGLISKNIEKSLVEAFTPIGIS
NP_001275352.1_CHS_Solanum_tuberosum RPLFELVSAAQTLVPDSEGAIDGHLREVGLTFHLLKDVPGLISKNIEKSLLEAFQPLGIS
AAB36038.1_CHS_Petunia_x_hybrida RPLFELVSAAQTLLPDSHGAIDGHLREVGLTFHLLKDVPGLISKNIEKSLEEAFKPLGIS
AGE84303.1_CHS_Malus_domestica KPLFELVSAAQTILPDSDGAIDGHLREVGLTFHLLKDVPGLISKNIEKSLNEAFKPIGIS
AAF23575.1_CHS_Arabidopsis_lyrata KPIFEMVSAAQTILPDSDGAIDGHLREVGLTFHLLKDVPGLISKNIVKSLDEAFKPLGIS
AAF23570.1_CHS_Arabidopsis_halleri KPIFEMVSAAQTILPDSDGAIDGHLREVGLTFHLLKDVPGLISKNIVKSLDEAFKPLGIS
AAF23559.1_CHS_Arabis_alpina KPIFEMVSAAQTILPDSDGAIDGHLREVGLTFHLLKDVPGLISKNIEKSLDEAFKPLGIS
BAB39764.1_CHS_Oryza_sativa RPLFQMVSASQTILPDSEGAIDGHLREVGLTFHLLKDVPGLISKNIERALGDAFTPLGIS
BAA03784.1_CHS_Daucus_carota KPLFEIVSAAQTILPDSDGAIDGHLREVGLTFHLLKDVPGLISKNIRKSLVEAFKPLGIS
BAA01512.1_CHS_Pisum_sativum KPIFEMVWTAQTIAPDSEGAIDGHLREQGLTFHLLKDVPGIVSKNIDKALVEAFKPLGIS
ABD24226.1_CHS_Populus_trichocarpa RPLFQIVSAAQTILPDSDGAIDGHLREVGLTFHLLKDVPGLISKNIEKSLVEAFAPIGIN
CAA43166.1_CHS_Pinus_sylvestris KPCFELMWTAQTILPDSDGAIDGHLREVGLTFHLLKDVPGLISKNIEKSLVEAFQQFGIS
CAA41250.1_CHS_Hordeum_vulgare RPLFQLVSASQTILPDSEGAIDGHLREVGLTFHLLKDVPGLISKNIERALEEAFKPLGID
CAA86218.1_CHS_Gerbera_hybrid RPLFEMVSAAQTILPDSEGAIDGHLREVGLTFHLLKDVPGLISKNIEKALTTAFSPLGIN
AAK49457.1_CHS_Nicotiana_tabacum RPLFELVSAAQTLLPDSEGAIDGHLREVGLTFHLLKDVPGLISKNIEKSLVEAFQPLGIS
AGW22222.1_CHS_Abelmoschus_esculentus KPMFELVSAAQTILPDSDGAIDGHLREVGLTFHLLKDVPGLISKNIEKSLVEAFQPLGIS
ACE60221.1_CHS_Abelmoschus_manihot KPMFELVSAAQTILPDSDGAIDGHLREVGLTFHLLKDVPGLISKNIEKSLVEAFQPLGIS
AIC75908.1_CHS_Hibiscus_cannabinus KPLFELVSAAQTILPDSDGAIDGHLREVGLTFHLLKDVPGLISKNIEKSLVEAFQPLGIT
EOY05368.1_CHS_Theobroma_cacao KPMFELVSAAQTILPDSDGAIDGHLREVGLTFHLLKDVPGLISKNIEKSLAEAFQPLGIS
XP_012454899.1_CHS_Gossypium_raimondii KPMFELVSAAQTILPDSDGAIDGHLREVGLTFHLLKDVPGLISKNIEKSLVEAFQPLGIS
KHG14899.1_CHS_Gossypium_arboreum KPMFELVSAAQTILPDSDGAIDGHLREVGLTFHLLKDVPGLISKNIEKSLVEAFQPLGIS
acc
gi|6684379_CHS_Arabidopsis_thaliana
η7 β12 α10 η8 α11 η9 α12
300 310 320 330 340 350
gi|6684379_CHS_Arabidopsis_thaliana DWNSLFWIAHPGGPAILDQVEIKLGLKEEKMRATRHVLSEYGNMSSACVLFILDEMRRKS
TRINITY_DN50385_c0_g1_i1 DWNSIFWIAHPGGPAILDQVEAKLSLKPEKMKATRQVLSEYGNMSSACVLFILDEMRKKS
AEO45114.1_CHS_Freesia_hybrid_cultivar DYNSLFWIAHPGGPAILDQVEAKIGLKPEKLRATRHVLSEYGNMSSACVLFILEEMRKKS
P23418.2_CHS_Solanum_lycopersicum DWNSLFWIAHPGGPAILDQVELKLGLKPEKLRATREVLSNYGNMSSACVLFILDEMRKAS
NP_001142246.1_CHS_Zea_mays DWNSIFWVAHPGGPAILDQVEAKVGLDKARMRATRHVLSEYGNMSSACVLFILDEMRKRS
NP_001267879.1_CHS_Vitis_vinifera DWNSLFWIAHPGGPAILDQVELKLGLKEEKLRATRHVLSEYGNMSSACVLFILDEMRKKS
NP_001275352.1_CHS_Solanum_tuberosum DWNSLFWIAHPGGPAILDQVELKLGLKQEKLRATREVLSNYGNMSSACVLFILDEMRKAS
AAB36038.1_CHS_Petunia_x_hybrida DWNSLFWIAHPGGPAILDQVEIKLGLKPEKLKATRNVLSDYGNMSSACVLFILDEMRKAS
AGE84303.1_CHS_Malus_domestica DWNSLFWIAHPGGPAILDQVESKLALKPEKLEATRQVLSNYGNMSSACVLFILDEVRRKS
AAF23575.1_CHS_Arabidopsis_lyrata DWNSLFWIAHPGGPAILDQVELKLGLKEEKMRMTRHVLSEYGNMSSACVLFILDEMRRKS
AAF23570.1_CHS_Arabidopsis_halleri DWNSLFWIAHPGGPAILDQVEIKLGLKEEKMRATRHVLSEYGNMSSACVLFILDEMRRKS
AAF23559.1_CHS_Arabis_alpina DWNSLFWIAHPGGPAILDQVEIKLGLKAEKMRATRHVLSEYGNMSSACVLFILDEMRKKS
BAB39764.1_CHS_Oryza_sativa DWNSIFWVAHPGGPAILDQVEAKVGLDKERMRATRHVLSEYGNMSSACVLFILDEMRKRS
BAA03784.1_CHS_Daucus_carota DWNSIFWIAHPGGPAILDQVETELSLKPEKLKSTRQVLRDYGNMSSACVLFILDEMRKAS
BAA01512.1_CHS_Pisum_sativum DYNSIFWIAHPGGPAILDQVEQKLGLKPEKMRATREVLSEYGNMSSACVLFILDQMRKKS
ABD24226.1_CHS_Populus_trichocarpa DWNSIFWIAHPGGPAILDQVEIKLDLKEEKLRATRNVLSDYGNMSSACVLFILDEMRNKS
CAA43166.1_CHS_Pinus_sylvestris DWNQLFWIAHPGGPAILDQVEAKLNLDPKKLSATRQVLSDYGNMSSACVHFILDEMRKSS
CAA41250.1_CHS_Hordeum_vulgare HWNSVFWIAHQGGPAILDMVEAKVNLNKERMRATRHVLSEYGNMSSACVLFIMDEMRKRS
CAA86218.1_CHS_Gerbera_hybrid DWNSIFWIAHPGGPAILDQVELKLGLKEEKLRATRHVLSEYGNMSSACVLFIIDEMRKKS
AAK49457.1_CHS_Nicotiana_tabacum DWNSLFWIAHPGGPAILDQVELKLGLKQEKLKATRKVLSNYGNMSSACVLFILDEMRKAS
AGW22222.1_CHS_Abelmoschus_esculentus DWNSLFWIAHPGGPAILDQVEAKLALKPEKLRATRHVLSEYGNMSSACVLFILDEMRKSS
ACE60221.1_CHS_Abelmoschus_manihot DWNSLFWIAHPGGPAILDQVEAKLALKPEKLRATRHVLSEYGNMSSACVLFILDEMRKSS
AIC75908.1_CHS_Hibiscus_cannabinus DWNSLFWIAHPGGPAILDQVEAKLALKPEKLRATRHVLSEYGNMSSACVLFILDEMRKKS
EOY05368.1_CHS_Theobroma_cacao DWNSLFWIAHPGGPAILDQVEAKLALKPEKLRATRHVLSEYGNMSSACVLFILDEMRKKS
XP_012454899.1_CHS_Gossypium_raimondii DWNSLFWIAHPGGPAILDQVEAKLALKPEKLRATRHVLSEYGNMSSACVLFILDEMRKKS
KHG14899.1_CHS_Gossypium_arboreum DWNSLFWIAHPGGPAILDQVEAKLALKPEKLRATRHVLSEYGNMSSACVLFILDEMRKKS
acc

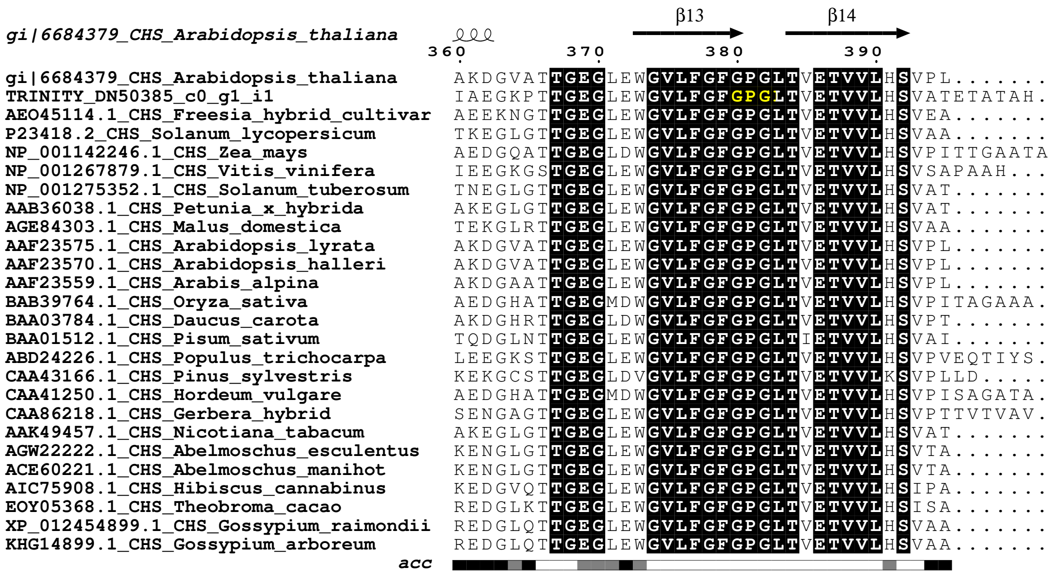

gi|6684379_CHS_Arabidopsis_thaliana
β13
β14
360
370
380
390
gi|6684379_CHS_Arabidopsis_thaliana AKDGVATTGEGLEWGVLFGFGPGLTVETVVLHSVPL.......
TRINITY_DN50385_c0_g1_i1 IAEGKPTTGEGLEWGVLFGFGPGLTVETVVLHSVATETATAH.
AEO45114.1_CHS_Freesia_hybrid_cultivar AEEKNGTTGEGLEWGVLFGFGPGLTVETVVLHSVEA.......
P23418.2_CHS_Solanum_lycopersicum TKEGLGTTGEGLEWGVLFGFGPGLTVETVVLHSVAA.......
NP_001142246.1_CHS_Zea_mays AEDGQATTGEGLDWGVLFGFGPGLTVETVVLHSVPITTGAATA
NP_001267879.1_CHS_Vitis_vinifera IEEGKGSTGEGLEWGVLFGFGPGLTVETVVLHSVSAPAAH...
NP_001275352.1_CHS_Solanum_tuberosum TNEGLGTTGEGLEWGVLFGFGPGLTVETVVLHSVAT.......
AAB36038.1_CHS_Petunia_x_hybrida AKEGLGTTGEGLEWGVLFGFGPGLTVETVVLHSVAT.......
AGE84303.1_CHS_Malus_domestica TEKGLRTTGEGLEWGVLFGFGPGLTVETVVLHSVAA.......
AAF23575.1_CHS_Arabidopsis_lyrata AKDGVATTGEGLEWGVLFGFGPGLTVETVVLHSVPL.......
AAF23570.1_CHS_Arabidopsis_halleri AKDGVATTGEGLEWGVLFGFGPGLTVETVVLHSVPL.......
AAF23559.1_CHS_Arabis_alpina AKDGAATTGEGLEWGVLFGFGPGLTVETVVLHSVPL.......
BAB39764.1_CHS_Oryza_sativa AEDGHATTGEGMDWGVLFGFGPGLTVETVVLHSVPITAGAAA.
BAA03784.1_CHS_Daucus_carota AKDGHRTTGEGLDWGVLFGFGPGLTVETVVLHSVPT.......
BAA01512.1_CHS_Pisum_sativum TQDGLNTTGEGLEWGVLFGFGPGLTIETVVLHSVAI.......
ABD24226.1_CHS_Populus_trichocarpa LEEGKSTTGEGLEWGVLFGFGPGLTVETVVLHSVPVEQTIYS.
CAA43166.1_CHS_Pinus_sylvestris KEKGCSTTGEGLDVGVLFGFGPGLTVETVVLKSVPLLD.....
CAA41250.1_CHS_Hordeum_vulgare AEDGHATTGEGMDWGVLFGFGPGLTVETVVLHSVPISAGATA.
CAA86218.1_CHS_Gerbera_hybrid SENGAGTTGEGLEWGVLFGFGPGLTVETVVLHSVPTTVTVAV.
AAK49457.1_CHS_Nicotiana_tabacum AKEGLGTTGEGLEWGVLFGFGPGLTVETVVLHSVAT.......
AGW22222.1_CHS_Abelmoschus_esculentus KENGLGTTGEGLEWGVLFGFGPGLTVETVVLHSVTA.......
ACE60221.1_CHS_Abelmoschus_manihot KENGLGTTGEGLEWGVLFGFGPGLTVETVVLHSVTA.......
AIC75908.1_CHS_Hibiscus_cannabinus KEDGVQTTGEGLEWGVLFGFGPGLTVETVVLHSIPA.......
EOY05368.1_CHS_Theobroma_cacao REDGLKTTGEGLEWGVLFGFGPGLTVETVVLHSISA.......
XP_012454899.1_CHS_Gossypium_raimondii REDGLQTTGEGLEWGVLFGFGPGLTVETVVLHSVAA.......
KHG14899.1_CHS_Gossypium_arboreum REDGLQTTGEGLEWGVLFGFGPGLTVETVVLHSVAA.......
acc

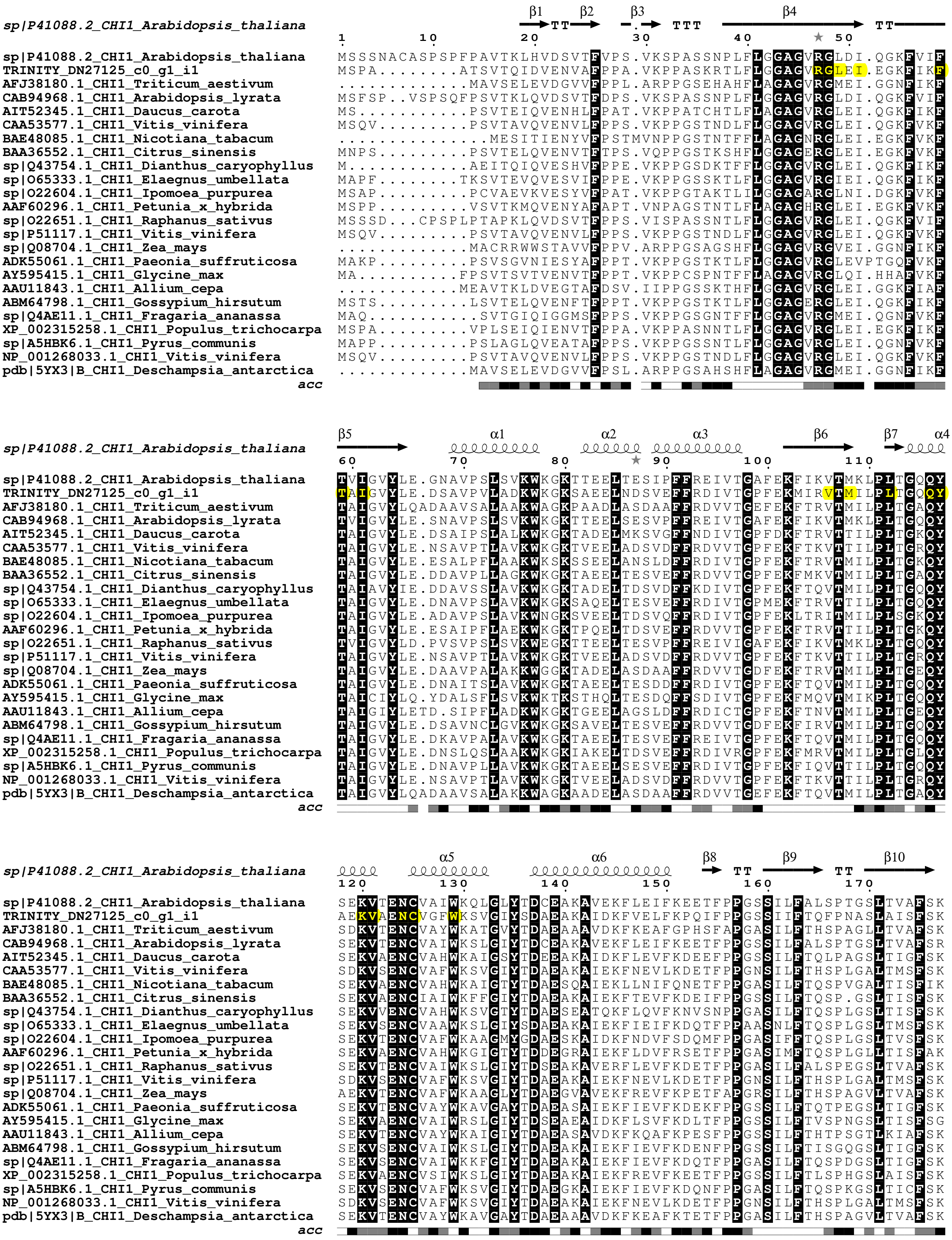

sp|P41088.2_CHI1_Arabidopsis_thaliana
β1 β2 β3 β4
TT TTT TT
1 10 20 30 40 50
sp|P41088.2_CHI1_Arabidopsis_thaliana MSSSNACASPSPFPAVTKLHVDSVTFVPS.VKSPASSNPLFLGGAGVRGLDI.QGKFVIF
TRINITY_DN27125_c0_g1_i1 MSPA........ATSVTQIDVENVAFPPA.VKPPASKRTLFLGGAGVRGLEI.EGKFIKF
AFJ38180.1_CHI1_Triticum_aestivum .............MAVSELEVDGVVFPPL.ARPPGSEHAHFLAGAGVRGMEI.GGNFIKF
CAB94968.1_CHI1_Arabidopsis_lyrata MSFSP..VSPSQFPSVTKLQVDSVTFDPS.VKSPASSNPLFLGGAGVRGLDI.QGKFVIF
AIT52345.1_CHI1_Daucus_carota MS..........PSVTEIQVENHLFPAT.VKPPATCHTLFLAGAGVRGLEI.QGKFIKF
CAA53577.1_CHI1_Vitis_vinifera MSQV........PSVTAVQVENVLFPPS.VKPPGSTNDLFLGGAGVRGLEI.QGKFVKF
BAE48085.1_CHI1_Nicotiana_tabacum ...............MESITIENYVFPSTMVNPPGSTNTFFLAGAGNRGLEI.EGKFVKF
BAA36552.1_CHI1_Citrus_sinensis MNPS........PSVTELQVENVTFTPS.VQPPGSTKSHFLGGAGERGLEI.EGKFVKF
sp|Q43754.1_CHI1_Dianthus_caryophyllus M...........AEITQIQVESHVFPPE.VKPPGSDKTFFLGGAGVRGLEI.QGKFIKF
sp|O65333.1_CHI1_Elaegnus_umbellata MAPF.......TKSVTEVQVESVIFPPE.VKPPGSSKTLFLGGAGVRGIEI.QGKFIKF
sp|O22604.1_CHI1_Ipomoea_purpurea MSAP........PCVAEVKVESYVFPAT.VKPPGTAKTLILGGAGARGLNI.DGKFVKF
AAF60296.1_CHI1_Petunia_x_hybrida MSPP........VSVTKMQVENYAFAPT.VNPAGSTNTLFLAGAGHRGLEI.EGKFVKF
sp|O22651.1_CHI1_Raphanus_sativus MSSSD...CPSPLPTAPKLQVDSVTFPPS.VISPASSNTLFLGGAGVRGLEI.QGKFVIF
sp|P51117.1_CHI1_Vitis_vinifera MSQV........PSVTAVQVENVLFPPS.VKPPGSTNDLFLGGAGVRGLEI.QGKFVKF
sp|Q08704.1_CHI1_Zea_mays .............MACRRWWSTAVVFPPV.ARPPGSAGSHFLGGAGVRGVEI.GGNFIKF
ADK55061.1_CHI1_Paeonia_suffruticosa MAKP........PSVSGVNIESYAFPPT.VKPPGSTKTLFLGGAGVRGLEVPTGQFVKF
AY595415.1_CHI1_Glycine_max MA..........FPSVTSVTVENVTFPPT.VKPPCSPNTFFLAGAGVRGLQI.HHAFVKF
AAU11843.1_CHI1_Allium_cepa ............MEAVTKLDVEGTAFDSV.IIPPGSSKTHFLGGAGVRGLEI.GGKFIAF
ABM64798.1_CHI1_Gossypium_hirsutum MSTS........LSVTELQVENFTFPPT.VKPPGSTKTLFLGGAGERGLEI.QGKFIKF
sp|Q4AE11.1_CHI1_Fragaria_ananassa MAQ..........SVTGIQIGGMSFPPS.VKPPGSGNTFFLGGAGVRGMEI.QGNFVKF
XP_002315258.1_CHI1_Populus_trichocarpa MSPA........VPLSEIQIENVTFPPA.VKPPASNNTLFLGGAGVRGLEI.EGKFIKF
sp|A5HBK6.1_CHI1_Pyrus_communis MAPP........PSLAGLQVEATAFPPS.VKPPGSSNTLFLGGAGVRGLEI.QGNFVKF
NP_001268033.1_CHI1_Vitis_vinifera MSQV........PSVTAVQVENVLFPPS.VKPPGSTNDLFLGGAGVRGLEI.QGKFVKF
pdb|5YX3|B_CHI1_Deschampsia_antarctica .............MAVSELEVDGVVFPSL.ARPPGSAHSHFLAGAGVRGMEI.GGNFIKF
acc

sp|P41088.2_CHI1_Arabidopsis_thaliana
β5 α1 α2 α3 β6 β7 α4
60 70 80 90 100 110
sp|P41088.2_CHI1_Arabidopsis_thaliana TVIGVYLE.GNAVPSLSVKWKGKTTEELTESIPFFREIVTGAFEKFIKVTMKLPLTGQQY
TRINITY_DN27125_c0_g1_i1 TAIGVYLE.DSAVPVLADKWKGKSAEELNDSVEFFRDIVTGPFEKMIRVTMILPLTGQQY
AFJ38180.1_CHI1_Triticum_aestivum TAIGVYLQADAAVSALAAKWAGKPAADLASDAAFFRDVVTGEFEKFTRVTMILPLTGAQY
CAB94968.1_CHI1_Arabidopsis_lyrata TVIGVYLE.SNAVPSLSVKWKGKTTEELSESVPFFREIVTGAFEKFIKVTMKLPLTGQQY
AIT52345.1_CHI1_Daucus_carota TAIGVYLE.DSAIPSLALKWKGKTADELMKSVGFFNDIVTGPFDKFTRVTTILPLTGKQY
CAA53577.1_CHI1_Vitis_vinifera TAIGVYLE.NSAVPTLAVKWKGKTVEELADSVDFFRDVVTGPFEKFTKVTTILPLTGRQY
BAE48085.1_CHI1_Nicotiana_tabacum TAIGVYLE.ESALPFLAAKWKSKSSEELANSLDFFRDIVTGPFEKFTRVTMILPLTGKQY
BAA36552.1_CHI1_Citrus_sinensis TAIGVYLE.DDAVPLLAGKWKGKTAEELTESVEFFRDVVTGPFEKFMKVTMILPLTGAQY
sp|Q43754.1_CHI1_Dianthus_caryophyllus TAIAVYIE.DDAVSSLAVKWKGKTADELTDSVDFFKDIVTGPFEKFTQVTMILPLTGQQY
sp|O65333.1_CHI1_Elaegnus_umbellata TAIGVYLE.DNAVPSLAVKWKGKSAQELTESVEFFRDIVTGPMEKFTRVTTILPLTGQQY
sp|O22604.1_CHI1_Ipomoea_purpurea TAIGVYLE.ADAVPSLAVKWNGKSVEELTDSVQFFRDIVTGPFEKLTRITMILPLSGKQY
AAF60296.1_CHI1_Petunia_x_hybrida TAIGVYLE.ESAIPFLAEKWKGKTPQELTDSVEFFRDVVTGPFEKFTRVTTILPLTGKQY
sp|O22651.1_CHI1_Raphanus_sativus TVIGVYLD.PVSVPSLSVKWEGKTTEELTESVPFFREIVIGAFEKFIKVTMKLPLTGQQY
sp|P51117.1_CHI1_Vitis_vinifera TAIGVYLE.NSAVPTLAVKWKGKTVEELADSVDFFRDVVTGPFEKFTKVTTILPLTGRQY
sp|Q08704.1_CHI1_Zea_mays TAIGVYLE.DAAVPALAKKWGGKTADELASDAAFFRDVVTGDFEKFTRVTMILPLTGEQY
ADK55061.1_CHI1_Paeonia_suffruticosa TAIGVYLE.DNAITSLAVKWKGKTAEELTESDDFFRDIVTGPFEKFTQVTMILPLTGQQY
AY595415.1_CHI1_Glycine_max TAICIYLQ.YDALSFLSVKWKTKSTHQLTESDQFFSDIVTGPFEKFMQVTMIKPLTGQQY
AAU11843.1_CHI1_Allium_cepa TAIGIYLETD.SIPFLADKWKGKTGEELAGSLDFFRDICTGPFEKFTNVTMILPLTGEQY
ABM64798.1_CHI1_Gossypium_hirsutum TAIGVYLE.DSAVNCLGVKWKGKSAVELTESVEFFRDVVTGDFEKFIRVTMILPLTGQQY
sp|Q4AE11.1_CHI1_Fragaria_ananassa TAIGVYLE.DKAVPALAVKWKGKTAEELTESVEFFREIVTGPFEKFTQVTMILPLTGQQY
XP_002315258.1_CHI1_Populus_trichocarpa TAIGVYLE.DNSLQSLAAKWKGKIAKELTDSVEFFRDIVRGPFEKFMRVTMILPLTGLQY
sp|A5HBK6.1_CHI1_Pyrus_communis TAIGVYLE.DNAVPLLAVKWKGKTAEELSESVEFFRDIVTGPFEKFIQVTTILPLTGQQY
NP_001268033.1_CHI1_Vitis_vinifera TAIGVYLE.NSAVPTLAVKWKGKTVEELADSVDFFRDVVTGPFEKFTKVTTILPLTGRQY
pdb|5YX3|B_CHI1_Deschampsia_antarctica TAIGVYLQADAAVSALAKKWAGKAADELASDAAFFRDVVTGEFEKFTQVTMILPLTGAQY
acc

sp|P41088.2_CHI1_Arabidopsis_thaliana
α5 α6 β8 β9 β10
TT TT
120 130 140 150 160 170
sp|P41088.2_CHI1_Arabidopsis_thaliana SEKVTENCVAIWKQLGLYTDCEAKAVEKFLEIFKEETFPPGSSILFALSPTGSLTVAFSK
TRINITY_DN27125_c0_g1_i1 AEKVAENCVGFWKSVGIYSDAEAKAIDKFVELFKPQIFPPGSSIIFTQFPNASLAISFSK
AFJ38180.1_CHI1_Triticum_aestivum SDKVTENCVAYWKATGVYTDAEAAAVDKFKEAFGPHSFAPGASILFTHSPAGLLTVAFSK
CAB94968.1_CHI1_Arabidopsis_lyrata SEKVTENCVAIWKSLGIYTDCEAKAVEKFLEIFKEETFPPGSSILFALSPTGSLTVAFSR
AIT52345.1_CHI1_Daucus_carota SEKVAENCVAHWKAIGSYTDEEAKAIDKFLEVFKDEEFPPGSSILFTQLPAGSLTIGFSK
CAA53577.1_CHI1_Vitis_vinifera SDKVSENCVAFWKSVGIYTDAEAKAIEKFNEVLKDETFPPGNSILFTHSPLGALTMSFSK
BAE48085.1_CHI1_Nicotiana_tabacum SEKVAENCVAHWKAIGTYTDAESQAIEKLLNIFQNETFPPGASILFTQSPVGALTISFIK
BAA36552.1_CHI1_Citrus_sinensis SEKVAENCIAIWKFFGIYTDAEAKAIEKFTEVFKDEIFPPGSSILFTQSP.GSLTISFSK
sp|Q43754.1_CHI1_Dianthus_caryophyllus SEKVVENCVAHWKSVGTYTDAESEATQKFLQVFKNVSNPPGASILFTQSPLGSLTISFSK
sp|O65333.1_CHI1_Elaegnus_umbellata SEKVSENCVAAWKSLGIYSDAEAKAIEKFIEIFKDQTFPPAASNLFTQSPLGSLTMSFSK
sp|O22604.1_CHI1_Ipomoea_purpurea SEKVTENCVAFWKAAGMYGDAESKAIDKFNDVFSDQMFPPGASIFFTQSPLGSLTVSFSK
AAF60296.1_CHI1_Petunia_x_hybrida SEKVAENCVAHWKGIGTYTDDEGRAIEKFLDVFRSETFPPGASIMFTQSPLGLLTISFAK
sp|O22651.1_CHI1_Raphanus_sativus SEKVTENCVAIWKSLGIYTDSEAKAVERFLEVFKDETFPPGASILFALSPEGSLTVAFSK
sp|P51117.1_CHI1_Vitis_vinifera SDKVSENCVAFWKSVGIYTDAEAKAIEKFNEVLKDETFPPGNSILFTHSPLGALTMSFSK
sp|Q08704.1_CHI1_Zea_mays AEKVTENCVAFWKAAGLYTDAEGVAVEKFREVFKPETFAPGRSILFTHSPAGVLTVAFSK
ADK55061.1_CHI1_Paeonia_suffruticosa SEKVTENCVAYWKAVGAYTDAEASAIEKFIEVFKDEKFPPGSSILFTQTPEGSLTIGFSK
AY595415.1_CHI1_Glycine_max SEKVAENCVAIWRSLGIYTDSEAEAIDKFLSVFKDLTFPPGSSILFTVSPNGSLTISFSG
AAU11843.1_CHI1_Allium_cepa SEKVTENCVAYWKAIGIYTDAEASAVDKFKQAFKPESFPPGSSILFTHTPSGTLKIAFSK
ABM64798.1_CHI1_Gossypium_hirsutum SEKVSENCVAIWKSLGIYTDAEAKAIEKFIEVFKDENFPPGSSILFTISGQGSLTIGFSK
sp|Q4AE11.1_CHI1_Fragaria_ananassa SEKVSENCVAIWKKFGIYTDAEAKAIEKFIEVFKDQTFPPGASILFTQSPDGSLTIGFSK
XP_002315258.1_CHI1_Populus_trichocarpa SEKVAENCVSIWKSLGIYTDAEAKAIEKFREVFKEETFPPGSSILFTLSPHGSLAISFSK
sp|A5HBK6.1_CHI1_Pyrus_communis SEKVSENCVAFWKSVGIYTDAEGKAIEKFIEVFKDQNFPPGASILFTQSPKGSLTICFSK
NP_001268033.1_CHI1_Vitis_vinifera SDKVSENCVAFWKSVGIYTDAEAKAIEKFNEVLKDETFPPGNSILFTHSPLGALTMSFSK
pdb|5YX3|B_CHI1_Deschampsia_antarctica SEKVTENCVAYWKAVGAYTDAEAAAVDKFKEAFKTETFPPGASILFTHSPAGVLTVAFSK
acc

```
                                            β11      α7                      α8                 α9
sp|P41088.2_CHI1_Arabidopsis_thaliana                                                       ...........
                                       180       190       200       210       220
sp|P41088.2_CHI1_Arabidopsis_thaliana  DDSIPETGIAVIENKLLAEAVLESIIGKNGVSPGTRLSVAERLSQLMMKNKD........
TRINITY_DN27125_c0_g1_i1               DGCIPDAENCLIENKLLSEAVLESIIGKHGVSPEARKCMAVRLVELFEKDAQ.......K
AFJ38180.1_CHI1_Triticum_aestivum      DSSVPESGGVAIDNARLCEAVLESIIGEHGVSPAAKLSLATRVAELL.EGAA........
CAB94968.1_CHI1_Arabidopsis_lyrata     DDSIPETGIAVIENKLLAEAVLESIIGKNGVSPGTRLSIAERLAKLMTKNKV........
AIT52345.1_CHI1_Daucus_carota          ETSIPKDGKAVIHNKQLAEAVLDSIIGEHGVSPAAKHSLAVRISDLLKHHDH........
CAA53577.1_CHI1_Vitis_vinifera         DGSLPEVGNAVIENKLLTEAVLESIIGKHGVSPEAKKSLAARLSELF..CKE........
BAE48085.1_CHI1_Nicotiana_tabacum      DDSITGTGNAVIENKQLSEAVLESIIGKHGVSPAAKCSIAERVSGLFKKSYA........
BAA36552.1_CHI1_Citrus_sinensis        DGSIPKDGVAVIESNLLSEAVLESMIGKNGVSPAAKKSLAERLSALLNVTSD........
sp|Q43754.1_CHI1_Dianthus_caryophyllus DGSLPENGKEVIVNKQLSEAVLQSIIGKHGVSPEAKKSLSARLSDLINQHEN........
sp|O65333.1_CHI1_Elaegnus_umbellata    DGSIPEVGNAVLENKLLSEAVLESIIGKHGVSPEAKQNLATRLVQLLNENSTTDLNESEN
sp|O22604.1_CHI1_Ipomoea_purpurea      DGSMPEIASAVIENKPLSEAVLESIIGSKGVSPEAKQSLAVRLSEMFINGGD........
AAF60296.1_CHI1_Petunia_x_hybrida      DDSVTGTANAVIENKQLSEAVLESIIGKHGVSPAAKCNVAERVAELLKKSYA........
sp|O22651.1_CHI1_Raphanus_sativus      DDSIPETGKAVIENKLLAEAVLESIIGKNRVSPGARLRVAERLAQLMKENKV........
sp|P51117.1_CHI1_Vitis_vinifera        DGSLPEVGNAVIENKLLTEAVLESIIGKHGVSPEAKKSLAARLSELF..CKE........
sp|Q08704.1_CHI1_Zea_mays              DSSVPAAGGVAIENKRLCEAVLESIIGERGVSPAAKLSLAARVSELLAKETA........
ADK55061.1_CHI1_Paeonia_suffruticosa   DGVLPEVGNAVVENKQLSEAVLESIIGKHGVSPEAKQSLAARISELLK............
AY595415.1_CHI1_Glycine_max            DETIPEVTSAVIENKLLSEAVLESMIGKNGVSPAAKQSLASRLSHLFKEPGVC.......
AAU11843.1_CHI1_Allium_cepa            DGLVPKDEGVLIENKALTQAVLESIIGEHGVSPAAKLSIASRLSEIMNKVGN........
ABM64798.1_CHI1_Gossypium_hirsutum     DSSVPEGGKVVIENKLLANSVLESVIGKNGVSPAAKESLASRLSPLFNDCG.........
sp|Q4AE11.1_CHI1_Fragaria_ananassa     DGCIPEVGNAVIENKLLSESVLESIIGKPGVSPEARKSVATRLSELLKESDHC.......
XP_002315258.1_CHI1_Populus_trichocarpa DGSVPEIENAVIENKLLSEAVLESMIGKHGVSPAAKQSLATTLSELLKESNE........
sp|A5HBK6.1_CHI1_Pyrus_communis        DASVPEAANAVIENKLLSEAVLESILGKHGVSPAAKRSLAARLSELLNGCK.........
NP_001268033.1_CHI1_Vitis_vinifera     DGSLPEVGNAVIENKLLTEAVLESIIGKHGVSPEAKKSLAARLSELF..CKE........
pdb|5YX3|B_CHI1_Deschampsia_antarctica NSSVPESGGAAIENRPLCEAVLESIIGEHGVSPAAKLSLATRVAELL.KEAA........
                                   acc

sp|P41088.2_CHI1_Arabidopsis_thaliana  ....
                                         230       240
sp|P41088.2_CHI1_Arabidopsis_thaliana  ....EKEVSDHSVEE....KLAKEN...................
TRINITY_DN27125_c0_g1_i1               EELIDKSPQNVGMVE....KPKETADFIQI..T..PQKNVN...
AFJ38180.1_CHI1_Triticum_aestivum      ......LAGGEPAAE.....................AVSVSV
CAB94968.1_CHI1_Arabidopsis_lyrata     ....EEDASNHSIDE....KLAKEN...................
AIT52345.1_CHI1_Daucus_carota          .........EVTGNG....KA.EVGKMASKDTE..TETPVQV..
CAA53577.1_CHI1_Vitis_vinifera         ...........AGDE....KI.EAEKVA..........PVAC..
BAE48085.1_CHI1_Nicotiana_tabacum      .........DASVFE....KPGIEKSSDPVIEEKPTIPEIGV..
BAA36552.1_CHI1_Citrus_sinensis        ....................................KMK...
sp|Q43754.1_CHI1_Dianthus_caryophyllus ...........PKKE.............................
sp|O65333.1_CHI1_Elaegnus_umbellata    EKL...NSNEVSKEE....KPLQVEKSAF........KEVEV..
sp|O22604.1_CHI1_Ipomoea_purpurea      ...........AISG....KVGVENDVIP........QTVVVSK
AAF60296.1_CHI1_Petunia_x_hybrida      ........EEASVFG....KPETEKS.........TIPVIGV..
sp|O22651.1_CHI1_Raphanus_sativus      ....EEDATKTDQEEANDLSLAKEN...................
sp|P51117.1_CHI1_Vitis_vinifera        ...........AGDE....KI.EAEKVA..........PVAC..
sp|Q08704.1_CHI1_Zea_mays              ......AAADAPQAE.....................PVSITA
ADK55061.1_CHI1_Paeonia_suffruticosa   ............................................
AY595415.1_CHI1_Glycine_max            ...................DPQSHK...................
AAU11843.1_CHI1_Allium_cepa            ......VEEKLPVLS.............................
ABM64798.1_CHI1_Gossypium_hirsutum     ...........ADSE....KPQS.....................
sp|Q4AE11.1_CHI1_Fragaria_ananassa     ..........VAGNG....KVDECTKEA..........EVKA..
XP_002315258.1_CHI1_Populus_trichocarpa ...........NGN..............................
sp|A5HBK6.1_CHI1_Pyrus_communis        ............................................
NP_001268033.1_CHI1_Vitis_vinifera     ...........AGDE....KI.EAEKVA..........PVAC..
pdb|5YX3|B_CHI1_Deschampsia_antarctica ......SVDEPAAAE.....................PVSVSA
                                   acc
```

```
                                                        η1         η2          β1
NP_190692.1_F3H_Arabidopsis_thaliana
                                       1           10         20        30        40
NP_190692.1_F3H_Arabidopsis_thaliana   MA.........PGTL....TELAGESKLNSKFVRDEDERPKVAYNVFSDEIPVISL..A
TRINITY_DN33424_c3_g3_i1               MAP........PSTL....TALSEEKTLQASFVRDEDERPKVAYNEFSNEIPVISL..A
AAU04792.1_F3H_Fragaria_x_ananassa     MAP.......TPTTL....TAIAGEKTLQQSFVRDEDERPKVAYNQFSNDIPIISL..S
NP_001236797.1_F3H_Glycine_max         MAP.......TAKTL....TYLAQEKTLESSFVRDEEERPKVAYNEFSDEIPVISL..A
ACR15123.1_F3H_Medicago_truncatula     MAP........AQTL....TYLAQEKTLESSFVREEDERPKVAYNNFSNEIPIISL..D
AQX36284.1_F3H_Prunus_persica          MA........PATTL....TSIAGEKTLQQKFVRDEDERPKVAYNNFSNEIPIISL..A
ABR13013.1_F3H_Triticum_aestivum       MAPV......SNET..FLPTAAWGEATLRPSFVRDEDERPKVAHDRLSDAVPVISL..D
NP_001130275.1_F3H_Zea_mays            MAPV......SISAVPFLPTAAEGETKLRASFVREEDERPKVPHDRFSDEVPVVSL..E
ACL54955.1_F3H_Actinidia_chinensis     MAP........TTTL....TALAEEKTLQSKFVRDEDERPKVAYNVFSSEIPVISL..A
AFN70721.1_F3H_Nekemias_grossedentata  MA.........PTTL....TALAGEKTLQSSFVRDEDERPKVAYNEFSNEIPIISL..E
ACM62745.1_F3H_Garcinia_mangostana     MAP.......TPTTL....TALEGETILRASFVRDEDERPKVAYNQFSNDVPVISL..E
AEO36935.1_F3H_Canarium_album          MAP........PATL....TTLAQQKTLEASFVRDEDERPKVAYNQFSNEIPVISL..A
AEG64806.1_F3H_Carthamus_tinctorius    MAP........ISSL.....KWDQGSAVENKYVRDEDERPKVPYDQFSNDIPVISL..E
AFC37250.1_F3H_Camellia_chekiangoleosa MAP........TTTL....TALAQEKSLQQKFVRDEDERPKAAYNVFSNEIPVISL..A
ACB56921.1_F3H_Pilosella_officinarum   MAP.........ASM.....KWDENSLLEKGFLRDEDERPKVPYNQFSNEIPVISL..E
AAB97310.1_F3H_Chrysanthemum_x_morifolium ....................MDDNSLHENRFVRDEDERPKVPYNKFSSEIPVISL..K
AAC15414.1_F3H_Nicotiana_tabacum       ....................MDDNSLHENRFVRDEDERPKVPYNKFSSEIPVISL..K
ABL86673.1_F3H_Gossypium_barbadense    MA.........PSTL....TALAEEKTLQASFVRDEDERPKVAYNQFSNDIPVISL..A
ADC96713.1_F3H_Gossypium_hirsutum      MA.........PSTL....TALAEEKTLQASFVRDEDERPKVAYNQFSNDIPVISL..A
XP_007046698.1_F3H_Theobroma_cacao     MA.........PSTL....TALAEEKTLQASFVRDEDERPKVAYNQFSNEIPVISL..A
ABO48521.1_F3H_Dimocarpus_longan       MA.........PATL....TALAQEKTLNASFVRDEDERPKVAYNEFSKEIPVISL..A
ACQ99190.1_F3H_Fagopyrum_tataricum     MAPV.....AKPSTL....TALAEEKTLNLSFVRDEDERPKVAYNHFSNEIPVISL..A
BAL43067.1_F3H_Ipomoea_coccinea        MT........TLSTL....TALAGETALRSSFVRDEDERPKVGYNEFSDEIPVISL..K
AAB41102.1_F3H_Ipomoea_purpurea        MT........TVSTL....TALAGETALRSSFVRDEDERPKVGYNEFSDEIPVISL..K
BAK78917.1_F3H_Ipomoea_quamoclit       MT........TLSTL....TALAGETALRSSFVRDEDERPKVGYNEFSDEIPVISL..K
ADO95201.1_F3H_Litchi_chinensis        MA.........LATL....TALAQEKTLNASFVRDEDERPKVAYNEFSNEIPVISL..A
AIB06738.1_F3H_Mangifera_indica        MA.........PATL....TALAQEKTLNSKFVRDEDERPKVAYNEFSNEIPVISL..A
AEF14415.1_F3H_Onobrychis_viciifolia   MAP.......AARTL....THLAKEKTLESSFVRDEDERPKVAYNNFSTEIPVISL..T
AFI71897.1_F3H_Paeonia_lactiflora      MAP.......TATTL....TALAGEKTLQSSFVRDEDERPKVAYNQFSNEIPIISL..A
AEN71544.1_F3H_Paeonia_suffruticosa    MAP.......TATTL....TALAGEKTLQSSFVRDEDERPKVAYNQFSNEIPIISL..A
CAA53579.1_F3H_Vitis_vinifera          MA.........PTTL....TALAGEKTLQSSFVRDEDERPKVAYNDFSNEIPVISLTKE
AGS57503.1_F3H_Vitis_rotundifolia      MA.........PTTL....TALAGEKTLQSSFVRDEDERPKVAYNDFSNEIPVISLTKE
ACM17897.1_F3H_Rubus_occidentalis      MAP.......TPTTL....TAIAGEKTLQQSFVRDEDERPKVAYNQFSNEIPIISL..S
ABW74548.1_F3H_Rubus_coreanus          MAP.......TPTTL....TAIAGEKTLQQSFVRDEDERPKVAYNQFSNEIPIISL..S
ADP09378.1_F3H_Pyrus_pyrifolia         MAP.......PAATL....TSIAHEKTLQQKFVRDEDERPKVAYNEFSNEIPIISL..A
AGL50918.1_F3H_Pyrus_communis          MA........PATTL....TSIAHEKTLQQKFVRDEDERPKVAYNEFSNEIPIISL..A
ACP30361.1_F3H_Malus_hybrid_cultivar   MA........PATTL....TSIAHEKTLQQKFVRDEDERPKVAYNDFSNEIPIISL..A
ABG78792.1_F3H_Aethusa_cynapium        MA.........PTTL....TALAQEKTLNSKFVRDEDERPKVAYNQFSNEIPVISL..A
AAX21540.1_F3H_Anethum_graveolens      MA.........PSTL....TALAQEKTLNSKFVRDEDERPKIAYNQFSDEIPVISL..A
AAX21539.1_F3H_Ammi_majus              MA.........PTTL....TALAQEKTLNSKFVRDEDERPKIAYNQFSDEIPVISL..A
AAU04791.1_F3H_Fragaria_x_ananassa     MAP.......TPTTL....TAIAGENTLQQSFVRDEDERPKVAYNQFSIDIPIISL..S
AAU93347.1_F3H_Ginkgo_biloba           MAPVQSVSAAPVPKFL....PTENEAKVLQSIFVRDEDERPKVAYNEFSKEIPIISL..A
AAT94365.1_F3H_Glycine_max             MAP.......TAKTL....TYLAQEKTLESSFVRDEEERPKVAYNEFSDEIPVISL..A
AAX21535.1_F3H_Pimpinella_anisum       MA.........PSTL....TALAQEKTLNSKFVRDEDERPKIAYNQFSDEIPVISL..A
AAP57394.1_F3H_Petroselinum_crispum    MA.........PSTL....TALAQEKTLNSKFVRDEDERPKIAYNKFSDEIPVISL..A
AAC49929.1_F3H_Petunia_x_hybrida       MA.........PSTL....TALAEEKTLQTSFIRDEDERPKVAYNQFSNEIPIISL..E
                                 acc

                                                          α1                β2          α2
NP_190692.1_F3H_Arabidopsis_thaliana   TT
                                           50               60        70        80        90
NP_190692.1_F3H_Arabidopsis_thaliana   GID.DVD.......GKRGEICRQIVEACENWGIFQVVDHGVDTNLVADMTRLARDFFALP
TRINITY_DN33424_c3_g3_i1               GIE...G.......ERRAEICKKIVEACEDWGIFQVIDHGVDSKLISEMTRLAKEFFALP
AAU04792.1_F3H_Fragaria_x_ananassa     GIE.EVE.......GRRAEICKKIVEACEDWGVFQIVDHGIDPKLISEMTRLAREFFALP
NP_001236797.1_F3H_Glycine_max         GID.EVD.......GRRREICEKIVEACENWGIFQVVDHGVDQQLVAEMTRLAKEFFALP
ACR15123.1_F3H_Medicago_truncatula     GID.DAG.......GRRAEICNKIVEACENWGIFQVVDHGVDSKLISEMTRFAKGFFDLP
AQX36284.1_F3H_Prunus_persica          GID.EVE.......GRRADICKKIVEACEDWGIFQIVDHGVDTKLISEMTGLAREFFALP
ABR13013.1_F3H_Triticum_aestivum       GID.......G..ARRAEIRDRVAAACEGWGIFQVVDHGVDADLIADMTRLSREFFALP
NP_001130275.1_F3H_Zea_mays            GID.......G..ARRAEIRARVAAACEDWGIFQVVDHGVDAALVADMARLARDFFALP
ACL54955.1_F3H_Actinidia_chinensis     GID.EVD.......GRRSEICRKIVEACEDWGIFQVVDHGVDAKLVGEMTRLARDFFALP
AFN70721.1_F3H_Nekemias_grossedentata  GID.EVG.......GRRDEICRKIVEACEDWGIFQVVNHGVDSNLISEMTRLAREFFALP
ACM62745.1_F3H_Garcinia_mangostana     GID.EGG.......QKRAEICKKIVEACEEWGIFQVVDHGVDTKLVSEMTRLARAFFALP
AEO36935.1_F3H_Canarium_album          GID.EVD.......GRRAEICKKIVEACEDWGIFQVIDHGVDANLISDMTRLAREFFALP
AEG64806.1_F3H_Carthamus_tinctorius    GID.DADGAG....SRRGEICAKIVKACEDWGIFQAVDHGVDTKLMSEMTRLAREFFQLP
AFC37250.1_F3H_Camellia_chekiangoleosa GID.EIE.......GRRSEICRKIVEACEDWGIFQVVDHGVDANLVAEMTRLAREFFALP
ACB56921.1_F3H_Pilosella_officinarum   GID.DVEGSGGG.RSRRAEICEKIVKAFEDWGIFQVVDHGVDAKLLSEMKRLAREFFLMP
AAB97310.1_F3H_Chrysanthemum_x_morifolium GID.DVEESSGGIKSRRAEICEKIIKACEDWGIFQVVDHGVDTKMLSEMTRLAREFFEMP
AAC15414.1_F3H_Nicotiana_tabacum       GID.DVEESSGGIKSRRAEICEKIIKACEDWGIFQVVDHGVDTKMLSEMTRLAREFFEMP
ABL86673.1_F3H_Gossypium_barbadense    GID.DVD.......GKRGEICKKIVEACEDWGVFQVVDHGVDTKLVSEMTRFAREFFALP
ADC96713.1_F3H_Gossypium_hirsutum      GID.DVD.......GKRGEICKKIVEACEDWGVFQVVDHGVDTKLVSEMTRFAREFFALP
XP_007046698.1_F3H_Theobroma_cacao     GID.DVD.......GNRAEICKKIVEACEDWGVFQVIDHGVDTKLISEMTRLAREFFALP
ABO48521.1_F3H_Dimocarpus_longan       GID.EVD.......GRRAEICKKIVEACEDWGVFQVIDHGVDTKLISDMTRLAREFFALP
ACQ99190.1_F3H_Fagopyrum_tataricum     GID.EVD.......GKRAEICNKIVKACEEWGIFQVIDHGVDTSLVSEMTRLATEFFALP
BAL43067.1_F3H_Ipomoea_coccinea        GID.DFL.......GRRVQICNDIVKACEDWGIFQVVDHGVDAELIGEMTRLSKDFFSLP
AAB41102.1_F3H_Ipomoea_purpurea        GID.DVL.......GRRVQIRKEIVKACEDWGIFQVVDHGVDAGLIGEMTRLSKDFFSLP
BAK78917.1_F3H_Ipomoea_quamoclit       GID.DVL.......GRRVQIRDEVVKACEDWGIFQVVDHGVDAGLIAEMTRLSKEFFSLP
ADO95201.1_F3H_Litchi_chinensis        GID.EVD.......GRRAEICQKIVEACEDWGIFQVIDHGVDTKLISDMTRLAREFFALP
AIB06738.1_F3H_Mangifera_indica        GID.EVD.......GRRAEICKKIVEACEEWGIFQVIDHGVDIKLISDMTALAREFFSLP
AEF14415.1_F3H_Onobrychis_viciifolia   GID.EVD.......GRRAEICKKIVEACENWGIFQVIDHGIDTNLISDMTRLAKLFFDLP
AFI71897.1_F3H_Paeonia_lactiflora      GID.D.D.......GQRSEICRKIVDACEDWGIFQVIDHGVDANLISDMNRLAREFFALP
AEN71544.1_F3H_Paeonia_suffruticosa    GID.D.D.......GQRSEICRKIVGACEDWGIFQVVDHGVDANLISDMNRLAREFFALP
CAA53579.1_F3H_Vitis_vinifera          SM..KLA.......AVVDEICRKIVEACEDWGIFQVVNHGVDSNLISEMTRLAREFFALP
AGS57503.1_F3H_Vitis_rotundifolia      SM..KLA.......AVVDEICRKIVEACEDWGIFQVVNHGVDSNLISEMTRLAREFFALP
ACM17897.1_F3H_Rubus_occidentalis      GID.EVE.......GRRAEICNKIVEACEDWGVFQIVDHGVDAKLISEMTRLARDFFALP
ABW74548.1_F3H_Rubus_coreanus          GID.EVE.......GRRAEICNKIVEACEDWGVFQIVDHGVDAKLISEMTRLARDFFALP
ADP09378.1_F3H_Pyrus_pyrifolia         GID.EVE.......GRRAEICKKIVEACEDWGIFQIVDHGVDAELVSEMTGLAREFFALP
AGL50918.1_F3H_Pyrus_communis          GID.EVE.......GRRAEICKKIVEACEDWGIFQIVDHGVDAELVSEMTGLAREFFALP
ACP30361.1_F3H_Malus_hybrid_cultivar   GID.EVE.......GRRGEICKKIVAACEDWGIFQIVDHGVDAELISEMTGLAREFFALP
ABG78792.1_F3H_Aethusa_cynapium        GIDDDSG.......DKRSQICRKIVEACEDWGIFQVVDHGIDSHLISEMTRLAREFFALP
AAX21540.1_F3H_Anethum_graveolens      GID..SD.......DKRSEICRKIVEACEDWGIFQVVDHGVDSDLISEMTRLAREFFALP
AAX21539.1_F3H_Ammi_majus              GIDDDSD.......DKRSQICRKIVEACEGWGIFQVVDHGIDTDLISEMTRLAREFFALP
AAU04791.1_F3H_Fragaria_x_ananassa     GIE.EVE.......GRRAEICKKIVEACEDWGVFQIVDHGIDPKLISEMTRLAREFFALP
AAU93347.1_F3H_Ginkgo_biloba           GME.......G..EERGRVREEVRAACEEWGIFQVLHHGVPSDLVHRMSQLSRSFFALP
AAT94365.1_F3H_Glycine_max             GID.EVD.......GRRREICEKIVEACENWGIFQVVDHGVDQQLVAEMTRLAKEFFALP
AAX21535.1_F3H_Pimpinella_anisum       GID...D.......DKRSQICRKIVEACEGWGIFQVVDHGIDTDLISEMTRLAREFFALP
AAP57394.1_F3H_Petroselinum_crispum    GIDDDSV.......DKRSQICRKIVEACEDWGIFQVVDHGIDIDLISEMTRLARQFFALP
AAC49929.1_F3H_Petunia_x_hybrida       GID.DET.......GKRAEICDKIVKACEDWGVFQVVDHGVDAELISQMTTFAKEFFALP
                                 acc
```

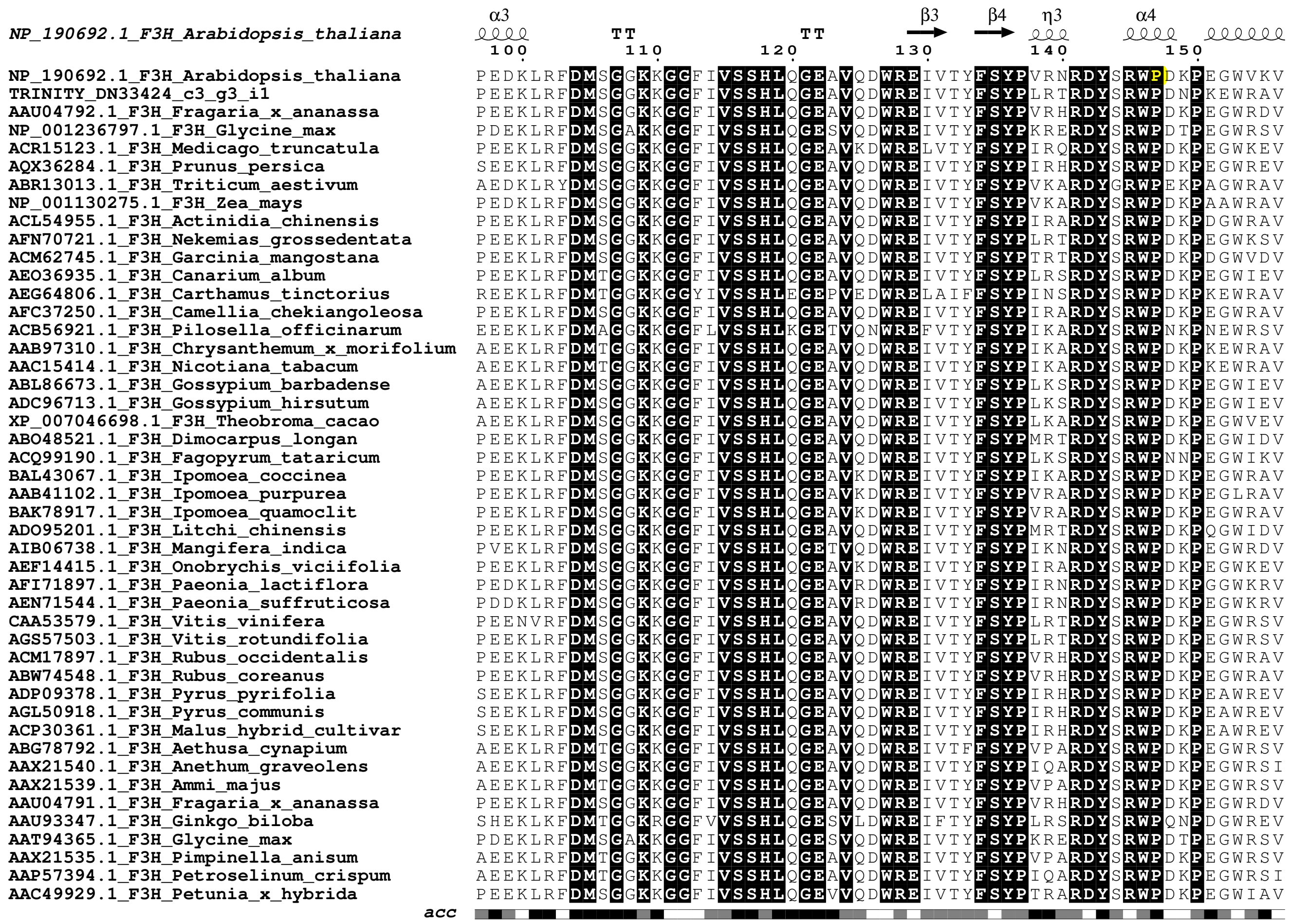
NP_190692.1_F3H_Arabidopsis_thaliana
α3
TT
TT
β3
β4
η3
α4
100 110 120 130 140 150
NP_190692.1_F3H_Arabidopsis_thaliana PEDKLRFDMSGGKKGGFIVSSHLQGEAVQDWREIVTYFSYPVRNRDYSRWPDKPEGWVKV
TRINITY_DN33424_c3_g3_i1 PEEKLRFDMSGGKKGGFIVSSHLQGEAVQDWREIVTYFSYPLRTRDYSRWPDNPKEWRAV
AAU04792.1_F3H_Fragaria_x_ananassa PEEKLRFDMSGGKKGGFIVSSHLQGEAVQDWREIVTYFSYPVRHRDYSRWPDKPEGWRDV
NP_001236797.1_F3H_Glycine_max PDEKLRFDMSGAKKGGFIVSSHLQGESVQDWREIVTYFSYPKRERDYSRWPDTPEGWRSV
ACR15123.1_F3H_Medicago_truncatula PEEKLRFDMSGGKKGGFIVSSHLQGEAVKDWRELVTYFSYPIRQRDYSRWPDKPEGWKEV
AQX36284.1_F3H_Prunus_persica SEEKLRFDMSGGKKGGFIVSSHLQGEAVQDWREIVTYFSYPIRHRDYSRWPDKPEGWREV
ABR13013.1_F3H_Triticum_aestivum AEDKLRYDMSGGKKGGFIVSSHLQGEAVQDWREIVTYFSYPVKARDYGRWPEKPAGWRAV
NP_001130275.1_F3H_Zea_mays PEDKLRFDMSGGKKGGFIVSSHLQGEAVQDWREIVTYFSYPVKARDYSRWPDKPAAWRAV
ACL54955.1_F3H_Actinidia_chinensis PEEKLRFDMSGGKKGGFIVSSHLQGEAVQDWREIVTYFSYPIRARDYSRWPDKPDGWRAV
AFN70721.1_F3H_Nekemias_grossedentata PEEKLRFDMSGGKKGGFIVSSHLQGEAVQDWREIVTYFSYPLRTRDYSRWPDKPEGWKSV
ACM62745.1_F3H_Garcinia_mangostana PEEKLRFDMSGGKKGGFIVSSHLQGEAVQDWREIVTYFSYPTRTRDYSRWPDKPDGWVDV
AEO36935.1_F3H_Canarium_album PEEKLRFDMTGGKKGGFIVSSHLQGEAVQDWREIVTYFSYPLRSRDYSRWPDKPEGWIEV
AEG64806.1_F3H_Carthamus_tinctorius REEKLRFDMTGGKKGGYIVSSHLEGEPVEDWRELAIFFSYPINSRDYSRWPDKPKEWRAV
AFC37250.1_F3H_Camellia_chekiangoleosa PEEKLRFDMSGGKKGGFIVSSHLQGEAVQDWREIVTYFSYPIRARDYSRWPDKPEGWRAV
ACB56921.1_F3H_Pilosella_officinarum EEEKLKFDMAGGKKGGFLVSSHLKGETVQNWREFVTYFSYPIKARDYSRWPNKPNEWRSV
AAB97310.1_F3H_Chrysanthemum_x_morifolium AEEKLRFDMTGGKKGGFIVSSHLQGEAVQDWREIVTYFSYPIKARDYSRWPDKPKEWRAV
AAC15414.1_F3H_Nicotiana_tabacum AEEKLRFDMTGGKKGGFIVSSHLQGEAVQDWREIVTYFSYPIKARDYSRWPDKPKEWRAV
ABL86673.1_F3H_Gossypium_barbadense AEEKLRFDMSGGKKGGFIVSSHLQGEAVQDWREIVTYFSYPLKSRDYSRWPDKPEGWIEV
ADC96713.1_F3H_Gossypium_hirsutum AEEKLRFDMSGGKKGGFIVSSHLQGEAVQDWREIVTYFSYPLKSRDYSRWPDKPEGWIEV
XP_007046698.1_F3H_Theobroma_cacao AEEKLRFDMSGGKKGGFIVSSHLQGEAVQDWREIVTYFSYPLKARDYSRWPDKPEGWVEV
ABO48521.1_F3H_Dimocarpus_longan PEEKLRFDMSGGKKGGFIVSSHLQGEAVQDWREIVTYFSYPMRTRDYSRWPDKPEGWIDV
ACQ99190.1_F3H_Fagopyrum_tataricum PEEKLKFDMSGGKKGGFIVSSHLQGEAVQDWREIVTYFSYPLKSRDYSRWPNNPEGWIKV
BAL43067.1_F3H_Ipomoea_coccinea PEEKLRFDMSGGKKGGFIVSSHLQGEAVKDWREIVTYFSYPIKARDYSRWPDKPEGWRAV
AAB41102.1_F3H_Ipomoea_purpurea PEEKLRFDMSGGKKGGFIVSSHLQGEAVKDWREIVTYFSYPVRARDYSRWPDKPEGLRAV
BAK78917.1_F3H_Ipomoea_quamoclit PEEKLRFDMSGGKKGGFIVSSHLQGEAVKDWREIVTYFSYPVRARDYSRWPDKPEGWRAV
ADO95201.1_F3H_Litchi_chinensis PEEKLRFDMSGGKKGGFIVSSHLQGEAVQDWREIVTYFSYPMRTRDYSRWPDKPQGWIDV
AIB06738.1_F3H_Mangifera_indica PVEKLRFDMSGGKKGGFIVSSHLQGETVQDWREIVTYFSYPIKNRDYSRWPDKPEGWRDV
AEF14415.1_F3H_Onobrychis_viciifolia PEEKLRFDMSGGKKGGFIVSSHLQGEAVKDWREIVTYFSYPIRARDYSRWPDKPEGWKEV
AFI71897.1_F3H_Paeonia_lactiflora PEDKLRFDMSGGKKGGFIVSSHLQGEAVRDWREIVTYFSYPIRNRDYSRWPDKPGGWKRV
AEN71544.1_F3H_Paeonia_suffruticosa PDDKLRFDMSGGKKGGFIVSSHLQGEAVRDWREIVTYFSYPIRNRDYSRWPDKPEGWKRV
CAA53579.1_F3H_Vitis_vinifera PEENVRFDMSGGKKGGFIVSSHLQGEAVQDWREIVTYFSYPLRTRDYSRWPDKPEGWRSV
AGS57503.1_F3H_Vitis_rotundifolia PEEKLRFDMSGGKKGGFIVSSHLQGEAVQDWREIVTYFSYPLRTRDYSRWPDKPEGWRSV
ACM17897.1_F3H_Rubus_occidentalis PEEKLRFDMSGGKKGGFIVSSHLQGEAVQDWREIVTYFSYPVRHRDYSRWPDKPEGWRAV
ABW74548.1_F3H_Rubus_coreanus PEEKLRFDMSGGKKGGFIVSSHLQGEAVQDWREIVTYFSYPVRHRDYSRWPDKPEGWRAV
ADP09378.1_F3H_Pyrus_pyrifolia SEEKLRFDMSGGKKGGFIVSSHLQGEAVQDWREIVTYFSYPIRHRDYSRWPDKPEAWREV
AGL50918.1_F3H_Pyrus_communis SEEKLRFDMSGGKKGGFIVSSHLQGEAVQDWREIVTYFSYPIRHRDYSRWPDKPEAWREV
ACP30361.1_F3H_Malus_hybrid_cultivar SEEKLRFDMSGGKKGGFIVSSHLQGEAVQDWREIVTYFSYPIRHRDYSRWPDKPEAWREV
ABG78792.1_F3H_Aethusa_cynapium AEEKLRFDMTGGKKGGFIVSSHLQGEAVQDWREIVTFFSYPVPARDYSRWPDKPEGWRSV
AAX21540.1_F3H_Anethum_graveolens AEEKLRFDMSGGKKGGFIVSSHLQGEAVQDWREIVTYFSYPIQARDYSRWPDKPEGWRSI
AAX21539.1_F3H_Ammi_majus AEEKLRFDMTGGKKGGFIVSSHLQGEAVQDWREIVTYFSYPVPARDYSRWPDKPEGWRSV
AAU04791.1_F3H_Fragaria_x_ananassa PEEKLRFDMSGGKKGGFIVSSHLQGEAVQDWREIVTYFSYPVRHRDYSRWPDKPEGWRDV
AAU93347.1_F3H_Ginkgo_biloba SHEKLKFDMTGGKRGGFVVSSHLQGESVLDWREIFTYFSYPLRSRDYSRWPQNPDGWREV
AAT94365.1_F3H_Glycine_max PDEKLRFDMSGAKKGGFIVSSHLQGESVQDWREIVTYFSYPKRERDYSRWPDTPEGWRSV
AAX21535.1_F3H_Pimpinella_anisum AEEKLRFDMTGGKKGGFIVSSHLQGEAVQDWREIVTYFSYPVPARDYSRWPDKPEGWRSV
AAP57394.1_F3H_Petroselinum_crispum AEEKLRFDMTGGKKGGFIVSSHLQGEAVQDWREIVTYFSYPIQARDYSRWPDKPEGWRSI
AAC49929.1_F3H_Petunia_x_hybrida PEEKLRFDMSGGKKGGFIVSSHLQGEVVQDWREIVTYFSYPTRARDYSRWPDKPEGWIAV
acc

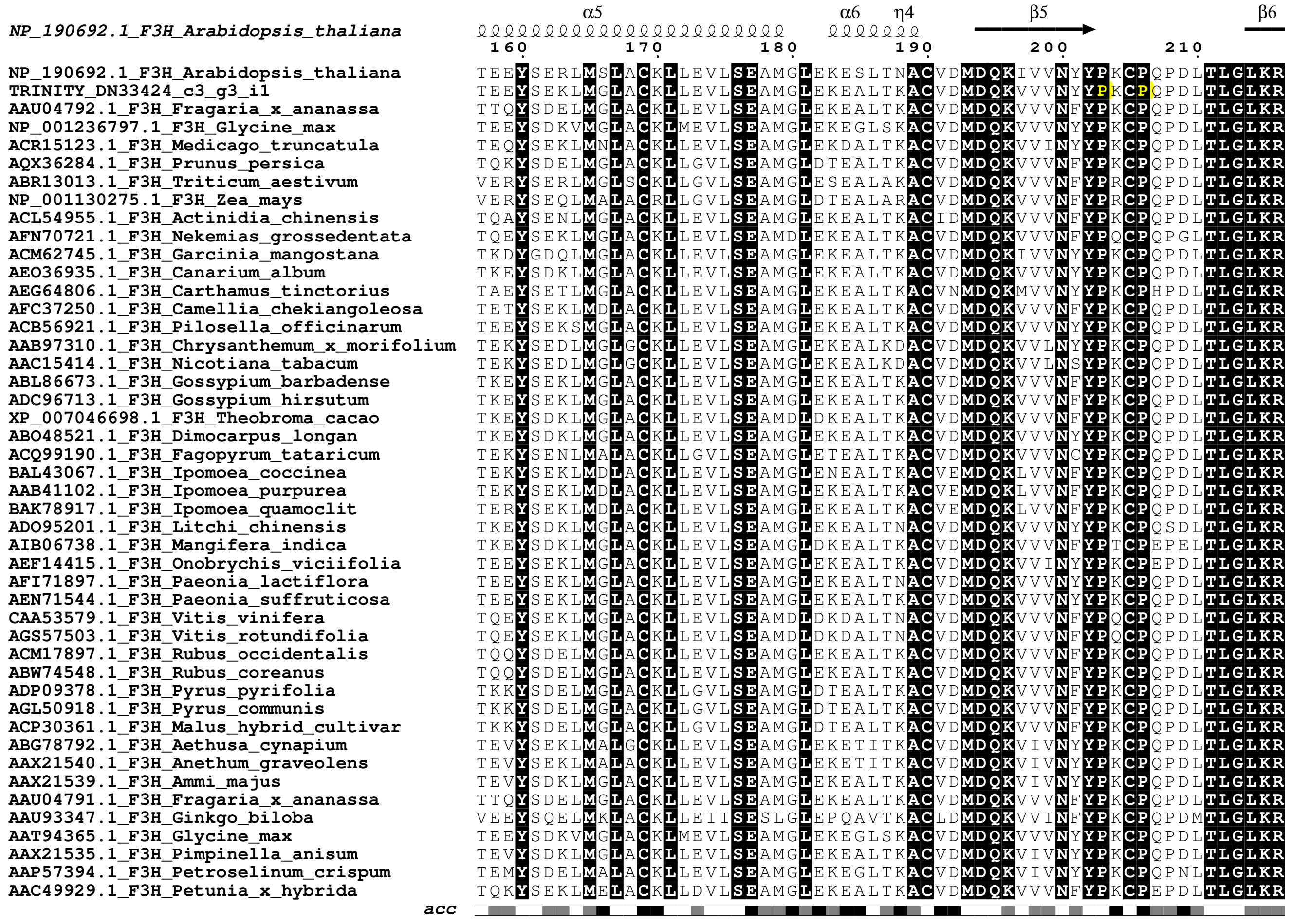
NP_190692.1_F3H_Arabidopsis_thaliana
α5
α6
η4
β5
β6
160 170 180 190 200 210
NP_190692.1_F3H_Arabidopsis_thaliana TEEYSERLMSLACKLLEVLSEAMGLEKESLTNACVDMDQKIVVNYYPKCPQPDLTLGLKR
TRINITY_DN33424_c3_g3_i1 TEEYSEKLMGLACKLLEVLSEAMGLEKEALTKACVDMDQKVVVNYYPKCPQPDLTLGLKR
AAU04792.1_F3H_Fragaria_x_ananassa TTQYSDELMGLACKLLEVLSEAMGLEKEALTKACVDMDQKVVVNFYPKCPQPDLTLGLKR
NP_001236797.1_F3H_Glycine_max TEEYSDKVMGLACKLMEVLSEAMGLEKEGLSKACVDMDQKVVVNYYPKCPQPDLTLGLKR
ACR15123.1_F3H_Medicago_truncatula TEQYSEKLMNLACKLLEVLSEAMGLEKDALTKACVDMDQKVVINYYPKCPQPDLTLGLKR
AQX36284.1_F3H_Prunus_persica TQKYSDELMGLACKLLGVLSEAMGLDTEALTKACVDMDQKVVVNFYPKCPQPDLTLGLKR
ABR13013.1_F3H_Triticum_aestivum VERYSERLMGLSCKLLGVLSEAMGLESEALAKACVDMDQKVVVNFYPRCPQPDLTLGLKR
NP_001130275.1_F3H_Zea_mays VERYSEQLMALACRLLGVLSEAMGLDTEALARACVDMDQKVVVNFYPRCPQPDLTLGLKR
ACL54955.1_F3H_Actinidia_chinensis TQAYSENLMGLACKLLEVLSEAMGLEKEALTKACIDMDQKVVVNFYPKCPQPDLTLGLKR
AFN70721.1_F3H_Nekemias_grossedentata TQEYSEKLMGLACKLLEVLSEAMDLEKEALTKACVDMDQKVVVNFYPQCPQPGLTLGLKR
ACM62745.1_F3H_Garcinia_mangostana TKDYGDQLMGLACKLLEVLSEAMGLEKEALTKACVDMDQKIVVNYYPKCPQPDLTLGLKR
AEO36935.1_F3H_Canarium_album TKEYSDKLMGLACKLLEVLSEAMGLEKEALTKACVDMDQKVVVNYYPKCPQPDLTLGLKR
AEG64806.1_F3H_Carthamus_tinctorius TAEYSETLMGLACKLLEVLSEAMGLEKEALTKACVNMDQKMVVNYYPKCPHPDLTLGLKR
AFC37250.1_F3H_Camellia_chekiangoleosa TETYSEKLMDLACKLLEVLSEAMGLEKEALTKACVDMDQKVVVNFYPKCPQPDLTLGLKR
ACB56921.1_F3H_Pilosella_officinarum TEEYSEKSMGLACKLLEVLSEAMGLEKEALTKACVDMDQKVVVNYYPKCPQPDLTLGLKR
AAB97310.1_F3H_Chrysanthemum_x_morifolium TEKYSEDLMGLGCKLLEVLSEAMGLEKEALKDACVDMDQKVVLNYYPKCPQPDLTLGLKR
AAC15414.1_F3H_Nicotiana_tabacum TEKYSEDLMGLGCKLLEVLSEAMGLEKEALKDACVDMDQKVVLNSYPKCPQPDLTLGLKR
ABL86673.1_F3H_Gossypium_barbadense TKEYSEKLMGLACKLLEVLSEAMGLEKEALTKACVDMDQKVVVNFYPKCPQPDLTLGLKR
ADC96713.1_F3H_Gossypium_hirsutum TKEYSEKLMGLACKLLEVLSEAMGLEKEALTKACVDMDQKVVVNFYPKCPQPDLTLGLKR
XP_007046698.1_F3H_Theobroma_cacao TKEYSDKLMGLACKLLEVLSEAMDLDKEALTKACVDMDQKVVVNFYPKCPQPDLTLGLKR
ABO48521.1_F3H_Dimocarpus_longan TKEYSDKLMGLACKLLEVLSEAMGLEKEALTKACVDMDQKVVVNYYPKCPQPDLTLGLKR
ACQ99190.1_F3H_Fagopyrum_tataricum TEKYSENLMALACKLLGVLSEAMGLETEALTKACVDMDQKVVVNCYPKCPQPDLTLGLKR
BAL43067.1_F3H_Ipomoea_coccinea TEKYSEKLMDLACKLLEVLSEAMGLENEALTKACVEMDQKLVVNFYPKCPQPDLTLGLKR
AAB41102.1_F3H_Ipomoea_purpurea TEKYSEKLMDLACKLLEVLSEAMGLEKEALTKACVEMDQKLVVNFYPKCPQPDLTLGLKR
BAK78917.1_F3H_Ipomoea_quamoclit TERYSEKLMDLACKLLEVLSEAMGLEKEALTKACVEMDQKLVVNFYPKCPQPDLTLGLKR
ADO95201.1_F3H_Litchi_chinensis TKEYSDKLMGLACKLLEVLSEAMGLEKEALTNACVDMDQKVVVNYYPKCPQSDLTLGLKR
AIB06738.1_F3H_Mangifera_indica TKEYSDKLMGLACKLLEVLSEAMGLDKEALTKACVDMDQKVVVNFYPTCPEPELTLGLKR
AEF14415.1_F3H_Onobrychis_viciifolia TEEYSEKLMGLACKLLEVLSEAMGLEKEALTKACVDMDQKVVINYYPKCPEPDLTLGLKR
AFI71897.1_F3H_Paeonia_lactiflora TEEYSEKLMGLACKLLEVLSEAMGLEKEALTNACVDMDQKVVVNYYPKCPQPDLTLGLKR
AEN71544.1_F3H_Paeonia_suffruticosa TEEYSEKLMGLACKLLEVLSEAMGLEKEALTKACVDMDQKVVVNYYPKCPQPDLTLGLKR
CAA53579.1_F3H_Vitis_vinifera TQEYSEKLMGLACKLLEVLSEAMDLDKDALTNACVDMDQKVVVNFYPQCPQPDLTLGLKR
AGS57503.1_F3H_Vitis_rotundifolia TQEYSEKLMGLACKLLEVLSEAMDLDKDALTNACVDMDQKVVVNFYPQCPQPDLTLGLKR
ACM17897.1_F3H_Rubus_occidentalis TQQYSDELMGLACKLLEVLSEAMGLEKEALTKACVDMDQKVVVNFYPKCPQPDLTLGLKR
ABW74548.1_F3H_Rubus_coreanus TQQYSDELMGLACKLLEVLSEAMGLEKEALTKACVDMDQKVVVNFYPKCPQPDLTLGLKR
ADP09378.1_F3H_Pyrus_pyrifolia TKKYSDELMGLACKLLGVLSEAMGLDTEALTKACVDMDQKVVVNFYPKCPQPDLTLGLKR
AGL50918.1_F3H_Pyrus_communis TKKYSDELMGLACKLLGVLSEAMGLDTEALTKACVDMDQKVVVNFYPKCPQPDLTLGLKR
ACP30361.1_F3H_Malus_hybrid_cultivar TKKYSDELMGLACKLLGVLSEAMGLDTEALTKACVDMDQKVVVNFYPKCPQPDLTLGLKR
ABG78792.1_F3H_Aethusa_cynapium TEVYSEKLMALGCKLLEVLSEAMGLEKETITKACVDMDQKVIVNYYPKCPQPDLTLGLKR
AAX21540.1_F3H_Anethum_graveolens TEVYSEKLMALACKLLEVLSEAMGLEKETITKACVDMDQKVIVNYYPKCPQPDLTLGLKR
AAX21539.1_F3H_Ammi_majus TEVYSDKLMGLACKLLEVLSEAMGLEKEALTKACVDMDQKVIVNYYPKCPQPDLTLGLKR
AAU04791.1_F3H_Fragaria_x_ananassa TTQYSDELMGLACKLLEVLSEAMGLEKEALTKACVDMDQKVVVNFYPKCPQPDLTLGLKR
AAU93347.1_F3H_Ginkgo_biloba VEEYSQELMKLACKLLEIISESLGLEPQAVTKACLDMDQKVVINFYPKCPQPDMTLGLKR
AAT94365.1_F3H_Glycine_max TEEYSDKVMGLACKLMEVLSEAMGLEKEGLSKACVDMDQKVVVNYYPKCPQPDLTLGLKR
AAX21535.1_F3H_Pimpinella_anisum TEVYSDKLMGLACKLLEVLSEAMGLEKEALTKACVDMDQKVIVNYYPKCPQPDLTLGLKR
AAP57394.1_F3H_Petroselinum_crispum TEMYSDELMALACKLLEVLSEAMGLEKEGLTKACVDMDQKVIVNYYPKCPQPNLTLGLKR
AAC49929.1_F3H_Petunia_x_hybrida TQKYSEKLMELACKLLDVLSEAMGLEKEALTKACVDMDQKVVVNFYPKCPEPDLTLGLKR
acc

```
                                          β7         β8        β9         β10    α7            β11
NP_190692.1_F3H_Arabidopsis_thaliana
                                   220       230       240       250       260       270
NP_190692.1_F3H_Arabidopsis_thaliana      HTDPGTITLLLQDQVGGLQATRDNGKTWITVQPVEGAFVVNLGDHGHFLSNGRFKNADHQ
TRINITY_DN33424_c3_g3_i1                  HTDPGTITLLLQDQVGGLQATRDNGKTWITVQPVEGAFVVNLGDHGHFLSNGRFRNADHQ
AAU04792.1_F3H_Fragaria_x_ananassa        HTDPGTITLLLQDQVGGLQATRDGGKTWITVQPVEGAFVVNLGDHGHFLSNGRFKNADHQ
NP_001236797.1_F3H_Glycine_max            HTDPGTITLLLQDQVGGLQATRDNGKTWITVQPVEAAFVVNLGDHAHYLSNGRFKNADHQ
ACR15123.1_F3H_Medicago_truncatula        HTDPGTITLLLQDQVGGLQATKDNGKTWITVQPVEGAFVVNLGDHGHYLSNGRFKNADHQ
AQX36284.1_F3H_Prunus_persica             HTDPGTITLLLQDQVGGLQATRDDGKTWITVQPVEGAFVVNLGDHGHFLSNGRFKNADHQ
ABR13013.1_F3H_Triticum_aestivum          HTDPGTITLLLQDLVGGLQATRDGGKTWITVQPISGAFVVNLGDHGHFMSNGRFKNADHQ
NP_001130275.1_F3H_Zea_mays               HTDPGTITLLLQDLVGGLQATRDGGRTWITVQPVEGAFVVNLGDHGHLLSNGRFKNADHQ
ACL54955.1_F3H_Actinidia_chinensis        HTDPGTITLLLQDQVGGLQATRDGGKTWITVQPVDGAFVVNLGDHGHYLSNGRFKNADHQ
AFN70721.1_F3H_Nekemias_grossedentata     HTDPGTITLLLQDQVGGLQATRDGGKTWITVQPVEGAFVVNLGDHGHYLSNGRFKNADHQ
ACM62745.1_F3H_Garcinia_mangostana        HTDPGTITLLLQDQVGGLQATRDNGETWITVQPIEGAFVVNLGDHGHFLSNGRFRNADHQ
AEO36935.1_F3H_Canarium_album             HTDPGTITLLLQDQVGGLQATRDDGKTWITVQPVEEAFVVNLGDHGHHLSNGRFRNADHQ
AEG64806.1_F3H_Carthamus_tinctorius       HTDAGTITLLLQDQVGGLQATRDGGESWITVQPIEGAFVVNLGDHGHYLSNGRFKNADHQ
AFC37250.1_F3H_Camellia_chekiangoleosa    HTDPGTITLLLQDQVGGLQATRDGGKTWITVQPVEGAFVVNLGDHGHYLSNGRFKNADHQ
ACB56921.1_F3H_Pilosella_officinarum      HTDPGTITLVLQDLVGGLQATRDGGKSWITVEPIEGAFVVNLGDHGHYLSNGRFKNADHQ
AAB97310.1_F3H_Chrysanthemum_x_morifolium HTDPGTITLLLQDQVGGLQATRDGGKTWITVEPIEGAFVVNLGDHGHYLSNGRFKNADHQ
AAC15414.1_F3H_Nicotiana_tabacum          HTDPGTITLLLQDQVGGLQATRDGGKTWITVEPIEGAFVVNLGDHGHYLSNGRFKNADHQ
ABL86673.1_F3H_Gossypium_barbadense       HTDPGTITLLLQDQVGGLQATRDNGKTWITVQPVEGAFVVNLGDHGHYLSNGRFKNADHQ
ADC96713.1_F3H_Gossypium_hirsutum         HTDPGTITLLLQDQVGGLQATRDNGKTWITVQPVEGAFVVNLGDHGHYLSNGRFKNADHQ
XP_007046698.1_F3H_Theobroma_cacao        HTDPGTITLLLQDQVGGLQATRDNGKTWITVQPVEGAFVVNLGDHGHFLSNGRFKNADHQ
ABO48521.1_F3H_Dimocarpus_longan          HTDPGTITLLLQDQVGGLQATRDNGKTWITVQPVDGAFVVNLGDHGHYLSNGRFKNADHQ
ACQ99190.1_F3H_Fagopyrum_tataricum        HTDPGTITLLLQDQVGGLQATRDDGKTWITVQPIEGAFVVNLGDHGHYLSNGRFRNADHQ
BAL43067.1_F3H_Ipomoea_coccinea           HTDPGTITLLLQDQVGGLQATKDGGKTWITVQPVDGAFVVNLGDHGHFLSNGRFKNADHQ
AAB41102.1_F3H_Ipomoea_purpurea           HTDPGTITLLLQDQVGGLQATKDGGKTWITVQPVDGAFVVNLGDHGHFLSNGRFKNADHQ
BAK78917.1_F3H_Ipomoea_quamoclit          HTDPGTITLLLQDQVGGLQATKDGGKTWITVQPVDGAFVVNLGDHGHFLSNGRFKNADHQ
ADO95201.1_F3H_Litchi_chinensis           HTDPGTITLLLQDQVGGLQATRDNGKTWITVQPVDGAFVVNLGDHGHYLSNGRFKNADHQ
AIB06738.1_F3H_Mangifera_indica           HTDPGTITLLLQDQVGGLQATRDNGETWITVQPVDGAFVVNLGDHGHYLSNGRFKNADHQ
AEF14415.1_F3H_Onobrychis_viciifolia      HTDPGTITLLLQDQVGGLQATKDNGKTWITVQPIEGAFVVNLGDHGHFLSNGRFKNADHQ
AFI71897.1_F3H_Paeonia_lactiflora         HTDPGTITLLLQDQVGGLQATRDGGKTWITVQPVEGAFVVNLGDHGHYLSNGRFKNADHQ
AEN71544.1_F3H_Paeonia_suffruticosa       HTDPGTITLLLQDQVGGLQATRDGGKTWITVQPVEGAFVVNLGDHGHYLSNGRFKNADHQ
CAA53579.1_F3H_Vitis_vinifera             HTDPGTITLLLQDQVGGLQATRDGGKTWITVQPVEGAFVVNLGDHGHYLSNGRFKNADHQ
AGS57503.1_F3H_Vitis_rotundifolia         HTDPGTITLLLQDQVGGLQATRDGGKTWITVQPVEGAFVVNLGDHGHYLSNGRFKNADHQ
ACM17897.1_F3H_Rubus_occidentalis         HTDPGTITLLLQDQVGGLQATRDGGKTWITVQPVEGAFVVNLGDHGHFLSNGRFKNADHQ
ABW74548.1_F3H_Rubus_coreanus             HTDPGTITLLLQDQVGGLQATRDGGKTWITGQPVEGAFVVNLGDHGHFLSNGRFKNADHQ
ADP09378.1_F3H_Pyrus_pyrifolia            HTDPGTITLLLQDQVGGLQATRDDGKTWITVQPVEGAFVVNLGDHGHFLSNGRFKNADHQ
AGL50918.1_F3H_Pyrus_communis             HTDPGTITLLLQDQVGGLQATRDDGKTWITVQPVEGAFVVNLGDHGHFLSNGRFKNADHQ
ACP30361.1_F3H_Malus_hybrid_cultivar      HTDPGTITLLLQDQVGGLQATRDDGKTWITVQPVEGAFVVNLGDHGHLLSNGRFKNADHQ
ABG78792.1_F3H_Aethusa_cynapium           HTDPGTITLLLQDQVGGLQATRDGGKTWITVQPVEGAFVVNLGDHGHYLSNGRFKNADHQ
AAX21540.1_F3H_Anethum_graveolens         HTDPGTITLLLQDQVGGLQATRDGGKTWITVQPVEGAFVVNLGDHGHYLSNGRFKNADHQ
AAX21539.1_F3H_Ammi_majus                 HTDPGTITLLLQDQVGGLQATRDGGKTWITVQPVEGAFVVNLGDHGHYLSNGRFKNADHQ
AAU04791.1_F3H_Fragaria_x_ananassa        HTDPGTITLLLQDQVGGLQATRDGGKTWITVQPVEGAFVVNLGDHGHFLSNGRFKNADHQ
AAU93347.1_F3H_Ginkgo_biloba              HTDPGTITLLLQDQVGGLQATKD.GLNWITVEPVEGAFVVNLGDHMHYLSNGKYKNADHQ
AAT94365.1_F3H_Glycine_max                HTDPGTITLLLQDQVGGLQATRDNGKTWITVQPVEAAFVVNLGDHAHYLSNGRFKNADHQ
AAX21535.1_F3H_Pimpinella_anisum          HTDPGTITLLLQDQVGGLQATRDGGKTWITVQPVEGAFVVNLGDHGHYLSNGRFKNADHQ
AAP57394.1_F3H_Petroselinum_crispum       HTDPGTITLLLQDQVGGLQATRDGGKTWITVQPVEGAFVVNLGDHGHYLSNGRFKNADHQ
AAC49929.1_F3H_Petunia_x_hybrida          HTDPGTITLLLQDQVGGLQATKDNGKTWITVQPVEGAFVVNLGDHGHFLSNGRFKNADHQ
                                      acc
```

```
                                                  β12                            α8
NP_190692.1_F3H_Arabidopsis_thaliana                                   TT
                                   280       290       300       310       320       330
NP_190692.1_F3H_Arabidopsis_thaliana      AVVNSNSSRLSIATFQNPAPDATVYPLK..VREGEKAILEEPITFAEMYKRKMGRDLELA
TRINITY_DN33424_c3_g3_i1                  AVVNSNCSRLSIATFQNPAPEATVYPLK..IEEGVKPVLDEPMTFAEMYRRKMSKDLELA
AAU04792.1_F3H_Fragaria_x_ananassa        AVVNSNHSRLSIATFQNPAQEAIVYPLK..VREGEKPILEEPMTYTEMYKKKMSKDLELA
NP_001236797.1_F3H_Glycine_max            AVVNSNHSRLSIATFQNPAPNATVYPLK..IREGEKPVMEEPITFAEMYRRKMSKDIEIA
ACR15123.1_F3H_Medicago_truncatula        AVVNSNYSRLSIATFQNPAPDATVYPLK..IRDGEKSVMEEPITFAEMYRRKMSKDLEIA
AQX36284.1_F3H_Prunus_persica             AVVNSNSSRLSIATFQNPAQEATVYPLS..IREGEKPILEGPITYTEMYKKKMTKDLELA
ABR13013.1_F3H_Triticum_aestivum          AVVNGESSRLSIATFQNPAPDARVWPLA..VREGEEPILEEPITFAEMYRRKMERDLDLA
NP_001130275.1_F3H_Zea_mays               AVVNSECSRLSIATFQNPAPDATVYPLA..VRDGEAPILDHPITFAEMYRRKMARDIELA
ACL54955.1_F3H_Actinidia_chinensis        AVVNSNCSRLSIATFQNPAPEATVYPLA..IQEGEKPVLEEPITFAEMYRRKMSKDLELA
AFN70721.1_F3H_Nekemias_grossedentata     AVVNSNYSRLSIATFQNPAPEATVYPLK..IREGEKAVLEEPITFAEMYRRKMSKDLELA
ACM62745.1_F3H_Garcinia_mangostana        AVVNSNCSRLSIATFQNPAPDAIVYPLK..IREGEKSILEEPITFSEMYRRKMAKDLELA
AEO36935.1_F3H_Canarium_album             AVVNSDCSRLSIATFQNPAPNATVYPLK..IREGEKPVLEEPITFAEMYRRKMSRDLELA
AEG64806.1_F3H_Carthamus_tinctorius       ARVNSNTNRLSIATFQNPAPDAIVYPLK..VNEGDRSIMEKPITSTEMYERKMSKDLELA
AFC37250.1_F3H_Camellia_chekiangoleosa    AVVNSNCSRLSIATFQNPAPEATVYPLK..IREGEKPVLEEPITFADMYKRKMSKDIELA
ACB56921.1_F3H_Pilosella_officinarum      AVVNSSTSRLSIATFQNPAPEAIVYPLK..INEGEKSIMEEAMTFMDMYKKKMSGDLELA
AAB97310.1_F3H_Chrysanthemum_x_morifolium AVVNSNTSRLSIATFQNPAPNAIVYPLI..VNEGEKSIMEEAITFMDMYKKKMSTDLELA
AAC15414.1_F3H_Nicotiana_tabacum          AVVNSNTSRLSIATFQNPAPNAIVYPLI..VNEGEKSIMEEAITFMDMYKKKMSTDLELA
ABL86673.1_F3H_Gossypium_barbadense       AVVNSNCSRLSIATFQNPAPDATVYPLK..IREGEKPILEEPITFAEMYRRKMSKDLELA
ADC96713.1_F3H_Gossypium_hirsutum         AVVNSNCSRLSIATFQNPAPDATVYPLK..IREGEKPILEEPITFAEMYRRKMSKDLELA
XP_007046698.1_F3H_Theobroma_cacao        AVVNSDCSRLSIATFQNPAPDATVYPLR..IREGEQPILEEPITFAEMYRRKMSKDLELA
ABO48521.1_F3H_Dimocarpus_longan          AVVDSNCSRLSIATFQNPAPEATVYPLK..IREGEKPILEEPITFAEMYRRKMSKDLELA
ACQ99190.1_F3H_Fagopyrum_tataricum        AVVNSNSSRLSIATFQNPAPEATVYPLA..IREGEKPILEEPITFAEMYRRKMSKDLELA
BAL43067.1_F3H_Ipomoea_coccinea           AVVNSERSRVSIATFQNPAPEAKVYPLK..VREGEKPILEEPITFAEMYRRKMSKDLELA
AAB41102.1_F3H_Ipomoea_purpurea           AVVNSEHSRMSIATFQNPAPEAKVYPLK..VREGEKAILEEPITFAEMYRRKMSKDLELA
BAK78917.1_F3H_Ipomoea_quamoclit          AVVNSERSRVSIATFQNPAPEAKVYPLT..VREGEKPILEEPITFAEMYRRKMSKDLELA
ADO95201.1_F3H_Litchi_chinensis           AVVNSNCSRLSIATFQNPAPEATVYPLK..IREGEKPILEQPITFAEMYRRKMSKDLELA
AIB06738.1_F3H_Mangifera_indica           AVVNSNCSRLSIATFQNPAPEATVYPLK..IREGEEAILEEPITFAEMYRRKMSKDLELA
AEF14415.1_F3H_Onobrychis_viciifolia      AVVNSNYSRLSIATFQNPAPDATVYPLK..VREGEKSVMEEPITFAEMYRRKMSKDLEIA
AFI71897.1_F3H_Paeonia_lactiflora         AVVNSNYSRLSIATFQNPAPEAIVYPLK..IRDGEKSVLEKPITFTEMYRTKMSKDIELA
AEN71544.1_F3H_Paeonia_suffruticosa       AVVNSNYSRLSIATFQNPAPEAIVYPLK..IRDGEKPVLEKPITFTEMYRTKMSKDIELA
CAA53579.1_F3H_Vitis_vinifera             AVVNSNHSRLSIATFQNPAPEATVYPLK..IREGEKAVLEGPITFAEMYRRKMSKDLELA
AGS57503.1_F3H_Vitis_rotundifolia         AVVNSNHSRLSIATFQNPAPEATVYPLK..IREGEKAVLEGPITFAEMYRRKMSKDLELA
ACM17897.1_F3H_Rubus_occidentalis         AVVNSNHSRLSIATFQNPAQEAIVYPLK..VREGEKPILEEPITYTEMYKKKMSKDLELA
ABW74548.1_F3H_Rubus_coreanus             AVVNSNHSRLSIATFQNPAQEAIVYPLK..VREGEKPILEEPITYTEMYKKKMSKDLELA
ADP09378.1_F3H_Pyrus_pyrifolia            AVVNSNSSRLSIATFQNPAQDAIVYPLS..VREGEKPILEAPITYTEMYKKKMSKDLEVA
AGL50918.1_F3H_Pyrus_communis             AVVNSNSSRLSIATFQNPAQEATVYPLS..VREGEKPILEAPITYTEMYKKKMSKDLELA
ACP30361.1_F3H_Malus_hybrid_cultivar      AVVNSNSSRLSIATFQNPAQEAIVYPLS..VREGEKPILEAPITYTEMYKKKMSKDLELA
ABG78792.1_F3H_Aethusa_cynapium           AVVNSDSSRMSIATFQNPAPNATVYPLK..IREGEKSVMEEAITFAEMYRRKMSKDIEVA
AAX21540.1_F3H_Anethum_graveolens         AVVNSNSSRMSIATFQNPAPNATVYPLK..IREGEKAVMEEPITFAEMYKRKMSKDIELA
AAX21539.1_F3H_Ammi_majus                 AVVNSNSSRMSIATFQNPAPNATVYPLK..IREGEKAVMEEPITFAEMYKRKMSKDIEMA
AAU04791.1_F3H_Fragaria_x_ananassa        AVVNSNHSRLSIATFQNPAQEAIVYPLK..VREGEKPILEEPMTYTEMYKKKMSKDLELA
AAU93347.1_F3H_Ginkgo_biloba              AVVNADTSRLSIATFQNPAQEAIVYPLEGVVKAEDKCVMEEPITFAQMYSHKMNRDLELA
AAT94365.1_F3H_Glycine_max                AVVNSNHSRLSIATFQNPAPNATVYPLK..IREGEKPVMEEPITFAEMYRRKMSKDIEIA
AAX21535.1_F3H_Pimpinella_anisum          AVVNSNSSRMSIATFQNPAPNATVYPLT..IREGEKAVMEEPITFAEMYKRKMSKDIEMA
AAP57394.1_F3H_Petroselinum_crispum       AVVNSNSSRMSIATFQNPAPNATVYPLK..IREGEKAVMEEPITFAEMYKRKMSRDIEMA
AAC49929.1_F3H_Petunia_x_hybrida          AVVNSNSSRLSIATFQNPAPEAIVYPLK..IREGEKSIMDEPITFAEMYRRKMSKDLELA
                                      acc
```

```
                                                             α9
NP_190692.1_F3H_Arabidopsis_thaliana
                                              340                  350
NP_190692.1_F3H_Arabidopsis_thaliana          RLKKLAK.EE..................RDHK.......EVDKP..VDQIFA......
TRINITY_DN33424_c3_g3_i1                      RLKKLAK.EQ....QAKDL.........EKSK.......LEGKP..IEEIFA......
AAU04792.1_F3H_Fragaria_x_ananassa            RLKKHAK.EQ....L.QDS.........EKTK.......LEAKP..VDDIFA......
NP_001236797.1_F3H_Glycine_max                RMKKLAK.EK....HLQDLENEKHLQELDQKAK.......LEAKP..LKEILA......
ACR15123.1_F3H_Medicago_truncatula            RMKKLAK.EEK...ELRDL.........EKAK.......IEAKP..LNEILA......
AQX36284.1_F3H_Prunus_persica                 RLKKLAK.EQ....QLQDS.........EK..........EGKP..KDDIFA......
ABR13013.1_F3H_Triticum_aestivum              KRKKQAK.DQL...MQQQLQLQ......QQQQAVAAAPMPTATKS..LNEILA......
NP_001130275.1_F3H_Zea_mays                   RLKKQVKADKS...ANKEF.........................ADSKP..LDAILA......
ACL54955.1_F3H_Actinidia_chinensis            RLKKLAK.ETQL..QEQEL.........EKAK.......LGAKG..VEEIFA......
AFN70721.1_F3H_Nekemias_grossedentata         RLKKLAK.EQ....QLQDL.........EKAK.......LESKP..IDEIFA......
ACM62745.1_F3H_Garcinia_mangostana            RLKKLAK.EQ....QLTDV.........EKAK.......LEAKP..IEKILA......
AEO36935.1_F3H_Canarium_album                 RLKKLAK.EK....QLQDL.........EKAK.......LEGKP..LEQILA......
AEG64806.1_F3H_Carthamus_tinctorius           RLKKLAK.EKQ.....QNL.........EKE...........KS..IEKISA......
AFC37250.1_F3H_Camellia_chekiangoleosa        KLKKLAK.EKKLLQDQQDI.........EKAK.......LEIKS..TDEIFA......
ACB56921.1_F3H_Pilosella_officinarum          RLKKLAK.DKK.....DNL.........EKV...........KP..TKNIFV......
AAB97310.1_F3H_Chrysanthemum_x_morifolium     RLKKLAK.AKQ.....QDL.........EKA...........KP..IESILA......
AAC15414.1_F3H_Nicotiana_tabacum              RLKKLAK.AKQ.....QDL.........EKA...........KP..IESILAKRLADF
ABL86673.1_F3H_Gossypium_barbadense           RLKKVAK.EQQ...QLKEKEAE.....NEKPK.......LEAKP..LEEILA......
ADC96713.1_F3H_Gossypium_hirsutum             RLKKVAK.EQQ...QLKEKEAE.....NEKPK.......LEAKP..LEEILA......
XP_007046698.1_F3H_Theobroma_cacao            RLKKLAK.EQQ...QVQEI.........EKTK.......LEAKP..LEKILA......
ABO48521.1_F3H_Dimocarpus_longan              RLKKLAK.EQKL..QLQDN.........EKSK.......LEAKP..IEQILA......
ACQ99190.1_F3H_Fagopyrum_tataricum            RLKKLAK.EQ....QSSDL.........QKAK.......LDSKP..IQDIFA......
BAL43067.1_F3H_Ipomoea_coccinea               RMKKFSK.EQK...QQMIIKAA......ADTN.......LETKP..IDQILA......
AAB41102.1_F3H_Ipomoea_purpurea               RIKKFAK.EQ....QMIKAAAA......ADTN.......LETKP..IDQILA......
BAK78917.1_F3H_Ipomoea_quamoclit              RIKKFAK.EQK...QMIKAAAA......ADTN.......LETKP..IDQILA......
ADO95201.1_F3H_Litchi_chinensis               RLKKLAK.EQ....QQQDT.........EKSK.......LEAKP..IEEILA......
AIB06738.1_F3H_Mangifera_indica               RLKKLAK.EQ....QLQES.........ETAK.......LDAQP..IEKILA......
AEF14415.1_F3H_Onobrychis_viciifolia          RMKKIAK..EK...ELRDL.........EKAK.......LEAKP..LNQILA......
AFI71897.1_F3H_Paeonia_lactiflora             RLKKLAK.E.....QMQDH....L....EKTQ.......LESKP..LEQILA......
AEN71544.1_F3H_Paeonia_suffruticosa           RLK...K.Q.....QMQDH....L....EKTQ.......LESKP..LDQILA......
CAA53579.1_F3H_Vitis_vinifera                 RLKKLAK.EQ....QLQDV.........EKAK.......LESKP..IDQILA......
AGS57503.1_F3H_Vitis_rotundifolia             RLKKLAK.EL....QLQDV.........EKAK.......LESKP..IDQILA......
ACM17897.1_F3H_Rubus_occidentalis             RLKKLAK.EQ....QPEDS.........EKAK.......LEVKQ..VDDIFA......
ABW74548.1_F3H_Rubus_coreanus                 RLKKLAK.EQ....QPEDS.........EKAK.......LEVKQ..VDDIFA......
ADP09378.1_F3H_Pyrus_pyrifolia                RLKKLAK.EQ....ELQDL.........EKDK.......VEIKP..ANDIFA......
AGL50918.1_F3H_Pyrus_communis                 RLKKLAK.EQ....QLQDL.........ETAK.......VDTKP..VDDIFA......
ACP30361.1_F3H_Malus_hybrid_cultivar          RLKKLAK.EQ....QSQDL.........EKAK.......VDTKP..VDDIFA......
ABG78792.1_F3H_Aethusa_cynapium               KLKKLAK.EKLL..QDQDT.........EKAK.......LQTKPKSADEIFA......
AAX21540.1_F3H_Anethum_graveolens             KLKKLAK.DKLL..QEQDA.........DKAK.......LQTTPKTADEIFA......
AAX21539.1_F3H_Ammi_majus                     KLKKLAK.EKLL..QEQEA.........EKAK.......LQMTPKSADEIFA......
AAU04791.1_F3H_Fragaria_x_ananassa            RLKKHAK.EQ....L.QDS.........EKTK.......LEAKP..VDDIFA......
AAU93347.1_F3H_Ginkgo_biloba                  RQKKLAKLQQ.............................DESK...............
AAT94365.1_F3H_Glycine_max                    RMKKLAK.EK....HLQDLENEKHLQELDQKAK.......LEAKP..LKEILA......
AAX21535.1_F3H_Pimpinella_anisum              KLKKLAK.EKVL..QEQEA.........EKAK.......LQMTPKSADEIFA......
AAP57394.1_F3H_Petroselinum_crispum           TLKKLAK.EKVL..QDQEV.........EKAK.......LQMTPKSADEIFA......
AAC49929.1_F3H_Petunia_x_hybrida              RLKKQAK.EQ....QLQAEVAA......EKAK.......LESKP..IEEILA......
                                         acc

NP_190692.1_F3H_Arabidopsis_thaliana

NP_190692.1_F3H_Arabidopsis_thaliana          ............................................................
TRINITY_DN33424_c3_g3_i1                      ............................................................
AAU04792.1_F3H_Fragaria_x_ananassa            ............................................................
NP_001236797.1_F3H_Glycine_max                ............................................................
ACR15123.1_F3H_Medicago_truncatula            ............................................................
AQX36284.1_F3H_Prunus_persica                 ............................................................
ABR13013.1_F3H_Triticum_aestivum              ............................................................
NP_001130275.1_F3H_Zea_mays                   ............................................................
ACL54955.1_F3H_Actinidia_chinensis            ............................................................
AFN70721.1_F3H_Nekemias_grossedentata         ............................................................
ACM62745.1_F3H_Garcinia_mangostana            ............................................................
AEO36935.1_F3H_Canarium_album                 ............................................................
AEG64806.1_F3H_Carthamus_tinctorius           ............................................................
AFC37250.1_F3H_Camellia_chekiangoleosa        ............................................................
ACB56921.1_F3H_Pilosella_officinarum          ............................................................
AAB97310.1_F3H_Chrysanthemum_x_morifolium     ............................................................
AAC15414.1_F3H_Nicotiana_tabacum              SLCHPVSVCSCQILLVEINLAIVCRNIVAYNPDFEKRILEAAANSNGTYILEMDDNSLHE
ABL86673.1_F3H_Gossypium_barbadense           ............................................................
ADC96713.1_F3H_Gossypium_hirsutum             ............................................................
XP_007046698.1_F3H_Theobroma_cacao            ............................................................
ABO48521.1_F3H_Dimocarpus_longan              ............................................................
ACQ99190.1_F3H_Fagopyrum_tataricum            ............................................................
BAL43067.1_F3H_Ipomoea_coccinea               ............................................................
AAB41102.1_F3H_Ipomoea_purpurea               ............................................................
BAK78917.1_F3H_Ipomoea_quamoclit              ............................................................
ADO95201.1_F3H_Litchi_chinensis               ............................................................
AIB06738.1_F3H_Mangifera_indica               ............................................................
AEF14415.1_F3H_Onobrychis_viciifolia          ............................................................
AFI71897.1_F3H_Paeonia_lactiflora             ............................................................
AEN71544.1_F3H_Paeonia_suffruticosa           ............................................................
CAA53579.1_F3H_Vitis_vinifera                 ............................................................
AGS57503.1_F3H_Vitis_rotundifolia             ............................................................
ACM17897.1_F3H_Rubus_occidentalis             ............................................................
ABW74548.1_F3H_Rubus_coreanus                 ............................................................
ADP09378.1_F3H_Pyrus_pyrifolia                ............................................................
AGL50918.1_F3H_Pyrus_communis                 ............................................................
ACP30361.1_F3H_Malus_hybrid_cultivar          ............................................................
ABG78792.1_F3H_Aethusa_cynapium               ............................................................
AAX21540.1_F3H_Anethum_graveolens             ............................................................
AAX21539.1_F3H_Ammi_majus                     ............................................................
AAU04791.1_F3H_Fragaria_x_ananassa            ............................................................
AAU93347.1_F3H_Ginkgo_biloba                  ............................................................
AAT94365.1_F3H_Glycine_max                    ............................................................
AAX21535.1_F3H_Pimpinella_anisum              ............................................................
AAP57394.1_F3H_Petroselinum_crispum           ............................................................
AAC49929.1_F3H_Petunia_x_hybrida              ............................................................
                                         acc
```

NP_190692.1_F3H_Arabidopsis_thaliana

```
NP_190692.1_F3H_Arabidopsis_thaliana              ............................................................
TRINITY_DN33424_c3_g3_i1                          ............................................................
AAU04792.1_F3H_Fragaria_x_ananassa                ............................................................
NP_001236797.1_F3H_Glycine_max                    ............................................................
ACR15123.1_F3H_Medicago_truncatula                ............................................................
AQX36284.1_F3H_Prunus_persica                     ............................................................
ABR13013.1_F3H_Triticum_aestivum                  ............................................................
NP_001130275.1_F3H_Zea_mays                       ............................................................
ACL54955.1_F3H_Actinidia_chinensis                ............................................................
AFN70721.1_F3H_Nekemias_grossedentata             ............................................................
ACM62745.1_F3H_Garcinia_mangostana                ............................................................
AEO36935.1_F3H_Canarium_album                     ............................................................
AEG64806.1_F3H_Carthamus_tinctorius               ............................................................
AFC37250.1_F3H_Camellia_chekiangoleosa            ............................................................
ACB56921.1_F3H_Pilosella_officinarum              ............................................................
AAB97310.1_F3H_Chrysanthemum_x_morifolium         ............................................................
AAC15414.1_F3H_Nicotiana_tabacum                  NRFVRDEDERPKVPYNKFSSEIPVISLKGIDDVEESSGGIKSRRAEICEKIIACEDWGIF
ABL86673.1_F3H_Gossypium_barbadense               ............................................................
ADC96713.1_F3H_Gossypium_hirsutum                 ............................................................
XP_007046698.1_F3H_Theobroma_cacao                ............................................................
ABO48521.1_F3H_Dimocarpus_longan                  ............................................................
ACQ99190.1_F3H_Fagopyrum_tataricum                ............................................................
BAL43067.1_F3H_Ipomoea_coccinea                   ............................................................
AAB41102.1_F3H_Ipomoea_purpurea                   ............................................................
BAK78917.1_F3H_Ipomoea_quamoclit                  ............................................................
ADO95201.1_F3H_Litchi_chinensis                   ............................................................
AIB06738.1_F3H_Mangifera_indica                   ............................................................
AEF14415.1_F3H_Onobrychis_viciifolia              ............................................................
AFI71897.1_F3H_Paeonia_lactiflora                 ............................................................
AEN71544.1_F3H_Paeonia_suffruticosa               ............................................................
CAA53579.1_F3H_Vitis_vinifera                     ............................................................
AGS57503.1_F3H_Vitis_rotundifolia                 ............................................................
ACM17897.1_F3H_Rubus_occidentalis                 ............................................................
ABW74548.1_F3H_Rubus_coreanus                     ............................................................
ADP09378.1_F3H_Pyrus_pyrifolia                    ............................................................
AGL50918.1_F3H_Pyrus_communis                     ............................................................
ACP30361.1_F3H_Malus_hybrid_cultivar              ............................................................
ABG78792.1_F3H_Aethusa_cynapium                   ............................................................
AAX21540.1_F3H_Anethum_graveolens                 ............................................................
AAX21539.1_F3H_Ammi_majus                         ............................................................
AAU04791.1_F3H_Fragaria_x_ananassa                ............................................................
AAU93347.1_F3H_Ginkgo_biloba                      ............................................................
AAT94365.1_F3H_Glycine_max                        ............................................................
AAX21535.1_F3H_Pimpinella_anisum                  ............................................................
AAP57394.1_F3H_Petroselinum_crispum               ............................................................
AAC49929.1_F3H_Petunia_x_hybrida                  ............................................................
                                          acc
```

NP_190692.1_F3H_Arabidopsis_thaliana

```
NP_190692.1_F3H_Arabidopsis_thaliana              ............................................................
TRINITY_DN33424_c3_g3_i1                          ............................................................
AAU04792.1_F3H_Fragaria_x_ananassa                ............................................................
NP_001236797.1_F3H_Glycine_max                    ............................................................
ACR15123.1_F3H_Medicago_truncatula                ............................................................
AQX36284.1_F3H_Prunus_persica                     ............................................................
ABR13013.1_F3H_Triticum_aestivum                  ............................................................
NP_001130275.1_F3H_Zea_mays                       ............................................................
ACL54955.1_F3H_Actinidia_chinensis                ............................................................
AFN70721.1_F3H_Nekemias_grossedentata             ............................................................
ACM62745.1_F3H_Garcinia_mangostana                ............................................................
AEO36935.1_F3H_Canarium_album                     ............................................................
AEG64806.1_F3H_Carthamus_tinctorius               ............................................................
AFC37250.1_F3H_Camellia_chekiangoleosa            ............................................................
ACB56921.1_F3H_Pilosella_officinarum              ............................................................
AAB97310.1_F3H_Chrysanthemum_x_morifolium         ............................................................
AAC15414.1_F3H_Nicotiana_tabacum                  QVVDHGVDTKMLSEMTRLAREFFEMSAEEKLRFDMTGGKKGGFIVSSHLQGEAVQDWREI
ABL86673.1_F3H_Gossypium_barbadense               ............................................................
ADC96713.1_F3H_Gossypium_hirsutum                 ............................................................
XP_007046698.1_F3H_Theobroma_cacao                ............................................................
ABO48521.1_F3H_Dimocarpus_longan                  ............................................................
ACQ99190.1_F3H_Fagopyrum_tataricum                ............................................................
BAL43067.1_F3H_Ipomoea_coccinea                   ............................................................
AAB41102.1_F3H_Ipomoea_purpurea                   ............................................................
BAK78917.1_F3H_Ipomoea_quamoclit                  ............................................................
ADO95201.1_F3H_Litchi_chinensis                   ............................................................
AIB06738.1_F3H_Mangifera_indica                   ............................................................
AEF14415.1_F3H_Onobrychis_viciifolia              ............................................................
AFI71897.1_F3H_Paeonia_lactiflora                 ............................................................
AEN71544.1_F3H_Paeonia_suffruticosa               ............................................................
CAA53579.1_F3H_Vitis_vinifera                     ............................................................
AGS57503.1_F3H_Vitis_rotundifolia                 ............................................................
ACM17897.1_F3H_Rubus_occidentalis                 ............................................................
ABW74548.1_F3H_Rubus_coreanus                     ............................................................
ADP09378.1_F3H_Pyrus_pyrifolia                    ............................................................
AGL50918.1_F3H_Pyrus_communis                     ............................................................
ACP30361.1_F3H_Malus_hybrid_cultivar              ............................................................
ABG78792.1_F3H_Aethusa_cynapium                   ............................................................
AAX21540.1_F3H_Anethum_graveolens                 ............................................................
AAX21539.1_F3H_Ammi_majus                         ............................................................
AAU04791.1_F3H_Fragaria_x_ananassa                ............................................................
AAU93347.1_F3H_Ginkgo_biloba                      ............................................................
AAT94365.1_F3H_Glycine_max                        ............................................................
AAX21535.1_F3H_Pimpinella_anisum                  ............................................................
AAP57394.1_F3H_Petroselinum_crispum               ............................................................
AAC49929.1_F3H_Petunia_x_hybrida                  ............................................................
                                          acc
```

NP_190692.1_F3H_Arabidopsis_thaliana

```
NP_190692.1_F3H_Arabidopsis_thaliana        ............................................................
TRINITY_DN33424_c3_g3_i1                    ............................................................
AAU04792.1_F3H_Fragaria_x_ananassa          ............................................................
NP_001236797.1_F3H_Glycine_max              ............................................................
ACR15123.1_F3H_Medicago_truncatula          ............................................................
AQX36284.1_F3H_Prunus_persica               ............................................................
ABR13013.1_F3H_Triticum_aestivum            ............................................................
NP_001130275.1_F3H_Zea_mays                 ............................................................
ACL54955.1_F3H_Actinidia_chinensis          ............................................................
AFN70721.1_F3H_Nekemias_grossedentata       ............................................................
ACM62745.1_F3H_Garcinia_mangostana          ............................................................
AEO36935.1_F3H_Canarium_album               ............................................................
AEG64806.1_F3H_Carthamus_tinctorius         ............................................................
AFC37250.1_F3H_Camellia_chekiangoleosa      ............................................................
ACB56921.1_F3H_Pilosella_officinarum        ............................................................
AAB97310.1_F3H_Chrysanthemum_x_morifolium   ............................................................
AAC15414.1_F3H_Nicotiana_tabacum            VTYFSYPIKARDYSRWPDKPKEWRAVTEKYSEDLMGLGCKLLEMLSEAMGLEKEALKDAC
ABL86673.1_F3H_Gossypium_barbadense         ............................................................
ADC96713.1_F3H_Gossypium_hirsutum           ............................................................
XP_007046698.1_F3H_Theobroma_cacao          ............................................................
ABO48521.1_F3H_Dimocarpus_longan            ............................................................
ACQ99190.1_F3H_Fagopyrum_tataricum          ............................................................
BAL43067.1_F3H_Ipomoea_coccinea             ............................................................
AAB41102.1_F3H_Ipomoea_purpurea             ............................................................
BAK78917.1_F3H_Ipomoea_quamoclit            ............................................................
ADO95201.1_F3H_Litchi_chinensis             ............................................................
AIB06738.1_F3H_Mangifera_indica             ............................................................
AEF14415.1_F3H_Onobrychis_viciifolia        ............................................................
AFI71897.1_F3H_Paeonia_lactiflora           ............................................................
AEN71544.1_F3H_Paeonia_suffruticosa         ............................................................
CAA53579.1_F3H_Vitis_vinifera               ............................................................
AGS57503.1_F3H_Vitis_rotundifolia           ............................................................
ACM17897.1_F3H_Rubus_occidentalis           ............................................................
ABW74548.1_F3H_Rubus_coreanus               ............................................................
ADP09378.1_F3H_Pyrus_pyrifolia              ............................................................
AGL50918.1_F3H_Pyrus_communis               ............................................................
ACP30361.1_F3H_Malus_hybrid_cultivar        ............................................................
ABG78792.1_F3H_Aethusa_cynapium             ............................................................
AAX21540.1_F3H_Anethum_graveolens           ............................................................
AAX21539.1_F3H_Ammi_majus                   ............................................................
AAU04791.1_F3H_Fragaria_x_ananassa          ............................................................
AAU93347.1_F3H_Ginkgo_biloba                ............................................................
AAT94365.1_F3H_Glycine_max                  ............................................................
AAX21535.1_F3H_Pimpinella_anisum            ............................................................
AAP57394.1_F3H_Petroselinum_crispum         ............................................................
AAC49929.1_F3H_Petunia_x_hybrida            ............................................................
                                        acc
```

NP_190692.1_F3H_Arabidopsis_thaliana

```
NP_190692.1_F3H_Arabidopsis_thaliana        ..................
TRINITY_DN33424_c3_g3_i1                    ..................
AAU04792.1_F3H_Fragaria_x_ananassa          ..................
NP_001236797.1_F3H_Glycine_max              ..................
ACR15123.1_F3H_Medicago_truncatula          ..................
AQX36284.1_F3H_Prunus_persica               ..................
ABR13013.1_F3H_Triticum_aestivum            ..................
NP_001130275.1_F3H_Zea_mays                 ..................
ACL54955.1_F3H_Actinidia_chinensis          ..................
AFN70721.1_F3H_Nekemias_grossedentata       ..................
ACM62745.1_F3H_Garcinia_mangostana          ..................
AEO36935.1_F3H_Canarium_album               ..................
AEG64806.1_F3H_Carthamus_tinctorius         ..................
AFC37250.1_F3H_Camellia_chekiangoleosa      ..................
ACB56921.1_F3H_Pilosella_officinarum        ..................
AAB97310.1_F3H_Chrysanthemum_x_morifolium   ..................
AAC15414.1_F3H_Nicotiana_tabacum            VDMDQKVVLNSYPKCPQP
ABL86673.1_F3H_Gossypium_barbadense         ..................
ADC96713.1_F3H_Gossypium_hirsutum           ..................
XP_007046698.1_F3H_Theobroma_cacao          ..................
ABO48521.1_F3H_Dimocarpus_longan            ..................
ACQ99190.1_F3H_Fagopyrum_tataricum          ..................
BAL43067.1_F3H_Ipomoea_coccinea             ..................
AAB41102.1_F3H_Ipomoea_purpurea             ..................
BAK78917.1_F3H_Ipomoea_quamoclit            ..................
ADO95201.1_F3H_Litchi_chinensis             ..................
AIB06738.1_F3H_Mangifera_indica             ..................
AEF14415.1_F3H_Onobrychis_viciifolia        ..................
AFI71897.1_F3H_Paeonia_lactiflora           ..................
AEN71544.1_F3H_Paeonia_suffruticosa         ..................
CAA53579.1_F3H_Vitis_vinifera               ..................
AGS57503.1_F3H_Vitis_rotundifolia           ..................
ACM17897.1_F3H_Rubus_occidentalis           ..................
ABW74548.1_F3H_Rubus_coreanus               ..................
ADP09378.1_F3H_Pyrus_pyrifolia              ..................
AGL50918.1_F3H_Pyrus_communis               ..................
ACP30361.1_F3H_Malus_hybrid_cultivar        ..................
ABG78792.1_F3H_Aethusa_cynapium             ..................
AAX21540.1_F3H_Anethum_graveolens           ..................
AAX21539.1_F3H_Ammi_majus                   ..................
AAU04791.1_F3H_Fragaria_x_ananassa          ..................
AAU93347.1_F3H_Ginkgo_biloba                ..................
AAT94365.1_F3H_Glycine_max                  ..................
AAX21535.1_F3H_Pimpinella_anisum            ..................
AAP57394.1_F3H_Petroselinum_crispum         ..................
AAC49929.1_F3H_Petunia_x_hybrida            ..................
                                        acc
```

```
Q9SD85.1_F3-H_Arabidopsis_thaliana           TT  TT    TT             TT                η1
                                             1        10        20        30        40
Q9SD85.1_F3-H_Arabidopsis_thaliana            ......MATLFLTILLATVLFLIL...RIFSHRRNR..SHNNRLPPGPNPWPIIGNLPHM
TRINITY_DN252_c0_g1_i1                        ...MSSFLLYF..ILFFISIYFL.....SSLRSFLR.....RRLPPGPKPWPIVGNLPHL
NP_001312537.1_F3-H_Nicotiana_tabacum         ...MAIFSLILYTVIFSFLLHSIF...SLFFRKRYP.....LTLPPGPKPWPIIGNLVHM
Q9SBQ9.1_F3-H_Petunia_x_hybrida               ...MEILSLILYTVIFSFLLQFIL..RSFFRKRYP.....LPLPPGPKPWPIIGNLVHL
AAG49298.1_F3-H_Callistephus_chinensis        ...MTILPFIFYTCITALVLYVL.....LNLLTRNP.....NRLPPGPTPWPIVGNLPHL
BAD91808.1_F3-H_Gentiana_triflora             .MEMNPSPFIVHTAVFGLFVVFIF..IFLKLVSRKKGHGRSLPLPPGPRPWPILGNIPHL
ABA64468.1_F3-H_Gerbera_hybrid_cultivar       ...MTPLTLLIGTCVTGLFLYVL.....LNRCTRNP.....NRLPPGPTPWPVVGNLPHL
AAO47847.1_F3-H_Glycine_max                   ...MSPLIVALATIAAAILIYRI.....IKFITRPS.....LPLPPGPKPWPIVGNLPHM
ABC47161.1_F3-H_Pilosella_officinarum         ...MTLLSLIIYLCITGVTAYVL.....VNLRTRRA.....NRLPPGPTPWPIVGNLPHL
BAD00190.1_F3-H_Ipomoea_nil                   ...MATLTLIFCTLVFAIFLYFLI....LRVKQRYP.....LPLPPGPKPWPVLGNLPHL
BAF49324.1_F3-H_Lobelia_erinus                ......MSLIFYTCIIAFLLYVLLSTLRTFLLNRNPA..SRKPLPPGPKPWPIVGNLPHL
AAG49301.1_F3-H_Matthiola_incana              ......MTTLILTILLATFLSLFI....FFLLRRNR..NRNHRLPPGPNPWPIVGNLPHM
ABB29899.1_F3-H_Osteospermum_hybrid_cultivar  ...MTILPLVLYSCITGLVIYVL.....LNLRTRHS.....NRLPPGPTPWPIVGNLPHL
AAG49315.1_F3-H_Pelargonium_x_hortorum        MYNMS.LYLLLGSSALAFAAYLV.....LFSFSKSR.....RRLPPGPKAWPIVGNLPHM
BAB59005.1_F3-H_Perilla_frutescens_var._crispa .MISAAVSLIICTSILGVLVYFLF.......LRRGGG.SNGRPLPPGPRPWPIVGNLPQL
BAE47006.1_F3-H_Vitis_vinifera                ...MNPLALFFCTALFCVLLYHF.....LTRRSV........RLPPGPKPWPIVGNLPHL
AAR00229.1_F3-H_Ipomoea_purpurea              ...MATLTLIFCTLVFAIFLYFLI....LRVKQRYP.....LPLPPGPKPWPVLGNLPHL
                                         acc

                                                 α1          β1      β2       α2      α3      η2
Q9SD85.1_F3-H_Arabidopsis_thaliana                             TT
                                             50        60        70        80        90        100
Q9SD85.1_F3-H_Arabidopsis_thaliana            GTKPHRTLSAMVTTYGPILHLRLGFVDVVVAASKSVAEQFLKIHDANFASRPPNSGAKHM
TRINITY_DN252_c0_g1_i1                        GPMPHHSIAALARTYGPLMHLRMGFVDVVVAASAAVASQFLKFHDANFSSRPPNSGAKYV
NP_001312537.1_F3-H_Nicotiana_tabacum         GPKPHQSTAAMARTYGPLMHLKMGFVDVVVASASVAAQFLKTHDANFSSRPPNSGAKHL
Q9SBQ9.1_F3-H_Petunia_x_hybrida               GPKPHQSTAAMAQTYGPLMYLKMGFVDVVVAASASVAAQFLKTHDANFSSRPPNSGAEHM
AAG49298.1_F3-H_Callistephus_chinensis        GMIPHHSLAALAQKYGPLMHLRLGFVDVVVAASASVAAQFLKTHDANFASRPPNSGAKHI
BAD91808.1_F3-H_Gentiana_triflora             GSKPHQTLAEMAKTYGPLMHLKFGLKDAVVASSASVAEQFLKKHDVNFSNRPPNSGAKHI
ABA64468.1_F3-H_Gerbera_hybrid_cultivar       GTIPHHSLAAMAKKYGPLMHLRLGFVDVVVAASASVAAQFLKTHDANFADRPPNSGAKHI
AAO47847.1_F3-H_Glycine_max                   GPVPHHSLAALARIHGPLMHLRLGFVDVVVAASASVAEQFLKIHDSNFSSRPPNAGAKYI
ABC47161.1_F3-H_Pilosella_officinarum         GTIPHHSLADLATRYGPLMHLRLGFVDVVVAASASVAAQFLKTHDANFASRPPNSGAKHM
BAD00190.1_F3-H_Ipomoea_nil                   GKKPHQSIAAMAERYGPLMHLRLGFVDVVVAASAAVAAQFLKVHDSNFSNRPPNSGAEHI
BAF49324.1_F3-H_Lobelia_erinus                GTMPHQTMAALARTYGPLMHLRLGSVDVVVAASGAVAEQFLKVHDANFLNRPPNSGAVHI
AAG49301.1_F3-H_Matthiola_incana              GPKPHQTLAAMVTTYGPILHLRLGFVNVVVAASKSVAEQFLKIHDANFASRPPNSGAKHI
ABB29899.1_F3-H_Osteospermum_hybrid_cultivar  GVVPHHSLAAMAEKYGPLMHLRLGFVDVVVAASAAVAAQFLKVHDANFASRPPNSGAKHI
AAG49315.1_F3-H_Pelargonium_x_hortorum        GSMPHQNLAAMARTYGPLVYLRLGFVDVVVALSASMASQFLKTHDSNFSSRPPNAGAKHI
BAB59005.1_F3-H_Perilla_frutescens_var._crispa GPKPHQSMAALARVHGPLMHLKMGFVHVVVAASATVAEKFLKVHDTNFLSRPPNSGAEHI
BAE47006.1_F3-H_Vitis_vinifera                GPVPHHSIAALAKTYGPLMHLRMGFVDVVVAASASVAAQFLKTHDANFSNRPPNSGAKHI
AAR00229.1_F3-H_Ipomoea_purpurea              GEKPHQSIAAMAERHGPLMHLRMGFVDVVVAASAAVAAQFLKVHDANFSNRPPNSGAEHI
                                         acc

                                                            α4            α5                β3
Q9SD85.1_F3-H_Arabidopsis_thaliana
                                            110       120        130       140       150       160
Q9SD85.1_F3-H_Arabidopsis_thaliana            AYNYQDLVFAP.YGHRWRLLRKISSVHLFSAKALEDFKHVRQEEVGTLTRELVRVGTKPV
TRINITY_DN252_c0_g1_i1                        AYNYQDLVFAP.YGPRWRMLRKISSVNLFSGKALDDFRHIRQHEVSVLTRALASAGSTPV
NP_001312537.1_F3-H_Nicotiana_tabacum         AYNYQDLVFAP.YGPRWRMLRKICSVHLFSAKALDDFSHVRQDEVRTLTRALASAAQKPV
Q9SBQ9.1_F3-H_Petunia_x_hybrida               AYNYQDLVFAP.YGPRWRMLRKICSVHLFSTKALDDFRHVRQDEVKTLTRALASAGQKPV
AAG49298.1_F3-H_Callistephus_chinensis        AYNYQDLVFAP.YGPRWRMLRKICSVHLFSTKALDDFRHVREEEVAILTRVLVHAGESAV
BAD91808.1_F3-H_Gentiana_triflora             AYNYQDLVFAP.YGPRWRLLRKICSVHLFSSKALDDFQHVRHEEICILIRAIASGGHAPV
ABA64468.1_F3-H_Gerbera_hybrid_cultivar       AYNYQDLVFAP.YGPRWRMLRKICSVHLFSTKALDDFRHVRQEEVAILARALVGAGKSPV
AAO47847.1_F3-H_Glycine_max                   AYNYQDLVFAP.YGPRWRLLRKLTSVHLFSGKAMNEFRHLRQEEVARLTCNLASSDTKAV
ABC47161.1_F3-H_Pilosella_officinarum         AYNYQDLVFAP.YGPRWRMLRKICSVHLFSAKSLDDFRHVRQEEVAILTRALVGAGKSTV
BAD00190.1_F3-H_Ipomoea_nil                   AYNYQDLVFAP.YGPRWRMLRKITSVHLFSAKALDDFCHVRQEEVATLTRSLASAGKTPV
BAF49324.1_F3-H_Lobelia_erinus                AYNYQDLVFAP.YGPRWRLLRKVSAVHLFSAKALDDFRHVRQEEVAVLTRDLASAGNAPV
AAG49301.1_F3-H_Matthiola_incana              AYNYQDLVFAP.YGQRWRMLRKISSVHLFSAKALEDFKHVRQEEIGRLTREVARADTKPV
ABB29899.1_F3-H_Osteospermum_hybrid_cultivar  AYNYQDLVFAPYYGPRWRMLRKICSVHLFSSKALDDFRHVRQEEVAILTRALIGAGDSPV
AAG49315.1_F3-H_Pelargonium_x_hortorum        AYNYHDLVFAP.YGPRWRLFRKITSIHLFSGKALDDYRHVRQEEVGVLASNLARAVSTIV
BAB59005.1_F3-H_Perilla_frutescens_var._crispa AYNYNDLVFAP.HGPRWRLLRKICALHLFSSKALDDFRHVREEEVGILIRNLASVGEMPA
BAE47006.1_F3-H_Vitis_vinifera                AYNYQDLVFAP.YGPRWRMLRKICSVHLFSGKALDDFRHIRQEEVAVLTRALARAGQTPV
AAR00229.1_F3-H_Ipomoea_purpurea              AYNYQDLVFAP.YGPRWRMLRKITSVHLFSAKALDDFCHVRQEEVATLTRSLASAGKTPV
                                         acc

                                                    α6                              α7          η3
Q9SD85.1_F3-H_Arabidopsis_thaliana
                                            170       180       190        200       210       220
Q9SD85.1_F3-H_Arabidopsis_thaliana            NLGQLVNMCVVNALGREMIGRRLFGADA.......DHKADEFRSMVTEMMALAGVFNIGD
TRINITY_DN252_c0_g1_i1                        NLGQLLNVCTANALGRVMIGRRVFGDGSGGG....DRKADEFKTMVVELMVLAGVFNIGD
NP_001312537.1_F3-H_Nicotiana_tabacum         KLGQLLNVCTTNALAQVMLGRRVFADANGGV....DPQAEEFKSMVVEAMVLAGVFNVGD
Q9SBQ9.1_F3-H_Petunia_x_hybrida               KLGQLLNVCTTNALARVMLGKRVFADGSGDV....DPQAAEFKSMVVEMMVVAGVFNIGD
AAG49298.1_F3-H_Callistephus_chinensis        KLGQLLNVCTTNALARVMLGRRVFADGSEGRG..VDPKADEFKDMVVELMELAGEFNIGD
BAD91808.1_F3-H_Gentiana_triflora             NLGKLLGVCTTNALARVMLGRRVF.EGDGGE....NPHADEFKSMVVEIMVLAGAFNLGD
ABA64468.1_F3-H_Gerbera_hybrid_cultivar       KLGQLLNVCTTNALARVMLGRRVFDSG........DAQADEFKDMVVELMVLAGEFNIGD
AAO47847.1_F3-H_Glycine_max                   NLGQLLNVCTTNALARAMIGRRVFNDGNGGC....DPRADEFKAMVMEVMVLAGVFNIGD
ABC47161.1_F3-H_Pilosella_officinarum         KLGQLLHVCTTNALVRVMLGRRVFGDGSGGG....DPKADEFKNMVIEMMVLAGEFNLGD
BAD00190.1_F3-H_Ipomoea_nil                   KLGQLLNVCTTNALARVMLGRKVFNDGGSKS....DPKAEEFKSMVEEMMVLAGSFNIGD
BAF49324.1_F3-H_Lobelia_erinus                HLNQLLSVCTTNALSRVMFGKRVFGGGG.......DSKADEFKQMVVEAMVLAGVFNIGD
AAG49301.1_F3-H_Matthiola_incana              NLGQLVNMCVVNALGREMIGRRLFGDGA.......DHKAEEFRSMVTEMMALAGVFNVGD
ABB29899.1_F3-H_Osteospermum_hybrid_cultivar  KLGQLLNVCTTNALARVMLGKRVFGDRSGGG....DPKADEFKDMVVEVMELAGEFNIGD
AAG49315.1_F3-H_Pelargonium_x_hortorum        NLGQLLNICATNALGRAVIGKKVFKDGTDDV....DPKADEFKSMVVELMVLAGVFNIGD
BAB59005.1_F3-H_Perilla_frutescens_var._crispa SIGQMMYVCATNAISRVMLGRHVLGDEHRGAAGGGDTTAEEFKAMVVELMALAGVFNVGD
BAE47006.1_F3-H_Vitis_vinifera                NLGQLLNVCTTNALGRVMLGRRVFGDGSGGE....DPKADEFKEMVVELMVLAGVFNIGD
AAR00229.1_F3-H_Ipomoea_purpurea              KLGQLLNVCTTNALARVMLGRKVFNDGGSKS....DPKAEEFKSMVEEMMVLAGSFNIGD
                                         acc
```

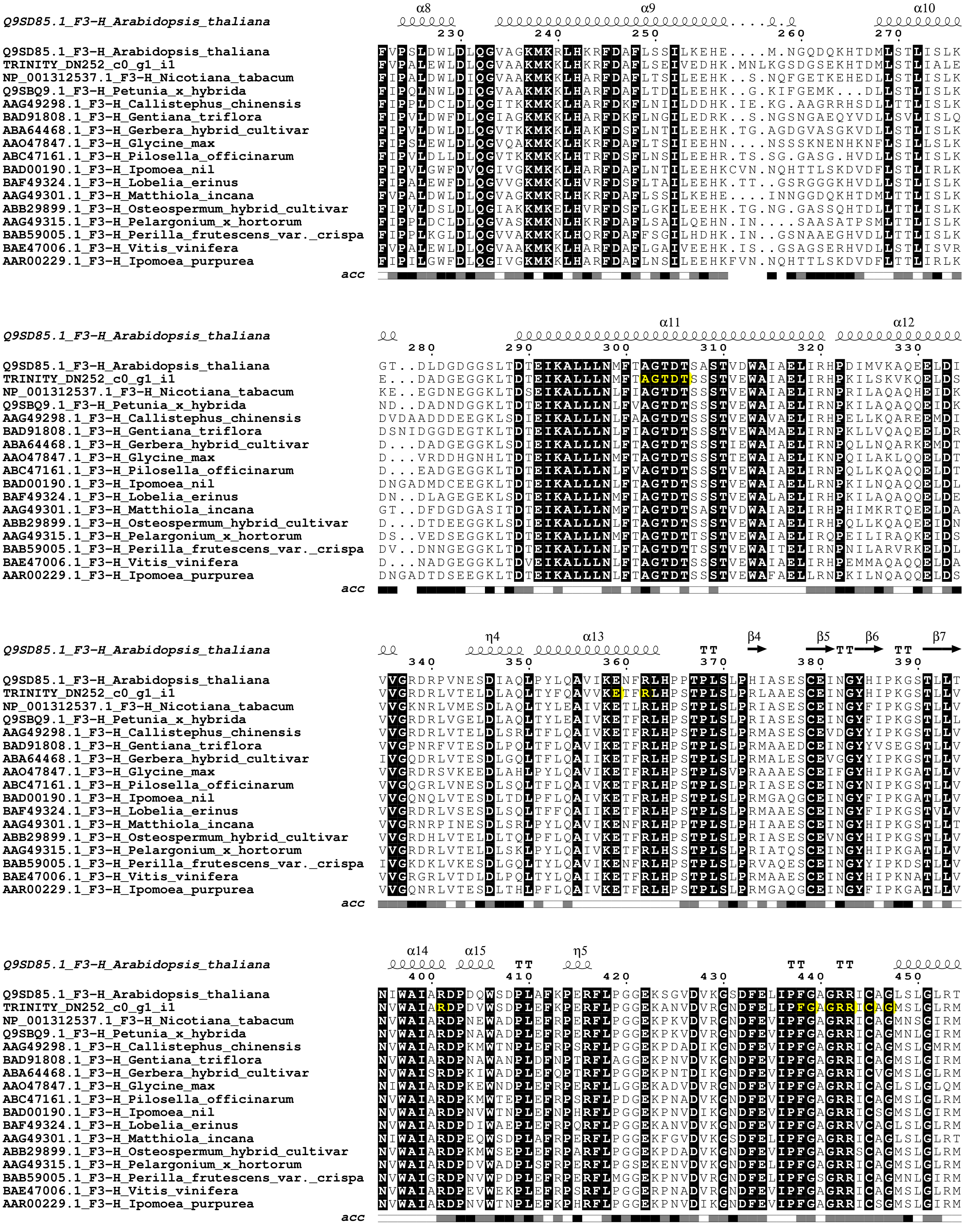

Q9SD85.1_F3-H_Arabidopsis_thaliana
α8 α9 α10 α11 α12 η4 α13 β4 β5 β6 β7 α14 α15 η5 TT
230 240 250 260 270 280 290 300 310 320 330 340 350 360 370 380 390 400 410 420 430 440 450
Q9SD85.1_F3-H_Arabidopsis_thaliana
TRINITY_DN252_c0_g1_i1
NP_001312537.1_F3-H_Nicotiana_tabacum
Q9SBQ9.1_F3-H_Petunia_x_hybrida
AAG49298.1_F3-H_Callistephus_chinensis
BAD91808.1_F3-H_Gentiana_triflora
ABA64468.1_F3-H_Gerbera_hybrid_cultivar
AAO47847.1_F3-H_Glycine_max
ABC47161.1_F3-H_Pilosella_officinarum
BAD00190.1_F3-H_Ipomoea_nil
BAF49324.1_F3-H_Lobelia_erinus
AAG49301.1_F3-H_Matthiola_incana
ABB29899.1_F3-H_Osteospermum_hybrid_cultivar
AAG49315.1_F3-H_Pelargonium_x_hortorum
BAB59005.1_F3-H_Perilla_frutescens_var._crispa
BAE47006.1_F3-H_Vitis_vinifera
AAR00229.1_F3-H_Ipomoea_purpurea
acc

```
*Q9SD85.1_F3-H_Arabidopsis_thaliana*            α16          β8          α17   β9    β10     β11
                                                        460         470         480         490         500         510
Q9SD85.1_F3-H_Arabidopsis_thaliana              IQFLTATLVQGFDWELAGGVTPEKLNMEESYGLTLQRAVPLVVHPKPRLAPNVYGLGSG
TRINITY_DN252_c0_g1_i1                          VQLLIATLVHGFDWELADGLMPDKLNMDEAFGLTLQRAVPLMVHPRPRLPLHAYGASN.
NP_001312537.1_F3-H_Nicotiana_tabacum           VQLMTATLIHAFNWDLSIGQSPEKLNMEEAFGLTLQRADPLVVHPCLRLEAQAYIG...
Q9SBQ9.1_F3-H_Petunia_x_hybrida                 VQLMIATLIHAFNWDLVSGQLPEMLNMEEAYGLTLQRADPLVVHPRPRLEAQAYIG...
AAG49298.1_F3-H_Callistephus_chinensis          VQLLIATLVQTFDWELANGLDPEKLNMEEAYGLTLQRAEPLMVHPRPRLSPHVYESR..
BAD91808.1_F3-H_Gentiana_triflora               VQLVTASLVHSFDWALLDGLKPEKLDMEEGYGLTLQRASPLIVHPKPRLSAQVYCM...
ABA64468.1_F3-H_Gerbera_hybrid_cultivar         VQLLTATLIHAFDWELADGLNPKKLNMEEAYGLTLQRAAPLVVHPRPRLAPHVYETTKV
AAO47847.1_F3-H_Glycine_max                     VQLLTAALAHSFDWELEDCMNPEKLNMDEAYGLTLQRAVPLSVHPRPRLAPHVYSMSS.
ABC47161.1_F3-H_Pilosella_officinarum           VQLLTATLIQAFDWELANGLEPRNLNMEEAYGLTLQRAQPLMVHPRPRLAPHVYGTG..
BAD00190.1_F3-H_Ipomoea_nil                     VHLLVATLVHAFDWDLVNGQSVETLNMEEAYGLTLQRAVPLMLHPKPRLQPHLYTLN..
BAF49324.1_F3-H_Lobelia_erinus                  VQLVTATLVHSFDWELADGQKPEELNMEEGYGLTLQRAKPLKVHPRPRLSEHVYQA...
AAG49301.1_F3-H_Matthiola_incana                IQLLTATLVHGFEWELAGGVTPEKLNMEETYGITVQRAVPLIVHPKPRLALNVYGVGSG
ABB29899.1_F3-H_Osteospermum_hybrid_cultivar    VQLLVATLVQTFDWELANGLKPEKLNMEEAYGLTLQRAAPLLVHPKPRLAPHVYGSN..
AAG49315.1_F3-H_Pelargonium_x_hortorum          VQLLTATLLHAFNWDLPQGQIPQELNMDEAYGLTLQRASPLHVRPRPRLPSHLY.....
BAB59005.1_F3-H_Perilla_frutescens_var._crispa  VQLLIATMVHAFDFELANGQLAKDLNMEEAYGITLQRADPLVVHPRPRLARHVYQAQV.
BAE47006.1_F3-H_Vitis_vinifera                  VHLLTATLVHAFNWELPEGQVAEKLNMDEAYGLTLQRAPPLMVHPRPRLSPQVFGK...
AAR00229.1_F3-H_Ipomoea_purpurea                VHLLVATLVHAFDWDLMNGQSVETLNMEEAYGLTLQRAVPLMLHPKPRLQPHLYTLN..
                                            acc
```

```
NP_001234840.2_F3-5-H_Solanum_lycopersicum                                                               π1
                                                                                                          1         10

NP_001234840.2_F3-5-H_Solanum_lycopersicum              .................................................MALRINELF.V
TRINITY_DN32466_c16_g7_i1                               .............................................MAFHALFFLQELS.V
TRINITY_DN32466_c16_g7_i3                               .............................................MAFHALFFLQELS.V
ACN38269.1_F3-5-H_Vitis_amurensis                       .............................................MAI.DTSLLLELA.A
NP_001268157.1_F3-5-H_Vitis_vinifera                    .............................................MAI.DTSLLLEFA.A
APY18930.1_F3-5-H_Camellia_sinensis_var._sinensis       .............................................MALDTVFLLRELS.F
BAA03438.1_F3-5-H_Petunia_x_hybrida                     ..................................................MMLLTELG.A
BAA03440.1_F3-5-H_Campanula_medium                      .............................................MSIDISTLFYELV.A
CAA50155.1_F3-5-H_Solanum_melongena                     .................................................MVILPSELI.G
BAC97831.1_F3-5-H_Vinca_major                           ...................................................MAIVDFL.A
CAA09850.1_F3-5-H_Catharanthus_roseus                   .............................................MAL.EKLVLFDFL.A
AAX51796.1_F3-5-H_Delphinium_grandiflorum               ..................................................MSISLFLA.G
BAA12735.1_F3-5-H_Gentiana_triflora                     .............................................MSPIYTTLTLHLA.T
AAM51564.1_F3-5-H_Glycine_max                           ...............................................MDSLLLLKEIA.T
AAP31058.1_F3-5-H_Gossypium_hirsutum                    .............................................MPSFDTILLRDLV.A
BAC10997.1_F3-5-H_Nierembergia_sp._NB17                 .................................................MMQLSTELA.I
AAG49300.1_F3-5-H_Lycianthes_rantonnei                  ...............................................M.VLLLISELC.A
AAV85471.1_F3-5-H_Solanum_tuberosum                     .................................................MTLRISELF.A
BAB20076.1_F3-5-H_Torenia_hybrid_cultivar               .............................................MTV.ATTLVVVLS.R
AAT34974.1_F3-5-H_Glandularia_x_hybrida                 ...........................................MTFSELINILFWDLT.A
AAG49299.1_F3-5-H_Callistephus_chinensis                .............................................MS..ILSLLVYFC.I
ABB43030.1_F3-5-H_Pericallis_cruenta                    .............................................MS..ILTLICTF..I
ABB43031.1_F3-5-H_Osteospermum_hybrid_cultivar          .............................................MAI.IMTLLMDTC.I
AHI15949.1_F3-5-H_Pohlia_nutansSolanum_tuberosum        .............................................MGA.VEDAVAHVC.G
AHI15952.1_F3-5-H_Pohlia_nutansVinca_major              MKRLLLLVVSISTSVIRALVVAVGLCRIHIFAKENFPAFACFCKNMGASIVAAVAHVTRH
                                                    acc
```

```
NP_001234840.2_F3-5-H_Solanum_lycopersicum              π2                          η1                              α1
                                                                 20        30        40        50        60

NP_001234840.2_F3-5-H_Solanum_lycopersicum              AAIIY...IIVHIIISKLITTVRE.R.GRRLPLPPGPTGWPVIGALPLLGSMPHVALAKM
TRINITY_DN32466_c16_g7_i1                               SAILY...FVTKIIIEQLLPKPYRPR......LPPGPKGWPVIGALPLLGTMPHVSLAKL
TRINITY_DN32466_c16_g7_i3                               SAILY...FVTKIIIEQLLPKPYRPR......LPPGPKGWPVIGALPLLGTMPHVSLAKL
ACN38269.1_F3-5-H_Vitis_amurensis                       ATLLF...FITRFFIRSLLPKSSR.K......VPPGPKGWPLVGALPLLGNMPHVALAKM
NP_001268157.1_F3-5-H_Vitis_vinifera                    ATLLF...FITRFFIRSLLPKPSR.K......LPPGPKGWPLLGALPLLGNMPHVALAKM
APY18930.1_F3-5-H_Camellia_sinensis_var._sinensis       ATLVI...LITHIFMRSILSKPLR.M......LPPGPTGLPLIGAIPHLGSMPHVALAKM
BAA03438.1_F3-5-H_Petunia_x_hybrida                     ATSIF...LIAHIIISTLISKTTG.R.H....LPPGPRGWPVIGALPLLGAMPHVSLAKM
BAA03440.1_F3-5-H_Campanula_medium                      AISLY...LATYSFIRFLFKPSHHHH......LPPGPTGWPIIGALPLLGTMPHVSLADM
CAA50155.1_F3-5-H_Solanum_melongena                     ATIIY...IIVYIIIQKLIATGSW.R.RRR..LPPGPEGWPVIGALPLLGGMPHVALAKM
BAC97831.1_F3-5-H_Vinca_major                           AFLIF...ILTQKLIQTLFRRTYR.R.K....LPPGPKGWPVIGALPYLGTMPHTSLAYM
CAA09850.1_F3-5-H_Catharanthus_roseus                   AISIL...ILVQKFIQIVFLRSSS.RIR....LPPGPKGWPIIGALPYLGTMPHSILANM
AAX51796.1_F3-5-H_Delphinium_grandiflorum               AAILF...FVTHLLLSPT.......RTRK...LPPGPKGWPVVGALPMLGNMPHVALANL
BAA12735.1_F3-5-H_Gentiana_triflora                     ALFLF...FHVQKLVHYLHGKATGHRCRR...LPPGPTGWPILGALPLLGNMPHVTFANM
AAM51564.1_F3-5-H_Glycine_max                           SILIF...LITRLSIQTFLKSYRQ.K......LPPGPKGWPVVGALPLMGSMPHVTLAKM
AAP31058.1_F3-5-H_Gossypium_hirsutum                    AACLF...FITRYFIRRLLSNPKR.T......LPPGPKGWPIVGALPLLGSMPHVELAKL
BAC10997.1_F3-5-H_Nierembergia_sp._NB17                 AAIIF...LLAHI....LISKTSG.R.R....LPPGPRGWPVIGALPLLGDMPHVSLAKM
AAG49300.1_F3-5-H_Lycianthes_rantonnei                  SAIIF...IIVHIIISKLIATGWG.R.RQR..LPPGPMGWPVIGALPLLGTMPHVALAKM
AAV85471.1_F3-5-H_Solanum_tuberosum                     AAIIY...IIVHIIISKLIATVRE.R.GRR..LPPGPTGWPVIGALSLLGSMPHVALAKM
BAB20076.1_F3-5-H_Torenia_hybrid_cultivar               ELLLYV..LVVYILVSKSLSTIIVVS.RKR..LPPGPTGFPVVGALPLLGSMPHVALAKM
AAT34974.1_F3-5-H_Glandularia_x_hybrida                 AILLY....VALILVCSLYSKPSTVS.RN...LPPGPKGFPVVGALPLLGTMPHIALAKM
AAG49299.1_F3-5-H_Callistephus_chinensis                SLLVI......IALVNMFITRHTN.R......LPPGPAPWPVVGNLPHLGAIPHHTLAAL
ABB43030.1_F3-5-H_Pericallis_cruenta                    TGLMF......YGLVNL.LSRRAS.R......LPPGPTPWPIIGNLMHLGKLPHHSLADL
ABB43031.1_F3-5-H_Osteospermum_hybrid_cultivar          TVLVF......YVLLNL.RTRHPN.R......LPPGPTPWPIVGNLPHLGPLPHHTLAAF
AHI15949.1_F3-5-H_Pohlia_nutansSolanum_tuberosum        SFFEYGVAVLAIFLLIVLIQRP...K.....NLPPGPTGLPIIGALHLLGQRPHETLARM
AHI15952.1_F3-5-H_Pohlia_nutansVinca_major              SLLEYGVAVLAILGLVMVLRRPLK.K.....KLPPGPVGLPIMGSLHLLGPRPHQRLAQM
                                                    acc
```

```
NP_001234840.2_F3-5-H_Solanum_lycopersicum                  β1    TT  β2          α2          η2         η3           TT
                                                               70        80        90        100       110       120

NP_001234840.2_F3-5-H_Solanum_lycopersicum              AKKYGPIMYLKVGTCGMVVASTPNAAKAFLKTLDINFSNRPPNAGATHLAYNAQDMVFAP
TRINITY_DN32466_c16_g7_i1                               SKKYGEVMYLKMGTCNMVVASSPESAKAFLKTLDLNFSNRPPNAGATHLAYNAQDMVFAD
TRINITY_DN32466_c16_g7_i3                               SKKYGEVMYLKMGTCNMVVASSPESAKAFLKTLDLNFSNRPPNAGATHLAYNAQDMVFAD
ACN38269.1_F3-5-H_Vitis_amurensis                       AKRYGPVMFLKMGTNGMVVASTPGAARAFLKTLDINFSNRPPNAGATLLAYHAQDMVFAD
NP_001268157.1_F3-5-H_Vitis_vinifera                    AKRYGPVMFLKMGTNSMVVASTPEAARAFLKTLDINFSNRPPNAGATLLAYHAQDMVFAD
APY18930.1_F3-5-H_Camellia_sinensis_var._sinensis       AKIYGPIVYLKMGTCGMVVASTPDSARAFLKTLDLNFSNRPPNAGATHLAYGAQDMVFAD
BAA03438.1_F3-5-H_Petunia_x_hybrida                     AKKYGAIMYLKVGTCGMAVASTPDAAKAFLKTLDINFSNRPPNAGATHLAYNAQDMVFAH
BAA03440.1_F3-5-H_Campanula_medium                      AVKYGPIMYLKLGSKGTVVASNPKAARAFLKTHDANFSNRPIDGGPTYLAYNAQDMVFAE
CAA50155.1_F3-5-H_Solanum_melongena                     AKKYGPIMYLKVGTCGMVVASTPNAAKAFLKTLDINFSNRPPNAGATHMAYNAQDMVFAP
BAC97831.1_F3-5-H_Vinca_major                           AKKYGPVMYLKVGTNDMVVVSTPDAARAFLKTLDINFSNRPPNAGATHLAYGAQDMVFAH
CAA09850.1_F3-5-H_Catharanthus_roseus                   AKKYGPIMYLKLGTNGMVVASTPDAVKAFLRTLDMNFSNRPIDAGATHLAYNAQDMVFAH
AAX51796.1_F3-5-H_Delphinium_grandiflorum               SRRYGPIVYLKLGSRGMVVASTPDSARAFLKTQDLNFSNRPTDAGATHIAYNSQDMVFAD
BAA12735.1_F3-5-H_Gentiana_triflora                     AKKYGSVMYLKVGSHGLAIASTPDAAKAFLKTLDLNFSNRPPNAGATHLAYNAQDMVFAH
AAM51564.1_F3-5-H_Glycine_max                           AKKYGPIMYLKMGTNNMVVASTPAAARAFLKTLDQNFSNRPSNAGATHLAYDARDMVFAH
AAP31058.1_F3-5-H_Gossypium_hirsutum                    AKKYGPVMYLKMGTCNMVVASTPDAARAFLKTLDLNFSNRPSNAGATHIAYNSQDMVFAE
BAC10997.1_F3-5-H_Nierembergia_sp._NB17                 AKKYGPIMYLKVGTCGMAVASTPHAAKAFLKTLDINFSNRPPNAGATHFAYNAQDMVFAP
AAG49300.1_F3-5-H_Lycianthes_rantonnei                  AKKYGPIMYLKVGTCGMVVASTPNAAKAFLKTLDINFSNRPPNAGATHLAYDAQDMVFAH
AAV85471.1_F3-5-H_Solanum_tuberosum                     AKNYGPIMYLKVGTCGMVVASTPNAAKAFLKTLDINFSNRPPNAGATHLAYNAQDMVFAP
BAB20076.1_F3-5-H_Torenia_hybrid_cultivar               AKKYGPVMYLKTGTLGMVVASTPSSARAFLKTLDSNFSNRPANAGATHLAYGAQDMVFAP
AAT34974.1_F3-5-H_Glandularia_x_hybrida                 AKTYGPVMYLKVGTWGMAVASTPDAARAFLKTLDTTFSDRPPNAGATLLAYNAQDMVFAH
AAG49299.1_F3-5-H_Callistephus_chinensis                ATKYGPLVYLRLGFVHVVVASSPSVAAQFLKVHDLKFASRPPNSGAKHIAYNYQDMVFAP
ABB43030.1_F3-5-H_Pericallis_cruenta                    AKKYGPLIHVRLGSVDVVVASSASVAGQFLKVHDANFANRPPNSGAKHMAYNYHDMVFAP
ABB43031.1_F3-5-H_Osteospermum_hybrid_cultivar          AKKYGPLIHLRFGFVDVVVASSPTVASQFLRDNDLNFASRPPNSGAKHMAYNYQDLVFAP
AHI15949.1_F3-5-H_Pohlia_nutansSolanum_tuberosum        ADKYGPLMSLYMGQQLCIVASSRDTAMEFLKRQDAVFCSRPPQRGFEVIF..PKDITFSD
AHI15952.1_F3-5-H_Pohlia_nutansVinca_major              AEKYGPVMSLYMGQKLCVVATSADTAMEFLKAQDAVFCSRPPLRAAQVIF..PTDITFSD
                                                    acc
```

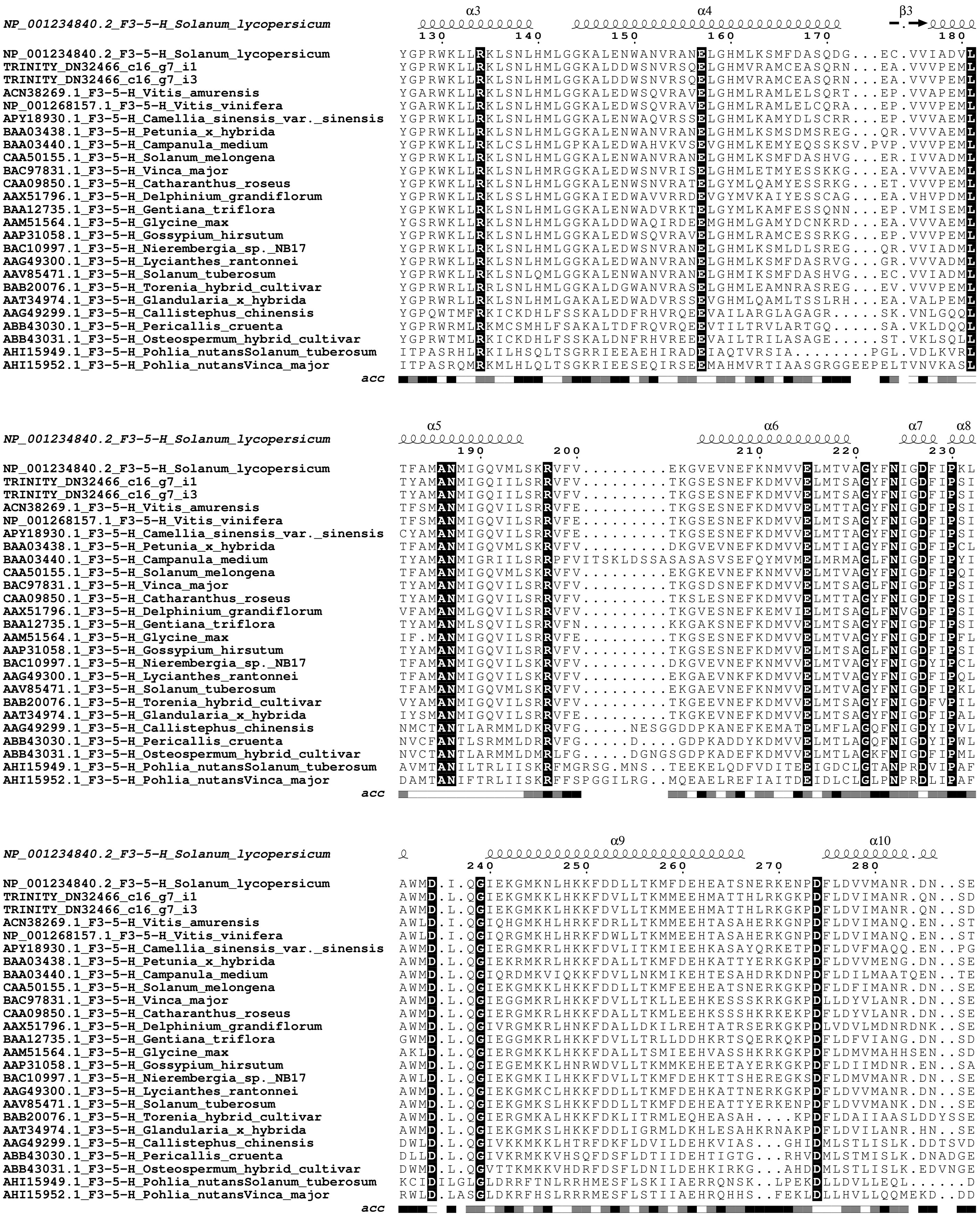
NP_001234840.2_F3-5-H_Solanum_lycopersicum
α3 α4 β3
130 140 150 160 170 180
NP_001234840.2_F3-5-H_Solanum_lycopersicum YGPRWKLLRKLSNLHMLGGKALENWANVRANELGHMLKSMFDASQDG...EC.VVIADVL
TRINITY_DN32466_c16_g7_i1 YGPRWKLLRKLSNLHMLGGKALDDWSNVRSQELGHMVRAMCEASQRN...EA.VVVPEML
TRINITY_DN32466_c16_g7_i3 YGPRWKLLRKLSNLHMLGGKALDDWSNVRSQELGHMVRAMCEASQRN...EA.VVVPEML
ACN38269.1_F3-5-H_Vitis_amurensis YGARWKLLRKLSNLHMLGGKALEDWSQVRAVELGHMLRAMLELSQRT...EP.VVAPEML
NP_001268157.1_F3-5-H_Vitis_vinifera YGARWKLLRKLSNLHMLGGKALEDWSQVRAVELGHMLRAMLELCQRA...EP.VVVPEML
APY18930.1_F3-5-H_Camellia_sinensis_var._sinensis YGPRWKLLRKLSNLHMLGGKALENWAQVRSSELGHMLKAMYDLSCRR...EP.VVVAEML
BAA03438.1_F3-5-H_Petunia_x_hybrida YGPRWKLLRKLSNLHMLGGKALENWANVRANELGHMLKSMSDMSREG...QR.VVVAEML
BAA03440.1_F3-5-H_Campanula_medium YGPKWKLLRKLCSLHMLGPKALEDWAHVKVSEVGHMLKEMYEQSSKSV.PVP.VVVPEML
CAA50155.1_F3-5-H_Solanum_melongena YGPRWKLLRKLSNLHMLGGKALENWANVRANELGHMLKSMFDASHVG...ER.IVVADML
BAC97831.1_F3-5-H_Vinca_major YGPKWKLLRKLSNLHMRGGKALENWSNVRISELGHMLETMYESSKKG...EA.VVVAEML
CAA09850.1_F3-5-H_Catharanthus_roseus YGPKWKLLRKLSNLHMLGGKALENWSNVRATELGYMLQAMYESSRKG...ET.VVVPEML
AAX51796.1_F3-5-H_Delphinium_grandiflorum YGPRWKLLRKLSSLHMLGGKAIEDWAVVRRDEVGYMVKAIYESSCAG...EA.VHVPDML
BAA12735.1_F3-5-H_Gentiana_triflora YGPKWKLLRKLSNLHMLGGKALENWADVRKTELGYMLKAMYESSQNN...EP.VMISEML
AAM51564.1_F3-5-H_Glycine_max YGSRWKLLRKLSNLHMLGGKALDDWAQIRDEEMGHMLGAMYDCNKRD...EA.VVVAEML
AAP31058.1_F3-5-H_Gossypium_hirsutum YGPRWKLLRKLSNLHMLGGKALEDWSQVRAVELGHMLRAMCESSRKG...EP.VVVPEML
BAC10997.1_F3-5-H_Nierembergia_sp._NB17 YGPRWKLLRKLSNLHMLGGKALEDWSNVRANELGHMLKSMLDLSREG...QR.VVIADML
AAG49300.1_F3-5-H_Lycianthes_rantonnei YGPRWKLLRKLSNLHMLGGKALEDWANVRANELGHMLKSMFDASRVG...GR.VVVADML
AAV85471.1_F3-5-H_Solanum_tuberosum YGPRWKLLRKLSNLQMLGGKALENWANVRANELGHMIKSMFDASHVG...EC.VVIADML
BAB20076.1_F3-5-H_Torenia_hybrid_cultivar YGPRWRLLRRLSNLHMLGGKALDGWANVRASELGHMLEAMNRASREG...EV.VVVPEML
AAT34974.1_F3-5-H_Glandularia_x_hybrida YGPRWRLLRKLSNLHMLGGKALEDWADVRSSEVGHMLQAMLTSSLRH...EA.VALPEML
AAG49299.1_F3-5-H_Callistephus_chinensis YGPQWTMFRKICKDHLFSSKALDDFRHVRQEEVAILARGLAGAGR.....SK.VNLGQQL
ABB43030.1_F3-5-H_Pericallis_cruenta YGPRWRMLRKMCSMHLFSAKALTDFRQVRQEEVTILTRVLARTGQ.....SA.VKLDQQL
ABB43031.1_F3-5-H_Osteospermum_hybrid_cultivar YGPRWTMLRKICKDHLFSSKALDNFRHVREEEVAILTRILASAGE.....ST.VKLSQLL
AHI15949.1_F3-5-H_Pohlia_nutansSolanum_tuberosum ITPASRHLRKILHSQLTSGRRIEEAEHIRADEIAQTVRSIA........PGL.VDLKVRL
AHI15952.1_F3-5-H_Pohlia_nutansVinca_major ITPASRQMRKMLHLQLTSGKRIEESEQIRSEEMAHMVRTIAASGRGGEEPELTVNVKASL
acc
NP_001234840.2_F3-5-H_Solanum_lycopersicum
α5 α6 α7 α8
190 200 210 220 230
NP_001234840.2_F3-5-H_Solanum_lycopersicum TFAMANMIGQVMLSKRVFV.........EKGVEVNEFKNMVVELMTVAGYFNIGDFIPKL
TRINITY_DN32466_c16_g7_i1 TYAMANMIGQIILSRRVFV.........TKGSESNEFKDMVVELMTSAGYFNIGDFIPSI
TRINITY_DN32466_c16_g7_i3 TYAMANMIGQIILSRRVFV.........TKGSESNEFKDMVVELMTSAGYFNIGDFIPSI
ACN38269.1_F3-5-H_Vitis_amurensis TFSMANMIGQVILSRRVFE.........TKGSESNEFKDMVVELMTTAGYFNIGDFIPSI
NP_001268157.1_F3-5-H_Vitis_vinifera TFSMANMIGQVILSRRVFE.........TKGSESNEFKDMVVELMTSAGYFNIGDFIPSI
APY18930.1_F3-5-H_Camellia_sinensis_var._sinensis CYAMANMIGQVILSRRVFE.........TKGSESNEFKDMVVELMTTAGYFNIGDFIPSI
BAA03438.1_F3-5-H_Petunia_x_hybrida TFAMANMIGQVILSKRVFV.........DKGVEVNEFKDMVVELMTIAGYFNIGDFIPCL
BAA03440.1_F3-5-H_Campanula_medium TYAMANMIGRIILSRRPFVITSKLDSSASASASVSEFQYMVMELMRMAGLFNIGDFIPYI
CAA50155.1_F3-5-H_Solanum_melongena TFAMANMIGQVMLSKRVFV.........EKGKEVNEFKNMVVELMTVAGYFNIGDFIPQI
BAC97831.1_F3-5-H_Vinca_major TYAMANMIGQVILSRRVFV.........TKGSDSNEFKDMVVELMTSAGYFNIGDFIPSI
CAA09850.1_F3-5-H_Catharanthus_roseus TYAMANMIGQVILSRRVFV.........TKSLESNEFKDMVVELMTTAGYFNIGDFIPSI
AAX51796.1_F3-5-H_Delphinium_grandiflorum VFAMANMLGQVILSRRVFV.........TKGVESNEFKEMVIELMTSAGLFNVGDFIPSI
BAA12735.1_F3-5-H_Gentiana_triflora TYAMANMIGQVILSRRVFN.........KKGAKSNEFKDMVVELMTSAGYFNIGDFIPSI
AAM51564.1_F3-5-H_Glycine_max IF.MANMIGQVILSRRVFE.........TKGSESNEFKDMVVELMTVAGYFNIGDFIPFL
AAP31058.1_F3-5-H_Gossypium_hirsutum TYAMANMIGQVILSRRVFV.........TKGSESNEFKDMVVELMTSAGLFNIGDFIPSI
BAC10997.1_F3-5-H_Nierembergia_sp._NB17 TFAMANMIGQVILSKRVFV.........DKGVEVNEFKDMVVELMTSAGYFNIGDYIPCL
AAG49300.1_F3-5-H_Lycianthes_rantonnei TFAMANMIGQVILSKRVFV.........EKGAEVNKFKDMVVELMTVAGYFNIGDFIPQL
AAV85471.1_F3-5-H_Solanum_tuberosum TFAMANMIGQVMLSKRVFV.........EKGAEVNEFKNMVVELMTVAGYFNIGDFIPKL
BAB20076.1_F3-5-H_Torenia_hybrid_cultivar VYAMANMIGQVILSRRVFV.........TKGEEVNEFKDMVVELMTSAGYFNIGDFVPIL
AAT34974.1_F3-5-H_Glandularia_x_hybrida IYSMANMIGQVILSRRVFE.........TKGKEVNEFKDMVVELMTSAGYFNIGDFIPAL
AAG49299.1_F3-5-H_Callistephus_chinensis NMCTANTLARMMLDKRVFG.....NESGGDDPKANEFKEMATELMFLAGQFNIGDYIPVL
ABB43030.1_F3-5-H_Pericallis_cruenta NVCFANTLSRMMLDRRVFG.....D....GDPKADDYKDMVVELMTLAGQFNIGDYIPWL
ABB43031.1_F3-5-H_Osteospermum_hybrid_cultivar NVCTANTLARMMLDMRLFG.....DGNGSGDPKADEFKDMVVELMTLAGKFNIGDFIPML
AHI15949.1_F3-5-H_Pohlia_nutansSolanum_tuberosum AVMTANILTRLIISKRFMGRSG.MNS..TEEKELQDFVDITEEIGDCLGTANPRDVIPAF
AHI15952.1_F3-5-H_Pohlia_nutansVinca_major DAMTANIFTRLIISKRFFSPGGILRG..MQEAELREFIAITDEIDLCLGLPNPRDLIPAF
acc
NP_001234840.2_F3-5-H_Solanum_lycopersicum
α9 α10
240 250 260 270 280
NP_001234840.2_F3-5-H_Solanum_lycopersicum AWMD.I.QGIEKGMKNLHKKFDDLLTKMFDEHEATSNERKENPDFLDVVMANR.DN..SE
TRINITY_DN32466_c16_g7_i1 AWMD.L.QGIEKGMKKLHKKFDVLLTKMMEEHMATTHLRKGKPDFLDVIMANR.QN..SD
TRINITY_DN32466_c16_g7_i3 AWMD.L.QGIEKGMKKLHKKFDVLLTKMMEEHMATTHLRKGKPDFLDVIMANR.QN..SD
ACN38269.1_F3-5-H_Vitis_amurensis AWLD.I.QGIQHGMKHLHRKFDRLLTKMMEEHTASAHERKGNPDFLDVIMANQ.EN..ST
NP_001268157.1_F3-5-H_Vitis_vinifera AWLD.I.QGIQRGMKHLHRKFDWLLTKMMEEHTASAHERKGNPDFLDVIMANQ.EN..ST
APY18930.1_F3-5-H_Camellia_sinensis_var._sinensis AWMD.L.QGIERGMKKLHKKFDVLITKMIEEHKASAYQRKETPDFLDVFMAQQ.EN..PG
BAA03438.1_F3-5-H_Petunia_x_hybrida AWMD.L.QGIEKRMKRLHKKFDALLTKMFDEHKATTYERKGKPDFLDVVMENG.DN..SE
BAA03440.1_F3-5-H_Campanula_medium AWMD.L.QGIQRDMKVIQKKFDVLLNKMIKEHTESAHDRKDNPDFLDILMAATQEN..TE
CAA50155.1_F3-5-H_Solanum_melongena AWMD.L.QGIEKGMKKLHKKFDDLLTKMFEEHEATSNERKGKPDFLDFIMANR.DN..SE
BAC97831.1_F3-5-H_Vinca_major AWMD.L.QGIEGGMKRLHKKFDVLLTKLLEEHKESSSKRKGKPDLLDYVLANR.DN..SE
CAA09850.1_F3-5-H_Catharanthus_roseus AWMD.L.QGIERGMKRLHKKFDALLTKMLEEHKSSSHKRKEKPDFLDYVLANR.DN..SE
AAX51796.1_F3-5-H_Delphinium_grandiflorum AWMD.L.QGIVRGMKRLHNKFDALLDKILREHTATRSERKGKPDLVDVLMDNRDNK..SE
BAA12735.1_F3-5-H_Gentiana_triflora GWMD.L.QGIEGGMKRLHKKFDVLLTRLLDDHKRTSQERKQKPDFLDFVIANG.DN..SD
AAM51564.1_F3-5-H_Glycine_max AKLD.L.QGIERGMKKLHKKFDALLTSMIEEHVASSHKRKGKPDFLDMVMAHHSEN..SD
AAP31058.1_F3-5-H_Gossypium_hirsutum AWMD.L.QGIEGEMKKLHNRWDVLLTKMMKEHEETAYERKGKPDFLDIIMDNR.EN..SA
BAC10997.1_F3-5-H_Nierembergia_sp._NB17 AWLD.L.QGIEKGMKILHKKFDVLLTKMFDEHKTTSHEREGKSDFLDMVMANR.DN..SE
AAG49300.1_F3-5-H_Lycianthes_rantonnei AWMD.L.QGIEKGMKCLHKKFDDLLTKMFDEHKATTNERKGKPDFLDVVMANR.DN..SE
AAV85471.1_F3-5-H_Solanum_tuberosum AWMD.L.QGIEKGMKSLHKKFDDLLTKMFDEHEATTYERKENPDFLDVVMANK.DN..SE
BAB20076.1_F3-5-H_Torenia_hybrid_cultivar AWMD.L.QGIERGMKALHKKFDKLITRMLEQHEASAH....KPDFLDAIIAASLDDYSSE
AAT34974.1_F3-5-H_Glandularia_x_hybrida AWMD.L.QGIEKGMKSLHKKFDDLIGRMLDKHLESAHKRNAKPDFLDVILANR.HN..SE
AAG49299.1_F3-5-H_Callistephus_chinensis DWLD.L.QGIVKKMKKLHTRFDKFLDVILDEHKVIAS...GHIDMLSTLISLK.DDTSVD
ABB43030.1_F3-5-H_Pericallis_cruenta DLLD.L.QGIVKRMKKVHSQFDSFLDTIIDEHTIGTG...RHVDMLSTMISLK.DNADGE
ABB43031.1_F3-5-H_Osteospermum_hybrid_cultivar DWMD.L.QGVTTKMKKVHDRFDSFLDNILDEHKIRKG...AHDDMLSTLISLK.EDVNGE
AHI15949.1_F3-5-H_Pohlia_nutansSolanum_tuberosum KCIDILGLGLDRRFTNLRRHMESFLSKIIAERRQNSK..LPEKDLLDVLLEQL......D
AHI15952.1_F3-5-H_Pohlia_nutansVinca_major RWLD.LASGLDKRFHSLRRRMESFLSTIIAEHRQHHS..FEKLDLLHVLLQQMEKD..DD
acc

```
                                                             α11                              α12
NP_001234840.2_F3-5-H_Solanum_lycopersicum
                                                    290       300       310       320       330       340
NP_001234840.2_F3-5-H_Solanum_lycopersicum          GERLSTTNIKALLLNLFTAGTDTSSSVIEWALAEMMKNPKIFEKAQQEMDQVIGKNRRLI
TRINITY_DN32466_c16_g7_i1                           GERLSVTNVKALLLNLFTAGTDTSSSIIEWSLAEMLKNPNIMKRAHAEMDQVIGRNRQLE
TRINITY_DN32466_c16_g7_i3                           GERLSVTNVKALLLNLFTAGTDTSSSIIEWSLAEMLKNPNIMKRAHAEMDQVIGRNRQLE
ACN38269.1_F3-5-H_Vitis_amurensis                   GEKLTITNIKALLLNLFTAGTDTSSSVIEWSLAEMLKNPSILKRAHEEMDQVIGRSRRLV
NP_001268157.1_F3-5-H_Vitis_vinifera                GEKLTITNIKALLLNLFTAGTDTSSSVIEWSLAEMLKNPSILKRAHEEMDQVIGRSRRLV
APY18930.1_F3-5-H_Camellia_sinensis_var._sinensis   EEKLNTTNIKALLLNLFTAGTDTSSSIIEWALAEMLKDPKILNRAHDEMDRVIGRNRRLQ
BAA03438.1_F3-5-H_Petunia_x_hybrida                 GERLSTTNIKALLLNLFTAGTDTSSSAIEWALAEMMKNPAILKKAQAEMDQVIGRNRRLL
BAA03440.1_F3-5-H_Campanula_medium                  GIQLNLVNVKALLLDLFTAGTDTSSSVIEWALAEMLNHRQILNRAHEEMDQVIGRNRRLE
CAA50155.1_F3-5-H_Solanum_melongena                 GERLSITNIKALLLNLFTAGTDTSSSVIEWALTEMMKNPTIFKKAQQEMDQIIGKNRRFI
BAC97831.1_F3-5-H_Vinca_major                       GERLTTTNIKALLLNLFSAGTDTSSSIIEWALSEMLRNPSILKRAQHEMVQVIGRNRRLV
CAA09850.1_F3-5-H_Catharanthus_roseus               GERLTTTNIKALLLNLFTAGTDTSSSVIEWAISEMLKNPTILKRAQEEMDQVIGRNRRLM
AAX51796.1_F3-5-H_Delphinium_grandiflorum           QERLTDTNIKALLLNLFSAGTDTSSSTIEWALTEMIKNPSIFRRAHAEMDQVIGRNRRLE
BAA12735.1_F3-5-H_Gentiana_triflora                 GERLNTDNIKALLLNLFTAGTDTSSSIIEWALAELLKNRTLLTRAQDEMDRVIGRDRRLL
AAM51564.1_F3-5-H_Glycine_max                       GEELSLTNIKALLLNLFTAGTDTSSSIIEWSLAEMLKKPSIMKKAHEEMDQVIGRDRRLK
AAP31058.1_F3-5-H_Gossypium_hirsutum                GERLSLTNVKALLLNLFTAGTDTSSSIIEWALAEILKNPKILNKAHEEMDKVIGRNRRLE
BAC10997.1_F3-5-H_Nierembergia_sp._NB17             GERLSTTNIKALLLNLFTAGTDTSSSVIEWALTEMLKNPSILKKAQVEMDQVIGKNRRLL
AAG49300.1_F3-5-H_Lycianthes_rantonnei              GERLSTNNIKALLLNLFTAGTDTSSSAIEWALAEMMKNPQILKKVQQEMDQIIGKNRRLI
AAV85471.1_F3-5-H_Solanum_tuberosum                 GERLSTTNIKALLLNLFTAGTDTSSSVIEWALAEMMKNPKIFKKAQEEMDQVIGKNRRLI
BAB20076.1_F3-5-H_Torenia_hybrid_cultivar           EERLSTSNIKALLLNLFTAGTDTSSSTIEWALSEMLKKGKILKRAQEEMDRVVGRERRLV
AAT34974.1_F3-5-H_Glandularia_x_hybrida             GESLTTTNIKALLLNLFTAGTDTSSSTIEWALAEMIKNPTILKTAHNEMDRVIGRDRRLL
AAG49299.1_F3-5-H_Callistephus_chinensis            GRKPSDIEIKALLLELFVAGTDTSSNTVEWAIAELIRQPHLLKRAQEEMDSVVGQNRLVT
ABB43030.1_F3-5-H_Pericallis_cruenta                GGKLSFIEIKALLLNLFSAGTDTSSSTVEWGIAELIRHPQLMKQAQEEMDIVVGKNRLVT
ABB43031.1_F3-5-H_Osteospermum_hybrid_cultivar      GGKLSDIEIKALLLNLFSAGTDTSSSSIEWTMAELIRNPQLLKQAQEEMDIVVGRDRLVT
AHI15949.1_F3-5-H_Pohlia_nutansSolanum_tuberosum    HHEITEDVVISIIWEAFAAGMETTVLATEWTMAEVLRSPQIQAQAQAELDDVVGHSRPVQ
AHI15952.1_F3-5-H_Pohlia_nutansVinca_major          GQEITEPQVTSIVWEAFAAGMETSALATEWAMAEILRNPHILKKAQSEVDAVVGKDRRVR
                                                acc
```

```
                                                      η4       α13          TT     β4      β5      TT   β6  TT  β7    α14
NP_001234840.2_F3-5-H_Solanum_lycopersicum
                                                    350       360       370       380       390       400
NP_001234840.2_F3-5-H_Solanum_lycopersicum          ESDIPNLPYLRAICKETFRKHPSTPLNLPRVSSEPC.....TVDGYYIPKNTRLSVNIWA
TRINITY_DN32466_c16_g7_i1                           ESDIPKLPYLQAICKETFRKHPSTPLNLPRVSTQAC.....EVNGYYIPKNTRLSVNIWA
TRINITY_DN32466_c16_g7_i3                           ESDIPKLPYLQAICKETFRKHPSTPLNLPRVSTQAC.....EVNGYYIPKNTRLSVNIWA
ACN38269.1_F3-5-H_Vitis_amurensis                   ESDLPKLPYLQAICKESFRKHPSTPLNLPRVSTQAC.....EVNGYYIPKNTRLSVNIRA
NP_001268157.1_F3-5-H_Vitis_vinifera                ESDLPKLPYLQAICKESFRKHPSTPLNLPRVSTQAC.....EVNGYYIPKNTRLSVNIWA
APY18930.1_F3-5-H_Camellia_sinensis_var._sinensis   ESDLPKLPYLQAICKETFRMHPSTPLNLPRVSAQAC.....RVNGYYIPKNTRLSVNIWA
BAA03438.1_F3-5-H_Petunia_x_hybrida                 ESDIPNLPYLRAICKETFRKHPSTPLNLPRISNEPC.....IVDGYYIPKNTRLSVNIWA
BAA03440.1_F3-5-H_Campanula_medium                  QSDIPNLPYFQAICKETFRKHPSTPLNLPRISTEAC.....EVDGFHIPKNTRLIVNIWA
CAA50155.1_F3-5-H_Solanum_melongena                 ESDIPNLPYLRAICKEAFRKHPSTPLNLPRVSSDAC.....TIDGYYIPKNTRLSVNIWA
BAC97831.1_F3-5-H_Vinca_major                       ESDISRLPYLQAICKETFRKHPSTPLNLPRIATEAC.....EVNGYYIPKGTRLSVNIWA
CAA09850.1_F3-5-H_Catharanthus_roseus               ESDIPKLPYLQAICKETFRKHPSTPLNLPRIAQKDC.....QVNGYYIPKGTRLSVNIWA
AAX51796.1_F3-5-H_Delphinium_grandiflorum           ESDIPKLPYLQAVCKETFRKHPSTPLNLPRVAIEPC.....EVEGYHIPKGTRLSVNIWA
BAA12735.1_F3-5-H_Gentiana_triflora                 ESDIPNLPYLQAICKETFRKHPSTPLNLPR....NCIRGHVDVNGYYIPKGTRLNVNIWA
AAM51564.1_F3-5-H_Glycine_max                       ESDIPKLPYFQAICKETYRKHPSTPLNLPRISSEPC.....QVNGYYIPENTRLNVNIWA
AAP31058.1_F3-5-H_Gossypium_hirsutum                ESDIPKLPYLQAICKETFRKHPSTPLNLPRVSTQAC.....EINGYYIPKNTRLSVNIWA
BAC10997.1_F3-5-H_Nierembergia_sp._NB17             ESDISNLPYLRAICKETFRKHPSTPLNLPRISSESC.....EVDGYYIPKNTRLSVNIWA
AAG49300.1_F3-5-H_Lycianthes_rantonnei              ESDIPNLPYLRAVCKETFRKHPSTPLNLPRISNEPC.....MVDGYYIPKNIRLSVNIWA
AAV85471.1_F3-5-H_Solanum_tuberosum                 ESDIPNLPYLRAICKETFRKHPSTPLNLPRVSTEPC.....TVDGYYIPKNTRLSVNIWA
BAB20076.1_F3-5-H_Torenia_hybrid_cultivar           ESDIEKLGYLKAICKETFRKHPSTPLNLPRISSEAC.....VVNGHYIPKGTRLSVNIWA
AAT34974.1_F3-5-H_Glandularia_x_hybrida             ESDLPKLPYLQAICKEAYRKHPSTPLNLPRISSQPC.....IVNGYYIPENTRLSVNIWA
AAG49299.1_F3-5-H_Callistephus_chinensis            EMDLSQLTFLQAIVKEAFRLHPSTPLSLPRIASESC.....EVDGYYIPKGSTLLVNIWA
ABB43030.1_F3-5-H_Pericallis_cruenta                EMDISQLTFLQAIVKETFRLHPATPLSLPRIASESC.....EVKGYHVPKGSILFVNVWA
ABB43031.1_F3-5-H_Osteospermum_hybrid_cultivar      ESDLTQLTFLHAIVKESFRLHPSTPLSLPRIASESC.....EVNGYHIPKGSTLLVNIWA
AHI15949.1_F3-5-H_Pohlia_nutansSolanum_tuberosum    ESDLPNLKYIRAIVKESFRLHPIIPLLIPHHSSHAC.....SACGYHIPAHTNLLVNVWA
AHI15952.1_F3-5-H_Pohlia_nutansVinca_major          ESDIPMLKYIKAIVKEALRLHPIIPLLVPHQSNVAC.....KALGYDIPTKTQLLVNVWA
                                                acc
```

```
                                                                       η5                                    α15
                                                                 TT            TTT  TT           TT
NP_001234840.2_F3-5-H_Solanum_lycopersicum
                                                          410       420       430       440       450       460
NP_001234840.2_F3-5-H_Solanum_lycopersicum          IGRDPDVW.ENPLEFTPERFL.SGKNAKIEPRGNDFELIPFGAGRRICAGTRMGIVMVEY
TRINITY_DN32466_c16_g7_i1                           IGRDPNVW.DNPLDFTPERFL.TEKYKKIDPRGNDFELIPFGAGRRICAGTRMGIVLVEY
TRINITY_DN32466_c16_g7_i3                           IGRDPNVW.DNPLDFTPERFL.TEKYKKIDPRGNDFELIPFGAGRRICAGTRMGIVLVEY
ACN38269.1_F3-5-H_Vitis_amurensis                   IGRDPDVW.ESPEEFRPERFL.SGRNAKIDPRGNDFELIPFGAGRRICAGTRMGIVLVEY
NP_001268157.1_F3-5-H_Vitis_vinifera                IGRDPDVW.ESPEEFRPERFL.SGRNTKIDPRGNDFELIPFGAGRRICAGTRMGIVLVEY
APY18930.1_F3-5-H_Camellia_sinensis_var._sinensis   IGRDPDVW.ERPLEFIPDRFL.SGKNAKTDPRGNDFELIPFGAGRRICAGTRMGIVLVEY
BAA03438.1_F3-5-H_Petunia_x_hybrida                 IGRDPQVW.ENPLEFNPERFL.SGRNSKIDPRGNDFELIPFGAGRRICAGTRMGIVMVEY
BAA03440.1_F3-5-H_Campanula_medium                  IGRDPKVW.ENPLDFTPERFL.SEKHAKIDPRGNHFELIPFGAGRRICAGARMGAASVEY
CAA50155.1_F3-5-H_Solanum_melongena                 IGRDPDVW.ENPLEFIPERFL.SEKNAKIEHRGNDFELIPFGAGRRICAGTRMGIVMVEY
BAC97831.1_F3-5-H_Vinca_major                       IGRDPDVW.ENPLEFNPDRFL.SGKNAKIDPRGNDFELIPFGAGRRICAGTRMGILLVEY
CAA09850.1_F3-5-H_Catharanthus_roseus               IGRDPNVW.ENPLEFNPDRFL.SGKMAKIEPRGNDFELIPFGAGRRICAGTRMGIVLVEY
AAX51796.1_F3-5-H_Delphinium_grandiflorum           IGRDPNVW.ENPLEFNPDRFL.TGKMAKIDPRGNNSELIPFGAGRRICAGTRMGIVLVEY
BAA12735.1_F3-5-H_Gentiana_triflora                 IGRDPSVWGDNPNEFDPERFL.YGRNAKIDPRGNHFELIPFGAGRRICAGTRMGILLVEY
AAM51564.1_F3-5-H_Glycine_max                       IGRDPDVW.NNPLEFMPERFL.SGKNAKIDPRGNDFELIPFGAGRRICAGTRMGIVLVHY
AAP31058.1_F3-5-H_Gossypium_hirsutum                IGRDPDVW.GNPLDFTPERFL.SGRFAKIDPRGNDFELIPFGAGRRICAGTRMGIVLVEY
BAC10997.1_F3-5-H_Nierembergia_sp._NB17             IGRDPDVW.ENPLKFNPERFL.SGKYAKIDPRGNDFELIPFGAGRRICAGTRMGIVMVEY
AAG49300.1_F3-5-H_Lycianthes_rantonnei              IGRDPDVW.ENPLEFNPERFL.SGKNVKIDPRGNDFELIPFGAGRRICAGTRMGIVMVEY
AAV85471.1_F3-5-H_Solanum_tuberosum                 IGRDPDVW.ENPLEFNPERFL.SGKNAKIEPRGNDFELIPFGAGRRICAGTRMGIVMVEY
BAB20076.1_F3-5-H_Torenia_hybrid_cultivar           IGRDPEVW.EDPLEFNPDRFL....HSKMDPRGNDFELMPFGAGRRICAGTRMGIVLVEY
AAT34974.1_F3-5-H_Glandularia_x_hybrida             IGRDPDVW.ENPLDFNPDRFL.SGKNAKIDPRGNNFELIPFGAGRRICAGARMAMVLVEY
AAG49299.1_F3-5-H_Callistephus_chinensis            IGRHPEVW.TDPLEFRPTRFLPGGEKPGIVVKVNDFEVLPFGAGRRICAGMSLALRTVQL
ABB43030.1_F3-5-H_Pericallis_cruenta                IARQSELW.TDPLEFRPGRFLIPGEKPNVEVKPNDFEIVPFGGGRRICAGMSLGLRMVNL
ABB43031.1_F3-5-H_Osteospermum_hybrid_cultivar      IGRHPEVW.ADPLEFRPARFLPGGEKPGVNVKVNDFEVLPFGAGRRICAGMSLALKMVHL
AHI15949.1_F3-5-H_Pohlia_nutansSolanum_tuberosum    IGRDPSVF.TNPLEFQPERFLPEGPHAGTEWT.KGFDLLIFGFGRRACMGMSLGALLVET
AHI15952.1_F3-5-H_Pohlia_nutansVinca_major          IGRDPTVW.TNPLTFDPERFLDDGAHADTDFSGKEFNLLPFGSGRRMCMGMSLGVLLVET
                                                acc
```

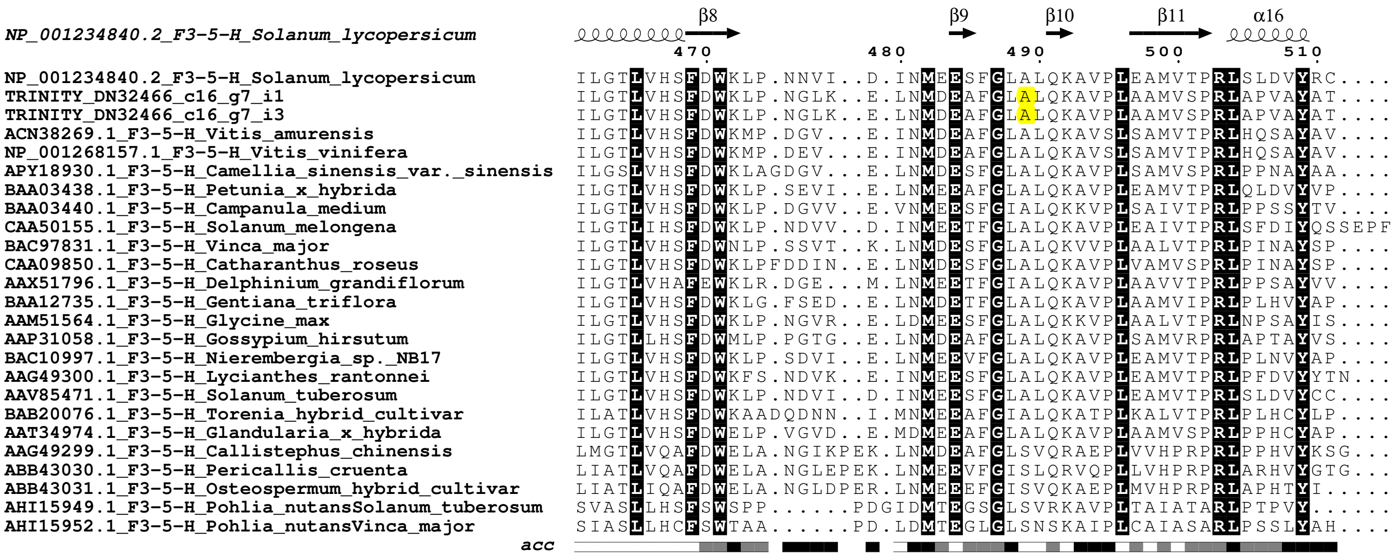

NP_001234840.2_F3-5-H_Solanum_lycopersicum
β8
β9
β10
β11
α16
470
480
490
500
510
NP_001234840.2_F3-5-H_Solanum_lycopersicum ILGTLVHSFDWKLP.NNVI..D.INMEESFGLALQKAVPLEAMVTPRLSLDVYRC....
TRINITY_DN32466_c16_g7_i1 ILGTLVHSFDWKLP.NGLK..E.LNMDEAFGLALQKAVPLAAMVSPRLAPVAYAT....
TRINITY_DN32466_c16_g7_i3 ILGTLVHSFDWKLP.NGLK..E.LNMDEAFGLALQKAVPLAAMVSPRLAPVAYAT....
ACN38269.1_F3-5-H_Vitis_amurensis ILGTLVHSFDWKMP.DGV...E.INMDEAFGLALQKAVSLSAMVTPRLHQSAYAV....
NP_001268157.1_F3-5-H_Vitis_vinifera ILGTLVHSFDWKMP.DEV...E.INMDEAFGLALQKAVSLSAMVTPRLHQSAYAV....
APY18930.1_F3-5-H_Camellia_sinensis_var._sinensis ILGSLVHSFDWKLAGDGV...E.LNMDESFGLALQKAVPLSAMVSPRLPPNAYAA....
BAA03438.1_F3-5-H_Petunia_x_hybrida ILGTLVHSFDWKLP.SEVI..E.LNMEEAFGLALQKAVPLEAMVTPRLQLDVYVP....
BAA03440.1_F3-5-H_Campanula_medium ILGTLVHSFDWKLP.DGVV..E.VNMEESFGIALQKKVPLSAIVTPRLPPSSYTV....
CAA50155.1_F3-5-H_Solanum_melongena ILGTLIHSFDWKLP.NDVV..D.INMEETFGLALQKAVPLEAIVTPRLSFDIYQSSEPF
BAC97831.1_F3-5-H_Vinca_major ILGTLVHSFDWNLP.SSVT..K.LNMDESFGLALQKVVPLAALVTPRLPINAYSP....
CAA09850.1_F3-5-H_Catharanthus_roseus ILGTLVHSFDWKLPFDDIN..E.LNMDESFGLALQKAVPLVAMVSPRLPINAYSP....
AAX51796.1_F3-5-H_Delphinium_grandiflorum ILGTLVHAFEWKLR.DGE...M.LNMEETFGIALQKAVPLAAVVTPRLPPSAYVV....
BAA12735.1_F3-5-H_Gentiana_triflora ILGTLVHSFDWKLG.FSED..E.LNMDETFGLALQKAVPLAAMVIPRLPLHVYAP....
AAM51564.1_F3-5-H_Glycine_max ILGTLVHSFDWKLP.NGVR..E.LDMEESFGLALQKKVPLAALVTPRLNPSAYIS....
AAP31058.1_F3-5-H_Gossypium_hirsutum ILGTLLHSFDWMLP.PGTG..E.LNMDEAFGLALQKAVPLSAMVRPRLAPTAYVS....
BAC10997.1_F3-5-H_Nierembergia_sp._NB17 ILGTLVHSFDWKLP.SDVI..E.LNMEEVFGLALQKAVPLEAMVTPRLPLNVYAP....
AAG49300.1_F3-5-H_Lycianthes_rantonnei ILGTLVHSFDWKFS.NDVK..E.INMEESFGLALQKAVPLEAMVTPRLPFDVYYTN...
AAV85471.1_F3-5-H_Solanum_tuberosum ILGTLVHSFDWKLP.NDVI..D.INMEESFGLALQKAVPLEAMVTPRLSLDVYCC....
BAB20076.1_F3-5-H_Torenia_hybrid_cultivar ILATLVHSFDWKAADQDNN..I.MNMEEAFGIALQKATPLKALVTPRLPLHCYLP....
AAT34974.1_F3-5-H_Glandularia_x_hybrida ILGTLVHSFDWELP.VGVD..E.MDMEEAFGLALQKAVPLAAMVSPRLPPHCYAP....
AAG49299.1_F3-5-H_Callistephus_chinensis LMGTLVQAFDWELA.NGIKPEK.LNMDEAFGLSVQRAEPLVVHPRPRLPPHVYKSG...
ABB43030.1_F3-5-H_Pericallis_cruenta LIATLVQAFDWELA.NGLEPEK.LNMEEVFGISLQRVQPLLVHPRPRLARHVYGTG...
ABB43031.1_F3-5-H_Osteospermum_hybrid_cultivar LIATLIQAFDWELA.NGLDPER.LNMEEEFGISVQKAEPLMVHPRPRLAPHTYI.....
AHI15949.1_F3-5-H_Pohlia_nutansSolanum_tuberosum SVASLLHSFSWSPP......PDGIDMTEGSGLSVRKAVPLTAIATARLPTPVY......
AHI15952.1_F3-5-H_Pohlia_nutansVinca_major SIASLLHCFSWTAA......PD.LDMTEGLGLSNSKAIPLCAIASARLPSSLYAH....
acc

```
NP_001190266.1_FLS1_Arabidopsis_thaliana         α1           η1       η2
                                                  1         10         20        30         40
NP_001190266.1_FLS1_Arabidopsis_thaliana          ............MEVE.RVQD.ISSSSLLTEAIPLEFIRSEKEQPAIT.TFR...G.PT
TRINITY_DN25915_c0_g1_i3                          ............MEVE.RVQG.IASITT..DTIPDAFIRLENEQPAIT.TVQ...G.VN
TRINITY_DN25915_c0_g2_i1                          ............MELE.RVQD.IAFASK..DTIPEAFIRLENEQPALT.TVQ...G.VD
NP_001237419.1_FLS_Glycine_max                    ............MEVL.RVQT.IASKSKD.AAIPAMFVRAETEQPGIT.TVQ...G.VN
AQR58516.1_FLS_Allium_cepa                        ............MEVE.RVQA.IATLTANLGTIPSEFIRSDHERPDLT.TYH...G.PV
AQR58515.1_FLS_Allium_cepa                        ............MEVE.RVQA.IATLTANLGTIPSEFIRSDHERPDLT.TYH...G.PV
AFA55179.1_FLS_Acacia_confusa                     ............MEVE.RVQC.LASGLL..NELPTQFIRPAHERPENTKAVE...G...
BBA27023.1_FLS_Cyclamen_purpurascens              ............MEVD.RVQA.IASLTKCQDTIPSEFIRSEHEQPANT.TVR...D.KL
BBA27024.1_FLS_Cyclamen_purpurascens              ..............ME.TVQV.LASHAKCHDTIPPEFIRPERERPAIT.TVR...S.KA
AAF64168.1_FLS_Eustoma_exaltatum_subsp._russ      ............MEVQ.RVQE.IASLSKVIDTIPAEYIRSENEQPVIS.TVH...G.VV
BAK09226.1_FLS_Gentiana_triflora                  ............MEEK.RVQE.IS..SNVLDTIPAEYIRSEKEQPAIT.TIH...G.VV
ACY00393.1_FLS_Ginkgo_biloba                      ............MAPT.RVQY.VAESRP..QTIPLEFVRPVEERPINT.TFNDDIG.LG
AEC33116.1_FLS_Fagopyrum_tataricum                ............MEVE.RVQA.IATLSRSVDTIPLEYIRSEKEQPAIT.TFQ...G.SV
ACF84961.1_FLS_Zea_mays                           ..........MGGETHLSVQE....LAASLGALPPEFVRSEQDQPGAT.TYR...GAAV
BAA36554.1_FLS_Citrus_unshiu                      ............MEVE.RVQA.IASLSHSNGTIPAEFIRPEKEQPAST.TYH...G.PA
QBQ58058.1_FLS1_Ornithogalum_longebracteatum      ............MEVE.RVQA.IASMTASLGTIPAEFIRSEHERPDIT.TYH...G.PV
QBQ58059.1_FLS2_Ornithogalum_longebracteatum      ............MEVE.RVQA.IASMTASLGTIPAEFIRSEHERPDIT.TYH...G.PV
AKJ87100.1_FLS_Vaccinium_corymbosum               ............MEVE.REQA.IAILSKFMDTIPAEFIRSETEQPAIT.TVR...G.EV
AJO70134.1_FLS_Prunus_persica                     ............MGVE.RVQDIASATSK..DTIPVEFIRSENEQPGIT.TVP...G.TV
AIS22436.1_FLS_Rosa_rugosa                        ............MGVE.RVQDIASATSK..DTIPVEFIRSENEQPGIT.TVP...G.TV
AHN19765.1_FLS_Fagopyrum_dibotrys                 ............MEVE.RVQA.IATLSRSVDTIPLEYIRSEKEQPAIT.TFQ...G.SV
AAZ78661.1_FLS_Fragaria_x_ananassa                ............MGVE.RVQDIASTISE..DTIPAEYIRSENEQPGIT.TVP...N.TV
ABM88786.1_FLS_Camellia_sinensis                  ............MEVE.RVQA.LSHVTL..HELPAKFIRPVHEQPENSKAIE...G...
QBO54037.1_flavonol_synthase_Muscari_aucheri      ............MDME.RVQA.IASLAGDLGTIPAEFIRPEHERPAMT.THH...G.PS
OsFLS_FLS_Oryza_sativa                            ...........MAECQ.SVQA....LASSLAAIPPEFVRSEHERPGAT.TFR...GGDA
AFS63900.1_FLS_Narcissus_tazetta                  ............MEFE.RVQA.IASLSASLNTIPPEFIRSEHERPDMT.TYR...G.PV
AEC33115.1_FLS_Fagopyrum_esculentum               ............MEVE.RVQA.IATLSRSVDTIPLEYIRSEKEQPAIT.TFQ...G.SV
ABE28017.1_FLS_Nicotiana_tabacum                  MKTAQAQSATTLTMEVA.RVQA.IASITKCMDTIPSEYIRSENEQPAST.TLH...G.VL
BAE75809.1_FLS_Vitis_vinifera                     ............MEIE.RVQA.IAFSSLSEGTIPPEFIRSEKEQPAIT.TFH...G.YV
AAX89401.1_FLS_Malus_domestica                    ............MGVE.SVER.ERESNE..GTIPAEFIRSENEQPGIT.TVH...G.KV
CAA80264.1_FLS_Petunia_x_hybrida                  MKTAQGVSA.TLTMEVA.RVQA.IASLSKCMDTIPSEYIRSENEQPAAT.TLH...G.VV
ADZ28516.1_FLS_Camellia_nitidissima               ............MELE.RVQV.IASLSKCIDTIPAEYIRSENEQPAIT.TIQ...G.KV
LcFLS_FLS_Litchi_chinensis                        ............MAVE.SVQA.VASICNLKDSIPAEFIRSEREQPAIT.TYH...G.AV
ABB53382.1_FLS_Antirrhinum_majus                  ............MEGE.RVQA.IASLSKYADTIPSEFIRSENEQPAAT.TLR...G.VV
AAP57395.1_FLS_Petroselinum_crispum               ............MEVE.RVQA.ISKMSRCMDTIPSEYIRSESEQPAVT.TMQ...G.VV
BAC10995.1_flavonol_synthase_Nierembergia_sp      ............MEVASRVQA.IASLIKCMDTIPSEYIRSENEQPATT.TLH...G.VE
                                              acc

NP_001190266.1_FLS1_Arabidopsis_thaliana          β1                    α2             β2           α3
                                                                50         60            70            80           90
NP_001190266.1_FLS1_Arabidopsis_thaliana          PAIPVVDLS........DPDEESVRRAVVKASEEWGLFQVVNHGIPTELIRRLQDVGRK
TRINITY_DN25915_c0_g1_i3                          LEVPVIDMK........DPDEEKTNRLMIEASEKWGMFQIINHGIPNEAIENLQKVGKE
TRINITY_DN25915_c0_g2_i1                          IGVPVIDLS........DKDQEKVNRLIVDASQKWGMFQITNHGIPNDVIRKLQNVGKE
NP_001237419.1_FLS_Glycine_max                    LEVPIIDFS........DPDEGKVVHEILEASRDWGMFQIVNHDIPSDVIRKLQSVGKM
AQR58516.1_FLS_Allium_cepa                        PELPIIDLA........NSSQENVVKQISEAAQEYGIFQLVNHGIPNEVINELQRVGKE
AQR58515.1_FLS_Allium_cepa                        PELPVIDLA........NSSQENVVKQISEAAREYGIFQLVNHGIPNEVINELQRVGKE
AFA55179.1_FLS_Acacia_confusa                     VTVPVISLCLP..........HDLLVKQIAEAASEWGVLLITDHGISPTLIARLQEVGQQ
BBA27023.1_FLS_Cyclamen_purpurascens              LEVPTIDLA........HSDEVHVVNLVAEAGREWGLFQVVNHGIPNEVISDLQRVGKY
BBA27024.1_FLS_Cyclamen_purpurascens              LEVPVIDLD........HSDETDLVRLVAEAGKEWGMFQVVNHGIPNEVISALQKAGKD
AAF64168.1_FLS_Eustoma_exaltatum_subsp._russ      LEVPVIDLS........DSDEKKIVGLVSEASKEWGIFQVVNHGIPNEVIRKLQEVGKH
BAK09226.1_FLS_Gentiana_triflora                  LEVPVIDLS........DSDEEKIVGLISQASKEWGIFQVVNHGIPNEAIAKLQEVGKE
ACY00393.1_FLS_Ginkgo_biloba                      RQIPVIDMC.....SLEAPELREKTFKEIARASKEWGIFQVINHAISPSLFESLETVGKQ
AEC33116.1_FLS_Fagopyrum_tataricum                LEVPAIDIN........ESNETSLVESIKKASEEWGLFQVVNHGIPIEVISHLQRVGKE
ACF84961.1_FLS_Zea_mays                           PDAPVIDIS...........EPGFGARMAAAAREWGLFQVVNHGVPSAAVAELQRVGRA
BAA36554.1_FLS_Citrus_unshiu                      PEIPTIDLD........DPVQDRLVRSIAEASREWGIFQVTNHGIPSDLICKLQAVGKE
QBQ58058.1_FLS1_Ornithogalum_longebracteatum      PDIPVVDLS........ATDRAGVTKAIAEAAQEWGIFQVVNHRVPIEAIKELQRVGKE
QBQ58059.1_FLS2_Ornithogalum_longebracteatum      PDIPVIDLS........DTDRAAVAKAIADAAQEWGIFQVVNHGIPVEAIKELQRVGKG
AKJ87100.1_FLS_Vaccinium_corymbosum               LEVPVIDLL.........GDEEEVVAVATAGAEWGIFQVVNHGIPEEVIGNLQRVGKE
AJO70134.1_FLS_Prunus_persica                     LECPIIDFS........DPDEEKLLKQIFEASTDWGMYQIVNHDISNEAIAKLQAVGKE
AIS22436.1_FLS_Rosa_rugosa                        LECPIIDFS........DPDEEKLLKQIFEASTDWGMYQIVNHDISNEAIAKLQAVGKE
AHN19765.1_FLS_Fagopyrum_dibotrys                 LEVPAIDIN........ELNESSLVESIKKASEEWGLFQVVNHGIPIEVISHLQRVGKE
AAZ78661.1_FLS_Fragaria_x_ananassa                LECPTIDFS........DPDEEKLLKQIFEASIDWGMYQIVNHDISNEAISKLQAVGKE
ABM88786.1_FLS_Camellia_sinensis                  VTVPVISLSQP..........HDVVVDALSKACSEWGFFLITDHGVEPSLIGRLKEVGEE
QBO54037.1_flavonol_synthase_Muscari_aucheri      PEIPVVDMT........SVDR...VKAIADAAKEWGIFQLVNHGIPVAAIEELKRVGTE
OsFLS_FLS_Oryza_sativa                            PEIPVIDMA...........APESAARVAEAAAEWGLFQVVNHGVPAAAVAELQRVGRE
AFS63900.1_FLS_Narcissus_tazetta                  PEIPVVDLS........TADRTRLTRAIADASEEWGIFQIVNHGIPVEVVKELQRVGKE
AEC33115.1_FLS_Fagopyrum_esculentum               LEVPAIDIN........ESNETSPVESIKKASEEWGLFQVVNHGIPIEVISHLQRVGKE
ABE28017.1_FLS_Nicotiana_tabacum                  LQVPVIDI...........DDKNVVKLISDASKEWGIFQVINHGIPDEVIANLQKVGKE
BAE75809.1_FLS_Vitis_vinifera                     PQVPTIDFS........DPDEANLTRLIAEASMEWGIFQIVNHGIPFHVITSLQKVGRE
AAX89401.1_FLS_Malus_domestica                    LEVPIIDFS........DPDEEKLIVQITEASSNWGMYQIVNHDIPSEVISKLQAVGKE
CAA80264.1_FLS_Petunia_x_hybrida                  LQVPVIDLR........DPDENKMVKLIADASKEWGIFQLINHGIPDEAIADLQKVGKE
ADZ28516.1_FLS_Camellia_nitidissima               LEVPVIDLS.......NDSDDQNIVQMVAEACRDWGIFQVVNHGIPDEVIGNLQRVGKD
LcFLS_FLS_Litchi_chinensis                        AEIPTIDLS........DPDTEKVERLMAEASREWGIFQVVNHGIPDDVIRKLQAVGKE
ABB53382.1_FLS_Antirrhinum_majus                  LEVPVIDLS.......DDFNEKILVKNISEASRDWGIFQVVNHGISNEVISKLQKVGKE
AAP57395.1_FLS_Petroselinum_crispum               LQVPVIDLG.......SSNNTEENLVELIAEASREWGIFQVVNHGIPDDAIAKLQKVGKE
BAC10995.1_flavonol_synthase_Nierembergia_sp      LQVPVIDLAPRVVGDEQHDHDDVEVVKQIADASKEWGIFQVINHGIPNDVIADLQKVGKE
                                              acc
```

```
NP_001190266.1_FLS1_Arabidopsis_thaliana          α4           TT      β3          T.T              β4        η3
                                                     100          110          120          130          140

NP_001190266.1_FLS1_Arabidopsis_thaliana          FFE.LPSSEKESVAKPEDSKDIEGYGTKLQKDP.EG...KKAWVDHLFHRIWPPSCVNYR
TRINITY_DN25915_c0_g1_i3                          FFE.LPQKEKEVYAKPADGKTVEGYGTSLQKEM.AG...KKGWVDHLFHRIWPPSAINYR
TRINITY_DN25915_c0_g2_i1                          FFE.LPQEEKEVYAKIPGSKSMEGYGTLISKEI.QG...KKAWSDHLFHRIWPPSAINYK
NP_001237419.1_FLS_Glycine_max                    FFE.LPQEEKELIAKPAGSDSIEGYGTKLQKEV.NG...KKGWVDHLFHIVWPPSSINYS
AQR58516.1_FLS_Allium_cepa                        FFQ.LPQEEKEVYATVPDSGSFEGYGTKLQKDL.EG...KKAWVDYLFHNVWPKHKINYK
AQR58515.1_FLS_Allium_cepa                        FFQ.LPQEEKEVYATVPDSGSFEGYGTKLQKDL.EG...KKAWVDYLFHNVWPKHKINYK
AFA55179.1_FLS_Acacia_confusa                     FFT.LPQKEKEVYANDPSTSSFEGYGTRMTKNL.DE...KVGWIDYYFHMISPPSKVNYD
BBA27023.1_FLS_Cyclamen_purpurascens              FFE.LPLEEKELCAKSGEGGSIEGYGTKLQKEV.EG...KKGWVDHLFHKIWPPSVINYK
BBA27024.1_FLS_Cyclamen_purpurascens              FFA.LPQEEKELCAKTGEGGSIEGYGTKLQKEM.DG...KKGWVDHLFHFIWPPSVINYK
AAF64168.1_FLS_Eustoma_exaltatum_subsp._russ      FFE.LPQEEKELIAKPEGSQSIEGYGTRLQKEV.DG...KKGWVDHLFHKIWPPSAINYQ
BAK09226.1_FLS_Gentiana_triflora                  FFE.LPQEEKELIAKPEGSQSIEGYGTRLQKEI.EG...KKGWVDHLFHKIWPPSAINYQ
ACY00393.1_FLS_Ginkgo_biloba                      FFQ.LPQEEKEAYACTGEDGSFTGYGTKLACTT.DG...RQGWSDFFFHMLWPPSLRDFS
AEC33116.1_FLS_Fagopyrum_tataricum                FFE.LPIEEKEEYAKPEGSKDVEGYGTKLQKEV.EG...KKGWVDHLFHRIWPPSAINYK
ACF84961.1_FLS_Zea_mays                           FFA.LPTEEKERYAMDPASGKIEGYGTKLQRDL.EG...KKTWNDFFFHVVAPPEKVDHA
BAA36554.1_FLS_Citrus_unshiu                      FFE.LPQEEKEVYSRPADAKDVQGYGTKLQKEV.EG...KKSWVDHLFHRVWPPSSINYR
QBQ58058.1_FLS1_Ornithogalum_longebracteatum      FFE.LPQEEKEAYATVPDSGSFEGYGTRLQKDL.EG...KKAWVDYLFHNVWPQSKITYR
QBQ58059.1_FLS2_Ornithogalum_longebracteatum      FFE.LPQEEKEAYATVPDSGSFEGYGTKLQKDL.GG...KKAWVDYLFHNVWPESKINYR
AKJ87100.1_FLS_Vaccinium_corymbosum               FFE.LPREEKEVYAKPKEGKSMEGYGTKLQKEV.EG...KKGWVDHLFHKVWPPSAINYQ
AJO70134.1_FLS_Prunus_persica                     FFE.LPHEEKEVYAKDPNSKSVEGYGTFLQKEL.EG...KKGWVDHLFHKIWPPSAINYC
AIS22436.1_FLS_Rosa_rugosa                        FFE.LPHEEKEVYAKDPNSKSVEGYGTFLQKEL.EG...KKGWVDHLFHKIWPPSAINYC
AHN19765.1_FLS_Fagopyrum_dibotrys                 FFE.LPIEEKEEYAKPEDSKDVEGYGTKLQKEV.EG...KKGWVDHLFHRIWPPSAINYK
AAZ78661.1_FLS_Fragaria_x_ananassa                FFE.LPQEEKEVYAKDPNSKSVEGYGTFLQKEL.EG...KKGWVDHLFHKIWPPSTINYR
ABM88786.1_FLS_Camellia_sinensis                  FFN.LPQKEKESYANDPSSGSFEGYGTKMTKNF.DE...KVEWIDYYFHVMHPPKKLNLD
QBO54037.1_flavonol_synthase_Muscari_aucheri      FFA.LPQEEKESYAAAP..GSLEGYGTRLQRES.EG...KKAWADYLFHNVWPDSSINYK
OsFLS_FLS_Oryza_sativa                            FFA.LPQEEKARYAMDASSGKMEGYGSKLQKDL.EG...KKAWADFFFHNVAPPAMVNHD
AFS63900.1_FLS_Narcissus_tazetta                  FFE.LPQGEKEAYVTGP..GSIEGYGTKLQKDL.EG...KKAWVDYLFHFVWPESRINLK
AEC33115.1_FLS_Fagopyrum_esculentum               FFE.LPIEEKEEYAKPEDSKDVEGYGTKLQKEV.EG...KKGWVDHLFHRIWPPSAINYK
ABE28017.1_FLS_Nicotiana_tabacum                  FFEVVPQEEKEVIAKTPGSQNIEGYGTSLQKEL.EG...KRGWVDHLFHKIWPPSAINYR
BAE75809.1_FLS_Vitis_vinifera                     FFE.LSPEEKELYAKPPDSKSIEGYGTKLQKEV.EG...KKAWVDHLFHKVWPPSAINYH
AAX89401.1_FLS_Malus_domestica                    FFE.LPQEEKEAYAKPPDSGSIEGYGTKLFKEISEGDTTKKGWVDNLFNKIWPPSVVNYQ
CAA80264.1_FLS_Petunia_x_hybrida                  FFEHVPQEEKELIAKTPGSNDIEGYGTSLQKEV.EG...KKGWVDHLFHKIWPPSAVNYR
ADZ28516.1_FLS_Camellia_nitidissima               FFE.LPQEEKEVYAKPPESKSVEGYGTKLQKEV.EG...KKGWVDHLFHKIWPPSAINYK
LcFLS_FLS_Litchi_chinensis                        FFE.LPQEEKEACAKIPGSQSYEGYGSKLQKEV.EG...RKSWVDHLFHIIWPPSSINYR
ABB53382.1_FLS_Antirrhinum_majus                  FFE.LPKEEKEFVAKTPES.GIEGYGTILQKEV.EG...KKGWVDHLFHKIWPPSAINYK
AAP57395.1_FLS_Petroselinum_crispum               FFE.LPQQEKEVIAKPEGYQGVEGYGTKLQKEL.GG...KKGWVDHLFHIIWPKSAVNYN
BAC10995.1_flavonol_synthase_Nierembergia_sp      FFENVPQEEKELIAKTPGSNEIEGYGTSLQKEV.EG...KKGWVDHLFHKIWPPSSINYR
                                              acc
```

```
NP_001190266.1_FLS1_Arabidopsis_thaliana                      α5                          α6     η4      β5
                                                  150        160        170        180          190        200

NP_001190266.1_FLS1_Arabidopsis_thaliana          FWPKNPPEYREVNEEYAVHVKKLSETLLGILSDGLG..LKRDALKEGLGGEMAEYMMKIN
TRINITY_DN25915_c0_g1_i3                          FWPKNPPSYRSANEEYAKYLHGAADRLFKTLSVGLE..LEENELKEGAGGDDIVYLLKIN
TRINITY_DN25915_c0_g2_i1                          FWPKNPPSYREANEEYAKHLHGIVNRLMKILSLGLG..LEENELKEAFGGEKIEYLLKIN
NP_001237419.1_FLS_Glycine_max                    FWPQNPPSYREVNEEYCKHLRGVVDKLFKSMSVGLG..LEENELKEGANEDDMHYLLKIN
AQR58516.1_FLS_Allium_cepa                        FWPRNPPAYRKANEEYTKHLQVVVDKMHSYLSLGLG..LESHVLKEAVGGDDLEYLLKIN
AQR58515.1_FLS_Allium_cepa                        FWPRNPPAYRKANEEYTKHLQVVVDKMHSYLSLGLG..LESHVLKEAVGGDDLEYLLKIN
AFA55179.1_FLS_Acacia_confusa                     MWPKNPPSYREVTEEYNKQVLKLTDKVLGLLSEGLG..LETKALKSSLGDEKIEMEMKIN
BBA27023.1_FLS_Cyclamen_purpurascens              FWPKNPPSYREANEVYAEHLRKVSRKLFKWLTLGLGPEFEGHELEEAAGGDDLVYQMKIN
BBA27024.1_FLS_Cyclamen_purpurascens              VWPKNPPSYREASEAYAGHLRKVANKLFKCLSLGLGPEFEGHELEAAVGGDDLVYLMKIN
AAF64168.1_FLS_Eustoma_exaltatum_subsp._russ      FWPKNPPAYREANEEYAKRLQLVVDNLFKYLSLGLD..LEPNSFKDGAGGDDLVYLMKIN
BAK09226.1_FLS_Gentiana_triflora                  FWPKNPPAYREANEEYAKRLHPVVDKMFMYLSLGLD..LEPNDLKNGAGGDDLVYLLKIN
ACY00393.1_FLS_Ginkgo_biloba                      KWPQKPSSYIEVTEEYSNRILGVLNKLLSALSISLE..LQESALKDALGGENLEMELKIN
AEC33116.1_FLS_Fagopyrum_tataricum                FWPKNPTSYKEANEEYCKCLRGVAEKIFRCMSLGLG..LEGPELMEAAGGDSLEYLLKIN
ACF84961.1_FLS_Zea_mays                           VWPRSLAGYREANEEYCRHMQRLTRELFEHLSLGLG..LHGGAMAEAFGGDGLVFLQKIN
BAA36554.1_FLS_Citrus_unshiu                      FWPKNPPSYRAVNEEYAKYMREVVDKLFTYLSLGLG..VEGGVLKEAAGGDDIEYMLKIN
QBQ58058.1_FLS1_Ornithogalum_longebracteatum      FWPNNPPEYRKANEEYAKYLQGVVDEMLTSLSLGLG..LESHVLKEAVGGDELEYLLKIN
QBQ58059.1_FLS2_Ornithogalum_longebracteatum      FWPNNPPEYRKANEEYAKYLQEVVDEMLSSLSVGLG..LEGHVLKEAVGGDELEYLLKIN
AKJ87100.1_FLS_Vaccinium_corymbosum               FWPKNPPSYREANEVYTEKLRAVADKLFKWLSLGLG..LDGHEVKTAVGGDDLEYMMKIN
AJO70134.1_FLS_Prunus_persica                     FWPKNPASYREANEEYAKNLHKVVEKLFKLLSLGLG..LEAQELKKAVGGDDLVYLLKIN
AIS22436.1_FLS_Rosa_rugosa                        FWPKNPASYREANEEYAKNLHKVVEKLFKLLSLGLG..LEAQELKKAVGGDDLVYLLKIN
AHN19765.1_FLS_Fagopyrum_dibotrys                 FWPKNPTSYKEANEEYCKCLRGVAEKIFRCMSLGLG..LEGPELMDAAGGDSLEYLLKIN
AAZ78661.1_FLS_Fragaria_x_ananassa                FWPKTPASYREANEEYAKNLHKVVEKLFKLLSLGLG..LEAQELKKAIGGDDLVYLLKIN
ABM88786.1_FLS_Camellia_sinensis                  MWPKNPSSYRGVTEEYNVEIMRTTNKLFELLSEGLG..LDGKVLSSSLGGDEIEFEMKIN
QBO54037.1_flavonol_synthase_Muscari_aucheri      FWPNNPPEYRKASEEYARRLQGVVDETLGSLSLWLG..LERHVLKEAVGGERLEYLLKIN
OsFLS_FLS_Oryza_sativa                            IWPSHPAGYREANEEYCKHMQRLARKLFEHLSTALG..LDGGAMWEAFGGDELVFLHKIN
AFS63900.1_FLS_Narcissus_tazetta                  FWPEKPEAYRTANKEYAKHLLRIVDEMLSSLSLGLG..LEEHTVKEAVGGDELEMLLKIN
AEC33115.1_FLS_Fagopyrum_esculentum               FWPKNPTSYKEANEEYCKCLRGVAEKIFRCMSLGLG..LEGPELMEAAGGDSLEYLLKIN
ABE28017.1_FLS_Nicotiana_tabacum                  YWPKNPPSYREANEEYAKRLREVAEKIFKSLSLGLG..LEAHEMMEAAGGEEIVYLLKIN
BAE75809.1_FLS_Vitis_vinifera                     FWPKTPPSYRDANEEYTKCLRGVADRLFSRLSLGLG..LDEDELKKSVGGDELTYLLKIN
AAX89401.1_FLS_Malus_domestica                    FWPKNPPSYREANEEYAKHLHNVVEKLFRLLSLGLG..LEGQELKKAAGGDNLEYLLKIN
CAA80264.1_FLS_Petunia_x_hybrida                  YWPKNPPSYREANEEYGKRMREVVDRIFKSLSLGLG..LEGHEMIEAAGGDEIVYLLKIN
ADZ28516.1_FLS_Camellia_nitidissima               FWPKNPPSYREANEVYAERLRAVADKLFRWLSLGLG..LEGHEVKEAVGGDDLIYLMKIN
LcFLS_FLS_Litchi_chinensis                        FWPKNPPSYKEVNEEYAKCMREVVDKLFQGLSIGLG..LEGHRLKEAAGGEEVVYMLKIN
ABB53382.1_FLS_Antirrhinum_majus                  FWPKNPPSYREANEEYTKKIRDVSDKLTNWLSLGLG..IKGHELKAAMGGDDTIFLMKIN
AAP57395.1_FLS_Petroselinum_crispum               FWPNNPPLYREANEEYAVALRGVVDKLFEALSLGIG..LEKHELKKASGGDDLIYMLKIN
BAC10995.1_flavonol_synthase_Nierembergia_sp      YWPKNPPSYREANEVYGKKLREVVDKIFKSLSLGLG..LEAHEMKEAAGGDDIVYLLKIN
                                              acc
```

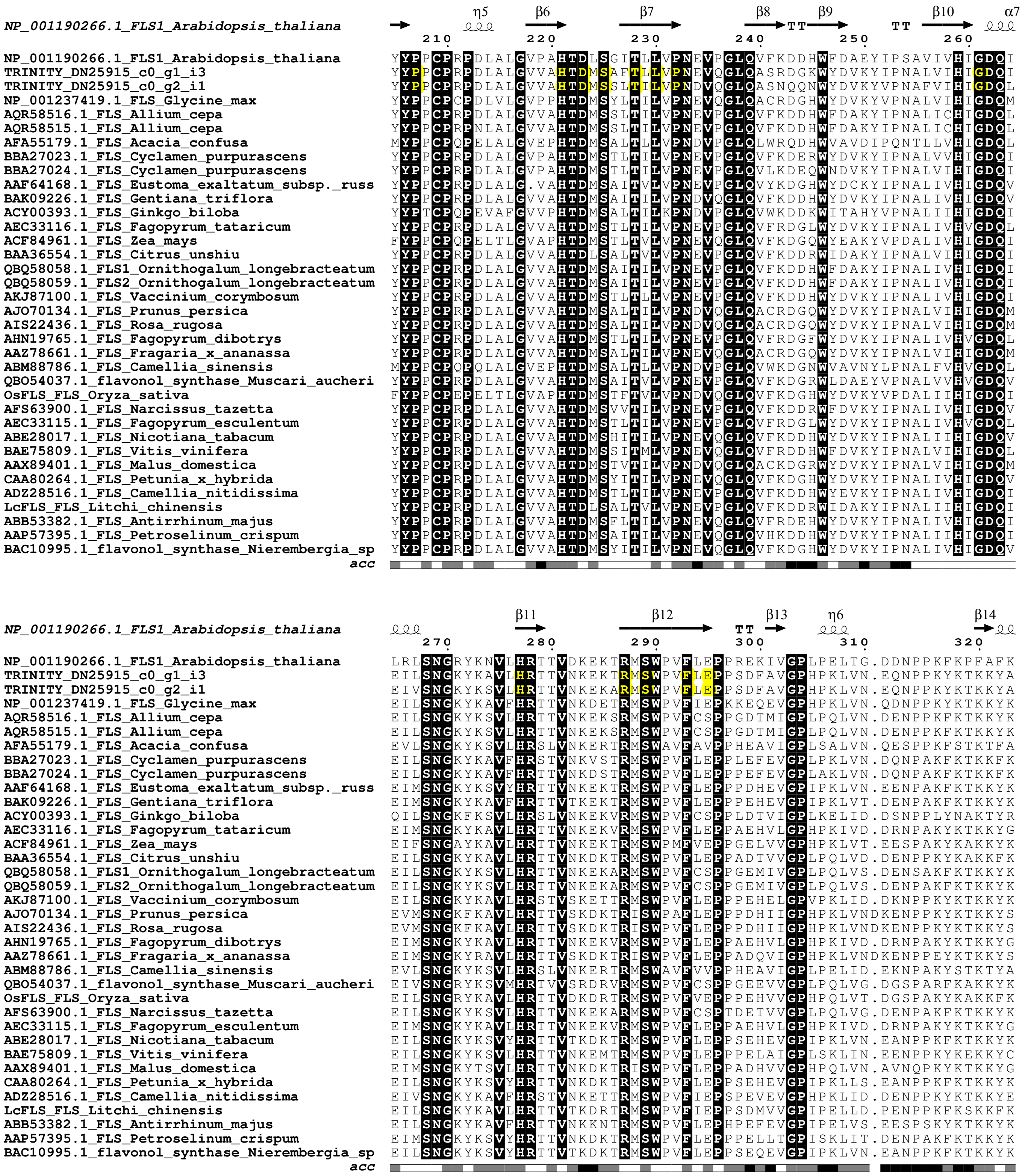
NP_001190266.1_FLS1_Arabidopsis_thaliana
η5 β6 β7 β8 TT β9 TT β10 α7
210 220 230 240 250 260
NP_001190266.1_FLS1_Arabidopsis_thaliana YYPPCPRPDLALGVPAHTDLSGITLLVPNEVPGLQVFKDDHWFDAEYIPSAVIVHIGDQI
TRINITY_DN25915_c0_g1_i3 YYPPCPRPDLALGVVAHTDMSAFTLLVPNEVQGLQASRDGKWYDVKYIPNALVIHIGDQI
TRINITY_DN25915_c0_g2_i1 YYPPCPRPDLALGVVAHTDMSSLTILVPNDVQGLQASNQQNWYDVKYVPNAFVIHIGDQL
NP_001237419.1_FLS_Glycine_max YYPPCPCPDLVLGVPPHTDMSYLTILVPNEVQGLQACRDGHWYDVKYVPNALVIHIGDQM
AQR58516.1_FLS_Allium_cepa YYPPCPRPDLALGVVAHTDMSSLTILVPNEVPGLQVFKDDHWFDAKYIPNALICHIGDQL
AQR58515.1_FLS_Allium_cepa YYPPCPRPNLALGVVAHTDMSSLTILVPNEVPGLQVFKDDHWFDAKYIPNALICHIGDQL
AFA55179.1_FLS_Acacia_confusa MYPPCPQPELALGVEPHTDMSALTLLVPNDVPGLQLWRQDHWVAVDIPQNTLLVHIGDQL
BBA27023.1_FLS_Cyclamen_purpurascens YYPPCPRPDLALGVPAHTDMSTLTILVPNEVPGLQVFKDERWYDVKYIPNALVVHIGDQI
BBA27024.1_FLS_Cyclamen_purpurascens YYPPCPRPDLALGVPAHTDMSTLTILVPNEVPGLQVLKDEQWNDVKYIPNALVIHIGDQI
AAF64168.1_FLS_Eustoma_exaltatum_subsp._russ YYPPCPRPDLALG.VAHTDMSAITVLVPNEVPGLQVFKDDHWYDCKYIPNALIVHIGDQV
BAK09226.1_FLS_Gentiana_triflora YYPPCPRPDLALGVVAHTDMSAITILVPNDVQGLQVFKDDHWYDVKYIPNALIVHIGDQV
ACY00393.1_FLS_Ginkgo_biloba YYPTCPQPEVAFGVVPHTDMSALTILKPNDVPGLQVWKDDKWITAHYVPNALIIHIGDQI
AEC33116.1_FLS_Fagopyrum_tataricum YYPPCPRPDLALGVVAHTDMSTLTILVPNEVPGLQVFRDGLWYDVKYIPNALIVHVGDQL
ACF84961.1_FLS_Zea_mays FYPPCPQPELTLGVAPHTDMSTLTVLVPNEVQGLQVFKDGQWYEAKYVPDALIVHIGDQI
BAA36554.1_FLS_Citrus_unshiu YYPPCPRPDLALGVVAHTDLSALTVLVPNEVPGLQVFKDDRWIDAKYIPNALVIHIGDQI
QBQ58058.1_FLS1_Ornithogalum_longebracteatum YYPPCPRPDLALGVVAHTDMSAITILVPNEVPGLQVFKDDHWFDAKYIPNALIVHIGDQI
QBQ58059.1_FLS2_Ornithogalum_longebracteatum YYPPCPRPDLALGVVAHTDMSAITILVPNEVPGLQVFKDDHWFDAKYIPNALIVHIGDQI
AKJ87100.1_FLS_Vaccinium_corymbosum YYPPCPRPDLALGVVAHTDMSTLTLLVPNEVPGLQVFKDDHWYDVKYIPNALIVHIGDQI
AJO70134.1_FLS_Prunus_persica YYPPCPRPDLALGVVAHTDMSALTILVPNDVQGLQACRDGQWYDVKYIPNALVIHIGDQM
AIS22436.1_FLS_Rosa_rugosa YYPPCPRPDLALGVVAHTDMSALTILVPNDVQGLQACRDGQWYDVKYIPNALVIHIGDQM
AHN19765.1_FLS_Fagopyrum_dibotrys YYPPCPRPDLALGVVAHTDMSTLTILVPNEVPGLQVFRDGFWYDVKYIPNALIVHVGDQL
AAZ78661.1_FLS_Fragaria_x_ananassa YYPPCPRPDLALGVVAHTDMSALTILVPNEVQGLQACRDGQWYDVKYIPNALVIHIGDQM
ABM88786.1_FLS_Camellia_sinensis MYPPCPQPQLALGVEPHTDMSALTLLVPNDVPGLQVWKDGNWVAVNYLPNALFVHVGDQL
QBO54037.1_flavonol_synthase_Muscari_aucheri YYPPCPRPDLALGVVAHTDMSAITVLVPNEVPGLQVFKDGRWLDAEYVPNALVVHVGDQV
OsFLS_FLS_Oryza_sativa FYPPCPEPELTLGVAPHTDMSTFTVLVPNDVQGLQVFKDGHWYDVKYVPDALIIHIGDQI
AFS63900.1_FLS_Narcissus_tazetta YYPPCPRPDLALGVVAHTDMSVVTILVPNEVPGLQVFKDDRWFDVKYIPNALIVHIGDQV
AEC33115.1_FLS_Fagopyrum_esculentum YYPPCPRPDLALGVVAHTDMSTLTILVPNEVPGLQVFRDGLWYDVKYIPNALIVHVGDQL
ABE28017.1_FLS_Nicotiana_tabacum YYPPCPRPDLALGVVAHTDMSHITILVPNEVQGLQVFKDDHWYDVKYIPNALIIHIGDQV
BAE75809.1_FLS_Vitis_vinifera YYPPCPRPDLALGVVAHTDMSSITMLVPNEVPGLQVFRDDHWFDVKYIPNALVIHIGDQL
AAX89401.1_FLS_Malus_domestica YYPPCPRPDLALGVVAHTDMSTVTILVPNDVQGLQACKDGRWYDVKYIPNALVIHIGDQM
CAA80264.1_FLS_Petunia_x_hybrida YYPPCPRPDLALGVVAHTDMSYITILVPNEVQGLQVFKDGHWYDVKYIPNALIVHIGDQV
ADZ28516.1_FLS_Camellia_nitidissima YYPPCPRPDLALGVVAHTDMSAITILVPNEVPGLQVFKDDHWYEVKYIPNALIVHIGDQI
LcFLS_FLS_Litchi_chinensis YYPPCPRPDLALGVVAHTDLSALTVLVPNEVPGLQVFKDDRWIDAKYIPNALIIHIGDQI
ABB53382.1_FLS_Antirrhinum_majus YYPPCPRPDLALGVVAHTDMSFLTILVPNEVQGLQVFRDEHWYDVKYIPNALIVHIGDQI
AAP57395.1_FLS_Petroselinum_crispum YYPPCPRPDLALGVVAHTDMSAITILVPNEVQGLQVHKDDHWYDVKYIPNALIIHIGDQI
BAC10995.1_flavonol_synthase_Nierembergia_sp YYPPCPRPDLALGVVAHTDMSYITILVPNEVQGLQVFKDGHWYDVKYIPNALIVHIGDQV
acc
NP_001190266.1_FLS1_Arabidopsis_thaliana
β11 β12 TT β13 η6 β14
270 280 290 300 310 320
NP_001190266.1_FLS1_Arabidopsis_thaliana LRLSNGRYKNVLHRTTVDKEKTRMSWPVFLEPPREKIVGPLPELTG.DDNPPKFKPFAFK
TRINITY_DN25915_c0_g1_i3 EILSNGKYKAVLHRTTVNKEKTRMSWPVFLEPPSDFAVGPHPKLVN.EQNPPKYKTKKYA
TRINITY_DN25915_c0_g2_i1 EIVSNGKYKSVLHRTTVNKEKARMSWPVFLEPPSDFAVGPHPKLVN.EQNPPKYKTKKYA
NP_001237419.1_FLS_Glycine_max EILSNGKYKAVFHRTTVNKDETRMSWPVFIEPKKEQEVGPHPKLVN.QDNPPKYKTKKYK
AQR58516.1_FLS_Allium_cepa EILSNGKYKSVLHRTTVNKEKSRMSWPVFCSPPGDTMIGPLPQLVN.DENPPKFKTKKYK
AQR58515.1_FLS_Allium_cepa EILSNGKYKSVLHRTTVNKEKSRMSWPVFCSPPGDTMIGPLPQLVN.DENPPKFKTKKYK
AFA55179.1_FLS_Acacia_confusa EVLSNGKYKSVLHRSLVNKERTRMSWAVFAVPPHEAVIGPLSALVN.QESPPKFSTKTFA
BBA27023.1_FLS_Cyclamen_purpurascens EILSNGKYKAVFHRSTVNKVSTRMSWPVFLEPPLEFEVGPLPKLVN.DQNPAKFKTKKFK
BBA27024.1_FLS_Cyclamen_purpurascens EILSNGKYKAVFHRTTVNKDSTRMSWPVFLEPPPEFEVGPLAKLVN.DQNPAKFKTKKFK
AAF64168.1_FLS_Eustoma_exaltatum_subsp._russ EIMSNGKYKSVYHRTTVNKEKTRMSWPVFLEPPPDHEVGPIPKLVN.EENPAKFKTKKYK
BAK09226.1_FLS_Gentiana_triflora EIMSNGKYKAVFHRTTVTKEKTRMSWPVFLEPPKEHEVGPIPKLVT.DENPAKFKTKKYK
ACY00393.1_FLS_Ginkgo_biloba QILSNGKFKSVLHRSLVNKEKVRMSWPVFCSPPLDTVIGPLKELID.DSNPPLYNAKTYR
AEC33116.1_FLS_Fagopyrum_tataricum EIMSNGKYKAVLHRTTVNKEKVRMSWPVFLEPPAEHVLGPHPKIVD.DRNPAKYKTKKYG
ACF84961.1_FLS_Zea_mays EIFSNGAYKAVLHRTTVNKEKTRMSWPMFVEPPGELVVGPHPKLVT.EESPAKYKAKKYK
BAA36554.1_FLS_Citrus_unshiu EILSNGKYKAVLHRTTVNKDKTRMSWPVFLEPPADTVVGPLPQLVD.DENPPKYKAKKFK
QBQ58058.1_FLS1_Ornithogalum_longebracteatum EILSNGKYKSVLHRTTVNKEKARMSWPVFCSPPGEMIVGPLPQLVS.DENSAKFKTKKYK
QBQ58059.1_FLS2_Ornithogalum_longebracteatum EILSNGKYKSVLHRTTVNKEKARMSWPVFCSPPGEMIVGPLPQLVS.DENPAKFKTKKYK
AKJ87100.1_FLS_Vaccinium_corymbosum EILSNGKYKSVLHRSTVSKETTRMSWPVFLEPPPEHELGPVPKLID.DKNPPKYKTKKYK
AJO70134.1_FLS_Prunus_persica EVMSNGKFKAVLHRTTVSKDKTRISWPAFLEPPPDHIIGPHPKLVNDKENPPKYKTKKYS
AIS22436.1_FLS_Rosa_rugosa EVMSNGKFKAVLHRTTVSKDKTRISWPVFLEPPPDHIIGPHPKLVNDKENPPKYKTKKYS
AHN19765.1_FLS_Fagopyrum_dibotrys EIMSNGKYKAVLHRTTVNKEKVRMSWPVFLEPPAEHVLGPHPKIVD.DRNPAKYKTKKYG
AAZ78661.1_FLS_Fragaria_x_ananassa EIMSNGKYRAVLHRTTVSKDKTRISWPVFLEPPADQVIGPHPKLVNDKENPPEYKTKKYS
ABM88786.1_FLS_Camellia_sinensis EVLSNGKYKSVLHRSLVNKERTRMSWAVFVVPPHEAVIGPLPELID.EKNPAKYSTKTYA
QBO54037.1_flavonol_synthase_Muscari_aucheri EIVSNGRYKSVMHRTVVSRDRVRMSWPVFCSPCGEEVVGPLPQLVG.DGSPARFKAKKYK
OsFLS_FLS_Oryza_sativa EILSNGRYKAVLHRTTVDKDRTRMSWPVFVEPPPEHVVGPHPQLVT.DGSPAKYKAKKFK
AFS63900.1_FLS_Narcissus_tazetta EILSNGKYKSVLHRTTVNKEKARMSWPVFCSPTDETVVGPLPQLVG.DENPAKFKTKKYK
AEC33115.1_FLS_Fagopyrum_esculentum EIMSNGKYKAVLHRTTVNKEKVRMSWPVFLEPPAEHVLGPHPKIVD.DRNPAKYKTKKYG
ABE28017.1_FLS_Nicotiana_tabacum EILSNGKYKSVYHRTTVTKDKTRMSWPVFLEPPSEHEVGPIPKLVN.EANPPKFKTKKYK
BAE75809.1_FLS_Vitis_vinifera EILSNGKYKSVLHRTTVNKEMTRMSWPVFLEPPPELAIGPLSKLIN.EENPPKYKEKKYC
AAX89401.1_FLS_Malus_domestica EIMSNGKYTSVLHRTTVNKDKTRISWPVFLEPPADHVVGPHPQLVN.AVNQPKYKTKKYG
CAA80264.1_FLS_Petunia_x_hybrida EILSNGKYKSVYHRTTVNKDKTRMSWPVFLEPPSEHEVGPIPKLLS.EANPPKFKTKKYK
ADZ28516.1_FLS_Camellia_nitidissima EIVSNGKYKAVFHRSTVNKETTRMSWPVFIEPPSPEVEVGPHPKLID.DENPPKYKTKKYE
LcFLS_FLS_Litchi_chinensis EILSNGKYKAVLHRTTVTKDRTRMSWPVFLEPPSDMVVGPIPELLD.PENPPKFKSKKFK
ABB53382.1_FLS_Antirrhinum_majus EILSNGKYKAVFHRTTVNKKNTRMSWPVFLEPHPEFEVGPIPELVS.EESPSKYKTKKYK
AAP57395.1_FLS_Petroselinum_crispum EIMSNGKYKSVYHRTTVNKDKTRMSWPVFLEPPPELLTGPISKLIT.DENPAKFKTKKYK
BAC10995.1_flavonol_synthase_Nierembergia_sp EILSNGKYKSVYHRTTVTKDKTRMSWPVFLEPPSEQEVGPIPKLVN.EANPPKFKTKKYK
acc

	α8
NP_001190266.1_FLS1_Arabidopsis_thaliana	TT
	330
NP_001190266.1_FLS1_Arabidopsis_thaliana	DYSYRKLNKLPLD
TRINITY_DN25915_c0_g1_i3	DYCYCKLNKIPQ.
TRINITY_DN25915_c0_g2_i1	DYCYCKLNKIPQ.
NP_001237419.1_FLS_Glycine_max	DYAYCKLNKIPQ.
AQR58516.1_FLS_Allium_cepa	DYAYCKINKLPQ.
AQR58515.1_FLS_Allium_cepa	DYAYCKINKLPQ.
AFA55179.1_FLS_Acacia_confusa	EFRHRKFNKLPQ.
BBA27023.1_FLS_Cyclamen_purpurascens	DYLYCKLNKLPQ.
BBA27024.1_FLS_Cyclamen_purpurascens	DYFYCKLNKLPQ.
AAF64168.1_FLS_Eustoma_exaltatum_subsp._russ	DYAYCKLNKLPQ.
BAK09226.1_FLS_Gentiana_triflora	DYAYCKLNKLPQ.
ACY00393.1_FLS_Ginkgo_biloba	EYKHRKINKLGQ.
AEC33116.1_FLS_Fagopyrum_tataricum	DYMYCKLNKLPQ.
ACF84961.1_FLS_Zea_mays	DYQHCKINKLPM.
BAA36554.1_FLS_Citrus_unshiu	DYSYCKLNKLPQ.
QBQ58058.1_FLS1_Ornithogalum_longebracteatum	DYSYCKINKLPQ.
QBQ58059.1_FLS2_Ornithogalum_longebracteatum	EYSYCKINKLPQ.
AKJ87100.1_FLS_Vaccinium_corymbosum	DYVYCKLNKLPQ.
AJO70134.1_FLS_Prunus_persica	EYVYNKLNKIPQ.
AIS22436.1_FLS_Rosa_rugosa	EYVYNKLNKIPQ.
AHN19765.1_FLS_Fagopyrum_dibotrys	DYMYCKLNKLPQ.
AAZ78661.1_FLS_Fragaria_x_ananassa	EYVYNKLNKMPQ.
ABM88786.1_FLS_Camellia_sinensis	EYRHRKFNKIPQ.
QBO54037.1_flavonol_synthase_Muscari_aucheri	DYSYCKINKLPQ.
OsFLS_FLS_Oryza_sativa	DYRHCKINKLPM.
AFS63900.1_FLS_Narcissus_tazetta	DYCYCKLNKLPQ.
AEC33115.1_FLS_Fagopyrum_esculentum	DYMYCKLNKLPQ.
ABE28017.1_FLS_Nicotiana_tabacum	DYVYCKLNKLPQ.
BAE75809.1_FLS_Vitis_vinifera	DYVYCKLNKIPQ.
AAX89401.1_FLS_Malus_domestica	DYVYCKINKLPQ.
CAA80264.1_FLS_Petunia_x_hybrida	DYVYCKLNKLPQ.
ADZ28516.1_FLS_Camellia_nitidissima	DYMYCKLNKLPQ.
LcFLS_FLS_Litchi_chinensis	DYSYCKLSKIP..
ABB53382.1_FLS_Antirrhinum_majus	DYVYCKLNKIPQ.
AAP57395.1_FLS_Petroselinum_crispum	DYVYCKLNKLPQ.
BAC10995.1_flavonol_synthase_Nierembergia_sp	DYVYCKLNKLPQ.
acc	

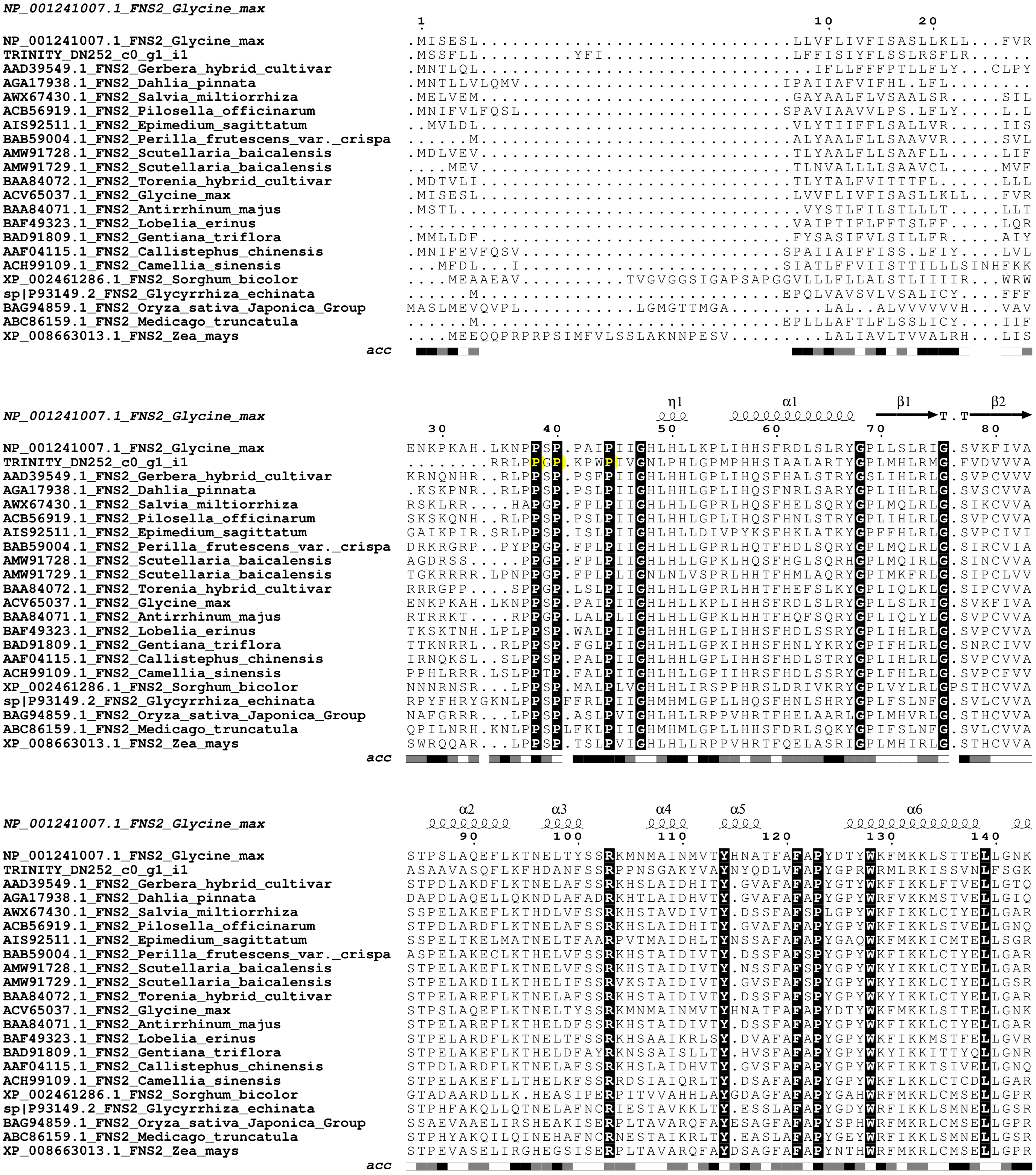
NP_001241007.1_FNS2_Glycine_max
1
10
20
NP_001241007.1_FNS2_Glycine_max .MISESL..........................LLVFLIVFISASLLKLL...FVR
TRINITY_DN252_c0_g1_i1 .MSSFLL........YFI...............LFFISIYFLSSLRSFLR......
AAD39549.1_FNS2_Gerbera_hybrid_cultivar .MNTLQL...........................IFLLFFFPTLLFLY...CLPY
AGA17938.1_FNS2_Dahlia_pinnata .MNTLLVLQMV.......................IPAIIAFVIFHL.LFL.......
AWX67430.1_FNS2_Salvia_miltiorrhiza .MELVEM...........................GAYAALFLVSAALSR....SIL
ACB56919.1_FNS2_Pilosella_officinarum .MNIFVLFQSL.......................SPAVIAAVVLPS.LFLY....L.L
AIS92511.1_FNS2_Epimedium_sagittatum ..MVLDL...........................VLYTIIFFLSALLVR.....IIS
BAB59004.1_FNS2_Perilla_frutescens_var._crispa ......M...........................ALYAALFLLSAAVVR.....SVL
AMW91728.1_FNS2_Scutellaria_baicalensis .MDLVEV...........................TLYAALFLLSAAFLL.....LIF
AMW91729.1_FNS2_Scutellaria_baicalensis ....MEV...........................TLNVALLLLSAAVCL.....MVF
BAA84072.1_FNS2_Torenia_hybrid_cultivar .MDTVLI...........................TLYTALFVITTTFL......LLL
ACV65037.1_FNS2_Glycine_max .MISESL...........................LVVFLIVFISASLLKLL...FVR
BAA84071.1_FNS2_Antirrhinum_majus .MSTL.............................VYSTLFILSTLLLT.....LLT
BAF49323.1_FNS2_Lobelia_erinus ..................................VAIPTIFLFFTSLFF.....LQR
BAD91809.1_FNS2_Gentiana_triflora .MMLLDF...........................FYSASIFVLSILLFR.....AIY
AAF04115.1_FNS2_Callistephus_chinensis .MNIFEVFQSV.......................SPAIIAIFFISS.LFIY....LVL
ACH99109.1_FNS2_Camellia_sinensis ...MFDL...I.......................SIATLFFVIISTTILLLSINHFKK
XP_002461286.1_FNS2_Sorghum_bicolor ....MEAAEAV.........TVGVGGSIGAPSAPGGVLLLFLLALSTLIIIIR...WRW
sp|P93149.2_FNS2_Glycyrrhiza_echinata ......M...........................EPQLVAVSVLVSALICY....FFF
BAG94859.1_FNS2_Oryza_sativa_Japonica_Group MASLMEVQVPL..........LGMGTTMGA........LAL..ALVVVVVVH...VAV
ABC86159.1_FNS2_Medicago_truncatula ......M...........................EPLLLAFTLFLSSLICY....IIF
XP_008663013.1_FNS2_Zea_mays ....MEEQQPRPRPSIMFVLSSLAKNNPESV.........LALIAVLTVVALRH...LIS
acc
NP_001241007.1_FNS2_Glycine_max
η1
α1
β1
T.T
β2
30
40
50
60
70
80
NP_001241007.1_FNS2_Glycine_max ENKPKAH.LKNPPSP.PAIPIIGHLHLLKPLIHHSFRDLSLRYGPLLSLRIG.SVKFIVA
TRINITY_DN252_c0_g1_i1 ........RRLPPGP.KPWPIVGNLPHLGPMPHHSIAALARTYGPLMHLRMG.FVDVVVA
AAD39549.1_FNS2_Gerbera_hybrid_cultivar KRNQNHR..RLPPSP.PSFPIIGHLHHLGPLIHQSFHALSTRYGSLIHLRLG.SVPCVVV
AGA17938.1_FNS2_Dahlia_pinnata .KSKPNR..RLPPSP.PSLPIIGHLHHLGPLIHQSFNRLSARYGPLIHLRLG.SVSCVVA
AWX67430.1_FNS2_Salvia_miltiorrhiza RSKLRR....HAPGP.FPLPIIGHLHLLGPRLHQSFHELSQRYGPLMQLRLG.SIKCVVA
ACB56919.1_FNS2_Pilosella_officinarum SKSKQNH..RLPPSP.PSLPIIGHLHHLGPLIHQSFHNLSTRYGPLIHLRLG.SVPCVVA
AIS92511.1_FNS2_Epimedium_sagittatum GAIKPIR.SRLPPSP.ISLPIIGHLHLLDIVPYKSFHKLATKYGPFFHLRLG.SVPCIVI
BAB59004.1_FNS2_Perilla_frutescens_var._crispa DRKRGRP..PYPPGP.FPLPIIGHLHLLGPRLHQTFHDLSQRYGPLMQLRLG.SIRCVIA
AMW91728.1_FNS2_Scutellaria_baicalensis AGDRSS.....PPGP.FPLPIIGHLHLLGPKLHQSFHGLSQRHGPLMQIRLG.SINCVVA
AMW91729.1_FNS2_Scutellaria_baicalensis TGKRRRR.LPNPPGP.FPLPLIGNLNLVSPRLHHTFHMLAQRYGPIMKFRLG.SIPCLVV
BAA84072.1_FNS2_Torenia_hybrid_cultivar RRRGPP....SPPGP.LSLPIIGHLHLLGPRLHHTFHEFSLKYGPLIQLKLG.SIPCVVA
ACV65037.1_FNS2_Glycine_max ENKPKAH.LKNPPSP.PAIPIIGHLHLLKPLIHHSFRDLSLRYGPLLSLRIG.SVKFIVA
BAA84071.1_FNS2_Antirrhinum_majus RTRRKT....RPPGP.LALPLIGHLHLLGPKLHHTFHQFSQRYGPLIQLYLG.SVPCVVA
BAF49323.1_FNS2_Lobelia_erinus TKSKTNH.LPLPPSP.WALPIIGHLHHLGPLIHHSFHDLSSRYGPLIHLRLG.SVPCVVA
BAD91809.1_FNS2_Gentiana_triflora TTKNRRL..RLPPSP.FGLPIIGHLHLLGPKIHHSFHNLYKRYGPIFHLRLG.SNRCIVV
AAF04115.1_FNS2_Callistephus_chinensis IRNQKSL..SLPPSP.PALPIIGHLHLLGPLIHHSFHDLSTRYGPLIHLRLG.SVPCVVA
ACH99109.1_FNS2_Camellia_sinensis PPHLRRR.LSLPPTP.FALPIIGHLHLLGPIIHRSFHDLSSRYGPLFHLRLG.SVPCFVV
XP_002461286.1_FNS2_Sorghum_bicolor NNNRNSR...LPPSP.MALPLVGHLHLIRSPPHRSLDRIVKRYGPLVYLRLGPSTHCVVA
sp|P93149.2_FNS2_Glycyrrhiza_echinata RPYFHRYGKNLPPSPFFRLPIIGHMHMLGPLLHQSFHNLSHRYGPLFSLNFG.SVLCVVA
BAG94859.1_FNS2_Oryza_sativa_Japonica_Group NAFGRRR...LPPSP.ASLPVIGHLHLLRPPVHRTFHELAARLGPLMHVRLG.STHCVVA
ABC86159.1_FNS2_Medicago_truncatula QPILNRH.KNLPPSPLFKLPIIGHMHMLGPLLHHSFDRLSQKYGPIFSLNFG.SVLCVVA
XP_008663013.1_FNS2_Zea_mays SWRQQAR...LPPSP.TSLPVIGHLHLLRPPVHRTFQELASRIGPLMHIRLG.STHCVVA
acc
NP_001241007.1_FNS2_Glycine_max
α2
α3
α4
α5
α6
90
100
110
120
130
140
NP_001241007.1_FNS2_Glycine_max STPSLAQEFLKTNELTYSSRKMNMAINMVTYHNATFAFAPYDTYWKFMKKLSTTELLGNK
TRINITY_DN252_c0_g1_i1 ASAAVASQFLKFHDANFSSRPPNSGAKYVAYNYQDLVFAPYGPRWRMLRKISSVNLFSGK
AAD39549.1_FNS2_Gerbera_hybrid_cultivar STPDLAKDFLKTNELAFSSRKHSLAIDHITY.GVAFAFAPYGTYWKFIKKLFTVELLGTQ
AGA17938.1_FNS2_Dahlia_pinnata DAPDLAQELLQKNDLAFADRKHTLAIDHVTY.GVAFAFAPYGPYWRFVKKMSTVELLGIQ
AWX67430.1_FNS2_Salvia_miltiorrhiza SSPELAKEFLKTHDLVFSSRKHSTAVDIVTY.DSSFAFSPLGPYWKFIKKLCTYELLGAR
ACB56919.1_FNS2_Pilosella_officinarum STPDLARDFLKTNELAFSSRKHSLAIDHITY.GVAFAFAPYGPYWKFIKKLSTVELLGNQ
AIS92511.1_FNS2_Epimedium_sagittatum SSPELTKELMATNELTFAARPVTMAIDHLTYNSSAFAFAPYGAQWKFMKKICMTELLSGR
BAB59004.1_FNS2_Perilla_frutescens_var._crispa ASPELAKECLKTHELVFSSRKHSTAIDIVTY.DSSFAFSPYGPYWKFIKKLCTYELLGAR
AMW91728.1_FNS2_Scutellaria_baicalensis STPELAKEFLKTNELVFSSRKHSTAIDIVTY.NSSFAFSPYGPYWKYIKKLCTYELLGAR
AMW91729.1_FNS2_Scutellaria_baicalensis STPELAKDILKTHELIFSSRVKSTAIDIVTY.GVSFAFSPYGPYWKYIKKLCTYELLGSR
BAA84072.1_FNS2_Torenia_hybrid_cultivar STPELAREFLKTNELAFSSRKHSTAIDIVTY.DSSFAFSPYGPYWKYIKKLCTYELLGAR
ACV65037.1_FNS2_Glycine_max STPSLAQEFLKTNELTYSSRKMNMAINMVTYHNATFAFAPYDTYWKFMKKLSTTELLGNK
BAA84071.1_FNS2_Antirrhinum_majus STPELAREFLKTHELDFSSRKHSTAIDIVTY.DSSFAFAPYGPYWKFIKKLCTYELLGAR
BAF49323.1_FNS2_Lobelia_erinus STPELARDFLKTNELTFSSRKHSAAIKRLSY.DVAFAFAPYGPYWKFIKKMSTFELLGVR
BAD91809.1_FNS2_Gentiana_triflora STPELAKEFLKTHELDFAYRKNSSAISLLTY.HVSFAFAPYGPYWKYIKKITTYQLLGNR
AAF04115.1_FNS2_Callistephus_chinensis STPDLARDFLKTNELAFSSRKHSLAIDHVTY.GVSFAFAPYGPYWKFIKKTSIVELLGNQ
ACH99109.1_FNS2_Camellia_sinensis STPELAKEFLLTHELKFSSRRDSIAIQRLTY.DSAFAFAPYGPYWKFLKKLCTCDLLGAR
XP_002461286.1_FNS2_Sorghum_bicolor GTADAARDLLK.HEASIPERPITVVAHHLAYGDAGFAFAPYGAHWRFMKRLCMSELLGPR
sp|P93149.2_FNS2_Glycyrrhiza_echinata STPHFAKQLLQTNELAFNCRIESTAVKKLTY.ESSLAFAPYGDYWRFIKKLSMNELLGSR
BAG94859.1_FNS2_Oryza_sativa_Japonica_Group SSAEVAAELIRSHEAKISERPLTAVARQFAYESAGFAFAPYSPHWRFMKRLCMSELLGPR
ABC86159.1_FNS2_Medicago_truncatula STPHYAKQILQINEHAFNCRNESTAIKRLTY.EASLAFAPYGEYWRFIKKLSMNELLGSR
XP_008663013.1_FNS2_Zea_mays STPEVASELIRGHEGSISERPLTAVARQFAYDSAGFAFAPYNTHWRFMKRLCMSELLGPR
acc

```
                                                  α7                          β3      α8
NP_001241007.1_FNS2_Glycine_max                   150       160       170       180       190
NP_001241007.1_FNS2_Glycine_max                   TLGHFLPIRTREVHDIIQFLFHKSKAQESVNLTEALLSLSNNVISQMMLSIK.....SSG
TRINITY_DN252_c0_g1_i1                            ALDDFRHIRQHEVSVLTRAL..ASAGSTPVNLGQLLNVCTANALGRVMIGRRVFGDGSGG
AAD39549.1_FNS2_Gerbera_hybrid_cultivar           NLSHFLPIRTHEIRELLRTLMVKSRAKERVNLTEELLKLTNNVISQMMMSIR.....CSG
AGA17938.1_FNS2_Dahlia_pinnata                    NLGHFLPIRTQEIHGLLLTLTEKSKQNESVNMTNELLKLSNNIICQMMMGIR.....CSG
AWX67430.1_FNS2_Salvia_miltiorrhiza               NLLHFQPVRTLEVNTFLQILLRKGKSGESFNLTEELVKLTSNVISHMMLSIR.....CSG
ACB56919.1_FNS2_Pilosella_officinarum             NLGHFLPIRTQEIHELLHTLMEKSKRKESVNLTEELLKLTNNVICQMMMSIR.....CSG
AIS92511.1_FNS2_Epimedium_sagittatum              ALNQLYPIRKEELLLLVQVLVHKSSEGETVNLSEALVNISNNIISRMMWGAT.....YSG
BAB59004.1_FNS2_Perilla_frutescens_var._crispa    NLAHFQPIRTLEVKSFLQILMRKGESGESFNVTEELVKLTSNVISHMMLSIR.....CSE
AMW91728.1_FNS2_Scutellaria_baicalensis           NLHHFQPIRTFEVHTFLRLLMEKSESGESFNVTEELIKLTSNVMSNMMLGTR.....CSA
AMW91729.1_FNS2_Scutellaria_baicalensis           MLNHFEPLRALEVREFLKDVMAMGKAGKSFNVTEELMKLTSNVMSNMMLSIR.....AAE
BAA84072.1_FNS2_Torenia_hybrid_cultivar           NLGHFQPIRNLEVRSFLQLLMHKSFKGESVNVTDELVRLTSNVISHMMLSIR.....CSE
ACV65037.1_FNS2_Glycine_max                       TLGHFLPIRTREVHDIIQFLFHKSKAQESVNLTEALLSLSNNVISQMMLSIK.....SSG
BAA84071.1_FNS2_Antirrhinum_majus                 NLSHFQPIRALEVNSFLRILYEKTEQKQSVNVTEELVKLTSNVISNMMLGIR.....CSG
BAF49323.1_FNS2_Lobelia_erinus                    NLSHFLPIRTREIHGLIHLLMKKSKGKERVNVTEELVRLTNNVISQMMMSIR.....CSG
BAD91809.1_FNS2_Gentiana_triflora                 NLTHFEPIRRLETNRVLYDLMVSSKHGKSVNLTEEMIKLTSNIISQMMLSIR.....CSD
AAF04115.1_FNS2_Callistephus_chinensis            NLSNFLPIRTQEVHELLQTLMVKSKKNESVNLSEELLKLTNNVICQMMMSIR.....CSG
ACH99109.1_FNS2_Camellia_sinensis                 SINHFLPTRTRELHCFVRLLIDKAVACEPVNITKELSTLANNIISQMMIGVR.....CSG
XP_002461286.1_FNS2_Sorghum_bicolor               TVDQLRPVREAELAAVLGAARQASASGERIDVSRYLISMSNNAIMRMVASAL.....PG.
sp|P93149.2_FNS2_Glycyrrhiza_echinata             SINNFQHLRAQETHQLLRLLSNRARAFEAVNITEELLKLTNNVISIMMV...........
BAG94859.1_FNS2_Oryza_sativa_Japonica_Group       TVEQLRPVRRAGLVSLLRHVL.SQPEAEAVDLTRELIRMSNTSIIRMAASTV.....PS.
ABC86159.1_FNS2_Medicago_truncatula               SISSFQHLRLQETHNLLKLFADKAKNYEAVNVTQELLKLSNNVISKMML...........
XP_008663013.1_FNS2_Zea_mays                      TVEQLRPVRRAGTVSLLADLLASSARGETVDLTRHLIRLSNTSIIRMVASTV.....PG.
                                            acc

                                                      α9             α10   α11   η2          α12
NP_001241007.1_FNS2_Glycine_max                   200       210       220       230       240       250
NP_001241007.1_FNS2_Glycine_max                   TDSQAEQARTLVREVTQIFGEFNVSDFLGFCKNLDLQGFRKRALDIHKRYDALLEKIISD
TRINITY_DN252_c0_g1_i1                            GDRKADEFKTMVVELMVLAGVFNIGDFVPALEWLDLQGVAAKMKKLHKRFDAFLSEIVED
AAD39549.1_FNS2_Gerbera_hybrid_cultivar           TNSEADEAKNLVREVTKIFGQFNVSDFIWFCKNIDLQGFKKRYEGTHRRYDALLERIIMG
AGA17938.1_FNS2_Dahlia_pinnata                    NKTEAEEAKNLVREVTTIFGEFNVSDFIWFCKKLDLQGFKKRYEDIRTRYDALLERIIFA
AWX67430.1_FNS2_Salvia_miltiorrhiza               TEGEAEAARTVVREVTQIFGEFDVADIICFCKNFDFQGIRKRSQDIRRRYDALLENIITL
ACB56919.1_FNS2_Pilosella_officinarum             TNSEADEAKNLVREVTTIFGQFNVSDFIWFCKNLDLQGFKKRYEDTHRRYDALLEKIISE
AIS92511.1_FNS2_Epimedium_sagittatum              KDDQAKEARVLIREVTQIFGTFNLSDFIWLLRKFDIQGLLKKSKDARKRYNVMIERIIKE
BAB59004.1_FNS2_Perilla_frutescens_var._crispa    TESEAEAARTVIREVTQIFGEFDVSDIIWLCKNFDFQGIRKRSEDIQRRYDALLEKIITD
AMW91728.1_FNS2_Scutellaria_baicalensis           TDGEAEAARTVIREVTEIFGEFDAADIIWFCKNFDLQGIRKRSEDIQRRYDALLEKIITD
AMW91729.1_FNS2_Scutellaria_baicalensis           SEEQAEVARTLIREVSQLFGEFDFGDMLWFCKSFDFQGIKKRSKDIKVRYDALLEKILTD
BAA84072.1_FNS2_Torenia_hybrid_cultivar           DEGDAEAARTVIREVTQIFGEFDVTDIIWFCKKFDLQGIKKRSEDIQRRYDALLEKIISD
ACV65037.1_FNS2_Glycine_max                       TDSQAEQARTLVREVTQIFGEFNVSDFLGFCKNLDLQGFRKRALDIHKRYDALLEKIISD
BAA84071.1_FNS2_Antirrhinum_majus                 TEGEAEVARTVIREVTQIFGEFDVSEIVWFCKNLDLQGIRKRSEDIRRRYDALLEKIISD
BAF49323.1_FNS2_Lobelia_erinus                    TDGEADEAKNLVREVTQIFGQFNLSDFVWFCRNVDLQGIKKRYEDTHRRYDALLEKIISE
BAD91809.1_FNS2_Gentiana_triflora                 TESGATNVRNVIRDVTELFGEFDVSDIIWFCKNTDLQGIKKRANGIHERYDALLEKIISD
AAF04115.1_FNS2_Callistephus_chinensis            TNNEADEAKNLVREVTKIFGEFNISDFICLFKNIDLQGFKKRYVDTHTRYNALLEKMIFE
ACH99109.1_FNS2_Camellia_sinensis                 TTGEAEEATTLAREVTKIFGEFNVSDFMWVIRNFDLQGFRKRVEDIYTRYDALLERIITN
XP_002461286.1_FNS2_Sorghum_bicolor               ..HMTEAARDCAKHVAELVGAFNIEDYVGICRGWDLQGITRRTREVRDKFDALMEIMITA
sp|P93149.2_FNS2_Glycyrrhiza_echinata             ..GEAEEARDVVRDVTEIFGEFNVSDFIWLFKKMDLQGFGKRIEDLFQRFDTLVERIISK
BAG94859.1_FNS2_Oryza_sativa_Japonica_Group       ..SVTEEAQELVKVVAELVGAFNADDYIALCRGWDLQGLGRRAADVHKRFDALLEEMIRH
ABC86159.1_FNS2_Medicago_truncatula               ..GEAEEARDVVRDVTEIFGEFNVSDFIWLFKKLDLQGFGKRIEDLFMRFDTLVERIISK
XP_008663013.1_FNS2_Zea_mays                      ..SVTDEAQEVVKDVAELVGAFNVDDYISLVRGWDLQGLRRRAAGVHRRFDALLEDILRH
                                            acc

                                                                                   α13
NP_001241007.1_FNS2_Glycine_max                                       TT
                                                  260       270       280       290       300
NP_001241007.1_FNS2_Glycine_max                   REELR...RKSKVDGCED..GDDEK.........VKDFLDILLDVAEQKECE.VQLTRNH
TRINITY_DN252_c0_g1_i1                            HKMNL...KGS.........DGSEKH.........TDMLSTLIALEEDADGEGGKLTDTE
AAD39549.1_FNS2_Gerbera_hybrid_cultivar           REENR...RR.....GKI..KDGEG..........KDFLDMLLDVLEDGKAE.IKITRDH
AGA17938.1_FNS2_Dahlia_pinnata                    REEMR...KE....GKGM..EDGKG..........KDFLDMLLDVLEDDKAE.IKITRNH
AWX67430.1_FNS2_Salvia_miltiorrhiza               RENHR...RS.......A..AAAVP..........RDFLDMFLDIMESGKAE.VTFTRDH
ACB56919.1_FNS2_Pilosella_officinarum             REEKR...RK....GGKR..DDVKG..........TDFLDMLLDVLEDGKAE.IKITRDH
AIS92511.1_FNS2_Epimedium_sagittatum              REEFR.TNKKL.........GSTEGKAD....GGVKDFLDILLDISEDDNAE.MKLNRDH
BAB59004.1_FNS2_Perilla_frutescens_var._crispa    REKQR...RTH...GGGG..GGGEA..........KDFLDMFLDIMESGKAE.VKFTREH
AMW91728.1_FNS2_Scutellaria_baicalensis           REKLR...RSH...R......GGEA..........KDFLDIFLDIMDSGNSE.VKFSREH
AMW91729.1_FNS2_Scutellaria_baicalensis           RENVR...RQN.........GVVEP..........KDMLDMFLDIMEGGKTD.VEFTREH
BAA84072.1_FNS2_Torenia_hybrid_cultivar           RERSR...RQNRDKHGGG..NNEEA..........KDFLDMLLDVMESGDTE.VKFTREH
ACV65037.1_FNS2_Glycine_max                       REELR...RKSKVDGCED..GDDEK.........VKDFLDILLDVAEQKECE.VQLTRNH
BAA84071.1_FNS2_Antirrhinum_majus                 RERLR...LRG...GGGG..GGGEV..........KDFLDMLLDVMESEKSE.VEFTREH
BAF49323.1_FNS2_Lobelia_erinus                    REEKR...RGK.MGGGKIGEGEGEG..........KDFLDMLLDVLEDKKAE.VKITRDH
BAD91809.1_FNS2_Gentiana_triflora                 RERTRIVEKKNSGAGGGS..GDGER..........NDFLDILMDAMEDDTSE.VKLSRNH
AAF04115.1_FNS2_Callistephus_chinensis            REEKR...KQ....KKS..EDGKG..........KDFLDILLDVLEDENAE.IKITRDH
ACH99109.1_FNS2_Camellia_sinensis                 REEVR...EKN.VQERKL..GVGEGH.......HVKDFLDLLLDVLEEDHSE.INFSRDN
XP_002461286.1_FNS2_Sorghum_bicolor               KEEKR...RSQGDDGAE..............TPTKDLLDILMDAAADENAE.VRLTREN
sp|P93149.2_FNS2_Glycyrrhiza_echinata             REQTR...KDRRRNGKKGEQGSGDG.........IRDFLDILLDCTEDENSE.IKIQRVH
BAG94859.1_FNS2_Oryza_sativa_Japonica_Group       KEEAR...MRKKTDTD...............VGSKDLLDILLDKAEDGAAE.VKLTRDN
ABC86159.1_FNS2_Medicago_truncatula               REELR...KNKGRKENKGEQGA..E.........FRDFLDILLDCAEDQNSE.IKVQRVH
XP_008663013.1_FNS2_Zea_mays                      KEEAR...AARRLDQDDD..GRGSSKQDKKQATHSKDLLDILMDKADDPAAE.IKLTREN
                                            acc
```

```
                                              α14                                   α15                       η3
NP_001241007.1_FNS2_Glycine_max                  310        320        330        340        350        360
NP_001241007.1_FNS2_Glycine_max                 VKSLILDYFTAATDTTAISVEWTIAELFNNPKVLKKAQEEVDRVTGNTQLVCEADIPNLP
TRINITY_DN252_c0_g1_i1                          IKALLLNMFTAGTDTSSSTVEWAIAELIRHPKILSKVKQELDSVVGRDRLVTELDLAQLT
AAD39549.1_FNS2_Gerbera_hybrid_cultivar         IKALILDFLTAGTDTTAIAIEWALVELINNPNALEKARQEIDQVIGDERLVQESDTPNLP
AGA17938.1_FNS2_Dahlia_pinnata                  IKALILDFVTAGTGTTAVIVEWTLVELIKNPMVMEKAKQELDEVVGNTRLVEESDAPKLP
AWX67430.1_FNS2_Salvia_miltiorrhiza             LKALILDFFTAGTDTTAIVSEWAISELINNPSVLKKAQDEIAKVVGSERVLHESDGPKLP
ACB56919.1_FNS2_Pilosella_officinarum           IKALILDFFTAATDTTAIAVEWTMVELINNPKVLEKAKKEVDNVIGNSRLVQESDAPNLP
AIS92511.1_FNS2_Epimedium_sagittatum            IKAFILDIFTAGTDTSAALTEWAMSELINNPKILDKARKEIDSVV.TGRMVEESDLPNLP
BAB59004.1_FNS2_Perilla_frutescens_var._crispa  LKALILDFFTAGTDTTAIVCEWAIAEVINNPNVLKKAQEEIANIVGFDRILQESDAPNLP
AMW91728.1_FNS2_Scutellaria_baicalensis         LKALILDFFTAGTDTTAISTEWAIAELMNNPKVLKKAQEEIQKVVGSCRLMDESDAPNLP
AMW91729.1_FNS2_Scutellaria_baicalensis         LKAVILDFLTAGTDTTAITVEWVLAELMNSPKAMKKAQDEMDRVVGRERMMAESDAPNLP
BAA84072.1_FNS2_Torenia_hybrid_cultivar         LKALILDFFTAGTDTTAIATEWAIAELINNPNVLKKAQEEISRIIGTKRIVQESDAPDLP
ACV65037.1_FNS2_Glycine_max                     VKSLILDYFTAATDTTAISVEWTIAELFNNPKVLKKAQEEVDRVTGNTQLVCEADIPNLP
BAA84071.1_FNS2_Antirrhinum_majus               LKALILDFFTAGTDTTAITTEWAIAELISNPNVLKKAQEEMDKVIGSQRLLQESDAPNLP
BAF49323.1_FNS2_Lobelia_erinus                  IKALILDFFTAATDTTAISIEWALAELMNNPKVLAKAQEEIDRVIGNKRLAQETDYPNLP
BAD91809.1_FNS2_Gentiana_triflora               IKAIILDFLTAATDTTAISLEWALSELINNPRVLKKAQEEINNVVGNQRLVKELDTPNFP
AAF04115.1_FNS2_Callistephus_chinensis          IKALILDFFTAATDTTAISIEWTLVELTNNPKVLENARKEIAEVVGDERLVQESDIPNLP
ACH99109.1_FNS2_Camellia_sinensis               IKGLILDFFTAGTDTSSIAIEWALAELINNPRVLQKAQEEIDNVVGKHRLVSESDGPNLP
XP_002461286.1_FNS2_Sorghum_bicolor             IKAFVIDIFTAGSDTTATSVEWMLAYLINHPACMDKLRAELDAVVGTSRLVGENDVPRLP
sp|P93149.2_FNS2_Glycyrrhiza_echinata           IKALIMDFFTAGTDTTAISTEWALVELVKKPSVLQKVREEIDNVVGKDRLVEESDCPNLP
BAG94859.1_FNS2_Oryza_sativa_Japonica_Group     IKAFIIDVVTAGSDTSAAMVEWMVAELMNHPEALRKVREEIEAVVGRDRIAGEGDLPRLP
ABC86159.1_FNS2_Medicago_truncatula             IKALIMDFFTAGTDTTSISTEWALVELMNNPSLLQKAREEIDNVVGKNRLVDESDGPNLP
XP_008663013.1_FNS2_Zea_mays                    IKAFIIDVVTAGSDTSAAMVEWMLAELMNHPETLRKVAEEIDAVVGGDRIASEADLPQLP
                                           acc

                                                   α16           β4                       β5    α17
NP_001241007.1_FNS2_Glycine_max                                 -.              T.T                         TT
                                                   370        380        390        400        410        420
NP_001241007.1_FNS2_Glycine_max                 YIHAIIKETMRLHPPIPM.IMRKGIEDCVVN.GNMIPKGSIVCVNIWAMGRDPNIWKNPL
TRINITY_DN252_c0_g1_i1                          YFQAVVKETFRLHPSTPLSLPRLAAESCEIN.GYHIPKGSTLLVNVWAIARDPDVWSDPL
AAD39549.1_FNS2_Gerbera_hybrid_cultivar         YIQAIIKEALRLHPPIPM.LIRKSTENVIVQ.GYDIPAGTLLFVNIWSIGRNPQCWETPL
AGA17938.1_FNS2_Dahlia_pinnata                  YIQAIIKEAFRLHPPIPM.IIRKSNENVSVKSGYEIPAGSILFVNNWSIGRNPKYWESPL
AWX67430.1_FNS2_Salvia_miltiorrhiza             YLQAVIKETFRLHPPIPM.LARKSISDCVVD.GYTIPSNTLLFVNLWSMGRDPKIWDCPE
ACB56919.1_FNS2_Pilosella_officinarum           YIQAIIKETLRLHPPIPM.LIRKSIEKVTVQ.GYEIPAGTMLFVNIWSIGRNAQYWESPL
AIS92511.1_FNS2_Epimedium_sagittatum            YIHAIYKEALRLHPPVPM.LPREASQECKVA.GYDIPAKTNLFVNLWSIQRDPNNWKDPL
BAB59004.1_FNS2_Perilla_frutescens_var._crispa  YLQALIKETFRLHPPIPM.LARKSISDCVID.GYMIPANTLLFVNLWSMGRNPKIWDYPT
AMW91728.1_FNS2_Scutellaria_baicalensis         YLEAIIKETFRLHPPIPM.LARKSVSDCVID.GYNIPASTLLFVNIWSIGRNPECWDSPF
AMW91729.1_FNS2_Scutellaria_baicalensis         YFLAIIKETFRLHPPIPL.IIRRSIEDCVID.GYHIPADTLAFINVWSMGRNEKYWDSPL
BAA84072.1_FNS2_Torenia_hybrid_cultivar         YLQAIIKETFRLHPPIPM.LSRKSTSDCTVN.GYKIQAKSLLFVNIWSIGRNPNYWESPM
ACV65037.1_FNS2_Glycine_max                     YIHAIIKETMRLHPPIPM.IMRKGIEDCVVN.GNMIPKGSIVCVNIWAMGRDPNIWKNPL
BAA84071.1_FNS2_Antirrhinum_majus               YLNAIIKETFRLHPPIPM.LTRKSISDVVVN.GYTIPAKTLLFVNLWSMGRNPNYWENPM
BAF49323.1_FNS2_Lobelia_erinus                  YIQAIIKENFRLHPPIPM.LIRKSIDDCTVQ.GYSIPSHTLLFVNIWSIGRNPNYWESPL
BAD91809.1_FNS2_Gentiana_triflora               YIKAIIKETFRLHPPIPM.VIRKSANDIQVA.GYDVPKNTMLFVNIWSIGRNPSYWEKAS
AAF04115.1_FNS2_Callistephus_chinensis          YIQAIIKETLRMHPPIPM.VIRKSIDNVTVQ.GYDIRAGTMLFVNIWSIGRNPLYWESPL
ACH99109.1_FNS2_Camellia_sinensis               YIQAIIREALRLHPPVPL.ITRKSIEDCMIQ.GYNIPANSMLFVNVWSLARNPKYWDSPL
XP_002461286.1_FNS2_Sorghum_bicolor             YLQAVFKETLRLQPPAVF.SQRETIEPVHVR.GYVIPRKTTVFFNIFSIGRDPAWWEDPL
sp|P93149.2_FNS2_Glycyrrhiza_echinata           YLQAILKETFRLHPPVPM.VTRRCVAECTVE.NYVIPEDSLLFVNVWSIGRNPKFWDNPL
BAG94859.1_FNS2_Oryza_sativa_Japonica_Group     YLQAAYKETLRLRPAAPI.AHRQSTEEIQIR.GFRVPAQTAVFINVWAIGRDPAYWEEPL
ABC86159.1_FNS2_Medicago_truncatula             YIQAIIKETFRLHPPVPM.VTRRCVTQCKIE.NYVIPENSLIFVNNWAMGRNSAYWDKPL
XP_008663013.1_FNS2_Zea_mays                    YLMAAYKETLRLHPAAPI.AHRQSSEEMVVR.GFTVPPQTAVFINVWAIGRDPAYWEEPL
                                           acc

                                                  η4    η5                                        α18
NP_001241007.1_FNS2_Glycine_max                                                 TT   TTT
                                                      430                 440        450        460        470
NP_001241007.1_FNS2_Glycine_max                 EFKPERFLEGEG....SAIDTKGHHFELLPFGSGRRGCPGMPLAMRELPTIIGALIQCFE
TRINITY_DN252_c0_g1_i1                          EFKPERFLPGGE...KANVDVRGNDFELIPFGAGRRICAGMSLGLRMVQLLIATLVHGFD
AAD39549.1_FNS2_Gerbera_hybrid_cultivar         EFKPHRFLDGGDL..KSSLDIKGHNFQLLPFGTGRRGCPGVNLAMRELSVVIANLIQCFD
AGA17938.1_FNS2_Dahlia_pinnata                  EFKPNRFLKEGVL..KPSLDIRGQNFQILPFGTGRRSCPGINMAMRQLPVVVAILIQCFE
AWX67430.1_FNS2_Salvia_miltiorrhiza             VFRPERFLEKAN....ASIDIKGQHFELLPFGTGRRGCPGMLLGIQEVVIIIGSMIQCFD
ACB56919.1_FNS2_Pilosella_officinarum           EFEPDRFFEGDTL..KSSLDIKGQSFQLLPFGTGRRGCPGINLAMRELPVVIAGLIQCFE
AIS92511.1_FNS2_Epimedium_sagittatum            EFKPERFMQLHDDQVGSFKEVRGQQYDLLPFGTGRRGCPGASLVLQQAPAMLAALIQCFD
BAB59004.1_FNS2_Perilla_frutescens_var._crispa  AFQPERFLEKEK....AAIDVKGQHFELLPFGTGRRGCPGMLLAIQEVVIIIGTMIQCFD
AMW91728.1_FNS2_Scutellaria_baicalensis         SFRPERFFEKDN....ASIDIKGQHFQLLPFGTGRRGCPGMLLAIQELLLIIGTMIQCFD
AMW91729.1_FNS2_Scutellaria_baicalensis         SFRPERFLEGDN....AAIDIKGMHFELLPFGSGRRGCPGMLSAIQEVLIIAGTVIQCFD
BAA84072.1_FNS2_Torenia_hybrid_cultivar         EFRPERFLEKGR....ESIDVKGQHFELLPFGTGRRGCPGMLLAIQEVVSIIGTMVQCFD
ACV65037.1_FNS2_Glycine_max                     EFKPERFLEGEG....SAIDTKGHHFELLPFGSGRRGCPGMPLAMRELPTIIGALIQCFE
BAA84071.1_FNS2_Antirrhinum_majus               EFRPERFLEKGT....GSIDVKGQHFELLPFGTGRRGCPGMLLGMQELFSIIGAMVQCFD
BAF49323.1_FNS2_Lobelia_erinus                  EFKPERFLEKNSP..VSSIDIKGHDFQLLPFGTGRRGCPGVALAMRELPTALAALIQCFE
BAD91809.1_FNS2_Gentiana_triflora               EFSPERFLADTDGGGLSHMDINGQYFELMPFGTGRRGCPGMPLAMQELPTVLSLMIQCFD
AAF04115.1_FNS2_Callistephus_chinensis          EFKPHRFLDGHA....RNLDVKGQCFQLLPFGTGRRGCPGISLAMRELPVVIAGLIQCFE
ACH99109.1_FNS2_Camellia_sinensis               DFLPERFLRPEKGGPVGPTDVKGQHFQLLPFGTGRRGCPGTSLAMQELPAMLAAMIQCFE
XP_002461286.1_FNS2_Sorghum_bicolor             EFRPERFMPGGA...GAAVDPKGQHMHFLPFGSGRRACPGMGLAMQAVPAFLAALVQCFH
sp|P93149.2_FNS2_Glycyrrhiza_echinata           EFRPERFLKLEGDS.SGVVDVRGSHFQLLPFGSGRRMCPGVSLAMQEVPALLGAIIQCFD
BAG94859.1_FNS2_Oryza_sativa_Japonica_Group     EFRPERFLAGGG...GEGVEPRGQHFQFMPFGSGRRGCPGMGLALQSVPAVVAALLQCFD
ABC86159.1_FNS2_Medicago_truncatula             EFNPERFLKNSTNS.NGVIDVRGQNFQILPFGSGRRMCPGVTLAMQEVPALLGAIIQCFD
XP_008663013.1_FNS2_Zea_mays                    AFRPERFMPGGA...AESLEPRGQHFQYMPFGSGRRGCPGMGLALQSVPAVLAALVQCFH
                                           acc
```

```
                                               β6                                       β7
NP_001241007.1_FNS2_Glycine_max                ───>       TT.T                          ────>
                                               480        490        500       510       520
NP_001241007.1_FNS2_Glycine_max                WKMLGSQGEI.LDHGRSLISMDERPGLTAPRANDLIGIPVARLNPTPFRQM........
TRINITY_DN252_c0_g1_i1                         WELADGL....M...PDKLNMDEAFGLTLQRAVPLMVHPRPRL.PLHAYGASN......
AAD39549.1_FNS2_Gerbera_hybrid_cultivar        WDVVGER..........LLNTDERAGLTAPRAVDFVCVPLERGNTLKILGS...N....
AGA17938.1_FNS2_Dahlia_pinnata                 WTVNDKQ..........VLNMDEKGGLTTPRATDLVCFPLLRKNSPHSMFT...SV...
AWX67430.1_FNS2_Salvia_miltiorrhiza            WKLPQGT.........RAIDMAERSGLTAPRAYDLICRVVPRIDPTLVFGALPDSV...
ACB56919.1_FNS2_Pilosella_officinarum          WDVNNKE..........ALITDERAGLTAPRAVDFVCVPSMRENCPKVF..........
AIS92511.1_FNS2_Epimedium_sagittatum           WEVAGHDDGIKL....ATVDMIERPGLTVPRANALLLVPTTRFNPFTDTVVHP......
BAB59004.1_FNS2_Perilla_frutescens_var._crispa WKLPDGS.........GHVDMAERPGLTAPRETDLFCRVVPRVDPLVVSTQ........
AMW91728.1_FNS2_Scutellaria_baicalensis        WELPEGS.........GPVDMTERAGLTAPRAEDLICRVSCRVDPKIVF..........
AMW91729.1_FNS2_Scutellaria_baicalensis        WEQADGS.........GRVDMSERPGLTTPREIDLVCRVVPRVDERVISGH........
BAA84072.1_FNS2_Torenia_hybrid_cultivar        WKLADGSG........NNVDMTERSGLTAPRAFDLVCRLYPRVDPATISGA........
ACV65037.1_FNS2_Glycine_max                    WKMLGSQGEI.LDHGRSLISMDERPGLTAPRANDLIGIPVARLNPTPFRQM........
BAA84071.1_FNS2_Antirrhinum_majus              WKLPDGV.........KSVDMTERPGLTAPRANDLVCQLVPRIDPVVVSGP........
BAF49323.1_FNS2_Lobelia_erinus                 WKAEGGE..........ALDMSERAGLTAPRAHDLVCVPVARINSPDIFDC........
BAD91809.1_FNS2_Gentiana_triflora              YIPLDFKGE....KAERVMDMSERPGLTAPRANELMCLLKPRIDLPNLLGNVKGE....
AAF04115.1_FNS2_Callistephus_chinensis         WNANDKE..........VLSMDERAGLTAPRAVDLEFVPLMRQNCPNIFVS...A....
ACH99109.1_FNS2_Camellia_sinensis              WKVVNQSGDV.M.NGDGALDMTEQPGMTAPRAHDLVCMPIPRIDQLYALLDP.......
XP_002461286.1_FNS2_Sorghum_bicolor            WAVPIPQGQ....STAPPLDMEEEAGLVTARKHHLVLIPTPRLNPLPVPVPVPATGKAT
sp|P93149.2_FNS2_Glycyrrhiza_echinata          FHVVGPKGEI.LKGDDIVINVDERPGLTAPRAHNLVCVPVDRTSGGGPLKIIEC.....
BAG94859.1_FNS2_Oryza_sativa_Japonica_Group    WQCMD..........NKLIDMEEADGLVCARKHRLLLHAHPRLHPFPPLL.........
ABC86159.1_FNS2_Medicago_truncatula            FNFVGPKGEI.LKGGDIVIDVNERPGLTAPRVHDLVCVPVERFACGGPLQSLGC.....
XP_008663013.1_FNS2_Zea_mays                   WATVDGDG.....GVNK.IDMSESDGLVCARKKPLLLRPTPRLTPFPAVV.........
                                          acc
```

NP_001268144.1_DFR_Vitis_vinifera

β1 → α1 β2

1 · 10 · 20 · 30

**NP_001268144.1_DFR_Vitis_vinifera**	.........MG..................SQSETVCVTGASGFIGSWLVMRLLERGYTVR
**TRINITY_DN27402_c0_g1_i3**	.........MET..................QTKTLCVTGASGFIGSWLVMRLLECGYMVR
**NP_199094.1_DFR_Arabidopsis_thaliana**	.........MV..................SQKETVCVTGASGFIGSWLVMRLLERGYFVR
**BAA85261.1_DFR_Arabidopsis_thaliana**	.........MV..................SQKETVCVTGASGFIGSWLVMRLLERGYFVR
**AEI59122.1_DFR_Medicago_sativa**	.........MG..................SVSETVCVTGASGFIGSWLVMRLIEHGYTVR
**BAD67185.1_DFR_Spinacia_oleracea**	.........MVV..................QGEIVCVTGAAGFIGSWLVKRLLEHGYIVR
**BAB40789.1_DFR_Lilium_hybrid**	.........MEN..................VKGPVVVTGASGYVGSWLVMKLLQYGYTVR
**CAA79154.1_DFR_Solanum_lycopersicum**	.........MASEAHAVV......DAHSPPKTTTVWVTGGAGFIGSWLVMRLLERGYNVH
**AHZ30596.1_DFR_Prunus_domestica**	.................................SVCVXGASGFXGSWLVMRLLERGYTVR
**AAD26204.1_DFR_Malus_domestica**	.........MG..................SESESVCVTGASGFIGSWLVMRLLEHGYTVR
**AAO39819.1_DFR_Pyrus_communis**	.........MG..................SESESVCVTGASGFIGSWLVMRLLEHGYTVR
**AAD56578.1_DFR_Daucus_carota**	.........MV..................KELHTVCVTGASGFIGSWLVLRLLQRGYTVR
**AKN56970.1_DFR_Gerbera_hybrid_cultivar**	.........MEE.................DSPATVCVTGAAGFIGSWLVMRLLERGYVVH
**AOF39984.11_DFR_Brassica_rapa**	.........MV..................AHKETVCVTGASGFIGSWLVMRLLERGYFVR
**P51106.1_DFR_Hordeum_vulgare**	.........MDG..................NKGPVVVTGASGFVGSWLVMKLLQAGYTVR
**BAE19953.1_DFR_Lotus_japonicus**	.........MS..................SESETVCVTGAAGFIGSWLVMRLIERGYTVR
**AII26023.1_DFR_Pisum_sativum**	.........MG..................SVSETVCVTGASGFIGSWLVMRLIEHGYTVR
**NP_001274988.1_DFR_Solanum_tuberosum**	.........MASEVHSVV......DAHSPPKTPTVCVTGAAGFIGSWLVMRLLERGYNVH
**NP_001152467.2_DFR_Zea_mays**	.MEG...GAGAS..................EKGKVLVTGASGFVGSWLVMKLLQAGYTVR
**AIZ74402.1_DFR_Anthurium_andraeanum**	.........MM...................HKGTVCVTGAAGFVGSWLIMRLLEQGYSVK
**AAB62873.1_DFR_Bromheadia_finlaysoniana**	.........MEN.................EKKGPVVVTGASGYVGSWLVMKLLQKGYDVR
**CAA91922.1_DFR_Callistephus_chinensis**	.........MKE.................DSPPTVCVTGAAGFIGSWLVMRLLERGYIVR
**AIM58715.1_DFR_Cymbidium_hybrid_cultivar**	.........MET.................ERKGPVVVTGASGYVGSWLVMKLLQKGYEVR
**ACZ48698.1_DFR_Fagopyrum_esculentum**	.........MV..................AEGEIVCVTGASGFVGSWLVMRLLEHGYVVR
**ACZ48697.1_DFR_Fagopyrum_tataricum**	.........MV..................AEGEIVCVTGASGFVGSWLVMRLLEHGYVVR
**BAO53730.1_DFR2_Glycine_max**	.........MGS................SSASESVCVTGASGFIGSWLVMRLIERGYTVR
**AAP13055.1_DFR_Gypsophila_elegans**	.........MVSSNNTTETLDGKHDTKPV.QGETVCVTGASGFIGSWLIMRLLERGYTVR
**XP_013466134.1_DFR_Medicago_truncatula**	.........MG..................SMAETVCVTGASGFIGSWLVMRLMERGYMVR
**BAF96936.1_DFR_Nicotiana_tabacum**	.........MASEAHAAV......HA.PPPVAPTVCVTGAAGFIGSWLVMRLLERGYNVH
**BAA36183.1_DFR_Oryza_sativa_Japonica_Group**	........MGEA..................VKGPVVVTGASGFVGSWLVMKLLQAGYTVR
**BAA12723.1_DFR_Rosa_hybrid_cultivar**	.........MA..................SESESVCVTGASGFIGSWLVMRLLDRGYTVR
**AHL46438.1_DFR_Fragaria_vesca**	.M......GLG..................AESGSVCVTGASGFVGSWLVMRLLERGYTVR
**AHL46443.1_DFR_Fragaria_x_ananassa**	.M......GLG..................AESGSVCVTGASGFVGSWLVMRLLEHGYTVR
**AHL46445.1_DFR_Fragaria_vesca**	.........MG..................SESESVCVHGASGFVGSWLVMRLLERGYTVR
**AHM27144.1_DFR_Angelonia_angustifolia**	.........METTATQPPP....PSS.AAAVPATVCVTGAAGFIGSWLVMKLLQRGYNVR
**AAT84073.1_DFR_Camellia_sinensis**	.........MKDSVA.........SA.TASAPGTVCVTGAAGFIGSWLVMRLLERGYIVR
**AAS00611.1_DFR_Citrus_sinensis**	.........MG..................SIAETVCVTGASGFIGSWLIMRLLERGYAVR
**AAX16491.1_DFR_Crataegus_monogyna**	.........MG..................SESESVCVTGASGFIGSWLVMRLLEHGYTVR
**BAF49325.1_DFR_Delphinium_belladonna**	.........MTV....................ETVCVTGAAGFIGSWLVMRLLERGYLVR
**CAA78930.1_DFR_Gerbera_hybrid_cultivar**	.........MEE.................DSPATVCVTGAAGFIGSWLVMRLLERGYVVH
**AAO39816.1_DFR_Malus_domestica**	.........MG..................SESESVCVTGASGFIGSWLVMRLLEHGYTVR
**AAO39817.1_DFR_Malus_domestica**	.........MG..................SESESVCVTGASGFIGSWLVMRLLEHGYTVR
**AAR27015.1_DFR2_Medicago_truncatula**	.........MG..................SVSETVCVTGASGFIGSWLVMRLMERGYTVR
**AEF14420.1_DFR_Onobrychis_viciifolia**	.........MG..................STSETVCVTGAAGFVGSWLVMRLIERGYTVR
**AAF60298.1_DFR_Petunia_x_hybrida**	...........MPLHLRC......SA.......TVCVTGAAGFIGSWLVMRLLERGYNVH
**AAQ77347.1_DFR_Triticum_aestivum**	.........MDG..................NKGPVVVTGASGFVGSWLVMKLLQVGYTVR
**AAL89715.1_DFR_Vaccinium_macrocarpon**	.........MKD.VN.........SG.LGT...TVCVTGAAGFIGSWLIMRLLERGYVVR
**AAX12420.1_DFR_Vaccinium_macrocarpon**	.........MKD.VN.........SG.LGT...TVCVTGAAGFIGSWLIMRLLERGYVVR
**CAA33543.1_DFR_Antirrhinum_majus**	.........MSPTSLNTSS.....ET.APPSSTTVCVTGAAGFIGSWLVMRLLERGYTVR
**AAB62873.1_DFR_Bromheadia_finlaysoniana**	.........MEN.................EKKGPVVVTGASGYVGSWLVMKLLQKGYDVR
**BAA84940.1_DFR_Camellia_sinensis**	.........MKDSVA.........SA.TASAPGTVCVTGAAGFIGSWLVMRLLERGYIVR
**AAC17843.1_DFR_Cymbidium_hybrid_cultivar**	.........MET.................ERKGPVVVTGASGYVGSWLVMKLLQKGYEVR
**CAA91924.1_DFR_Dianthus_caryophyllus**	.........MVSS.TINETLDGKHDINKVGQGETVCVTGASGFIGSWLIMRLLERGYTVR
**CAA70345.1_DFR_Forsythia_x_intermedia**	.........MET............DA.LPLPATTVCVTGASGFIGSWLVMRLLERGYFVR
**AAC25960.1_DFR_Fragaria_x_ananassa**	.M......GLG..................AESGSVCVTGASGFVGSWLVMRLLEHGYTVR
**BAA12736.1_DFR_Gentiana_triflora**	.........ME.............GG.ILSNATTVCVTGASGYIGSWLAMRLLERGYTVR
**AAD54273.1_DFR1_Glycine_max**	.........MG..................SASESVCVTGASGFIGSWLVMRLIERGYTVR
**BAA59332.1_DFR_Ipomoea_nil**	.........MVGGNHTPA...........SPAPTVCVTGAAGFVGSWLVMKLLQRGYIVH
**BAA36405.1_DFR_Ipomoea_purpurea**	.........MVGGNHTPA...........SPAPTVCVTGAAGFVGSWLVMKLLQRGYIVH
**AAD49343.1_DFR_Lilium_hybrid_division_VII**	.........MEN..................AKGPVVVTGASGYVGSWLVMKLLQYGYTIR
**AAF23884.2_DFR3_Lotus_corniculatus**	.........MG..................SVPETVCVTGAAGFIGSWLVMRLMERGYMVR
**BAE19948.1_DFR_Lotus_japonicus**	.........MG..................SAAKTVCVTGSTGFIGSWLVMRLMERGYMVR
**BAE19949.1_DFR_Lotus_japonicus**	.........MG..................SVPETVCVTGAAGFIGSWLVMRLMERGYMVR
**BAE19950.1_DFR_Lotus_japonicus**	.........MG..................SAAKTVCVTGSTGFIGSWLVMRLMEGGYTVR
**BAE19951.1_DFR_Lotus_japonicus**	.........MG..................SAAKTVCVTGSTGFIGSWLVMRLMERGYMVR
**BAE19953.1_DFR_Lotus_japonicus**	.........MS..................SESETVCVTGAAGFIGSWLVMRLIERGYTVR
**BAA19658.1_DFR_Perilla_frutescens**	.........MSLETVAAP...........PPATTVCVTGASGFIGSWIIMRLLERGYVVR
**BAB20075.1_DFR_Torenia_hybrid_cultivar**	.........MSMEVVVPK...........AQPITVCVTGASGFIGSWLVMKLLNRGYTVH
**AAL35830.1_DFR_Triticum_monococcum**	.........MDG..................SKGPVVVTGASGFVGSWLVMKLLQAGYTVR
**QFQ61498.1_DFR_Dryopteris_erythrosora**	.........MDK.................PLHSTVLVTGCTGHIGSWLVLRLLEKGYSVR
**QFQ61499.1_DFR_Dryopteris_erythrosora**	.MAPNAVAVMAL.................P..ELVCVTGASGHLGTWLVKRLLERGYRVR
**BAA74700.1_DFR_Ipomoea_purpurea**	.........MVDGNHPL.............PAPKVCVTGAAGFIGSWLVKTLLQRGYHVH
**BAF93896.1_DFR_Iris_x_hollandica**	..............................MMSPVVVTGASGYVGSWLVMKLLRDGYAVR
**XP_008797532.1_DFR_Phoenix_dactylifera**	.........ME...................TKGPVVVTGASGFIGTWLVMRLLEEGYIVR
**AEQ92209.1_DFR_Ipomoea_batatas**	.........MVDGNHP................KVVCVTGAAGFIGSWLVMTLLQRGYHVH
**BAH98155.1_DFR_Tulipa_gesneriana**	.........MKV..................VKGPVVATGASGYVGSWLVMKLLENGYTVR
**APG32494.1_DFR2_Freesia_hybrid_cultivar**	...............................MGTVVVTGASGYVGSWLLMKLLQNGYAVR
**AAO63026.1_DFR_Allium_cepa**	MMK.....EIGA..................AGGAVVVTGAGGYVGSWLVMKLLHYGYTVR
**AFP58815.1_DFR_Hyacinthus_orientalis**	.........MEM..................EKGPVVATGAGGYIGSWLVMKLLRAGYTVR
acc	

```
                                                                                   α2
NP_001268144.1_DFR_Vitis_vinifera          →                                         40         50

NP_001268144.1_DFR_Vitis_vinifera          ATV.RDP....................................TNVKKVKHLLDL.PKA.
TRINITY_DN27402_c0_g1_i3                   ATV.RDP....................................DNAKKVQHLLDL.PNA.
NP_199094.1_DFR_Arabidopsis_thaliana       ATV.RDP....................................GNLKKVQHLLDL.PNA.
BAA85261.1_DFR_Arabidopsis_thaliana        ATV.RDP....................................GNLKKVQHLLDL.PNA.
AEI59122.1_DFR_Medicago_sativa             ATV.RDP....................................DNIKKVKHLLEL.PGA.
BAD67185.1_DFR_Spinacia_oleracea           ATV.RDP....................................GNVKKVQHLLDL.PNA.
BAB40789.1_DFR_Lilium_hybrid               ATV.RDP....................................RDLRKTKPLLDL.PGA.
CAA79154.1_DFR_Solanum_lycopersicum        ATV.RDP....................................ENQKKVKHLLEL.PKA.
AHZ30596.1_DFR_Prunus_domestica            ATV.RDP....................................TNQKKVKHLLEL.PKA.
AAD26204.1_DFR_Malus_domestica             ATV.RDP....................................TNQKKVKHLLDL.PKA.
AAO39819.1_DFR_Pyrus_communis              ATV.RDP....................................TNQKKVKHLLDL.PKA.
AAD56578.1_DFR_Daucus_carota               ATV.RDP....................................GNPQKVKHLLQL.PKA.
AKN56970.1_DFR_Gerbera_hybrid_cultivar     ATV.RDP....................................GDLKKVKHLLEL.PKA.
AOF39984.11_DFR_Brassica_rapa              ATV.RDP....................................GNLKKVQHLLDL.PNA.
P51106.1_DFR_Hordeum_vulgare               ATV.RDP....................................ANVEKTKPLLEL.PGA.
BAE19953.1_DFR_Lotus_japonicus             ATI.RDP....................................ANMKKVKHLLEL.PDA.
AII26023.1_DFR_Pisum_sativum               ATV.RDP....................................DNVKKVKHLLEL.PDA.
NP_001274988.1_DFR_Solanum_tuberosum       ATV.RDP....................................ENQKKVKHLLEL.PKA.
NP_001152467.2_DFR_Zea_mays                ATV.RDP....................................ANVGKTKPLMDL.PGA.
AIZ74402.1_DFR_Anthurium_andraeanum        ATV.RDP....................................SNMKKVKHLLDL.PGA.
AAB62873.1_DFR_Bromheadia_finlaysoniana    ATI.RDP....................................TNLEKVKPLLDL.PRS.
CAA91922.1_DFR_Callistephus_chinensis      ATV.RNP....................................GDMKKVKHLLEL.PKA.
AIM58715.1_DFR_Cymbidium_hybrid_cultivar   AAV.RDS....................................TNFEKVKPLLDL.PGS.
ACZ48698.1_DFR_Fagopyrum_esculentum        ATV.RDP....................................TNMKKVKHLLDL.PKS.
ACZ48697.1_DFR_Fagopyrum_tataricum         ATV.RDP....................................SNMKKVKHLLDL.PKS.
BAO53730.1_DFR2_Glycine_max                ATV.HDP....................................ANMKKVKHLVEL.PGA.
AAP13055.1_DFR_Gypsophila_elegans          GTV.RDP....................................DNTKKVQHLLDL.PQA.
XP_013466134.1_DFR_Medicago_truncatula     ATV.RDP....................................ENLKKVSHLLEL.PGA.
BAF96936.1_DFR_Nicotiana_tabacum           ATV.RDP....................................ENKKKVKHLFEL.PKA.
BAA36183.1_DFR_Oryza_sativa_Japonica_Group ATV.RDP....................................SNVGKTKPLLEL.AGS.
BAA12723.1_DFR_Rosa_hybrid_cultivar        ATV.RDP....................................ANKKKVNHLLDL.PKA.
AHL46438.1_DFR_Fragaria_vesca              ATV.RDP....................................ANLKKVRHLLEL.PQA.
AHL46443.1_DFR_Fragaria_x_ananassa         ATV.RDP....................................ANLKKVRHLLEL.PQA.
AHL46445.1_DFR_Fragaria_vesca              ATV.RDP....................................TNAKKVKHLLDL.PKA.
AHM27144.1_DFR_Angelonia_angustifolia      ATV.RDP....................................GNLKKIKHLTQL.PKA.
AAT84073.1_DFR_Camellia_sinensis           ATV.RDP....................................ANLKKVKHLLDL.PKA.
AAS00611.1_DFR_Citrus_sinensis             ATV.RDP....................................DNKKKVKHLLEL.PKA.
AAX16491.1_DFR_Crataegus_monogyna          ATV.RDP....................................TNQKKVKHLLDL.PKA.
BAF49325.1_DFR_Delphinium_belladonna       ATV.RNP....................................DNLKKLRHLLEL.PNA.
CAA78930.1_DFR_Gerbera_hybrid_cultivar     ATV.RDP....................................GDLKKVKHLLEL.PKA.
AAO39816.1_DFR_Malus_domestica             ATV.RDP....................................TNQKKVKHLLDL.PKA.
AAO39817.1_DFR_Malus_domestica             ATV.RDP....................................TNQKKVKHLLDL.PKA.
AAR27015.1_DFR2_Medicago_truncatula        ATV.RDP....................................DNMKKVKHLLEL.PGA.
AEF14420.1_DFR_Onobrychis_viciifolia       ATV.RDP....................................ANMKKVKHLLDL.PDA.
AAF60298.1_DFR_Petunia_x_hybrida           ATV.RDP....................................ENKKKVKHLLEL.PKA.
AAQ77347.1_DFR_Triticum_aestivum           ATV.RDPGERSLRLRLPHFLSPTHTRLTRYLTAFVICFCVFVSANVEKNKPLLEL.PGA.
AAL89715.1_DFR_Vaccinium_macrocarpon       ATV.RDP....................................GNLKKVKHLLEL.PKA.
AAX12420.1_DFR_Vaccinium_macrocarpon       ATV.RDP....................................GNLKKVKHLLEL.PKA.
CAA33543.1_DFR_Antirrhinum_majus           ATV.RDP....................................GNMKKVKHLIEL.PKA.
AAB62873.1_DFR_Bromheadia_finlaysoniana    ATI.RDP....................................TNLEKVKPLLDL.PRS.
BAA84940.1_DFR_Camellia_sinensis           ATV.RDP....................................ANLKKVKHLLDL.PKA.
AAC17843.1_DFR_Cymbidium_hybrid_cultivar   AAV.RDS....................................TNFEKVKPLLDL.PGS.
CAA91924.1_DFR_Dianthus_caryophyllus       ATV.RDP....................................DNTKKVQHLLDL.PNA.
CAA70345.1_DFR_Forsythia_x_intermedia      ATA.RDP....................................ENKQKVKHLLEL.PRA.
AAC25960.1_DFR_Fragaria_x_ananassa         ATV.RDP....................................ANLKKVRHLLEL.PQA.
BAA12736.1_DFR_Gentiana_triflora           ATV.RDP....................................GNLKKVQHLLEL.PKA.
AAD54273.1_DFR1_Glycine_max                ATV.RDP....................................VNMKKVKHLVEL.PGA.
BAA59332.1_DFR_Ipomoea_nil                 ATV.RDP....................................GNAQKVKHLLEL.PKG.
BAA36405.1_DFR_Ipomoea_purpurea            ATV.RDP....................................GNTQKVKHLLEL.PKA.
AAD49343.1_DFR_Lilium_hybrid_division_VII  ATV.RDP....................................RDLRKTKPLLDI.PGA.
AAF23884.2_DFR3_Lotus_corniculatus         ATV.RDP....................................ANMKKVKHLLEL.PEA.
BAE19948.1_DFR_Lotus_japonicus             ATVQRDP....................................DNMKKVKHLLEL.PGA.
BAE19949.1_DFR_Lotus_japonicus             ATV.RDP....................................ANMKKVKHLLEL.PEA.
BAE19950.1_DFR_Lotus_japonicus             ATVQRDP....................................DNMKKVKHLLEL.PGA.
BAE19951.1_DFR_Lotus_japonicus             ATVQRDP....................................ENMKKVKHLLEL.PGA.
BAE19953.1_DFR_Lotus_japonicus             ATI.RDP....................................ANMKKVKHLLEL.PDA.
BAA19658.1_DFR_Perilla_frutescens          ATV.RDP....................................GDSKKVKHLIEL.PGA.
BAB20075.1_DFR_Torenia_hybrid_cultivar     ATV.RDP....................................ENMKKVKHLLEL.PRAD
AAL35830.1_DFR_Triticum_monococcum         ATV.RDP....................................ANVEKNKPLLEL.PGA.
QFQ61498.1_DFR_Dryopteris_erythrosora      AAV.LDP....................................EDKQDVQPLLGLAPSL.
QFQ61499.1_DFR_Dryopteris_erythrosora      ATV.RNP....................................GDPTKTAILRNL.PGA.
BAA74700.1_DFR_Ipomoea_purpurea            ATV.RDP....................................GNTKKVKHLVEL.PKA.
BAF93896.1_DFR_Iris_x_hollandica           ATV.RDP....................................TNVEKTKPLLDL.PGA.
XP_008797532.1_DFR_Phoenix_dactylifera     ATV.RDP....................................TNLKKTQPLLDL.PGA.
AEQ92209.1_DFR_Ipomoea_batatas             ATV.RDP....................................GNTKKVKHLLEL.PKA.
BAH98155.1_DFR_Tulipa_gesneriana           ATV.RDP....................................KDQGKTKPLLDL.RGA.
APG32494.1_DFR2_Freesia_hybrid_cultivar    ATV.RDP....................................TNLRKTKPLLDL.SGA.
AAO63026.1_DFR_Allium_cepa                 ATL.RDS....................................SDEAKTKPLLEL.PGA.
AFP58815.1_DFR_Hyacinthus_orientalis       ATV.RDP....................................ANTKKLKPLLDI.PGA.
                                     acc
```

```
                                            α3    β3               α4        β4                         α5
NP_001268144.1_DFR_Vitis_vinifera                          TT
                                                 60           70          80          90
NP_001268144.1_DFR_Vitis_vinifera           ETHLTLWKADLAD.EGSFDEAIKGCTGVFHVATPMDFESKDPE.................
TRINITY_DN27402_c0_g1_i3                    KTHMSLWKADLSV.EGSFDEPIQGCNGVFHVATPMDFESKDPE.................
NP_199094.1_DFR_Arabidopsis_thaliana        KTLLTLWKADLSE.EGSYDDAINGCDGVFHVATPMDFESKDPE.................
BAA85261.1_DFR_Arabidopsis_thaliana         KTQLTLWKADLSE.EGSYDDAINGCDGVFHVATPMDFESKDPE.................
AEI59122.1_DFR_Medicago_sativa              NSKLSLWKADLGE.EGSFDEAIKGCTGVFHVATPMDFESKDPE.................
BAD67185.1_DFR_Spinacia_oleracea            NTHLTLWKADLNE.QGSFDEAISGCAGVFHVATPMDFDSNDPE.................
BAB40789.1_DFR_Lilium_hybrid                DERLTIWKADLSE.DGSFDEAINGCTGVFHVATPMDFDSEDPE.................
CAA79154.1_DFR_Solanum_lycopersicum         DTNLTLWKADLAV.EGSFDEAIQGCQGVFHVATPMDFESKDPE.................
AHZ30596.1_DFR_Prunus_domestica             ETHLTLWKADLAD.EGSFDEAIQGCTGVFHVATPMDFESKDPE.................
AAD26204.1_DFR_Malus_domestica              ETHLTLWKADLAD.EGSFDEAIQGCSGVFHVATPMDFESKDPE.................
AAO39819.1_DFR_Pyrus_communis               ETHLTLWKADLAD.EGSFDEAIQGCSGVFHVATPMDFESKDPE.................
AAD56578.1_DFR_Daucus_carota                ETNLILCRADLNE.EGSFDDAVKGCHAVFHMATPMDIESQDPE.................
AKN56970.1_DFR_Gerbera_hybrid_cultivar      QTNLKLWKADLTQ.EGSFDEAVQGCHGVFHLATPMDFESKDPE.................
AOF39984.11_DFR_Brassica_rapa               KTQLTLWKADLSD.EGSYDDAINGCDGVFHIATPMDFESKDPE.................
P51106.1_DFR_Hordeum_vulgare                KERLSIWKADLSE.DGSFNEAIAGCTGVFHVATPMDFDSQDPE.................
BAE19953.1_DFR_Lotus_japonicus              KTKLSLWKADLAE.EGSFDEAIRGCTGVFHVATPMDFESKDPE.................
AII26023.1_DFR_Pisum_sativum                KSKLSLWKADLAE.EGSFDEAIKGCVGVFHVATPMDFESKDPE.................
NP_001274988.1_DFR_Solanum_tuberosum        DTNLTLWKADLAV.EGSFDEAIQGCQGVFHVATPMDFESKDPE.................
NP_001152467.2_DFR_Zea_mays                 TERLSIWKADLAE.EGSFHDAIRGCTGVFHVATPMDFLSKDPE.................
AIZ74402.1_DFR_Anthurium_andraeanum         ANRLTLWKADLVD.EDSFDEPIQGCTGVFHVATPMDFESKDPE.................
AAB62873.1_DFR_Bromheadia_finlaysoniana     NELLSIWKADLNDIEGSFDEVIRGCVGVFHVATPMNFQSKDPE.................
CAA91922.1_DFR_Callistephus_chinensis       ETNLTLWKADLTQ.EGSFDEAIEGCHGVFHVATPMDFESKDPE.................
AIM58715.1_DFR_Cymbidium_hybrid_cultivar    NELLSIWKADLNDIDETFDEVTRGSVGLFHVATPMNFQSEDPE.................
ACZ48698.1_DFR_Fagopyrum_esculentum         KTNLSLWKADLSE.EGSFDEAIQGCAGVFHVATPMDFESKDPE.................
ACZ48697.1_DFR_Fagopyrum_tataricum          KTNLSLWKADLSE.EGSFDEAIQGCAGVFHVATPMDFESKDPE.................
BAO53730.1_DFR2_Glycine_max                 KTKLSLWKADLAQ.EGSFDEAIKGCTGVFHVATPMDFDSKDPE.................
AAP13055.1_DFR_Gypsophila_elegans           KTNLTLWKADLNE.EGSFDKAVDGCSGVFHIATPMDFESKDPE.................
XP_013466134.1_DFR_Medicago_truncatula      KGKLSLWKADLGE.EGSFDEAIKGCTGVFHVATPMDFESKDPE.................
BAF96936.1_DFR_Nicotiana_tabacum            DTNLTLWKADLSV.EGSFDEAIQGCQGVFHVATPMDFESEDPE.................
BAA36183.1_DFR_Oryza_sativa_Japonica_Group  KERLTLWKADLGE.EGSFDAAIRGCTGVFHVATPMDFESEDPE.................
BAA12723.1_DFR_Rosa_hybrid_cultivar         ATHLTLWKADLAE.EGSFDEAIKGCTGVFHVATPMDFESKDPE.................
AHL46438.1_DFR_Fragaria_vesca               ATLLTLWKADLDI.EGSFDEAIKGCTGVFHVATPMDFESEDPE.................
AHL46443.1_DFR_Fragaria_x_ananassa          ATRLTLWKADLDV.EGSFDEAIKGCTGVFHVATPMDFESEDPE.................
AHL46445.1_DFR_Fragaria_vesca               ATHLTLWKADLAD.EGSFDEAIKGCTGVFHVATPMDFESKDPE.................
AHM27144.1_DFR_Angelonia_angustifolia       DTNLTLWKADLME.EASFDEAIEGCQGVFHLATPMEFDSKDPE.................
AAT84073.1_DFR_Camellia_sinensis            DTNLTLWKADLNE.EGSFDEAIEGCSGVFHVATPMDFKSKDPE.................
AAS00611.1_DFR_Citrus_sinensis              STHLTLWKADLAE.EGNFDEAIRGCTGVFHLATPMDFESKDPE.................
AAX16491.1_DFR_Crataegus_monogyna           ETHLTLWKADLAD.EGSFDEAIQGCSGVFHVATPMDFESKDPE.................
BAF49325.1_DFR_Delphinium_belladonna        KSKLTLWKADLTE.DGSYDDAIKGCTGVFHVATPMDFESKDPE.................
CAA78930.1_DFR_Gerbera_hybrid_cultivar      QTNLKLWKADLTQ.EGSFDEAIQGCHGVFHLATPMDFESKDPE.................
AAO39816.1_DFR_Malus_domestica              ETHLTLWKADLAD.EGSFDEAIQGCSGVFHVATPMDFESKDPE.................
AAO39817.1_DFR_Malus_domestica              ETHLTLWKADLAD.EGSFDEAIQGCSGVFHVATPMDFESKDPE.................
AAR27015.1_DFR2_Medicago_truncatula         NSKLSLWKADLGE.EGSFDEAIKGCTGVFHVATPMDFESKDPE.................
AEF14420.1_DFR_Onobrychis_viciifolia        KTKLSLWKADLAH.EGSFDEAIKGCTGVFHVATPMDFDSKDPE.................
AAF60298.1_DFR_Petunia_x_hybrida            DTNLTLWKADLTV.EGSFDEAIQGCQGVFHVATPMDFESKDPE.................
AAQ77347.1_DFR_Triticum_aestivum            KERLSIWKADLSE.EGSFDDAIAGCTGVFHVATPMDFDSKDPE.................
AAL89715.1_DFR_Vaccinium_macrocarpon        DTNLTLWKADLNE.EGSFDEAIEGCVGVFHVATPMDFESKDPE.................
AAX12420.1_DFR_Vaccinium_macrocarpon        DTNLTLWKADLNE.EGSFDEAIEGCVGVFHVATPMDFESKDPE.................
CAA33543.1_DFR_Antirrhinum_majus            DTNLTLWKADMTV.EGSFDEAIQGCEGVFHLATSMEFDSVDPE.................
AAB62873.1_DFR_Bromheadia_finlaysoniana     NELLSIWKADLNDIEGSFDEVIRGCVGVFHVATPMNFQSKDPE.................
BAA84940.1_DFR_Camellia_sinensis            DTNLTLWKADLNE.EGSFDEAIEGCSGVFHVATPMDFESKDPE.................
AAC17843.1_DFR_Cymbidium_hybrid_cultivar    NELLSIWKADLNDIDETFDEVTRGSVGLFHVATPMNFQSEDPE.................
CAA91924.1_DFR_Dianthus_caryophyllus        KTNLTLWKADLHE.EGSFDAAVDGCTGVFHIATPMDFESKDPE.................
CAA70345.1_DFR_Forsythia_x_intermedia       DTNLTLWKADMTV.EGSFDEAIQGCEGVFHVATPMDFESKDPE.................
AAC25960.1_DFR_Fragaria_x_ananassa          ATRLTLWKADLDV.EGSFDEAIKGCTGVFHVATPMDFESEDPE.................
BAA12736.1_DFR_Gentiana_triflora            STNLTLLKADLTE.EGSFDEAIHGCHGVFHVATPMDFESKDPK.................
AAD54273.1_DFR1_Glycine_max                 KSKLSLWKADLAE.EGSFDEAIKGCTGVFHVATPMDFESKDPE.................
BAA59332.1_DFR_Ipomoea_nil                  EGKLKVWKGVLEE.EGSFDEAIAGCEGVFHVAAAVNFASKDPE.................
BAA36405.1_DFR_Ipomoea_purpurea             EGKLKVWKGVLEE.EGSFDEAIAGCEGVFHVAAAVNFASKDPE.................
AAD49343.1_DFR_Lilium_hybrid_division_VII   DERLTIWKADLSE.DASFDEAINGCTGVYHVATPMDFDSKDPE.................
AAF23884.2_DFR3_Lotus_corniculatus          KTKPTLWKADLAE.EGSFDEAIKGCTGVFHVATPMDFESKNPE.................
BAE19948.1_DFR_Lotus_japonicus              KTNLTIWNADLTE.EGSFDEAIKGCSGVFHVASPMDFNSKDPE.................
BAE19949.1_DFR_Lotus_japonicus              KTKLTLWKADLAE.EGSFDEAIKGCTGVFHVATPMDFESKDPE.................
BAE19950.1_DFR_Lotus_japonicus              KTNLTIWNADLTE.EGSFDEAINGCSGVFHVASPMDFNSKDPE.................
BAE19951.1_DFR_Lotus_japonicus              KTNLTIWNADLTE.EGSFDEAINGCSGVFHLATPMDFNSKDPE.................
BAE19953.1_DFR_Lotus_japonicus              KTKLSLWKADLAE.EGSFDEAIRGCTGVFHVATPMDFESKDPE.................
BAA19658.1_DFR_Perilla_frutescens           DTNLTLWRADMNI.EGSYDEAVQGCECVFHMATPMDFESDDPE.................
BAB20075.1_DFR_Torenia_hybrid_cultivar      DASLRLFKADMNV.EGSFDEAVGSCECVFHMATPMDFESDDPE.................
AAL35830.1_DFR_Triticum_monococcum          KERLSIWKADLSD.QGSFDDAIAGCTGVFHVATPNGLRLPRPRGKYQYPGTLKQSITTEI
QFQ61498.1_DFR_Dryopteris_erythrosora       AERLEIVKADLRV.KGDFDKVAQGCDGVFHLACPTNFLVEDPQ.................
QFQ61499.1_DFR_Dryopteris_erythrosora       DERLQIVRGQLLE.ESGFDDAVNGCDGVFHCACPTEFSFEDPY.................
BAA74700.1_DFR_Ipomoea_purpurea             DTNLTVWKGVMEE.EGSFDEAIAGCEGVFHVATPMDFDSKDPE.................
BAF93896.1_DFR_Iris_x_hollandica            DALLTIWKADLGQ.DGSFDKAVAGCTAVFHVATPMDFESKDPE.................
XP_008797532.1_DFR_Phoenix_dactylifera      SERVTIWKADLDE.EGSFDEAIEGCSGVFHVATPMDFESKDPE.................
AEQ92209.1_DFR_Ipomoea_batatas              DTNLRVWKGVMEE.EGSFDEAIAGCEGVFHVATPMDFDSKDPE.................
BAH98155.1_DFR_Tulipa_gesneriana            DERLTIWKADLND.EGSFDNAINGCTGVFHVATPMDFESKDPE.................
APG32494.1_DFR2_Freesia_hybrid_cultivar     DRLLTIWKADLGE.EGSFDEVVQGCTAVFHVATPMDFESQDPE.................
AAO63026.1_DFR_Allium_cepa                  DTRLSLWEADLLQ.DGSFDHVISGSIAVFHVATPMDFDSIDPE.................
AFP58815.1_DFR_Hyacinthus_orientalis        GSRLTIWKADLND.EGSFDDVIDGCTGVFHVATPMDFESKDPE.................
                                      acc
```

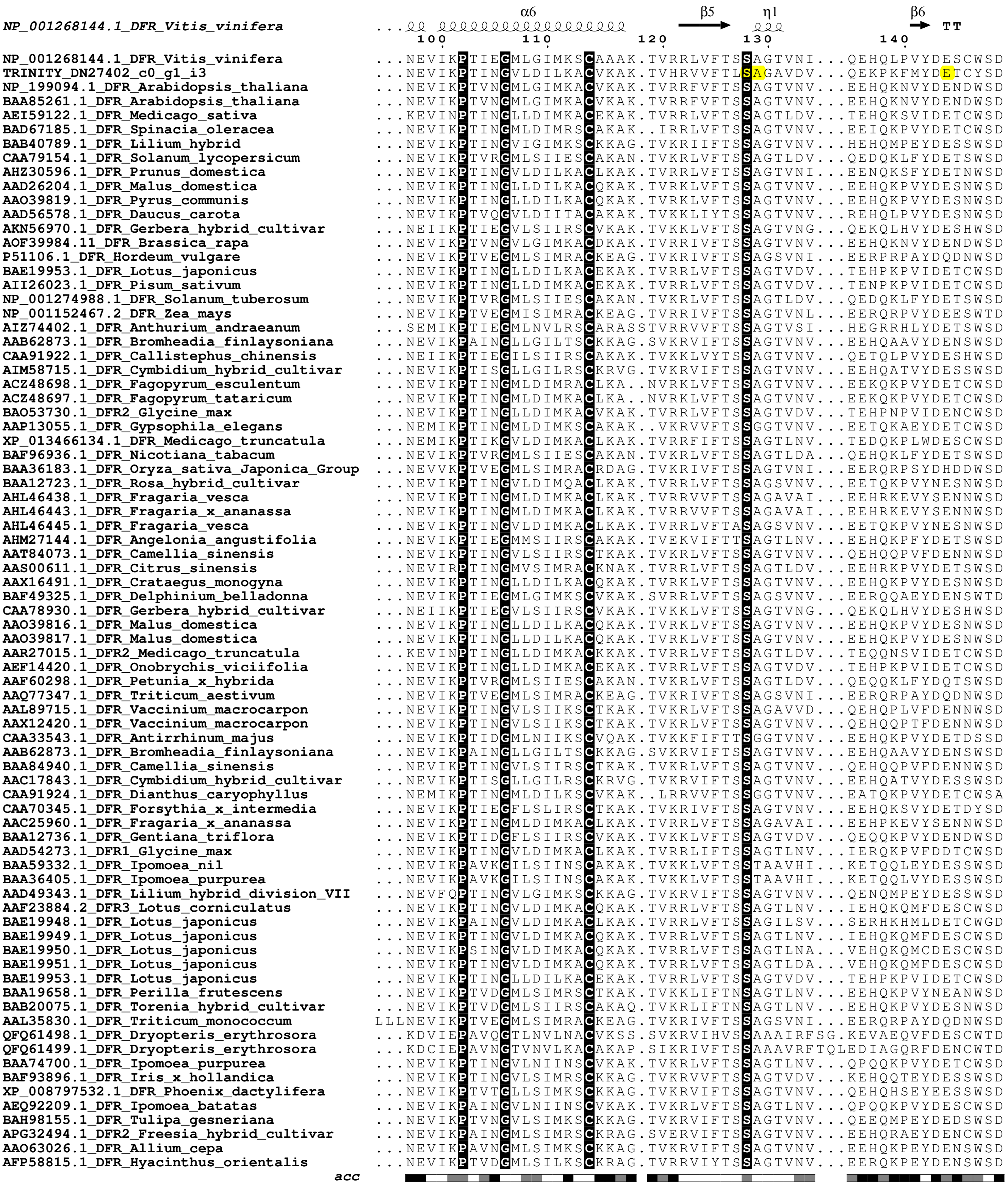

α6
β5
η1
β6
TT
NP_001268144.1_DFR_Vitis_vinifera
100
110
120
130
140
NP_001268144.1_DFR_Vitis_vinifera ...NEVIKPTIEGMLGIMKSCAAAK.TVRRLVFTSSAGTVNI...QEHQLPVYDESCWSD
TRINITY_DN27402_c0_g1_i3 ...NEVIKPTINGVLDILKACVKAK.TVHRVVFTLSAGAVDV...QEKPKFMYDETCYSD
NP_199094.1_DFR_Arabidopsis_thaliana ...NEVIKPTVNGMLGIMKACVKAK.TVRRFVFTSSAGTVNV...EEHQKNVYDENDWSD
BAA85261.1_DFR_Arabidopsis_thaliana ...NEVIKPTVNGMLGIMKACVKAK.TVRRFVFTSSAGTVNV...EEHQKNVYDENDWSD
AEI59122.1_DFR_Medicago_sativa ...KEVINPTINGLLDIMKACEKAK.TVRRLVFTSSAGTLDV...TEHQKSVIDETCWSD
BAD67185.1_DFR_Spinacia_oleracea ...NEVIKPTINGMLDIMRSCAKAK..IRRLVFTSSAGTVNV...EEIQKPVYDETCWSD
BAB40789.1_DFR_Lilium_hybrid ...NEVIKPTINGVIGIMKSCKKAG.TVKRIIFTSSAGTVNV...HEHQMPEYDESSWSD
CAA79154.1_DFR_Solanum_lycopersicum ...NEVIKPTVRGMLSIIESCAKAN.TVKRLVFTSSAGTLDV...QEDQKLFYDETSWSD
AHZ30596.1_DFR_Prunus_domestica ...NEVIKPTINGVLDILKACLKAK.TVRRLVFTSSAGTVNI...EENQKSFYDETNWSD
AAD26204.1_DFR_Malus_domestica ...NEVIKPTINGLLDILKACQKAK.TVRKLVFTSSAGTVNV...EEHQKPVYDESNWSD
AAO39819.1_DFR_Pyrus_communis ...NEVIKPTINGLLDILKACQKAK.TVRKLVFTSSAGTVNV...EEHQKPVYDESNWSD
AAD56578.1_DFR_Daucus_carota ...NEVIKPTVQGVLDIITACAKAK.TVKKLIYTSSAGTVNV...REHQLPVYDESNWSD
AKN56970.1_DFR_Gerbera_hybrid_cultivar ...NEIIKPTIEGVLSIIRSCVKAK.TVKKLVFTSSAGTVNG...QEKQLHVYDESHWSD
AOF39984.11_DFR_Brassica_rapa ...NEVIKPTVNGVLGIMKACDKAK.TVRRIVFTSSAGTVNV...EEHQKNVYDENDWSD
P51106.1_DFR_Hordeum_vulgare ...NEVIKPTVEGMLSIMRACKEAG.TVKRIVFTSSAGSVNI...EERPRPAYDQDNWSD
BAE19953.1_DFR_Lotus_japonicus ...NEVIKPTINGLLDILKACEKAK.TVRRLVFTSSAGTVDV...TEHPKPVIDETCWSD
AII26023.1_DFR_Pisum_sativum ...NEVIKPTINGLLDIMKACEKAK.TVRRLVFTSSAGTVDV...TENPKPVIDETCWSD
NP_001274988.1_DFR_Solanum_tuberosum ...NEVIKPTVRGMLSIIESCAKAN.TVKRLVFTSSAGTLDV...QEDQKLFYDETSWSD
NP_001152467.2_DFR_Zea_mays ...NEVIKPTVEGMISIMRACKEAG.TVRRIVFTSSAGTVNL...EERQRPVYDEESWTD
AIZ74402.1_DFR_Anthurium_andraeanum ...SEMIKPTIEGMLNVLRSCARASSTVRRVVFTSSAGTVSI...HEGRRHLYDETSWSD
AAB62873.1_DFR_Bromheadia_finlaysoniana ...NEVIKPAINGLLGILTSCKKAG.SVKRVIFTSSAGTVNV...EEHQAAVYDENSWSD
CAA91922.1_DFR_Callistephus_chinensis ...NEIIKPTIEGILSIIRSCAKAK.TVKKLVYTSSAGTVNV...QETQLPVYDESHWSD
AIM58715.1_DFR_Cymbidium_hybrid_cultivar ...NEVIKPTISGLLGILRSCKRVG.TVKRVIFTSSAGTVNV...EEHQATVYDESSWSD
ACZ48698.1_DFR_Fagopyrum_esculentum ...NEVIKPTINGMLDIMRACLKA..NVRKLVFTSSAGTVNV...EEKQKPVYDETCWSD
ACZ48697.1_DFR_Fagopyrum_tataricum ...NEVIKPTINGMLDIMKACLKA..NVRKLVFTSSAGTVNV...EEKQKPVYDETCWSD
BAO53730.1_DFR2_Glycine_max ...NEVIKPTINGLLDIMKACVKAK.TVRRLVFTSSAGTVDV...TEHPNPVIDENCWSD
AAP13055.1_DFR_Gypsophila_elegans ...NEMIKPTINGMLDIMKSCAKAK..VKRVVFTSSGGTVNV...EETQKAEYDETCWSD
XP_013466134.1_DFR_Medicago_truncatula ...NEMIKPTIKGVLDIMKACLKAK.TVRRFIFTSSAGTLNV...TEDQKPLWDESCWSD
BAF96936.1_DFR_Nicotiana_tabacum ...NEVIKPTVRGMLSIIESCAKAN.TVKRLVFTSSAGTLDA...QEHQKLFYDETSWSD
BAA36183.1_DFR_Oryza_sativa_Japonica_Group ...NEVVKPTVEGMLSIMRACRDAG.TVKRIVFTSSAGTVNI...EERQRPSYDHDDWSD
BAA12723.1_DFR_Rosa_hybrid_cultivar ...NEVIKPTINGVLDIMQACLKAK.TVRRLVFTSSAGSVNV...EETQKPVYNESNWSD
AHL46438.1_DFR_Fragaria_vesca ...NEVIKPTINGMLDIMKACLKAK.TVRRVVFTSSAGAVAI...EEHRKEVYSENNWSD
AHL46443.1_DFR_Fragaria_x_ananassa ...NEVIKPTINGMLDIMKACLKAK.TVRRVVFTSSAGAVAI...EEHRKEVYSENNWSD
AHL46445.1_DFR_Fragaria_vesca ...NEVIKPTINGVLDIMKACLKAK.TVRRLVFTASAGSVNV...EETQKPVYNESNWSD
AHM27144.1_DFR_Angelonia_angustifolia ...NEVIKPTIEGMMSIIRSCAKAK.TVEKVIFTTSAGTLNV...EEHQKPFYDETSWSD
AAT84073.1_DFR_Camellia_sinensis ...NEVIKPTINGVLSIIRSCTKAK.TVKRLVFTSSAGTVNV...QEHQQPVFDENNWSD
AAS00611.1_DFR_Citrus_sinensis ...NEVIRPTINGMVSIMRACKNAK.TVRRLVFTSSAGTLDV...EEHRKPVYDETSWSD
AAX16491.1_DFR_Crataegus_monogyna ...NEVIKPTINGLLDILKACQKAK.TVRKLVFTSSAGTVNV...EEHQKPVYDESNWSD
BAF49325.1_DFR_Delphinium_belladonna ...NEVIKPTIEGMLGIMKSCVKAK.SVRRLVFTSSAGSVNV...EERQQAEYDENSWTD
CAA78930.1_DFR_Gerbera_hybrid_cultivar ...NEIIKPTIEGVLSIIRSCVKAK.TVKKLVFTSSAGTVNG...QEKQLHVYDESHWSD
AAO39816.1_DFR_Malus_domestica ...NEVIKPTINGLLDILKACQKAK.TVRKLVFTSSAGTVNV...EEHQKPVYDESNWSD
AAO39817.1_DFR_Malus_domestica ...NEVIKPTINGLLDILKACQKAK.TVRKLVFTSSAGTVNV...EEHQKPVYDESNWSD
AAR27015.1_DFR2_Medicago_truncatula ...KEVINPTINGLLDIMKACKKAK.TVRRLVFTSSAGTLDV...TEQQNSVIDETCWSD
AEF14420.1_DFR_Onobrychis_viciifolia ...NEVIKPTINGLLDIMKACEKAK.TVRRLVFTSSAGTVDV...TEHPKPVIDETCWSD
AAF60298.1_DFR_Petunia_x_hybrida ...NEVIKPTVRGMLSIIESCAKAN.TVKRLVFTSSAGTLDV...QEQQKLFYDQTSWSD
AAQ77347.1_DFR_Triticum_aestivum ...NEVIKPTVEGMLSIMRACKEAG.TVKRIVFTSSAGSVNI...EERQRPAYDQDNWSD
AAL89715.1_DFR_Vaccinium_macrocarpon ...NEVIKPTINGVLSIIKSCTKAK.TVKRLVFTSSAGAVVD...QEHQPLVFDENNWSD
AAX12420.1_DFR_Vaccinium_macrocarpon ...NEVIKPTINGVLSIIKSCTKAK.TVKRLVFTSSAGTVNV...QEHQQPTFDENNWSD
CAA33543.1_DFR_Antirrhinum_majus ...NEVIKPTIDGMLNIIKSCVQAK.TVKKFIFTTSGGTVNV...EEHQKPVYDETDSSD
AAB62873.1_DFR_Bromheadia_finlaysoniana ...NEVIKPAINGLLGILTSCKKAG.SVKRVIFTSSAGTVNV...EEHQAAVYDENSWSD
BAA84940.1_DFR_Camellia_sinensis ...NEVIKPTINGVLSIIRSCTKAK.TVKRLVFTSSAGTVNV...QEHQQPVFDENNWSD
AAC17843.1_DFR_Cymbidium_hybrid_cultivar ...NEVIKPTISGLLGILRSCKRVG.TVKRVIFTSSAGTVNV...EEHQATVYDESSWSD
CAA91924.1_DFR_Dianthus_caryophyllus ...NEMIKPTINGMLDILKSCVKAK..LRRVVFTSSGGTVNV...EATQKPVYDETCWSA
CAA70345.1_DFR_Forsythia_x_intermedia ...NEVIKPTIEGFLSLIRSCTKAK.TVKRIVFTSSAGTVNV...EEHQKSVYDETDYSD
AAC25960.1_DFR_Fragaria_x_ananassa ...NEVIKPTINGMLDIMKACLKAK.TVRRLVFTSSAGAVAI...EEHPKEVYSENNWSD
BAA12736.1_DFR_Gentiana_triflora ...NEVIKPTIDGFLSIIRSCVKAK.TVKKLVFTSSAGTVDV...QEQQKPVYDENDWSD
AAD54273.1_DFR1_Glycine_max ...NEVIKPTINGVLDIMKACLKAK.TVRRLIFTSSAGTLNV...IERQKPVFDDTCWSD
BAA59332.1_DFR_Ipomoea_nil ...NEVIKPAVKGILSIINSCAKAK.TVKKLVFTSSSTAAVHI...KETQQLEYDESSWSD
BAA36405.1_DFR_Ipomoea_purpurea ...NEVIKPAVKGILSIINSCAKAK.TVKKLVFTSSSTAAVHI...KETQQLVYDESSWSD
AAD49343.1_DFR_Lilium_hybrid_division_VII ...NEVFQPTINGVLGIMKSCKKAG.TVKRVIFTSSAGTVNV...QENQMPEYDESSWSD
AAF23884.2_DFR3_Lotus_corniculatus ...NEVIKPTINGVLDIMKACQKAK.TVRRLVFTSSAGTLNV...IEHQKQMFDESCWSD
BAE19948.1_DFR_Lotus_japonicus ...NEVIKPAINGVLDIMKACLKAK.TVRRLVFTSSAGILSV...SERHKHMLDETCWGD
BAE19949.1_DFR_Lotus_japonicus ...NEVIKPTINGVLDIMKACQKAK.TVRRLVFTSSAGTLNV...IEHQKQMFDESCWSD
BAE19950.1_DFR_Lotus_japonicus ...NEVIKPSINGVLDIMKACQKAK.TVRRLVFTSSAGTLNA...VEHQKQMCDESCWSD
BAE19951.1_DFR_Lotus_japonicus ...NEVIKPTINGVLDIMKACQKAK.TVRRLVFTSSAGTLDA...VEHQKQMFDESCWSD
BAE19953.1_DFR_Lotus_japonicus ...NEVIKPTINGLLDILKACEKAK.TVRRLVFTSSAGTVDV...TEHPKPVIDETCWSD
BAA19658.1_DFR_Perilla_frutescens ...NEVIKPTVDGMLSIMRSCTKAK.TVKKLIFTNSAGTLNV...EEHQKPVYNEANWSD
BAB20075.1_DFR_Torenia_hybrid_cultivar ...NEVIKPTVDGMLSIIRSCAKAQ.TVKRLIFTNSAGTLNV...EEHQKPVYDESNWSD
AAL35830.1_DFR_Triticum_monococcum LLLNEVIKPTVEGMLSIMRACKEAG.TVKRIVFTSSAGSVNI...EERQRPAYDQDNWSD
QFQ61498.1_DFR_Dryopteris_erythrosora ...KDVIEPAVQGTLNVLNACVKSS.SVKRVIHVSSAAAIRFSG.KEVAEQVFDESCWTD
QFQ61499.1_DFR_Dryopteris_erythrosora ...KDCIEPAVNGTVNVLKACAKAP.SIKRIVFTSSAAAVRFTQLEDIAGQRFDENCWTD
BAA74700.1_DFR_Ipomoea_purpurea ...NEVIKPTINGVLNIINSCVKAK.TVKRLVFTSSAGTLNV...QPQQKPVYDETCWSD
BAF93896.1_DFR_Iris_x_hollandica ...NEVIKPTINGMLSIMRSCKKAG.TVKRVVFTSSAGTVDV...KEHQQTEYDESSWSD
XP_008797532.1_DFR_Phoenix_dactylifera ...NEVIKPTVTGLLSIMKSCKKAG.TVKRVIFTSSAGTVNV...QEHQHSEYEESSWSD
AEQ92209.1_DFR_Ipomoea_batatas ...NEVIKPAINGVLNIINSCVKAK.TVKRLVFTSSAGTLNV...QPQQKPVYDESCWSD
BAH98155.1_DFR_Tulipa_gesneriana ...NEVIKPTVNGVLSIMKSCKKAG.TVKRVIFTSSAGTVNV...QEEQMPEYDEDSWSD
APG32494.1_DFR2_Freesia_hybrid_cultivar ...NEVIKPAINGMLSIMRSCKRAG.SVERVIFTSSAGTVNV...EEHQRAEYDENCWSD
AAO63026.1_DFR_Allium_cepa ...NEVIKPAVNGMLSIMKSCKKAG.TVKRVIFTSSAGTVNV...EEHQKPEYDENSWSD
AFP58815.1_DFR_Hyacinthus_orientalis ...NEVIKPTVDGMLSILKSCKRAG.TVRRVIYTSSAGTVNV...EERQKPEYDENSWSD
acc

```
                                            α7                  α8                      β7    β8          α9
NP_001268144.1_DFR_Vitis_vinifera
                                   150       160 ★     170       180       190       200
NP_001268144.1_DFR_Vitis_vinifera           MEFCRAKKMTAWMYFVSKTLAEQAAWKYAKENNIDFITIIPTLVVGPFIMSSMPPSLITA
TRINITY_DN27402_c0_g1_i3                    LDFILSKKMTGWMYFVAKTIAEKEAFKFAKENNLDFISIIPSLVVGQFLMPSLPPSLITA
NP_199094.1_DFR_Arabidopsis_thaliana        LEFIMSKKMTGWMYFVSKTLAEKAAWDFAEEKGLDFISIIPTLVVGPFITTSMPPSLITA
BAA85261.1_DFR_Arabidopsis_thaliana         LEFIMSKKMTGWMYFVSKSLAEKAAWDFAEEKGLDFISIIPTLVVGPFITTSMPPSLITA
AEI59122.1_DFR_Medicago_sativa              VEFCRRVKMTGWMYFVSKTLAEQEAWKFSKEHNIDFVSIIPPLVVGPFIMPSMPPSLITA
BAD67185.1_DFR_Spinacia_oleracea            MEFCSSKKMTGWMYFVSKTLAEKAAWKFAAENNLDFISIIPPLVVGPFITPTMPPSLITA
BAB40789.1_DFR_Lilium_hybrid                IDFIRRVKMTGWMYFVSKILAEKAAWDFAKENDIQFISIIPTLVVGPFITTTMPPSMLTA
CAA79154.1_DFR_Solanum_lycopersicum         LDFIYAKKMTGWMYFVSKILAEKAAMEEARKNNIDFISIIPPLVVGPFITSTFPPSLITA
AHZ30596.1_DFR_Prunus_domestica             VEFCRSVKMTGWMYFVSKTLAEQAAWKFAKENNIDFITIIPTLVIGPFLMPSMPPSLITG
AAD26204.1_DFR_Malus_domestica              VEFCRSVKMTGWMYFVSKTLAEQAAWKYAKENNIDFITIIPTLVIGPFLMPSMPPSLITG
AAO39819.1_DFR_Pyrus_communis               VEFCRSVKMTGWMYFVSKTLAEQAAWKYAKENNIDFITIIPTLVIGPFLMPSMPPSLITG
AAD56578.1_DFR_Daucus_carota                MDFIYSTKMTAWMYFVSKSLAEKAAWQAAEENNIQFISIIPTLVVGPFISPTFPPSLITA
AKN56970.1_DFR_Gerbera_hybrid_cultivar      LDFIYSKKMTAWMYFVSKTLAEKAAWDAMKGNNISFISIIPTLVVCPFITSTFPPSLVTA
AOF39984.11_DFR_Brassica_rapa               LDFIMSKKMTGWMYFMSKTLAEKAAWDYAKEKGIDFISIIPTLVIGPFITTSMPPSLVTA
P51106.1_DFR_Hordeum_vulgare                IDYCRRVKMTGWMYFVSKALAEKAAMEYASENGLDFISIIPTLVVGPFLSAGMPPSLVTA
BAE19953.1_DFR_Lotus_japonicus              IEFCLRVKMTGWMYFVSKTRAEQEAWKYAKEHNIDFVSVIPPLVVGPFLMPTMPPSLITA
AII26023.1_DFR_Pisum_sativum                VDFCRRVNMTGWMYFVSKTLAEQEAWKYSKEHKIDFVSIIPPLVVGPFLMPSMPPSLITA
NP_001274988.1_DFR_Solanum_tuberosum        LDFIYAKKMTGWMYFVSKILAEKAAMKEAKKNNINFISIIPPLVVGPFITPTFPPSLITA
NP_001152467.2_DFR_Zea_mays                 VDFCRRVKMTGWMYFVSKTLAEKAALAYAAEHGLDLVTIIPTLVVGPFISASMPPSLITA
AIZ74402.1_DFR_Anthurium_andraeanum         VDFCRAKKMTGWMYFVSKTLAEKAAWDFAEKNNIDFISIIPTLVNGPFVMPTMPPSMLSA
AAB62873.1_DFR_Bromheadia_finlaysoniana     LHFVTRVKMTGWMYFVSKTLAEKAAWEFVKENAIHFIAIIPTLVVGSFITNEMPPSLITA
CAA91922.1_DFR_Callistephus_chinensis       LDFIYSKKMTAWMYFVSKTLAEKAAMEAAKENNIDFVSIIPPLVVGPFINPTFPPSLITA
AIM58715.1_DFR_Cymbidium_hybrid_cultivar    LDFVTRVKMTGWMYFVSKTLAEKAAWEFVSDNDIHFITIIPTLVVGSFLISRMPPSLITA
ACZ48698.1_DFR_Fagopyrum_esculentum         VDFCRRVKMTGWMYFVSKTLAEQAAWKFAEENNMDFISIIPTLVVGPFIMPSFPPSLITA
ACZ48697.1_DFR_Fagopyrum_tataricum          VDFCRRVKMTGWMYFVSKTLAEQAAWKFAEENNMDFISIIPTLVVGPFIMPSFPPSLITA
BAO53730.1_DFR2_Glycine_max                 VDFCTRVKMTGWMYFVSKTLAEQEAWKYAKEHNIDFISVIPPLVVGPFLMPTMPPSLITA
AAP13055.1_DFR_Gypsophila_elegans           LDFIRSVKMTGWMYFVSKILAEQAAWKYAAENNLDFISIIPPLVVGPFIMPSMPPSLITA
XP_013466134.1_DFR_Medicago_truncatula      VEFCRRVKMTGWMYFVSKTLAEQEAWKFAKEHNMDFITIIPPLVVGPFLIPTMPPSLITA
BAF96936.1_DFR_Nicotiana_tabacum            LDFIYAKKMTGWMYFVSKILAEKAAMEAAKKKNFDFISIIPPLVVGPFLTPTFPPSLITA
BAA36183.1_DFR_Oryza_sativa_Japonica_Group  IDFCRRVKMTGWMYFVSKSLAEKAAMEYAREHGLDLISVIPTLVVGPFISNGMPPSHVTA
BAA12723.1_DFR_Rosa_hybrid_cultivar         VEFCRRVKMTGWMYFASKTLAEQEAWKFAKENNIDFITIIPTLVIGPFLMPSMPPSLITG
AHL46438.1_DFR_Fragaria_vesca               VVFCRKVKMTGWMYFVSKTLAEQAAWKFAKENNIDFITIIPTLVIGPFLAPSMPPSLISG
AHL46443.1_DFR_Fragaria_x_ananassa          VVFCRKVKMTGWMYFVSKTLAEQAAWKFAKENNIDFITIIPTLVIGPFLAPSMPPSLISG
AHL46445.1_DFR_Fragaria_vesca               VEFCRRVKMTGWMYFVSKTLAEQEAWKFAKENNIDFITIIPTLVIGPFLMPAMPPSLITG
AHM27144.1_DFR_Angelonia_angustifolia       LDFIYSKKMTGWMYFVSKYLAEKQAVEAAKLNNIEFISIIPPVVVGPFINPAFPPSLVTA
AAT84073.1_DFR_Camellia_sinensis            LDFINKKKMTGWMYFVSKTLAEKAAWEAAKENNIDFISIIPTLVVGPFIMPTFPPSLITA
AAS00611.1_DFR_Citrus_sinensis              LDFVRSVKMTGWMYFVSKTLAEQAAWKFAEENNIDFISIIPSLVVGPFLTSSMPPSLITA
AAX16491.1_DFR_Crataegus_monogyna           VEFCRSVKMTGWMYFVSKTLAEQAAWKYAKENNIDFITIIPTLVIGPFLMPSMPPSLITG
BAF49325.1_DFR_Delphinium_belladonna        VEFCRTRKMTGWMYFVSKTLAEKAAWEFAQQNNIDFISIIPTLVVGPFLMPSMPPSLITA
CAA78930.1_DFR_Gerbera_hybrid_cultivar      LDFIYSKKMTAWMYFVSKTLAEKAAWDATKGNNISFISIIPTLVVGPFITSTFPPSLVTA
AAO39816.1_DFR_Malus_domestica              VEFCRSVKMTGWMYFVSKTLAEQAAWKYAKENNIDFITIIPTLVIGPFLMPSMPPSLITG
AAO39817.1_DFR_Malus_domestica              VEFCRSVKMTGWMYFVSKTLAEQAAWKYAKENNIDFITIIPTLVIGPFLMPSMPPSLITG
AAR27015.1_DFR2_Medicago_truncatula         VEFCRRVKMTGWMYFVSKTLAEQEAWKFSKEHNIDFVSIIPPLVVGPFIMPSMPPSLITA
AEF14420.1_DFR_Onobrychis_viciifolia        IEFCRRVKMTGWMYFVSKTLAEQEAWKYAKEHNIDFISVIPPLVVGPFLMPTMPPSLITA
AAF60298.1_DFR_Petunia_x_hybrida            LDFIYAKKMTGWMYFASKILAEKAAMEEAKKKNIDFISIIPPLVVGPFITPTFPPSLITA
AAQ77347.1_DFR_Triticum_aestivum            IDFCRRVKMTGWMYFVSKSLAEKAAMEYASENGLDFISIIPTLVVGPFLSAGMPPSLVTA
AAL89715.1_DFR_Vaccinium_macrocarpon        VDFLYDKKMTGWTYFVSKTLAERAAMEAAKEISIDFISIIPTLVVGPFISPTFPPSLITV
AAX12420.1_DFR_Vaccinium_macrocarpon        LDFIYKIKMTGWMYFVSKTLAEKAAWEAAKENNIDFISIIPTLVVGPFIMPTFPPSLITA
CAA33543.1_DFR_Antirrhinum_majus            MDFINSKKMTGWMYFVSKILAEKAGMEAAKENNIDFISIIPPLVVGPFIMPTFPPSLITA
AAB62873.1_DFR_Bromheadia_finlaysoniana     LHFVTRVKMTGWMYFVSKTLAEKAAWEFVKENAIHFIAIIPTLVVGSFITNEMPPSLITA
BAA84940.1_DFR_Camellia_sinensis            LDFINKKKMTGWMYFVSKTLAEKAAWEAAKENNIDFISIIPTLVVGPFIMPTFPPSLITA
AAC17843.1_DFR_Cymbidium_hybrid_cultivar    LDFVTRVKMTGWMYFVSKTLAEKAAWEFVSDNDIHFITIIPTLVVGSFLISRMPPSLITA
CAA91924.1_DFR_Dianthus_caryophyllus        LDFIRSVKMTGWMYFVSKILAEQAAWKYAAENNLEFISIIPPLVVGPFIMPSMPPSLITA
CAA70345.1_DFR_Forsythia_x_intermedia       LNFIYSKKMTGWMYFASKILAEKVAWEAAKENSIGFISIIPTLVVGPFIMPTFPPSLITA
AAC25960.1_DFR_Fragaria_x_ananassa          VVFCRKVKMTGWMYFVSKTLAEQAAWKFAKENNIDFITIIPTLVIGPFLAPSMPPSLISG
BAA12736.1_DFR_Gentiana_triflora            LDFINSTKMTGWMYFVSKILAEKAAWEVTKANDIGFISIIPTLVVGPFITTTFPPSLITA
AAD54273.1_DFR1_Glycine_max                 VEFCRRVKMTGWMYFVSKTLAEKEAWKFAKEQGLDFITIIPPLVVGPFLMPTMPPSLITA
BAA59332.1_DFR_Ipomoea_nil                  LDFIYANKMGGWMYFASKTLAEKEAWKAAKEKQIEFISIIPPLVIGPFLIPTFPLSLVTA
BAA36405.1_DFR_Ipomoea_purpurea             LDFIYANKMGGWMYFASKTLAEKEAWKAAKEKQIEFISIIPPLVIGPFLIPTFPLSLVTA
AAD49343.1_DFR_Lilium_hybrid_division_VII   VDFCRRVKMTGWMYFVSKTLAEKAAWEFAKENDIQLISIIPTLVVGPFITSTMPPSMLTA
AAF23884.2_DFR3_Lotus_corniculatus          VEFCRRVKMTGWMYFVSKTLAEQEAWKFAKEHGIDFITIIPPLVVGSFLMPTMPPSLITA
BAE19948.1_DFR_Lotus_japonicus              LEFCKKVKMTGWMYFVSKELAEQEALKFAKENNIDFVSIIPSLVVGPFLMPTMPPSLYTA
BAE19949.1_DFR_Lotus_japonicus              VEFCRRVKMTGWMYFVSKTLAEQEAWKFAKEHGIDFITIIPPLVVGSFLMPTMPPSLITA
BAE19950.1_DFR_Lotus_japonicus              VEFCRRVKMTGWMYFVSKTLAEQEAWKFAQEHDIDFITIIPSLVVGSFLMPTLPPSLTTA
BAE19951.1_DFR_Lotus_japonicus              VEFCRRVKMTGWMYFVSKTLAEQEAWKFAKEHDIDFITTIPSLVVGSFLMPTMPPSLITA
BAE19953.1_DFR_Lotus_japonicus              IEFCLRVKMTGWMYFVSKTRAEQEAWKYAKEHNIDFVSVIPPLVVGPFLMPTMPPSLITA
BAA19658.1_DFR_Perilla_frutescens           LDFIYSKKMTGWMYFVSKILAEKEAMKAAKESNINFISIIPPVVVGPFFMPTFPPSLITA
BAB20075.1_DFR_Torenia_hybrid_cultivar      LDFIYSTKMTGWMYFVSKVLAEKAAIKACKENNIDFISIIPPVVVGPFIIDNWPPSLITA
AAL35830.1_DFR_Triticum_monococcum          IDFCRRVKMTGWMYFVSKALAEKAAMEYASENGLDFISIIPTLVVGPFLSAGMPPSLVTA
QFQ61498.1_DFR_Dryopteris_erythrosora       VDFCIDNKIPGWPYFVGKTLGEKAAVEFAKEHNLDLVVVNPSIVHGPFLLSNIPNSVKDC
QFQ61499.1_DFR_Dryopteris_erythrosora       IEMCERDKPHGWPYFVSKTLSEKKAFELAQEYNLDLVTILPPLVNGPFLIDKIPNSVADA
BAA74700.1_DFR_Ipomoea_purpurea             LDFIYAKKMTGWMYFASKILAEKEAWKATKEKKIDFISIIPPLVVGPFITPTFPPSLITA
BAF93896.1_DFR_Iris_x_hollandica            VDFCRRVKMTGWMYFVSKTLAERATWEFARENGIDFISIIPTLVVGPFITTTMPPSMVTA
XP_008797532.1_DFR_Phoenix_dactylifera      IEFCRRVKMTGWMYFVSKTLAEQAAWEFARENGIHFISIIPTLVVGPFISSSMPPSLITA
AEQ92209.1_DFR_Ipomoea_batatas              LDFIYAKKMTGWMYFASKILAEKEAWKATKEKKIDFISIIPPLVVGPFITPTFPPSLITA
BAH98155.1_DFR_Tulipa_gesneriana            IDFCRRVKMTGWMYFVSKTLAEKAAWEFAKDNDIQLISIIPTLVVGPFITTSMPPSMITA
APG32494.1_DFR2_Freesia_hybrid_cultivar     VEFCRRVKMTGWMYFVSKTLAEKAAWDFALENGIHLITIIPTLVVGPFITTTMPPSMITA
AAO63026.1_DFR_Allium_cepa                  IDFCRRVKMTGWMYFVSKSLAEKAAWEFAKANGIDLVTIIPTLVVGAFITTAMPPSMITA
AFP58815.1_DFR_Hyacinthus_orientalis        IEFCRRVKMTGWMYFVSKSLAERAAWDFARENSMDLITIIPTLVVGPFITSTMPPSMITA
acc
```

```
                                                α10      η2   α11       β9                α12                 β10       β11        α13
NP_001268144.1_DFR_Vitis_vinifera
                                       210       220       230       240       250       260
NP_001268144.1_DFR_Vitis_vinifera      LSPITGNEAHYSIIRQGQFVHLDDLCNAHIYLFENPKAEGRYICSSHDCIILDLAKMLRE
TRINITY_DN27402_c0_g1_i3               LSPITGTESHYHIIKQGQFVHLDDLCMAHIYLFENPKAQGRYMCSSHEATILELAKLLRE
NP_199094.1_DFR_Arabidopsis_thaliana   LSPITRNEAHYSIIRQGQYVHLDDLCNAHIFLYEQAAAKGRYICSSHDATILTISKFLRP
BAA85261.1_DFR_Arabidopsis_thaliana    LSPITRNEAHYSIIRQGQYVHLDDLCNAHIFLYEQAAAKGRYICSSHDATILTISKFLRP
AEI59122.1_DFR_Medicago_sativa         LSLITGNEAHYSIIKQGQYIHLHDLCLAHIFLFENPKAQGRYICCSHEATIHEVAKLINK
BAD67185.1_DFR_Spinacia_oleracea       LSPITRNEAHYSIIKQGQFVHLDDLCMAHIYLYEHPNAKGRYIASACDATIYDIGKMLRE
BAB40789.1_DFR_Lilium_hybrid           LSLITGNEAHYSILKQIQLVHLDDVCNAHIFLFENPEAIGRYICSSYDTTIYDLARKIKD
CAA79154.1_DFR_Solanum_lycopersicum    LSLIT...AHYGIIKQGQYVHLDDLCEAHIFLYEHPKAEGRFICSSHHAIIYDVAKMVRQ
AHZ30596.1_DFR_Prunus_domestica        LSPLTGNTSHYSIIKRGQFVHLDDLCLSHIYLYEHPKAEGRYICSSHDATIYDIAKLLRE
AAD26204.1_DFR_Malus_domestica         LSPILRNESHYGIIKQGQYVHLDDLCLSHIYLYEHPKAEGRYICSSHDATIHELVKMLRE
AAO39819.1_DFR_Pyrus_communis          LSPILRNESHYGIIKQGQYVHLDDLCLSHIYLYKHPKAEGRYICSSHDATIHELVKMLRE
AAD56578.1_DFR_Daucus_carota           LSPLTGNEAHYSIIKQCQYVHLDDLCESQIFLYEHPTAQGRYICSSHDATIHDVVKLIRE
AKN56970.1_DFR_Gerbera_hybrid_cultivar LSLITGNEAHYSIIKQGQYVHLDDLCECHIYLYENPKAKGRYICSSHDATIHQLAKIIQD
AOF39984.11_DFR_Brassica_rapa          LSPITRNEAHYSIIRQGQYVHLDDLCNAHIFLYEQAAAKGRYVCSSHDATILTISEFLRQ
P51106.1_DFR_Hordeum_vulgare           LALITGNEAHYSILKQVQLVHLDDLCDAMTFLFEHPEANGRYICSSHDATIHGLARMLQD
BAE19953.1_DFR_Lotus_japonicus         LSLITGNEAHYSIIKQGQYVHLDDLCLAHIFLFENPKAQGRYMCSAYEATIHEVARMINK
AII26023.1_DFR_Pisum_sativum           LSLITGNEAHYTIIKQGQYVHLDDLCLAHIFLFENPKAQGRYICCSHEATIHEVATLIKQ
NP_001274988.1_DFR_Solanum_tuberosum   LSLITGNEAHYGIIKQGQYVHLDDLCEAHIFLYEHPKAEGRFICSSHHAIIYDVAKMVRQ
NP_001152467.2_DFR_Zea_mays            LALITGNAPHYSILKQVQLIHLDDLCDAEIFLFENPAAAGRYVCSSHDVTIHGLAAMLRD
AIZ74402.1_DFR_Anthurium_andraeanum    LALITRNEPHYSILNPVQFVHLDDLCNAHIFLFECPDAKGRYICSSHDVTIAGLAQILRQ
AAB62873.1_DFR_Bromheadia_finlaysoniana LSLISGNEAHYSILKQAQFVHLDDLCDAHIFVYEHPEANGRYICSSHDSTIYDLANMLKN
CAA91922.1_DFR_Callistephus_chinensis  LSLINGAESHYSIIKQGQYVHLDDLCECHIFLYENPEAKGRYICSKQDATIHQLARMIKQ
AIM58715.1_DFR_Cymbidium_hybrid_cultivar LSLITGNEAHYSILRQAQFVHLDDLCDAHIFLFEHHKANGRYICSSHDSTIYSLAKMLKN
ACZ48698.1_DFR_Fagopyrum_esculentum    LSPITRTEGHYTIIKQCQYVHLDDLCMSHIYLYEKAGSKGRYVCSSDNATIYDLGKMLRN
ACZ48697.1_DFR_Fagopyrum_tataricum     LSPITRTEGHYTIIKQCQYVHLDDLCMSHIYLYEKAGSKGRYVCSSHNATIYDLGKMLRN
BAO53730.1_DFR2_Glycine_max            LSLITGNESHYHIIKQGQFVHLDDLCLGHIFVFENPKAEGRYICCSHEATIHDIAKLLNQ
AAP13055.1_DFR_Gypsophila_elegans      LSPITRTESHYTIIKQGQFVHLDDLCMAHIFLYENPKAQGRYIASACDATIYDIAKMLRE
XP_013466134.1_DFR_Medicago_truncatula LSPITGNEAHYSIIKQGQFVHLDDLCEAHIFLFEHMEVEGRYLCSACEANIHDIAKLINT
BAF96936.1_DFR_Nicotiana_tabacum       LSLITGNEAHYCIIKQGQYVHLDDLCEAHIFLYEQPKAEGRFICASHHAIIYDVAKMVRE
BAA36183.1_DFR_Oryza_sativa_Japonica_Group LALLTGNEAHYSILKQVQFVHLDDLCDAEIFLFESPEARGRYVCSSHDATIHGLATMLAD
BAA12723.1_DFR_Rosa_hybrid_cultivar    LSPLTGNESHYSIIKQGQFIHLDDLCQSHIYLYEHPKAEGRYICSSHDATIHEIAKLLKG
AHL46438.1_DFR_Fragaria_vesca          LSPLTGNEAHYGIIKQCQYVHLDDLCQSHIFLYEHPKAEGRYICSSHDATIHDIAKLLNE
AHL46443.1_DFR_Fragaria_x_ananassa     LSPLTGNEAHYGIIKQCQYVHLDDLCQSHIFLYEHPKAEGRYICSSHDATIHDIAKLLNE
AHL46445.1_DFR_Fragaria_vesca          LLPLFGNEGHYSIIKQGQFVHLDDLCQSHIYLYEHPKAEGRYICSSHDATIHEIGKLLRE
AHM27144.1_DFR_Angelonia_angustifolia  LSPITGNEAHYTIMKQGQFVHLDDICEAHIFLFEHPKADGRYICSSHDATIYDIARLIRD
AAT84073.1_DFR_Camellia_sinensis       LSPITRNEGHYSIIKQGQFVHLDDLCESHIFLYERPQAEGRYICSSHDATIHDLAKLMRE
AAS00611.1_DFR_Citrus_sinensis         LSPITRNEAHYPIIKQGQFVHLDDLCSAHIFLFEHPNAKGRYICSSHPATILELAKFLRE
AAX16491.1_DFR_Crataegus_monogyna      LSPILRNESHYGIIKQGQYVHLDDLCLSHIYLYKHPKAEGRYICSSHDATIHELVKMLRE
BAF49325.1_DFR_Delphinium_belladonna   LSPITGNQSHYSILKQIQLVHLDDLCNAHIYLFEHPESNGRYICSSHDATITDVANLLRH
CAA78930.1_DFR_Gerbera_hybrid_cultivar LSLITGNEAHYSIIKQGQYVHLDDLCECHIYLYENPKAKGRYICSSHDATIHQLAKIIKD
AAO39816.1_DFR_Malus_domestica         LSPILRNESHYGIIKQGQYVHLDDLCLSHIYLYEHPKAEGRYICSSHDATIHELVKMLRE
AAO39817.1_DFR_Malus_domestica         LSPILRNESHYGIIKQGQYVHLDDLCLSHIYLYEHPKAEGRYICSSHDATIHELVKMLRE
AAR27015.1_DFR2_Medicago_truncatula    LSLITGYEAHYSIIKQGQYIHLDDLCLAHIFLFENPKAHGRYICCSHEATIHEVAKLINK
AEF14420.1_DFR_Onobrychis_viciifolia   LSLITGNESHYHIIKQGQYVHLDDLCLGHIFLFEHPKPEGRYICCSDEATIHDVAKLINK
AAF60298.1_DFR_Petunia_x_hybrida       LSLITGNEAHYCIIKQGQYVHLDDLCEAHIFLYEHPKADGRFICSSHHAIIYDVAKMVRE
AAQ77347.1_DFR_Triticum_aestivum       LALITGNEAHYSILKQVQLVHLDDLCDAMTFLFEHPEANGRYICSSHDATIHGLARMLRD
AAL89715.1_DFR_Vaccinium_macrocarpon   LSPITGNDPHYSIIKQGQFVHLDDLCESHIYLFEHPEAEGRYICSSHDATIYDLAKIMRR
AAX12420.1_DFR_Vaccinium_macrocarpon   LSPITGNEPHYSIIKQGQFVHLDDLCESHIYLFEHPEAEGRYICSSHDATIYDLAKIMRE
CAA33543.1_DFR_Antirrhinum_majus       LSPITGNEAHYSIIKQCQYVHLDDLCEGHIFLFEYPKAEGRYICSSHDATIYDIAKLITE
AAB62873.1_DFR_Bromheadia_finlaysoniana LSLISGNEAHYSILKQAQFVHLDDLCDAHIFVYEHPEANGRYICSSHDSTIYDLANMLKN
BAA84940.1_DFR_Camellia_sinensis       LSPITRNEGHYSIIKQGQFVHLDDLCESHIFLYERPQAEGRYICSSHDATIHDLAKLMRE
AAC17843.1_DFR_Cymbidium_hybrid_cultivar LSLITGNEAHYSILRQAQFVHLDDLCDAHIFLFEHHKANGRYICSSHDSTIYSLAKMLKN
CAA91924.1_DFR_Dianthus_caryophyllus   LSPITRTESHYTIIKQGQFVHLDDLCMSHIFLYENPKANGRYIASACAATIYDIAKMLRE
CAA70345.1_DFR_Forsythia_x_intermedia  LSPITGNEAHYSIIDQCQYVHLDDLCEAHIFLYEYPNAEGRYICSSHDATIYDVAKMIRD
AAC25960.1_DFR_Fragaria_x_ananassa     LSPLTGNEAHYGIIKQCQYVHLDDLCQSHIFLYEHAKAEGRYICSSHDATIHDIAKLLNE
BAA12736.1_DFR_Gentiana_triflora       LSLITGNEAHYGIIKQGQFVHLDDLCEAHIFLYEHPEAEGRYICSSHDTTIHDLAKMIRQ
AAD54273.1_DFR1_Glycine_max            LSPITGNEDHYSIIKQGQFVHLDDLCLAHIFLFEEPEVEGRYICSACDATIHDIAKLINQ
BAA59332.1_DFR_Ipomoea_nil             LSPIMGNGLHHNIIKQGKFVHLDDLCEAQIFLYQHPKAGGRFICSSHHATIHDVAKMIRH
BAA36405.1_DFR_Ipomoea_purpurea        LSPIMGNGLHHNIIKQGKFVHLDDLCEAQIFLYQHPEAGGRFICSSHHATIHDVAKMIRH
AAD49343.1_DFR_Lilium_hybrid_division_VII LSLITGNEAHYSILKQIQLVHLDDVCKAHIFLFENPEASGRYICSSYDATIYDLARKIKD
AAF23884.2_DFR3_Lotus_corniculatus     LSPITGNEAHYSIIKQGQYVHLDDLCLAHIFLFEHPESEGRYICSASEATIHDIAKLINS
BAE19948.1_DFR_Lotus_japonicus         LCPITGNEAHYMIMKQSQFVHVDDLCLAHIFLFEHPESEGRYMCSACDANIHDIAKLINT
BAE19949.1_DFR_Lotus_japonicus         LSPITGNEAHYSIIKQGQYVHLDDLCLAHIFLFEHPESEGRYICSASEATIHDIAKLINS
BAE19950.1_DFR_Lotus_japonicus         LSPITGNEAHYSIIKQGQYVHLDDLCLAHIFLFEHPKSEGRYICSASEATIHDIAKLINS
BAE19951.1_DFR_Lotus_japonicus         LSPITGNEAHYSIIKQGQFVHLDDLCLAHIFLFEHPESEGRYILSASEATIHDIAKLINS
BAE19953.1_DFR_Lotus_japonicus         LSLITGNEAHYSIIKQGQYVHLDDLCLAHIFLFENPKAQGRYMCSAYEATIHEVARMINK
BAA19658.1_DFR_Perilla_frutescens      LSPITGNEAHYSIIKQGQFVHVDDLCEAHIFLFEHPAAEGRYICSSHDATIYDIAKMIGE
BAB20075.1_DFR_Torenia_hybrid_cultivar LSPITGNEAHYSIIKQGQFVHVDDLCEAHIFLSEHPKTEERYICSSHDATIYDIANMIRE
AAL35830.1_DFR_Triticum_monococcum     LALITGNEAHYSILKQVQLVHLDDLCDAMTFLFEHPEANGRYICSSHDATIHGLATMLRD
QFQ61498.1_DFR_Dryopteris_erythrosora  LALITGELFKLPFLARMSYIHTDDLVRAFIFLYENPEASGRYICSAVDASITEVVNIISS
QFQ61499.1_DFR_Dryopteris_erythrosora  ISLVTGDISHHKFIRRISYVHIDDIIDAHIFLYETPAAQGRYNGSCADASITEIAELLKK
BAA74700.1_DFR_Ipomoea_purpurea        LSLITGNQAHYSIIKQGQYVHLDDLCEAHIFLYEHPKAEGRFICSSHHTTIHGLAEMITQ
BAF93896.1_DFR_Iris_x_hollandica       LSFMTGNEAHYHIIKHAQLVHLDDLCAAHIYLLNRPEANGRYICSSHEATIHDLARMVRE
XP_008797532.1_DFR_Phoenix_dactylifera LSLITGNEAHYSILKQIQFVHLDDLCVAHIFLFEHPEANGRYICSSHDVTIYELARMMKE
AEQ92209.1_DFR_Ipomoea_batatas         LSLITGNQAHYSIIKQGQYVHLDDLCEAHIFLYEHPKAEGRFICSSHHTTIHGLGEMIRQ
BAH98155.1_DFR_Tulipa_gesneriana       LSLITGNDSHYSILKQIQLVHLDDLCIAHIFLFENQEASGRYICSSFDATIWDLARLMKD
APG32494.1_DFR2_Freesia_hybrid_cultivar LSLITGNEGHYSIIKQAQLVHLDDLCAAHIYLLNHPEAKGRYICSSHDATIYDLARMLRE
AAO63026.1_DFR_Allium_cepa             LSLITGNEAHYSIIKQAQLVHLDDLCEAHILLLNHPKAEGRYICSSHDVTIYDMAKMIRQ
AFP58815.1_DFR_Hyacinthus_orientalis   LSLITGNEAHYSIIKQVQLVHLDDLCEAHVFLLDHPKANGRYICSSHDATIHDLAGMIRR
                                  acc
```

```
                                                    β12    α14                    α15
NP_001268144.1_DFR_Vitis_vinifera       TT    TT
                                    270         280         290         300         310  ★      320
NP_001268144.1_DFR_Vitis_vinifera             KYPEY.NIPTEFKGVD.ENLKSVCFSSKKLTDLGF..EFKY.SLEDMFTGAVDTCRA.KG
TRINITY_DN27402_c0_g1_i3                      KFPEN.NIPSKFKDVD.ENLQNIVFSSKKLTDMGF..EFKY.SLEDMFSAAVETCRA.KG
NP_199094.1_DFR_Arabidopsis_thaliana          KYPEY.NVPSTFEGVD.ENLKSIEFSSKKLTDMGF..NFKY.SLEEMFIESIETCRQ.KG
BAA85261.1_DFR_Arabidopsis_thaliana           KYPEY.NVPSTFEGVD.ENLKSIEFSSKKLTEMGF..NFKY.SLEEMFIESIETCRQ.KG
AEI59122.1_DFR_Medicago_sativa                KYPEF.NVPTKFKDIP.DELEIIKFSSKKITDLGF..KFEY.SLEDMFTGAIETCRE.KG
BAD67185.1_DFR_Spinacia_oleracea              EYPEY.NIPTKFKDFE.EDMEHVHFSSEKLMGLNF..KFKY.ELKDMYKGAVDTCRT.KG
BAB40789.1_DFR_Lilium_hybrid                  RYPQY.AIPQKFEGID.DQIKPVHFSSKKLIDLGF..KYQY.TFEEMFDEGIRSCIE.KN
CAA79154.1_DFR_Solanum_lycopersicum           KWPEY.YVPTEFKGID.KDLALVSFSSKKLMDIKF..QFKH.TLEDMYKGAIETCRQ.KQ
AHZ30596.1_DFR_Prunus_domestica               KYPEY.NIPTKFDNIE.ENLTNIHFSSKKLTDQGF..EFKY.NLDDMFVGAVDTCRA.KG
AAD26204.1_DFR_Malus_domestica                KYPEY.NIPTKFKGID.DNLEPVHFSSKKLREIGF..EFKY.SLEDMFVGAVDACRA.KG
AAO39819.1_DFR_Pyrus_communis                 KYPEY.NIPTKFKGID.DNLEPVHFSSKKLREIGF..EFKY.SLEDMFVGAVDACRA.KG
AAD56578.1_DFR_Daucus_carota                  KWPEY.NVPTEFKGIE.KDLPVISFSSKKLTDMGF..TFKY.NLEDMFKGAIETCRE.KG
AKN56970.1_DFR_Gerbera_hybrid_cultivar        KWPEY.YISTKFPGID.EELPIVSFSSKKLIDTGF..EFKY.NLEDMFKGAIDTCRE.KG
AOF39984.11_DFR_Brassica_rapa                 KYPEY.NVPSTFEGVD.ENLKSIMFSSKKPIDMGF..NFKY.SLEDMLVESIETCRQ.KG
P51106.1_DFR_Hordeum_vulgare                  RFPEY.DIPQKFAGVD.DNLQPIHFSSKKLLDHGF..SFRY.TTEDMFDAAIHTCRD.KG
BAE19953.1_DFR_Lotus_japonicus                KYPEF.NVPTKFKDIP.DELDIIKFSSKKITDLGF..KFKY.SLEDMYTGAVETCRE.KG
AII26023.1_DFR_Pisum_sativum                  KYPEF.NVPTKFKDIP.DELEIIKFCSKKITDLGF..KFKY.SLEDMFTGAVETCRE.KG
NP_001274988.1_DFR_Solanum_tuberosum          KWPEY.YVPTEFKGID.KDLPIVSFSSKKLMDMGF..QFKY.TLEDMYKGAIETCRQ.KQ
NP_001152467.2_DFR_Zea_mays                   RYPEY.DVPQRFPGIQ.DDLQPVRFSSKKLQDLGF..TFRYKTLEDMFDAAIRTCQE.KG
AIZ74402.1_DFR_Anthurium_andraeanum           RYPEF.DVPTEFGDM..EVFDIISYSSKKLTDLGF..EFKY.SLEDMFDGAIQSCRE.KG
AAB62873.1_DFR_Bromheadia_finlaysoniana       RYATY.AIPQKFKEID.PNIKSVSFSSKKLMDLGF..KYKY.TIEEMFDDAIKTCRD.KN
CAA91922.1_DFR_Callistephus_chinensis         KWPEY.HVPTQFAGID.EELPTVSFSSKKLIDMGF..KFKY.DLEDMFKGAIDSCKE.KG
AIM58715.1_DFR_Cymbidium_hybrid_cultivar      RYATY.DIPLKFKEID.PNIESVSFSSKKLLDLGFKYKYKY.TMEEMFDDAIKTCRD.KN
ACZ48698.1_DFR_Fagopyrum_esculentum           KYPEY.NVPTKFRDFD.ENMEAVSFSSKKLTDEGF..EFKY.SLEDMFVGAVETCRE.KG
ACZ48697.1_DFR_Fagopyrum_tataricum            KYPEY.NVPTKFRDFD.ENMEAVSFSSKKLTDEGF..EFKY.SLEDMFVGAVETCRE.KG
BAO53730.1_DFR2_Glycine_max                   KYPEY.NVLTKFKNIP.DELDIIKFSSKKITDLGF..KFKY.SLEDMFTGAVETCRE.KG
AAP13055.1_DFR_Gypsophila_elegans             EYPEY.NVPTKFKDYK.EDMDLVHFSSKKLTELGF..EFKY.GLKDMYTGAVETCRA.KG
XP_013466134.1_DFR_Medicago_truncatula        KYPEY.NIPTKFNNIP.DELELVRFSSKKIKDLGF..EFKY.SLEDMYTEAIDTCIE.KG
BAF96936.1_DFR_Nicotiana_tabacum              KWPEY.YVPTEFKGID.KDLPVVYFSPKKLTDMGF..QFKY.TLEDMYKGAIETCRQ.KQ
BAA36183.1_DFR_Oryza_sativa_Japonica_Group    MFPEY.DVPRSFPGIDADHLQPVHFSSWKLLAHGF..RFRY.TLEDMFEAAVRTCRE.KG
BAA12723.1_DFR_Rosa_hybrid_cultivar           KYPEY.NVPTTFKGIE.ENLPKVHFSSKKLLETGF..EFKY.SLEDMFVGAVDACKE.KG
AHL46438.1_DFR_Fragaria_vesca                 RYPKY.NVPKKFKGIE.ENLTNIHFSSKKLKEMGF..EFKH.SLEDMFTGAVDACRA.KG
AHL46443.1_DFR_Fragaria_x_ananassa            KYPKY.NVPKKFKGIE.ENLTNIHFSSKKLKEMGF..EFKH.SLEDMFTGAVDACRE.KG
AHL46445.1_DFR_Fragaria_vesca                 KYPEY.NVPTKFKGIE.DNLTKVHFSSKKLLETGF..EFKY.SLEDMFVGAVDACKA.KG
AHM27144.1_DFR_Angelonia_angustifolia         KWPEY.NVPMEFKGID.KDIPVVRFSSKKLTSMGF..KFKY.SLEDMYRGAIETCRE.KG
AAT84073.1_DFR_Camellia_sinensis              KWPEY.NVPTEFKGID.KDLPVVSFSSKKLIGMGF..EFKY.SLEDMFRGAIDTCRE.KG
AAS00611.1_DFR_Citrus_sinensis                KYPEF.NVPTEFEDVD.ENMKNMLFSSKKLTDLGF..KFKY.SLDDMFTGAVDTCRA.KG
AAX16491.1_DFR_Crataegus_monogyna             KYPEY.NIPTKFKGID.DNLEPVHFSSKKLREIGF..EFKY.SLEDMFVGAVDACRA.KG
BAF49325.1_DFR_Delphinium_belladonna          KFPEY.NVPTKFMGVD.ENLKAVVFSSKRLKDLGF..NYKY.TMEDMFVGAVTTCRR.NG
CAA78930.1_DFR_Gerbera_hybrid_cultivar        KWPEY.YIPTKFPGID.EELPIVSFSSKKLIDTGF..EFKY.NLEDMFKGAIDTCRE.KG
AAO39816.1_DFR_Malus_domestica                KYPEY.NIPTKFKGID.DNLEPVHFSSKKLREIGF..EFKY.SLEDMFVGAVDACRA.KG
AAO39817.1_DFR_Malus_domestica                KYPEY.NIPTKFKGID.DNLEPVHFSSKKLREIGF..EFKY.SLEDMFVGAVDACRA.KG
AAR27015.1_DFR2_Medicago_truncatula           KYPEF.NVPTKFKDIP.DDLEIIKFSSKKITDLGF..IFKY.SLEDMFTGAIETCRE.KG
AEF14420.1_DFR_Onobrychis_viciifolia          IYPEF.NVPTKFKDIP.DELEIIKFSSKKITDLGF..KFKY.TLEDMLTGAVETCRE.KG
AAF60298.1_DFR_Petunia_x_hybrida              KWPEY.YVPTEFKGID.KDLPVVSFSSKKLTDMGF..QFKY.TLEDMYKGAIDTCRQ.KQ
AAQ77347.1_DFR_Triticum_aestivum              RFPEY.SIPHKFAGVG.DDLQPIHFSSKKLLDHGF..SFRY.TAEDMFDAAIRTCRE.KG
AAL89715.1_DFR_Vaccinium_macrocarpon          NAPST.MSPTEFKGIP.KDLPNVSFPSKKLIGMGF..QFKY.SLEDMFRGAIDTCQETKG
AAX12420.1_DFR_Vaccinium_macrocarpon          KWPEY.NVPTEFKGIP.KDLPNVSFSSKKLIGMGF..QFKY.SLEDMFRGAIDTCRE.KG
CAA33543.1_DFR_Antirrhinum_majus              NWPEY.HIPDEFEGID.KDIPVVSFSSKKMIGMGF..IFKY.TLEDMVRGAIDTCRE.KG
AAB62873.1_DFR_Bromheadia_finlaysoniana       RYATY.AIPQKFKEID.PNIKSVSFSSKKLMDLGF..KYKY.TIEEMFDDAIKTCRD.KN
BAA84940.1_DFR_Camellia_sinensis              KWPEY.NVPTEFKGID.KDLPVVSFSSKKLIGMGF..EFKY.SLEDMFRGAIDTCRE.KG
AAC17843.1_DFR_Cymbidium_hybrid_cultivar      RYATY.DIPLKFKEID.PNIESVSFSSKKLLDLGFKYKYKY.TMEEMFDDAIKTCRD.KN
CAA91924.1_DFR_Dianthus_caryophyllus          EYPEY.NVPTKFKDYK.EDMGQVQFSSKKLTDLGF..EFKY.GLKDMYTAAVESCRA.KG
CAA70345.1_DFR_Forsythia_x_intermedia         KWPKY.DIPTEFEGIG.KDIPVVSFSSKKLIGMGF..TFKY.TLEDMYRGAIETCRE.KG
AAC25960.1_DFR_Fragaria_x_ananassa            KYPKY.NVPKKFKGIE.ENLTNIHFSSKKLKEMGF..EFKH.SLEDMFTGAVDACRE.KG
BAA12736.1_DFR_Gentiana_triflora              NWPEY.YIPTKLKGID.EDIPVVSFSSNKLIDLGF..QYKY.TLEDMFRGAIDTCKE.KR
AAD54273.1_DFR1_Glycine_max                   KYPEY.KVPTKFKNIP.DQLELVRFSSKKITDLGF..KFKY.SLEDMYTGAIDTCRD.KG
BAA59332.1_DFR_Ipomoea_nil                    NWPEY.YVPSEFKGIE.KELPIVSFSSKKLQEMGF..QFKY.TLEDMYKGAIETLRK.KG
BAA36405.1_DFR_Ipomoea_purpurea               NWPEY.YVPSEFKGIE.KELPIVSFSSKKLQEMGF..QFKY.TLEDMYKGAIETLRK.KG
AAD49343.1_DFR_Lilium_hybrid_division_VII     RYPKY.AIPQKFEGID.DQIKPVHFSSKKLMDLGF..KYQY.TFEEMFDEGIRSCIE.KK
AAF23884.2_DFR3_Lotus_corniculatus            KYPEY.NIPTKFKNIP.DELELVRFSSKKIKDMGF..EFKY.SLEDMYTGAIDTCKE.KG
BAE19948.1_DFR_Lotus_japonicus                KYPEY.NVPTKFKNIP.DELELVRFSSKKIKDMGF..QFKY.TLEDMYTGAIDACRE.KG
BAE19949.1_DFR_Lotus_japonicus                KYPEY.NIPTKFKNIP.DELELVRFSSKKIKDMGF..EFKY.SLEDMYTGAIDTCKE.KG
BAE19950.1_DFR_Lotus_japonicus                KYPEY.NVPTKFKNIP.DELELVRFSSKKIKDMGF..EFKY.SLEDMYTGAIDTCKE.KG
BAE19951.1_DFR_Lotus_japonicus                KYPEY.NVPTKI.....................F..QMNW.SLSDFHQR.....RS.K.
BAE19953.1_DFR_Lotus_japonicus                KYPEF.NVPTKFKDIP.DELDIIKFSSKKITDLGF..KFKY.SLEDMYTGAVETCRE.KG
BAA19658.1_DFR_Perilla_frutescens             NWPEY.HVPTEFEGIH.KDIAVVSFSSKKLVDMGF..SFKY.TLEDMYRGAIESCRE.KG
BAB20075.1_DFR_Torenia_hybrid_cultivar        KWPEY.DVPTEFEGID.KDIPVVRFSSKKLMGMGF..TFKY.TLEDMFREAIETCRD.KG
AAL35830.1_DFR_Triticum_monococcum            RFPEY.SIPHKFPGVD.DDLQPIHFSSKKLLDHGF..SFRY.TAEDMFDAAIRTCRE.KG
QFQ61498.1_DFR_Dryopteris_erythrosora         RYPEF.KIPTDLAYL..GEPVIHYFDSSKLKRLGF..QFNF.TLEDMYHDAIKCCKE.KG
QFQ61499.1_DFR_Dryopteris_erythrosora         LFPKI.KLPDNFDYV..GEILLHYMDNSKLKKLGF..EFKH.GLADMFESAVKCCVE.KG
BAA74700.1_DFR_Ipomoea_purpurea               NWPEY.YIPSQFKGIE.KELAVVYFSSKKLQDMGF..QFKY.SLEDMYRGAIETLRK.KG
BAF93896.1_DFR_Iris_x_hollandica              RHPWCGSIPEKFDGIE.KDVRTVHFSSKRLLDLGF..EFKY.TVEEMFDEAIRSCVE.KK
XP_008797532.1_DFR_Phoenix_dactylifera        RYPQY.SIPQKFEGID.DGIGRVCFSSKKLIDLGF..KFRY.SMEDMFDAAIQICRE.KK
AEQ92209.1_DFR_Ipomoea_batatas                NWPEY.YIPSQFKGIE.KDLPVVYFSSKKLQDMGF..QFKY.SLEDMYRGAIETLRK.KG
BAH98155.1_DFR_Tulipa_gesneriana              RYPQY.AIPQEFEDID.EKIKPVRFSSKKLMDLGF..NYQY.TIEEMFDEGIRSCTE.KK
APG32494.1_DFR2_Freesia_hybrid_cultivar       RHPWC.SIPQKFKGIN.EGIETVHFSSKKLMDLGF..RFKY.SMEEMFDEAIQTCIE.KK
AAO63026.1_DFR_Allium_cepa                    NYPQY.YIPQQFEGID.KGIQPVRFSSKKLVDLGF..RYKY.SMESMFDEAIKTCVE.RK
AFP58815.1_DFR_Hyacinthus_orientalis          KYHQY.HIPQKIEGMD.DEIQTVRFSSKKLLEFGF..EYKY.TMEEMFDEAIKSCVE.KK
                                         acc
```

NP_001268144.1_DFR_Vitis_vinifera

```
                                                                     330
                                                                       .
NP_001268144.1_DFR_Vitis_vinifera            L..L......PPS.HE........KP..................................
TRINITY_DN27402_c0_g1_i3                     L..L......PFS...............................................
NP_199094.1_DFR_Arabidopsis_thaliana         F..L......PVSLSY........QSISEIK...............TKNENIDV......
BAA85261.1_DFR_Arabidopsis_thaliana          F..L......PVSLSY........QSISEIKVP.............TKNEIIEV......
AEI59122.1_DFR_Medicago_sativa               L..L......PKV...............................................
BAD67185.1_DFR_Spinacia_oleracea             L..L......PPS...............................................
BAB40789.1_DFR_Lilium_hybrid                 L..I......PRQ.TQ........ERYYADDKLNLC........................
CAA79154.1_DFR_Solanum_lycopersicum          L..L......PFS.TR........STAD................................
AHZ30596.1_DFR_Prunus_domestica              L..I......PIS.AE........K...................................
AAD26204.1_DFR_Malus_domestica               LIPI......PIP.AE........K...................................
AAO39819.1_DFR_Pyrus_communis                L..I......PIP.AE........K...................................
AAD56578.1_DFR_Daucus_carota                 L..L......PNSTTL........QENDQEKK............................
AKN56970.1_DFR_Gerbera_hybrid_cultivar       M..L......PYS.T.............................................
AOF39984.11_DFR_Brassica_rapa                F..L......PVTLPE........HLKSEDKVP.............GSDDNKEI......
P51106.1_DFR_Hordeum_vulgare                 L..I......PLG.DV........PAPAAGGKLGA.........................
BAE19953.1_DFR_Lotus_japonicus               L..L......PKT.A.............................................
AII26023.1_DFR_Pisum_sativum                 L..L......PKA.VE............................................
NP_001274988.1_DFR_Solanum_tuberosum         L..L......PFS.TR........SSAD................................
NP_001152467.2_DFR_Zea_mays                  L..I......PLA..............TAAGGDGFAS.......................
AIZ74402.1_DFR_Anthurium_andraeanum          L..L......PPA.TK........EPSYATEQL...........................
AAB62873.1_DFR_Bromheadia_finlaysoniana      L..M......PLN.TE........ELVLAAEKYDEV........................
CAA91922.1_DFR_Callistephus_chinensis        F..L......PYS.TN........E...................................
AIM58715.1_DFR_Cymbidium_hybrid_cultivar     L..I......PLH.TE........EMVSANEKFDEV........................
ACZ48698.1_DFR_Fagopyrum_esculentum          L..L......PKT.FE........EI..................................
ACZ48697.1_DFR_Fagopyrum_tataricum           L..L......PKT.FE........EI..................................
BAO53730.1_DFR2_Glycine_max                  L..L......PKP.EE........TTVN................................
AAP13055.1_DFR_Gypsophila_elegans            L..L......PLS...............................................
XP_013466134.1_DFR_Medicago_truncatula       L..L......PKF.V.............................................
BAF96936.1_DFR_Nicotiana_tabacum             L..L......PFS.TQ........STAD................................
BAA36183.1_DFR_Oryza_sativa_Japonica_Group   L..L.....PPLP.PP........PTTAVAGGDGSAG.......................
BAA12723.1_DFR_Rosa_hybrid_cultivar          L..L......PPP.TE........R...................................
AHL46438.1_DFR_Fragaria_vesca                L..L......PLP.QE........E...................................
AHL46443.1_DFR_Fragaria_x_ananassa           L..L......PLP.QE........EE..................................
AHL46445.1_DFR_Fragaria_vesca                L..L......PPP.TE........R...................................
AHM27144.1_DFR_Angelonia_angustifolia        L..L......PYS.TH........NQSNGEKKESTLQSLEK.....HSDDQEKVL.....
AAT84073.1_DFR_Camellia_sinensis             L..L......PHSFAE........NPVNGNKV............................
AAS00611.1_DFR_Citrus_sinensis               L..L......PLL.CE........N...................................
AAX16491.1_DFR_Crataegus_monogyna            L..I......PIP.AE........K...................................
BAF49325.1_DFR_Delphinium_belladonna         I..L......PFT...............................................
CAA78930.1_DFR_Gerbera_hybrid_cultivar       L..L......PYS.T.............................................
AAO39816.1_DFR_Malus_domestica               LIPI......PIP.AE........K...................................
AAO39817.1_DFR_Malus_domestica               LIPI......PIP.AE........K...................................
AAR27015.1_DFR2_Medicago_truncatula          L..L......PKV...............................................
AEF14420.1_DFR_Onobrychis_viciifolia         L..L......PKA.VE............................................
AAF60298.1_DFR_Petunia_x_hybrida             L..L......PFS.PR........SAED................................
AAQ77347.1_DFR_Triticum_aestivum             L..I......PLG.DA........PAPAAAGKLGA.........................
AAL89715.1_DFR_Vaccinium_macrocarpon         L..L......PYS.NE........TTANGNGNG...........................
AAX12420.1_DFR_Vaccinium_macrocarpon         L..L......PYS.NE........TTANGNGNG...........................
CAA33543.1_DFR_Antirrhinum_majus             M..L......PYS.TK........NNKGDEKEP.ILNSLEN....NYNIQDKELF.....
AAB62873.1_DFR_Bromheadia_finlaysoniana      L..M......PLN.TE........ELVLAAEKYDEV........................
BAA84940.1_DFR_Camellia_sinensis             L..L......PHSFAE........NPVNGNKV............................
AAC17843.1_DFR_Cymbidium_hybrid_cultivar     L..I......PLH.TE........EMVSANEKFDEV........................
CAA91924.1_DFR_Dianthus_caryophyllus         L..L......PLS...............................................
CAA70345.1_DFR_Forsythia_x_intermedia        L..L......PYS.TR........NQANREKKE.LLLNLKK....FHGHDQE........
AAC25960.1_DFR_Fragaria_x_ananassa           L..L......PLP.QE........EE..................................
BAA12736.1_DFR_Gentiana_triflora             M..L......PLSIGH........Q...................................
AAD54273.1_DFR1_Glycine_max                  L..L......PKP.AE............................................
BAA59332.1_DFR_Ipomoea_nil                   L..L......PYS.TK........EPADIEQEQHSG........................
BAA36405.1_DFR_Ipomoea_purpurea              L..L......PYS.TK........EPAGIEQ.............................
AAD49343.1_DFR_Lilium_hybrid_division_VII    L..I......PHQ.TQ........ERYYVHDELDLG........................
AAF23884.2_DFR3_Lotus_corniculatus           L..L......PKA.A.............................................
BAE19948.1_DFR_Lotus_japonicus               L..L......PKA.A.............................................
BAE19949.1_DFR_Lotus_japonicus               L..L......PKA.A.............................................
BAE19950.1_DFR_Lotus_japonicus               L..L......PKA.A.............................................
BAE19951.1_DFR_Lotus_japonicus               ............................................................
BAE19953.1_DFR_Lotus_japonicus               L..L......PKT.A.............................................
BAA19658.1_DFR_Perilla_frutescens            M..L......PF.STQ........IHTNGENKESLSNSQEKHSQINTNGENKDSI.....
BAB20075.1_DFR_Torenia_hybrid_cultivar       L..L......PYSSTR........DHIHGEHKIE..........................
AAL35830.1_DFR_Triticum_monococcum           L..I......PLG.DA........PPPAAAGKLGA.........................
QFQ61498.1_DFR_Dryopteris_erythrosora        L..L........................................................
QFQ61499.1_DFR_Dryopteris_erythrosora        L..LQYPGEAP.................................................
BAA74700.1_DFR_Ipomoea_purpurea              L..L......PYS.TK........EAAAIEEEQETVP.......................
BAF93896.1_DFR_Iris_x_hollandica             L..I......P...............LPENGNVDAAA.......................
XP_008797532.1_DFR_Phoenix_dactylifera       L..I......PHQ.T..........KEPLCSN............................
AEQ92209.1_DFR_Ipomoea_batatas               L..L......PYS.TK........EPAAIEEEQETVP.......................
BAH98155.1_DFR_Tulipa_gesneriana             L..L......PIH.TQ........ELFYIDDKIDLGGRKKNLINEMMRGQSEQLSMYTVE
APG32494.1_DFR2_Freesia_hybrid_cultivar      L..I......T...............LPQSGD............................
AAO63026.1_DFR_Allium_cepa                   F..I......PLQ.TAVELQLKPYELLEHNNK...............NG...........
AFP58815.1_DFR_Hyacinthus_orientalis         L..I......PPQ.AV........EALAENGK............................
                                        acc
```

NP_001268144.1_DFR_Vitis_vinifera

```
NP_001268144.1_DFR_Vitis_vinifera            .....................................................VDGKT..
TRINITY_DN27402_c0_g1_i3                     .....................................................HQPSVSS
NP_199094.1_DFR_Arabidopsis_thaliana         ...............................KTGD...GLTDGM.......KPCNKTETG
BAA85261.1_DFR_Arabidopsis_thaliana          ...............................KTGD...GLTDGM.......KPCNKTETG
AEI59122.1_DFR_Medicago_sativa               ........................................................EETP
BAD67185.1_DFR_Spinacia_oleracea             .....................................................YGENENN
BAB40789.1_DFR_Lilium_hybrid                 .........CTK............MTNDKLDLGGMK.........LNSLDEIVRGH.NEQ
CAA79154.1_DFR_Solanum_lycopersicum          ................................NGKD.............KEAIPISTENY
AHZ30596.1_DFR_Prunus_domestica              .....................................................HE.AD..
AAD26204.1_DFR_Malus_domestica               .....................................................TEAAE..
AAO39819.1_DFR_Pyrus_communis                .....................................................TEAAE..
AAD56578.1_DFR_Daucus_carota                 ...................................................YLFSATDDH
AKN56970.1_DFR_Gerbera_hybrid_cultivar       ..............................IKNHI.....................NGNH
AOF39984.11_DFR_Brassica_rapa                ...............................KNGSA..GLTDGM.......VACKKTEPG
P51106.1_DFR_Hordeum_vulgare                 ...................................................LAAGEGQA.
BAE19953.1_DFR_Lotus_japonicus               ........................................................ETPA
AII26023.1_DFR_Pisum_sativum                 .......................................................SVETE
NP_001274988.1_DFR_Solanum_tuberosum         ................................NGKD.............KEAIPISTENY
NP_001152467.2_DFR_Zea_mays                  ...............................V...................RAPGETEAT
AIZ74402.1_DFR_Anthurium_andraeanum          .........................I..............................ATGQ
AAB62873.1_DFR_Bromheadia_finlaysoniana      .........................................................KEQ
CAA91922.1_DFR_Callistephus_chinensis        ..............................VKKGL.....................FESS
AIM58715.1_DFR_Cymbidium_hybrid_cultivar     .........................................................KEQ
ACZ48698.1_DFR_Fagopyrum_esculentum          ........................................................EKNH
ACZ48697.1_DFR_Fagopyrum_tataricum           ........................................................EKNH
BAO53730.1_DFR2_Glycine_max                  ................................NV.................LLPKPAETT
AAP13055.1_DFR_Gypsophila_elegans            .....................................................L...ENH
XP_013466134.1_DFR_Medicago_truncatula       .........................................................KST
BAF96936.1_DFR_Nicotiana_tabacum             ................................NGRD.............KETIPLSAENY
BAA36183.1_DFR_Oryza_sativa_Japonica_Group   ...............................VAGEK.........E...PILGRGTGTA.
BAA12723.1_DFR_Rosa_hybrid_cultivar          .....................................................VEKQEVD
AHL46438.1_DFR_Fragaria_vesca                ............................................................
AHL46443.1_DFR_Fragaria_x_ananassa           .....................................................TEKRRAG
AHL46445.1_DFR_Fragaria_vesca                .....................................................VEKQENG
AHM27144.1_DFR_Angelonia_angustifolia        ............................EKRVNGR.....LDKQFEEQKKELLPIREEKH
AAT84073.1_DFR_Camellia_sinensis             ............................................................
AAS00611.1_DFR_Citrus_sinensis               ............................................................
AAX16491.1_DFR_Crataegus_monogyna            .....................................................TEAAE..
BAF49325.1_DFR_Delphinium_belladonna         ...................................................NKPKSATKW
CAA78930.1_DFR_Gerbera_hybrid_cultivar       ..............................IKNHI.....................NGNH
AAO39816.1_DFR_Malus_domestica               .....................................................TEAAE..
AAO39817.1_DFR_Malus_domestica               .....................................................TEAAE..
AAR27015.1_DFR2_Medicago_truncatula          ........................................................TETP
AEF14420.1_DFR_Onobrychis_viciifolia         ........................................................ETTP
AAF60298.1_DFR_Petunia_x_hybrida             ................................NGHN.............REAIAISAQNY
AAQ77347.1_DFR_Triticum_aestivum             ...................................................LAAGKGQA.
AAL89715.1_DFR_Vaccinium_macrocarpon         ................................NG..........................
AAX12420.1_DFR_Vaccinium_macrocarpon         ................................NG..........................
CAA33543.1_DFR_Antirrhinum_majus             ........................PISEEKHINGQE.NALLSNT...QDKELLPTSEEKR
AAB62873.1_DFR_Bromheadia_finlaysoniana      .........................................................KEQ
BAA84940.1_DFR_Camellia_sinensis             ............................................................
AAC17843.1_DFR_Cymbidium_hybrid_cultivar     .........................................................KEQ
CAA91924.1_DFR_Dianthus_caryophyllus         .....................................................L...EHH
CAA70345.1_DFR_Forsythia_x_intermedia        ............................ELPANG......................KEKH
AAC25960.1_DFR_Fragaria_x_ananassa           .....................................................TEKRRAG
BAA12736.1_DFR_Gentiana_triflora             .....................................................KESTDPE
AAD54273.1_DFR1_Glycine_max                  ................................KG.................LFTKPGETP
BAA59332.1_DFR_Ipomoea_nil                   ..........................................................KE
BAA36405.1_DFR_Ipomoea_purpurea              ...........................................................E
AAD49343.1_DFR_Lilium_hybrid_division_VII    .........CSK............MTNDKLDLGGSK.........LNSMDEMVRGH.NER
AAF23884.2_DFR3_Lotus_corniculatus           .........................................................ENP
BAE19948.1_DFR_Lotus_japonicus               .........................................................ETP
BAE19949.1_DFR_Lotus_japonicus               .........................................................ENP
BAE19950.1_DFR_Lotus_japonicus               .........................................................ENP
BAE19951.1_DFR_Lotus_japonicus               ......................................................KWGSNL
BAE19953.1_DFR_Lotus_japonicus               ........................................................ETPA
BAA19658.1_DFR_Perilla_frutescens            .........................LNSQINTNGENKDSILNSH.........DKHSQIH
BAB20075.1_DFR_Torenia_hybrid_cultivar       ............................................................
AAL35830.1_DFR_Triticum_monococcum           ...................................................LAAGEGQA.
QFQ61498.1_DFR_Dryopteris_erythrosora        ............................................................
QFQ61499.1_DFR_Dryopteris_erythrosora        ............................................................
BAA74700.1_DFR_Ipomoea_purpurea              .........................LKVEEPT............AIEQKQETVPLKLEEE
BAF93896.1_DFR_Iris_x_hollandica             ........................GAKDMVHGAEEH.........A...RIAMELEPKKK
XP_008797532.1_DFR_Phoenix_dactylifera       ...............................KAGEH..............VAMDA...KR
AEQ92209.1_DFR_Ipomoea_batatas               .........................LKVQEPTKQEATTVPLKPAAIEQKQETVPLKL.EE
BAH98155.1_DFR_Tulipa_gesneriana             KMFGEQIRSCTESKLLPLQTQELFYINDKIDLGGSK.........MNSIKEMMRGQ.TEQ
APG32494.1_DFR2_Freesia_hybrid_cultivar      ..........................................................RK
AAO63026.1_DFR_Allium_cepa                   ........................VVTNTIKIVGQM.........VNTKAMITEHEENEP
AFP58815.1_DFR_Hyacinthus_orientalis         .............................LEVTGEQ.........VDVAKAAMNGE.KEE
                                       acc
```

NP_001268144.1_DFR_Vitis_vinifera

**NP_001268144.1_DFR_Vitis_vinifera**	....
**TRINITY_DN27402_c0_g1_i3**	QNS...
**NP_199094.1_DFR_Arabidopsis_thaliana**	ITGERTDAP............................MLAQQMCA...............
**BAA85261.1_DFR_Arabidopsis_thaliana**	VTGERTDAP............................MLAQQMCA...............
**AEI59122.1_DFR_Medicago_sativa**	VNDTMKK...
**BAD67185.1_DFR_Spinacia_oleracea**	ENPNTIID..
**BAB40789.1_DFR_Lilium_hybrid**	VSVALQ..
**CAA79154.1_DFR_Solanum_lycopersicum**	SSGKENAPVANCT.........GKFTNGEI..............................
**AHZ30596.1_DFR_Prunus_domestica**	DNTVVDVK.................VSG................................
**AAD26204.1_DFR_Malus_domestica**	ESNLVDVK.................VG.................................
**AAO39819.1_DFR_Pyrus_communis**	ESNLVDVK.................VGG................................
**AAD56578.1_DFR_Daucus_carota**	HNGHEKDLFHHSIDKDAI....GKEKRGETES.....LVAA...................
**AKN56970.1_DFR_Gerbera_hybrid_cultivar**	VNGVHHYIKNNG..........DDQEKGLL.......CCSKEGQ................
**AOF39984.11_DFR_Brassica_rapa**	MAGEKADSH............................MSAQQICA...............
**P51106.1_DFR_Hordeum_vulgare**	IGAET...
**BAE19953.1_DFR_Lotus_japonicus**	TNGTTQK...
**AII26023.1_DFR_Pisum_sativum**	VNDTMKK...
**NP_001274988.1_DFR_Solanum_tuberosum**	SSGKENAPVANCT.........GKFTNGEI..............................
**NP_001152467.2_DFR_Zea_mays**	IGA...
**AIZ74402.1_DFR_Anthurium_andraeanum**	DNGH..
**AAB62873.1_DFR_Bromheadia_finlaysoniana**	IAVK..
**CAA91922.1_DFR_Callistephus_chinensis**	INGNVHGQKGNQ..........KIGDEGVK.......LVN....................
**AIM58715.1_DFR_Cymbidium_hybrid_cultivar**	IAVK..
**ACZ48698.1_DFR_Fagopyrum_esculentum**	VNGNGH..
**ACZ48697.1_DFR_Fagopyrum_tataricum**	VNGNGH..
**BAO53730.1_DFR2_Glycine_max**	VNDTMRK...
**AAP13055.1_DFR_Gypsophila_elegans**	ENVYA...
**XP_013466134.1_DFR_Medicago_truncatula**	NK..
**BAF96936.1_DFR_Nicotiana_tabacum**	ASGKENSPVANGT.........GKSTNGEI..............................
**BAA36183.1_DFR_Oryza_sativa_Japonica_Group**	VGAETEALVK..
**BAA12723.1_DFR_Rosa_hybrid_cultivar**	ESSVVRVK.................VTG................................
**AHL46438.1_DFR_Fragaria_vesca**	....
**AHL46443.1_DFR_Fragaria_x_ananassa**	....
**AHL46445.1_DFR_Fragaria_vesca**	DSSVVHVE.................VTG................................
**AHM27144.1_DFR_Angelonia_angustifolia**	TNGSLEKCSDDQA.KVLLPLPEEKQTNGSLEK...HVMINIKTCE...............
**AAT84073.1_DFR_Camellia_sinensis**	....
**AAS00611.1_DFR_Citrus_sinensis**	..HVSEVS.................I..................................
**AAX16491.1_DFR_Crataegus_monogyna**	DSNLVDVK.................VGG................................
**BAF49325.1_DFR_Delphinium_belladonna**	SN..
**CAA78930.1_DFR_Gerbera_hybrid_cultivar**	VNGVHHYIKNND..........DDHEKGLL.......CCSKEGQ................
**AAO39816.1_DFR_Malus_domestica**	ESNLVDVK.................AG.................................
**AAO39817.1_DFR_Malus_domestica**	ESNLVDVK.................VG.................................
**AAR27015.1_DFR2_Medicago_truncatula**	VNDTMKK...
**AEF14420.1_DFR_Onobrychis_viciifolia**	VNGTTHK...
**AAF60298.1_DFR_Petunia_x_hybrida**	ASGKENAPVANHT.........EMLSNVEV..............................
**AAQ77347.1_DFR_Triticum_aestivum**	IGAET...
**AAL89715.1_DFR_Vaccinium_macrocarpon**	.NGN.....................GNGTI..............................
**AAX12420.1_DFR_Vaccinium_macrocarpon**	.NGN.....................GNGTI..............................
**CAA33543.1_DFR_Antirrhinum_majus**	VNGLESALLSKIQDKEVLPTSGVKHAKGQENAL.LPDIANDHTDGRI.............
**AAB62873.1_DFR_Bromheadia_finlaysoniana**	IAVK..
**BAA84940.1_DFR_Camellia_sinensis**	....
**AAC17843.1_DFR_Cymbidium_hybrid_cultivar**	IAVK..
**CAA91924.1_DFR_Dianthus_caryophyllus**	LCVFRVTLIFFK..
**CAA70345.1_DFR_Forsythia_x_intermedia**	TNG...
**AAC25960.1_DFR_Fragaria_x_ananassa**	....
**BAA12736.1_DFR_Gentiana_triflora**	VDEVVKEME............................LIQDSLD................
**AAD54273.1_DFR1_Glycine_max**	VNAMHK..
**BAA59332.1_DFR_Ipomoea_nil**	PKS...
**BAA36405.1_DFR_Ipomoea_purpurea**	PKS...
**AAD49343.1_DFR_Lilium_hybrid_division_VII**	VSVALQ..
**AAF23884.2_DFR3_Lotus_corniculatus**	SNGK..
**BAE19948.1_DFR_Lotus_japonicus**	SNGIMEK...
**BAE19949.1_DFR_Lotus_japonicus**	SNGK..
**BAE19950.1_DFR_Lotus_japonicus**	SNGITEK...
**BAE19951.1_DFR_Lotus_japonicus**	NTA...
**BAE19953.1_DFR_Lotus_japonicus**	TNGTTQK...
**BAA19658.1_DFR_Perilla_frutescens**	TNGENKESILNSQEK.....HSQIRTNGENKESIFNSLEKHDTDNNQEKELLPIPKEAHA
**BAB20075.1_DFR_Torenia_hybrid_cultivar**	....
**AAL35830.1_DFR_Triticum_monococcum**	IGAET...
**QFQ61498.1_DFR_Dryopteris_erythrosora**	....
**QFQ61499.1_DFR_Dryopteris_erythrosora**	....
**BAA74700.1_DFR_Ipomoea_purpurea**	PIAIEKKQE.......VVP..................LKA....................
**BAF93896.1_DFR_Iris_x_hollandica**	VK..
**XP_008797532.1_DFR_Phoenix_dactylifera**	VN.GLTIQKS..
**AEQ92209.1_DFR_Ipomoea_batatas**	PTAIEQKQK.......VVP..................LKA....................
**BAH98155.1_DFR_Tulipa_gesneriana**	LSTAFH..
**APG32494.1_DFR2_Freesia_hybrid_cultivar**	VENG..
**AAO63026.1_DFR_Allium_cepa**	IATH..
**AFP58815.1_DFR_Hyacinthus_orientalis**	VH....IASR..

acc

NP_001268144.1_DFR_Vitis_vinifera

**NP_001268144.1_DFR_Vitis_vinifera**	. . . .
**TRINITY_DN27402_c0_g1_i3**	. . . .
**NP_199094.1_DFR_Arabidopsis_thaliana**	. . . .
**BAA85261.1_DFR_Arabidopsis_thaliana**	. . . .
**AEI59122.1_DFR_Medicago_sativa**	. . . .
**BAD67185.1_DFR_Spinacia_oleracea**	. . . .
**BAB40789.1_DFR_Lilium_hybrid**	. . . .
**CAA79154.1_DFR_Solanum_lycopersicum**	. . . .
**AHZ30596.1_DFR_Prunus_domestica**	. . . .
**AAD26204.1_DFR_Malus_domestica**	. . . .
**AAO39819.1_DFR_Pyrus_communis**	. . . .
**AAD56578.1_DFR_Daucus_carota**	. . . .
**AKN56970.1_DFR_Gerbera_hybrid_cultivar**	. . . .
**AOF39984.11_DFR_Brassica_rapa**	. . . .
**P51106.1_DFR_Hordeum_vulgare**	. . . .
**BAE19953.1_DFR_Lotus_japonicus**	. . . .
**AII26023.1_DFR_Pisum_sativum**	. . . .
**NP_001274988.1_DFR_Solanum_tuberosum**	. . . .
**NP_001152467.2_DFR_Zea_mays**	. . . .
**AIZ74402.1_DFR_Anthurium_andraeanum**	. . . .
**AAB62873.1_DFR_Bromheadia_finlaysoniana**	. . . .
**CAA91922.1_DFR_Callistephus_chinensis**	. . . .
**AIM58715.1_DFR_Cymbidium_hybrid_cultivar**	. . . .
**ACZ48698.1_DFR_Fagopyrum_esculentum**	. . . .
**ACZ48697.1_DFR_Fagopyrum_tataricum**	. . . .
**BAO53730.1_DFR2_Glycine_max**	. . . .
**AAP13055.1_DFR_Gypsophila_elegans**	. . . .
**XP_013466134.1_DFR_Medicago_truncatula**	. . . .
**BAF96936.1_DFR_Nicotiana_tabacum**	. . . .
**BAA36183.1_DFR_Oryza_sativa_Japonica_Group**	. . . .
**BAA12723.1_DFR_Rosa_hybrid_cultivar**	. . . .
**AHL46438.1_DFR_Fragaria_vesca**	. . . .
**AHL46443.1_DFR_Fragaria_x_ananassa**	. . . .
**AHL46445.1_DFR_Fragaria_vesca**	. . . .
**AHM27144.1_DFR_Angelonia_angustifolia**	. . . .
**AAT84073.1_DFR_Camellia_sinensis**	. . . .
**AAS00611.1_DFR_Citrus_sinensis**	. . . .
**AAX16491.1_DFR_Crataegus_monogyna**	. . . .
**BAF49325.1_DFR_Delphinium_belladonna**	. . . .
**CAA78930.1_DFR_Gerbera_hybrid_cultivar**	. . . .
**AAO39816.1_DFR_Malus_domestica**	. . . .
**AAO39817.1_DFR_Malus_domestica**	. . . .
**AAR27015.1_DFR2_Medicago_truncatula**	. . . .
**AEF14420.1_DFR_Onobrychis_viciifolia**	. . . .
**AAF60298.1_DFR_Petunia_x_hybrida**	. . . .
**AAQ77347.1_DFR_Triticum_aestivum**	. . . .
**AAL89715.1_DFR_Vaccinium_macrocarpon**	. . . .
**AAX12420.1_DFR_Vaccinium_macrocarpon**	. . . .
**CAA33543.1_DFR_Antirrhinum_majus**	. . . .
**AAB62873.1_DFR_Bromheadia_finlaysoniana**	. . . .
**BAA84940.1_DFR_Camellia_sinensis**	. . . .
**AAC17843.1_DFR_Cymbidium_hybrid_cultivar**	. . . .
**CAA91924.1_DFR_Dianthus_caryophyllus**	. . . .
**CAA70345.1_DFR_Forsythia_x_intermedia**	. . . .
**AAC25960.1_DFR_Fragaria_x_ananassa**	. . . .
**BAA12736.1_DFR_Gentiana_triflora**	. . . .
**AAD54273.1_DFR1_Glycine_max**	. . . .
**BAA59332.1_DFR_Ipomoea_nil**	. . . .
**BAA36405.1_DFR_Ipomoea_purpurea**	. . . .
**AAD49343.1_DFR_Lilium_hybrid_division_VII**	. . . .
**AAF23884.2_DFR3_Lotus_corniculatus**	. . . .
**BAE19948.1_DFR_Lotus_japonicus**	. . . .
**BAE19949.1_DFR_Lotus_japonicus**	. . . .
**BAE19950.1_DFR_Lotus_japonicus**	. . . .
**BAE19951.1_DFR_Lotus_japonicus**	. . . .
**BAE19953.1_DFR_Lotus_japonicus**	. . . .
**BAA19658.1_DFR_Perilla_frutescens**	D R Q E M Q I
**BAB20075.1_DFR_Torenia_hybrid_cultivar**	. . . .
**AAL35830.1_DFR_Triticum_monococcum**	. . . .
**QFQ61498.1_DFR_Dryopteris_erythrosora**	. . . .
**QFQ61499.1_DFR_Dryopteris_erythrosora**	. . . .
**BAA74700.1_DFR_Ipomoea_purpurea**	. . . .
**BAF93896.1_DFR_Iris_x_hollandica**	. . . .
**XP_008797532.1_DFR_Phoenix_dactylifera**	. . . .
**AEQ92209.1_DFR_Ipomoea_batatas**	. . . .
**BAH98155.1_DFR_Tulipa_gesneriana**	. . . .
**APG32494.1_DFR2_Freesia_hybrid_cultivar**	. . . .
**AAO63026.1_DFR_Allium_cepa**	. . . .
**AFP58815.1_DFR_Hyacinthus_orientalis**	. . . .

acc

```
sp|Q4W2K4_LAR_Vitis_vinifera
                                                1
sp|Q4W2K4_LAR_Vitis_vinifera                  .....MTVSP............VPS..................................P
TRINITY_DN33042_c3_g1_i3                      .....MTASATL..........FST..................................M
NP_001352050.1_LAR_Glycine_max                .....MVTSPPA..........IPTTT.................................
ADD51357.1_LAR_Theobroma_cacao                MKSTNMNGSSPN..........VSE..................................E
CAI56321.1_LAR_Pinus_taeda                    .....MACATDVARQFLPCVQPVPSSMGGETARSINLTCNGLSPPQPQYNAENNHDQDTT
CAI56322.1_LAR_Phaseolus_coccineus            .....MVTS.PT..........IPSHT.................................
CAI56320.1_LAR_Hordeum_vulgare_subsp._vulgare .......MAPC..EELQ...EEVA...................................R
CAD79341.1_LAR_Desmodium_uncinatum            ......MTVSGA..........IPSMT.................................
AIS92512_LAR_Epimedium_sagittatum             .......MAPPS..........VDSVATF........................SFETCSK
AII26024.1_LAR_Pisum_sativum                  .......MAPTS..........SPPTTL..............................AS
AEF14422.1_LAR_Onobrychis_viciifolia          .....MATSPAN..........IPPTL.................................
ABE90657.1_LAR_Medicago_truncatula            .......MAPSS..........S.PTTP..............................IS
CAI56326.1_LAR_Vitis_shuttleworthii           .....MTVSP............VPS..................................L
ABC71327.1_LAR_Lotus_corniculatus             ......MVSTAA..........TPPAT.................................
AHA14498.1_LAR_Fagopyrum_tataricum            .....MTVAVTA..........IPE..................................S
AAZ82410.1_LAR_Vitis_vinifera                 .....MTVSP............VPS..................................P
ADY15310.1_LAR_Prunus_avium                   .....MTVSTCV..........SAA..................................K
BAH89267.1_LAR_Diospyros_kaki                 .....MTVSPSF.........AAAA..................................K
AAX12186.1_LAR_Malus_domestica                .....MTVSPSL..........SVA..................................R
AEY62396.1_LAR_Fagopyrum_dibotrys             .....MTVAVTA..........IPE..................................S
AAZ79364.1_LAR_Malus_domestica                .....MTVSSSL..........SVA..................................K
CAI56319.1_LAR_Gossypium_arboreum             .....MTV..............SVA...................................
CAI56323.1_LAR_Gossypium_arboreum             MKSTQMNGSYPN..........ES...................................E
CAI56324.1_LAR_Gossypium_raimondii            .....MTV..............SVA...................................
CAI56325.1_LAR_Gossypium_raimondii            MKSTHMNGSYPN..........ES...................................E
CAI56328.1_LAR_Oryza_sativa_Japonica_Group    .......MAPAAQELLL...QEVPQ................................PRR
CAI26308.1_LAR_Vitis_vinifera                 ..MTVLSVSTPP..........APQAPP..............................AA
ABF95070.1_LAR_Oryza_sativa_Japonica_Group    .......MAPAAQELLL...QEVPQ................................PRR
ADD51358.1_LAR_Theobroma_cacao                MKSTNMNGSSPN..........VSE..................................E
ACI41981.1_LAR_Diospyros_kaki                 .....MTVSPSF.........AAAA..................................K
ABC71329.1_LAR_Lotus_corniculatus             ...........................M..............................AT
ABH07785.2_LAR_Fragaria_x_ananassa            .....MTVSPSI.........ASAA..................................K
ABB77697.1_LAR_Pyrus_communis                 .....MTVSPSL..........SVA..................................I
                                          acc
```

```
sp|Q4W2K4_LAR_Vitis_vinifera                    β1        α1            β2              α2          β3
                                              10        20        30   ★    40        50        60
sp|Q4W2K4_LAR_Vitis_vinifera                  KG.RVLIAGATGFIGQFVATASLDAHRPTYILARP.GPRS.P.SKAKIFKALEDKGAIIV
TRINITY_DN33042_c3_g1_i3                      GG.SVLIAGATGFIGQYVVQASLDSGRRTYVLVRP.SATACP.SRAKFIKCLEEKGAIIL
NP_001352050.1_LAR_Glycine_max                KD.RVLIIGATGFIGKFVAEASLTSEHPTCLLVRP.GPLV.P.SKDAIVKTFQDKGAIVI
ADD51357.1_LAR_Theobroma_cacao                TG.RTLVVGSGGFMGRFVTEASLDSGRPTYILARS.S.SNSP.SKASTIKFLQDRGATVI
CAI56321.1_LAR_Pinus_taeda                    VATRVLIIGATGFIGRFVAEASVKSGRPTYALVRP.TTLS...SKPKVIQSLVDSGIQVV
CAI56322.1_LAR_Phaseolus_coccineus            KA.RVLIIGATGFIGKFVTEASLLTAHPTYLLLRP.PPLV.P.SKDAIVKTFQEKGAMII
CAI56320.1_LAR_Hordeum_vulgare_subsp._vulgare SG.PALIVGATGYIGRFVAEACLDSGRRTFILVRP.GNAC.P.ARAASVDALLRKGAFVV
CAD79341.1_LAR_Desmodium_uncinatum            KN.RTLVVGGTGFIGQFITKASLGFGYPTFLLVRP.GPVS.P.SKAVIIKTFQDKGAKVI
AIS92512_LAR_Epimedium_sagittatum             AG.RTLIIGATGFIGGFIVDACLASGRPTYILSRS......KSTKVGAHKELQDKGAIVL
AII26024.1_LAR_Pisum_sativum                  KN.RVLIIGATGFMGKFLTEASLSSSHPTYLLIRPGGPLL.S.PKSTTIKTFQDKGAIIV
AEF14422.1_LAR_Onobrychis_viciifolia          KG.RVLIVGATGFMGKFVAEASLSSAHATFLLLRP.GPII.S.SKASIVKAFQDKGARVI
ABE90657.1_LAR_Medicago_truncatula            KG.RVLIVGATGFMGKFVTEASISTAHPTYLLIRP.GPLI.S.SKAATIKTFQEKGAIVI
CAI56326.1_LAR_Vitis_shuttleworthii           KG.RVLIAGATGFIGQFVAAASLDAHRPTYILARP.GPRS.P.SKAKIIKAHEDKGAIIV
ABC71327.1_LAR_Lotus_corniculatus             AG.RILIIGATGFMGQFMTKASLGLGRSTYLLLRP.GSLT.P.SKAAIVKSFQDRGAKVI
AHA14498.1_LAR_Fagopyrum_tataricum            KC.RTLVAGATGFIGRFVTESSLESERPTFILVRP.GPIS.P.SKTKIIKALEDKGAIIV
AAZ82410.1_LAR_Vitis_vinifera                 KG.RVLIAGATGFIGQFVAAASLDAHRPTYILARP.GPRS.P.SKANIFKALEDKGAIIV
ADY15310.1_LAR_Prunus_avium                   NG.RILIVGATGFIGRFVAEASLDAGQPTYVLVRP.GPLD.P.SKADIIKALKDRGAIIL
BAH89267.1_LAR_Diospyros_kaki                 QG.RVLIAGATGFIGQFVAEASLEAGRTTYVLVRS.G....P.SKAKTIKALQEKGAIPI
AAX12186.1_LAR_Malus_domestica                NG.RVLIVGATGFIGRFVAEASLAAGRPTYVLVRP.GPLH.P.SKADTVKSFKHKGAIIL
AEY62396.1_LAR_Fagopyrum_dibotrys             KC.RTLVAGATGFIGRFVTESSLESERPTFILVRP.GPIS.P.SKTKIIKALEDKGAIIV
AAZ79364.1_LAR_Malus_domestica                NG.RVLIAGATGFIGRFVAEASLAAGQPTYVLVRP.GPLH.P.SKADTVKSFKDKGAIIL
CAI56319.1_LAR_Gossypium_arboreum             NG.RVLIVGATGFIGRFVADASLDAGRPTYVLVRP.SSGN.QYSKDKVAKALQDRGAILL
CAI56323.1_LAR_Gossypium_arboreum             AG.QTVVIGSSGFIGRFITEACLDSGRPTYILVRS.S.SNSP.SKASTIKFLQDKGAIVI
CAI56324.1_LAR_Gossypium_raimondii            NG.RVLIVGATGFIGRFVADASLDAGRPTYVLVRP.SSGN.QYSKDKVAKALRDRGAILL
CAI56325.1_LAR_Gossypium_raimondii            TG.QTLVIGSSGFIGRFITEACLDSGRPTYILVRS.S.SNSP.SKASTIKFLQDKGAIVI
CAI56328.1_LAR_Oryza_sativa_Japonica_Group    TG.AALIVGATGYIGRFVAEACLDSGRDTFILVRP.GNAC.P.ARAASVDALRQKGAVVI
CAI26308.1_LAR_Vitis_vinifera                 TGPRTLEVGASGFIGRFVAEASLSSGHPTYVLVRS.SATTSS.SKASTIKSLEDQGAILV
ABF95070.1_LAR_Oryza_sativa_Japonica_Group    TG.AALIVGATGYIGRFVAEACLDSGRDTFILVRP.GNAC.P.ARAASVDALRQKGAVVI
ADD51358.1_LAR_Theobroma_cacao                TG.RTLVVGSGGFMGRFVTEASLDSGRPTYILARS.S.SNSP.SKASTIKFLQDRGATVI
ACI41981.1_LAR_Diospyros_kaki                 QG.RVLIVGATGFIGQFVAEASLEAGRTTYVLVRS.G....P.SKAKTIKALQEKGAIPI
ABC71329.1_LAR_Lotus_corniculatus             KG.RVLIIGATGFMGRFMAEASLAAAHPTYLLVRL..PLI.P.SKATIVKTFQDKGAIVI
ABH07785.2_LAR_Fragaria_x_ananassa            SG.RVLIIGATGFIGKFVAEASLDSGLPTYVLVRP.GPSR.P.SKSDTIKSLKDRGAIIL
ABB77697.1_LAR_Pyrus_communis                 NG.RVLIVGATGFIGRFVAEASLAAGQPTYVLVRP.GPLH.P.SKADTVKSFKHKGAIIL
                                          acc
```

```
sp|Q4W2K4_LAR_Vitis_vinifera                        α3          β4       η1        α4             β5
                                                                   70         80      ★ 90          100     ★   110        120
sp|Q4W2K4_LAR_Vitis_vinifera                 Y.GLINEQE...AMEKILKEHEIDIVVSTV.GGESILDQIALVKAMKAVGTIKRFLPSEF
TRINITY_DN33042_c3_g1_i3                     L.GLINDKE...NMEKLLKEHKIDIVISAV.GGENVLDQLVLVEAINAVGTVKRYVPSEF
NP_001352050.1_LAR_Glycine_max               H.GVINNKD...FVEKILKEYEIDIVISAI.GAKSLLDQLILVEAMKSVKTIKRFLPSEF
ADD51357.1_LAR_Theobroma_cacao               Y.GSITDKE...FMEKVLKEHKIEVVISAV.GGGSILDQFNLIEAIRNVDTVKRFLPSEF
CAI56321.1_LAR_Pinus_taeda                   Y.GCLHDHN...SLVKAIR..QVDVVISTV.GGALILDQLKIVDAIKEVGTVKRFLPSEF
CAI56322.1_LAR_Phaseolus_coccineus           H.GVINNKD...FVEKILKEHEIDIVISAI.GAKSLLDQLILVEAMKSLKTIKRFLASEF
CAI56320.1_LAR_Hordeum_vulgare_subsp._vulgare E.GRVDGKDGKRSVETALRAHGIEVVISVM.GGANILDQLGLIKAIQAAGTVKRFLPSEF
CAD79341.1_LAR_Desmodium_uncinatum           Y.GVINDKE...CMEKILKEYEIDVVISLV.GGARLLDQLTLLEAIKSVKTIKRFLPSEF
AIS92512_LAR_Epimedium_sagittatum            Y.GKINDRH...TMENILKDHKIEMVISAV.GGDTLLEQIDLVHAIKAVGSVQRFLPSEF
AII26024.1_LAR_Pisum_sativum                 Y.GVVDNKE...FMEKILKKYEIDIVISAI.GAESLLDQITLVEAMKSIKTVKRFLPSEF
AEF14422.1_LAR_Onobrychis_viciifolia         Y.GVVNNKE...LMEKILKEYEIDIVISAI.GAESLMDQLTLVEAMKSVKSIKRFLPSEF
ABE90657.1_LAR_Medicago_truncatula           Y.GVVNNKE...FVEMILKKYEIDTVISAI.GAESLLDQLTLVEAMKSIKTIKRFLPSEF
CAI56326.1_LAR_Vitis_shuttleworthii          Y.GLINEQE...SMEKILKEHEIDIVVSTV.GGESILDQIALVKAMKAVGTIKRFLPSEF
ABC71327.1_LAR_Lotus_corniculatus            H.GVINDKE...LMVKILKDYEIDVVISLV.GGGNLMDQRTLVDAIKSVKTVKRFLPSEF
AHA14498.1_LAR_Fagopyrum_tataricum           Q.GLINNKE...RMEKILRENEINVVISAV.GGGNILDQIPLVHAIKSVPSVKRFLASEF
AAZ82410.1_LAR_Vitis_vinifera                Y.GLINEQE...AMEKILKEHEIDIVVSTV.GGESILDQIALVKAMKAVGTIKRFLPSEF
ADY15310.1_LAR_Prunus_avium                  Q.GVISDKA...LMEKLLREHEIEVVISAV.GGATILDQITLVEAIQAVGTIKRFLPSEF
BAH89267.1_LAR_Diospyros_kaki                PQGNINDQE...FLEKILKEYEIDVVISAV.GGDSIRDQLTLVHAIKAVGTIKRFLPSEF
AAX12186.1_LAR_Malus_domestica               H.GLISDKT...LMEKILREHEIETVISAV.GGATILDQIALVEAIAAVGTVKRFLPSEF
AEY62396.1_LAR_Fagopyrum_dibotrys            Q.GLINNKE...RMEKILRENEINVVISAV.GGGNILDQIPLVHAIKSVPSVKRFLASEF
AAZ79364.1_LAR_Malus_domestica               H.GLISDKT...LMEKMLREHEIEIVISAV.GGATILDQITLVDAIAAVGTVKRFLPSEF
CAI56319.1_LAR_Gossypium_arboreum            N.GLANDKE...LMVKLLKEHQIEIVISAL.GGATILDQLSLVEAIHSVGTVKRFLPSEF
CAI56323.1_LAR_Gossypium_arboreum            Y.GSITDQE...FMEKVLREYKIEVVISAV.GGESILDQLSLIEAIKNVNTVKRFVPSEF
CAI56324.1_LAR_Gossypium_raimondii           N.GLANDKE...LMVKLLKEHQIEIVISAL.GGATILDQLSLVEAIHSAGTVKRFLPSEF
CAI56325.1_LAR_Gossypium_raimondii           Y.GSITDQE...FMEKVLREYKIEVVISAV.GGESILDQFSLIEAIKNVNTVKRFVPSEF
CAI56328.1_LAR_Oryza_sativa_Japonica_Group   E.GCVGGKEGRKSVEAALRARGVEVVISVM.GGASILDQLGLIEAIRAAGTVKRFLPSEF
CAI26308.1_LAR_Vitis_vinifera                T.GSIGDKE...VMIEILRKYEIEVVISAV.GGATILDQLTLAEAIKAVGSIKRFLPSEF
ABF95070.1_LAR_Oryza_sativa_Japonica_Group   E.GCVGGKEGRKSVEAALRARGVEVVISVM.GGASILDQLGLIEAIRAAGTVKRFLPSEF
ADD51358.1_LAR_Theobroma_cacao               Y.GSITDKE...FMEKVLKEHKIEVVISAV.GGGSILDQFNLIEAIRNVDTVKRFLPSEF
ACI41981.1_LAR_Diospyros_kaki                P.GNINDQE...FLEKILKEYEIDVVISAV.GGDSIRDQLTLVHAIKAVGTXKRFLPSEF
ABC71329.1_LAR_Lotus_corniculatus            Q.GVMNDKE...FMQKILKEYQIDVVISTVGGAHGLLDQLTLVEAMKSVNTIKRFLPSEF
ABH07785.2_LAR_Fragaria_x_ananassa           H.GVMSDKP...LMEKLLKEHEIEIVISAV.GGATILDQITLVEAITSVGTVKRFLPSEF
ABB77697.1_LAR_Pyrus_communis                H.GLISDKT...LMEKILREHEIETVISAV.GGATILDQIALVEAIAAVGTVKRFLPSEF
                                         acc

sp|Q4W2K4_LAR_Vitis_vinifera                          α5                 β6  β7
                                                  130       140       150 ★     160    ★  170       180
sp|Q4W2K4_LAR_Vitis_vinifera                 GHDVNRADPVEPGLNMYREKRRVRQLVEESGIPFTYICCNSIASWPYYNNIHPSEVLPPT
TRINITY_DN33042_c3_g1_i3                     GHDVDRADPVEPGDKMYMEKRKVRRLIEEYGIPYTYICCNSIASWPYYTNKHPAEVLPPL
NP_001352050.1_LAR_Glycine_max               GHDVDKADPVEPGLTMYKEKRLVRRVVEESGVPFTNICCNSIASWPYHDNCHPSQLPPPL
ADD51357.1_LAR_Theobroma_cacao               GHDTDRADPVEPGLTMYEQKRQIRRQIEKSGIPYTYICCNSIAAWPYHDNTHPADVLPPL
CAI56321.1_LAR_Pinus_taeda                   GHDVDRADPVEPALSFYIEKRKVRRAVEEAKIPYTYICCNSIAGWPYYYHTHPTELPPPK
CAI56322.1_LAR_Phaseolus_coccineus           GHDVDRADPVEPGLTMYKEKQLVRRVVEQSGVPYTNICCNSIASWPYYDNCHPSQLPPPL
CAI56320.1_LAR_Hordeum_vulgare_subsp._vulgare GHDVDRARPVGAGLGFYEEKRRVRRAAEAAGVPYTYICCNSIAGWPYFDNMHPSEVRPPL
CAD79341.1_LAR_Desmodium_uncinatum           GHDVDRTDPVEPGLTMYKEKRLVRRAVEEYGIPFTNICCNSIASWPYYDNCHPSQVPPPM
AIS92512_LAR_Epimedium_sagittatum            GHDVDRADPVEPGLTMYNEKRKVRRVIEEVGIPYTYICCNSIAAWPYYDNTHPSEVLPPM
AII26024.1_LAR_Pisum_sativum                 GHDVDRADPVEPGLAMYKQKRLVRRVIEESGVPYTYICCNSIASWPYYNNCHPSSLPPPL
AEF14422.1_LAR_Onobrychis_viciifolia         GHDVDRADPVEPGLAMYKEKRLVRRVIEQSGVPYTYICCNSIASWPYYDNCHPSQLPPPL
ABE90657.1_LAR_Medicago_truncatula           GHDVDRADPVEPGLAMYKQKRLVRRVIEESGVPYTYICCNSIASWPYYDNCHPSQLPPPL
CAI56326.1_LAR_Vitis_shuttleworthii          GHDVNRADPVEPGLNMYREKRRVRQLVEESGIPFTYICCNSIASWPYYNNIHPSEVLPPT
ABC71327.1_LAR_Lotus_corniculatus            GHDTDRANPVEPGLTMYKEKRLVRRLIEESGIPYTYICCNSIASWPYHDNCHPSKVPPPV
AHA14498.1_LAR_Fagopyrum_tataricum           GHDVDRANPVEPGLTMYLEKRAVRRAIEEAGVPYTHICCNSIASWPYYDNTHPSEVTPPM
AAZ82410.1_LAR_Vitis_vinifera                GHDVNRADPVEPGLNMYREKRRVRQLVEESGIPFTYICCNSIASWPYYNNIHPSEVLPPT
ADY15310.1_LAR_Prunus_avium                  GHDVDRADPVEPGLTMYLEKRQVRRWVEKSGVPYTYICCNSIASWPYFDNKHPSEVLPPL
BAH89267.1_LAR_Diospyros_kaki                GHDVDRANPVEPGLAMYIEKRTVRRVVEESGVPYTYICCNSIASWPYYDNTHPSEVLPPL
AAX12186.1_LAR_Malus_domestica               GHDVDRADPVEPGLTMYEEKRKVRRWVEKSGVAYTYICCNSIASWPYFDNTHPSEVLPPL
AEY62396.1_LAR_Fagopyrum_dibotrys            GHDVDRANPVEPGLTMYLEKRAVRRAIEEAGVPYTHICCNSIASWPYYDNTHPSEVTPPM
AAZ79364.1_LAR_Malus_domestica               GHDVDRADPVEPGLTMYLEKRKVRRSVETSGVPYTYICCNSIASWPYFDNTHPSEVLPPL
CAI56319.1_LAR_Gossypium_arboreum            GHDVDRADPVEPGLTMYKEKRQVRRLIEKLEIPYTYICCNSIASWPYHNNTHPSEVIPPL
CAI56323.1_LAR_Gossypium_arboreum            GHDIDRAEPVEPGLTMYEQKRKIRRQIEECGIPYSYICCNSIAAWPYHDNTHPTDVLPPL
CAI56324.1_LAR_Gossypium_raimondii           GHDVDRADPVEPGLTMYKEKRQVRRLIEKLEIPYTYICCNSIASWPYHNNRHPSEVIPPL
CAI56325.1_LAR_Gossypium_raimondii           GHDIDRAEPVEPGLTMYEQKSKIRRQIEECGIPYSYICCNSIAAWPYHDNTHPADVLPPL
CAI56328.1_LAR_Oryza_sativa_Japonica_Group   GHDVDRARPVGAGLRFYEEKRLVRRAAEASGVPYTFICCNSIAGWPYHDSTHPSELPPPL
CAI26308.1_LAR_Vitis_vinifera                GHDIDRAEPVEPGLTMYMEKRKVRRFIEEAAIPYTYICCNSIAAWPYHDNTHPADVLPPL
ABF95070.1_LAR_Oryza_sativa_Japonica_Group   GHDVDRARPVGAGLRFYEEKRLVRRAAEASGVPYTFICCNSIAGWPYHDSTHPSELPPPL
ADD51358.1_LAR_Theobroma_cacao               GHDTDRADPVEPGLTMYEQKRQIRRQVEKSGIPYTYICCNSIAAWPYHDNTHPADVLPPL
ACI41981.1_LAR_Diospyros_kaki                GHDVDRANPVEPGLAMYIEKRRVRRVVEESGVPYTYICCNSIASWPYYDNTHPSEVLPPL
ABC71329.1_LAR_Lotus_corniculatus            GHDVDRADPVEPGLAMYKEKRLVRRVIEESGIPYTYICCNSIASWPYYNNCHPSQLPPPL
ABH07785.2_LAR_Fragaria_x_ananassa           GHDVDRADPVEPGLTMYLEKRKVRRAIEKSGVPYTYICCNSIASWPYYDNKHPSEVVPPL
ABB77697.1_LAR_Pyrus_communis                GHDVDRADPVEPGLTMYVEKRKVRRWVEKSGVPYTYICCNSIASWPYFDNTHPSEVLPPL
                                         acc
```

```
sp|Q4W2K4_LAR_Vitis_vinifera               β8  TT   β9    α6            η2   β10    η3 β11    α7
                                                  190        200        210        220        230        240
sp|Q4W2K4_LAR_Vitis_vinifera               DFFQIYGDGNVKAYFVAGTDIGKFTMKTVDDVRTLNKSVHFRPSCNCLNINELASVWEKK
TRINITY_DN33042_c3_g1_i3                   DEFQIYGDGNTKAYFVAGSDIGKFTMKAVDDVRTINKKVHFQPSCNFYNMNELAALWENK
NP_001352050.1_LAR_Glycine_max             DQLQIYGHGNVKAYFVDGIDIGKFTMKVIDDVRTVNKNVHFRPSNNCYSVNELASLWEKK
ADD51357.1_LAR_Theobroma_cacao             DRFKIYGDGTVKAYFVAGTDIGKFTIMSIEDDRTLNKTVHFQPPSNLLNINEMASLWEEK
CAI56321.1_LAR_Pinus_taeda                 EQFEIYGDGSVKAFFVTGDDIGAYTMKAVDDPRTLNKSIHFRPPKNFLNLNELADIWENK
CAI56322.1_LAR_Phaseolus_coccineus         DQLQIYGHGNVKAYFVDGIDIGKFTMKVIDDVKTINKNVHFRPSKNCYSINELASLGEMK
CAI56320.1_LAR_Hordeum_vulgare_subsp._vulgare DRFQIYGDGTVRAFFVAGTDIGKFTVKAAYDARSVNKAVHFRPACNLLSTNEMACLWESK
CAD79341.1_LAR_Desmodium_uncinatum         DQFQIYGDGNTKAYFIDGNDIGKFTMKTIDDIRTLNKNVHFRPSSNCYSINELASLWEKK
AIS92512_LAR_Epimedium_sagittatum          DQFLIYGDGSVKAYFVDGKDIGKFTMKALDDIRTINKSVHFRPPSNCLSINDMATLWEKK
AII26024.1_LAR_Pisum_sativum               DQLHVYGNGNVKAYFVDGFDIGKFTMKIVDDHRTINKNIHFRPSINCYSMNELASLWESK
AEF14422.1_LAR_Onobrychis_viciifolia       DQLHIYGDGRVQAYFIDGYDIGKFTMKVVDDVRTINKNVHFRPPTNCYSMNGLASLWEKK
ABE90657.1_LAR_Medicago_truncatula         DQLHIYGHGNVKAYFVDGYDIGKFTMKVVDDERTINKSVHFRPSTNCYSMNELASLWENK
CAI56326.1_LAR_Vitis_shuttleworthii        DFFQIYGDGNVKAYFVAGTDIGKFTMKTVDDVRTLNKSVHFRPSCNCLNINELASVWEKK
ABC71327.1_LAR_Lotus_corniculatus          DQFLIYGDGSVKAYFVDGNDIGKFTMKAIDDIRTRNKNVHFRPPSNCYSINELASLWEKI
AHA14498.1_LAR_Fagopyrum_tataricum         DRFQIYGDGNVKAYFVAGSDIGKFTMKTLEDPRTLNKMVHFRPPSNYLTINELASLWEKK
AAZ82410.1_LAR_Vitis_vinifera              DFFQIYGDGNVKAYFVAGTDIGKFTMKTVDDVRTLNKSVHFRPSCNCLNINELASVWEKK
ADY15310.1_LAR_Prunus_avium                DQFQIYGDGSVKAYFIDGTDIGKFTIKTVEDIRTINKNVHFRPPSNLYDINGLASLWEKK
BAH89267.1_LAR_Diospyros_kaki              DHFQIYGDGSVKAYFVAGSDIGKFTIKAADDIRTLNKSIHFRPPCNFLNINELASMWEKK
AAX12186.1_LAR_Malus_domestica             DRFQIYGDGTVKAYFVDGTDIGKFTIKTVDDIRTINKNIHFRPPSNLYDINGLASLWEKK
AEY62396.1_LAR_Fagopyrum_dibotrys          DRFQIYGDGNVKAYFVAGSDIGKFTMKTLEDPRTLNKMVHFRPPSNYLTINELASLWEKK
AAZ79364.1_LAR_Malus_domestica             DRFQIYGDGTVKAYFVDGTDIGKFTMKTVDDMRTINKNVHFRPPSNLYDINGLASLWEKK
CAI56319.1_LAR_Gossypium_arboreum          DHFEIYGDGSVKAYFVAGTDIGKFTMKTVDDIRTLNKSVHFRPACNFYNMNELAALWERK
CAI56323.1_LAR_Gossypium_arboreum          DRFQIYGDGTVKAYFVAGSDIGKFTVMSIDDDRTLNKTVHFQPPSNLLNMNEMASLWETK
CAI56324.1_LAR_Gossypium_raimondii         DHFEIYGDGSVKAYFVAGTDIGKFTMKTVDDIRTLNKSVHFRPACNFYNMNELAALWERK
CAI56325.1_LAR_Gossypium_raimondii         DRFQIYGDGTVKAYFVAGSDIGKFTVMSIDDDRTLNKTVHFQPPSNLLNMNEMASLWETK
CAI56328.1_LAR_Oryza_sativa_Japonica_Group DRFQIYGDGDVRAFFVAGSDIGKFTIRAAYDARSINKIVHFRPACNLLSTNEMASLWESK
CAI26308.1_LAR_Vitis_vinifera              DRFHIYGDGSVKAYFVAGTDIGKFTIETINDDRTVNKSLHFRPPSNLVSINELASLWEKK
ABF95070.1_LAR_Oryza_sativa_Japonica_Group DRFQIYGDGDVRAFFVAGSDIGKFTIRAAYDARSINKIVHFRPACNLLSTNEMASLWESK
ADD51358.1_LAR_Theobroma_cacao             DRFKIYGDGTVKAYFVAGTDIGKFTIMSIEDDRTLNKTVHFQPPSNLLNINEMASLWEEK
ACI41981.1_LAR_Diospyros_kaki              DHFQIYGDGSVKAYFVAGSDIGKFTIKAADDIRTLNKSLHFRPPCNFLNINELASMWEKK
ABC71329.1_LAR_Lotus_corniculatus          DQMHIYGDGTVKAYFVDGFDIGKFTMKLVDDARALNKVVHFRPSSNYYSMNELASLWENK
ABH07785.2_LAR_Fragaria_x_ananassa         DQFQIYGDGTVKAYFVDGPDIGKFTMKTVDDIRTMNKNVHFRPSSNLYDINGLASLWEKK
ABB77697.1_LAR_Pyrus_communis              DRFQIYGDGTVKAYFVDGTDIGKFTIKTAKDIRTINKNVHFRPPSNLYDINGLASLWEKK
                                      acc
```

```
sp|Q4W2K4_LAR_Vitis_vinifera                     β12    α8              α9                     TT β13  α10
                                                    250        260        270        280        290        300
sp|Q4W2K4_LAR_Vitis_vinifera               IGRTLPRVTVTEDDLLAAAGENIIPQSVVAAFTHDIFIKGCQVNFSIDGPEDVEVTTLYP
TRINITY_DN33042_c3_g1_i3                   IGRTLPRSTISENDLLAAAAENRIPESIVASFTHDIFIRGCQI.FSIEGPDDVDIATLYP
NP_001352050.1_LAR_Glycine_max             IGRTIPRVTISEDDLLAVAAENCIPRSIVASFTHDIFIKGCQVNFNIDGLGDVEISTLYP
ADD51357.1_LAR_Theobroma_cacao             IGRTLPRVTITEEDLLQMAKEMRIPQSVVAALTHDIFINGCQINFSLDKPTDVEVCSLYP
CAI56321.1_LAR_Pinus_taeda                 INRTLPRVSVSAEDLVMIAKANFMPSSIVAALTHDIFINGCQFKFPIQEPHHVEACELYP
CAI56322.1_LAR_Phaseolus_coccineus         IGRTIPRVTISEDDLLAAAAENCIPQSIVASFTHDIFIKGCQVNFSIDGVDDVEISTLYP
CAI56320.1_LAR_Hordeum_vulgare_subsp._vulgare IGRTLPRVTLSKEELLAMAAEDIIPESIVASLTHDIFINGCQTNFGIDGSRDIEISSLYP
CAD79341.1_LAR_Desmodium_uncinatum         IGRTLPRFTVTADKLLAHAAENIIPESIVSSFTHDIFINGCQVNFSIDEHSDVEIDTLYP
AIS92512_LAR_Epimedium_sagittatum          IKKTLPRKTITEEDLLEAANENRIPESIVAAFTHDIFIKGCQINFPIDGIKDLEATSLYP
AII26024.1_LAR_Pisum_sativum               IARKIPRLFVSENDLLAIAAENIIPESVVASLTHDIFINGCQVSYKIDGVHDVEISTLYP
AEF14422.1_LAR_Onobrychis_viciifolia       LGRKIPRVTVSEDDLLGIAAENCIPESIVASITHDIFINGCQVKFHIDGIHDVEIGTLYP
ABE90657.1_LAR_Medicago_truncatula         IARKIPRAIVSEDDLLGIAAENCIPESVVASITHDIFINGCQVNFKIDGIHDVEISTLYP
CAI56326.1_LAR_Vitis_shuttleworthii        IGRTLPRVTVTEDDLLAAAGENIIPQSVVAAFTHDIFIKGCQVNFSIDGPEDVEVTTLYP
ABC71327.1_LAR_Lotus_corniculatus          IGRKIPRAIVSAEDLLAAAAENCIPRSIVAAFTHDIFINGCQINFSIEGPNDIEIGTLYP
AHA14498.1_LAR_Fagopyrum_tataricum         IGKTLPRVTVTEDDLLDLAAENRIPESIVASFTHDIFIKGCQVDFAVDGPNEVEIEKLYP
AAZ82410.1_LAR_Vitis_vinifera              IGRTLPRVTVTEDDLLAAAGENIIPQSVVAAFTHDIFIKGCQVNFSIDGPEDVEVTTLYP
ADY15310.1_LAR_Prunus_avium                IVRTLPRVTITEDDLLTIAAENRIPESIVASFTHDIFIKGCQINFPVEGPHDVEIGTLYP
BAH89267.1_LAR_Diospyros_kaki              IGRSLPRVTVTEQDLLAAAGENIIPQSIVASFTHDIFIKGCQVNFSIDGPNEVELSSLYP
AAX12186.1_LAR_Malus_domestica             IGRTLPRITITENHLLALAAENRIPESIVASFTHDIFIKGCQVNFAVEGPRDVEVGTLYP
AEY62396.1_LAR_Fagopyrum_dibotrys          IGKTLPRVTVTEDDLLDLAAENRIPESIVASFTHDIFIKGCQVDFAVDGPNEVEIEKLYP
AAZ79364.1_LAR_Malus_domestica             IGRTLPRVTVTEDHLLALAADNRIPESIVASFTHDIFIKGCQVNFAVEGPHDVEVGTLYP
CAI56319.1_LAR_Gossypium_arboreum          IRRTLPRVTVTEEDLLSAAAENIIPQSVVASFTHDIFIKGCQINFPIEGPNETEACSLYP
CAI56323.1_LAR_Gossypium_arboreum          IGRVLPRVTITEQDLLQRAQEMRIPQSVVAAITHDIFINGCQINFSLDKTTDVEICSLYP
CAI56324.1_LAR_Gossypium_raimondii         IRRTLPRVTVTEEDLLSAAAENIIPQSVVASFTHDIFIKGCQINFPIEGPNETEACSLYP
CAI56325.1_LAR_Gossypium_raimondii         IGRVLPRVNITEQDLLQRAQEMRIPQSVVAAITHDIFINGCQINFSLDKTTDVEVCSLYP
CAI56328.1_LAR_Oryza_sativa_Japonica_Group IGRTLPRVTLTEEDLIAMAADDIIPESIVASLTHDIFINGCQTHFYIDGPRDIEISSLYP
CAI26308.1_LAR_Vitis_vinifera              IGRTLPRVTVEEDDLLAAAAEMCIPESIVASFTHDIFIKGCQVNFSLDKPTDLEATSLYP
ABF95070.1_LAR_Oryza_sativa_Japonica_Group IGRTLPRVTLTEEDLIAMAADDIIPESIVASLTHDIFINGCQTHFYIDGPRDIEISSLYP
ADD51358.1_LAR_Theobroma_cacao             IGRTLPRVTITEEDLLQMAKEMRIPQSVVAALTHDIFINGCQINFSLDKPTDVEVCSLYP
ACI41981.1_LAR_Diospyros_kaki              IGRSLPRVTVTEQDLLAAAGENIIPQSIVASFTHDIFIKGCQVNFSIDGPNEVELSSLYP
ABC71329.1_LAR_Lotus_corniculatus          VGRKIPRVTISEDDLLALAAENCIPESIVASITHDIFIKGCQVNYNIDGVHDIEIGTLYP
ABH07785.2_LAR_Fragaria_x_ananassa         IGRTLPKVTITENDLLTMAAENRIPESIVASFTHDIFIKGCQTNFPIEGPNDVDIGTLYP
ABB77697.1_LAR_Pyrus_communis              IGRTLPRITITENHLLALAAENRIPESIVASFTHDIFIKGCQVNFAVEGPRDVEVGTLYP
                                      acc
```

```
                                                       α11
sp|Q4W2K4_LAR_Vitis_vinifera                           310         320

sp|Q4W2K4_LAR_Vitis_vinifera                   EDSFRTVEECFGEYIVKMEE...........K.......QPTADSA..............
TRINITY_DN33042_c3_g1_i3                       EETYQTLDECFEEFLVRLNE.......K..NE......TTTVAPKP..............
NP_001352050.1_LAR_Glycine_max                 EEAFRSLEDCFDAFAIMIDD.......K..........IHKGENK...............
ADD51357.1_LAR_Theobroma_cacao                 DTPFRTINECFEDFAKKIID.......N...........AKAVSKP..............
CAI56321.1_LAR_Pinus_taeda                     DIKYTTMEDFFQGYL.............................................
CAI56322.1_LAR_Phaseolus_coccineus             DEEFRSLEDCYEDFAHMIED.......N..........IHKGEHK...............
CAI56320.1_LAR_Hordeum_vulgare_subsp._vulgare  DIPFRTIDECFDDYARGLHL.........EEE......A.EESKKS..............
CAD79341.1_LAR_Desmodium_uncinatum             DEKFRSLDDCYEDFVPMVHD.......K..........IHAGKSGEIKIKDGKPLVQTGT
AIS92512_LAR_Epimedium_sagittatum              ETPFRTLDDCFDDFLTKTVN.....KKN.ADE......RTKIVDKPL.............
AII26024.1_LAR_Pisum_sativum                   GESFRSMEDCFESFVVMAAD.......K..........IRKGENG...............
AEF14422.1_LAR_Onobrychis_viciifolia           GEEFRSLEDCFGDFVHMAVDNNINNNNN..........NHKGENGG........GVTTTT
ABE90657.1_LAR_Medicago_truncatula             GESFRSLEDCFESFVAMAAD.......K..........IHKGENG...............
CAI56326.1_LAR_Vitis_shuttleworthii            EDSFRTVEECFGEYIVKIEE...........K.......QPTADSA..............
ABC71327.1_LAR_Lotus_corniculatus              DEKFRCLEECFKDFVPMTHD......MN..........VHVGTT................
AHA14498.1_LAR_Fagopyrum_tataricum             KDKYITIDECFEEFVITSNN.......N..KEIIEEVVVTEAFDDE..............
AAZ82410.1_LAR_Vitis_vinifera                  EDSFRTVEECFGEYIVKIEE...........K.......QPTADSA..............
ADY15310.1_LAR_Prunus_avium                    GESFRTLDECFNDFLLKLED.......KLELE...........KNK..............
BAH89267.1_LAR_Diospyros_kaki                  DESFRSVDECFDEFAVKMKD.......M..HQ.......EGAKDDG..............
AAX12186.1_LAR_Malus_domestica                 GDSFRTLDECFNDFLLKLKD.......N..LEP.....VHEE...N..............
AEY62396.1_LAR_Fagopyrum_dibotrys              KDKYITIDECFEEFVITSNN.......N..KEIIEEVVVTEAFDDE..............
AAZ79364.1_LAR_Malus_domestica                 GDSFRTLDECFDGFLLKLKD.......NLELEL.....LQEEEDQK..............
CAI56319.1_LAR_Gossypium_arboreum              NEPFRTLDDCFNDFLAKMKD.......E.NMK.......QSDENTK..............
CAI56323.1_LAR_Gossypium_arboreum              NTSFRTIAECFDDSAKKISD.......N...........EKAVSKP..............
CAI56324.1_LAR_Gossypium_raimondii             NEPFRTLDDCFNDFVAKMKD.......E.NMK.......QSDENTK..............
CAI56325.1_LAR_Gossypium_raimondii             NTSFRTIAECFDDFAKKISD.......N...........EKAVSKP..............
CAI56328.1_LAR_Oryza_sativa_Japonica_Group     DIPFRTIDECFDDYIHVLNL.........AEE......AKEEEEKK..............
CAI26308.1_LAR_Vitis_vinifera                  EMQFRTIDECFDEFVEKIMG.......G...........QAAAEK...............
ABF95070.1_LAR_Oryza_sativa_Japonica_Group     DIPFRTIDECFDDYIHVLNL.........AEE......AKEEEEKK..............
ADD51358.1_LAR_Theobroma_cacao                 DTPFRTINECFEDFAKKIID.......N...........AKAVSKP..............
ACI41981.1_LAR_Diospyros_kaki                  DESFRSVDECFDEFAVKMKD.......M..HQ.......EGAKDDG..............
ABC71329.1_LAR_Lotus_corniculatus              DKTFRSLEDCFEDFVTMIVE.......K..........IHKGENE...............
ABH07785.2_LAR_Fragaria_x_ananassa             EESFRTLDECFNDFLVKVGG.......K..LE...........TDK..............
ABB77697.1_LAR_Pyrus_communis                  GDSFRTLDECFNDFLLKLKD.......N..LEL.....VQEEKDQK..............
                                           acc


sp|Q4W2K4_LAR_Vitis_vinifera
                                                                  330

sp|Q4W2K4_LAR_Vitis_vinifera                   ...................IANT...................................GP
TRINITY_DN33042_c3_g1_i3                       ...................IGNE...................................KT
NP_001352050.1_LAR_Glycine_max                 ...................IAG...................................TES
ADD51357.1_LAR_Theobroma_cacao                 ...................AASN...................................NA
CAI56321.1_LAR_Pinus_taeda                     ............................................................
CAI56322.1_LAR_Phaseolus_coccineus             ...................ITG...................................TKS
CAI56320.1_LAR_Hordeum_vulgare_subsp._vulgare  .........................................................NAP
CAD79341.1_LAR_Desmodium_uncinatum             IEEINKDIKTLVETQPNEEIKKD..................................MKA
AIS92512_LAR_Epimedium_sagittatum              ..................EVTSK............HSSGAAEHMEVISRHNTGA...AEQ
AII26024.1_LAR_Pisum_sativum                   ...................VAGG..................................TKS
AEF14422.1_LAR_Onobrychis_viciifolia           ..................ATAGT..................................KKT
ABE90657.1_LAR_Medicago_truncatula             ...................VTGG..................................TKA
CAI56326.1_LAR_Vitis_shuttleworthii            ...................IANT...................................GP
ABC71327.1_LAR_Lotus_corniculatus              ..................EINNN..................................RKS
AHA14498.1_LAR_Fagopyrum_tataricum             ...................IGNKKQSNKRNVENEEDASGNKKRSSMNKITSTAAAAKSSH
AAZ82410.1_LAR_Vitis_vinifera                  ...................IANT...................................GP
ADY15310.1_LAR_Prunus_avium                    ...................VSNKT..................................NA
BAH89267.1_LAR_Diospyros_kaki                  ...................IAAQ...................................NH
AAX12186.1_LAR_Malus_domestica                 ...................VSTK...................................NA
AEY62396.1_LAR_Fagopyrum_dibotrys              ...................IGNKKQSNKRNVENEEDASGNKKRSSMNKITSTAAAAKSSH
AAZ79364.1_LAR_Malus_domestica                 ...................VSTE...................................NT
CAI56319.1_LAR_Gossypium_arboreum              ...................QSNEIPP...............................PKP
CAI56323.1_LAR_Gossypium_arboreum              ..................VTASN...................................TD
CAI56324.1_LAR_Gossypium_raimondii             ...................QSNEIPP...............................PKP
CAI56325.1_LAR_Gossypium_raimondii             ..................VTASN...................................TD
CAI56328.1_LAR_Oryza_sativa_Japonica_Group     .........................................................NAP
CAI26308.1_LAR_Vitis_vinifera                  ...................AAGK...................................EG
ABF95070.1_LAR_Oryza_sativa_Japonica_Group     .........................................................NAP
ADD51358.1_LAR_Theobroma_cacao                 ...................AASN...................................NA
ACI41981.1_LAR_Diospyros_kaki                  ...................IAAQ...................................NH
ABC71329.1_LAR_Lotus_corniculatus              ...................VYG...................................TKS
ABH07785.2_LAR_Fragaria_x_ananassa             ...................LAAK...................................NK
ABB77697.1_LAR_Pyrus_communis                  ...................VSTK...................................NA
                                           acc
```

sp|Q4W2K4_LAR_Vitis_vinifera

```
                                                         340
                                                           .
sp|Q4W2K4_LAR_Vitis_vinifera                   V.......V.GM......RQVTATCA.......................
TRINITY_DN33042_c3_g1_i3                       VRDETAAMV.EP......LIVTATCA.......................
NP_001352050.1_LAR_Glycine_max                 V.......V.EA......VPFKASCGE..EPPPKKCSKFVVTNY.....
ADD51357.1_LAR_Theobroma_cacao                 I..FVPTAKPGA......LPITAICT.......................
CAI56321.1_LAR_Pinus_taeda                     .................................................
CAI56322.1_LAR_Phaseolus_coccineus             V.......V.EA......VPIMASCGNIYE...................
CAI56320.1_LAR_Hordeum_vulgare_subsp._vulgare  M.......V.EI......LAVYPTCA.......................
CAD79341.1_LAR_Desmodium_uncinatum             L.......V.EA......VPISAMG........................
AIS92512_LAR_Epimedium_sagittatum              I.......I.ESKTDYLHLILSSTDS..............TPYLGYLGS
AII26024.1_LAR_Pisum_sativum                   M.......V.EP......VIITASC........................
AEF14422.1_LAR_Onobrychis_viciifolia           L.......I.EA......VPITASC........................
ABE90657.1_LAR_Medicago_truncatula             L.......V.EP......VPITASC........................
CAI56326.1_LAR_Vitis_shuttleworthii            V.......V.GM......RQVTATCA.......................
ABC71327.1_LAR_Lotus_corniculatus              L.......V.EV......APITAMG........................
AHA14498.1_LAR_Fagopyrum_tataricum             V.......V.EA......LPVPAVC........................
AAZ82410.1_LAR_Vitis_vinifera                  V.......V.GM......RQVTATCA.......................
ADY15310.1_LAR_Prunus_avium                    V.......V.ET......RAVTATCA.......................
BAH89267.1_LAR_Diospyros_kaki                  V.......VEKM......LPITAMCA.......................
AAX12186.1_LAR_Malus_domestica                 V.......V.ES......RAVTPTCA.......................
AEY62396.1_LAR_Fagopyrum_dibotrys              V.......V.EA......LPVPAVC........................
AAZ79364.1_LAR_Malus_domestica                 V.......V.ES......RTVTATCA.......................
CAI56319.1_LAR_Gossypium_arboreum              V.......V.EA......FAITATCA.......................
CAI56323.1_LAR_Gossypium_arboreum              I..FVPTAKPEA......LAITAICT.......................
CAI56324.1_LAR_Gossypium_raimondii             V.......V.EA......FAITATCA.......................
CAI56325.1_LAR_Gossypium_raimondii             I..FVPTAKPEA......LAITAICT.......................
CAI56328.1_LAR_Oryza_sativa_Japonica_Group     T.......V.GR......LAIPPTCA.......................
CAI26308.1_LAR_Vitis_vinifera                  I..VVPASAPDA......LVITATCA.......................
ABF95070.1_LAR_Oryza_sativa_Japonica_Group     T.......V.GR......LAIPPTCA.......................
ADD51358.1_LAR_Theobroma_cacao                 I..FVPTAKPGA......LPITAICT.......................
ACI41981.1_LAR_Diospyros_kaki                  V.......VEKM......LPITAMCA.......................
ABC71329.1_LAR_Lotus_corniculatus              L.......V.EA......VPITASC........................
ABH07785.2_LAR_Fragaria_x_ananassa             AA....VGV.EP......MAITATCA.......................
ABB77697.1_LAR_Pyrus_communis                  V.......V.ES......RAVTPTCA.......................
                                           acc
```

```
sp|Q96323.1_ANS_Arabidopsis_thaliana                 α1         η1   α2         α3
                                         1         10        20        30        40
sp|Q96323.1_ANS_Arabidopsis_thaliana     ..MVAV.......ERVESLAKSGIISIPKEYIRPKEELES...INDVFLEEKK....EDG
TRINITY_DN32893_c8_g1_i1                 ..MMT.....TVVSRVESLSTSGIHSIPKEYIRPKEELTS...IGNVFEEEKK....VKG
NP_001268147.1_ANS_Vitis_vinifera        ..MVT.....SVAPRVESLSSSGIQSIPKEYIRPQEELTS...IGNVFEEEKK....DEG
NP_001312972.1_ANS_Nicotiana_tabacum     MVVISAV..VPTPSRVESLAKSGIQAIPKEYVRPQEELNG...IGNIFEEEKK....DEG
NP_001106074.1_ANS_Zea_mays              ..MESSPLLQLPAARVEALSLSGLSAIPPEYVRPADERAG...LGDAFDLARTHANDHTA
AAD56580.1_ANS_Daucus_carota             ..MVAT...TLESPRVETLAGSGIQLIPKEYVRPKDELIS...ITNIFEEEKS....DEG
ABM66367.1_ANS_Allium_cepa               MPIESVII.APPAPRVETLSKSNLHSIPLEYIRPEHERAC...LGDALEQLHN...SNSG
AFK32781.1_ANS_Fragaria_x_ananassa       ..MVTA...ASIGSRVESLASSGISTIPKEYVRPEEELVN...IGDIFEDEKS....TEG
ALA55544.1_ANS_Lilium_hybrid             ..MPTEI..MPLPGRVESLAGSGLATIPKEYVRPESERDN...LGDAFDEALKL..NSSG
ABU40983.1_ANS_Medicago_truncatula       ...MG.....TVAQRVESLALSGISSIPKEYVRPKEELAN...IGNIFDEEKK.....EG
BAE54520.1_ANS_Spinacia_oleracea         ..MTAT...IAPSRVESLASSGIESIPKEYVRLNEELTS...MGNVFEEEKK....DDG
P51092.1_ANS_Petunia_x_hybrida           ..MVNAV..VTTPSRVESLAKSGIQAIPKEYVRPQEELNG...IGNIFEEEKK....DEG
ACC66093.1_ANS_Ginkgo_biloba             ..MVST........RVESLAQSGLLTIPSEYIRPVEERPTDCIIVNA.EEKNGKYDERT
AAZ79374.1_ANS_Malus_domestica           ..MVSS...DSVNSRVETLAGSGISTIPKEYIRPKDELVN...IGDIFEQEKN....NEG
AAB66560.1_ANS_Callistephus_chinensis    ..MVI.....SANTRVESLATSGSDLIPKEYIRTHDELTT...ITNIFDEEKS....DQG
AAT02642.1_ANS_Citrus_sinensis           ..MVT.....PTARRVESLARSGIQAIPKEYVRPKEELMG...IGNIFEEEEK....DEG
AEN71543.1_ANS_Paeonia_suffruticosa      ..MVN.....SVAPRVESLATSGIQSIPKEYVRTKEEQTS...ITNIFEEEKK....EEG
AFI71900.1_ANS_Paeonia_lactiflora        ..MVN.....SVAPRVESLATSGIQSIPKEYVRTIEEQTS...ITNIFEEEKK....EEG
AGO02175.1_ANS_Nekemias_grossedentata    .MVTT.....SVAPRVESLASSGIQSIPKEYIRPQEELTS...IGNVFEEEKK....DEG
ADD51356.1_ANS_Theobroma_cacao           ..MVT.....SMAPRVESLASSGIQSIPKEYIRPQEELTS...IGNVFEEEKK....EEG
AGL50919.1_ANS_Pyrus_communis            ..MVSS...DSVNSRVETLAGSGISTIPKEYIRPKDELVN...IGDIFEQEKN....NEG
AAU12368.1_ANS_Fragaria_x_ananassa       ..MVTA...ASIGSRVESLASSGISTIPKEYVRPEEELVN...IGDIFEDEKS....TEG
ACC66092.1_ANS_Ginkgo_biloba             ..MVST........RVESLAQSGLLTIPSEYIRPVEERPTDCIIVNA.EEKNGKYDERT
BAB71811.1_ANS_Ipomoea_nil               ..MVSTIT.ATVPSRVERLASSGIERIPKEYIRPEEERRS...IGDIFEEEKI....AGG
CAA69252.1_ANS_Oryza_sativa              ..MTDAEL......RVEALSLSGASAIPPEYVRPEEERAD...LGDALELARAASDDDAT
BAE54521.1_ANS_Phytolacca_americana      ..MVGE....ISPSRVETLATSGIQSIPKEYVRPKEELTS...IGNVFEEEMK....DGG
BAA20143.1_ANS_Perilla_frutescens        ..MVTSA..MGPSPRVEELARSGLDTIPKDYVRPEEELKS..IIGNILAEEKS....SEG
CAA39022.1_ANS_Zea_mays                  ..MESSPLLQLPAARVEALSLSGLSAIPPEYVRPADERAG...LGDAFDLARTHANDHTA
                                    acc

sp|Q96323.1_ANS_Arabidopsis_thaliana        β1              α4               β2          α5
                                             50        60        70        80        90        100
sp|Q96323.1_ANS_Arabidopsis_thaliana     PQVPTIDLKNI..ESDDEKIRENCIEELKKASLDWGVMHLINHGIPADLMERVKKAGEEF
TRINITY_DN32893_c8_g1_i1                 PRVPTIDLKDV..DSNDKNIREKCRAEVIRTSIEWGVMHLVNHGISDQLIGRVKNAGEGF
NP_001268147.1_ANS_Vitis_vinifera        PQVPTIDLKDI..ESEDEVVRERCREELKKAAMEWGVMHLVNHGISDDLINRVKVAGETF
NP_001312972.1_ANS_Nicotiana_tabacum     PQVPTIDLTEI..DSEDKEIREKCHQELKKAAIEWGVMHLVNHGISDDLIDRVKVSGDTF
NP_001106074.1_ANS_Zea_mays              PRIPVVDISPFLDSSSQQQQRDECVEAVRAAAADWGVMHIAGHGIPAELMDRLRAAGTAF
AAD56580.1_ANS_Daucus_carota             PQVPTVDIADI..LSEDKAVREKCYERIKDAAVEWGVMHLVNHGISNELMDRVRVAGQAF
ABM66367.1_ANS_Allium_cepa               PQIPITDL.....DSRD......CIEKVTKAAKEWGVMHIVNHGISSELMEKVRAAGKAF
AFK32781.1_ANS_Fragaria_x_ananassa       PQVPTIDLKEI..DSEDIKVREKCREELKKAAIDWGVMHLINHGISDELMERVKKAGKAF
ALA55544.1_ANS_Lilium_hybrid             PQVPVVDLSGF..DSPDEAVRAKCVEELKKAAEDWGVMHIVNHRIPLELIDRVREVGKGF
ABU40983.1_ANS_Medicago_truncatula       PQVPTIDLKEI..NSSDEIVRGKCREKLKKAAEEWGVMHLVNHGISDDLINRLKKAGETF
BAE54520.1_ANS_Spinacia_oleracea         YQIPTIDIKDI..ASKDQEVRDKAIQELRRAAMEWGVMHLVNYGISDELIHRVKVAGETF
P51092.1_ANS_Petunia_x_hybrida           PQVPTIDLKEI..DSEDKEIREKCHQ.LKKAAMEWGVMHLVNHGISDELINRVKVAGETF
ACC66093.1_ANS_Ginkgo_biloba             GQIPVVDMAGW..EEGGEARRKEIMRQVAEASEEWGVMQVLNHGISDSLIARLQAAGKAF
AAZ79374.1_ANS_Malus_domestica           PQVPTIDLKEI..ESDNEKVRAKCREELKKAAVDWGVMHLVNHGISDELMDKVRKAGKAF
AAB66560.1_ANS_Callistephus_chinensis    PQVPIIDLHDI..NSNDPKTRDKCTYELRKAATEWGVMHLVNHGISSDLINRVKAAGKSF
AAT02642.1_ANS_Citrus_sinensis           PQVPTIDLKEI..DSEDRVEREKCREELKKAAMDWGVMHLVNHGISDDLTERVKRAGQAF
AEN71543.1_ANS_Paeonia_suffruticosa      PQVPTIDLKEI..DSEDIELREKCREELKKAAMDWGVMHLVNHGISDELLERVKKAGEVF
AFI71900.1_ANS_Paeonia_lactiflora        PQVPTIDLKEI..DSEDIKVREKCREELKKAAMDWGVMHLVNHGISNELLEKVKKAGEVF
AGO02175.1_ANS_Nekemias_grossedentata    PQVPTIDLKDI..ESEDEVVRERCREELKKAATEWGVMHLVNHGISDDLLNRVKVAGEAF
ADD51356.1_ANS_Theobroma_cacao           PQVPTIDLKEI..DSEDREVRERCRQELRRAATEWGVMHLVNHGISDELMERVKKAGQKF
AGL50919.1_ANS_Pyrus_communis            PQVPTIDLKEI..ESDNEKVRAKCREELKKAAVDWGVMHLVNHGISDELMDKVRKAGKAF
AAU12368.1_ANS_Fragaria_x_ananassa       PQVPTIDLKEI..DSDDIKVREKCREDLKKAAVEWGVMHLINHGISDELMERVKKAGKAF
ACC66092.1_ANS_Ginkgo_biloba             GQIPVVDMAGW..EEGGEARRKEIMRQVAEASEEWGVMQVLNHGISDSLIARLQAAGKAF
BAB71811.1_ANS_Ipomoea_nil               PQVPTVDLKGI..NSEDLEVREKCREELRKAAVDWGVMHLVNHGISEELTGRVKAAGEGF
CAA69252.1_ANS_Oryza_sativa              ARIPVVDISAF...DNDGDGRHACVEAVRAAAEEWGVMHIAGHGLPGDVLGRLRAAGEAF
BAE54521.1_ANS_Phytolacca_americana      PQVPTIDIRDI..ASDDREVRDRAILELNRAAMEWGVMHLINHGIPNELIDRVKAVGGAF
BAA20143.1_ANS_Perilla_frutescens        PQLPTIDLEEM..DSRDEEGRKKCHEELKKAATDWGVMHLINHGIPEELIDRVKKAGKEF
CAA39022.1_ANS_Zea_mays                  PRIPVVDISPFLDSSSQQQQRDECVEAVRAAAADWGVMHIAGHGIPAELMDRLRAAGTAF
                                    acc

sp|Q96323.1_ANS_Arabidopsis_thaliana       α6   η2     η3    β3        TT       β4        η4   η5
                                               110       120       130       140       150       160
sp|Q96323.1_ANS_Arabidopsis_thaliana     FSLSVEEKEKYANDQATGKIQGYGSKLANNASGQLEWEDYFFHLAYPEEKRDLSIWPKTP
TRINITY_DN32893_c8_g1_i1                 FNLPVEEKEKYANDQISGNVQGYGSKLANNSSGQLEWEDYFFHLIHPENKCDFSIWPKIP
NP_001268147.1_ANS_Vitis_vinifera        FNLPMEEKEKYANDQASGKIAGYGSKLANNASGQLEWEDYFFHLIFPEDKRDMTIWPKTP
NP_001312972.1_ANS_Nicotiana_tabacum     FDQPVEEKEKYANDQPSGNVQGYGSKLANSACGQLEWEDYFFHCVFPEDKCNLSIWPKTP
NP_001106074.1_ANS_Zea_mays              FALPVQDKEAYANDPAAGRLQGYGSRLATNTCGQREWEDYLFHLVHPDGLADHALWPAYP
AAD56580.1_ANS_Daucus_carota             FAEPIGEKEKYANDPGTGMIQGYGSKLANNASGQLEWEDYFFHLVYPEEKADLSIWPKRP
ABM66367.1_ANS_Allium_cepa               FNLPLEAKEEYANDQSKGKIQGYGSKLANNASGQLEWEDYFFHLIFPDDKVDLSVWPKQP
AFK32781.1_ANS_Fragaria_x_ananassa       FDLPIEQKEKYANDQASGKIQGYGSKLANNASGQLEWEDYFFHCVYPEDKRDLSIWPQTP
ALA55544.1_ANS_Lilium_hybrid             FDLPVEQKEKYANDQASGEIQGYGSKLANNESGQLEWEDYYFHLIFPEEKTDLSRWPKEP
ABU40983.1_ANS_Medicago_truncatula       FELPVEEKEKYANDQSSGKIQGYGSKLANNASGQLEWEDYFFHCIFPEDKRDLSIWPKTP
BAE54520.1_ANS_Spinacia_oleracea         FELPVEEKEKYANDQASGNLQGYGSKLANSASGRLEWEDYYFHLCFPEDKRDFSIWPKTP
P51092.1_ANS_Petunia_x_hybrida           FDQPVEEKEKYANDQANGNVQGYGSKLANSACGQLEWEDYFFHCAFPEDKRDLSIWPKNP
ACC66093.1_ANS_Ginkgo_biloba             FDLPIEEKEVYANDSASGKIAGYGSKLANNASGQLEWEDYYFHLLWPTHERDMTTWPKYP
AAZ79374.1_ANS_Malus_domestica           FDLPIELKEKYANDQASGKIQGYGSKLANNASGQLEWEDYFFHCVYPEDKRDLSIWPQTP
AAB66560.1_ANS_Callistephus_chinensis    FDQPVEEKEKYCNDIASGKIQGYGSKLANNACGQLEWEDYFFHLAFPEEKRDLSIWPTTP
AAT02642.1_ANS_Citrus_sinensis           FDQPVEEKEKYANEQASGKIQGYGSKLANNASGQLEWEDYFFHLIYPEDKRDMSIWPKTP
AEN71543.1_ANS_Paeonia_suffruticosa      FQQPVEEKEKYANDQETGKIAGYGSKLANNASGQLEWEDYFFHLVFPEEKRDMSIWPKTP
AFI71900.1_ANS_Paeonia_lactiflora        FQQSVEEKEKYANDQETGKIAGYGSKLANNASGQLEWEDYFFHLVFPEEKRDMSIWPKTP
AGO02175.1_ANS_Nekemias_grossedentata    FNLPIEEKEKYANDQASGKIAGYGSKLANNASGQLEWEDYFFHLIFPEDKRDMSIWPKTP
ADD51356.1_ANS_Theobroma_cacao           FELSVEEKEKYANDQTLGKIQGYGSKLANNASGQLEWEDYFFHLVYPEDKRDLSIWPQTP
AGL50919.1_ANS_Pyrus_communis            FDLPIEQKEKYANDQASGKIQGYGSKLANNASGQLEWEDYFFHCVYPEDKRDLSIWPQTP
AAU12368.1_ANS_Fragaria_x_ananassa       FDLPIEQKEKYANDQASGKIQGYGSKLANNASGQLEWEDYFFHCVYPEDKRDLSIWPQTP
ACC66092.1_ANS_Ginkgo_biloba             FDLPIEEKEVYANDSASGKIAGYGSKLANNASGQLEWEDYYFHLLWPTHERDMTTWPKYP
BAB71811.1_ANS_Ipomoea_nil               FGQPIEEKEKYANDQAAGNVQGYGSKLANNASGQLEWEDYFFHCIFPEDKTDLSIWPKTP
CAA69252.1_ANS_Oryza_sativa              FALPIAEKEAYANDPAAGRLQGYGSKLAANASGKREWEDYLFHLVHPDHLADHSLWPANP
BAE54521.1_ANS_Phytolacca_americana      FELPIEEKEKYANDQATGNVQGYGSKLAENASGQLEWEDYFFHLIFPEDKRDLSIWPTTP
BAA20143.1_ANS_Perilla_frutescens        FELPVEEKEAYANDQAAGNVQGYGSKLANNASGQLEWEDYFFHCVYPEHKTDLSIWPTKP
CAA39022.1_ANS_Zea_mays                  FALPVQDKEAYANDPAAGRLQGYGSRLATNTCGQREWEDYLFHLVHPDGLADHALWPAYP
                                    acc
```

```
sp|Q96323.1_ANS_Arabidopsis_thaliana    α7 α8 α9 β5
                                        170        180       190       200       210

sp|Q96323.1_ANS_Arabidopsis_thaliana    SDYIEATSEYAKCLRLLATKVFKALSVGLGL.EP.DRLEKEVGGL.......EELLLQMK
TRINITY_DN32893_c8_g1_i1                SDYRDATSEYARQLRALATKILSILSLGLGV.EE.GRLEKEVGGV.......EDLLLQMK
NP_001268147.1_ANS_Vitis_vinifera       SDYVPATCEYSVKLRSLATKILSVLSLGLGL.EE.GRLEKEVGGM.......EELLLQKK
NP_001312972.1_ANS_Nicotiana_tabacum    TDYIPATSEYAKQIRNLATKILAVLSIGLRL.EE.GRLEKEVGGM.......EDLLLQMK
NP_001106074.1_ANS_Zea_mays             PDYIAATRDFGRRTRDLASTLLAILSMGLLGTDRGDALEKALTTTTTRTAADDDLLLQLK
AAD56580.1_ANS_Daucus_carota            QDYIPATREYAKELRGLATKLLSALSFGLGL.EE.GRLEKEVGGM.......EELILQMK
ABM66367.1_ANS_Allium_cepa              SDYIEIMQEFGSQLRILASKMLSILSLGLQLPTK.DRLEQELKGP.......EDLLLQLK
AFK32781.1_ANS_Fragaria_x_ananassa      SDCIVATSEYAKELRGLATKMLSILSLGLGL.EE.GRLEKEVGGL.......EELLLQMK
ALA55544.1_ANS_Lilium_hybrid            EDYTEVTKEFAKELRVVVTKMLSMLSQGLGL.ES.GKLEKELGGM.......DDLLMQMK
ABU40983.1_ANS_Medicago_truncatula      ADYTKVTSEYAKELRVLASKIMEVLSLELGL.EG.GRLEKEAGGM.......EELLLQMK
BAE54520.1_ANS_Spinacia_oleracea        ADYIPAVSEYSKELRSLATKILSALSLALGL.EE.GRLEKEVGGI.......EELLLQFK
P51092.1_ANS_Petunia_x_hybrida          TDYTPATSEYAKQIRALATKILTVLSIGLGL.EE.GRLEKEVGGM.......EDLLLQMK
ACC66093.1_ANS_Ginkgo_biloba            SDYIEATDEYGREIRKLVSRILGTLSMELGL.EE.PRMETILGG........QDLEMQLK
AAZ79374.1_ANS_Malus_domestica          ADYIEATAEYAKQLRELATKVLKVLSLGLGL.DE.GRLEKEVGGL.......EELLLQMK
AAB66560.1_ANS_Callistephus_chinensis   HDYIPATTEYAKQLRALATKILAALSLGLGL.EE.GRLEKEVGGM.......EELLVQLK
AAT02642.1_ANS_Citrus_sinensis          SDYTEATSEYARQLRSLATKILAVLSLGLGL.EE.GRLEKEVGGL.......EELLLQMK
AEN71543.1_ANS_Paeonia_suffruticosa     SDYTEATSEYAKQLRILVTKILSILSIGLGL.EE.GRLEQEVGGM.......EELLLQMK
AFI71900.1_ANS_Paeonia_lactiflora       SDYIEATSEYAKQLRILVTKILSIPSIGLGL.EE.GRLEQEVGGM.......EELLLQMK
AGO02175.1_ANS_Nekemias_grossedentata   SDYIPATCEYSVELRGLATKILSVLSLGLGL.EE.GRLEKEVGGM.......EELLLQKK
ADD51356.1_ANS_Theobroma_cacao          SDYTEVTSEYARQLRVLASKILSALSLCLGL.EE.GRLEKEVGGL.......EELLLQMK
AGL50919.1_ANS_Pyrus_communis           ADYIEATAEYAKQLRELATKVLKVLSLGLGL.DE.GRLEKEVGGL.......EELLLQMK
AAU12368.1_ANS_Fragaria_x_ananassa      SDYIVATSEYAKELRGLATKILSILSLGLGL.EE.GRLEKEVGGL.......EELLLQMK
ACC66092.1_ANS_Ginkgo_biloba            SDYIEATDEYGREIRKLVSRILGTLSMELGL.EE.PRMETILGG........QDLEMQLK
BAB71811.1_ANS_Ipomoea_nil              SDYIDATKEYAKQLRALATKVLAVLSLGLGL.EE.GRLEKEVGGM.......EELLLQMK
CAA69252.1_ANS_Oryza_sativa             PEYVPVSRDFGGRVRTLASKLLAILSLGLGLPE..ETLERRLRGHEL.AGVDDDLLLQLK
BAE54521.1_ANS_Phytolacca_americana     SDYICAVSEYAKELRALATKILSALSLALGL.EE.PRLEKEVGGL.......EELLLQLK
BAA20143.1_ANS_Perilla_frutescens       PDYIPATSEYAKQLRALATKILSVLSIGLGL.EK.GRLEKEVGGA.......EDLIVQMK
CAA39022.1_ANS_Zea_mays                 PDYIAATRDFGRRTRDLASTLLAILSMGLLGTDRGDALEKALTTTTTRTAADDDLLLQLK
                                   acc
```

```
sp|Q96323.1_ANS_Arabidopsis_thaliana    TT β6 β7 β8 TT β9 TT β10
                                        220       230       240       250       260       270

sp|Q96323.1_ANS_Arabidopsis_thaliana    INYYPKCPQPELALGVEAHTDVSALTFILHNMVPGLQLFYEGKWVTAKCVPDSIVMHIGD
TRINITY_DN32893_c8_g1_i1                INYYPKCPQPELALGVEAHTDISSLTFILHNMVPGLQLFYKGKWVTAKCVPNSIIMHIGD
NP_001268147.1_ANS_Vitis_vinifera       INYYPKCPQPELALGVEAHTDVSALTFILHNMVPGLQLFYEGKWVTAKCVPNSIIMHIGD
NP_001312972.1_ANS_Nicotiana_tabacum    INYYPKCPQPELALGVEAHTDVSALTFILHNMVPGLQLFYEGQWVTAKCVPNSIIMHIGD
NP_001106074.1_ANS_Zea_mays             INYYPRCPQPELAVGVEAHTDVSALSFILHNGVPGLQVLHGARWVTARHEPGTIIVHVGD
AAD56580.1_ANS_Daucus_carota            INYYPKCPQPELALGVEAHTDVSALSFILHNTVPGLQLFYGGKWVTAKCVPDSIIVHIGD
ABM66367.1_ANS_Allium_cepa              INYYPKCPQPHLALGVEAHTDVSALSFILHNNVPGLQVLYEGEWVTAKLVPDSLIVHVGD
AFK32781.1_ANS_Fragaria_x_ananassa      INYYPKCPLPELALGVEAHTDISALTFILHNMVPGLQLFYGGKWVTAKCVPNSVVMHIGD
ALA55544.1_ANS_Lilium_hybrid            INYYPKCPQPELALGVEAHTDVSSLTFLLTNMVPGLQLYYGGKWVIAQCVPDSLLVHIGD
ABU40983.1_ANS_Medicago_truncatula      INYYPICPQPELALGVEAHTDVSSLTFLLHNMVPGLQLFYEGKWVTAKCVPDSILMHIGD
BAE54520.1_ANS_Spinacia_oleracea        INYYPKCPQPELALGVEAHTDVSALTFILHNMVPGLQLFYEGKWVTAKCVPNSIIMHIGD
P51092.1_ANS_Petunia_x_hybrida          INYYPKCPQPELALGVEAHTDVSALTFILHNMVPGLQLFYEGQWVTAKCVPNSIIMHIGD
ACC66093.1_ANS_Ginkgo_biloba            INYYPRCPQPELALGVEAHTDISALTFLLHNMVPGFQLFKDGKWVTAKCIPGALIVHIGD
AAZ79374.1_ANS_Malus_domestica          INYYPKCPQPELALGVEAHTDVSALTFILHNMVPGLQLFYEGKWVTAKCVPNSIVMHIGD
AAB66560.1_ANS_Callistephus_chinensis   INYYPKCPQPDVALGVEAHTDVSALTFILHNMVPGLQLFYGGKWVTAKCVPDSIIMHIGD
AAT02642.1_ANS_Citrus_sinensis          INYYPKCPQPELALGVEAHTDVSALTFILHNMVPGLQLFYKDKWVTAKCVPNSIILHIGD
AEN71543.1_ANS_Paeonia_suffruticosa     INYYPKCPQPELALGVEAHTDVSALTFILHNMVPGLQLFYQGKWVTAKCVPNSIIMHIGD
AFI71900.1_ANS_Paeonia_lactiflora       INYYPKCPQPELALGVEAHTDVSALTFILHNMVPGLQLFYQGKWVTAKCVPNSIIMHIGD
AGO02175.1_ANS_Nekemias_grossedentata   INYYPKCPQPELALGVEAHTDVSALTFILHNMVPGLQLFYEGKWVTAKCVPNSIIMHIGD
ADD51356.1_ANS_Theobroma_cacao          INYYPKCPQPELALGVEAHTDVSALTFILHNMVPGLQLFYEGKWITAKCVPNSIIMHIGD
AGL50919.1_ANS_Pyrus_communis           INYYPKCPQPELALGVEAHTDVSALTFILHNMVPGLQLFYEGKWVTAKCVPNSIVMHIGD
AAU12368.1_ANS_Fragaria_x_ananassa      INYYPKCPQPELALGVEAHTDISALTFILHNMVPGLQLFYGGKWVTAKCVPNSIVMHIGD
ACC66092.1_ANS_Ginkgo_biloba            INYYPRCPQPELALGVEAHTDISALTFLLHNMVPGFQLFKDGKWVTAKCIPGALIVHIGD
BAB71811.1_ANS_Ipomoea_nil              INYYPKCPQPELALGVEAHTDVSALTFILHNMVPGLQLFYGGKWVTAKCVPNSIIMHVGD
CAA69252.1_ANS_Oryza_sativa             INYYPRCPRPDLAVGVEAHTDVSALSFILHNGVPGLQVHHAGSWVTARPEPGTIVVHVGD
BAE54521.1_ANS_Phytolacca_americana     INYYPKCPQPELALGVEAHTDVSALTFILHNMVPGLQLFYEGKWVTAKCVPNSIIMHIGD
BAA20143.1_ANS_Perilla_frutescens       INFYPKCPQPELALGWEAHTDVSALTFILHNMVPGLQLFYEDKWVTAKCVPNSIIMHIGD
CAA39022.1_ANS_Zea_mays                 INYYPRCPQPELAVGVEAHTDVSALSFILHNGVPGLQVLHGARWVTARHEPGTIIVHVGD
                                   acc
```

```
sp|Q96323.1_ANS_Arabidopsis_thaliana    α10 β11 β12 TTT β13 η6
                                        280       290       300       310       320       330

sp|Q96323.1_ANS_Arabidopsis_thaliana    TLEILSNGKYKSILHRGLVNKEKVRISWAVFCEPPKDKIVLKPLPEMVSV..ESPAKFPP
TRINITY_DN32893_c8_g1_i1                TIEILSNGKYKSILHRGLVNKEKVRISWAVFCEPPKETIILKPLPEVVTE..DEPPLFPP
NP_001268147.1_ANS_Vitis_vinifera       TIEILSNGKYKSILHRGLVNKEKVRISWAVFCEPPKEKIILKPLPETVSE..TEPPLFPP
NP_001312972.1_ANS_Nicotiana_tabacum    TLEILSNGKYKSILHRGVVNKEKIRISWAIFCEPPKEKIILKPLPETITE..AEPPRFPP
NP_001106074.1_ANS_Zea_mays             ALEILSNGRYTSVLHRGLVNREAVRISWVVFCEPPPDSVLLHPLPELVTE..GHPARFTP
AAD56580.1_ANS_Daucus_carota            TLEILSNGKYKSILHRGLVNKEKVRISWAVFCEPPKEKIILKPLPETVTE..AEPPLFPP
ABM66367.1_ANS_Allium_cepa              SLEILSNGIYKSVLHRGLVNKEKVRISWAVFCEPPKDAVVLKPLDEVVTD..DAPARYTP
AFK32781.1_ANS_Fragaria_x_ananassa      TLEILSNGKYKSILHRGLVNKEKVRISWAVFCEPPKEKIILKPLPETVSE..EEPAIFPP
ALA55544.1_ANS_Lilium_hybrid            TLEILSNGRYRSILHRSLVNKERVRISWAVFCEPPKETIVLKPLPELVTE..GAPAKFPP
ABU40983.1_ANS_Medicago_truncatula      TIEILSNGKYKSILHRGLVNKEKVRISWAVFCEPPKEKIILKPLPELVTE..KEPARFPP
BAE54520.1_ANS_Spinacia_oleracea        TIEILSNGKYKSILHRGLVNKEKVRISWAVFCEPPKEKIVLMPLPDLVSG..EEVARYPP
P51092.1_ANS_Petunia_x_hybrida          TIEILSNGKYKSILHRGVVNKEKVRFSWAIFCEPPKEKIILKPLPETVTE..AEPPRFPP
ACC66093.1_ANS_Ginkgo_biloba            QVEILSNGKYKSGLHRGLVNKEKVRISWAVFCDPPKDAMI.GPMKELINE..KNPPLFAA
AAZ79374.1_ANS_Malus_domestica          TLEILSNGKYKSILHRGMVNKEKVRISWAVFCEPPKEKIILKPLPETVSE..EEPAMFPP
AAB66560.1_ANS_Callistephus_chinensis   TIEILSNGKYKSILHRGLVNKEKVRISWAVFCEPPKDKIILKPLPETISE..DEPPLFPP
AAT02642.1_ANS_Citrus_sinensis          TIEILSNGEYKSILHRGLVNKEKVRISWAVFCEPPKDKIILKPLPETVSE..QKPAMFPP
AEN71543.1_ANS_Paeonia_suffruticosa     TIEILSNGKYKSILHRGLVNKDKVRISWAVFCEPPKEKIILKPVLEVVSE..EEPALFPP
AFI71900.1_ANS_Paeonia_lactiflora       TIEILSNGKYKSILHRGLVNKEKVRISWAVFCEPPKEKIILKPLPEVVSE..DEPALFPP
AGO02175.1_ANS_Nekemias_grossedentata   TIEILSNGKYKSILHRGLVNKEKVRISWAVFCEPPKEKIILKPLPETVSE..TEPPLFPP
ADD51356.1_ANS_Theobroma_cacao          TVEILSNGKYKSILHRGLVNKEKVRISWAVFCEPPKEKIILKPLPETVSE..TEPPLFPP
AGL50919.1_ANS_Pyrus_communis           TLEILSNGKYKSILHRGMVNKEKVRISWAVFCEPPKEKIILKPLPETVSE..DEPAMFPP
AAU12368.1_ANS_Fragaria_x_ananassa      TLEILSNGKYKSILHRGLVNKEKVRISWAVFCEPPKEKIILKPLPETVSE..EEPAIFPP
ACC66092.1_ANS_Ginkgo_biloba            QVEILSNGKYKSGLHRGLVNKEKVRISWAVFCDPPKDAMI.GPMKELINE..KNPPLFAA
BAB71811.1_ANS_Ipomoea_nil              TVEILSNGKYKSILHRGVVNREKVRVSWAVFCEPPKDKILLQPLPETVSE..AEPPRFPP
CAA69252.1_ANS_Oryza_sativa             ALEILTNGRYTSVLHRGLVSRDAVRLSWVVFCEPPPESVLLQPVQELLADGAGKPL.FAP
BAE54521.1_ANS_Phytolacca_americana     TIEILSNGKYKSILHRGLVNKEKVRISWAVFCEPPKEKIILKPLTELLTE..EEPARFPP
BAA20143.1_ANS_Perilla_frutescens       TLEILSNGKYKSILHRGLVNKEKVRISWAVFCEPPKEKIVLQPLPETVSE..VEPPRFPP
CAA39022.1_ANS_Zea_mays                 ALEILSNGRYTSVLHRGLVNREAVRISWVVFCEPPPDSVLLHPLPELVTE..GHPARFTP
                                   acc
```

```
                                        β14                          α11
sp|Q96323.1_ANS_Arabidopsis_thaliana
                                                340                               350
sp|Q96323.1_ANS_Arabidopsis_thaliana    RTFAQHIEHKLFGK.............................EQEELVSEKND......
TRINITY_DN32893_c8_g1_i1                RTFAQHLEHKLFRK.............................SQEALLANK........
NP_001268147.1_ANS_Vitis_vinifera       RTFSQHIQHKLFRK.............................TQEALLSK.........
NP_001312972.1_ANS_Nicotiana_tabacum    RTFAQHMAHKLFKKDDQDAAAEHKVSKKDDPDSAAEHKPFKKDDQDAVAQQKV...LKED
NP_001106074.1_ANS_Zea_mays             RTFKQHLDRKLFKK.............................KQQHKAKAEEE......
AAD56580.1_ANS_Daucus_carota            RTFAQHMAHKLFRK.............................SQEAIDDSKK...VQPQ
ABM66367.1_ANS_Allium_cepa              RTFAQHLERKLFKK.........KVGDL...............DDSDV............
AFK32781.1_ANS_Fragaria_x_ananassa      RTFFEHIQHKLFRQ.............................SQEALVSTKESAALKST
ALA55544.1_ANS_Lilium_hybrid            RTFKQHIQHKLFKK.............................TEEDFTSLK........
ABU40983.1_ANS_Medicago_truncatula      RTFAQHIHHKLFRK.............................DEEEKKDDPK.......
BAE54520.1_ANS_Spinacia_oleracea        RTFAQHVQYKLFRK.............................TQD...P..........
P51092.1_ANS_Petunia_x_hybrida          RTFAQHMAHKLFRKDDKDAAVEHKVFNEDELDTAAEHKVLKKDNQDAVAENKD...IKED
ACC66093.1_ANS_Ginkgo_biloba            KTFKDHTDHKLFKK.............................GQSKKN...........
AAZ79374.1_ANS_Malus_domestica          RTFAEHIQHKLFRK.............................SQGALLPK.........
AAB66560.1_ANS_Callistephus_chinensis   RTFQQHMEHKLFRK.............................NNDAVDPK.........
AAT02642.1_ANS_Citrus_sinensis          RTFQQHIEHKLFRR.............................TQDALLSDEE.......
AEN71543.1_ANS_Paeonia_suffruticosa     RTFAQHIQHKLFRK.............................TQEEFKN..........
AFI71900.1_ANS_Paeonia_lactiflora       RTFAQHIQHKLFRK.............................TQEEFKN..........
AGO02175.1_ANS_Nekemias_grossedentata   RTFAQHIQHKLFRK.............................TQEALLTK.........
ADD51356.1_ANS_Theobroma_cacao          RTFAQHIHHKLFRK.............................TQDGLSN..........
AGL50919.1_ANS_Pyrus_communis           RTFAEHIQHKLFRK.............................SQEALLPK.........
AAU12368.1_ANS_Fragaria_x_ananassa      RTFFEHIQHKLFRQ.............................SQEALVSTKESAALKST
ACC66092.1_ANS_Ginkgo_biloba            KTFKDHTDHKLFKK.............................GQSKKN...........
BAB71811.1_ANS_Ipomoea_nil              RTFAQHIKHKLFRQ............................SDQEAADTPKPD...NDD
CAA69252.1_ANS_Oryza_sativa             RTFKQHVQRKLFKK..........................LKDQQDNNAAA.........
BAE54521.1_ANS_Phytolacca_americana     RTFAQHIQYKLFRK.............................TQDVQAPVSN.......
BAA20143.1_ANS_Perilla_frutescens       RTFAQHLKHKLFRK.............................TDGDLDEKPTY......
CAA39022.1_ANS_Zea_mays                 RTFKQHLDRKLFKK.............................KQQHKAKAEEE......
                                  acc

sp|Q96323.1_ANS_Arabidopsis_thaliana

sp|Q96323.1_ANS_Arabidopsis_thaliana    .......................................................
TRINITY_DN32893_c8_g1_i1                .......................................................
NP_001268147.1_ANS_Vitis_vinifera       .......................................................
NP_001312972.1_ANS_Nicotiana_tabacum    E..............QNAAAEHKVFKKDNQDAAAEESK.................
NP_001106074.1_ANS_Zea_mays             ............DGGNGDHHRHEPPPQTN..........................
AAD56580.1_ANS_Daucus_carota            EQ.............NNAETD...IPQPEEQQKTEESNPQKIEILKPGEEASSSP
ABM66367.1_ANS_Allium_cepa              .......................................................
AFK32781.1_ANS_Fragaria_x_ananassa      T..............ESAL.......KSTKEAALISTN.................
ALA55544.1_ANS_Lilium_hybrid            .......................................................
ABU40983.1_ANS_Medicago_truncatula      ..........................K............................
BAE54520.1_ANS_Spinacia_oleracea        .......................................................
P51092.1_ANS_Petunia_x_hybrida          EQCGPAEHKDIKEDGQGAAAENKVFKENNQDVAAEESK.................
ACC66093.1_ANS_Ginkgo_biloba            .......................................................
AAZ79374.1_ANS_Malus_domestica          .......................................................
AAB66560.1_ANS_Callistephus_chinensis   .......................................................
AAT02642.1_ANS_Citrus_sinensis          .......................................................
AEN71543.1_ANS_Paeonia_suffruticosa     .......................................................
AFI71900.1_ANS_Paeonia_lactiflora       .......................................................
AGO02175.1_ANS_Nekemias_grossedentata   .......................................................
ADD51356.1_ANS_Theobroma_cacao          .......................................................
AGL50919.1_ANS_Pyrus_communis           .......................................................
AAU12368.1_ANS_Fragaria_x_ananassa      T..............ESAL.......KSTKEAALISTN.................
ACC66092.1_ANS_Ginkgo_biloba            .......................................................
BAB71811.1_ANS_Ipomoea_nil              HH........................QSN..........................
CAA69252.1_ANS_Oryza_sativa             .............ASNGMITK..................................
BAE54521.1_ANS_Phytolacca_americana     .......................................................
BAA20143.1_ANS_Perilla_frutescens       .......................................................
CAA39022.1_ANS_Zea_mays                 ............DGGNGDHHRHEPPPQTN..........................
                                  acc
```

```
BAD89742.1_ANR_Vitis_vinifera
                                                                                                β1
                                                                              1          10         20
BAD89742.1_ANR_Vitis_vinifera                     ...............................MATQHP....IGKKTACVVGGTGFVASL
TRINITY_DN30161_c9_g1_i2                          ...............................MATQL.....NPNKTACVIGGSGFVASL
TRINITY_DN30161_c9_g1_i3                          ...............................MATQL.....NPNKTACVIGGSGFVASL
Q9SEV0.2_ANR_AT1G61720_Arabidopsis_thaliana       .................................MDQTL.THTGSKKACVIGGTGNLASI
AKV89239.1_ANR_Prunus_cerasifera                  ...............................MAT.QP....ISKKTACVIGGTGFVASL
AJK93561.1_ANR_Vicia_faba                         ...............................MASIK.....EIGKKKACVIGGTGFVASM
AII26022.1_ANR_Pisum_sativum                      ...............................MASIK.....EVGKKKACVIGGTGFVASI
AGL81352.1_ANR_Pyrus_communis                     ...............................MATQQP....ISKKTACVVGGTGFVASL
ACV72641.1_ANR_Gossypium_hirsutum                 ...............................MASQL.....VGTKRACVVGGSGFVASL
ADD51353.1_ANR_Theobroma_cacao                    ...............................MASQT.....VGKKTACVVGGTGYVASL
AAT68773.1_ANR_Camellia_sinensis                  ...............................MEAQP.....TAPKAACVVGGTGFVAAT
ABM64802.1_ANR_Gossypium_hirsutum                 ...............................MASQI.....VGTKRACVVGGSGFVASL
AAN77735.1_ANR_Medicago_truncatula                ...............................MASIKQ..IEIEKKKACVIGGTGFVASL
ASU87432.1_ANR_Camellia_sinensis                  ...............................MEAQP.....TAPKAACVVGGTGFVAAT
AEC10993.1_ANR_Camellia_sinensis                  ...............................MEAQP.....TAPKAACVVGGTGFVAAT
ADZ58168.1_ANR_Camellia_sinensis                  ..........................MAMAMATTTTTTKPMIGAKAACAVGGTGFVAAT
AHJ11240.1_ANR_Camellia_sinensis                  ..........................MAMAMATTTTATKPMIGAKAACVVGGTGFVAAT
NP_001267885.1_ANR_Vitis_vinifera                 ...............................MATQHP....IGKKTACVVGGTGFVASL
ABD95362.1_ANR_Fragaria_x_ananassa                ...............................MSTTQP...IISTKSACVIGGTGFVASQ
AEL79861.1_ANR_Malus_domestica                    ...............................MATQQP....ISKKTACVVGGTGFVASL
BAF56654.1_ANR_Diospyros_kaki                     ...............................MAAAPPP..TLGAKAACVIGGTGFVAAT
ADD51354.1_ANR_Theobroma_cacao                    ...............................MASQT.....VGKKTACVVGGTGYVASL
CAD91909.1_ANR_Phaseolus_coccineus                ...............................MATVK....KIG.KKACVIGGSGFMASC
ABM90632.1_ANR_Lotus_uliginosus                   ...............................MASIKP...IYEKKKACVIGGTGFVASL
ABC71336.1_ANR_Lotus_corniculatus                 ...............................MASIKP...IYEKKKACVIGGTGFVASL
XP_002317270.2_ANR_Populus_trichocarpa            MHPPMEELNQIKFIFNLARKNSDMIHIFSSSMASQT.....K.KNTACVIGGTGFVASL
CAD91910.1_ANR_Gossypium_arboreum                 ...............................MASQL.....VGTKRACVVGGSGFVASL
AEL79859.1_ANR_Malus_domestica                    ...............................MATQQP....ISKKTACVVGGTGFVASL
AEL79860.1_ANR_Malus_domestica                    ...............................MATQQP....ISKKTACVVGGTGFVASL
AEL79861.1_ANR_Malus_domestica                    ...............................MATQQP....ISKKTACVVGGTGFVASL
ACY30421.1_C.BANa_Brassica_napus                  ...............................MTSTDQT...TGTKKVCVIGGTGNLASI
ACY30422.1_C.BANb_Brassica_napus                  ..............................MATVDQTA.VTTGTKKACVIGGTGNLASI
ACY30423.1_A.BANa_Brassica_napus                  ...............................MTSTDQT...TGTKKVCVIGGTGNLASI
ACY30424.1_A.BANb_Brassica_napus                  ..............................MATVDQTA.VTTGTKKACVIGGTGNLASI
ACY30425.1_C.BANa_Brassica_oleracea               ...............................MTSTDQT...TGTKKVCVIGGTGNLASI
ABG76842.1_ANR_Fragaria_x_ananassa                ...............................MATTQP...IISTKSACVIGGTGFVASQ
ABC71337.1_ANR-1_Lotus_corniculatus               ...............................MASIKP...IYEKKKACVIGGTGFVASL
ABC71332.1_ANR1-1_Lotus_corniculatus              ...............................MASIKP...IYEKKKACVIGGTGFVASL
ABC71333.1_ANR1-2_Lotus_corniculatus              ...............................MASIKP...IYEKKKACVIGGTGFVASL
ABC71335.1_ANR1-4_Lotus_corniculatus              ...............................MASIKP...IYEKKKACVIGGTGFVASL
AAZ79363.1_ANR_Malus_domestica                    ...............................MATQQP....ISKKTACVVGGTGFVASL
AAX12184.1_ANR_Malus_domestica                    ...............................MATQQP....ISKKTACVVGGTGFVASL
AAZ17408.1_ANR_Malus_domestica                    ...............................MATQQP....ISKKTACVVGGTGFVASL
EEE86150.1_ANR_Populus_trichocarpa                ...............................MASQL.....T.KKTACVIGGTGFVASL
EEE97882.1_ANR_Populus_trichocarpa                ...............................MASQT.....K.KNTACVIGGTGFVASL
ABB77695.1_ANR_Pyrus_communis                     ...............................MATQQP....ISKKTACVVGGTGFVASL
BAD89742.1_ANR_Vitis_vinifera                     ...............................MATQHP....IGKKTACVVGGTGFVASL
AAZ82409.1_ANR_Vitis_vinifera                     ...............................MATQHP....IGKKTACVVGGTGFVASL
                                             acc
```

```
BAD89742.1_ANR_Vitis_vinifera
                                                  α1             β2                   α2     η1       β3              α3
                                                      30         40          50         60         70         80
BAD89742.1_ANR_Vitis_vinifera                     LVKLLLQKGYAVNTTVRDPDNQKKVSHLLELQELGDLKIFRADLTDELSFEAPIAGCDFV
TRINITY_DN30161_c9_g1_i2                          LVKLLLQKGYTVHTTVRDPANQKKISHLLPLKELGGLRIFGADLTNEESFNAPIAGCELV
TRINITY_DN30161_c9_g1_i3                          LVKLLLQKGYTVHTTVRDPANQKKISHLLPLKELGGLRIFGADLTNEESFNAPIAGCELV
Q9SEV0.2_ANR_AT1G61720_Arabidopsis_thaliana       LIKHLLQSGYKVNTTVRDPENEKKIAHLRKLQELGDLKIFKADLTDEDSFESSFSGCEYI
AKV89239.1_ANR_Prunus_cerasifera                  LVKLLLEKGYAVKTTVRDPDNQKKISHLTALQDLGELEIFAADLTDEGSFDAPIAGCDLV
AJK93561.1_ANR_Vicia_faba                         LIKQLLEKGYAVNTTVRDPDNPKKTSHLVALQSLGELNMFKAELTVEEDFDAPIAGCELV
AII26022.1_ANR_Pisum_sativum                      LIKQLLEKGYAVNTTVRDLDNPKKISHLVALKSLGELNLFKAELTVDGDFDAPIAGCELV
AGL81352.1_ANR_Pyrus_communis                     LVKLLLQKGYAVRTTVRDPDNHKKVSHLTALQELGELEILAGDLTDEGSFDAPIAGCDLV
ACV72641.1_ANR_Gossypium_hirsutum                 LVKLLLEKGYAVNTTVRDPDNQKKISHLVTLQELGDLKIFQADLTDEGSFDAPIAGCDLV
ADD51353.1_ANR_Theobroma_cacao                    LVKLLLEKGYAVNTTVRDPDNQKKIPHLVTLQKLGDLKIFRADLTDEGSFDVPIAGCDLV
AAT68773.1_ANR_Camellia_sinensis                  LIKLLLEKGYAVNTTVRDPGNQKKTSHLLALKGSGNLKIFRADLTDEQSFDTPVAGCDLV
ABM64802.1_ANR_Gossypium_hirsutum                 LVKLLLEKGYAVNTTVRDPDNQKKISHLVTLQELGDLKIFQADLTDEGSFDAPIAGCDLV
AAN77735.1_ANR_Medicago_truncatula                LIKQLLEKGYAVNTTVRDLDSANKTSHLIALQSLGELNLFKAELTIEEDFDAPISGCELV
ASU87432.1_ANR_Camellia_sinensis                  LIKLLLEKGYAVNTTVRDPGNQKKTSHLLALKGSGNLKIFRADLTDEQSFDTPVAGCDLV
AEC10993.1_ANR_Camellia_sinensis                  LIKLLLEKGYAVNTTVRDPGNQKKTSHLLALKGSGNLKIFRADLTDEQSFDTPVAGCDLV
ADZ58168.1_ANR_Camellia_sinensis                  LVKMLLERGYSVNTTVRDPDNKKNISHLVALEGMGNLKIFRADLTDEQSFDAPIAGCDLV
AHJ11240.1_ANR_Camellia_sinensis                  LVKMLLERGYSVNTTVRDPDNKKNISHLVALEGMGNLKIFRADLTDEQSFDAPIAGCDLV
NP_001267885.1_ANR_Vitis_vinifera                 LVKLLLQKGYAVNTTVRDPDNQKKVSHLLELQELGDLKIFRADLTDELSFEAPIAGCDFV
ABD95362.1_ANR_Fragaria_x_ananassa                LIKLLLEKGYAVRTTVRDPDNLKKISHLTALQELGELTIFRGDLTDEGSFDAAIAGSDLV
AEL79861.1_ANR_Malus_domestica                    LVKLLLQKGYAVRTTVRDPDNHKKVSHLTALQELGELEILAGDLTDEGSFDAPIAGCDLV
BAF56654.1_ANR_Diospyros_kaki                     LIKLLLEKGYAVNTTVRDPDDKKKISHLLTMKSLGSLNIFQAELTDEKSFDAPIAGCDLV
ADD51354.1_ANR_Theobroma_cacao                    LVKLLLEKGYAVNTTVRDPDNQKKIPHLVTLQKLGDLKIFRADLTDEGSLDVPIAGCDLV
CAD91909.1_ANR_Phaseolus_coccineus                LVKQLLHKGYAVNTTVRDPDNAKKISHLLALQSLGDLNIFGADLTGERDFDAPIAGCELV
ABM90632.1_ANR_Lotus_uliginosus                   LIKQLLEKGYAVNTTVRDPDNHKKISHLVALQSLGELNIFGGELTVENDFDAPIAGSQLV
ABC71336.1_ANR_Lotus_corniculatus                 LIKQLLEKGYAVNTTVRDP.......................................GSELV
XP_002317270.2_ANR_Populus_trichocarpa            LIKLLLEKGYAVNTTVRDPDNQKKIAHLIALQNLGDLNIFGADLTNEESFNAPIACCDLV
CAD91910.1_ANR_Gossypium_arboreum                 LVKLLLEKGFAVNTTVRDPDNQKKISHLVTLQELGDLKIFQADLTDEGSFDAPIAGCDLV
AEL79859.1_ANR_Malus_domestica                    LVKLLLQKGYAVRTTVRDPDNHKKVSHLTALQELGELEILAGDLTDEGSFDAPIAGCDLV
AEL79860.1_ANR_Malus_domestica                    LVKLLLQKGYAVRTTVRDPDNHKKVSHLTSLQELGELEILAGDLTDEGSFDAPIAGCDLV
AEL79861.1_ANR_Malus_domestica                    LVKLLLQKGYAVRTTVRDPDNHKKVSHLTALQELGELEILAGDLTDEGSFDAPIAGCDLV
ACY30421.1_C.BANa_Brassica_napus                  LIDRLLQSGYEVNTTVRDPENVKKMAVLRVLQEQGDLNIFKADLTDEGSFNSPVSGCEYV
ACY30422.1_C.BANb_Brassica_napus                  LIKHLLQSGYKVNTTVRDPENEKKMAHLKVLQELGDLKIFKADLTDEGSFTSPISGCEYV
ACY30423.1_A.BANa_Brassica_napus                  LIDRLLQSGYEVNTTVRDPENEKKMAVLRVLQEQGDLNIFKADLTDEGSFNSPVSGCEYV
ACY30424.1_A.BANb_Brassica_napus                  LIKHLLQSGYKVNTTVRDPENEKKMAHLKVLQQLGDLKIFKADLTDEGSFTSPISGCEYV
ACY30425.1_C.BANa_Brassica_oleracea               LIDRLLQSGYEVNTTVRDPENVKKMAVLRVLQEQGDLNIFKADLTDEGSFNSPVSGCEYV
ABG76842.1_ANR_Fragaria_x_ananassa                LIKLLLEKGYAVRTTVRDPDNLKKISHLTALQELGELTIFRGDLTDEGSFDAAIAGSDLV
ABC71337.1_ANR-1_Lotus_corniculatus               LIKQLLEKGYAVNTTVRDPDNHKKISHLLVLQSLGELNIFGGELTVENDFDTPIEGSELV
ABC71332.1_ANR1-1_Lotus_corniculatus              LIKQLLEKGYAVNTTVRDPDNHKKISHLLALQSLGELNIFGGELTVENDFDTPIAGSELV
ABC71333.1_ANR1-2_Lotus_corniculatus              LIKQLLEKGYAVNTTVRDPDNHKKISHLLALQSWGELNIFEGELTVENDFDTPIAGSELV
ABC71335.1_ANR1-4_Lotus_corniculatus              LIKQLLEKGYAVNTTVRDPDNHKKISHLLALQSLGELNIFGGELTVENDFDTPIEGSELV
AAZ79363.1_ANR_Malus_domestica                    LVKLLLQKGYAVRTTVRDPDDRKKVSHLTALQELGELEIFAGDLTDEGSFDAPIAGCDLV
AAX12184.1_ANR_Malus_domestica                    LVKLLLQKGYAVRTTVRDPDNHKKVSHLTALQELGELEIFAGDLTDEGSFDAPIAGCDLV
AAZ17408.1_ANR_Malus_domestica                    LVKLLLQKGYAVRTTVRDPDNHKKVSHLTALQELGELEILAGDLTDEGSFDAPIAGCDLV
EEE86150.1_ANR_Populus_trichocarpa                LVKLLLEKGYAVNTTVRDPDNQKKVAHLIALQNLGDLNIFGADLTDEESFNAPIAGCELV
EEE97882.1_ANR_Populus_trichocarpa                LIKLLLEKGYAVNTTVRDPDNQKKIAHLIALQNLGDLNIFGADLTNEESFNAPIACCDLV
ABB77695.1_ANR_Pyrus_communis                     LVKLLLQKGYAVRTTVRDPDNHKKVSHLTALQELGELEILAGDLTDEGSFDAPIAGCDLV
BAD89742.1_ANR_Vitis_vinifera                     LVKLLLQKGYAVNTTVRDPDNQKKVSHLLELQELGDLKIFRADLTDELSFEAPIAGCDFV
AAZ82409.1_ANR_Vitis_vinifera                     LVKLLLQKGYAVNTTVRDPDNQKKVSHLLELQELGDLKIFRADLTDELSFEAPIAGCDFV
                                             acc
```

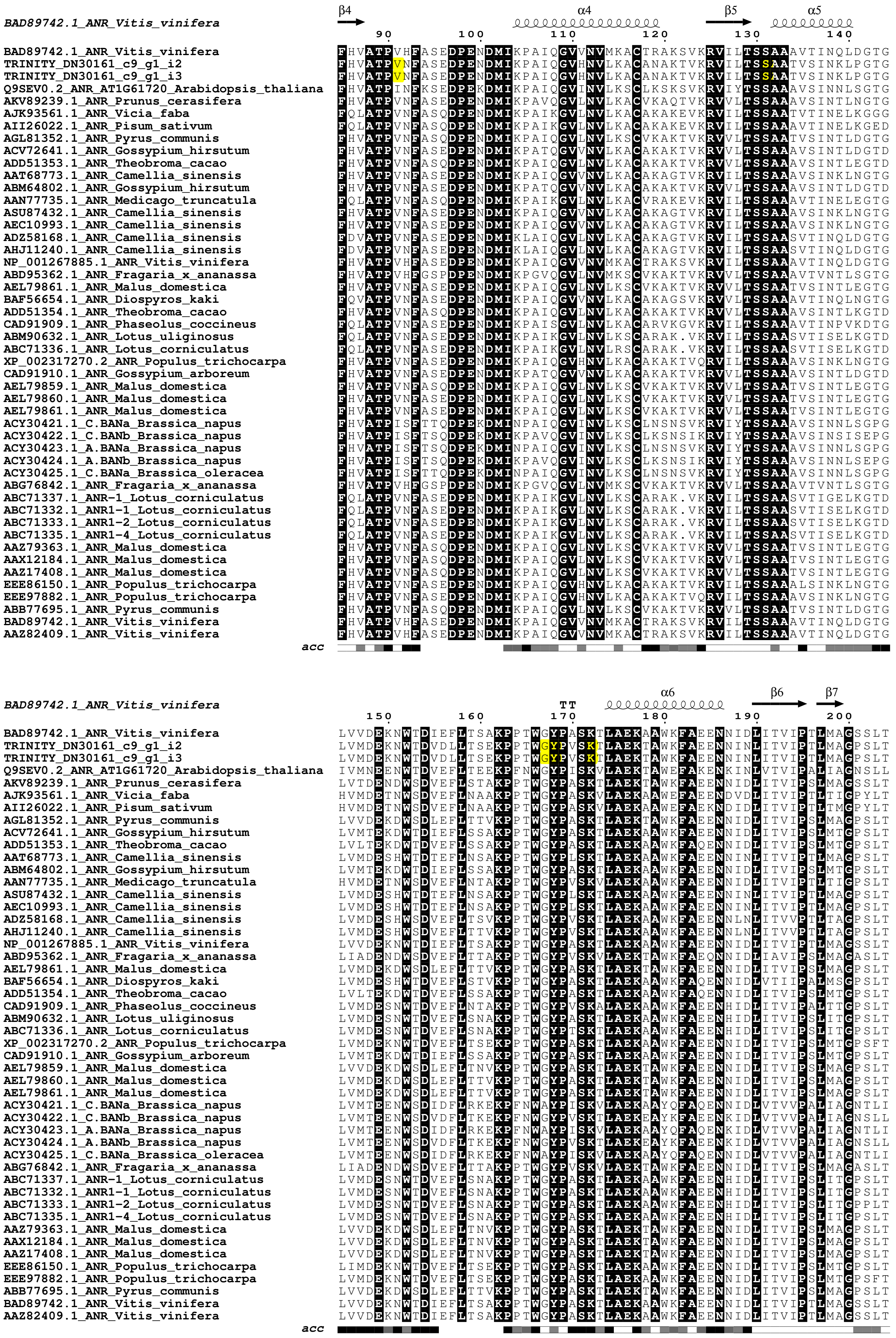

BAD89742.1_ANR_Vitis_vinifera
β4
α4
β5
α5
acc
BAD89742.1_ANR_Vitis_vinifera
TT
α6
β6
β7
acc

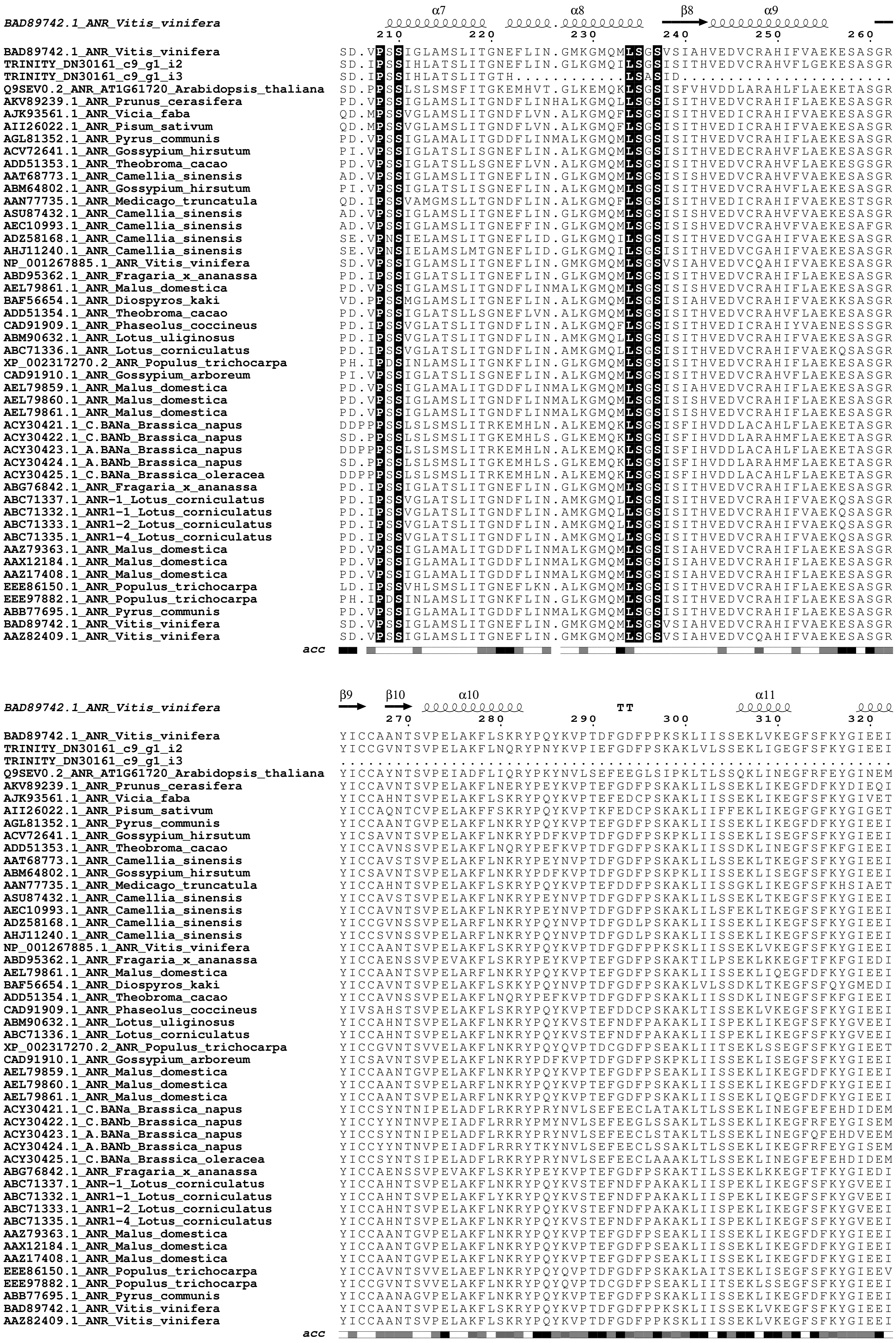

BAD89742.1_ANR_Vitis_vinifera
α7
α8
β8
α9
210
220
230
240
250
260
acc
BAD89742.1_ANR_Vitis_vinifera
β9
β10
α10
TT
α11
270
280
290
300
310
320
acc

	α12
BAD89742.1_ANR_Vitis_vinifera	
	330
**BAD89742.1_ANR_Vitis_vinifera**	YDESVEYFKAKGLLQN.
**TRINITY_DN30161_c9_g1_i2**	YDQTVEYLKGKGILK..
**TRINITY_DN30161_c9_g1_i3**	....
**Q9SEV0.2_ANR_AT1G61720_Arabidopsis_thaliana**	YDQMIEYFESKGLIKAK
**AKV89239.1_ANR_Prunus_cerasifera**	YDQAVDYFKAKGLLQN.
**AJK93561.1_ANR_Vicia_faba**	FDHTVEYLKTKGILKE.
**AII26022.1_ANR_Pisum_sativum**	YAQTIEYLKIKGVLKK.
**AGL81352.1_ANR_Pyrus_communis**	YDQTVEYFKAKGLLQN.
**ACV72641.1_ANR_Gossypium_hirsutum**	YDQTVEYLKSKGLLK..
**ADD51353.1_ANR_Theobroma_cacao**	YDQTVEYMNAKGLLK..
**AAT68773.1_ANR_Camellia_sinensis**	YDQSVEYFKAKGILKN.
**ABM64802.1_ANR_Gossypium_hirsutum**	YDQTVEYLKSKGLLK..
**AAN77735.1_ANR_Medicago_truncatula**	FDQTVEYLKTQGIK...
**ASU87432.1_ANR_Camellia_sinensis**	YDQSVEYFKAKGILKN.
**AEC10993.1_ANR_Camellia_sinensis**	YDQSGEYFKVKGILKN.
**ADZ58168.1_ANR_Camellia_sinensis**	FDHSVAYLKTKGLLQN.
**AHJ11240.1_ANR_Camellia_sinensis**	FDHSVAYLKTKGLLQN.
**NP_001267885.1_ANR_Vitis_vinifera**	YDESVEYFKAKGLLQN.
**ABD95362.1_ANR_Fragaria_x_ananassa**	YDQTVEYLKLKGVLQN.
**AEL79861.1_ANR_Malus_domestica**	YDQTVEYFKAKGLLQN.
**BAF56654.1_ANR_Diospyros_kaki**	YDQSVEYFKAKGILKN.
**ADD51354.1_ANR_Theobroma_cacao**	YDQTVEYMNAKGLLK..
**CAD91909.1_ANR_Phaseolus_coccineus**	YDQTVEYLKNKGTLKN.
**ABM90632.1_ANR_Lotus_uliginosus**	FDQTLEYLKTKGALKN.
**ABC71336.1_ANR_Lotus_corniculatus**	FDQTLEYLKTKGALKN.
**XP_002317270.2_ANR_Populus_trichocarpa**	YDQTVEYFKANGLLN..
**CAD91910.1_ANR_Gossypium_arboreum**	YDQTVEYLKSKGLLK..
**AEL79859.1_ANR_Malus_domestica**	YDQTVEYFKAKGLLQK.
**AEL79860.1_ANR_Malus_domestica**	YDQTVEYFKAKGLLQN.
**AEL79861.1_ANR_Malus_domestica**	YDQTVEYFKAKGLLQN.
**ACY30421.1_C.BANa_Brassica_napus**	YDQMVEYFKTNRWA...
**ACY30422.1_C.BANb_Brassica_napus**	YDEMTKYFESKGLIKP.
**ACY30423.1_A.BANa_Brassica_napus**	YDQMVEHFKTNRWA...
**ACY30424.1_A.BANb_Brassica_napus**	YDEMTEYFESKGLIKP.
**ACY30425.1_C.BANa_Brassica_oleracea**	YDQMVEYFKTNRWA...
**ABG76842.1_ANR_Fragaria_x_ananassa**	YDQTVEYLKLKGVLQN.
**ABC71337.1_ANR-1_Lotus_corniculatus**	FDQTLEYLKTKGALKN.
**ABC71332.1_ANR1-1_Lotus_corniculatus**	FDQTLEYLKTKGALKN.
**ABC71333.1_ANR1-2_Lotus_corniculatus**	FDQTLEYLKTKGALKN.
**ABC71335.1_ANR1-4_Lotus_corniculatus**	FDQTLEYLKTKGALKN.
**AAZ79363.1_ANR_Malus_domestica**	YDQTVEYFKAKGLLQN.
**AAX12184.1_ANR_Malus_domestica**	YDQTVEYFKAKGLLQN.
**AAZ17408.1_ANR_Malus_domestica**	YDQTVEYFKAKGLLQK.
**EEE86150.1_ANR_Populus_trichocarpa**	YDQTVEYFKAKGLLN..
**EEE97882.1_ANR_Populus_trichocarpa**	YDQTVEYFKANGLLN..
**ABB77695.1_ANR_Pyrus_communis**	YDQTVEYFKAKGLLQN.
**BAD89742.1_ANR_Vitis_vinifera**	YDESVEYFKAKGLLQN.
**AAZ82409.1_ANR_Vitis_vinifera**	YDESVEYFKAKGLLQN.
acc	
