# Supplementary figures and images for: Automatic Identification of Players in the Flavonoid Biosynthesis with Application on the Biomedicinal Plant Croton tiglium
Source: Plants (Basel). 2020 Aug 27;9(9):1103. doi: 10.3390/plants9091103 (PMC7570183; doi:10.3390/plants9091103)

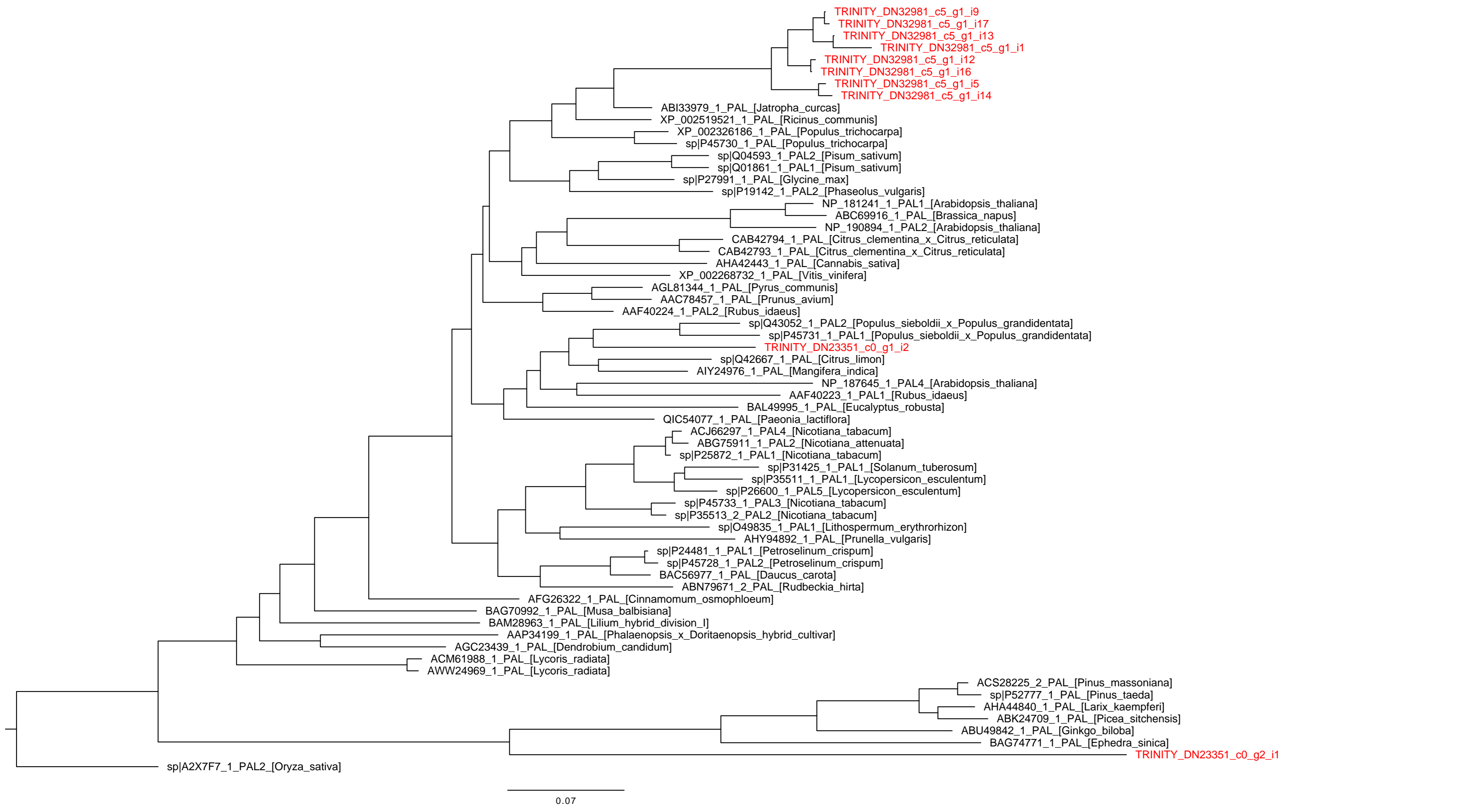

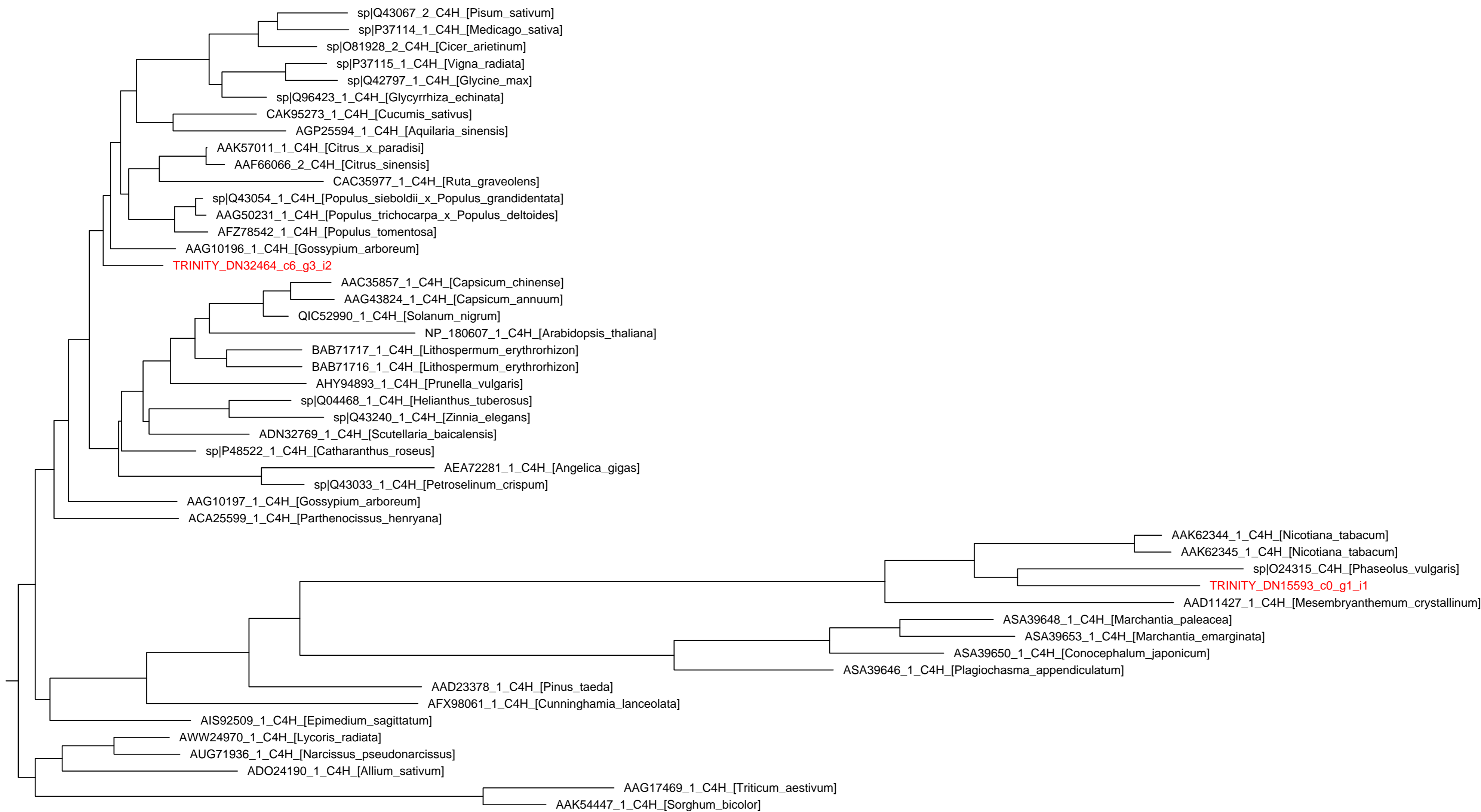

0.08

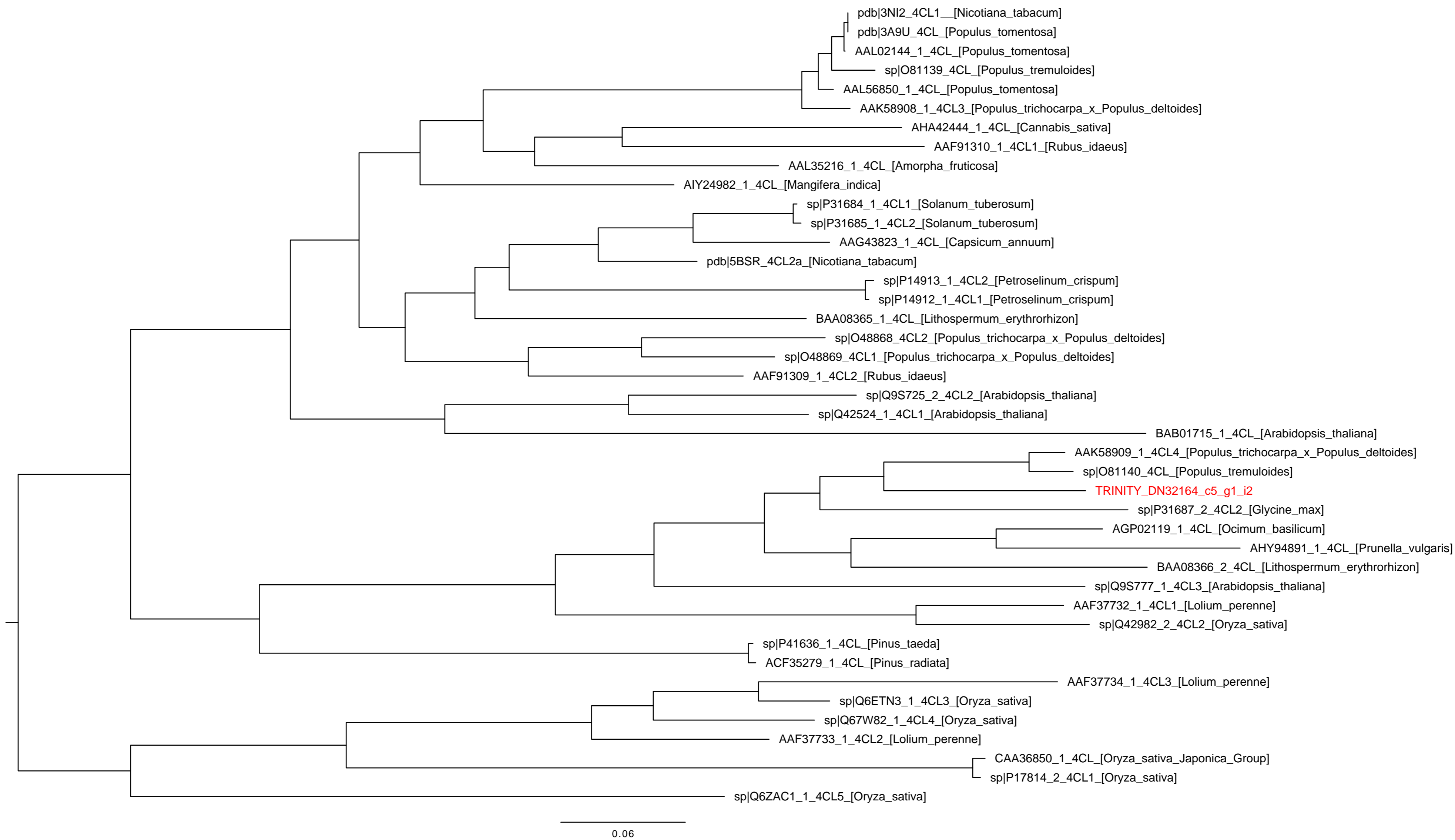

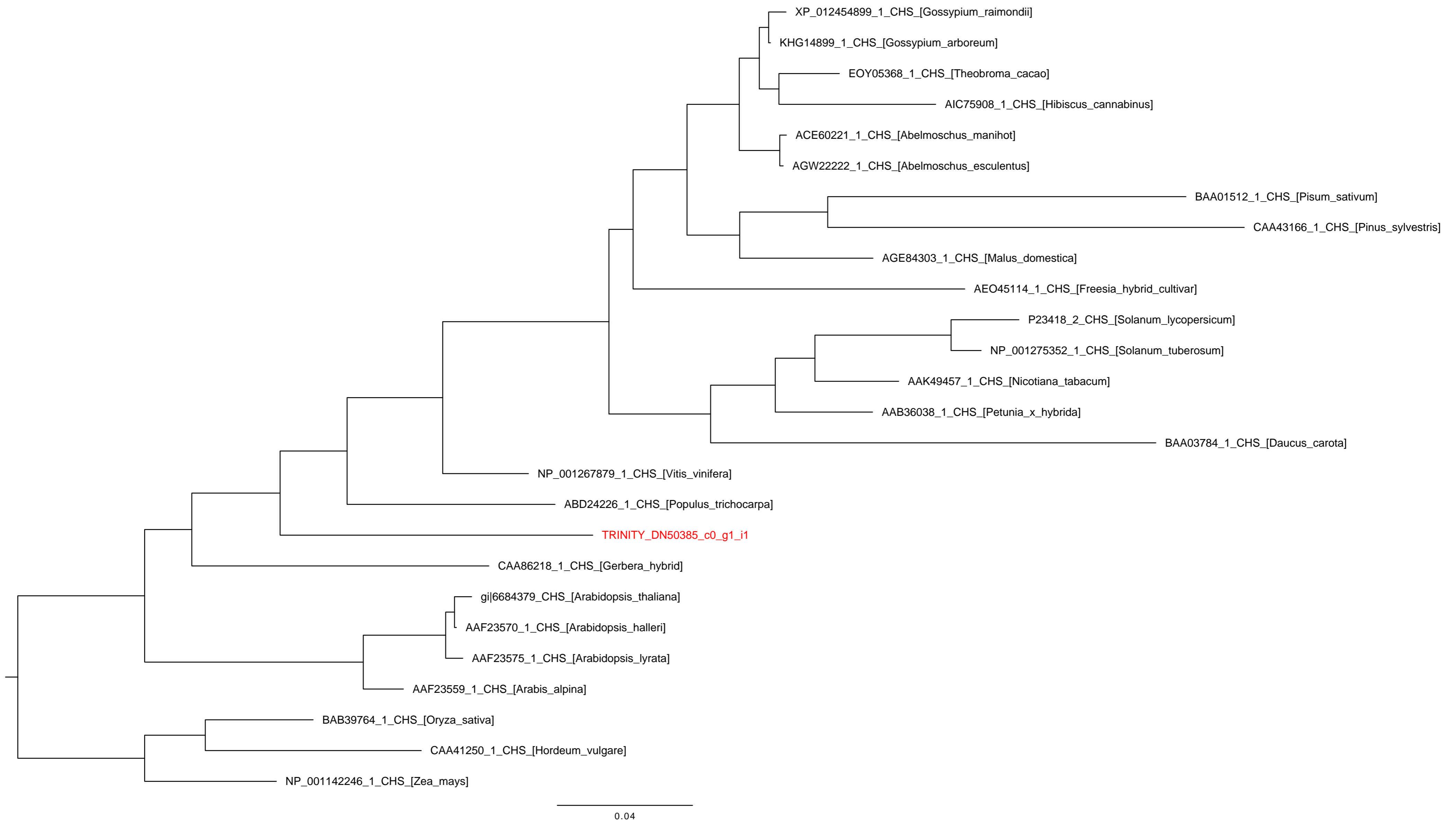

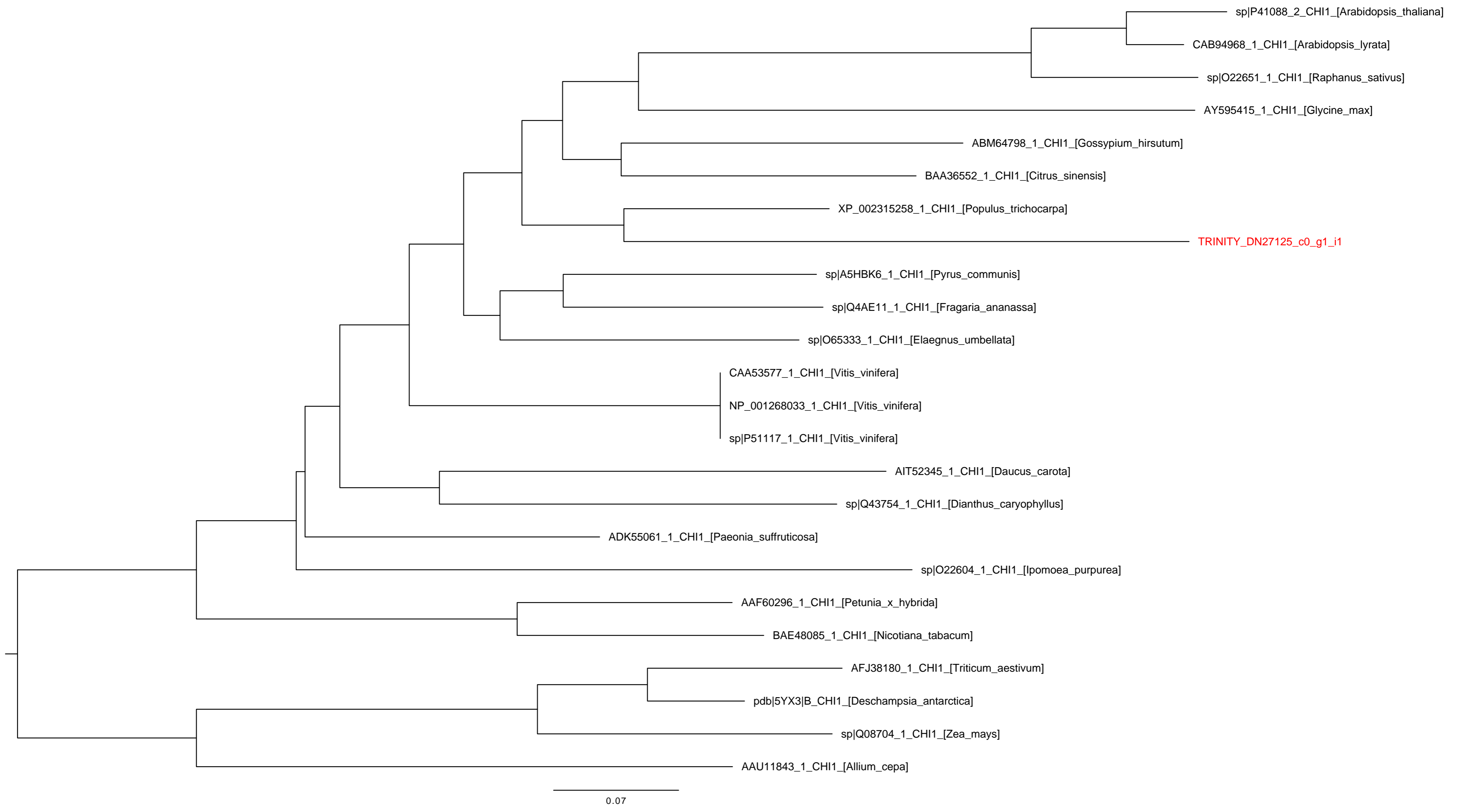

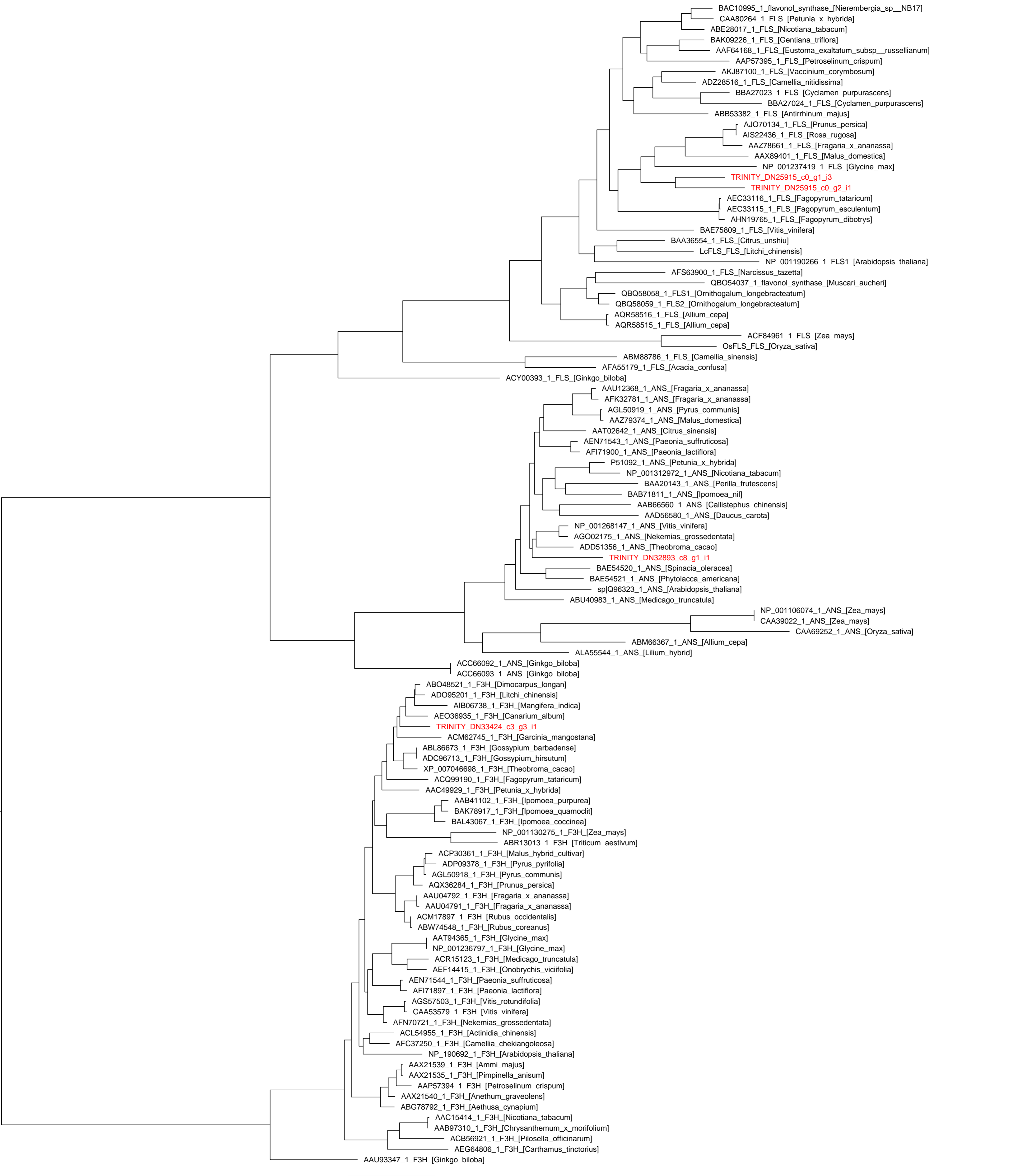

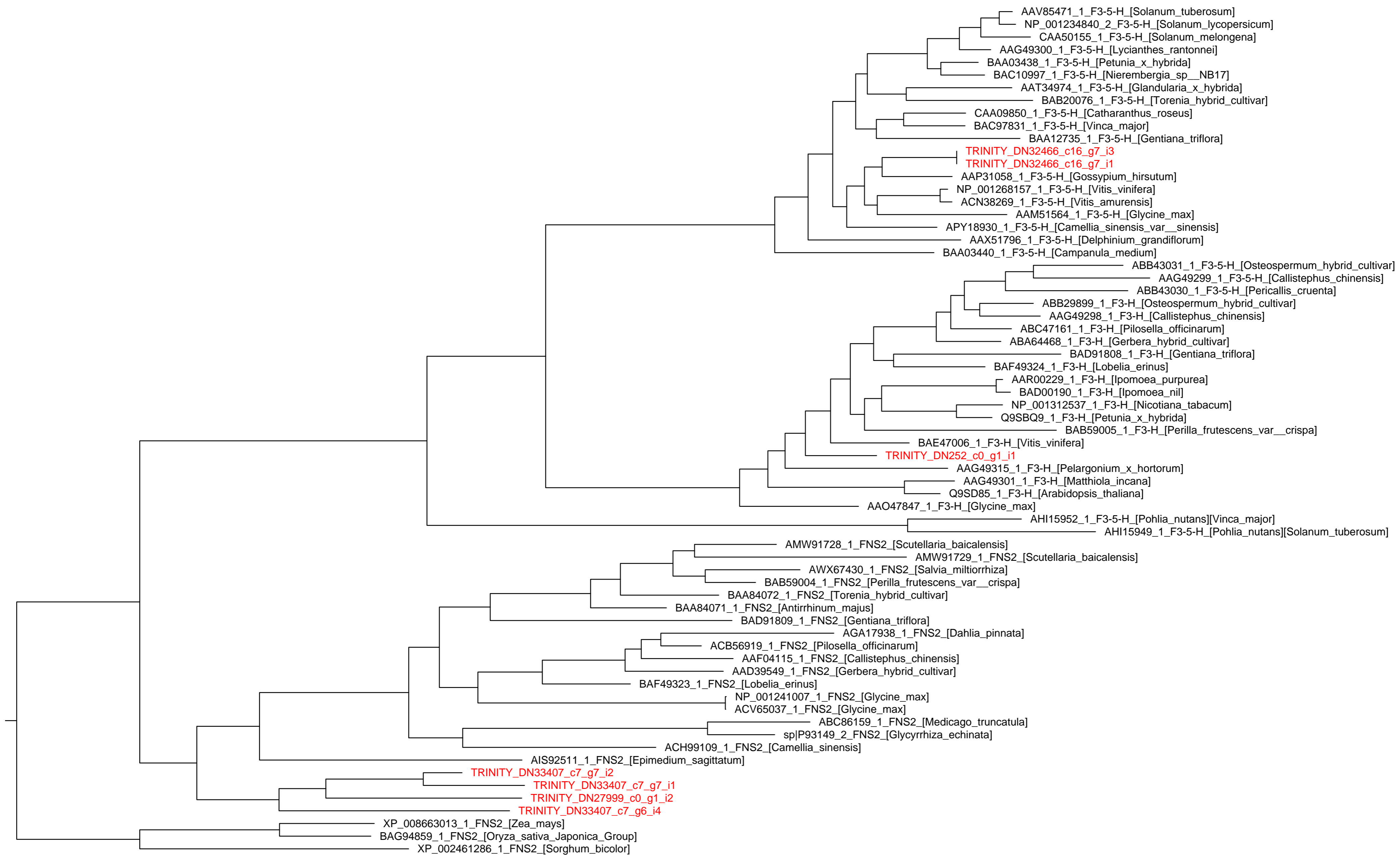

0.2

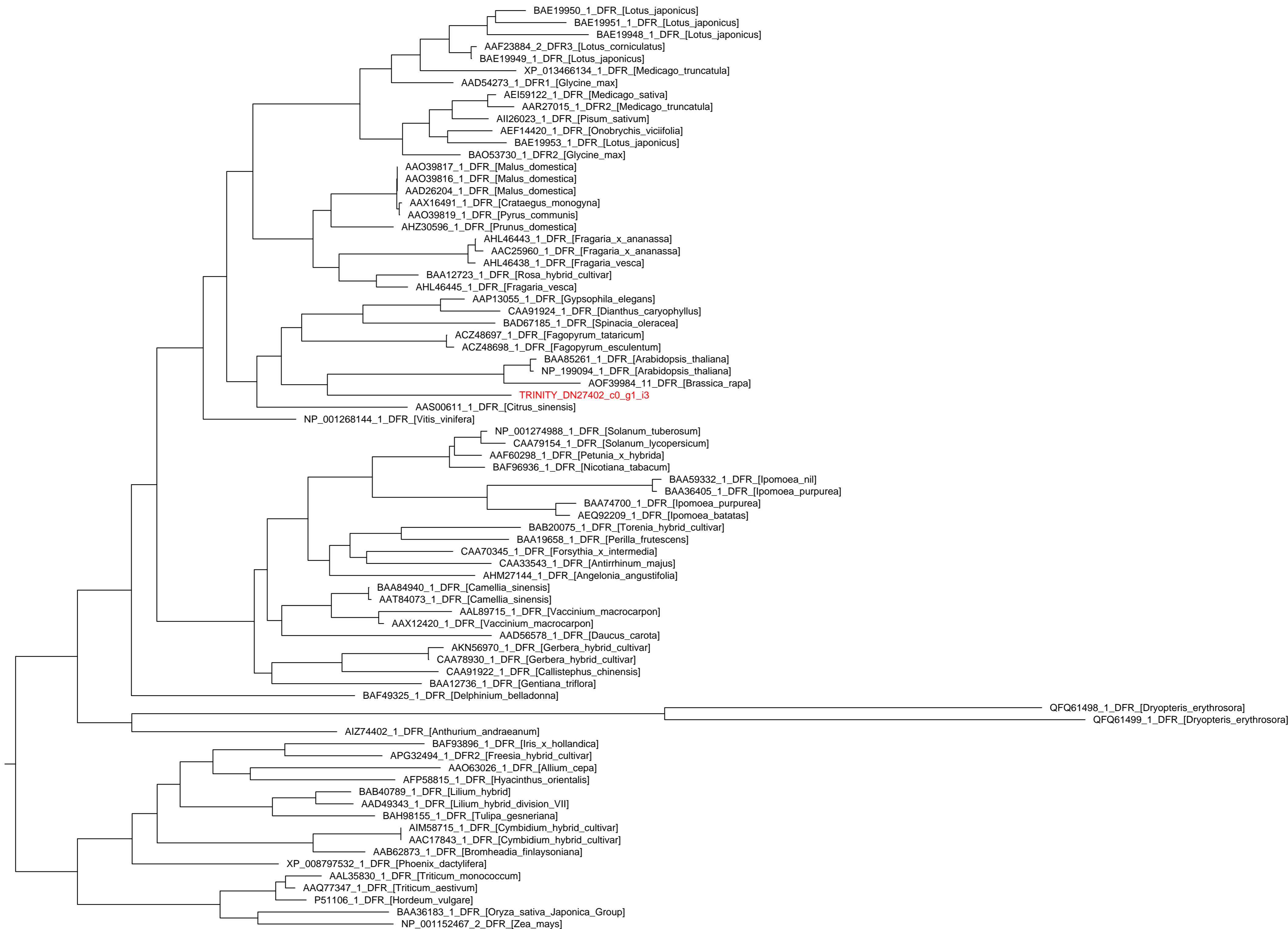

0.2

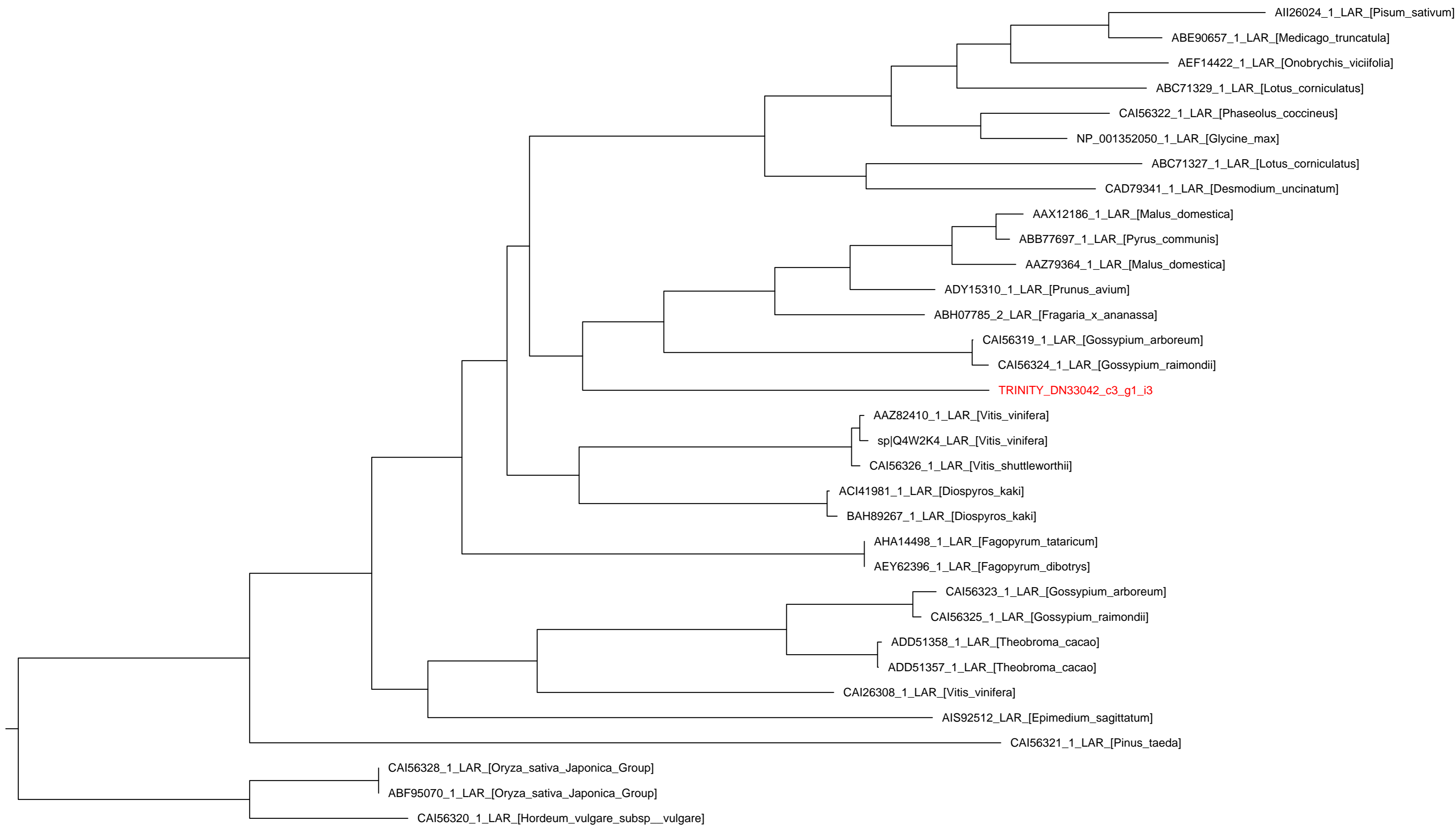

0.09

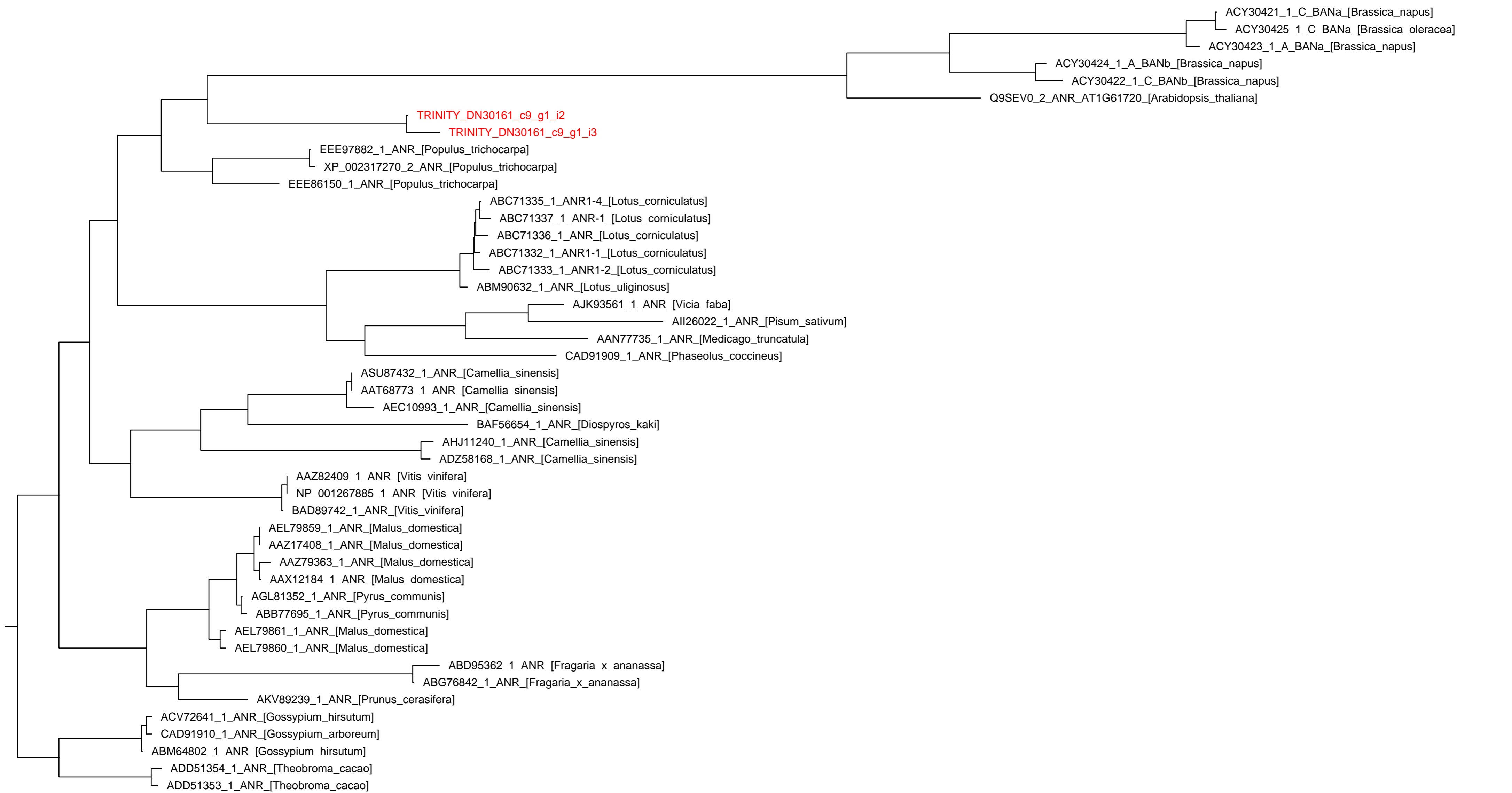

0.07

Supplement: Supplementary file 1 [file plants-09-01103-s001.zip › 20200724_supplements/File_S2.pdf]
