# Supplementary material for: Automatic Identification of Players in the Flavonoid Biosynthesis with Application on the Biomedicinal Plant Croton tiglium
Source: Plants (Basel). 2020 Aug 27;9(9):1103. doi: 10.3390/plants9091103 (PMC7570183; doi:10.3390/plants9091103)
Supplement: Supplementary file 1 [file File_S7.pdf › 20200724_supplements/File_S7.pdf]

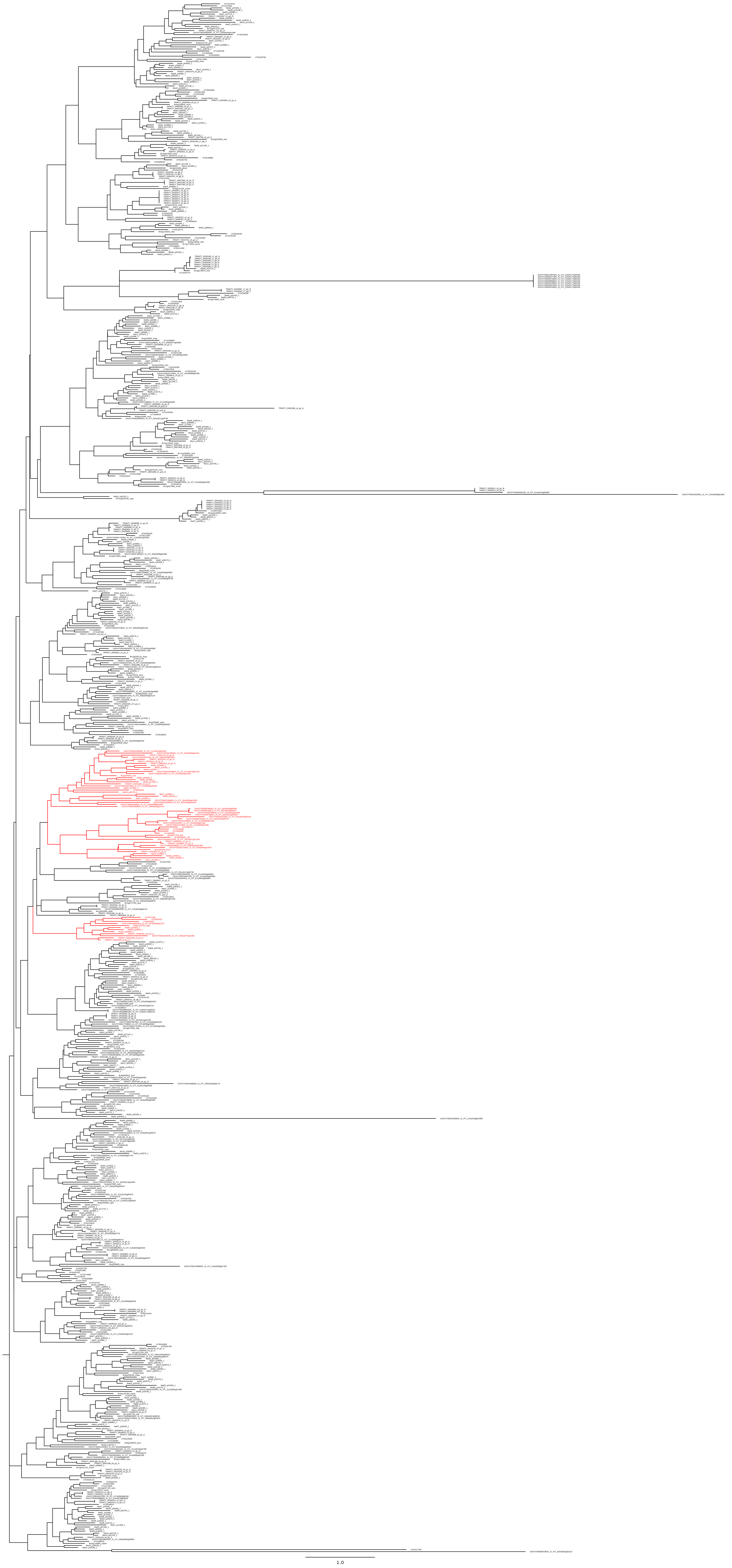
1.0

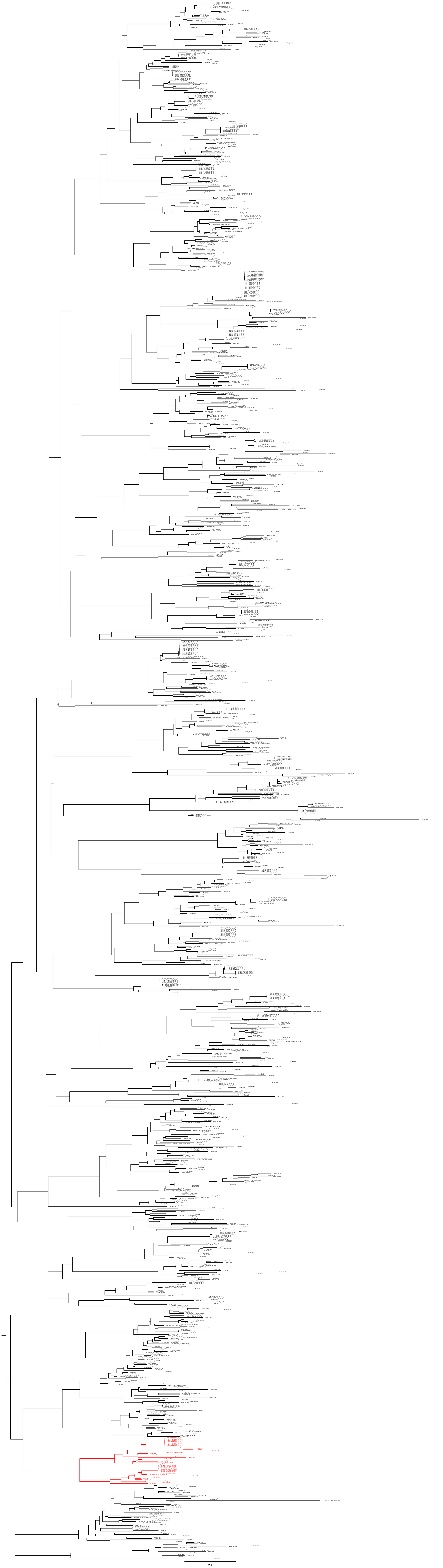
0.5

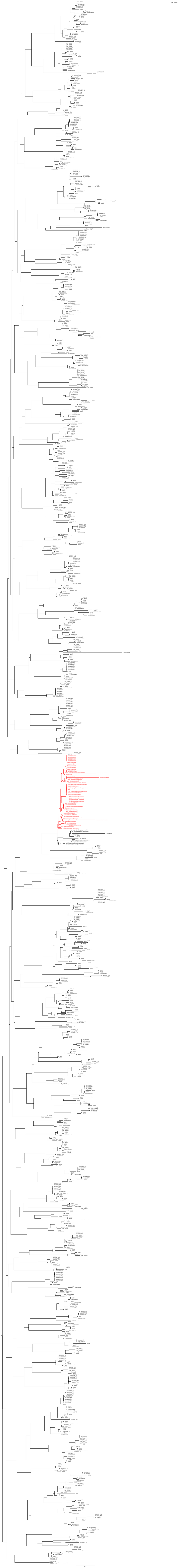
0.6
